# Identification of a key environment-responsive gene mediating environmental impact on postmenopausal osteoporosis

**DOI:** 10.3389/fpubh.2025.1536851

**Published:** 2025-03-27

**Authors:** Baicheng Wan, Junhong Zhou, Yilin Teng, Ye Tong, Shaohui Zong

**Affiliations:** ^1^Department of Spine Osteopathic, The First Affiliated Hospital of Guangxi Medical University, Nanning, China; ^2^Wuming Hospital of Guangxi Medical University, Nanning, China

**Keywords:** environment-responsive gene, postmenopausal women, osteoporosis, AKT1, BPA

## Abstract

**Background:**

Osteoporosis is a multifactorial disease influenced by genetic, environmental, and metabolic factors. AKT serine/threonine kinase 1 (AKT1), a central regulator of cellular survival and metabolism, has been implicated in bone remodeling, yet its precise role in osteoporosis remains unclear.

**Methodology:**

Gene expression analysis and molecular docking simulations were performed to identify key pathways and interactions involving bisphenol A (BPA) and AKT1. Molecular dynamics simulations further assessed the stability of BPA-AKT1 binding. Experimental validation was conducted using bone marrow-derived macrophages (BMMs) treated with BPA. Osteoclastogenesis was evaluated through TRAcP staining, and AKT1 expression was analyzed via real-time PCR. Protein-level validation of AKT1 phosphorylation was performed using Western blot analysis to confirm its activation during osteoclast differentiation.

**Results:**

Computational analyses identified AKT1 as a key mediator linking BPA exposure to bone remodeling pathways. Molecular docking revealed strong interactions between BPA and AKT1, supported by molecular dynamics simulations showing stable binding. Experimental assays demonstrated that BPA significantly enhanced RANKL-mediated osteoclastogenesis, upregulated AKT1 mRNA expression, and promoted AKT1 phosphorylation. These findings indicate that BPA promotes bone resorption through AKT1 activation, potentially contributing to osteoporosis pathogenesis.

**Conclusion:**

AKT1 emerges as a critical node connecting environmental pollutants like BPA to bone health, highlighting its potential as a therapeutic target. These findings underscore the complexity of osteoporosis pathophysiology and the importance of mitigating environmental pollutant exposure.

## 1 Introduction

Osteoporosis, a common metabolic bone disease, is marked by reduced bone mass and structural deterioration, leading to an elevated risk of fractures, particularly in postmenopausal women (1). Recent research highlights the significant role of environmental factors, alongside genetic and hormonal influences, in osteoporosis pathogenesis (2). Specifically, exposure to environmental pollutants and endocrine-disrupting chemicals (EDCs) has been increasingly associated with adverse effects on bone health, potentially exacerbating osteoporosis through largely unexplored molecular mechanism (2, 3).

AKT1, also known as AKT serine/threonine kinase 1, is a key regulator of multiple cellular processes, including cell proliferation, survival, and metabolism (4). AKT1’s role in bone remodeling is of particular interest, as it modulates osteoblast and osteoclast activities that are essential for maintaining bone density and strength (5). Dysregulated AKT1 signaling has been linked to numerous diseases, and emerging studies suggest it may also contribute to osteoporosis pathophysiology. Despite these insights, the specific interactions between AKT1 and environmental pollutants that might influence bone metabolism have yet to be fully elucidated. The comparative toxicogenomics database (CTD) has cataloged numerous gene–environment interactions, providing a unique opportunity to explore the molecular effects of environmental pollutants on gene activity. By leveraging CTD data, this study aims to identify AKT1-related environmental response genes that may contribute to osteoporosis progression. Key chemicals known to interact with AKT1, such as bisphenol A (BPA), doxorubicin, and estradiol, are examined for their impact on AKT1 phosphorylation—a post-translational modification critical for its functional activity.

Our approach integrates multiple analytic techniques: differential gene expression analysis to identify osteoporosis-associated gene sets, followed by molecular docking and molecular dynamics (MD) simulations to explore the interactions between AKT1 and selected chemicals at an atomic level. Gene set enrichment analysis (GSEA) provided insight into enriched signaling pathways potentially affected by these interactions, while MD simulations were employed to examine binding stability and structural dynamics of AKT1-ligand complexes. In sum, this study seeks to clarify the molecular mechanisms by which AKT1 interacts with environmental pollutants, enhancing our understanding of how such exposures may exacerbate osteoporosis risk.

## 2 Methods

### 2.1 Data collection

This study utilized three publicly available datasets from the Gene Expression Omnibus (GEO) database^1^ to investigate molecular signatures associated with bone mineral density (BMD) and postmenopausal osteoporosis (PMO). GSE56815 includes circulating monocyte samples from 80 individuals, of which 40 postmenopausal women (20 with high BMD and 20 with low BMD) were analyzed to focus on the population most affected by PMO. GSE62402 comprises peripheral blood monocyte samples from 10 female participants (5 with high BMD and 5 with low BMD), although menopausal status is not specified, representing a limitation in its applicability to PMO-specific analyses. GSE230665 features femur tissue samples collected during hip arthroplasty from 12 postmenopausal osteoporosis patients and 3 healthy postmenopausal controls, providing direct insights into bone-specific molecular changes. These datasets, derived from *Homo sapiens*, were selected based on their relevance to BMD phenotypes, biological sample types pertinent to bone remodeling, and stratified data availability. Detailed sample characteristics are provided in Supplementary Table S1.

### 2.2 Differential gene expression analysis

To investigate molecular differences related to osteoporosis, differential expression analysis was conducted on selected subsets from the GEO datasets using the limma package (6) in R software. A subset of 40 postmenopausal women from GSE56815, categorized into high and low BMD groups, was analyzed to identify differentially expressed genes (DEGs). Similarly, the GSE62402 dataset, containing peripheral blood monocyte samples from individuals in high and low BMD groups, was examined. The thresholds for DEG selection were set at *p* < 0.05 and |logFC| > 0.2 to balance the detection of biologically meaningful changes and the inclusion of a broader gene set for exploratory analyses. This choice reflects the exploratory nature of the study, aiming to capture subtle yet potentially significant changes in gene expression, which are particularly relevant in chronic metabolic diseases like osteoporosis. Using a less stringent threshold (*p* < 0.05) without adjustment for multiple comparisons increases the risk of false positives. However, applying adjusted *p*-values significantly reduced the number of DEGs, potentially excluding biologically relevant genes. To enhance the robustness of the findings, we performed an overlap analysis to identify genes consistently upregulated or downregulated across GSE56815 and GSE62402, highlighting key molecular signatures associated with BMD variation. These intersecting DEGs were further validated using the independent GSE230665 dataset, focusing on femur tissue samples, to ensure consistency across sample types and datasets.

### 2.3 KEGG pathway enrichment analysis

To investigate the biological pathways associated with osteoporosis-related gene expression changes, we conducted Kyoto Encyclopedia of Genes and Genomes (KEGG) pathway enrichment analysis using Gene Set Enrichment Analysis (GSEA). This approach allows for the evaluation of predefined gene sets across the entire ranked list of genes, avoiding the need for arbitrary thresholds in defining differentially expressed genes (DEGs). Separate analyses were performed for genes upregulated and downregulated in the GSE56815 and GSE62402 datasets. The enrichment analysis was implemented using the gseKEGG function from the clusterProfiler package (7) in R, which evaluates pathway significance through permutation testing and adjusts *p*-values using the Benjamini-Hochberg (BH) method to control the false discovery rate (FDR). Pathways with FDR-adjusted *p*-values < 0.05 were considered significantly enriched. This approach ensures robust identification of key pathways underlying osteoporosis pathophysiology while minimizing biases associated with DEG selection.

### 2.4 Integration of CTD data and identification of environment-responsive genes

The Comparative Toxicogenomics Database (CTD^2^) was leveraged to obtain comprehensive data on genes and environmental pollutants associated with the progression of postmenopausal osteoporosis (PMO). The CTD integrates manually curated information on chemical-gene-disease interactions, providing a valuable resource for exploring molecular mechanisms influenced by environmental exposures (8). From the CTD, we selected genes with an inference score > 25 to identify key targets and chemicals potentially associated with PMO. By intersecting these CTD-derived genes with the DEGs identified in our study, we pinpointed a subset of overlapping genes termed key environment-responsive genes (KERGs). These KERGs represent critical molecular targets influenced by environmental pollutants, providing a robust framework for understanding their role in PMO pathogenesis. To further explore the environmental factors influencing these KERGs, we searched for associated chemicals in CTD. We applied an additional selection criterion of reference count >10 to ensure the reliability of chemical-gene interactions. Only chemicals meeting this threshold were considered for network construction. Subsequently, we developed a disease-gene-chemical interaction network to visualize the relationships between PMO, identified genes, and environmental pollutants.

### 2.5 Molecular docking and molecular dynamics simulation

To further explore the interaction between environmental pollutants and the identified KERGs, molecular docking and molecular dynamics simulations were performed. The molecular docking was conducted using AutoDock Vina (version 1.5.6) to predict the binding affinity between selected chemicals and the KERGs. The three-dimensional (3D) structures of the chemicals were obtained from the PubChem database,^3^ while the protein structure of KERGs was retrieved from the Protein Data Bank (PDB)^4^ (9). Docking results were assessed based on binding energy, with values below −5.0 kcal/mol considered indicative of favorable interactions. Molecular dynamics (MD) simulations were carried out for the most promising chemical-KERG complexes using GROMACS software (version 2020.6-MODIFIED) with the *Amber99sb-ildn* force field. The simulations employed a TIP3P water model to accurately represent the aqueous environment, and periodic boundary conditions were applied to mimic physiological conditions. The prepared system underwent energy minimization to eliminate unfavorable contacts and optimize the initial structure. This was followed by equilibration in two stages: first under an NVT ensemble to stabilize the temperature, and subsequently under an NPT ensemble to ensure pressure stability. A 100 ns production run was then performed to observe the stability and dynamic behavior of the complexes. To analyze the stability of the chemical-KERG interactions, we analyzed three key parameters: root mean square deviation (RMSD) to monitor overall structural stability throughout the simulation, root mean square fluctuation (RMSF) to assess residue-level flexibility and identify regions with greater structural fluctuations, and radius of gyration (Rg) to determine the compactness of the protein-ligand complex, offering insights into both structural stability and conformational changes. In addition to these stability metrics, we generated a 3D free energy landscape (FEL) to visualize the conformational states and free energy minima of the complexes during the simulation.

### 2.6 Osteoclast culture and cytotoxicity assay

Bisphenol A (BPA; CAS: 80–05-7, molecular formula: C₁₅H₁₆O₂, HY-18260, purity: 99.95%) was purchased from Med Chem Express (New Jersey, United States). A 1 mM stock solution was prepared in DMSO and subsequently diluted in culture medium to achieve the desired working concentrations. Bone marrow-derived macrophages (BMMs) were isolated from the tibias and femurs of 4-to 5-week-old C57BL/6 J mice. Bone marrow cells were flushed out and cultured in α-MEM complete medium supplemented with 10% fetal bovine serum (FBS), 1% penicillin–streptomycin, and 30 ng/mL macrophage colony-stimulating factor (M-CSF). Osteoclast differentiation was induced by treating BMMs with α-MEM complete medium containing 50 ng/mL receptor activator of nuclear factor kappa-B ligand (RANKL) and 30 ng/mL M-CSF, with RANKL-containing medium replaced every 2 days. Cytotoxicity was assessed by culturing BMMs in 96-well plates with complete medium containing various concentrations of BPA (0, 5, 10, 15, 20, and 25 μM) for 24 or 48 h. After the treatment period, CCK-8 reagent was added to each well, followed by incubation for 60 min. Absorbance at 450 nm was measured using a microplate reader to evaluate cell viability.

### 2.7 TRAcP staining and RT-qPCR

The concentration of BPA (20 μM) used in the intervention group was determined based on the cytotoxicity assay results, which confirmed that this dose was non-cytotoxic to bone marrow-derived macrophages (BMMs) while allowing for the evaluation of its biological effects on osteoclastogenesis. Osteoclast differentiation was analyzed using tartrate-resistant acid phosphatase (TRAcP) staining. Following fixation in 4% paraformaldehyde for 10 min, cells were stained for TRAcP and visualized using the EVOS FL Auto 2 imaging system (Thermo Fisher Scientific, United States). Quantification of TRAcP-positive multinucleated osteoclasts, including their number and size, was performed using ImageJ (v1.8.0) to assess the extent of osteoclastogenesis. To investigate the molecular effects of BPA on AKT1 expression, RNA was extracted from BMMs treated with BPA (20 μM) during the osteoclast differentiation process. Total RNA was isolated using Trizol reagent, and complementary DNA (cDNA) was synthesized according to the manufacturer’s instructions. RT-qPCR was conducted using the LightCycler 480 system (Roche, Switzerland) to measure the expression levels of AKT1, with GAPDH serving as an internal control. Relative expression levels were calculated using the ΔΔCt method. The primer sequences were: AKT1, 5′-TGTATGAGAAGAAGCTGAGCCC-3′ (Forward), 5′-AGTAGGAGAACTTGATCAGGCG-3′ (Reverse); GAPDH, 5′-CTCAGGAGAGTGTTTCCTCGTC-3′ (Forward), 5′-CCTTGACTGTGCCGTTGAATTT-3′ (Reverse).

### 2.8 Protein extraction and western blot analysis

After washing the cells with PBS, they were lysed in RIPA buffer containing 1% PMSF and 1% protease/phosphatase inhibitors. The lysates were then centrifuged at low temperature to extract total protein. Next, the protein samples were separated on a 10% SDS-PAGE gel at a constant voltage of 120 V for 60 min and transferred onto a PVDF membrane. The membrane was blocked with 5% skim milk for 90 min. Subsequently, the membrane was incubated at 4°C for 14 h with primary antibodies specific for p-AKT (Proteintech Group, 66444-1-Ig), AKT (Proteintech Group, 10176-2-AP), and GAPDH (Proteintech Group, 60004-1-Ig). This was followed by a 60-min incubation at room temperature with a fluorescently labeled secondary antibody (Goat anti-Rabbit IgG (H + L), Thermo Fisher Scientific, SA5-35571). Finally, the membrane was visualized using an infrared fluorescence imaging system (LI-COR Biosciences, United States), and band intensities were quantified with ImageJ (v1.8.0).

## 3 Results

### 3.1 Differential expressed genes

**Figure 1:**
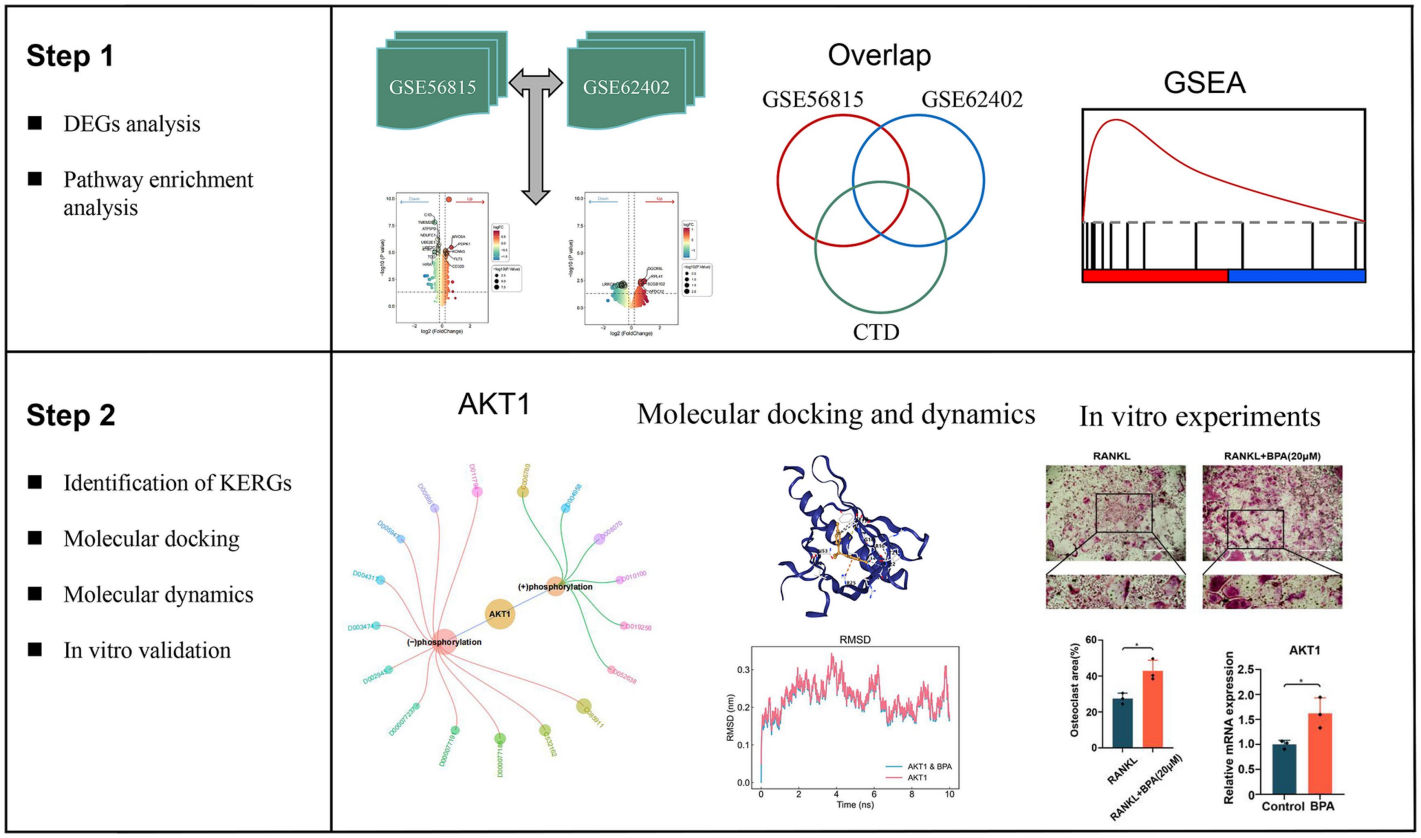
Overview of the study workflow detailing the step-by-step analysis used to identify key environment-responsive genes (KERGs) and their role in osteoporosis. The workflow begins with differential expression analysis (DEGs) performed on datasets GSE56815 and GSE62402, followed by overlap analysis using the Comparative Toxicogenomics Database (CTD) to identify KERGs. Gene set enrichment analysis (GSEA) was conducted to identify significantly enriched pathways associated with DEGs. The identified KERGs, including AKT1, were further evaluated through molecular docking and molecular dynamics simulations to assess their interactions with bisphenol A (BPA). Finally, *in vitro* experiments using bone marrow-derived macrophages were conducted to validate the molecular findings, including AKT1 expression and phosphorylation, as well as BPA’s effect on osteoclastogenesis. DEGs, Differentially expressed genes; CTD, Comparative Toxicogenomics Database; KERGs, Key environment-responsive genes; GSEA, Gene set enrichment analysis; AKT1, AKT serine/threonine kinase 1; BPA, Bisphenol A.

**Figure 2:**
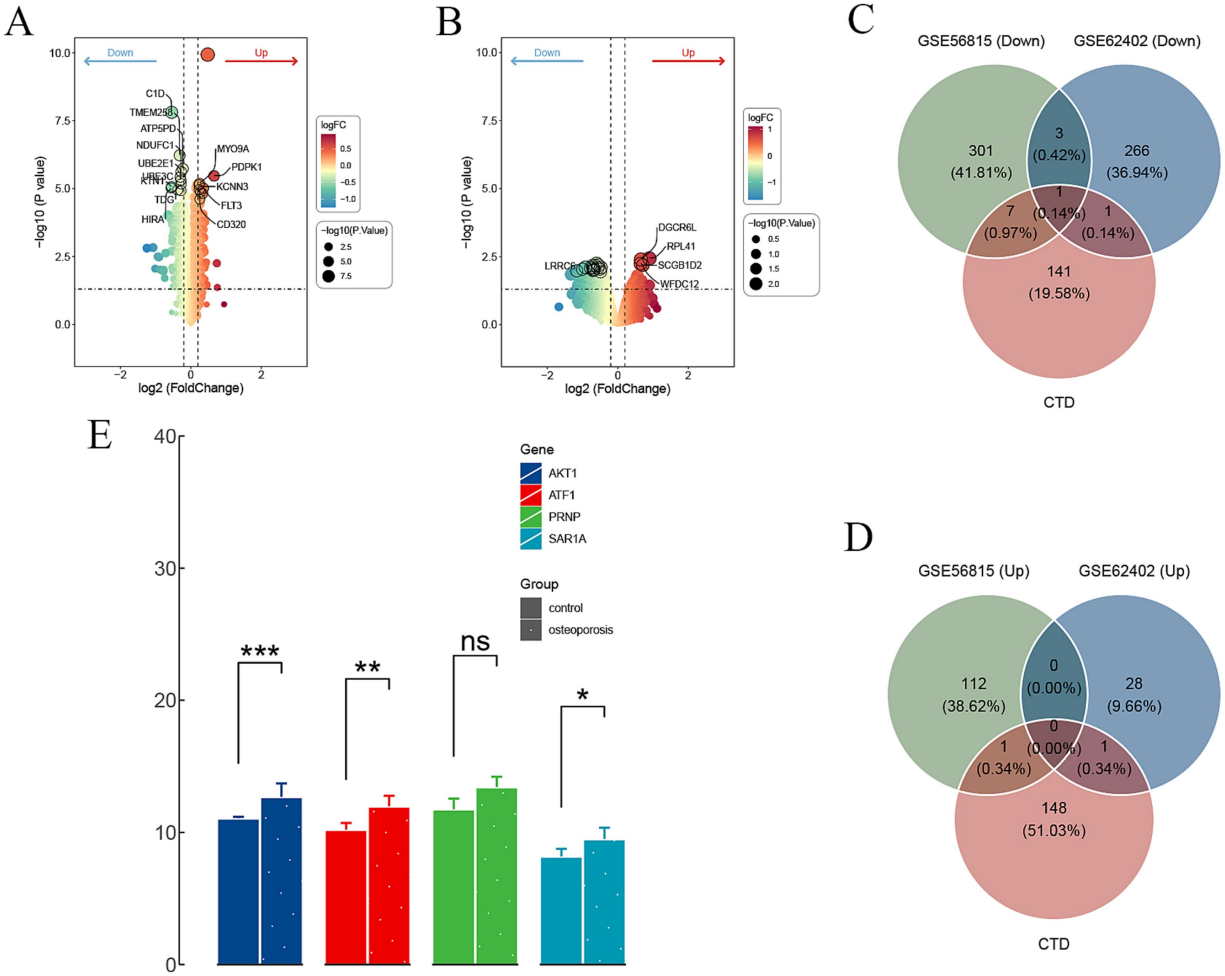
Differential gene expression and overlap analysis of KERGs. **(A,B)** Volcano plots depicting DEGs in datasets GSE56815 and GSE62402, respectively. **(C,D)** Venn diagrams illustrating the overlap between downregulated **(C)** and upregulated **(D)** DEGs from GSE56815, GSE62402, and CTD-derived genes. **(E)** Expression levels of selected downregulated KERGs in control and osteoporosis groups (*p < 0.05, **p < 0.01, ***p < 0.001, ns = not significant). DEGs, Differentially expressed genes; CTD, Comparative Toxicogenomics Database; KERGs, Key environment-responsive genes; AKT1, AKT serine/threonine kinase 1.

The comprehensive data screening strategy is outlined in the flowchart presented in Figure 1. From the GSE56815 dataset, which includes 40 postmenopausal women categorized into high and low BMD groups, a total of 425 DEGs were identified. Among these DEGs, 113 genes were found to be upregulated, while 312 genes were downregulated in the high BMD group compared to the low BMD group (Figure 2A). Similarly, the analysis of the GSE62402 dataset, consisting of peripheral blood monocyte samples from individuals with high and low BMD, identified 300 DEGs, with 29 genes upregulated and 271 genes downregulated in the high BMD group (Figure 2B). A total of four genes—AKT1, ATF1, PRNP, and SAR1A—were consistently downregulated across both datasets, indicating robust and reproducible molecular signatures related to BMD regulation (Figure 2C). Notably, no overlapping genes were identified in the upregulated category between the two datasets (Figure 2D). The expression levels of these four downregulated genes were further validated using the independent GSE230665 dataset, which contains clinical samples from PMO patients and healthy controls. As shown in the Figure 2E, the results confirmed the downregulation of AKT1, ATF1, PRNP, and SAR1A in osteoporosis samples compared to controls. Specifically, AKT1 and ATF1 showed statistically significant reductions in expression (*p* < 0.001 and *p* < 0.01, respectively), while PRNP exhibited no significant difference. SAR1A showed a moderate but statistically significant reduction in expression (*p* < 0.05).

### 3.2 Pathway enrichment analysis

**Figure 3:**
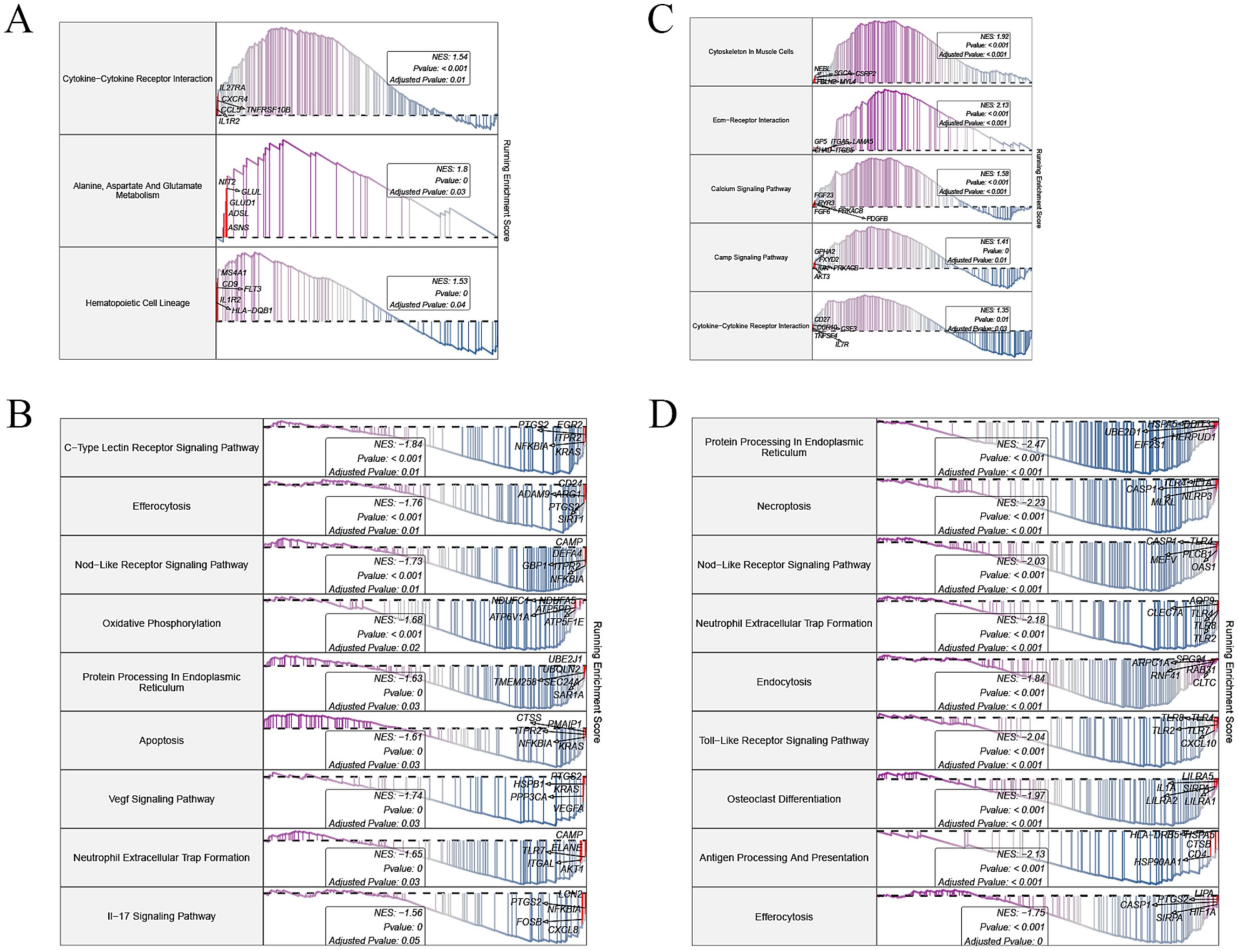
GSEA of differentially expressed genes in osteoporosis-related datasets. **(A,B)** Enriched KEGG pathways in the activated **(A)** and inhibited **(B)** gene sets from GSE56815. **(C,D)** Enriched KEGG pathways in the activated **(C)** and inhibited **(D)** gene sets from GSE62402. GSEA, Gene set enrichment analysis; KEGG, Kyoto Encyclopedia of Genes and Genomes.

The KEGG pathway enrichment analysis, performed using GSEA, provided insights into key biological processes associated with BMD variation. DEGs were ranked by logFC to generate gene lists, with enrichment direction reflecting the relative activity in the high BMD group compared to the low BMD (control) group. Both datasets revealed several unique pathways, as well as overlapping pathways, that were consistently activated or suppressed in the high BMD group. Specifically, in GSE56815, pathways such as cytokine-cytokine receptor interaction (NES = 1.54, adjusted *p*-value = 0.01) and alanine, aspartate, and glutamate metabolism (NES = 1.8, adjusted *p*-value = 0.03) were activated, indicating enhanced immune signaling and amino acid metabolism (Figure 3A). Conversely, oxidative phosphorylation (NES = −1.68, adjusted *p*-value = 0.02) and apoptosis (NES = −1.61, adjusted *p*-value = 0.03) were suppressed, reflecting decreased mitochondrial activity and apoptotic signaling (Figure 3B). In GSE62402, pathways including cytoskeleton in muscle cells (NES = 1.92, adjusted *p*-value < 0.001) and ECM-receptor interaction (NES = 2.13, adjusted *p*-value < 0.001) showed activation in the high BMD group, highlighting increased cellular structural reorganization (Figure 3C). Immune-related pathways such as Toll-like receptor signaling (NES = −2.04, adjusted *p*-value < 0.001) and neutrophil extracellular trap formation (NES = −2.18, adjusted *p*-value < 0.001) were suppressed, indicating a dampened inflammatory response (Figure 3D). Crucially, two pathways were consistently regulated across both datasets. The cytokine−cytokine receptor interaction pathway was activated in the high BMD group in both datasets, highlighting its essential role in immune signaling and bone remodeling. Conversely, the efferocytosis pathway was suppressed across both datasets, indicating a potential reduction in the clearance of apoptotic cells, which may contribute to changes in the bone microenvironment.

### 3.3 Identification of KERGs and chemical interactions

From the CTD, we retrieved a total of 16,758 data points linking genes and environmental pollutants to PMO (Supplementary Table S2). Using an inference score > 25 as the threshold, 150 genes associated with environmental chemicals influencing PMO were identified for further analysis. To pinpoint key molecular targets, these 150 genes were intersected with the DEGs identified from the GSE56815 and GSE62402 datasets. This intersection yielded a single overlapping gene, AKT1, which we identified as the KERG (Figures 2C,D). The identification of AKT1 suggests that it may serve as a critical molecular link between environmental pollutants and BMD regulation. To investigate the potential environmental modulators of AKT1, we filtered chemicals from the CTD using a reference count threshold of >10. The interactions were categorized into two primary groups: chemicals that increase AKT1 phosphorylation and those that decrease AKT1 phosphorylation. Several chemicals, such as bisphenol A (BPA) and lipopolysaccharides (LPS), were identified as increasing AKT1 phosphorylation, with BPA having a notable reference count of 24. Conversely, compounds like resveratrol and doxorubicin were linked to decreased AKT1 phosphorylation, suggesting an inhibitory influence on AKT1 activity. Interestingly, two chemicals, hydrogen peroxide and BPA, exhibited dual actions, capable of both increasing and decreasing AKT1 phosphorylation depending on the experimental context or biological conditions (Supplementary Figure S1; Supplementary Table S3). This duality highlights the complexity of AKT1 regulation and the context-dependent nature of environmental chemical interactions.

### 3.4 Molecular docking and molecular dynamics simulation results

**Figure 4:**
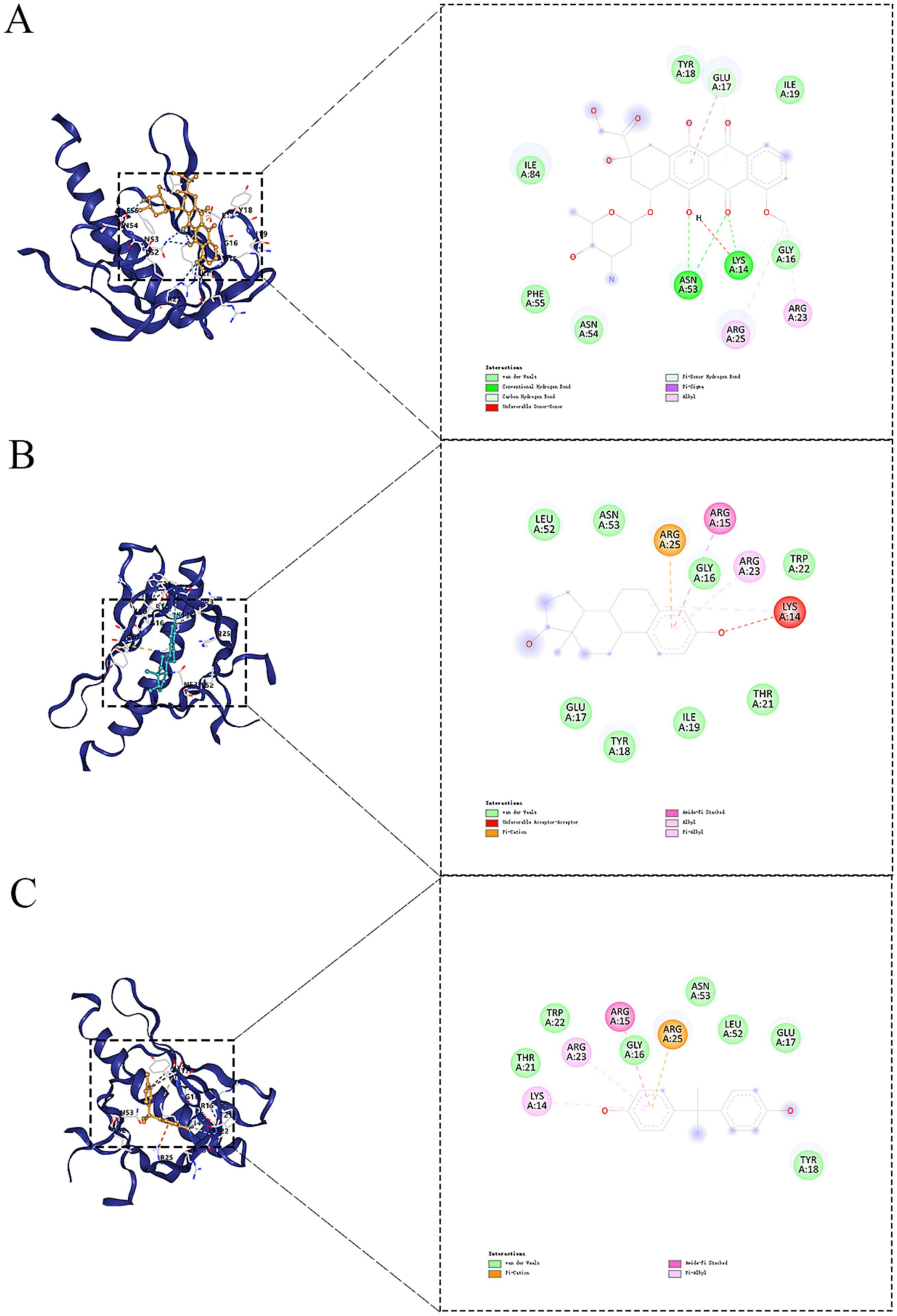
Molecular docking results of selected compounds with AKT1. **(A)** Doxorubicin binding pose within the AKT1 binding pocket. **(B)** Estradiol binding pose in the AKT1 binding site. **(C)** Docking pose of BPA with AKT1. AKT1, AKT serine/threonine kinase 1; BPA, Bisphenol A.

**Figure 5:**
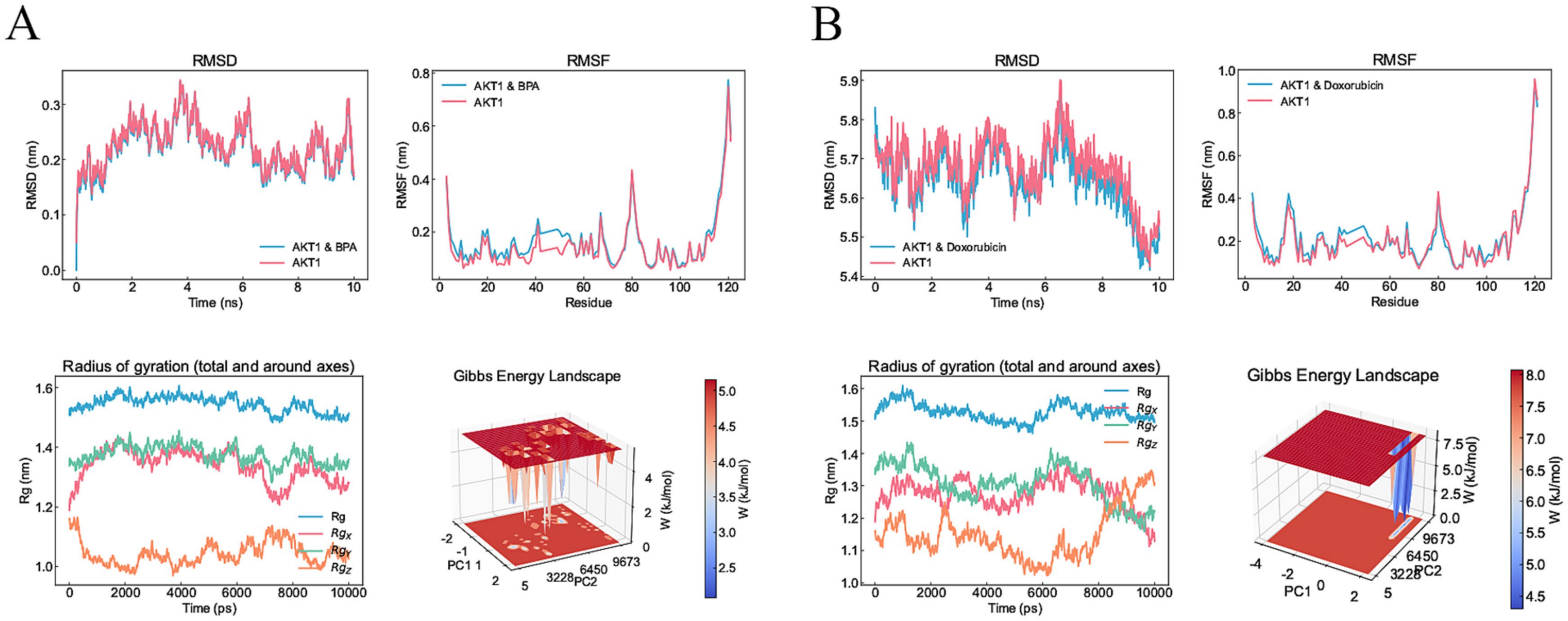
Molecular dynamics simulation results for BPA and doxorubicin binding to AKT1. **(A)** Molecular dynamics results for the AKT1-BPA complex. The RMSD plot shows structural stability of the AKT1-BPA complex compared to free AKT1 over a 10 ns simulation. The RMSF highlights localized flexibility in residues upon BPA binding. The Rg remains consistent, indicating compactness of the AKT1-BPA complex. The Gibbs free energy landscape reveals distinct energy minima, representing stable conformational states during the simulation. **(B)** Molecular dynamics results for the AKT1-doxorubicin complex. The RMSD plot demonstrates the stability of the AKT1-doxorubicin complex compared to free AKT1. RMSF analysis shows localized flexibility at binding residues. Rg analysis indicates structural compactness of the complex, while the Gibbs free energy landscape reveals well-defined energy minima, indicating strong binding and stable conformational states. RMSD, Root mean square deviation; RMSF, Root mean square fluctuation; Rg, Radius of Gyration; AKT1, AKT serine/threonine kinase 1; BPA, Bisphenol A.

From the interactions identified between KERGs and environmental chemicals, we selected compounds that were categorized as increasing AKT1 phosphorylation for molecular docking studies. Due to the unavailability of 3D structures in the PubChem database, LPS, cadmium chloride, and particulate matter were excluded from the analysis. As a result, four key compounds—BPA, hydrogen peroxide, doxorubicin, and estradiol—were used for detailed molecular docking. The docking results (Table 1) revealed varying binding energies, with doxorubicin showing the strongest binding affinity to AKT1 (−7.6 kcal/mol) (Figure 4A), followed by estradiol (−6.2 kcal/mol) (Figure 4B), BPA (−5.9 kcal/mol) (Figure 4C), and hydrogen peroxide (−2.4 kcal/mol). Given that hydrogen peroxide exhibited a relatively weak interaction, it was excluded from further consideration for dynamic studies. For molecular dynamics simulations, both BPA and doxorubicin were selected. BPA was included due to its significant environmental relevance as a widespread contaminant and endocrine disruptor, as well as its novel potential impact on AKT1 and bone health, areas with limited prior investigation. Doxorubicin, while primarily a chemotherapeutic agent, was included due to its strong binding affinity to AKT1 and its potential to provide insights into AKT1 modulation mechanisms. The inclusion of doxorubicin adds depth to the study by allowing a comparative analysis between an environmental pollutant and a pharmacological compound, thereby broadening the understanding of AKT1 interactions. Molecular dynamics simulations were conducted to analyze the interactions of BPA and doxorubicin with AKT1, providing insights into their stability and dynamic behavior. For BPA, the root mean square deviation (RMSD) analysis revealed stable binding throughout the simulation, with minimal fluctuations in the AKT1-BPA complex compared to the free AKT1 structure. The root mean square fluctuation (RMSF) showed localized flexibility, particularly at residues interacting with BPA, indicating structural adaptability to accommodate the ligand. The radius of gyration (Rg) remained relatively constant, suggesting compactness and stability of the protein-ligand complex. The Gibbs free energy landscape demonstrated well-defined energy minima, reflecting stable conformational states of the AKT1-BPA complex (Figure 5A). For doxorubicin, the RMSD showed slightly higher fluctuations compared to BPA, indicating dynamic interactions within the AKT1-doxorubicin complex. The RMSF analysis highlighted regions of flexibility at binding residues, which may contribute to the observed higher fluctuations. The Rg values suggested sustained structural integrity of the AKT1-doxorubicin complex over the simulation period. Notably, the Gibbs free energy landscape displayed deeper and more distinct energy minima compared to BPA, indicating stronger binding affinity and conformational stability of the AKT1-doxorubicin complex (Figure 5B).

**Table 1:** Binding energies and phosphorylation effects of selected compounds on AKT1.

Chemical	Interaction actions	Binding energy (kcal/mol)
Doxorubicin	Increases phosphorylation	−7.6
Estradiol	Increases phosphorylation	−6.2
Bisphenol A	Increases/decreases phosphorylation	−5.9
Hydrogen peroxide	Increases/decreases phosphorylation	−2.4

AKT1, AKT serine/threonine kinase 1.

### 3.5 BPA promotes RANKL-mediated osteoclastogenesis *in vitro* and increases AKT1 gene expression

**Figure 6:**
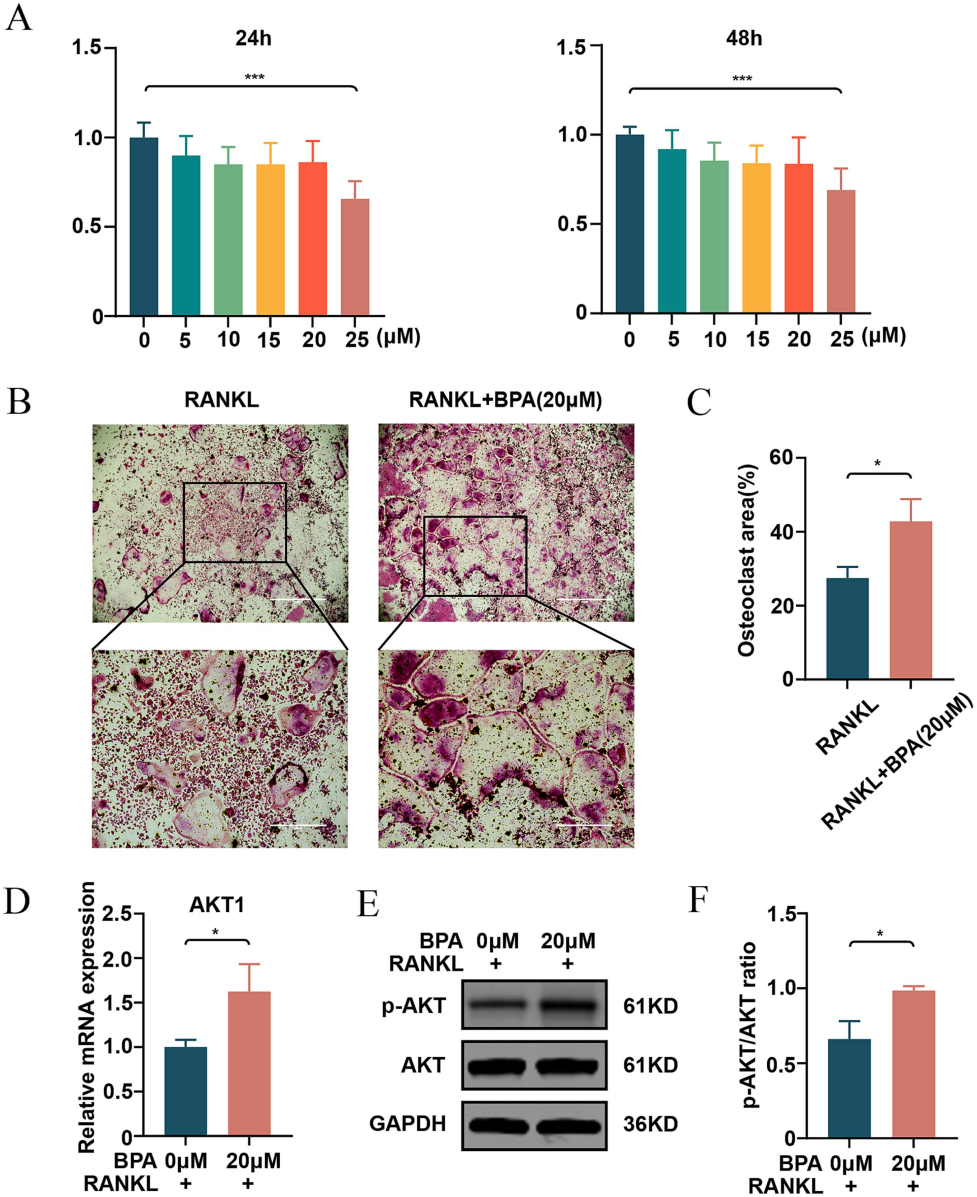
Effects of BPA on cytotoxicity, osteoclastogenesis, and AKT1 expression. **(A)** Cell viability of BMMs treated with increasing concentrations of BPA (0, 5, 10, 15, 20, and 25 μM) for 24 and 48 h, as assessed by CCK-8 assays. **(B)** Representative images of TRAcP-positive multinucleated osteoclasts formed in cultures treated with RANKL alone or in combination with BPA (20 μM). **(C)** Quantitative analysis of the percentage of osteoclast area in RANKL-only and BPA-treated groups. **(D)** Relative mRNA expression of AKT1 in BMMs treated with RANKL alone or in combination with BPA (20 μM), as determined by real-time PCR. **(E)** Western blot analysis of p-AKT and AKT protein levels in BMMs during osteoclast differentiation with or without BPA treatment. **(F)** Quantification of p-AKT relative to non-phosphorylated AKT (*p < 0.05, **p < 0.01, ***p < 0.001). Results are presented as mean ± SD (*n* = 3/group). BMMs, Bone marrow-derived macrophages; TRAcP, Tartrate-resistant acid phosphatase; AKT1, AKT serine/threonine kinase 1; p-AKT, Phosphorylated AKT; BPA, Bisphenol A.

Concentrations of BPA up to 20 μM maintained acceptable levels of cell viability, while 25 μM significantly reduced viability, indicating potential cytotoxicity at higher doses (Figure 6A). Based on these results, 20 μM was selected as the working concentration for subsequent experiments. Osteoclastogenesis assays revealed that BPA significantly enhanced the formation of TRAcP-positive multinucleated osteoclasts compared to the RANKL-only group. The size of osteoclasts was markedly increased in the BPA-treated group, as visualized by TRAcP staining (Figure 6B) and quantified as a higher percentage of osteoclast area (Figure 6C). At the molecular level, real-time PCR analysis demonstrated that BPA treatment significantly upregulated AKT1 mRNA expression in BMMs during osteoclast differentiation (Figure 6D). Western blot analysis further showed that, following RANKL induction, BPA notably promoted AKT phosphorylation (Figures 6E,F). These findings suggest that BPA promotes osteoclastogenesis and enhances AKT1 expression, indicating its potential role in driving bone resorption and contributing to osteoporosis pathogenesis.

## 4 Discussion

This study systematically identified and validated KERGs associated with postmenopausal osteoporosis, with a particular focus on AKT1, which appears to play a crucial role in bone metabolism regulation. Our differential gene expression analysis identified a distinct set of genes that were differentially expressed between high and low BMD groups, with AKT1 notably upregulated in osteoporosis patients. The observed overlap of DEGs across multiple datasets supports the robustness of our gene selection. Additionally, the validation of AKT1 expression in the GSE230665 dataset reinforces its potential importance as a regulatory factor in bone remodeling, although further investigation is warranted to fully understand its role.

Pathway enrichment analysis revealed several signaling pathways that were either commonly activated or inhibited in osteoporosis. Notably, cytokine-cytokine receptor interaction emerged as a commonly activated pathway across both GSE56815 and GSE62402 datasets. This pathway plays a crucial role in immune regulation and inflammation, both of which are increasingly recognized as significant contributors to bone remodeling and osteoporosis progression (10). Previous studies have demonstrated that chronic inflammation can lead to increased osteoclast activity and bone resorption, thereby exacerbating bone loss in osteoporosis patients (11). On the other hand, the efferocytosis pathway was consistently found to be inhibited in both datasets. Efferocytosis, the process of clearing apoptotic cells, is vital for maintaining tissue homeostasis, including bone tissue (12). Inhibition of this pathway may lead to an accumulation of apoptotic cells and a pro-inflammatory environment, further promoting osteoclast activity and bone degradation (13). Recent studies have also suggested that impaired efferocytosis contributes to chronic inflammatory states (14), which can negatively impact bone health and exacerbate osteoporosis. Contrary to initial expectations, our findings revealed that the efferocytosis pathway is suppressed in individuals with high BMD compared to those with osteoporosis (low BMD). This unexpected result suggests a complex relationship between efferocytosis activity and bone remodeling. In osteoporosis, heightened efferocytosis may reflect a compensatory mechanism triggered by increased cell turnover and inflammation, where macrophages attempt to clear excessive apoptotic cells and maintain tissue integrity (15). This heightened activity could be driven by chronic inflammation often observed in low BMD individuals, potentially contributing to a dysregulated inflammatory microenvironment (16). Although this response may temporarily limit tissue damage, it could also perpetuate osteoclast activation, accelerating bone resorption. Doxorubicin, a widely used chemotherapeutic agent, has been implicated in significant skeletal toxicity, including bone loss and impaired bone remodeling. Evidence from recent studies highlights doxorubicin-induced disruptions in oxidative stress pathways and mitochondrial function as key mechanisms underlying its adverse effects on bone tissue (17). Moreover, doxorubicin has been shown to negatively regulate the AKT signaling pathway, an essential mediator of cell survival and differentiation, by reducing AKT phosphorylation and altering downstream targets such as Bcl-2 and Bax (18). This dysregulation promotes apoptosis and diminishes cellular function, potentially exacerbating bone resorption. The findings from our molecular dynamics simulations further demonstrate the strong binding affinity of doxorubicin to AKT1, supporting its potential role in modulating AKT1 activity and contributing to skeletal toxicity.

Our findings also highlight the impact of environmental pollutants, specifically BPA, on bone health through interactions with AKT1. BPA is a widely used synthetic compound in the production of polycarbonate plastics and epoxy resins, found in food packaging, storage containers, and medical devices. Over time, BPA leaches into soil, water, and food, creating persistent environmental exposure with potential health risks (19). Data from the Comparative Toxicogenomics Database (CTD) indicate a bidirectional effect of BPA on AKT1 phosphorylation, which may enhance or inhibit AKT1 activity depending on cellular context. BPA exhibits a context-dependent dual regulatory effect on AKT1 phosphorylation, as highlighted by multiple studies. In 3 T3-L1 adipocytes, BPA downregulates total AKT and phosphorylated AKT levels, leading to suppressed adiponectin production and secretion, and disrupting metabolic homeostasis (20). Similarly, BPA has been shown to inhibit AKT phosphorylation in mouse testicular cells by targeting DPY30-mediated H3K4me3 enrichment, thereby reducing PI3K subunit expression and impairing cell cycle progression (21). Conversely, In lupus-prone MRL/lpr mice, BPA exposure at both low and high doses was associated with increased phosphorylation of key proteins in the PI3K/AKT/mTOR axis, including p-AKT (Ser473, Thr308), p-PI3K, and p-mTOR, contributing to enhanced Th17 cell differentiation and elevated IL-17 levels. This activation of the PI3K/AKT/mTOR pathway suggests a pro-inflammatory role of BPA under specific pathological conditions, such as autoimmune diseases (22). These findings suggest that the effects of BPA on AKT1 phosphorylation are highly dependent on cellular context, exposure conditions, and epigenetic regulation. This dual regulatory potential reflects BPA’s role as an endocrine-disrupting chemical (EDC), known to mimic estrogen and interfere with hormonal functions, thereby posing significant health risks, including impacts on bone health. Molecular docking and dynamics simulations suggest that BPA binds stably to AKT1, potentially modulating its activity in bone remodeling. For example, recent studies indicate that BPA activates the retinoic acid-related orphan receptor α (RORα), a mechanism independent of estrogen receptors, which in turn inhibits osteogenic activity through downregulation of key bone markers such as RUNX2, OSX, and ALP (23). Similarly, other studies have shown that BPA can affect PI3K/AKT signaling pathways, as it activates PI3K/AKT signaling in uterine leiomyoma cells by upregulating XBP1, thereby promoting cell proliferation (24). This suggests that BPA could influence bone health through similar mechanisms, potentially affecting AKT1-mediated bone turnover processes and disrupting the balance between bone formation and resorption.

As an EDC, BPA exerts effects beyond traditional estrogen and androgen receptors, influencing immune cells such as CD4+ T-helper cells and macrophages, both of which play significant roles in bone resorption and formation (25). This multi-pathway impact, including immune modulation, suggests that BPA’s influence on bone health is complex and extends beyond direct hormonal interference (26). Even low-dose exposures, typical of environmental contamination, have been shown to induce significant physiological effects across various biological systems, particularly in endocrine and immune regulation. CLARITY-BPA highlights the complex, non-linear, and sometimes non-monotonic dose–response effects of BPA, challenging traditional toxicological assumptions and suggesting that current regulatory thresholds may underestimate the potential risks of BPA exposure (27). Notably, BPA’s effects on cellular pathways, including bone cells, are dose-dependent, with low-dose exposures potentially contributing to chronic health issues over time. This highlights the public health risk of widespread low-dose BPA exposure, especially for populations at risk of osteoporosis (26). A previous study reported that serum BPA concentration in postmenopausal women with osteoporosis did not show statistically significant associations with clinical variables related to bone metabolism, such as BMD or biochemical bone markers. This finding underscores the complexity of BPA’s influence on bone health, as serum BPA levels alone may not directly predict clinical outcomes due to various confounding factors, including differences in exposure duration, individual metabolic rates, and cellular context (28). Given the complexity of BPA exposure, future studies should incorporate longitudinal BPA exposure assessments, bone biopsies, and tissue-specific gene expression analyses to further investigate the impact of BPA on bone health. Our *in vitro* experiments provide direct mechanistic evidence that BPA enhances RANKL-mediated osteoclastogenesis and promotes AKT1 signaling pathway activation, indicating that BPA disrupts bone homeostasis through the PI3K/AKT signaling pathway. These findings not only further confirm the role of BPA in osteoclast-mediated bone resorption but also emphasize the need for future research to explore its long-term effects on bone metabolism, potential therapeutic interventions, and the underlying molecular mechanisms driving these processes.

This study experimentally validated that BPA significantly enhances RANKL-mediated osteoclastogenesis *in vitro*. BPA treatment increased the formation and size of TRAcP-positive multinucleated osteoclasts and markedly upregulated AKT1 mRNA expression during osteoclast differentiation. Importantly, Western blot analysis confirmed that BPA promotes AKT1 phosphorylation, providing critical protein-level evidence of AKT1 activation in response to BPA exposure. These findings provide direct evidence of BPA’s role in promoting osteoclastogenesis and activating AKT1. By promoting osteoclast activity and upregulating AKT1, BPA may disrupt bone remodeling, favoring bone resorption and contributing to osteoporosis pathogenesis. These results highlight the need for further studies on BPA’s impact on skeletal health and support regulatory efforts to limit environmental exposure to this chemical. These findings align with growing evidence linking EDCs to a wide range of chronic diseases, highlighting the urgent need for stricter environmental regulations to limit EDC exposure. Public health policies aimed at reducing exposure to BPA and other EDCs have the potential to significantly mitigate the burden of osteoporosis, particularly among high-risk populations such as postmenopausal women. Global discrepancies in EDC regulation have already demonstrated substantial differences in health outcomes, emphasizing the necessity of proactive, science-based policy interventions (3). By minimizing BPA exposure, regulatory actions can protect bone remodeling processes, reduce the incidence of bone-related disorders, and alleviate the societal and economic burden associated with osteoporosis.

Although this study provides valuable insights into the role of BPA and AKT1 in osteoporosis, several limitations should be acknowledged. The gene expression analyses were based on publicly available datasets with relatively small sample sizes, which may constrain the generalizability of the findings. Additionally, while the use of different cell types may introduce certain limitations to the study, numerous previous studies have demonstrated that gene expression changes in PBMCs often correlate with those in bone cells, especially concerning osteoporosis-related pathways. BMMs offer greater physiological relevance in studying osteoclast differentiation and function (29, 30). While this approach is widely accepted and biologically relevant for studying bone resorption, the lack of human peripheral blood samples in our experimental validation may limit the direct translation of the findings. The *in vitro* experiments using mouse osteoclasts confirmed both the upregulation of AKT1 mRNA and its phosphorylation upon BPA treatment; however, further mechanistic exploration is needed to elucidate the downstream effects of AKT1 activation on osteoclast function and bone metabolism. In addition, molecular docking and dynamics simulations indicated a stable interaction between BPA and AKT1; however, as an *in silico* approach, this requires additional experimental validation to confirm its biological relevance *in vivo*. To address these limitations and build on the current findings, several future directions are proposed. Further *in vivo* studies using animal models can evaluate the impact of BPA on bone mineral density, bone remodeling, and AKT1-related pathways, including histological and biochemical assessments. Additionally, clinical investigations are needed to explore the association between BPA exposure levels, AKT1 expression, and bone health outcomes in populations vulnerable to osteoporosis, such as postmenopausal women. Expanding the scope of research to include other endocrine-disrupting chemicals will provide a broader perspective on environmental factors influencing bone health. These future studies will validate and extend the current findings, bridging the gap between molecular mechanisms and clinical applications, and advancing the understanding and treatment of osteoporosis.

## 5 Conclusion

This study highlights the role of AKT1 in osteoporosis pathogenesis, demonstrating its upregulation during BPA-induced osteoclastogenesis and its involvement in critical pathways of bone metabolism. By combining computational predictions with experimental validation, we identified AKT1 as a key mediator linking environmental exposure to BPA with disrupted bone remodeling. These findings underscore the complexity of osteoporosis pathophysiology and suggest AKT1 as a potential target for therapeutic intervention. Further research is needed to fully elucidate the dual regulatory effects of BPA on AKT1 activity and its broader implications for bone health.

## Code availability

Parts of the analysis code used in this study have been uploaded to Zenodo and are available at https://doi.org/10.5281/zenodo.14600617. Steps involving software operations that do not generate explicit code (e.g., graphical user interface interactions) can be replicated by following the relevant software user guides and instructions. For additional inquiries or details, readers are encouraged to contact the corresponding author.

## Data Availability

The original contributions presented in the study are included in the article/Supplementary material, further inquiries can be directed to the corresponding authors.

## Footnotes

^1^ https://www.ncbi.nlm.nih.gov/geo/

^2^ http://ctdbase.org

^3^ https://pubchem.ncbi.nlm.nih.gov/

^4^ https://www.rcsb.org/
