# Supplementary figures and images for: Identification of a key environment-responsive gene mediating environmental impact on postmenopausal osteoporosis
Source: Front Public Health. 2025 Mar 27;13:1536851. doi: 10.3389/fpubh.2025.1536851 (PMC11983502; doi:10.3389/fpubh.2025.1536851)

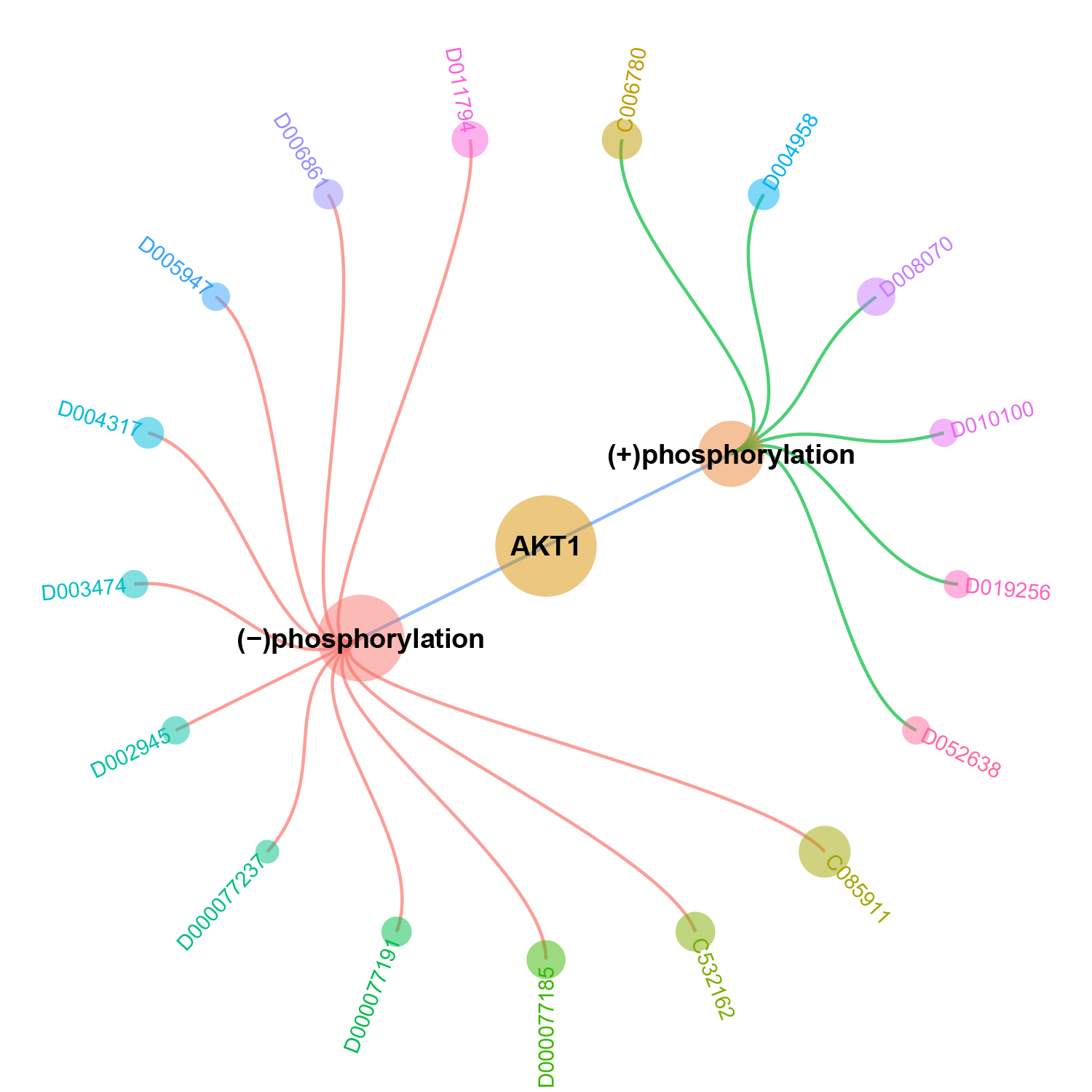

Supplement: Supplementary file 1 [file Image_1.tif]
