# Supplementary material for: Identification of a key environment-responsive gene mediating environmental impact on postmenopausal osteoporosis
Source: Front Public Health. 2025 Mar 27;13:1536851. doi: 10.3389/fpubh.2025.1536851 (PMC11983502; doi:10.3389/fpubh.2025.1536851)
Supplement: Supplementary file 2 [file Table_1.doc]

Supplementary Table S1. Overview of GEO datasets and sample information

| **Accession number** | **Date information** | |
| --- | --- | --- |
| GSE56815 | Sample size | 40 high (20 pre- and 20 postmanopausal) and  40 low hip BMD (20 pre- and 20 postmanopausal) subjects |
|  | Sample source | circulating monocytes |
|  | Organism | Homo sapiens |
| GSE62402 | Sample size | 5 high hip BMD subjects and 5 low hip BMD subjects (Female) |
|  | Sample source | Peripheral blood monocytes |
|  | Organism | Homo sapiens |
| GSE230665 | Sample size | 12 patients with postmenopausal osteoporosis, another 3 healthy postmenopausal women as control group |
|  | Sample source | Femur tissue collected from each patient during hip arthroplasty |
|  | Organism | Homo sapiens |

BMD :Bone mineral density
