# Supplementary material for: Identification of a key environment-responsive gene mediating environmental impact on postmenopausal osteoporosis
Source: Front Public Health. 2025 Mar 27;13:1536851. doi: 10.3389/fpubh.2025.1536851 (PMC11983502; doi:10.3389/fpubh.2025.1536851)
Supplement: Supplementary file 4 [file Table_3.doc]

**Supplementary Table S3. Environmental chemicals affecting AKT1 phosphorylation, including chemical names, IDs, interaction actions, and reference counts.**

| **Chemical Name** | **Chemical ID** | **Gene Symbol** | **Interaction Actions** | **Reference Count** |
| --- | --- | --- | --- | --- |
| 2-(4-morpholinyl)-8-phenyl-4H-1-benzopyran-4-one | C085911 | AKT1 | Decreases phosphorylation | 48 |
| **bisphenol A** | **C006780** | **AKT1** | **Increases phosphorylation** | **24** |
| *bisphenol A* | C006780 | AKT1 | Decreases phosphorylation | 14 |
| 2-(1H-indazol-4-yl)-6-(4-methanesulfonylpiperazin-1-ylmethyl)-4-morpholin-4-ylthieno(3,2-d)pyrimidine | C532162 | AKT1 | Decreases phosphorylation | 23 |
| Resveratrol | D000077185 | AKT1 | Decreases phosphorylation | 22 |
| Quercetin | D011794 | AKT1 | Decreases phosphorylation | 19 |
| Doxorubicin | D004317 | AKT1 | Decreases phosphorylation | 14 |
| Wortmannin | D000077191 | AKT1 | Decreases phosphorylation | 13 |
| *Hydrogen Peroxide* | D006861 | AKT1 | Decreases phosphorylation | 13 |
| **Hydrogen Peroxide** | **D006861** | **AKT1** | **Increases phosphorylation** | **13** |
| Glucose | D005947 | AKT1 | Decreases phosphorylation | 12 |
| Cisplatin | D002945 | AKT1 | Decreases phosphorylation | 12 |
| **Lipopolysaccharides** | D008070 | AKT1 | **Increases phosphorylation** | 21 |
| **Estradiol** | D004958 | AKT1 | **Increases phosphorylation** | 14 |
| **Oxygen** | D010100 | AKT1 | **(deficiency) increases phosphorylation** | 12 |
| **Cadmium Chloride** | D019256 | AKT1 | **Increases phosphorylation** | 12 |
| **Particulate Matter** | D052638 | AKT1 | **Increases phosphorylation** | 12 |
| **Doxorubicin** | D004317 | AKT1 | **Increases phosphorylation** | 11 |
| Arsenic Trioxide | D000077237 | AKT1 | Decreases phosphorylation | 11 |
| Curcumin | D003474 | AKT1 | Decreases phosphorylation | 12 |
