# Supplementary material for: Identification of a key environment-responsive gene mediating environmental impact on postmenopausal osteoporosis
Source: Front Public Health. 2025 Mar 27;13:1536851. doi: 10.3389/fpubh.2025.1536851 (PMC11983502; doi:10.3389/fpubh.2025.1536851)
Supplement: Supplementary file 3 [file Table_2.doc]

Supplementary Table S2. Environmental pollutants associated with postmenopausal osteoporosis identified from the Comparative Toxicogenomics Database.

| Gene Symbol | Disease Name | Inference Score |
| --- | --- | --- |
| IL6 | Osteoporosis, Postmenopausal | 41.81 |
| TGFB1 | Osteoporosis, Postmenopausal | 40.75 |
| TNF | Osteoporosis, Postmenopausal | 34.05 |
| IL1B | Osteoporosis, Postmenopausal | 31.58 |
| CAT | Osteoporosis, Postmenopausal | 24.67 |
| FDPS | Osteoporosis, Postmenopausal | 17.31 |
| PTH | Osteoporosis, Postmenopausal | 36.47 |
| VDR | Osteoporosis, Postmenopausal | 45.32 |
| SERPINE1 | Osteoporosis, Postmenopausal | 44.9 |
| ESR1 | Osteoporosis, Postmenopausal | 41.55 |
| TNFRSF11B | Osteoporosis, Postmenopausal | 40.72 |
| ESR2 | Osteoporosis, Postmenopausal | 40.54 |
| TNFSF11 | Osteoporosis, Postmenopausal | 39.8 |
| MAPK14 | Osteoporosis, Postmenopausal | 39.08 |
| MGP | Osteoporosis, Postmenopausal | 38.17 |
| WNT5A | Osteoporosis, Postmenopausal | 38.12 |
| CYP24A1 | Osteoporosis, Postmenopausal | 37.8 |
| SPP1 | Osteoporosis, Postmenopausal | 37.8 |
| CDH2 | Osteoporosis, Postmenopausal | 37.71 |
| RUNX2 | Osteoporosis, Postmenopausal | 37.65 |
| CTSK | Osteoporosis, Postmenopausal | 37.44 |
| COL1A2 | Osteoporosis, Postmenopausal | 37.42 |
| MAPK3 | Osteoporosis, Postmenopausal | 37.14 |
| MAPK1 | Osteoporosis, Postmenopausal | 36.93 |
| BGLAP | Osteoporosis, Postmenopausal | 36.29 |
| CYP27B1 | Osteoporosis, Postmenopausal | 36.09 |
| TGFA | Osteoporosis, Postmenopausal | 35.58 |
| CTNNB1 | Osteoporosis, Postmenopausal | 35.32 |
| AR | Osteoporosis, Postmenopausal | 34.9 |
| IL4 | Osteoporosis, Postmenopausal | 34.87 |
| TIMP2 | Osteoporosis, Postmenopausal | 33.76 |
| TLR2 | Osteoporosis, Postmenopausal | 33.67 |
| CYP3A4 | Osteoporosis, Postmenopausal | 33.66 |
| MMP2 | Osteoporosis, Postmenopausal | 33.01 |
| CRP | Osteoporosis, Postmenopausal | 32.8 |
| PLAT | Osteoporosis, Postmenopausal | 32.75 |
| NCOA1 | Osteoporosis, Postmenopausal | 32.65 |
| TP53 | Osteoporosis, Postmenopausal | 32.57 |
| RELA | Osteoporosis, Postmenopausal | 32.4 |
| ATP1A1 | Osteoporosis, Postmenopausal | 31.95 |
| TRPV6 | Osteoporosis, Postmenopausal | 31.71 |
| RBBP8 | Osteoporosis, Postmenopausal | 31.64 |
| BAD | Osteoporosis, Postmenopausal | 31.63 |
| NOS3 | Osteoporosis, Postmenopausal | 31.55 |
| LDLR | Osteoporosis, Postmenopausal | 31.54 |
| CASP3 | Osteoporosis, Postmenopausal | 31.45 |
| SPARC | Osteoporosis, Postmenopausal | 31.38 |
| TGFBR2 | Osteoporosis, Postmenopausal | 30.81 |
| HES1 | Osteoporosis, Postmenopausal | 30.62 |
| CEBPB | Osteoporosis, Postmenopausal | 30.6 |
| SOD1 | Osteoporosis, Postmenopausal | 30.47 |
| TERT | Osteoporosis, Postmenopausal | 30.47 |
| LHB | Osteoporosis, Postmenopausal | 30.46 |
| TRPV5 | Osteoporosis, Postmenopausal | 30.41 |
| ELF3 | Osteoporosis, Postmenopausal | 30.38 |
| EGFR | Osteoporosis, Postmenopausal | 30.07 |
| FKBP5 | Osteoporosis, Postmenopausal | 30.05 |
| VEGFA | Osteoporosis, Postmenopausal | 29.95 |
| AKT1 | Osteoporosis, Postmenopausal | 29.71 |
| PGR | Osteoporosis, Postmenopausal | 29.7 |
| IER3 | Osteoporosis, Postmenopausal | 29.68 |
| AREG | Osteoporosis, Postmenopausal | 29.57 |
| CYP19A1 | Osteoporosis, Postmenopausal | 29.54 |
| NCOA3 | Osteoporosis, Postmenopausal | 29.51 |
| PPARG | Osteoporosis, Postmenopausal | 29.46 |
| ITGAM | Osteoporosis, Postmenopausal | 29.3 |
| CCND1 | Osteoporosis, Postmenopausal | 29.29 |
| ARG1 | Osteoporosis, Postmenopausal | 29.25 |
| RB1 | Osteoporosis, Postmenopausal | 29.12 |
| GREB1 | Osteoporosis, Postmenopausal | 29.1 |
| TGFB2 | Osteoporosis, Postmenopausal | 29.09 |
| CD14 | Osteoporosis, Postmenopausal | 29.04 |
| BCL2L1 | Osteoporosis, Postmenopausal | 28.96 |
| IGFBP3 | Osteoporosis, Postmenopausal | 28.77 |
| SLC2A4 | Osteoporosis, Postmenopausal | 28.62 |
| MUC1 | Osteoporosis, Postmenopausal | 28.52 |
| PTGS2 | Osteoporosis, Postmenopausal | 28.49 |
| EDN1 | Osteoporosis, Postmenopausal | 28.44 |
| GPER1 | Osteoporosis, Postmenopausal | 28.44 |
| CD80 | Osteoporosis, Postmenopausal | 28.4 |
| VCAM1 | Osteoporosis, Postmenopausal | 28.24 |
| HMGB1 | Osteoporosis, Postmenopausal | 28.17 |
| IL6ST | Osteoporosis, Postmenopausal | 28.17 |
| CDKN2B | Osteoporosis, Postmenopausal | 28.09 |
| VIM | Osteoporosis, Postmenopausal | 28.07 |
| SERPINB2 | Osteoporosis, Postmenopausal | 28.06 |
| EGR1 | Osteoporosis, Postmenopausal | 28 |
| TLR4 | Osteoporosis, Postmenopausal | 27.97 |
| SMAD7 | Osteoporosis, Postmenopausal | 27.91 |
| ABCC2 | Osteoporosis, Postmenopausal | 27.77 |
| NCOA2 | Osteoporosis, Postmenopausal | 27.66 |
| KIF20A | Osteoporosis, Postmenopausal | 27.55 |
| FUT3 | Osteoporosis, Postmenopausal | 27.52 |
| CDC6 | Osteoporosis, Postmenopausal | 27.38 |
| APOV1 | Osteoporosis, Postmenopausal | 27.35 |
| JUN | Osteoporosis, Postmenopausal | 27.32 |
| TGFB3 | Osteoporosis, Postmenopausal | 27.21 |
| ACP3 | Osteoporosis, Postmenopausal | 27.1 |
| G0S2 | Osteoporosis, Postmenopausal | 27.05 |
| DUSP1 | Osteoporosis, Postmenopausal | 27.03 |
| F2R | Osteoporosis, Postmenopausal | 26.96 |
| CCNG2 | Osteoporosis, Postmenopausal | 26.86 |
| IL17RB | Osteoporosis, Postmenopausal | 26.85 |
| WWC1 | Osteoporosis, Postmenopausal | 26.85 |
| S100G | Osteoporosis, Postmenopausal | 26.69 |
| AGR2 | Osteoporosis, Postmenopausal | 26.68 |
| ACTA2 | Osteoporosis, Postmenopausal | 26.67 |
| HSD17B12 | Osteoporosis, Postmenopausal | 26.63 |
| PCNA | Osteoporosis, Postmenopausal | 26.61 |
| BMP4 | Osteoporosis, Postmenopausal | 26.53 |
| BCL2 | Osteoporosis, Postmenopausal | 26.35 |
| IL4R | Osteoporosis, Postmenopausal | 26.3 |
| FOS | Osteoporosis, Postmenopausal | 26.27 |
| CD68 | Osteoporosis, Postmenopausal | 26.26 |
| MAP2K2 | Osteoporosis, Postmenopausal | 26.26 |
| CXCL10 | Osteoporosis, Postmenopausal | 26.2 |
| TNFRSF11A | Osteoporosis, Postmenopausal | 26.19 |
| IGF2 | Osteoporosis, Postmenopausal | 26.12 |
| KLK2 | Osteoporosis, Postmenopausal | 26.09 |
| TFF1 | Osteoporosis, Postmenopausal | 26.09 |
| ANXA2 | Osteoporosis, Postmenopausal | 25.98 |
| LIF | Osteoporosis, Postmenopausal | 25.98 |
| STMN1 | Osteoporosis, Postmenopausal | 25.98 |
| NFKB1 | Osteoporosis, Postmenopausal | 25.95 |
| PDGFB | Osteoporosis, Postmenopausal | 25.9 |
| TSC22D3 | Osteoporosis, Postmenopausal | 25.82 |
| TP73 | Osteoporosis, Postmenopausal | 25.68 |
| OXTR | Osteoporosis, Postmenopausal | 25.58 |
| CXCL2 | Osteoporosis, Postmenopausal | 25.57 |
| CYP1B1 | Osteoporosis, Postmenopausal | 25.56 |
| GPX2 | Osteoporosis, Postmenopausal | 25.55 |
| SP7 | Osteoporosis, Postmenopausal | 25.5 |
| RBL1 | Osteoporosis, Postmenopausal | 25.49 |
| F13A1 | Osteoporosis, Postmenopausal | 25.45 |
| RAP1A | Osteoporosis, Postmenopausal | 25.37 |
| HBP1 | Osteoporosis, Postmenopausal | 25.31 |
| CDK2 | Osteoporosis, Postmenopausal | 25.28 |
| KCNN4 | Osteoporosis, Postmenopausal | 25.27 |
| LBH | Osteoporosis, Postmenopausal | 25.26 |
| TOP2B | Osteoporosis, Postmenopausal | 25.26 |
| CYP2E1 | Osteoporosis, Postmenopausal | 25.25 |
| KL | Osteoporosis, Postmenopausal | 25.24 |
| MCPT1 | Osteoporosis, Postmenopausal | 25.23 |
| ASPH | Osteoporosis, Postmenopausal | 25.21 |
| EFNB2 | Osteoporosis, Postmenopausal | 25.21 |
| BMERB1 | Osteoporosis, Postmenopausal | 25.18 |
| ICAM1 | Osteoporosis, Postmenopausal | 25.18 |
| HGF | Osteoporosis, Postmenopausal | 25.17 |
| NR1I2 | Osteoporosis, Postmenopausal | 25.12 |
| LEP | Osteoporosis, Postmenopausal | 25.08 |
| GLUL | Osteoporosis, Postmenopausal | 25.03 |
| TMT1A | Osteoporosis, Postmenopausal | 25.03 |
| HSD3B1 | Osteoporosis, Postmenopausal | 24.98 |
| NOS2A | Osteoporosis, Postmenopausal | 24.89 |
| CDKN1B | Osteoporosis, Postmenopausal | 24.88 |
| ESR2A | Osteoporosis, Postmenopausal | 24.84 |
| NTS | Osteoporosis, Postmenopausal | 24.83 |
| ALOX5 | Osteoporosis, Postmenopausal | 24.82 |
| KIF15 | Osteoporosis, Postmenopausal | 24.78 |
| TP63 | Osteoporosis, Postmenopausal | 24.74 |
| CDKN1A | Osteoporosis, Postmenopausal | 24.71 |
| FASTKD1 | Osteoporosis, Postmenopausal | 24.7 |
| PIK3R3 | Osteoporosis, Postmenopausal | 24.69 |
| ADGRG1 | Osteoporosis, Postmenopausal | 24.66 |
| ATP8B1 | Osteoporosis, Postmenopausal | 24.66 |
| CYP2C29 | Osteoporosis, Postmenopausal | 24.66 |
| RXRA | Osteoporosis, Postmenopausal | 24.64 |
| IL1RN | Osteoporosis, Postmenopausal | 24.59 |
| EIF5A | Osteoporosis, Postmenopausal | 24.58 |
| MKI67 | Osteoporosis, Postmenopausal | 24.56 |
| E2F2 | Osteoporosis, Postmenopausal | 24.53 |
| SREBF1 | Osteoporosis, Postmenopausal | 24.5 |
| TNFRSF21 | Osteoporosis, Postmenopausal | 24.49 |
| CASP7 | Osteoporosis, Postmenopausal | 24.42 |
| NCOR2 | Osteoporosis, Postmenopausal | 24.39 |
| PPM1M | Osteoporosis, Postmenopausal | 24.38 |
| XRCC5 | Osteoporosis, Postmenopausal | 24.38 |
| ELAPOR1 | Osteoporosis, Postmenopausal | 24.36 |
| MCM10 | Osteoporosis, Postmenopausal | 24.34 |
| CXCL8 | Osteoporosis, Postmenopausal | 24.32 |
| PRKCA | Osteoporosis, Postmenopausal | 24.32 |
| ROCK1 | Osteoporosis, Postmenopausal | 24.31 |
| CENPA | Osteoporosis, Postmenopausal | 24.3 |
| DNMT1 | Osteoporosis, Postmenopausal | 24.3 |
| OLR1 | Osteoporosis, Postmenopausal | 24.27 |
| CAV1 | Osteoporosis, Postmenopausal | 24.26 |
| CCNA1 | Osteoporosis, Postmenopausal | 24.26 |
| ATP2B1 | Osteoporosis, Postmenopausal | 24.23 |
| ITPR3 | Osteoporosis, Postmenopausal | 24.23 |
| EGR3 | Osteoporosis, Postmenopausal | 24.22 |
| PMAIP1 | Osteoporosis, Postmenopausal | 24.19 |
| SLC19A1 | Osteoporosis, Postmenopausal | 24.12 |
| CD86 | Osteoporosis, Postmenopausal | 24.11 |
| PLA2G2A | Osteoporosis, Postmenopausal | 24.11 |
| DNAJC3 | Osteoporosis, Postmenopausal | 24.07 |
| ECT2 | Osteoporosis, Postmenopausal | 24.07 |
| BMP7 | Osteoporosis, Postmenopausal | 24.04 |
| LYN | Osteoporosis, Postmenopausal | 24.04 |
| TXNIP | Osteoporosis, Postmenopausal | 24.03 |
| JAK2 | Osteoporosis, Postmenopausal | 24.01 |
| MT1X | Osteoporosis, Postmenopausal | 23.9 |
| HSD17B2 | Osteoporosis, Postmenopausal | 23.89 |
| TMPO | Osteoporosis, Postmenopausal | 23.85 |
| OCLN | Osteoporosis, Postmenopausal | 23.84 |
| IGF1 | Osteoporosis, Postmenopausal | 23.83 |
| SMAD3 | Osteoporosis, Postmenopausal | 23.83 |
| SOX4 | Osteoporosis, Postmenopausal | 23.79 |
| ITGA2 | Osteoporosis, Postmenopausal | 23.78 |
| TFPI | Osteoporosis, Postmenopausal | 23.75 |
| SULF2 | Osteoporosis, Postmenopausal | 23.72 |
| AHNAK | Osteoporosis, Postmenopausal | 23.71 |
| NAIP | Osteoporosis, Postmenopausal | 23.71 |
| AXIN2 | Osteoporosis, Postmenopausal | 23.66 |
| IL10 | Osteoporosis, Postmenopausal | 23.65 |
| CEBPA | Osteoporosis, Postmenopausal | 23.62 |
| PRL | Osteoporosis, Postmenopausal | 23.61 |
| SERPINH1 | Osteoporosis, Postmenopausal | 23.6 |
| SLC18A2 | Osteoporosis, Postmenopausal | 23.58 |
| CCNB1 | Osteoporosis, Postmenopausal | 23.56 |
| CASP9 | Osteoporosis, Postmenopausal | 23.52 |
| SLC30A1 | Osteoporosis, Postmenopausal | 23.52 |
| TJP1 | Osteoporosis, Postmenopausal | 23.51 |
| MTOR | Osteoporosis, Postmenopausal | 23.5 |
| NOS2 | Osteoporosis, Postmenopausal | 23.49 |
| H3C4 | Osteoporosis, Postmenopausal | 23.48 |
| KLK3 | Osteoporosis, Postmenopausal | 23.45 |
| CYP1A1 | Osteoporosis, Postmenopausal | 23.44 |
| NFKBIZ | Osteoporosis, Postmenopausal | 23.42 |
| SKP2 | Osteoporosis, Postmenopausal | 23.38 |
| CA4 | Osteoporosis, Postmenopausal | 23.37 |
| GJB2 | Osteoporosis, Postmenopausal | 23.35 |
| ARFGEF3 | Osteoporosis, Postmenopausal | 23.32 |
| RELB | Osteoporosis, Postmenopausal | 23.31 |
| UNG | Osteoporosis, Postmenopausal | 23.31 |
| IGFBP4 | Osteoporosis, Postmenopausal | 23.28 |
| ACP5 | Osteoporosis, Postmenopausal | 23.26 |
| MMP1 | Osteoporosis, Postmenopausal | 23.26 |
| CDKN2C | Osteoporosis, Postmenopausal | 23.25 |
| AGT | Osteoporosis, Postmenopausal | 23.22 |
| CDC42 | Osteoporosis, Postmenopausal | 23.21 |
| MMP12 | Osteoporosis, Postmenopausal | 23.19 |
| EFNA1 | Osteoporosis, Postmenopausal | 23.18 |
| ALPL | Osteoporosis, Postmenopausal | 23.17 |
| NFKBIA | Osteoporosis, Postmenopausal | 23.17 |
| FSHB | Osteoporosis, Postmenopausal | 23.16 |
| CDH1 | Osteoporosis, Postmenopausal | 23.14 |
| GH1 | Osteoporosis, Postmenopausal | 23.13 |
| TRAV8-6 | Osteoporosis, Postmenopausal | 23.12 |
| ESR2B | Osteoporosis, Postmenopausal | 23.08 |
| KLF5 | Osteoporosis, Postmenopausal | 23.06 |
| MMP9 | Osteoporosis, Postmenopausal | 23.06 |
| ATP1B1 | Osteoporosis, Postmenopausal | 23 |
| SERPINE2 | Osteoporosis, Postmenopausal | 23 |
| WNK2 | Osteoporosis, Postmenopausal | 23 |
| ZFX | Osteoporosis, Postmenopausal | 23 |
| GHR | Osteoporosis, Postmenopausal | 22.97 |
| AGER | Osteoporosis, Postmenopausal | 22.96 |
| FABP4 | Osteoporosis, Postmenopausal | 22.96 |
| MYL9 | Osteoporosis, Postmenopausal | 22.95 |
| SRC | Osteoporosis, Postmenopausal | 22.94 |
| XDH | Osteoporosis, Postmenopausal | 22.94 |
| BTG1 | Osteoporosis, Postmenopausal | 22.9 |
| ERBB3 | Osteoporosis, Postmenopausal | 22.9 |
| SELP | Osteoporosis, Postmenopausal | 22.88 |
| RPS6KB1 | Osteoporosis, Postmenopausal | 22.82 |
| CSF2 | Osteoporosis, Postmenopausal | 22.81 |
| SOD2 | Osteoporosis, Postmenopausal | 22.75 |
| FST | Osteoporosis, Postmenopausal | 22.73 |
| PTPN1 | Osteoporosis, Postmenopausal | 22.73 |
| MED13 | Osteoporosis, Postmenopausal | 22.72 |
| HP | Osteoporosis, Postmenopausal | 22.7 |
| COX1 | Osteoporosis, Postmenopausal | 22.67 |
| GLS | Osteoporosis, Postmenopausal | 22.66 |
| TMEM117 | Osteoporosis, Postmenopausal | 22.66 |
| TNFAIP6 | Osteoporosis, Postmenopausal | 22.65 |
| HNF4A | Osteoporosis, Postmenopausal | 22.62 |
| TH | Osteoporosis, Postmenopausal | 22.61 |
| CYP11A1 | Osteoporosis, Postmenopausal | 22.6 |
| IFNG | Osteoporosis, Postmenopausal | 22.56 |
| PTGES | Osteoporosis, Postmenopausal | 22.53 |
| SOD3 | Osteoporosis, Postmenopausal | 22.52 |
| SLC22A2 | Osteoporosis, Postmenopausal | 22.5 |
| PTGS1 | Osteoporosis, Postmenopausal | 22.49 |
| CHAC2 | Osteoporosis, Postmenopausal | 22.48 |
| SQSTM1 | Osteoporosis, Postmenopausal | 22.47 |
| CBX6 | Osteoporosis, Postmenopausal | 22.44 |
| NFKB2 | Osteoporosis, Postmenopausal | 22.44 |
| ALDH1A1 | Osteoporosis, Postmenopausal | 22.43 |
| CCN2 | Osteoporosis, Postmenopausal | 22.43 |
| PHTF2 | Osteoporosis, Postmenopausal | 22.42 |
| CDH6 | Osteoporosis, Postmenopausal | 22.4 |
| HAMP | Osteoporosis, Postmenopausal | 22.4 |
| LMNB1 | Osteoporosis, Postmenopausal | 22.38 |
| H2BC5 | Osteoporosis, Postmenopausal | 22.36 |
| PRNP | Osteoporosis, Postmenopausal | 22.34 |
| SP4 | Osteoporosis, Postmenopausal | 22.32 |
| ANAPC1 | Osteoporosis, Postmenopausal | 22.31 |
| CCL3 | Osteoporosis, Postmenopausal | 22.3 |
| MYLK | Osteoporosis, Postmenopausal | 22.27 |
| HOMER2 | Osteoporosis, Postmenopausal | 22.26 |
| SYBU | Osteoporosis, Postmenopausal | 22.26 |
| HMOX1 | Osteoporosis, Postmenopausal | 22.24 |
| APOBEC3B | Osteoporosis, Postmenopausal | 22.19 |
| ADGRE5 | Osteoporosis, Postmenopausal | 22.14 |
| KRT19 | Osteoporosis, Postmenopausal | 22.11 |
| UGT1A1 | Osteoporosis, Postmenopausal | 22.11 |
| TPST1 | Osteoporosis, Postmenopausal | 22.1 |
| MAP3K5 | Osteoporosis, Postmenopausal | 22.09 |
| KLHL5 | Osteoporosis, Postmenopausal | 22.08 |
| DHCR24 | Osteoporosis, Postmenopausal | 22.04 |
| SGK3 | Osteoporosis, Postmenopausal | 22.03 |
| TK1 | Osteoporosis, Postmenopausal | 22.01 |
| CYP2C9 | Osteoporosis, Postmenopausal | 21.99 |
| SEMA3F | Osteoporosis, Postmenopausal | 21.98 |
| DERL3 | Osteoporosis, Postmenopausal | 21.97 |
| PIGR | Osteoporosis, Postmenopausal | 21.95 |
| MPO | Osteoporosis, Postmenopausal | 21.93 |
| NPPC | Osteoporosis, Postmenopausal | 21.93 |
| MYOD1 | Osteoporosis, Postmenopausal | 21.87 |
| TICAM1 | Osteoporosis, Postmenopausal | 21.84 |
| CASP4 | Osteoporosis, Postmenopausal | 21.83 |
| EGF | Osteoporosis, Postmenopausal | 21.83 |
| CSF1 | Osteoporosis, Postmenopausal | 21.82 |
| PTPRN2 | Osteoporosis, Postmenopausal | 21.81 |
| CREBBP | Osteoporosis, Postmenopausal | 21.8 |
| ITPR1 | Osteoporosis, Postmenopausal | 21.78 |
| PLK1 | Osteoporosis, Postmenopausal | 21.78 |
| CCNA2 | Osteoporosis, Postmenopausal | 21.76 |
| BECN1 | Osteoporosis, Postmenopausal | 21.75 |
| PRDX2 | Osteoporosis, Postmenopausal | 21.75 |
| GALM | Osteoporosis, Postmenopausal | 21.73 |
| MLPH | Osteoporosis, Postmenopausal | 21.73 |
| SLC2A2 | Osteoporosis, Postmenopausal | 21.73 |
| EPHA1 | Osteoporosis, Postmenopausal | 21.72 |
| SLC2A9 | Osteoporosis, Postmenopausal | 21.72 |
| PTPN12 | Osteoporosis, Postmenopausal | 21.7 |
| REN | Osteoporosis, Postmenopausal | 21.7 |
| DUSP5 | Osteoporosis, Postmenopausal | 21.68 |
| ATP2A2 | Osteoporosis, Postmenopausal | 21.66 |
| AURKA | Osteoporosis, Postmenopausal | 21.65 |
| RIPOR2 | Osteoporosis, Postmenopausal | 21.64 |
| INPP4B | Osteoporosis, Postmenopausal | 21.62 |
| SKAP2 | Osteoporosis, Postmenopausal | 21.58 |
| TXNRD1 | Osteoporosis, Postmenopausal | 21.58 |
| DHRS2 | Osteoporosis, Postmenopausal | 21.52 |
| STAT3 | Osteoporosis, Postmenopausal | 21.52 |
| CCL2 | Osteoporosis, Postmenopausal | 21.51 |
| CCL5 | Osteoporosis, Postmenopausal | 21.47 |
| ASRGL1 | Osteoporosis, Postmenopausal | 21.46 |
| CIT | Osteoporosis, Postmenopausal | 21.42 |
| SNAI2 | Osteoporosis, Postmenopausal | 21.42 |
| SFTPB | Osteoporosis, Postmenopausal | 21.41 |
| SESN2 | Osteoporosis, Postmenopausal | 21.4 |
| CLGN | Osteoporosis, Postmenopausal | 21.38 |
| BMP2 | Osteoporosis, Postmenopausal | 21.37 |
| CTHRC1 | Osteoporosis, Postmenopausal | 21.37 |
| PDGFRL | Osteoporosis, Postmenopausal | 21.37 |
| CYBA | Osteoporosis, Postmenopausal | 21.32 |
| HPRT1 | Osteoporosis, Postmenopausal | 21.3 |
| LRIG1 | Osteoporosis, Postmenopausal | 21.29 |
| PDGFRB | Osteoporosis, Postmenopausal | 21.29 |
| PSEN2 | Osteoporosis, Postmenopausal | 21.29 |
| IL1R1 | Osteoporosis, Postmenopausal | 21.27 |
| PTPRN | Osteoporosis, Postmenopausal | 21.27 |
| SMAD2 | Osteoporosis, Postmenopausal | 21.26 |
| CHN2 | Osteoporosis, Postmenopausal | 21.25 |
| PRKD1 | Osteoporosis, Postmenopausal | 21.24 |
| CCL8 | Osteoporosis, Postmenopausal | 21.23 |
| KIF14 | Osteoporosis, Postmenopausal | 21.23 |
| CEBPD | Osteoporosis, Postmenopausal | 21.21 |
| CD40 | Osteoporosis, Postmenopausal | 21.19 |
| RAPH1 | Osteoporosis, Postmenopausal | 21.15 |
| GCNT3 | Osteoporosis, Postmenopausal | 21.14 |
| LGR5 | Osteoporosis, Postmenopausal | 21.14 |
| CDC20 | Osteoporosis, Postmenopausal | 21.13 |
| CDC25C | Osteoporosis, Postmenopausal | 21.11 |
| HHIP | Osteoporosis, Postmenopausal | 21.11 |
| STBD1 | Osteoporosis, Postmenopausal | 21.1 |
| S100A8 | Osteoporosis, Postmenopausal | 21.08 |
| ADM | Osteoporosis, Postmenopausal | 21.06 |
| BAG2 | Osteoporosis, Postmenopausal | 21.06 |
| ATF3 | Osteoporosis, Postmenopausal | 21.04 |
| HOXA1 | Osteoporosis, Postmenopausal | 21.04 |
| MCPT4 | Osteoporosis, Postmenopausal | 21.02 |
| ACHE | Osteoporosis, Postmenopausal | 21.01 |
| ITGB1 | Osteoporosis, Postmenopausal | 21.01 |
| S100A9 | Osteoporosis, Postmenopausal | 21.01 |
| CCNB2 | Osteoporosis, Postmenopausal | 21 |
| CHAF1A | Osteoporosis, Postmenopausal | 21 |
| RAD51C | Osteoporosis, Postmenopausal | 21 |
| SIAH2 | Osteoporosis, Postmenopausal | 20.97 |
| GGH | Osteoporosis, Postmenopausal | 20.92 |
| ATP6V1A | Osteoporosis, Postmenopausal | 20.9 |
| COL2A1 | Osteoporosis, Postmenopausal | 20.9 |
| HSP90B1 | Osteoporosis, Postmenopausal | 20.86 |
| ANXA1 | Osteoporosis, Postmenopausal | 20.84 |
| CEMIP | Osteoporosis, Postmenopausal | 20.83 |
| GADD45A | Osteoporosis, Postmenopausal | 20.83 |
| MAP3K14 | Osteoporosis, Postmenopausal | 20.83 |
| PKMYT1 | Osteoporosis, Postmenopausal | 20.83 |
| BAX | Osteoporosis, Postmenopausal | 20.82 |
| NR3C1 | Osteoporosis, Postmenopausal | 20.82 |
| ANO1 | Osteoporosis, Postmenopausal | 20.8 |
| LXN | Osteoporosis, Postmenopausal | 20.8 |
| PTGIS | Osteoporosis, Postmenopausal | 20.8 |
| TIMP1 | Osteoporosis, Postmenopausal | 20.8 |
| NFE2L2 | Osteoporosis, Postmenopausal | 20.79 |
| PTH1R | Osteoporosis, Postmenopausal | 20.79 |
| TP53I3 | Osteoporosis, Postmenopausal | 20.79 |
| CLMN | Osteoporosis, Postmenopausal | 20.76 |
| DUSP6 | Osteoporosis, Postmenopausal | 20.76 |
| ITGAX | Osteoporosis, Postmenopausal | 20.75 |
| PARP1 | Osteoporosis, Postmenopausal | 20.75 |
| GRK5 | Osteoporosis, Postmenopausal | 20.74 |
| KRAS | Osteoporosis, Postmenopausal | 20.73 |
| IHH | Osteoporosis, Postmenopausal | 20.71 |
| CLDN1 | Osteoporosis, Postmenopausal | 20.69 |
| KRT23 | Osteoporosis, Postmenopausal | 20.69 |
| AQP7 | Osteoporosis, Postmenopausal | 20.66 |
| MAPK13 | Osteoporosis, Postmenopausal | 20.66 |
| UBB | Osteoporosis, Postmenopausal | 20.65 |
| FN1 | Osteoporosis, Postmenopausal | 20.63 |
| NCOA4 | Osteoporosis, Postmenopausal | 20.63 |
| SP1 | Osteoporosis, Postmenopausal | 20.63 |
| MAFF | Osteoporosis, Postmenopausal | 20.62 |
| MAPK11 | Osteoporosis, Postmenopausal | 20.62 |
| OLFML3 | Osteoporosis, Postmenopausal | 20.62 |
| PLAUR | Osteoporosis, Postmenopausal | 20.62 |
| SLC3A2 | Osteoporosis, Postmenopausal | 20.58 |
| SMC2 | Osteoporosis, Postmenopausal | 20.58 |
| F2 | Osteoporosis, Postmenopausal | 20.57 |
| CLDN11 | Osteoporosis, Postmenopausal | 20.54 |
| OARD1 | Osteoporosis, Postmenopausal | 20.54 |
| PKP2 | Osteoporosis, Postmenopausal | 20.54 |
| CDA | Osteoporosis, Postmenopausal | 20.53 |
| KISS1 | Osteoporosis, Postmenopausal | 20.53 |
| CREB1 | Osteoporosis, Postmenopausal | 20.52 |
| ORC6 | Osteoporosis, Postmenopausal | 20.52 |
| CENPU | Osteoporosis, Postmenopausal | 20.5 |
| KRT15 | Osteoporosis, Postmenopausal | 20.5 |
| RRM2 | Osteoporosis, Postmenopausal | 20.48 |
| CDK1 | Osteoporosis, Postmenopausal | 20.47 |
| DTL | Osteoporosis, Postmenopausal | 20.46 |
| HSPG2 | Osteoporosis, Postmenopausal | 20.46 |
| MIR148A | Osteoporosis, Postmenopausal | 20.46 |
| NFATC2 | Osteoporosis, Postmenopausal | 20.46 |
| MMP13 | Osteoporosis, Postmenopausal | 20.45 |
| CCND2 | Osteoporosis, Postmenopausal | 20.44 |
| FOXO3 | Osteoporosis, Postmenopausal | 20.44 |
| RASGRP1 | Osteoporosis, Postmenopausal | 20.43 |
| ETV4 | Osteoporosis, Postmenopausal | 20.41 |
| FANCI | Osteoporosis, Postmenopausal | 20.39 |
| PPP1R15A | Osteoporosis, Postmenopausal | 20.39 |
| TP53INP1 | Osteoporosis, Postmenopausal | 20.39 |
| PALLD | Osteoporosis, Postmenopausal | 20.37 |
| SERPINB9 | Osteoporosis, Postmenopausal | 20.36 |
| NRAS | Osteoporosis, Postmenopausal | 20.32 |
| PSRC1 | Osteoporosis, Postmenopausal | 20.32 |
| ACTB | Osteoporosis, Postmenopausal | 20.31 |
| PXN | Osteoporosis, Postmenopausal | 20.29 |
| LMCD1 | Osteoporosis, Postmenopausal | 20.28 |
| PTGER3 | Osteoporosis, Postmenopausal | 20.28 |
| XPO1 | Osteoporosis, Postmenopausal | 20.28 |
| INS1 | Osteoporosis, Postmenopausal | 20.27 |
| BIRC5 | Osteoporosis, Postmenopausal | 20.26 |
| CDK5R1 | Osteoporosis, Postmenopausal | 20.26 |
| MMP3 | Osteoporosis, Postmenopausal | 20.26 |
| IFIH1 | Osteoporosis, Postmenopausal | 20.25 |
| DDB2 | Osteoporosis, Postmenopausal | 20.24 |
| FAM83E | Osteoporosis, Postmenopausal | 20.23 |
| GRB2 | Osteoporosis, Postmenopausal | 20.23 |
| RFC3 | Osteoporosis, Postmenopausal | 20.23 |
| E2F7 | Osteoporosis, Postmenopausal | 20.2 |
| CCN1 | Osteoporosis, Postmenopausal | 20.19 |
| HBG2 | Osteoporosis, Postmenopausal | 20.18 |
| PAK1 | Osteoporosis, Postmenopausal | 20.17 |
| TDO2 | Osteoporosis, Postmenopausal | 20.17 |
| CDCA5 | Osteoporosis, Postmenopausal | 20.16 |
| BTG2 | Osteoporosis, Postmenopausal | 20.15 |
| FOXO1 | Osteoporosis, Postmenopausal | 20.15 |
| TPD52L1 | Osteoporosis, Postmenopausal | 20.13 |
| IRS1 | Osteoporosis, Postmenopausal | 20.12 |
| EPHA4 | Osteoporosis, Postmenopausal | 20.11 |
| CXCR4 | Osteoporosis, Postmenopausal | 20.1 |
| MAOA | Osteoporosis, Postmenopausal | 20.1 |
| PLK4 | Osteoporosis, Postmenopausal | 20.1 |
| RPS6KA3 | Osteoporosis, Postmenopausal | 20.09 |
| ANXA6 | Osteoporosis, Postmenopausal | 20.06 |
| KIF20B | Osteoporosis, Postmenopausal | 20.06 |
| FOSL1 | Osteoporosis, Postmenopausal | 20.04 |
| EIF1 | Osteoporosis, Postmenopausal | 20.03 |
| IRF3 | Osteoporosis, Postmenopausal | 20.03 |
| NCAPG | Osteoporosis, Postmenopausal | 20.01 |
| AGO2 | Osteoporosis, Postmenopausal | 19.97 |
| SLC7A7 | Osteoporosis, Postmenopausal | 19.96 |
| HRAS | Osteoporosis, Postmenopausal | 19.95 |
| IDO1 | Osteoporosis, Postmenopausal | 19.95 |
| FOXA1 | Osteoporosis, Postmenopausal | 19.93 |
| MUG2 | Osteoporosis, Postmenopausal | 19.92 |
| DDIT4 | Osteoporosis, Postmenopausal | 19.91 |
| IKBKB | Osteoporosis, Postmenopausal | 19.91 |
| CYBB | Osteoporosis, Postmenopausal | 19.89 |
| COL11A1 | Osteoporosis, Postmenopausal | 19.88 |
| FASN | Osteoporosis, Postmenopausal | 19.87 |
| ZNF697 | Osteoporosis, Postmenopausal | 19.87 |
| IL13 | Osteoporosis, Postmenopausal | 19.86 |
| NUCB2 | Osteoporosis, Postmenopausal | 19.85 |
| ACKR3 | Osteoporosis, Postmenopausal | 19.84 |
| EIF2S2 | Osteoporosis, Postmenopausal | 19.84 |
| HSP90AA1 | Osteoporosis, Postmenopausal | 19.84 |
| NR4A1 | Osteoporosis, Postmenopausal | 19.84 |
| LEFTY2 | Osteoporosis, Postmenopausal | 19.82 |
| SCD | Osteoporosis, Postmenopausal | 19.82 |
| CD24 | Osteoporosis, Postmenopausal | 19.81 |
| MYBL2 | Osteoporosis, Postmenopausal | 19.8 |
| IGF1R | Osteoporosis, Postmenopausal | 19.79 |
| ATM | Osteoporosis, Postmenopausal | 19.77 |
| FOXA2 | Osteoporosis, Postmenopausal | 19.77 |
| SMC4 | Osteoporosis, Postmenopausal | 19.77 |
| TRIP13 | Osteoporosis, Postmenopausal | 19.77 |
| EIF4EBP1 | Osteoporosis, Postmenopausal | 19.76 |
| CRISPLD2 | Osteoporosis, Postmenopausal | 19.75 |
| GRB10 | Osteoporosis, Postmenopausal | 19.75 |
| SYK | Osteoporosis, Postmenopausal | 19.74 |
| TNS1 | Osteoporosis, Postmenopausal | 19.74 |
| CDT1 | Osteoporosis, Postmenopausal | 19.73 |
| KDR | Osteoporosis, Postmenopausal | 19.73 |
| NGF | Osteoporosis, Postmenopausal | 19.73 |
| BAK1 | Osteoporosis, Postmenopausal | 19.72 |
| GTSE1 | Osteoporosis, Postmenopausal | 19.71 |
| PCLAF | Osteoporosis, Postmenopausal | 19.71 |
| RAD51AP1 | Osteoporosis, Postmenopausal | 19.7 |
| F3 | Osteoporosis, Postmenopausal | 19.68 |
| FBXO5 | Osteoporosis, Postmenopausal | 19.67 |
| MAP2K1 | Osteoporosis, Postmenopausal | 19.67 |
| CASP8 | Osteoporosis, Postmenopausal | 19.66 |
| NDUFS1 | Osteoporosis, Postmenopausal | 19.66 |
| CLK1 | Osteoporosis, Postmenopausal | 19.65 |
| PAEP | Osteoporosis, Postmenopausal | 19.65 |
| S100P | Osteoporosis, Postmenopausal | 19.65 |
| COL3A1 | Osteoporosis, Postmenopausal | 19.63 |
| SPAG5 | Osteoporosis, Postmenopausal | 19.6 |
| PLA2G7 | Osteoporosis, Postmenopausal | 19.58 |
| CDC45 | Osteoporosis, Postmenopausal | 19.57 |
| HNRNPH1 | Osteoporosis, Postmenopausal | 19.57 |
| HID1 | Osteoporosis, Postmenopausal | 19.56 |
| IL7R | Osteoporosis, Postmenopausal | 19.56 |
| LEPR | Osteoporosis, Postmenopausal | 19.56 |
| HSPD1 | Osteoporosis, Postmenopausal | 19.54 |
| LAMB1 | Osteoporosis, Postmenopausal | 19.54 |
| MAP2K3 | Osteoporosis, Postmenopausal | 19.54 |
| INSIG2 | Osteoporosis, Postmenopausal | 19.51 |
| ROCK2 | Osteoporosis, Postmenopausal | 19.51 |
| UGT1A3 | Osteoporosis, Postmenopausal | 19.51 |
| ABCC4 | Osteoporosis, Postmenopausal | 19.49 |
| CEACAM1 | Osteoporosis, Postmenopausal | 19.49 |
| FASLG | Osteoporosis, Postmenopausal | 19.49 |
| GMNN | Osteoporosis, Postmenopausal | 19.48 |
| KRT5 | Osteoporosis, Postmenopausal | 19.48 |
| P4HA3 | Osteoporosis, Postmenopausal | 19.48 |
| CD44 | Osteoporosis, Postmenopausal | 19.46 |
| HSPA4L | Osteoporosis, Postmenopausal | 19.45 |
| ALDH1B1 | Osteoporosis, Postmenopausal | 19.44 |
| PON1 | Osteoporosis, Postmenopausal | 19.43 |
| CAR3 | Osteoporosis, Postmenopausal | 19.42 |
| ISG20 | Osteoporosis, Postmenopausal | 19.42 |
| CD38 | Osteoporosis, Postmenopausal | 19.39 |
| MYBL1 | Osteoporosis, Postmenopausal | 19.39 |
| SLC8A1 | Osteoporosis, Postmenopausal | 19.39 |
| ABL1 | Osteoporosis, Postmenopausal | 19.38 |
| FAS | Osteoporosis, Postmenopausal | 19.38 |
| APP | Osteoporosis, Postmenopausal | 19.36 |
| MXD1 | Osteoporosis, Postmenopausal | 19.36 |
| JUP | Osteoporosis, Postmenopausal | 19.34 |
| TACC3 | Osteoporosis, Postmenopausal | 19.34 |
| EIF5 | Osteoporosis, Postmenopausal | 19.33 |
| NRIP1 | Osteoporosis, Postmenopausal | 19.33 |
| RGS13 | Osteoporosis, Postmenopausal | 19.33 |
| CYP4A14 | Osteoporosis, Postmenopausal | 19.32 |
| HAL | Osteoporosis, Postmenopausal | 19.32 |
| CPE | Osteoporosis, Postmenopausal | 19.31 |
| MYB | Osteoporosis, Postmenopausal | 19.3 |
| CD7 | Osteoporosis, Postmenopausal | 19.25 |
| HELLS | Osteoporosis, Postmenopausal | 19.25 |
| MIR20B | Osteoporosis, Postmenopausal | 19.23 |
| MMP10 | Osteoporosis, Postmenopausal | 19.23 |
| RACGAP1 | Osteoporosis, Postmenopausal | 19.23 |
| SDC1 | Osteoporosis, Postmenopausal | 19.23 |
| LIFR | Osteoporosis, Postmenopausal | 19.22 |
| UGT2B15 | Osteoporosis, Postmenopausal | 19.22 |
| WARS1 | Osteoporosis, Postmenopausal | 19.22 |
| SDHA | Osteoporosis, Postmenopausal | 19.21 |
| CDCA3 | Osteoporosis, Postmenopausal | 19.2 |
| HNRNPA2B1 | Osteoporosis, Postmenopausal | 19.17 |
| UGT1A8 | Osteoporosis, Postmenopausal | 19.17 |
| PROS1 | Osteoporosis, Postmenopausal | 19.14 |
| AQP9 | Osteoporosis, Postmenopausal | 19.13 |
| NCOR1 | Osteoporosis, Postmenopausal | 19.12 |
| GPR3 | Osteoporosis, Postmenopausal | 19.09 |
| KLHL24 | Osteoporosis, Postmenopausal | 19.09 |
| LDHA | Osteoporosis, Postmenopausal | 19.08 |
| OXT | Osteoporosis, Postmenopausal | 19.06 |
| LDHB | Osteoporosis, Postmenopausal | 19.05 |
| RRAD | Osteoporosis, Postmenopausal | 19.03 |
| ABCA1 | Osteoporosis, Postmenopausal | 19.02 |
| KPNA2 | Osteoporosis, Postmenopausal | 19.02 |
| SLC1A3 | Osteoporosis, Postmenopausal | 19.02 |
| TFAP2A | Osteoporosis, Postmenopausal | 19.02 |
| THY1 | Osteoporosis, Postmenopausal | 19.02 |
| UGT1A10 | Osteoporosis, Postmenopausal | 19.02 |
| C1S | Osteoporosis, Postmenopausal | 19.01 |
| CILP2 | Osteoporosis, Postmenopausal | 19.01 |
| MAP3K1 | Osteoporosis, Postmenopausal | 19.01 |
| SESN1 | Osteoporosis, Postmenopausal | 19.01 |
| TMSB15A | Osteoporosis, Postmenopausal | 19 |
| MITF | Osteoporosis, Postmenopausal | 18.99 |
| NOX1 | Osteoporosis, Postmenopausal | 18.96 |
| CENPE | Osteoporosis, Postmenopausal | 18.95 |
| RSAD2 | Osteoporosis, Postmenopausal | 18.95 |
| NEK2 | Osteoporosis, Postmenopausal | 18.94 |
| GCLC | Osteoporosis, Postmenopausal | 18.93 |
| HCAR3 | Osteoporosis, Postmenopausal | 18.93 |
| COL1A1 | Osteoporosis, Postmenopausal | 18.9 |
| IL1R2 | Osteoporosis, Postmenopausal | 18.9 |
| NAT2 | Osteoporosis, Postmenopausal | 18.9 |
| TTK | Osteoporosis, Postmenopausal | 18.9 |
| TUBB6 | Osteoporosis, Postmenopausal | 18.87 |
| CALCR | Osteoporosis, Postmenopausal | 18.84 |
| DLGAP5 | Osteoporosis, Postmenopausal | 18.84 |
| HSPA1A | Osteoporosis, Postmenopausal | 18.84 |
| RET | Osteoporosis, Postmenopausal | 18.82 |
| CHRM3 | Osteoporosis, Postmenopausal | 18.81 |
| UCN | Osteoporosis, Postmenopausal | 18.81 |
| EGLN3 | Osteoporosis, Postmenopausal | 18.8 |
| KIF2C | Osteoporosis, Postmenopausal | 18.8 |
| RBP4 | Osteoporosis, Postmenopausal | 18.8 |
| PRSS23 | Osteoporosis, Postmenopausal | 18.79 |
| UGT1A7 | Osteoporosis, Postmenopausal | 18.79 |
| HSPA4 | Osteoporosis, Postmenopausal | 18.78 |
| NDC80 | Osteoporosis, Postmenopausal | 18.78 |
| CYP26B1 | Osteoporosis, Postmenopausal | 18.77 |
| SMAP2 | Osteoporosis, Postmenopausal | 18.77 |
| MMP7 | Osteoporosis, Postmenopausal | 18.76 |
| PRKDC | Osteoporosis, Postmenopausal | 18.76 |
| TOP2A | Osteoporosis, Postmenopausal | 18.75 |
| TCF4 | Osteoporosis, Postmenopausal | 18.74 |
| EFCAB7 | Osteoporosis, Postmenopausal | 18.7 |
| TFRC | Osteoporosis, Postmenopausal | 18.7 |
| APOE | Osteoporosis, Postmenopausal | 18.69 |
| TNFSF10 | Osteoporosis, Postmenopausal | 18.68 |
| RGS16 | Osteoporosis, Postmenopausal | 18.67 |
| SLC27A2 | Osteoporosis, Postmenopausal | 18.67 |
| TNFRSF9 | Osteoporosis, Postmenopausal | 18.66 |
| CFD | Osteoporosis, Postmenopausal | 18.65 |
| KIF23 | Osteoporosis, Postmenopausal | 18.65 |
| NEMP1 | Osteoporosis, Postmenopausal | 18.63 |
| SCNN1A | Osteoporosis, Postmenopausal | 18.63 |
| PPARA | Osteoporosis, Postmenopausal | 18.61 |
| SULT1E1 | Osteoporosis, Postmenopausal | 18.61 |
| HECTD4 | Osteoporosis, Postmenopausal | 18.6 |
| MCL1 | Osteoporosis, Postmenopausal | 18.6 |
| ZBTB24 | Osteoporosis, Postmenopausal | 18.59 |
| ADA | Osteoporosis, Postmenopausal | 18.58 |
| VWF | Osteoporosis, Postmenopausal | 18.58 |
| ASPM | Osteoporosis, Postmenopausal | 18.57 |
| LPL | Osteoporosis, Postmenopausal | 18.57 |
| MCM7 | Osteoporosis, Postmenopausal | 18.57 |
| BRCA2 | Osteoporosis, Postmenopausal | 18.54 |
| B3GNTL1 | Osteoporosis, Postmenopausal | 18.53 |
| ANKMY2 | Osteoporosis, Postmenopausal | 18.5 |
| KCNC4 | Osteoporosis, Postmenopausal | 18.5 |
| MSI1 | Osteoporosis, Postmenopausal | 18.5 |
| CCKAR | Osteoporosis, Postmenopausal | 18.49 |
| DHX38 | Osteoporosis, Postmenopausal | 18.49 |
| MORN2 | Osteoporosis, Postmenopausal | 18.49 |
| HSD17B1 | Osteoporosis, Postmenopausal | 18.48 |
| CAPN1 | Osteoporosis, Postmenopausal | 18.46 |
| CPNE7 | Osteoporosis, Postmenopausal | 18.46 |
| GDF15 | Osteoporosis, Postmenopausal | 18.46 |
| NHSL3 | Osteoporosis, Postmenopausal | 18.46 |
| CASKIN2 | Osteoporosis, Postmenopausal | 18.45 |
| CCL11 | Osteoporosis, Postmenopausal | 18.43 |
| IL12B | Osteoporosis, Postmenopausal | 18.43 |
| SPIN4 | Osteoporosis, Postmenopausal | 18.43 |
| BAZ2A | Osteoporosis, Postmenopausal | 18.42 |
| COL5A1 | Osteoporosis, Postmenopausal | 18.42 |
| NREP | Osteoporosis, Postmenopausal | 18.42 |
| PFKFB3 | Osteoporosis, Postmenopausal | 18.42 |
| EZH2 | Osteoporosis, Postmenopausal | 18.41 |
| IFT88 | Osteoporosis, Postmenopausal | 18.4 |
| FHL2 | Osteoporosis, Postmenopausal | 18.39 |
| PTEN | Osteoporosis, Postmenopausal | 18.39 |
| TMPRSS2 | Osteoporosis, Postmenopausal | 18.39 |
| DNAJA1 | Osteoporosis, Postmenopausal | 18.38 |
| KIF11 | Osteoporosis, Postmenopausal | 18.38 |
| MATN3 | Osteoporosis, Postmenopausal | 18.37 |
| NRF1 | Osteoporosis, Postmenopausal | 18.37 |
| CAPN15 | Osteoporosis, Postmenopausal | 18.36 |
| STAT1 | Osteoporosis, Postmenopausal | 18.36 |
| COL18A1 | Osteoporosis, Postmenopausal | 18.34 |
| ITGB3 | Osteoporosis, Postmenopausal | 18.34 |
| POLR2C | Osteoporosis, Postmenopausal | 18.34 |
| SPP2 | Osteoporosis, Postmenopausal | 18.34 |
| TNFRSF10A | Osteoporosis, Postmenopausal | 18.34 |
| MCM4 | Osteoporosis, Postmenopausal | 18.33 |
| TYR | Osteoporosis, Postmenopausal | 18.32 |
| FGFR3 | Osteoporosis, Postmenopausal | 18.31 |
| IL34 | Osteoporosis, Postmenopausal | 18.31 |
| ORM1 | Osteoporosis, Postmenopausal | 18.31 |
| SLC7A11 | Osteoporosis, Postmenopausal | 18.31 |
| APC | Osteoporosis, Postmenopausal | 18.3 |
| BMP8B | Osteoporosis, Postmenopausal | 18.3 |
| LY75 | Osteoporosis, Postmenopausal | 18.3 |
| SLC2A1 | Osteoporosis, Postmenopausal | 18.3 |
| CSF1R | Osteoporosis, Postmenopausal | 18.29 |
| DNAJB4 | Osteoporosis, Postmenopausal | 18.29 |
| GPAM | Osteoporosis, Postmenopausal | 18.29 |
| PRKACA | Osteoporosis, Postmenopausal | 18.29 |
| ACTA1 | Osteoporosis, Postmenopausal | 18.28 |
| KATNAL1 | Osteoporosis, Postmenopausal | 18.27 |
| OSGIN1 | Osteoporosis, Postmenopausal | 18.25 |
| PDCD4 | Osteoporosis, Postmenopausal | 18.25 |
| STC2 | Osteoporosis, Postmenopausal | 18.25 |
| ENTPD3 | Osteoporosis, Postmenopausal | 18.24 |
| HSD17B4 | Osteoporosis, Postmenopausal | 18.24 |
| HSPE1 | Osteoporosis, Postmenopausal | 18.24 |
| UHRF1 | Osteoporosis, Postmenopausal | 18.24 |
| BUB1 | Osteoporosis, Postmenopausal | 18.23 |
| EFR3A | Osteoporosis, Postmenopausal | 18.23 |
| S100A6 | Osteoporosis, Postmenopausal | 18.23 |
| SSH1 | Osteoporosis, Postmenopausal | 18.21 |
| ARG2 | Osteoporosis, Postmenopausal | 18.2 |
| PROCA1 | Osteoporosis, Postmenopausal | 18.2 |
| WAS | Osteoporosis, Postmenopausal | 18.2 |
| ATF2 | Osteoporosis, Postmenopausal | 18.19 |
| PRLR | Osteoporosis, Postmenopausal | 18.19 |
| ABCC3 | Osteoporosis, Postmenopausal | 18.18 |
| APPBP2 | Osteoporosis, Postmenopausal | 18.18 |
| FBXO32 | Osteoporosis, Postmenopausal | 18.18 |
| JAK1 | Osteoporosis, Postmenopausal | 18.18 |
| PBK | Osteoporosis, Postmenopausal | 18.18 |
| SHOX2 | Osteoporosis, Postmenopausal | 18.18 |
| SLC22A5 | Osteoporosis, Postmenopausal | 18.18 |
| SYNGR3 | Osteoporosis, Postmenopausal | 18.18 |
| CITED2 | Osteoporosis, Postmenopausal | 18.16 |
| CDK4 | Osteoporosis, Postmenopausal | 18.14 |
| ANXA3 | Osteoporosis, Postmenopausal | 18.13 |
| DNMT3B | Osteoporosis, Postmenopausal | 18.12 |
| NPM1 | Osteoporosis, Postmenopausal | 18.12 |
| TMEM40 | Osteoporosis, Postmenopausal | 18.12 |
| HOXB6 | Osteoporosis, Postmenopausal | 18.11 |
| PWP2 | Osteoporosis, Postmenopausal | 18.11 |
| DRD1 | Osteoporosis, Postmenopausal | 18.1 |
| CDKN3 | Osteoporosis, Postmenopausal | 18.09 |
| MIR29C | Osteoporosis, Postmenopausal | 18.08 |
| CCNE1 | Osteoporosis, Postmenopausal | 18.07 |
| H1-5 | Osteoporosis, Postmenopausal | 18.07 |
| BCL6 | Osteoporosis, Postmenopausal | 18.06 |
| HMGB2 | Osteoporosis, Postmenopausal | 18.06 |
| HRC | Osteoporosis, Postmenopausal | 18.06 |
| PGS1 | Osteoporosis, Postmenopausal | 18.06 |
| GIPC1 | Osteoporosis, Postmenopausal | 18.05 |
| PDIA3 | Osteoporosis, Postmenopausal | 18.05 |
| TMEM263 | Osteoporosis, Postmenopausal | 18.05 |
| BUB1B | Osteoporosis, Postmenopausal | 18.04 |
| CD74 | Osteoporosis, Postmenopausal | 18.04 |
| SEZ6L2 | Osteoporosis, Postmenopausal | 18.04 |
| EEF2 | Osteoporosis, Postmenopausal | 18.03 |
| NRG2 | Osteoporosis, Postmenopausal | 18.03 |
| HSPB8 | Osteoporosis, Postmenopausal | 18.02 |
| GSN | Osteoporosis, Postmenopausal | 18.01 |
| PRKCE | Osteoporosis, Postmenopausal | 18.01 |
| ABCC1 | Osteoporosis, Postmenopausal | 18 |
| MAP2K6 | Osteoporosis, Postmenopausal | 18 |
| TMC4 | Osteoporosis, Postmenopausal | 18 |
| FANCB | Osteoporosis, Postmenopausal | 17.99 |
| GSR | Osteoporosis, Postmenopausal | 17.99 |
| MPHOSPH9 | Osteoporosis, Postmenopausal | 17.99 |
| SULT5A1 | Osteoporosis, Postmenopausal | 17.99 |
| MYH7 | Osteoporosis, Postmenopausal | 17.98 |
| TGFBI | Osteoporosis, Postmenopausal | 17.98 |
| FADD | Osteoporosis, Postmenopausal | 17.97 |
| ADIPOQ | Osteoporosis, Postmenopausal | 17.96 |
| TP53I11 | Osteoporosis, Postmenopausal | 17.96 |
| DGAT2 | Osteoporosis, Postmenopausal | 17.95 |
| CENPF | Osteoporosis, Postmenopausal | 17.94 |
| COL9A3 | Osteoporosis, Postmenopausal | 17.94 |
| ENPP2 | Osteoporosis, Postmenopausal | 17.93 |
| JAG1 | Osteoporosis, Postmenopausal | 17.93 |
| ZNF367 | Osteoporosis, Postmenopausal | 17.92 |
| CEP78 | Osteoporosis, Postmenopausal | 17.91 |
| PLEKHF2 | Osteoporosis, Postmenopausal | 17.91 |
| ABCB1 | Osteoporosis, Postmenopausal | 17.9 |
| CDKN1C | Osteoporosis, Postmenopausal | 17.89 |
| CIP2A | Osteoporosis, Postmenopausal | 17.89 |
| PIP5K1B | Osteoporosis, Postmenopausal | 17.89 |
| FGFR1 | Osteoporosis, Postmenopausal | 17.88 |
| ENC1 | Osteoporosis, Postmenopausal | 17.87 |
| SLC6A4 | Osteoporosis, Postmenopausal | 17.86 |
| CXCL5 | Osteoporosis, Postmenopausal | 17.85 |
| MMD2 | Osteoporosis, Postmenopausal | 17.85 |
| SLC26A3 | Osteoporosis, Postmenopausal | 17.84 |
| DPP4 | Osteoporosis, Postmenopausal | 17.83 |
| ITGBL1 | Osteoporosis, Postmenopausal | 17.83 |
| TMEM156 | Osteoporosis, Postmenopausal | 17.83 |
| HCFC1R1 | Osteoporosis, Postmenopausal | 17.82 |
| IGFBP2 | Osteoporosis, Postmenopausal | 17.82 |
| NAP1L4 | Osteoporosis, Postmenopausal | 17.82 |
| SULT1A3 | Osteoporosis, Postmenopausal | 17.82 |
| TMEM107 | Osteoporosis, Postmenopausal | 17.81 |
| CYB5A | Osteoporosis, Postmenopausal | 17.79 |
| SUSD3 | Osteoporosis, Postmenopausal | 17.79 |
| GHRH | Osteoporosis, Postmenopausal | 17.78 |
| HOOK1 | Osteoporosis, Postmenopausal | 17.78 |
| MYC | Osteoporosis, Postmenopausal | 17.78 |
| SBF2 | Osteoporosis, Postmenopausal | 17.78 |
| NMRK1 | Osteoporosis, Postmenopausal | 17.74 |
| TP53BP2 | Osteoporosis, Postmenopausal | 17.74 |
| ZNF395 | Osteoporosis, Postmenopausal | 17.74 |
| LRATD1 | Osteoporosis, Postmenopausal | 17.72 |
| NCOA6 | Osteoporosis, Postmenopausal | 17.72 |
| S100A2 | Osteoporosis, Postmenopausal | 17.72 |
| STAR | Osteoporosis, Postmenopausal | 17.72 |
| TSPAN1 | Osteoporosis, Postmenopausal | 17.72 |
| CYP3A2 | Osteoporosis, Postmenopausal | 17.71 |
| CBR1 | Osteoporosis, Postmenopausal | 17.7 |
| HTR2A | Osteoporosis, Postmenopausal | 17.7 |
| PDGFRA | Osteoporosis, Postmenopausal | 17.68 |
| EP300 | Osteoporosis, Postmenopausal | 17.67 |
| IRS2 | Osteoporosis, Postmenopausal | 17.67 |
| CYP26A1 | Osteoporosis, Postmenopausal | 17.66 |
| EPO | Osteoporosis, Postmenopausal | 17.66 |
| MAZ | Osteoporosis, Postmenopausal | 17.66 |
| SLC6A11 | Osteoporosis, Postmenopausal | 17.66 |
| ASS1 | Osteoporosis, Postmenopausal | 17.65 |
| CA2 | Osteoporosis, Postmenopausal | 17.65 |
| NR1D1 | Osteoporosis, Postmenopausal | 17.65 |
| PIK3CA | Osteoporosis, Postmenopausal | 17.64 |
| VCPIP1 | Osteoporosis, Postmenopausal | 17.64 |
| ABCG2 | Osteoporosis, Postmenopausal | 17.63 |
| KSR1 | Osteoporosis, Postmenopausal | 17.63 |
| MCM2 | Osteoporosis, Postmenopausal | 17.63 |
| HACD2 | Osteoporosis, Postmenopausal | 17.61 |
| NFIL3 | Osteoporosis, Postmenopausal | 17.61 |
| ZFP36 | Osteoporosis, Postmenopausal | 17.61 |
| CKB | Osteoporosis, Postmenopausal | 17.6 |
| STK26 | Osteoporosis, Postmenopausal | 17.6 |
| RAB11B | Osteoporosis, Postmenopausal | 17.58 |
| RHOF | Osteoporosis, Postmenopausal | 17.58 |
| LINGO1 | Osteoporosis, Postmenopausal | 17.57 |
| PIAS3 | Osteoporosis, Postmenopausal | 17.57 |
| UBE2C | Osteoporosis, Postmenopausal | 17.57 |
| ACOX3 | Osteoporosis, Postmenopausal | 17.56 |
| DUOX1 | Osteoporosis, Postmenopausal | 17.56 |
| ITSN2 | Osteoporosis, Postmenopausal | 17.56 |
| MDM1 | Osteoporosis, Postmenopausal | 17.56 |
| EDNRA | Osteoporosis, Postmenopausal | 17.55 |
| FKBP3 | Osteoporosis, Postmenopausal | 17.55 |
| GPT | Osteoporosis, Postmenopausal | 17.55 |
| HMGCS2 | Osteoporosis, Postmenopausal | 17.55 |
| SMURF1 | Osteoporosis, Postmenopausal | 17.55 |
| CAV3 | Osteoporosis, Postmenopausal | 17.54 |
| DNAJB9 | Osteoporosis, Postmenopausal | 17.54 |
| MRPL33 | Osteoporosis, Postmenopausal | 17.53 |
| KIT | Osteoporosis, Postmenopausal | 17.52 |
| MGLL | Osteoporosis, Postmenopausal | 17.52 |
| CYP27A1 | Osteoporosis, Postmenopausal | 17.51 |
| FGF1 | Osteoporosis, Postmenopausal | 17.51 |
| LCORL | Osteoporosis, Postmenopausal | 17.51 |
| SH3YL1 | Osteoporosis, Postmenopausal | 17.51 |
| TMEM64 | Osteoporosis, Postmenopausal | 17.5 |
| VDAC1 | Osteoporosis, Postmenopausal | 17.5 |
| CASP6 | Osteoporosis, Postmenopausal | 17.49 |
| HERPUD1 | Osteoporosis, Postmenopausal | 17.49 |
| MDFIC | Osteoporosis, Postmenopausal | 17.49 |
| MAX | Osteoporosis, Postmenopausal | 17.48 |
| PER2 | Osteoporosis, Postmenopausal | 17.48 |
| SLC1A2 | Osteoporosis, Postmenopausal | 17.48 |
| SLC22A1 | Osteoporosis, Postmenopausal | 17.46 |
| SLC25A19 | Osteoporosis, Postmenopausal | 17.46 |
| MCM6 | Osteoporosis, Postmenopausal | 17.45 |
| MRPL20 | Osteoporosis, Postmenopausal | 17.45 |
| MRPL49 | Osteoporosis, Postmenopausal | 17.45 |
| S100A16 | Osteoporosis, Postmenopausal | 17.45 |
| ARHGEF39 | Osteoporosis, Postmenopausal | 17.44 |
| ACYP1 | Osteoporosis, Postmenopausal | 17.43 |
| RAB23 | Osteoporosis, Postmenopausal | 17.41 |
| TNFRSF12A | Osteoporosis, Postmenopausal | 17.41 |
| RARA | Osteoporosis, Postmenopausal | 17.4 |
| JUND | Osteoporosis, Postmenopausal | 17.39 |
| INHBA | Osteoporosis, Postmenopausal | 17.38 |
| ANXA5 | Osteoporosis, Postmenopausal | 17.36 |
| CEP152 | Osteoporosis, Postmenopausal | 17.36 |
| IGFBP5 | Osteoporosis, Postmenopausal | 17.36 |
| AURKB | Osteoporosis, Postmenopausal | 17.33 |
| UGT1A9 | Osteoporosis, Postmenopausal | 17.33 |
| NARF | Osteoporosis, Postmenopausal | 17.32 |
| SEC14L1 | Osteoporosis, Postmenopausal | 17.32 |
| UHMK1 | Osteoporosis, Postmenopausal | 17.32 |
| CILP | Osteoporosis, Postmenopausal | 17.31 |
| LGALS3 | Osteoporosis, Postmenopausal | 17.31 |
| THPO | Osteoporosis, Postmenopausal | 17.31 |
| ALPP | Osteoporosis, Postmenopausal | 17.29 |
| OTUD7B | Osteoporosis, Postmenopausal | 17.29 |
| PSAT1 | Osteoporosis, Postmenopausal | 17.28 |
| TAF9 | Osteoporosis, Postmenopausal | 17.28 |
| TFAM | Osteoporosis, Postmenopausal | 17.28 |
| TNC | Osteoporosis, Postmenopausal | 17.28 |
| ARHGEF16 | Osteoporosis, Postmenopausal | 17.26 |
| EHBP1L1 | Osteoporosis, Postmenopausal | 17.26 |
| IL32 | Osteoporosis, Postmenopausal | 17.26 |
| INS | Osteoporosis, Postmenopausal | 17.26 |
| ALPI | Osteoporosis, Postmenopausal | 17.25 |
| IL18 | Osteoporosis, Postmenopausal | 17.25 |
| CD3G | Osteoporosis, Postmenopausal | 17.23 |
| IBSP | Osteoporosis, Postmenopausal | 17.23 |
| TENT5B | Osteoporosis, Postmenopausal | 17.23 |
| VTG2 | Osteoporosis, Postmenopausal | 17.23 |
| SLC2A3 | Osteoporosis, Postmenopausal | 17.22 |
| ARNT | Osteoporosis, Postmenopausal | 17.21 |
| BIRC2 | Osteoporosis, Postmenopausal | 17.21 |
| EDNRB | Osteoporosis, Postmenopausal | 17.21 |
| ESRP1 | Osteoporosis, Postmenopausal | 17.21 |
| SETD5 | Osteoporosis, Postmenopausal | 17.2 |
| ETNK2 | Osteoporosis, Postmenopausal | 17.17 |
| GPC6 | Osteoporosis, Postmenopausal | 17.17 |
| HSD11B2 | Osteoporosis, Postmenopausal | 17.17 |
| MTFR2 | Osteoporosis, Postmenopausal | 17.17 |
| RMI2 | Osteoporosis, Postmenopausal | 17.17 |
| ITGB3BP | Osteoporosis, Postmenopausal | 17.16 |
| PITX2 | Osteoporosis, Postmenopausal | 17.16 |
| CDK2AP2 | Osteoporosis, Postmenopausal | 17.14 |
| NLRP12 | Osteoporosis, Postmenopausal | 17.14 |
| ALB | Osteoporosis, Postmenopausal | 17.13 |
| ALPK2 | Osteoporosis, Postmenopausal | 17.13 |
| BHLHE40 | Osteoporosis, Postmenopausal | 17.13 |
| DIO2 | Osteoporosis, Postmenopausal | 17.13 |
| ESPNP | Osteoporosis, Postmenopausal | 17.13 |
| PRKAA1 | Osteoporosis, Postmenopausal | 17.13 |
| RAB38 | Osteoporosis, Postmenopausal | 17.13 |
| SLC7A5 | Osteoporosis, Postmenopausal | 17.13 |
| FBXO3 | Osteoporosis, Postmenopausal | 17.12 |
| TIMP3 | Osteoporosis, Postmenopausal | 17.12 |
| ZSWIM6 | Osteoporosis, Postmenopausal | 17.12 |
| ABHD3 | Osteoporosis, Postmenopausal | 17.11 |
| CBFB | Osteoporosis, Postmenopausal | 17.11 |
| NPR1 | Osteoporosis, Postmenopausal | 17.11 |
| CCNE2 | Osteoporosis, Postmenopausal | 17.1 |
| ATXN3 | Osteoporosis, Postmenopausal | 17.09 |
| LOX | Osteoporosis, Postmenopausal | 17.09 |
| RNF43 | Osteoporosis, Postmenopausal | 17.09 |
| SRPK1 | Osteoporosis, Postmenopausal | 17.09 |
| PASK | Osteoporosis, Postmenopausal | 17.08 |
| BCAM | Osteoporosis, Postmenopausal | 17.07 |
| FGF23 | Osteoporosis, Postmenopausal | 17.07 |
| GAS2L3 | Osteoporosis, Postmenopausal | 17.07 |
| PIF1 | Osteoporosis, Postmenopausal | 17.07 |
| RGS17 | Osteoporosis, Postmenopausal | 17.07 |
| SCAPER | Osteoporosis, Postmenopausal | 17.07 |
| VRK1 | Osteoporosis, Postmenopausal | 17.07 |
| CACNA1I | Osteoporosis, Postmenopausal | 17.06 |
| EDN2 | Osteoporosis, Postmenopausal | 17.06 |
| FGD6 | Osteoporosis, Postmenopausal | 17.06 |
| ID3 | Osteoporosis, Postmenopausal | 17.06 |
| OTUB2 | Osteoporosis, Postmenopausal | 17.06 |
| UTP20 | Osteoporosis, Postmenopausal | 17.06 |
| WWC2 | Osteoporosis, Postmenopausal | 17.06 |
| IDI1 | Osteoporosis, Postmenopausal | 17.05 |
| HOXA5 | Osteoporosis, Postmenopausal | 17.04 |
| PTGFRN | Osteoporosis, Postmenopausal | 17.04 |
| RALB | Osteoporosis, Postmenopausal | 17.04 |
| CAMP | Osteoporosis, Postmenopausal | 17.03 |
| CRLF1 | Osteoporosis, Postmenopausal | 17.03 |
| RAD51 | Osteoporosis, Postmenopausal | 17.03 |
| BMP2K | Osteoporosis, Postmenopausal | 17.02 |
| ERCC3 | Osteoporosis, Postmenopausal | 17.02 |
| GDPD5 | Osteoporosis, Postmenopausal | 17.02 |
| CYP2A1 | Osteoporosis, Postmenopausal | 17 |
| HSPH1 | Osteoporosis, Postmenopausal | 17 |
| NUPR1 | Osteoporosis, Postmenopausal | 17 |
| TBC1D8 | Osteoporosis, Postmenopausal | 17 |
| CXCL1 | Osteoporosis, Postmenopausal | 16.98 |
| NMU | Osteoporosis, Postmenopausal | 16.98 |
| PLCD3 | Osteoporosis, Postmenopausal | 16.98 |
| MASTL | Osteoporosis, Postmenopausal | 16.97 |
| MPC1 | Osteoporosis, Postmenopausal | 16.97 |
| HSP90AB1 | Osteoporosis, Postmenopausal | 16.96 |
| SLA | Osteoporosis, Postmenopausal | 16.96 |
| CDCA7L | Osteoporosis, Postmenopausal | 16.95 |
| KLF4 | Osteoporosis, Postmenopausal | 16.95 |
| FANCC | Osteoporosis, Postmenopausal | 16.94 |
| MPP7 | Osteoporosis, Postmenopausal | 16.94 |
| SLC44A4 | Osteoporosis, Postmenopausal | 16.94 |
| KLK1 | Osteoporosis, Postmenopausal | 16.93 |
| NDUFS3 | Osteoporosis, Postmenopausal | 16.93 |
| RHOA | Osteoporosis, Postmenopausal | 16.93 |
| TGM2 | Osteoporosis, Postmenopausal | 16.93 |
| BAZ2B | Osteoporosis, Postmenopausal | 16.92 |
| MIR22 | Osteoporosis, Postmenopausal | 16.92 |
| PPARGC1A | Osteoporosis, Postmenopausal | 16.92 |
| MEGF6 | Osteoporosis, Postmenopausal | 16.91 |
| RERG | Osteoporosis, Postmenopausal | 16.91 |
| SQLE | Osteoporosis, Postmenopausal | 16.91 |
| MSMO1 | Osteoporosis, Postmenopausal | 16.9 |
| CYP19A1B | Osteoporosis, Postmenopausal | 16.89 |
| IRX3 | Osteoporosis, Postmenopausal | 16.89 |
| PGPEP1 | Osteoporosis, Postmenopausal | 16.89 |
| PLPP2 | Osteoporosis, Postmenopausal | 16.89 |
| RBM24 | Osteoporosis, Postmenopausal | 16.89 |
| SPAG1 | Osteoporosis, Postmenopausal | 16.89 |
| ESPN | Osteoporosis, Postmenopausal | 16.88 |
| KAZN | Osteoporosis, Postmenopausal | 16.88 |
| BDNF | Osteoporosis, Postmenopausal | 16.87 |
| CASP12 | Osteoporosis, Postmenopausal | 16.86 |
| CENPJ | Osteoporosis, Postmenopausal | 16.86 |
| CYP3A11 | Osteoporosis, Postmenopausal | 16.86 |
| TAOK3 | Osteoporosis, Postmenopausal | 16.86 |
| CSGALNACT1 | Osteoporosis, Postmenopausal | 16.85 |
| SP5 | Osteoporosis, Postmenopausal | 16.84 |
| CHUK | Osteoporosis, Postmenopausal | 16.82 |
| MDC1 | Osteoporosis, Postmenopausal | 16.82 |
| MMP16 | Osteoporosis, Postmenopausal | 16.81 |
| SLC30A10 | Osteoporosis, Postmenopausal | 16.81 |
| TCIM | Osteoporosis, Postmenopausal | 16.81 |
| SALL1 | Osteoporosis, Postmenopausal | 16.8 |
| SCT | Osteoporosis, Postmenopausal | 16.8 |
| SLC12A6 | Osteoporosis, Postmenopausal | 16.78 |
| FKBP1B | Osteoporosis, Postmenopausal | 16.77 |
| PKIB | Osteoporosis, Postmenopausal | 16.77 |
| RALGDS | Osteoporosis, Postmenopausal | 16.77 |
| THBS1 | Osteoporosis, Postmenopausal | 16.77 |
| CLIP4 | Osteoporosis, Postmenopausal | 16.76 |
| AP1G1 | Osteoporosis, Postmenopausal | 16.75 |
| FGF8 | Osteoporosis, Postmenopausal | 16.75 |
| FOXA3 | Osteoporosis, Postmenopausal | 16.75 |
| HSD11B1 | Osteoporosis, Postmenopausal | 16.75 |
| RASSF2 | Osteoporosis, Postmenopausal | 16.75 |
| AQP5 | Osteoporosis, Postmenopausal | 16.74 |
| NOTCH4 | Osteoporosis, Postmenopausal | 16.74 |
| SMIM3 | Osteoporosis, Postmenopausal | 16.74 |
| SOX9 | Osteoporosis, Postmenopausal | 16.74 |
| ENAH | Osteoporosis, Postmenopausal | 16.73 |
| IGFBP1 | Osteoporosis, Postmenopausal | 16.73 |
| RIN2 | Osteoporosis, Postmenopausal | 16.73 |
| CHTF18 | Osteoporosis, Postmenopausal | 16.72 |
| DDIAS | Osteoporosis, Postmenopausal | 16.72 |
| FLT1 | Osteoporosis, Postmenopausal | 16.72 |
| IL2 | Osteoporosis, Postmenopausal | 16.72 |
| LACTB2 | Osteoporosis, Postmenopausal | 16.72 |
| MN1 | Osteoporosis, Postmenopausal | 16.72 |
| PPARD | Osteoporosis, Postmenopausal | 16.72 |
| SELE | Osteoporosis, Postmenopausal | 16.72 |
| STIL | Osteoporosis, Postmenopausal | 16.72 |
| CCL12 | Osteoporosis, Postmenopausal | 16.71 |
| RGS10 | Osteoporosis, Postmenopausal | 16.71 |
| SLC12A5 | Osteoporosis, Postmenopausal | 16.71 |
| BRCA1 | Osteoporosis, Postmenopausal | 16.69 |
| CARD10 | Osteoporosis, Postmenopausal | 16.68 |
| CELSR2 | Osteoporosis, Postmenopausal | 16.68 |
| GCLM | Osteoporosis, Postmenopausal | 16.68 |
| HK2 | Osteoporosis, Postmenopausal | 16.68 |
| IVL | Osteoporosis, Postmenopausal | 16.68 |
| PARPBP | Osteoporosis, Postmenopausal | 16.68 |
| SGO2 | Osteoporosis, Postmenopausal | 16.68 |
| TMOD1 | Osteoporosis, Postmenopausal | 16.68 |
| CPN1 | Osteoporosis, Postmenopausal | 16.67 |
| CRABP1 | Osteoporosis, Postmenopausal | 16.67 |
| PITPNC1 | Osteoporosis, Postmenopausal | 16.67 |
| PTGER1 | Osteoporosis, Postmenopausal | 16.67 |
| TUFT1 | Osteoporosis, Postmenopausal | 16.67 |
| ATG5 | Osteoporosis, Postmenopausal | 16.66 |
| C2CD2 | Osteoporosis, Postmenopausal | 16.66 |
| COPB2 | Osteoporosis, Postmenopausal | 16.66 |
| CTH | Osteoporosis, Postmenopausal | 16.65 |
| NUP155 | Osteoporosis, Postmenopausal | 16.64 |
| ADGRL2 | Osteoporosis, Postmenopausal | 16.63 |
| CREB3L3 | Osteoporosis, Postmenopausal | 16.63 |
| PIK3R2 | Osteoporosis, Postmenopausal | 16.63 |
| TIPIN | Osteoporosis, Postmenopausal | 16.62 |
| HR | Osteoporosis, Postmenopausal | 16.6 |
| NUDT1 | Osteoporosis, Postmenopausal | 16.6 |
| PIDD1 | Osteoporosis, Postmenopausal | 16.6 |
| ID1 | Osteoporosis, Postmenopausal | 16.59 |
| IER5L | Osteoporosis, Postmenopausal | 16.59 |
| MFAP5 | Osteoporosis, Postmenopausal | 16.59 |
| SNORA61 | Osteoporosis, Postmenopausal | 16.58 |
| CADPS2 | Osteoporosis, Postmenopausal | 16.56 |
| CYP3A23-3A1 | Osteoporosis, Postmenopausal | 16.56 |
| HBEGF | Osteoporosis, Postmenopausal | 16.56 |
| KCND3 | Osteoporosis, Postmenopausal | 16.56 |
| LPGAT1 | Osteoporosis, Postmenopausal | 16.56 |
| SNAI1 | Osteoporosis, Postmenopausal | 16.56 |
| TYRP1 | Osteoporosis, Postmenopausal | 16.56 |
| IL12A | Osteoporosis, Postmenopausal | 16.55 |
| SLC12A4 | Osteoporosis, Postmenopausal | 16.55 |
| UBA5 | Osteoporosis, Postmenopausal | 16.55 |
| ERO1B | Osteoporosis, Postmenopausal | 16.54 |
| SAFB | Osteoporosis, Postmenopausal | 16.54 |
| TFAP2C | Osteoporosis, Postmenopausal | 16.54 |
| XPOT | Osteoporosis, Postmenopausal | 16.53 |
| F8 | Osteoporosis, Postmenopausal | 16.52 |
| NUP153 | Osteoporosis, Postmenopausal | 16.52 |
| PTPN21 | Osteoporosis, Postmenopausal | 16.52 |
| E2F1 | Osteoporosis, Postmenopausal | 16.51 |
| PTPRU | Osteoporosis, Postmenopausal | 16.51 |
| VASN | Osteoporosis, Postmenopausal | 16.51 |
| LSR | Osteoporosis, Postmenopausal | 16.49 |
| ZWILCH | Osteoporosis, Postmenopausal | 16.48 |
| NKX3-1 | Osteoporosis, Postmenopausal | 16.47 |
| RBMS1 | Osteoporosis, Postmenopausal | 16.47 |
| TNPO1 | Osteoporosis, Postmenopausal | 16.47 |
| ENDOD1 | Osteoporosis, Postmenopausal | 16.46 |
| NR0B2 | Osteoporosis, Postmenopausal | 16.46 |
| RHBDF1 | Osteoporosis, Postmenopausal | 16.46 |
| HSPA1B | Osteoporosis, Postmenopausal | 16.45 |
| DSCC1 | Osteoporosis, Postmenopausal | 16.44 |
| HMCN1 | Osteoporosis, Postmenopausal | 16.44 |
| MYH14 | Osteoporosis, Postmenopausal | 16.44 |
| RNASEH2A | Osteoporosis, Postmenopausal | 16.44 |
| TCOF1 | Osteoporosis, Postmenopausal | 16.44 |
| ACAD11 | Osteoporosis, Postmenopausal | 16.43 |
| ADAM9 | Osteoporosis, Postmenopausal | 16.43 |
| CHEK2 | Osteoporosis, Postmenopausal | 16.43 |
| HSPA12A | Osteoporosis, Postmenopausal | 16.43 |
| PCCA | Osteoporosis, Postmenopausal | 16.43 |
| GPX8 | Osteoporosis, Postmenopausal | 16.42 |
| MAP2K5 | Osteoporosis, Postmenopausal | 16.42 |
| PGM2L1 | Osteoporosis, Postmenopausal | 16.42 |
| SLC26A2 | Osteoporosis, Postmenopausal | 16.42 |
| FLOT1 | Osteoporosis, Postmenopausal | 16.4 |
| HTRA2 | Osteoporosis, Postmenopausal | 16.4 |
| NELL2 | Osteoporosis, Postmenopausal | 16.4 |
| ALOX12 | Osteoporosis, Postmenopausal | 16.38 |
| EOMES | Osteoporosis, Postmenopausal | 16.37 |
| CABLES1 | Osteoporosis, Postmenopausal | 16.36 |
| G2E3 | Osteoporosis, Postmenopausal | 16.36 |
| G6PC1 | Osteoporosis, Postmenopausal | 16.36 |
| TYMS | Osteoporosis, Postmenopausal | 16.36 |
| MIR491 | Osteoporosis, Postmenopausal | 16.35 |
| RHOU | Osteoporosis, Postmenopausal | 16.35 |
| SULT2B1 | Osteoporosis, Postmenopausal | 16.35 |
| CGNL1 | Osteoporosis, Postmenopausal | 16.34 |
| GADD45B | Osteoporosis, Postmenopausal | 16.34 |
| GPSM2 | Osteoporosis, Postmenopausal | 16.33 |
| CYCS | Osteoporosis, Postmenopausal | 16.32 |
| TMEM158 | Osteoporosis, Postmenopausal | 16.32 |
| TNFAIP3 | Osteoporosis, Postmenopausal | 16.32 |
| TNRC18 | Osteoporosis, Postmenopausal | 16.32 |
| MATR3 | Osteoporosis, Postmenopausal | 16.31 |
| SAMD4A | Osteoporosis, Postmenopausal | 16.31 |
| YPEL2 | Osteoporosis, Postmenopausal | 16.31 |
| NPPB | Osteoporosis, Postmenopausal | 16.3 |
| ASAP1 | Osteoporosis, Postmenopausal | 16.29 |
| DCBLD2 | Osteoporosis, Postmenopausal | 16.29 |
| PIMREG | Osteoporosis, Postmenopausal | 16.29 |
| SERPINB1 | Osteoporosis, Postmenopausal | 16.29 |
| SH3BGRL | Osteoporosis, Postmenopausal | 16.29 |
| SPTLC2 | Osteoporosis, Postmenopausal | 16.29 |
| DFFB | Osteoporosis, Postmenopausal | 16.28 |
| ELOVL1 | Osteoporosis, Postmenopausal | 16.28 |
| CCPG1 | Osteoporosis, Postmenopausal | 16.27 |
| CFTR | Osteoporosis, Postmenopausal | 16.27 |
| DEFB1 | Osteoporosis, Postmenopausal | 16.27 |
| HDGF | Osteoporosis, Postmenopausal | 16.27 |
| PRKCD | Osteoporosis, Postmenopausal | 16.26 |
| DEPDC1 | Osteoporosis, Postmenopausal | 16.25 |
| LRP4 | Osteoporosis, Postmenopausal | 16.25 |
| AUH | Osteoporosis, Postmenopausal | 16.24 |
| PKM | Osteoporosis, Postmenopausal | 16.24 |
| PTPRG | Osteoporosis, Postmenopausal | 16.24 |
| FLRT3 | Osteoporosis, Postmenopausal | 16.23 |
| RAF1 | Osteoporosis, Postmenopausal | 16.23 |
| RPS6 | Osteoporosis, Postmenopausal | 16.23 |
| FOXC1 | Osteoporosis, Postmenopausal | 16.22 |
| LPAR6 | Osteoporosis, Postmenopausal | 16.22 |
| POM121C | Osteoporosis, Postmenopausal | 16.22 |
| ABI3BP | Osteoporosis, Postmenopausal | 16.21 |
| NAT8 | Osteoporosis, Postmenopausal | 16.21 |
| NUMA1 | Osteoporosis, Postmenopausal | 16.21 |
| OASL | Osteoporosis, Postmenopausal | 16.21 |
| PRDX1 | Osteoporosis, Postmenopausal | 16.21 |
| SCPEP1 | Osteoporosis, Postmenopausal | 16.21 |
| SLC41A2 | Osteoporosis, Postmenopausal | 16.21 |
| TRIM59 | Osteoporosis, Postmenopausal | 16.21 |
| DMBT1 | Osteoporosis, Postmenopausal | 16.2 |
| PSME4 | Osteoporosis, Postmenopausal | 16.2 |
| SLC39A4 | Osteoporosis, Postmenopausal | 16.19 |
| ATF6 | Osteoporosis, Postmenopausal | 16.18 |
| DACT1 | Osteoporosis, Postmenopausal | 16.18 |
| DOCK4 | Osteoporosis, Postmenopausal | 16.18 |
| SKA1 | Osteoporosis, Postmenopausal | 16.18 |
| STK39 | Osteoporosis, Postmenopausal | 16.18 |
| SEMA3B | Osteoporosis, Postmenopausal | 16.17 |
| APAF1 | Osteoporosis, Postmenopausal | 16.16 |
| MT1 | Osteoporosis, Postmenopausal | 16.16 |
| PBX1 | Osteoporosis, Postmenopausal | 16.16 |
| MIR34A | Osteoporosis, Postmenopausal | 16.15 |
| PLXNC1 | Osteoporosis, Postmenopausal | 16.15 |
| RRAS | Osteoporosis, Postmenopausal | 16.15 |
| ARHGEF3 | Osteoporosis, Postmenopausal | 16.14 |
| ATP2A3 | Osteoporosis, Postmenopausal | 16.14 |
| CEL | Osteoporosis, Postmenopausal | 16.14 |
| EHBP1 | Osteoporosis, Postmenopausal | 16.14 |
| GAB1 | Osteoporosis, Postmenopausal | 16.14 |
| PHLDB2 | Osteoporosis, Postmenopausal | 16.14 |
| PTPRE | Osteoporosis, Postmenopausal | 16.13 |
| CYP2S1 | Osteoporosis, Postmenopausal | 16.12 |
| DYRK2 | Osteoporosis, Postmenopausal | 16.12 |
| GFRA2 | Osteoporosis, Postmenopausal | 16.12 |
| FGL1 | Osteoporosis, Postmenopausal | 16.11 |
| KCTD12 | Osteoporosis, Postmenopausal | 16.11 |
| LGALS8 | Osteoporosis, Postmenopausal | 16.11 |
| ATP1B3 | Osteoporosis, Postmenopausal | 16.1 |
| COMT | Osteoporosis, Postmenopausal | 16.1 |
| MIR629 | Osteoporosis, Postmenopausal | 16.1 |
| PRKAR2A | Osteoporosis, Postmenopausal | 16.1 |
| DRAM1 | Osteoporosis, Postmenopausal | 16.09 |
| MTA1 | Osteoporosis, Postmenopausal | 16.09 |
| RUNX1T1 | Osteoporosis, Postmenopausal | 16.09 |
| SOCS3 | Osteoporosis, Postmenopausal | 16.09 |
| EIF3B | Osteoporosis, Postmenopausal | 16.08 |
| KIF4A | Osteoporosis, Postmenopausal | 16.08 |
| SLC23A2 | Osteoporosis, Postmenopausal | 16.08 |
| CDKN2D | Osteoporosis, Postmenopausal | 16.07 |
| CENPW | Osteoporosis, Postmenopausal | 16.07 |
| ERCC6L | Osteoporosis, Postmenopausal | 16.07 |
| GNAI3 | Osteoporosis, Postmenopausal | 16.07 |
| GPX1 | Osteoporosis, Postmenopausal | 16.07 |
| OIP5 | Osteoporosis, Postmenopausal | 16.07 |
| RABGAP1L | Osteoporosis, Postmenopausal | 16.07 |
| C3 | Osteoporosis, Postmenopausal | 16.06 |
| PRKCQ | Osteoporosis, Postmenopausal | 16.06 |
| LMNB2 | Osteoporosis, Postmenopausal | 16.05 |
| MMP8 | Osteoporosis, Postmenopausal | 16.05 |
| CTSB | Osteoporosis, Postmenopausal | 16.04 |
| ERBB2 | Osteoporosis, Postmenopausal | 16.04 |
| GSK3B | Osteoporosis, Postmenopausal | 16.04 |
| SERPINB5 | Osteoporosis, Postmenopausal | 16.04 |
| CYP2R1 | Osteoporosis, Postmenopausal | 16.03 |
| H1-2 | Osteoporosis, Postmenopausal | 16.03 |
| WDHD1 | Osteoporosis, Postmenopausal | 16.03 |
| CSTA | Osteoporosis, Postmenopausal | 16.02 |
| RHOBTB1 | Osteoporosis, Postmenopausal | 16.02 |
| SGK1 | Osteoporosis, Postmenopausal | 16.02 |
| TFF3 | Osteoporosis, Postmenopausal | 16.02 |
| THRA | Osteoporosis, Postmenopausal | 16.02 |
| DCT | Osteoporosis, Postmenopausal | 16.01 |
| RAI14 | Osteoporosis, Postmenopausal | 16.01 |
| SSR1 | Osteoporosis, Postmenopausal | 16.01 |
| SYNJ2 | Osteoporosis, Postmenopausal | 16.01 |
| ACADSB | Osteoporosis, Postmenopausal | 16 |
| IL1A | Osteoporosis, Postmenopausal | 16 |
| NSD2 | Osteoporosis, Postmenopausal | 16 |
| ELOVL3 | Osteoporosis, Postmenopausal | 15.99 |
| P2RY2 | Osteoporosis, Postmenopausal | 15.99 |
| TRPM7 | Osteoporosis, Postmenopausal | 15.99 |
| BBC3 | Osteoporosis, Postmenopausal | 15.98 |
| DYSF | Osteoporosis, Postmenopausal | 15.98 |
| MET | Osteoporosis, Postmenopausal | 15.98 |
| YBX3 | Osteoporosis, Postmenopausal | 15.98 |
| CDC7 | Osteoporosis, Postmenopausal | 15.95 |
| EPOR | Osteoporosis, Postmenopausal | 15.95 |
| FBXO30 | Osteoporosis, Postmenopausal | 15.95 |
| SCD2 | Osteoporosis, Postmenopausal | 15.95 |
| SLC10A2 | Osteoporosis, Postmenopausal | 15.95 |
| SNCA | Osteoporosis, Postmenopausal | 15.95 |
| GAS7 | Osteoporosis, Postmenopausal | 15.94 |
| HSD17B11 | Osteoporosis, Postmenopausal | 15.94 |
| INSIG1 | Osteoporosis, Postmenopausal | 15.94 |
| PPFIBP1 | Osteoporosis, Postmenopausal | 15.94 |
| VSNL1 | Osteoporosis, Postmenopausal | 15.94 |
| FIGNL1 | Osteoporosis, Postmenopausal | 15.93 |
| NUDT7 | Osteoporosis, Postmenopausal | 15.93 |
| EIF2S1 | Osteoporosis, Postmenopausal | 15.92 |
| HAO2 | Osteoporosis, Postmenopausal | 15.92 |
| MIR33A | Osteoporosis, Postmenopausal | 15.92 |
| MIR663A | Osteoporosis, Postmenopausal | 15.92 |
| PPT1 | Osteoporosis, Postmenopausal | 15.92 |
| GINS1 | Osteoporosis, Postmenopausal | 15.9 |
| MYO5A | Osteoporosis, Postmenopausal | 15.9 |
| SULT1C2 | Osteoporosis, Postmenopausal | 15.9 |
| TTN | Osteoporosis, Postmenopausal | 15.9 |
| CAMKK2 | Osteoporosis, Postmenopausal | 15.89 |
| CP | Osteoporosis, Postmenopausal | 15.89 |
| H2AX | Osteoporosis, Postmenopausal | 15.89 |
| PCSK5 | Osteoporosis, Postmenopausal | 15.89 |
| ACE | Osteoporosis, Postmenopausal | 15.87 |
| CA12 | Osteoporosis, Postmenopausal | 15.87 |
| CXADR | Osteoporosis, Postmenopausal | 15.87 |
| SESN3 | Osteoporosis, Postmenopausal | 15.87 |
| HIBCH | Osteoporosis, Postmenopausal | 15.86 |
| LIN7A | Osteoporosis, Postmenopausal | 15.86 |
| PAFAH1B1 | Osteoporosis, Postmenopausal | 15.86 |
| BIRC3 | Osteoporosis, Postmenopausal | 15.85 |
| CD82 | Osteoporosis, Postmenopausal | 15.84 |
| DEPDC1B | Osteoporosis, Postmenopausal | 15.84 |
| RGS1 | Osteoporosis, Postmenopausal | 15.84 |
| DGKA | Osteoporosis, Postmenopausal | 15.83 |
| NID2 | Osteoporosis, Postmenopausal | 15.83 |
| TNNC1 | Osteoporosis, Postmenopausal | 15.83 |
| TYROBP | Osteoporosis, Postmenopausal | 15.83 |
| DHX9 | Osteoporosis, Postmenopausal | 15.82 |
| HIVEP2 | Osteoporosis, Postmenopausal | 15.81 |
| SIPA1L2 | Osteoporosis, Postmenopausal | 15.81 |
| DBF4 | Osteoporosis, Postmenopausal | 15.8 |
| DTYMK | Osteoporosis, Postmenopausal | 15.8 |
| NCAPG2 | Osteoporosis, Postmenopausal | 15.8 |
| EPB41L1 | Osteoporosis, Postmenopausal | 15.79 |
| MT2A | Osteoporosis, Postmenopausal | 15.79 |
| NEB | Osteoporosis, Postmenopausal | 15.79 |
| STK17B | Osteoporosis, Postmenopausal | 15.79 |
| TCF7 | Osteoporosis, Postmenopausal | 15.79 |
| NPY1R | Osteoporosis, Postmenopausal | 15.78 |
| USP7 | Osteoporosis, Postmenopausal | 15.78 |
| RAMP1 | Osteoporosis, Postmenopausal | 15.77 |
| FCER1G | Osteoporosis, Postmenopausal | 15.76 |
| HNF1A | Osteoporosis, Postmenopausal | 15.76 |
| KIF18A | Osteoporosis, Postmenopausal | 15.76 |
| LYZ | Osteoporosis, Postmenopausal | 15.76 |
| COX5B | Osteoporosis, Postmenopausal | 15.75 |
| NBN | Osteoporosis, Postmenopausal | 15.75 |
| SYNPO | Osteoporosis, Postmenopausal | 15.75 |
| CALB1 | Osteoporosis, Postmenopausal | 15.74 |
| UCP2 | Osteoporosis, Postmenopausal | 15.74 |
| ATP5MC1 | Osteoporosis, Postmenopausal | 15.72 |
| H2AC6 | Osteoporosis, Postmenopausal | 15.72 |
| OLFM1 | Osteoporosis, Postmenopausal | 15.72 |
| SLCO4A1 | Osteoporosis, Postmenopausal | 15.72 |
| BCL2L11 | Osteoporosis, Postmenopausal | 15.71 |
| MED1 | Osteoporosis, Postmenopausal | 15.71 |
| ALDH4A1 | Osteoporosis, Postmenopausal | 15.7 |
| APOM | Osteoporosis, Postmenopausal | 15.7 |
| LCN2 | Osteoporosis, Postmenopausal | 15.7 |
| TBXA2R | Osteoporosis, Postmenopausal | 15.7 |
| AMIGO2 | Osteoporosis, Postmenopausal | 15.69 |
| DFFA | Osteoporosis, Postmenopausal | 15.69 |
| GAA | Osteoporosis, Postmenopausal | 15.69 |
| GOT1 | Osteoporosis, Postmenopausal | 15.69 |
| GZMB | Osteoporosis, Postmenopausal | 15.69 |
| CFI | Osteoporosis, Postmenopausal | 15.68 |
| NINJ1 | Osteoporosis, Postmenopausal | 15.68 |
| WFDC2 | Osteoporosis, Postmenopausal | 15.67 |
| TPTE | Osteoporosis, Postmenopausal | 15.66 |
| METRNL | Osteoporosis, Postmenopausal | 15.64 |
| WSB1 | Osteoporosis, Postmenopausal | 15.64 |
| MKNK2 | Osteoporosis, Postmenopausal | 15.63 |
| TPR | Osteoporosis, Postmenopausal | 15.63 |
| GPC1 | Osteoporosis, Postmenopausal | 15.62 |
| RTL10 | Osteoporosis, Postmenopausal | 15.62 |
| SLC5A3 | Osteoporosis, Postmenopausal | 15.62 |
| RARRES1 | Osteoporosis, Postmenopausal | 15.61 |
| DHRS7 | Osteoporosis, Postmenopausal | 15.6 |
| DSC2 | Osteoporosis, Postmenopausal | 15.6 |
| KPNB1 | Osteoporosis, Postmenopausal | 15.6 |
| NPPA | Osteoporosis, Postmenopausal | 15.6 |
| ORC1 | Osteoporosis, Postmenopausal | 15.6 |
| PAPPA | Osteoporosis, Postmenopausal | 15.6 |
| SLC51A | Osteoporosis, Postmenopausal | 15.6 |
| AHSG | Osteoporosis, Postmenopausal | 15.59 |
| CASP10 | Osteoporosis, Postmenopausal | 15.59 |
| HPCAL1 | Osteoporosis, Postmenopausal | 15.59 |
| JUNB | Osteoporosis, Postmenopausal | 15.59 |
| POTEE | Osteoporosis, Postmenopausal | 15.59 |
| SOX17 | Osteoporosis, Postmenopausal | 15.59 |
| TSHR | Osteoporosis, Postmenopausal | 15.59 |
| CDCA7 | Osteoporosis, Postmenopausal | 15.58 |
| IFI6 | Osteoporosis, Postmenopausal | 15.57 |
| SLCO1A2 | Osteoporosis, Postmenopausal | 15.57 |
| ACOX2 | Osteoporosis, Postmenopausal | 15.56 |
| CTSD | Osteoporosis, Postmenopausal | 15.56 |
| DRD2 | Osteoporosis, Postmenopausal | 15.56 |
| EEF1A2 | Osteoporosis, Postmenopausal | 15.56 |
| PFKFB4 | Osteoporosis, Postmenopausal | 15.55 |
| PLAU | Osteoporosis, Postmenopausal | 15.55 |
| SPSB1 | Osteoporosis, Postmenopausal | 15.55 |
| CLU | Osteoporosis, Postmenopausal | 15.54 |
| PIK3CB | Osteoporosis, Postmenopausal | 15.54 |
| APOA1 | Osteoporosis, Postmenopausal | 15.53 |
| CACNA1G | Osteoporosis, Postmenopausal | 15.53 |
| KIF1B | Osteoporosis, Postmenopausal | 15.53 |
| SMO | Osteoporosis, Postmenopausal | 15.53 |
| LRP2 | Osteoporosis, Postmenopausal | 15.52 |
| ODC1 | Osteoporosis, Postmenopausal | 15.52 |
| TNFRSF10B | Osteoporosis, Postmenopausal | 15.52 |
| LPP | Osteoporosis, Postmenopausal | 15.51 |
| EPS8 | Osteoporosis, Postmenopausal | 15.5 |
| TFDP1 | Osteoporosis, Postmenopausal | 15.5 |
| BLM | Osteoporosis, Postmenopausal | 15.48 |
| LTBP2 | Osteoporosis, Postmenopausal | 15.48 |
| PDHA1 | Osteoporosis, Postmenopausal | 15.48 |
| PIK3R1 | Osteoporosis, Postmenopausal | 15.48 |
| SPINK1 | Osteoporosis, Postmenopausal | 15.48 |
| HIF1A | Osteoporosis, Postmenopausal | 15.47 |
| HPSE | Osteoporosis, Postmenopausal | 15.47 |
| PSME1 | Osteoporosis, Postmenopausal | 15.47 |
| COL8A1 | Osteoporosis, Postmenopausal | 15.45 |
| MELK | Osteoporosis, Postmenopausal | 15.45 |
| PSAP | Osteoporosis, Postmenopausal | 15.45 |
| CRYZ | Osteoporosis, Postmenopausal | 15.44 |
| GPRC5A | Osteoporosis, Postmenopausal | 15.44 |
| KAT2B | Osteoporosis, Postmenopausal | 15.44 |
| FAM83D | Osteoporosis, Postmenopausal | 15.43 |
| LRG1 | Osteoporosis, Postmenopausal | 15.43 |
| QSOX1 | Osteoporosis, Postmenopausal | 15.43 |
| CD40LG | Osteoporosis, Postmenopausal | 15.42 |
| CPEB2 | Osteoporosis, Postmenopausal | 15.42 |
| FGF9 | Osteoporosis, Postmenopausal | 15.42 |
| PLSCR1 | Osteoporosis, Postmenopausal | 15.42 |
| STEAP4 | Osteoporosis, Postmenopausal | 15.42 |
| KNSTRN | Osteoporosis, Postmenopausal | 15.39 |
| SLC51B | Osteoporosis, Postmenopausal | 15.39 |
| TNFAIP8 | Osteoporosis, Postmenopausal | 15.38 |
| CXCL12 | Osteoporosis, Postmenopausal | 15.37 |
| PPP1R3B | Osteoporosis, Postmenopausal | 15.37 |
| EGLN1 | Osteoporosis, Postmenopausal | 15.36 |
| MTUS1 | Osteoporosis, Postmenopausal | 15.36 |
| IQGAP1 | Osteoporosis, Postmenopausal | 15.35 |
| CDKN2A | Osteoporosis, Postmenopausal | 15.34 |
| CEP55 | Osteoporosis, Postmenopausal | 15.33 |
| LRP5L | Osteoporosis, Postmenopausal | 15.33 |
| TRAF1 | Osteoporosis, Postmenopausal | 15.33 |
| PDZK1 | Osteoporosis, Postmenopausal | 15.32 |
| ARPC1B | Osteoporosis, Postmenopausal | 15.31 |
| CYP3A7 | Osteoporosis, Postmenopausal | 15.31 |
| NR2F1 | Osteoporosis, Postmenopausal | 15.31 |
| MIR424 | Osteoporosis, Postmenopausal | 15.3 |
| POLA1 | Osteoporosis, Postmenopausal | 15.3 |
| RBM13 | Osteoporosis, Postmenopausal | 15.3 |
| ACSM3 | Osteoporosis, Postmenopausal | 15.29 |
| ANP32A | Osteoporosis, Postmenopausal | 15.29 |
| DYNLL1 | Osteoporosis, Postmenopausal | 15.29 |
| IQGAP2 | Osteoporosis, Postmenopausal | 15.29 |
| TNFRSF25 | Osteoporosis, Postmenopausal | 15.29 |
| DEK | Osteoporosis, Postmenopausal | 15.28 |
| GC | Osteoporosis, Postmenopausal | 15.28 |
| KNL1 | Osteoporosis, Postmenopausal | 15.28 |
| NCAPD2 | Osteoporosis, Postmenopausal | 15.28 |
| OPTN | Osteoporosis, Postmenopausal | 15.28 |
| POLE2 | Osteoporosis, Postmenopausal | 15.28 |
| RSRP1 | Osteoporosis, Postmenopausal | 15.28 |
| SLC9A1 | Osteoporosis, Postmenopausal | 15.28 |
| VAV3 | Osteoporosis, Postmenopausal | 15.28 |
| IMPDH2 | Osteoporosis, Postmenopausal | 15.27 |
| XIAP | Osteoporosis, Postmenopausal | 15.27 |
| FSTL1 | Osteoporosis, Postmenopausal | 15.26 |
| RAB31 | Osteoporosis, Postmenopausal | 15.26 |
| SRSF1 | Osteoporosis, Postmenopausal | 15.26 |
| CTNNA1 | Osteoporosis, Postmenopausal | 15.25 |
| ITGA3 | Osteoporosis, Postmenopausal | 15.25 |
| NPHS1 | Osteoporosis, Postmenopausal | 15.25 |
| THRB | Osteoporosis, Postmenopausal | 15.25 |
| CPS1 | Osteoporosis, Postmenopausal | 15.24 |
| DEPTOR | Osteoporosis, Postmenopausal | 15.24 |
| GDA | Osteoporosis, Postmenopausal | 15.24 |
| LAMC1 | Osteoporosis, Postmenopausal | 15.24 |
| NIBAN1 | Osteoporosis, Postmenopausal | 15.24 |
| PGRMC1 | Osteoporosis, Postmenopausal | 15.24 |
| PPM1D | Osteoporosis, Postmenopausal | 15.24 |
| HMGCS1 | Osteoporosis, Postmenopausal | 15.23 |
| PIK3CD | Osteoporosis, Postmenopausal | 15.23 |
| CKS1B | Osteoporosis, Postmenopausal | 15.22 |
| PRIM1 | Osteoporosis, Postmenopausal | 15.22 |
| CACYBP | Osteoporosis, Postmenopausal | 15.21 |
| CNN1 | Osteoporosis, Postmenopausal | 15.21 |
| CA9 | Osteoporosis, Postmenopausal | 15.2 |
| ABLIM1 | Osteoporosis, Postmenopausal | 15.19 |
| CRELD2 | Osteoporosis, Postmenopausal | 15.19 |
| HSPA5 | Osteoporosis, Postmenopausal | 15.19 |
| PLXNA2 | Osteoporosis, Postmenopausal | 15.19 |
| PSMB10 | Osteoporosis, Postmenopausal | 15.19 |
| RFC4 | Osteoporosis, Postmenopausal | 15.19 |
| TRADD | Osteoporosis, Postmenopausal | 15.19 |
| MAPT | Osteoporosis, Postmenopausal | 15.18 |
| APOD | Osteoporosis, Postmenopausal | 15.16 |
| TCF19 | Osteoporosis, Postmenopausal | 15.16 |
| CYP2B7P | Osteoporosis, Postmenopausal | 15.15 |
| THBS2 | Osteoporosis, Postmenopausal | 15.15 |
| GINS2 | Osteoporosis, Postmenopausal | 15.14 |
| NASP | Osteoporosis, Postmenopausal | 15.14 |
| ALOX5AP | Osteoporosis, Postmenopausal | 15.13 |
| CCL13 | Osteoporosis, Postmenopausal | 15.13 |
| RPS6KA1 | Osteoporosis, Postmenopausal | 15.13 |
| SLC5A1 | Osteoporosis, Postmenopausal | 15.13 |
| STK11 | Osteoporosis, Postmenopausal | 15.13 |
| BCAR3 | Osteoporosis, Postmenopausal | 15.12 |
| BGN | Osteoporosis, Postmenopausal | 15.12 |
| EIF4A2 | Osteoporosis, Postmenopausal | 15.12 |
| IL13RA1 | Osteoporosis, Postmenopausal | 15.12 |
| SLCO2A1 | Osteoporosis, Postmenopausal | 15.12 |
| TUBB | Osteoporosis, Postmenopausal | 15.12 |
| PROC | Osteoporosis, Postmenopausal | 15.11 |
| UBE2T | Osteoporosis, Postmenopausal | 15.11 |
| ISX | Osteoporosis, Postmenopausal | 15.1 |
| ISYNA1 | Osteoporosis, Postmenopausal | 15.1 |
| NR5A1 | Osteoporosis, Postmenopausal | 15.1 |
| TPSG1 | Osteoporosis, Postmenopausal | 15.09 |
| SOX30 | Osteoporosis, Postmenopausal | 15.08 |
| EIF2AK3 | Osteoporosis, Postmenopausal | 15.07 |
| F7 | Osteoporosis, Postmenopausal | 15.07 |
| NF1 | Osteoporosis, Postmenopausal | 15.07 |
| SERHL | Osteoporosis, Postmenopausal | 15.07 |
| SMARCA4 | Osteoporosis, Postmenopausal | 15.07 |
| TG | Osteoporosis, Postmenopausal | 15.07 |
| CLIC4 | Osteoporosis, Postmenopausal | 15.06 |
| CYP7A1 | Osteoporosis, Postmenopausal | 15.06 |
| FGF2 | Osteoporosis, Postmenopausal | 15.05 |
| SERPINC1 | Osteoporosis, Postmenopausal | 15.05 |
| IL1RAP | Osteoporosis, Postmenopausal | 15.04 |
| NR2F2 | Osteoporosis, Postmenopausal | 15.04 |
| SELL | Osteoporosis, Postmenopausal | 15.04 |
| YWHAE | Osteoporosis, Postmenopausal | 15.04 |
| ASF1B | Osteoporosis, Postmenopausal | 15.03 |
| ESPL1 | Osteoporosis, Postmenopausal | 15.03 |
| SEMA3C | Osteoporosis, Postmenopausal | 15.03 |
| CHORDC1 | Osteoporosis, Postmenopausal | 15.02 |
| COL6A3 | Osteoporosis, Postmenopausal | 15.02 |
| PLEC | Osteoporosis, Postmenopausal | 15.02 |
| TGM1 | Osteoporosis, Postmenopausal | 15.02 |
| CDCA2 | Osteoporosis, Postmenopausal | 15.01 |
| HAS2 | Osteoporosis, Postmenopausal | 15.01 |
| MBNL2 | Osteoporosis, Postmenopausal | 15.01 |
| MYLIP | Osteoporosis, Postmenopausal | 15.01 |
| KLRC3 | Osteoporosis, Postmenopausal | 15 |
| PON2 | Osteoporosis, Postmenopausal | 15 |
| SLC7A2 | Osteoporosis, Postmenopausal | 15 |
| TALDO1 | Osteoporosis, Postmenopausal | 15 |
| ELK1 | Osteoporosis, Postmenopausal | 14.99 |
| GLI1 | Osteoporosis, Postmenopausal | 14.99 |
| HNRNPD | Osteoporosis, Postmenopausal | 14.98 |
| SH2D4B | Osteoporosis, Postmenopausal | 14.98 |
| TNFSF9 | Osteoporosis, Postmenopausal | 14.98 |
| LYPD5 | Osteoporosis, Postmenopausal | 14.97 |
| TNFRSF19 | Osteoporosis, Postmenopausal | 14.96 |
| FLNB | Osteoporosis, Postmenopausal | 14.95 |
| ELOVL5 | Osteoporosis, Postmenopausal | 14.94 |
| KIAA0753 | Osteoporosis, Postmenopausal | 14.94 |
| PPP2CA | Osteoporosis, Postmenopausal | 14.94 |
| TCF7L2 | Osteoporosis, Postmenopausal | 14.94 |
| NCAPH | Osteoporosis, Postmenopausal | 14.92 |
| RBL2 | Osteoporosis, Postmenopausal | 14.92 |
| TWIST1 | Osteoporosis, Postmenopausal | 14.92 |
| CTPS1 | Osteoporosis, Postmenopausal | 14.91 |
| PDE4D | Osteoporosis, Postmenopausal | 14.91 |
| PPP1R12A | Osteoporosis, Postmenopausal | 14.91 |
| ZNF488 | Osteoporosis, Postmenopausal | 14.91 |
| ARHGDIB | Osteoporosis, Postmenopausal | 14.9 |
| HSPB1 | Osteoporosis, Postmenopausal | 14.9 |
| GALR3 | Osteoporosis, Postmenopausal | 14.89 |
| IER2 | Osteoporosis, Postmenopausal | 14.89 |
| SLC39A8 | Osteoporosis, Postmenopausal | 14.89 |
| ASAH1 | Osteoporosis, Postmenopausal | 14.88 |
| COTL1 | Osteoporosis, Postmenopausal | 14.88 |
| SMOX | Osteoporosis, Postmenopausal | 14.88 |
| CAPG | Osteoporosis, Postmenopausal | 14.87 |
| IRAK2 | Osteoporosis, Postmenopausal | 14.87 |
| NHERF1 | Osteoporosis, Postmenopausal | 14.87 |
| SHH | Osteoporosis, Postmenopausal | 14.86 |
| C2CD4A | Osteoporosis, Postmenopausal | 14.85 |
| SLC31A1 | Osteoporosis, Postmenopausal | 14.85 |
| CYB5R3 | Osteoporosis, Postmenopausal | 14.83 |
| EIF2AK2 | Osteoporosis, Postmenopausal | 14.83 |
| GJA1 | Osteoporosis, Postmenopausal | 14.83 |
| LIPE | Osteoporosis, Postmenopausal | 14.83 |
| PPARGC1B | Osteoporosis, Postmenopausal | 14.83 |
| RRM2B | Osteoporosis, Postmenopausal | 14.83 |
| C4ORF46 | Osteoporosis, Postmenopausal | 14.82 |
| CAMK2D | Osteoporosis, Postmenopausal | 14.82 |
| G6PD | Osteoporosis, Postmenopausal | 14.81 |
| GSTK1 | Osteoporosis, Postmenopausal | 14.81 |
| MAP3K8 | Osteoporosis, Postmenopausal | 14.81 |
| FHL1 | Osteoporosis, Postmenopausal | 14.8 |
| GFAP | Osteoporosis, Postmenopausal | 14.8 |
| ZNF91 | Osteoporosis, Postmenopausal | 14.8 |
| FMO5 | Osteoporosis, Postmenopausal | 14.79 |
| LAMB3 | Osteoporosis, Postmenopausal | 14.79 |
| RAD50 | Osteoporosis, Postmenopausal | 14.79 |
| VPS35L | Osteoporosis, Postmenopausal | 14.79 |
| INHBB | Osteoporosis, Postmenopausal | 14.78 |
| PROKR1 | Osteoporosis, Postmenopausal | 14.78 |
| NUF2 | Osteoporosis, Postmenopausal | 14.77 |
| BSG | Osteoporosis, Postmenopausal | 14.76 |
| CSRP2 | Osteoporosis, Postmenopausal | 14.76 |
| ID4 | Osteoporosis, Postmenopausal | 14.76 |
| KRT17 | Osteoporosis, Postmenopausal | 14.76 |
| P4HA1 | Osteoporosis, Postmenopausal | 14.76 |
| DMD | Osteoporosis, Postmenopausal | 14.75 |
| POMC | Osteoporosis, Postmenopausal | 14.75 |
| PTGDS | Osteoporosis, Postmenopausal | 14.75 |
| PMEPA1 | Osteoporosis, Postmenopausal | 14.74 |
| CTSH | Osteoporosis, Postmenopausal | 14.73 |
| CYP2C19 | Osteoporosis, Postmenopausal | 14.73 |
| DEPP1 | Osteoporosis, Postmenopausal | 14.73 |
| H2BC17 | Osteoporosis, Postmenopausal | 14.73 |
| KCNK15 | Osteoporosis, Postmenopausal | 14.73 |
| NFAT5 | Osteoporosis, Postmenopausal | 14.73 |
| SHC1 | Osteoporosis, Postmenopausal | 14.73 |
| PTHLH | Osteoporosis, Postmenopausal | 14.72 |
| ARRDC3 | Osteoporosis, Postmenopausal | 14.71 |
| ENPP1 | Osteoporosis, Postmenopausal | 14.71 |
| HMGA2 | Osteoporosis, Postmenopausal | 14.71 |
| LHCGR | Osteoporosis, Postmenopausal | 14.71 |
| MAF | Osteoporosis, Postmenopausal | 14.71 |
| AMPD3 | Osteoporosis, Postmenopausal | 14.7 |
| KCNE5 | Osteoporosis, Postmenopausal | 14.7 |
| TAGLN2 | Osteoporosis, Postmenopausal | 14.7 |
| UPK2 | Osteoporosis, Postmenopausal | 14.7 |
| SFRP1 | Osteoporosis, Postmenopausal | 14.69 |
| ZWINT | Osteoporosis, Postmenopausal | 14.69 |
| COL12A1 | Osteoporosis, Postmenopausal | 14.68 |
| ABCD3 | Osteoporosis, Postmenopausal | 14.67 |
| C1QB | Osteoporosis, Postmenopausal | 14.67 |
| MDM2 | Osteoporosis, Postmenopausal | 14.67 |
| NQO2 | Osteoporosis, Postmenopausal | 14.67 |
| DST | Osteoporosis, Postmenopausal | 14.66 |
| NOTCH2 | Osteoporosis, Postmenopausal | 14.66 |
| ABCG8 | Osteoporosis, Postmenopausal | 14.65 |
| CYP2D6 | Osteoporosis, Postmenopausal | 14.64 |
| CYP4B1 | Osteoporosis, Postmenopausal | 14.64 |
| EBP | Osteoporosis, Postmenopausal | 14.64 |
| F2RL1 | Osteoporosis, Postmenopausal | 14.64 |
| MGST3 | Osteoporosis, Postmenopausal | 14.64 |
| RND1 | Osteoporosis, Postmenopausal | 14.64 |
| TMEM200B | Osteoporosis, Postmenopausal | 14.64 |
| ZNF469 | Osteoporosis, Postmenopausal | 14.64 |
| BDH1 | Osteoporosis, Postmenopausal | 14.63 |
| BDKRB2 | Osteoporosis, Postmenopausal | 14.63 |
| IRAK1 | Osteoporosis, Postmenopausal | 14.63 |
| RGCC | Osteoporosis, Postmenopausal | 14.63 |
| ANLN | Osteoporosis, Postmenopausal | 14.62 |
| C5 | Osteoporosis, Postmenopausal | 14.62 |
| IFITM1 | Osteoporosis, Postmenopausal | 14.62 |
| NPW | Osteoporosis, Postmenopausal | 14.61 |
| EFEMP1 | Osteoporosis, Postmenopausal | 14.59 |
| FKBP4 | Osteoporosis, Postmenopausal | 14.59 |
| GLUD1 | Osteoporosis, Postmenopausal | 14.59 |
| MIR101A | Osteoporosis, Postmenopausal | 14.59 |
| PNPLA2 | Osteoporosis, Postmenopausal | 14.59 |
| POU2AF3 | Osteoporosis, Postmenopausal | 14.59 |
| GAL | Osteoporosis, Postmenopausal | 14.58 |
| MYO1B | Osteoporosis, Postmenopausal | 14.58 |
| ACSL3 | Osteoporosis, Postmenopausal | 14.57 |
| BCHE | Osteoporosis, Postmenopausal | 14.57 |
| CALD1 | Osteoporosis, Postmenopausal | 14.57 |
| COL6A1 | Osteoporosis, Postmenopausal | 14.56 |
| F10 | Osteoporosis, Postmenopausal | 14.56 |
| PGF | Osteoporosis, Postmenopausal | 14.56 |
| PHLDA2 | Osteoporosis, Postmenopausal | 14.56 |
| PLOD2 | Osteoporosis, Postmenopausal | 14.56 |
| SLC5A5 | Osteoporosis, Postmenopausal | 14.56 |
| DHRS3 | Osteoporosis, Postmenopausal | 14.55 |
| DMBX1 | Osteoporosis, Postmenopausal | 14.55 |
| PPL | Osteoporosis, Postmenopausal | 14.55 |
| SPC25 | Osteoporosis, Postmenopausal | 14.55 |
| ETS2 | Osteoporosis, Postmenopausal | 14.54 |
| SIGMAR1 | Osteoporosis, Postmenopausal | 14.54 |
| WT1 | Osteoporosis, Postmenopausal | 14.54 |
| DUOXA2 | Osteoporosis, Postmenopausal | 14.53 |
| CHRM1 | Osteoporosis, Postmenopausal | 14.51 |
| PRKCZ | Osteoporosis, Postmenopausal | 14.51 |
| USP2 | Osteoporosis, Postmenopausal | 14.51 |
| KLF8 | Osteoporosis, Postmenopausal | 14.5 |
| ZNF114 | Osteoporosis, Postmenopausal | 14.5 |
| CIDEC | Osteoporosis, Postmenopausal | 14.49 |
| HTR5A | Osteoporosis, Postmenopausal | 14.49 |
| ITGAV | Osteoporosis, Postmenopausal | 14.49 |
| AGTR1A | Osteoporosis, Postmenopausal | 14.48 |
| CCNF | Osteoporosis, Postmenopausal | 14.48 |
| CEACAM5 | Osteoporosis, Postmenopausal | 14.48 |
| LUM | Osteoporosis, Postmenopausal | 14.48 |
| SERPINF1 | Osteoporosis, Postmenopausal | 14.48 |
| CPT1A | Osteoporosis, Postmenopausal | 14.46 |
| P2RX7 | Osteoporosis, Postmenopausal | 14.46 |
| LBP | Osteoporosis, Postmenopausal | 14.45 |
| SLC25A20 | Osteoporosis, Postmenopausal | 14.45 |
| LDOC1 | Osteoporosis, Postmenopausal | 14.44 |
| MIR21 | Osteoporosis, Postmenopausal | 14.44 |
| NLRP3 | Osteoporosis, Postmenopausal | 14.44 |
| PLCG1 | Osteoporosis, Postmenopausal | 14.44 |
| PNPLA5 | Osteoporosis, Postmenopausal | 14.44 |
| YWHAZ | Osteoporosis, Postmenopausal | 14.44 |
| ALCAM | Osteoporosis, Postmenopausal | 14.43 |
| GHRHR | Osteoporosis, Postmenopausal | 14.43 |
| MIR362 | Osteoporosis, Postmenopausal | 14.43 |
| NEDD4L | Osteoporosis, Postmenopausal | 14.42 |
| RGS4 | Osteoporosis, Postmenopausal | 14.42 |
| DUSP4 | Osteoporosis, Postmenopausal | 14.41 |
| RBP1 | Osteoporosis, Postmenopausal | 14.41 |
| STAT5A | Osteoporosis, Postmenopausal | 14.41 |
| BNIP3L | Osteoporosis, Postmenopausal | 14.4 |
| PHLDA3 | Osteoporosis, Postmenopausal | 14.4 |
| BID | Osteoporosis, Postmenopausal | 14.38 |
| PMP22 | Osteoporosis, Postmenopausal | 14.38 |
| PPP3CA | Osteoporosis, Postmenopausal | 14.38 |
| CD36 | Osteoporosis, Postmenopausal | 14.37 |
| TMEM121B | Osteoporosis, Postmenopausal | 14.37 |
| UPP1 | Osteoporosis, Postmenopausal | 14.37 |
| ASTE1 | Osteoporosis, Postmenopausal | 14.36 |
| C14ORF132 | Osteoporosis, Postmenopausal | 14.36 |
| HNRNPA1 | Osteoporosis, Postmenopausal | 14.36 |
| UBC | Osteoporosis, Postmenopausal | 14.36 |
| ZNF84 | Osteoporosis, Postmenopausal | 14.36 |
| GPD1 | Osteoporosis, Postmenopausal | 14.35 |
| GRIA1 | Osteoporosis, Postmenopausal | 14.35 |
| CXCL14 | Osteoporosis, Postmenopausal | 14.34 |
| GNMT | Osteoporosis, Postmenopausal | 14.33 |
| MAD2L1 | Osteoporosis, Postmenopausal | 14.32 |
| ALOX15B | Osteoporosis, Postmenopausal | 14.31 |
| CXCL3 | Osteoporosis, Postmenopausal | 14.31 |
| IL6R | Osteoporosis, Postmenopausal | 14.31 |
| MSH2 | Osteoporosis, Postmenopausal | 14.31 |
| VNN1 | Osteoporosis, Postmenopausal | 14.31 |
| ULK1 | Osteoporosis, Postmenopausal | 14.29 |
| DENND11 | Osteoporosis, Postmenopausal | 14.28 |
| PAX4 | Osteoporosis, Postmenopausal | 14.28 |
| ZNF580 | Osteoporosis, Postmenopausal | 14.28 |
| ITGA6 | Osteoporosis, Postmenopausal | 14.27 |
| PRAP1 | Osteoporosis, Postmenopausal | 14.26 |
| HRH1 | Osteoporosis, Postmenopausal | 14.24 |
| KLRC1 | Osteoporosis, Postmenopausal | 14.24 |
| PCSK9 | Osteoporosis, Postmenopausal | 14.24 |
| SLC38A11 | Osteoporosis, Postmenopausal | 14.24 |
| FEN1 | Osteoporosis, Postmenopausal | 14.23 |
| LIPG | Osteoporosis, Postmenopausal | 14.23 |
| S100A7 | Osteoporosis, Postmenopausal | 14.23 |
| AVP | Osteoporosis, Postmenopausal | 14.22 |
| INHBE | Osteoporosis, Postmenopausal | 14.22 |
| PYGL | Osteoporosis, Postmenopausal | 14.22 |
| NFKBIB | Osteoporosis, Postmenopausal | 14.21 |
| S100B | Osteoporosis, Postmenopausal | 14.21 |
| C1ORF115 | Osteoporosis, Postmenopausal | 14.2 |
| GLTPD2 | Osteoporosis, Postmenopausal | 14.2 |
| IL8R | Osteoporosis, Postmenopausal | 14.2 |
| SLC29A1 | Osteoporosis, Postmenopausal | 14.2 |
| STAT1-2 | Osteoporosis, Postmenopausal | 14.2 |
| TPX2 | Osteoporosis, Postmenopausal | 14.2 |
| TRAV17 | Osteoporosis, Postmenopausal | 14.2 |
| TRAV21 | Osteoporosis, Postmenopausal | 14.2 |
| TRAV23DV6 | Osteoporosis, Postmenopausal | 14.2 |
| DKK1 | Osteoporosis, Postmenopausal | 14.19 |
| PLCB1 | Osteoporosis, Postmenopausal | 14.18 |
| SPTBN1 | Osteoporosis, Postmenopausal | 14.18 |
| BCL3 | Osteoporosis, Postmenopausal | 14.16 |
| GBP2 | Osteoporosis, Postmenopausal | 14.16 |
| SHBG | Osteoporosis, Postmenopausal | 14.15 |
| EMP1 | Osteoporosis, Postmenopausal | 14.14 |
| ANK | Osteoporosis, Postmenopausal | 14.13 |
| KCNJ14 | Osteoporosis, Postmenopausal | 14.13 |
| FBP1 | Osteoporosis, Postmenopausal | 14.12 |
| ZEB1 | Osteoporosis, Postmenopausal | 14.12 |
| CD9 | Osteoporosis, Postmenopausal | 14.11 |
| RORC | Osteoporosis, Postmenopausal | 14.11 |
| SLC16A3 | Osteoporosis, Postmenopausal | 14.11 |
| NFATC1 | Osteoporosis, Postmenopausal | 14.1 |
| NUSAP1 | Osteoporosis, Postmenopausal | 14.1 |
| RASD1 | Osteoporosis, Postmenopausal | 14.1 |
| CYBC1 | Osteoporosis, Postmenopausal | 14.09 |
| CYP17A1 | Osteoporosis, Postmenopausal | 14.09 |
| LIPC | Osteoporosis, Postmenopausal | 14.09 |
| TKT | Osteoporosis, Postmenopausal | 14.09 |
| TRIB1 | Osteoporosis, Postmenopausal | 14.09 |
| GAS6 | Osteoporosis, Postmenopausal | 14.08 |
| PRC1 | Osteoporosis, Postmenopausal | 14.08 |
| SLC11A2 | Osteoporosis, Postmenopausal | 14.08 |
| ABCC5 | Osteoporosis, Postmenopausal | 14.07 |
| AKAP12 | Osteoporosis, Postmenopausal | 14.07 |
| EIF4E | Osteoporosis, Postmenopausal | 14.07 |
| IL11 | Osteoporosis, Postmenopausal | 14.07 |
| ACBD6 | Osteoporosis, Postmenopausal | 14.06 |
| CEACAM19 | Osteoporosis, Postmenopausal | 14.06 |
| ISM2 | Osteoporosis, Postmenopausal | 14.06 |
| KREMEN2 | Osteoporosis, Postmenopausal | 14.06 |
| MIR185 | Osteoporosis, Postmenopausal | 14.06 |
| NCF2 | Osteoporosis, Postmenopausal | 14.06 |
| ROM1 | Osteoporosis, Postmenopausal | 14.06 |
| RRM1 | Osteoporosis, Postmenopausal | 14.06 |
| SLC20A1 | Osteoporosis, Postmenopausal | 14.06 |
| ZNF79 | Osteoporosis, Postmenopausal | 14.06 |
| RNF113A | Osteoporosis, Postmenopausal | 14.05 |
| UCP1 | Osteoporosis, Postmenopausal | 14.05 |
| CAPN2 | Osteoporosis, Postmenopausal | 14.04 |
| HMMR | Osteoporosis, Postmenopausal | 14.04 |
| IFIT3 | Osteoporosis, Postmenopausal | 14.04 |
| IL1RL1 | Osteoporosis, Postmenopausal | 14.04 |
| HEXB | Osteoporosis, Postmenopausal | 14.03 |
| PLK3 | Osteoporosis, Postmenopausal | 14.03 |
| SLC38A2 | Osteoporosis, Postmenopausal | 14.02 |
| CLDN4 | Osteoporosis, Postmenopausal | 14.01 |
| CTSS | Osteoporosis, Postmenopausal | 14.01 |
| KITLG | Osteoporosis, Postmenopausal | 14.01 |
| CDC25B | Osteoporosis, Postmenopausal | 14 |
| TSEN34 | Osteoporosis, Postmenopausal | 14 |
| ALDH3A2 | Osteoporosis, Postmenopausal | 13.99 |
| BPIFB1 | Osteoporosis, Postmenopausal | 13.99 |
| FCHSD1 | Osteoporosis, Postmenopausal | 13.99 |
| ITGA5 | Osteoporosis, Postmenopausal | 13.99 |
| MIRLET7E | Osteoporosis, Postmenopausal | 13.99 |
| MUP1 | Osteoporosis, Postmenopausal | 13.99 |
| NEDD9 | Osteoporosis, Postmenopausal | 13.99 |
| ADRB1 | Osteoporosis, Postmenopausal | 13.98 |
| ALDH1A3 | Osteoporosis, Postmenopausal | 13.98 |
| GLUD2 | Osteoporosis, Postmenopausal | 13.98 |
| SERPINA1 | Osteoporosis, Postmenopausal | 13.98 |
| TMED6 | Osteoporosis, Postmenopausal | 13.98 |
| RC3H2 | Osteoporosis, Postmenopausal | 13.97 |
| AGTR1B | Osteoporosis, Postmenopausal | 13.96 |
| PRDX6 | Osteoporosis, Postmenopausal | 13.96 |
| ACAA2 | Osteoporosis, Postmenopausal | 13.95 |
| CDC25A | Osteoporosis, Postmenopausal | 13.95 |
| TAC4 | Osteoporosis, Postmenopausal | 13.95 |
| DAPK1 | Osteoporosis, Postmenopausal | 13.94 |
| DDIT3 | Osteoporosis, Postmenopausal | 13.92 |
| HPGD | Osteoporosis, Postmenopausal | 13.92 |
| MIR503 | Osteoporosis, Postmenopausal | 13.92 |
| NGFR | Osteoporosis, Postmenopausal | 13.92 |
| FAM200C | Osteoporosis, Postmenopausal | 13.91 |
| FOXM1 | Osteoporosis, Postmenopausal | 13.9 |
| CSN2 | Osteoporosis, Postmenopausal | 13.89 |
| P4HB | Osteoporosis, Postmenopausal | 13.89 |
| PTX3 | Osteoporosis, Postmenopausal | 13.89 |
| EEF1A1 | Osteoporosis, Postmenopausal | 13.88 |
| PLD1 | Osteoporosis, Postmenopausal | 13.88 |
| HSPA2 | Osteoporosis, Postmenopausal | 13.87 |
| ZNF362 | Osteoporosis, Postmenopausal | 13.87 |
| FLRT1 | Osteoporosis, Postmenopausal | 13.86 |
| MCM3 | Osteoporosis, Postmenopausal | 13.86 |
| PLA2G10 | Osteoporosis, Postmenopausal | 13.86 |
| CDK5R2 | Osteoporosis, Postmenopausal | 13.85 |
| HPF1 | Osteoporosis, Postmenopausal | 13.85 |
| IFIT1 | Osteoporosis, Postmenopausal | 13.85 |
| PFKP | Osteoporosis, Postmenopausal | 13.85 |
| PPP1R3C | Osteoporosis, Postmenopausal | 13.85 |
| CFL1 | Osteoporosis, Postmenopausal | 13.83 |
| TIPARP | Osteoporosis, Postmenopausal | 13.83 |
| GAP43 | Osteoporosis, Postmenopausal | 13.82 |
| RAB4B | Osteoporosis, Postmenopausal | 13.82 |
| TNFRSF1B | Osteoporosis, Postmenopausal | 13.8 |
| C3ORF52 | Osteoporosis, Postmenopausal | 13.79 |
| PKD2L1 | Osteoporosis, Postmenopausal | 13.79 |
| ADORA2A | Osteoporosis, Postmenopausal | 13.78 |
| SELENOT | Osteoporosis, Postmenopausal | 13.78 |
| TGM4 | Osteoporosis, Postmenopausal | 13.78 |
| LEF1 | Osteoporosis, Postmenopausal | 13.76 |
| SLCO2B1 | Osteoporosis, Postmenopausal | 13.76 |
| APCDD1L | Osteoporosis, Postmenopausal | 13.75 |
| ECH1 | Osteoporosis, Postmenopausal | 13.75 |
| FAM131B | Osteoporosis, Postmenopausal | 13.75 |
| MIR134 | Osteoporosis, Postmenopausal | 13.75 |
| C11ORF54 | Osteoporosis, Postmenopausal | 13.72 |
| COL6A6 | Osteoporosis, Postmenopausal | 13.72 |
| COMMD8 | Osteoporosis, Postmenopausal | 13.72 |
| CYP4F11 | Osteoporosis, Postmenopausal | 13.72 |
| EN2 | Osteoporosis, Postmenopausal | 13.72 |
| GPX5 | Osteoporosis, Postmenopausal | 13.72 |
| PRCC | Osteoporosis, Postmenopausal | 13.72 |
| TM4SF5 | Osteoporosis, Postmenopausal | 13.72 |
| CBS | Osteoporosis, Postmenopausal | 13.71 |
| FMO1 | Osteoporosis, Postmenopausal | 13.71 |
| HBG1 | Osteoporosis, Postmenopausal | 13.71 |
| NRP1 | Osteoporosis, Postmenopausal | 13.71 |
| KLF9 | Osteoporosis, Postmenopausal | 13.7 |
| SIRT1 | Osteoporosis, Postmenopausal | 13.7 |
| TSPO | Osteoporosis, Postmenopausal | 13.7 |
| CUZD1 | Osteoporosis, Postmenopausal | 13.69 |
| GK5 | Osteoporosis, Postmenopausal | 13.69 |
| HOXA13 | Osteoporosis, Postmenopausal | 13.69 |
| SLC22A6 | Osteoporosis, Postmenopausal | 13.69 |
| SNX30 | Osteoporosis, Postmenopausal | 13.69 |
| ULBP2 | Osteoporosis, Postmenopausal | 13.69 |
| FOXE1 | Osteoporosis, Postmenopausal | 13.68 |
| TSHB | Osteoporosis, Postmenopausal | 13.67 |
| ARL14 | Osteoporosis, Postmenopausal | 13.66 |
| CEP72 | Osteoporosis, Postmenopausal | 13.66 |
| CYP7B1 | Osteoporosis, Postmenopausal | 13.66 |
| DCN | Osteoporosis, Postmenopausal | 13.66 |
| EPCAM | Osteoporosis, Postmenopausal | 13.66 |
| GPR83 | Osteoporosis, Postmenopausal | 13.66 |
| HMGCR | Osteoporosis, Postmenopausal | 13.66 |
| MAPK8IP2 | Osteoporosis, Postmenopausal | 13.66 |
| ALDOC | Osteoporosis, Postmenopausal | 13.65 |
| GSS | Osteoporosis, Postmenopausal | 13.65 |
| SLPI | Osteoporosis, Postmenopausal | 13.65 |
| CRYBB2 | Osteoporosis, Postmenopausal | 13.64 |
| BAG3 | Osteoporosis, Postmenopausal | 13.63 |
| BMP6 | Osteoporosis, Postmenopausal | 13.63 |
| PIM1 | Osteoporosis, Postmenopausal | 13.63 |
| PRDM10 | Osteoporosis, Postmenopausal | 13.63 |
| RAB42 | Osteoporosis, Postmenopausal | 13.63 |
| SPAG16 | Osteoporosis, Postmenopausal | 13.63 |
| AQP1 | Osteoporosis, Postmenopausal | 13.62 |
| SULT2A1 | Osteoporosis, Postmenopausal | 13.62 |
| TMPRSS3 | Osteoporosis, Postmenopausal | 13.62 |
| VLDLR | Osteoporosis, Postmenopausal | 13.62 |
| ACNAT2 | Osteoporosis, Postmenopausal | 13.6 |
| MISP | Osteoporosis, Postmenopausal | 13.6 |
| PADI1 | Osteoporosis, Postmenopausal | 13.6 |
| RNFT1 | Osteoporosis, Postmenopausal | 13.6 |
| SNHG32 | Osteoporosis, Postmenopausal | 13.6 |
| GNAS | Osteoporosis, Postmenopausal | 13.59 |
| H2AC4 | Osteoporosis, Postmenopausal | 13.59 |
| DRG2 | Osteoporosis, Postmenopausal | 13.57 |
| HSF4 | Osteoporosis, Postmenopausal | 13.57 |
| KDM1B | Osteoporosis, Postmenopausal | 13.57 |
| SDK1 | Osteoporosis, Postmenopausal | 13.57 |
| SLC45A2 | Osteoporosis, Postmenopausal | 13.57 |
| SOCS2 | Osteoporosis, Postmenopausal | 13.57 |
| WDR86 | Osteoporosis, Postmenopausal | 13.57 |
| NEFL | Osteoporosis, Postmenopausal | 13.56 |
| PLK2 | Osteoporosis, Postmenopausal | 13.56 |
| B2M | Osteoporosis, Postmenopausal | 13.55 |
| ASPHD1 | Osteoporosis, Postmenopausal | 13.54 |
| BAIAP2L2 | Osteoporosis, Postmenopausal | 13.54 |
| MEP1A | Osteoporosis, Postmenopausal | 13.54 |
| SLC41A1 | Osteoporosis, Postmenopausal | 13.54 |
| WFDC21 | Osteoporosis, Postmenopausal | 13.54 |
| GAD1 | Osteoporosis, Postmenopausal | 13.53 |
| TSC22D1 | Osteoporosis, Postmenopausal | 13.53 |
| TPI1 | Osteoporosis, Postmenopausal | 13.52 |
| ANGPTL8 | Osteoporosis, Postmenopausal | 13.51 |
| C1ORF116 | Osteoporosis, Postmenopausal | 13.51 |
| CHRNA4 | Osteoporosis, Postmenopausal | 13.51 |
| SOWAHA | Osteoporosis, Postmenopausal | 13.51 |
| ZNF462 | Osteoporosis, Postmenopausal | 13.51 |
| COL26A1 | Osteoporosis, Postmenopausal | 13.5 |
| GPNMB | Osteoporosis, Postmenopausal | 13.49 |
| OLAH | Osteoporosis, Postmenopausal | 13.49 |
| SELENBP1 | Osteoporosis, Postmenopausal | 13.49 |
| ACTBL2 | Osteoporosis, Postmenopausal | 13.48 |
| DUOXA1 | Osteoporosis, Postmenopausal | 13.48 |
| DYNLT2B | Osteoporosis, Postmenopausal | 13.48 |
| HMCN2 | Osteoporosis, Postmenopausal | 13.48 |
| IRF2BP1 | Osteoporosis, Postmenopausal | 13.48 |
| PTCHD4 | Osteoporosis, Postmenopausal | 13.48 |
| WNT9B | Osteoporosis, Postmenopausal | 13.48 |
| AQP3 | Osteoporosis, Postmenopausal | 13.47 |
| DDC | Osteoporosis, Postmenopausal | 13.47 |
| AOX1 | Osteoporosis, Postmenopausal | 13.46 |
| IFRD1 | Osteoporosis, Postmenopausal | 13.46 |
| LRRC75B | Osteoporosis, Postmenopausal | 13.45 |
| NSUN6 | Osteoporosis, Postmenopausal | 13.45 |
| PADI3 | Osteoporosis, Postmenopausal | 13.45 |
| POU5F1 | Osteoporosis, Postmenopausal | 13.45 |
| RNF185 | Osteoporosis, Postmenopausal | 13.45 |
| SLC16A1 | Osteoporosis, Postmenopausal | 13.45 |
| SLC40A1 | Osteoporosis, Postmenopausal | 13.45 |
| TLCD3A | Osteoporosis, Postmenopausal | 13.45 |
| ABCG5 | Osteoporosis, Postmenopausal | 13.44 |
| AIF1 | Osteoporosis, Postmenopausal | 13.44 |
| TGFBR1 | Osteoporosis, Postmenopausal | 13.44 |
| CREG2 | Osteoporosis, Postmenopausal | 13.43 |
| SI | Osteoporosis, Postmenopausal | 13.43 |
| TUBA1A | Osteoporosis, Postmenopausal | 13.43 |
| COG6 | Osteoporosis, Postmenopausal | 13.42 |
| GALNT4 | Osteoporosis, Postmenopausal | 13.42 |
| HS1BP3 | Osteoporosis, Postmenopausal | 13.42 |
| IRF7 | Osteoporosis, Postmenopausal | 13.42 |
| MYF5 | Osteoporosis, Postmenopausal | 13.42 |
| POLM | Osteoporosis, Postmenopausal | 13.42 |
| ANAPC16 | Osteoporosis, Postmenopausal | 13.4 |
| CHST9 | Osteoporosis, Postmenopausal | 13.4 |
| LGALS1 | Osteoporosis, Postmenopausal | 13.4 |
| MIR193A | Osteoporosis, Postmenopausal | 13.4 |
| MKS1 | Osteoporosis, Postmenopausal | 13.4 |
| NUMBL | Osteoporosis, Postmenopausal | 13.4 |
| PLAC9 | Osteoporosis, Postmenopausal | 13.4 |
| PRR3 | Osteoporosis, Postmenopausal | 13.4 |
| SSTR5 | Osteoporosis, Postmenopausal | 13.4 |
| CRISPLD1 | Osteoporosis, Postmenopausal | 13.38 |
| HSPB3 | Osteoporosis, Postmenopausal | 13.38 |
| SECTM1 | Osteoporosis, Postmenopausal | 13.38 |
| SPX | Osteoporosis, Postmenopausal | 13.38 |
| FAM234B | Osteoporosis, Postmenopausal | 13.37 |
| FAM3D | Osteoporosis, Postmenopausal | 13.37 |
| PPP4R3A | Osteoporosis, Postmenopausal | 13.37 |
| PTTG1 | Osteoporosis, Postmenopausal | 13.37 |
| STARD3 | Osteoporosis, Postmenopausal | 13.37 |
| SULT2A2 | Osteoporosis, Postmenopausal | 13.37 |
| VCPKMT | Osteoporosis, Postmenopausal | 13.37 |
| AHR | Osteoporosis, Postmenopausal | 13.36 |
| HSH2D | Osteoporosis, Postmenopausal | 13.36 |
| AKT2 | Osteoporosis, Postmenopausal | 13.35 |
| BCL2L12 | Osteoporosis, Postmenopausal | 13.35 |
| FGFR2 | Osteoporosis, Postmenopausal | 13.35 |
| HDAC1 | Osteoporosis, Postmenopausal | 13.35 |
| MAMLD1 | Osteoporosis, Postmenopausal | 13.35 |
| SAT1 | Osteoporosis, Postmenopausal | 13.35 |
| ALAS1 | Osteoporosis, Postmenopausal | 13.34 |
| CYP2C8 | Osteoporosis, Postmenopausal | 13.34 |
| PDK1 | Osteoporosis, Postmenopausal | 13.34 |
| SCML2 | Osteoporosis, Postmenopausal | 13.34 |
| STARD9 | Osteoporosis, Postmenopausal | 13.33 |
| ENO2 | Osteoporosis, Postmenopausal | 13.32 |
| ARL6 | Osteoporosis, Postmenopausal | 13.31 |
| DAPL1 | Osteoporosis, Postmenopausal | 13.31 |
| GALNT5 | Osteoporosis, Postmenopausal | 13.31 |
| HC | Osteoporosis, Postmenopausal | 13.31 |
| NPAS3 | Osteoporosis, Postmenopausal | 13.31 |
| TSPYL1 | Osteoporosis, Postmenopausal | 13.31 |
| ZNF704 | Osteoporosis, Postmenopausal | 13.31 |
| GP96 | Osteoporosis, Postmenopausal | 13.3 |
| SOX2 | Osteoporosis, Postmenopausal | 13.3 |
| TRAV26-2 | Osteoporosis, Postmenopausal | 13.3 |
| EIF2S3 | Osteoporosis, Postmenopausal | 13.29 |
| MAPKBP1 | Osteoporosis, Postmenopausal | 13.29 |
| S100A4 | Osteoporosis, Postmenopausal | 13.29 |
| TXNDC11 | Osteoporosis, Postmenopausal | 13.29 |
| WDFY2 | Osteoporosis, Postmenopausal | 13.29 |
| ATP23 | Osteoporosis, Postmenopausal | 13.27 |
| CPA3 | Osteoporosis, Postmenopausal | 13.27 |
| SSC5D | Osteoporosis, Postmenopausal | 13.27 |
| TMEM200A | Osteoporosis, Postmenopausal | 13.27 |
| ZNF165 | Osteoporosis, Postmenopausal | 13.27 |
| RPE | Osteoporosis, Postmenopausal | 13.26 |
| SULT1A2 | Osteoporosis, Postmenopausal | 13.26 |
| ZNF385B | Osteoporosis, Postmenopausal | 13.26 |
| CAMSAP3 | Osteoporosis, Postmenopausal | 13.24 |
| CCDC15 | Osteoporosis, Postmenopausal | 13.24 |
| GEMIN2 | Osteoporosis, Postmenopausal | 13.24 |
| IPO13 | Osteoporosis, Postmenopausal | 13.24 |
| MIR17 | Osteoporosis, Postmenopausal | 13.24 |
| MMP14 | Osteoporosis, Postmenopausal | 13.24 |
| SLC24A2 | Osteoporosis, Postmenopausal | 13.24 |
| TMEM139 | Osteoporosis, Postmenopausal | 13.24 |
| CENPO | Osteoporosis, Postmenopausal | 13.23 |
| CYSRT1 | Osteoporosis, Postmenopausal | 13.23 |
| HAPSTR1 | Osteoporosis, Postmenopausal | 13.23 |
| ITGB2 | Osteoporosis, Postmenopausal | 13.23 |
| SERF2 | Osteoporosis, Postmenopausal | 13.23 |
| UPRT | Osteoporosis, Postmenopausal | 13.23 |
| CAPN9 | Osteoporosis, Postmenopausal | 13.21 |
| CLTRN | Osteoporosis, Postmenopausal | 13.21 |
| LGI3 | Osteoporosis, Postmenopausal | 13.21 |
| RIMKLA | Osteoporosis, Postmenopausal | 13.21 |
| SERPINB3 | Osteoporosis, Postmenopausal | 13.21 |
| NGLY1 | Osteoporosis, Postmenopausal | 13.2 |
| POM121 | Osteoporosis, Postmenopausal | 13.2 |
| RHBDD3 | Osteoporosis, Postmenopausal | 13.2 |
| TIMM8A | Osteoporosis, Postmenopausal | 13.2 |
| CGAS | Osteoporosis, Postmenopausal | 13.19 |
| KLRK1 | Osteoporosis, Postmenopausal | 13.19 |
| NAMPT | Osteoporosis, Postmenopausal | 13.19 |
| NME6 | Osteoporosis, Postmenopausal | 13.19 |
| PCK2 | Osteoporosis, Postmenopausal | 13.19 |
| CASP1 | Osteoporosis, Postmenopausal | 13.18 |
| ENTPD8 | Osteoporosis, Postmenopausal | 13.18 |
| SLC22A8 | Osteoporosis, Postmenopausal | 13.18 |
| SLC26A11 | Osteoporosis, Postmenopausal | 13.18 |
| TTF1 | Osteoporosis, Postmenopausal | 13.18 |
| ZYG11B | Osteoporosis, Postmenopausal | 13.18 |
| CLDN8 | Osteoporosis, Postmenopausal | 13.17 |
| DNAJB1 | Osteoporosis, Postmenopausal | 13.17 |
| DSG3 | Osteoporosis, Postmenopausal | 13.17 |
| EHHADH | Osteoporosis, Postmenopausal | 13.17 |
| FAM53C | Osteoporosis, Postmenopausal | 13.17 |
| PLAAT4 | Osteoporosis, Postmenopausal | 13.17 |
| SOST | Osteoporosis, Postmenopausal | 13.16 |
| CELF3 | Osteoporosis, Postmenopausal | 13.15 |
| COX4I2 | Osteoporosis, Postmenopausal | 13.15 |
| GTF2E2 | Osteoporosis, Postmenopausal | 13.15 |
| HAPLN2 | Osteoporosis, Postmenopausal | 13.15 |
| MIR10A | Osteoporosis, Postmenopausal | 13.15 |
| MYLK2 | Osteoporosis, Postmenopausal | 13.15 |
| PHYKPL | Osteoporosis, Postmenopausal | 13.15 |
| PRSS16 | Osteoporosis, Postmenopausal | 13.15 |
| RNASEH1 | Osteoporosis, Postmenopausal | 13.15 |
| SLC2A14 | Osteoporosis, Postmenopausal | 13.15 |
| PGK1 | Osteoporosis, Postmenopausal | 13.14 |
| SLC37A3 | Osteoporosis, Postmenopausal | 13.14 |
| ALDH3B2 | Osteoporosis, Postmenopausal | 13.13 |
| DLK2 | Osteoporosis, Postmenopausal | 13.13 |
| PROM2 | Osteoporosis, Postmenopausal | 13.13 |
| RMI1 | Osteoporosis, Postmenopausal | 13.13 |
| UBXN7 | Osteoporosis, Postmenopausal | 13.13 |
| PEX5 | Osteoporosis, Postmenopausal | 13.12 |
| POFUT1 | Osteoporosis, Postmenopausal | 13.12 |
| GLYAT | Osteoporosis, Postmenopausal | 13.11 |
| NR1H3 | Osteoporosis, Postmenopausal | 13.11 |
| BCL2L15 | Osteoporosis, Postmenopausal | 13.1 |
| CHML | Osteoporosis, Postmenopausal | 13.1 |
| GNGT2 | Osteoporosis, Postmenopausal | 13.1 |
| METTL18 | Osteoporosis, Postmenopausal | 13.1 |
| MIR206 | Osteoporosis, Postmenopausal | 13.1 |
| MTERF2 | Osteoporosis, Postmenopausal | 13.1 |
| RNF183 | Osteoporosis, Postmenopausal | 13.1 |
| SETMAR | Osteoporosis, Postmenopausal | 13.1 |
| TMA16 | Osteoporosis, Postmenopausal | 13.1 |
| CNIH2 | Osteoporosis, Postmenopausal | 13.09 |
| KNG1 | Osteoporosis, Postmenopausal | 13.09 |
| MS4A6A | Osteoporosis, Postmenopausal | 13.09 |
| ARSI | Osteoporosis, Postmenopausal | 13.08 |
| BRF2 | Osteoporosis, Postmenopausal | 13.08 |
| CASP2 | Osteoporosis, Postmenopausal | 13.08 |
| MPPED2 | Osteoporosis, Postmenopausal | 13.08 |
| QSOX2 | Osteoporosis, Postmenopausal | 13.08 |
| SLC7A4 | Osteoporosis, Postmenopausal | 13.08 |
| DHCR7 | Osteoporosis, Postmenopausal | 13.07 |
| DNMT3A | Osteoporosis, Postmenopausal | 13.07 |
| RGS2 | Osteoporosis, Postmenopausal | 13.07 |
| TPM1 | Osteoporosis, Postmenopausal | 13.07 |
| WBP1L | Osteoporosis, Postmenopausal | 13.06 |
| BRINP3 | Osteoporosis, Postmenopausal | 13.05 |
| CHODL | Osteoporosis, Postmenopausal | 13.05 |
| FZD9 | Osteoporosis, Postmenopausal | 13.05 |
| GGT1 | Osteoporosis, Postmenopausal | 13.05 |
| KCNF1 | Osteoporosis, Postmenopausal | 13.05 |
| KLHL18 | Osteoporosis, Postmenopausal | 13.05 |
| NBEA | Osteoporosis, Postmenopausal | 13.05 |
| TIGD2 | Osteoporosis, Postmenopausal | 13.05 |
| GABPB2 | Osteoporosis, Postmenopausal | 13.04 |
| MIR20A | Osteoporosis, Postmenopausal | 13.04 |
| IPO11 | Osteoporosis, Postmenopausal | 13.03 |
| MAP7D2 | Osteoporosis, Postmenopausal | 13.02 |
| MXRA5 | Osteoporosis, Postmenopausal | 13.02 |
| P2RY10 | Osteoporosis, Postmenopausal | 13.01 |
| RMND1 | Osteoporosis, Postmenopausal | 13.01 |
| EDARADD | Osteoporosis, Postmenopausal | 13 |
| EXOSC7 | Osteoporosis, Postmenopausal | 13 |
| GADD45G | Osteoporosis, Postmenopausal | 13 |
| GOLGA8A | Osteoporosis, Postmenopausal | 13 |
| NEK4 | Osteoporosis, Postmenopausal | 13 |
| VXN | Osteoporosis, Postmenopausal | 13 |
| CACNG4 | Osteoporosis, Postmenopausal | 12.99 |
| ENOSF1 | Osteoporosis, Postmenopausal | 12.99 |
| H3-4 | Osteoporosis, Postmenopausal | 12.99 |
| PSD4 | Osteoporosis, Postmenopausal | 12.99 |
| NR1I3 | Osteoporosis, Postmenopausal | 12.98 |
| PPP4R4 | Osteoporosis, Postmenopausal | 12.98 |
| PTPRCAP | Osteoporosis, Postmenopausal | 12.98 |
| RAC1 | Osteoporosis, Postmenopausal | 12.98 |
| USP32 | Osteoporosis, Postmenopausal | 12.98 |
| HS3ST3A1 | Osteoporosis, Postmenopausal | 12.97 |
| POLR2I | Osteoporosis, Postmenopausal | 12.97 |
| MGA | Osteoporosis, Postmenopausal | 12.95 |
| OSBPL7 | Osteoporosis, Postmenopausal | 12.95 |
| PRIMA1 | Osteoporosis, Postmenopausal | 12.95 |
| SNX14 | Osteoporosis, Postmenopausal | 12.95 |
| SPIRE2 | Osteoporosis, Postmenopausal | 12.95 |
| USP6NL | Osteoporosis, Postmenopausal | 12.95 |
| H1-3 | Osteoporosis, Postmenopausal | 12.94 |
| LMNA | Osteoporosis, Postmenopausal | 12.94 |
| PRSS22 | Osteoporosis, Postmenopausal | 12.94 |
| RABEPK | Osteoporosis, Postmenopausal | 12.94 |
| TMEM86B | Osteoporosis, Postmenopausal | 12.94 |
| TMX3 | Osteoporosis, Postmenopausal | 12.94 |
| KRT34 | Osteoporosis, Postmenopausal | 12.93 |
| MSH5 | Osteoporosis, Postmenopausal | 12.93 |
| RAPGEFL1 | Osteoporosis, Postmenopausal | 12.93 |
| SLC6A3 | Osteoporosis, Postmenopausal | 12.93 |
| BRAP | Osteoporosis, Postmenopausal | 12.92 |
| CCND3 | Osteoporosis, Postmenopausal | 12.92 |
| CD300A | Osteoporosis, Postmenopausal | 12.92 |
| DIO1 | Osteoporosis, Postmenopausal | 12.92 |
| ACTR6 | Osteoporosis, Postmenopausal | 12.91 |
| DEGS2 | Osteoporosis, Postmenopausal | 12.91 |
| GLRX5 | Osteoporosis, Postmenopausal | 12.91 |
| MIR27A | Osteoporosis, Postmenopausal | 12.91 |
| PLA2G2D | Osteoporosis, Postmenopausal | 12.91 |
| PRDM8 | Osteoporosis, Postmenopausal | 12.91 |
| RCBTB1 | Osteoporosis, Postmenopausal | 12.91 |
| RRP1 | Osteoporosis, Postmenopausal | 12.91 |
| SHROOM1 | Osteoporosis, Postmenopausal | 12.91 |
| SLC2A10 | Osteoporosis, Postmenopausal | 12.91 |
| SPSB3 | Osteoporosis, Postmenopausal | 12.91 |
| TAF5 | Osteoporosis, Postmenopausal | 12.91 |
| ZNF703 | Osteoporosis, Postmenopausal | 12.91 |
| BMP3 | Osteoporosis, Postmenopausal | 12.89 |
| GOLT1A | Osteoporosis, Postmenopausal | 12.89 |
| KHDC4 | Osteoporosis, Postmenopausal | 12.89 |
| NLRP1 | Osteoporosis, Postmenopausal | 12.89 |
| PRKCB | Osteoporosis, Postmenopausal | 12.89 |
| RUNDC3B | Osteoporosis, Postmenopausal | 12.89 |
| SLC49A4 | Osteoporosis, Postmenopausal | 12.89 |
| H2-M10.1 | Osteoporosis, Postmenopausal | 12.88 |
| LRRN1 | Osteoporosis, Postmenopausal | 12.88 |
| MED14 | Osteoporosis, Postmenopausal | 12.88 |
| AFP | Osteoporosis, Postmenopausal | 12.87 |
| CYP1A2 | Osteoporosis, Postmenopausal | 12.87 |
| ABRACL | Osteoporosis, Postmenopausal | 12.86 |
| CD24A | Osteoporosis, Postmenopausal | 12.86 |
| CSRNP2 | Osteoporosis, Postmenopausal | 12.86 |
| GPRIN1 | Osteoporosis, Postmenopausal | 12.86 |
| MIR27B | Osteoporosis, Postmenopausal | 12.86 |
| PUS10 | Osteoporosis, Postmenopausal | 12.86 |
| RNF141 | Osteoporosis, Postmenopausal | 12.86 |
| SLC35F3 | Osteoporosis, Postmenopausal | 12.86 |
| CD58 | Osteoporosis, Postmenopausal | 12.85 |
| FYTTD1 | Osteoporosis, Postmenopausal | 12.85 |
| IPO8 | Osteoporosis, Postmenopausal | 12.85 |
| PHF8 | Osteoporosis, Postmenopausal | 12.85 |
| PLEKHG2 | Osteoporosis, Postmenopausal | 12.85 |
| REPS2 | Osteoporosis, Postmenopausal | 12.85 |
| APOL1 | Osteoporosis, Postmenopausal | 12.84 |
| CCL20 | Osteoporosis, Postmenopausal | 12.84 |
| CLEC3B | Osteoporosis, Postmenopausal | 12.84 |
| CRCP | Osteoporosis, Postmenopausal | 12.83 |
| KCNA1 | Osteoporosis, Postmenopausal | 12.83 |
| NQO1 | Osteoporosis, Postmenopausal | 12.83 |
| RBM17 | Osteoporosis, Postmenopausal | 12.83 |
| BCAN | Osteoporosis, Postmenopausal | 12.82 |
| METTL16 | Osteoporosis, Postmenopausal | 12.82 |
| NEIL2 | Osteoporosis, Postmenopausal | 12.82 |
| UBQLN4 | Osteoporosis, Postmenopausal | 12.82 |
| BRINP1 | Osteoporosis, Postmenopausal | 12.81 |
| CAMTA2 | Osteoporosis, Postmenopausal | 12.81 |
| EXOSC6 | Osteoporosis, Postmenopausal | 12.81 |
| HPCA | Osteoporosis, Postmenopausal | 12.81 |
| HSPBAP1 | Osteoporosis, Postmenopausal | 12.81 |
| IDH2 | Osteoporosis, Postmenopausal | 12.81 |
| NKAIN1 | Osteoporosis, Postmenopausal | 12.81 |
| NRBF2 | Osteoporosis, Postmenopausal | 12.81 |
| RABAC1 | Osteoporosis, Postmenopausal | 12.81 |
| TFF2 | Osteoporosis, Postmenopausal | 12.81 |
| YTHDC2 | Osteoporosis, Postmenopausal | 12.81 |
| AKAP11 | Osteoporosis, Postmenopausal | 12.8 |
| ALDOA | Osteoporosis, Postmenopausal | 12.8 |
| CPN2 | Osteoporosis, Postmenopausal | 12.8 |
| CASP14 | Osteoporosis, Postmenopausal | 12.79 |
| INSR | Osteoporosis, Postmenopausal | 12.79 |
| P2RY14 | Osteoporosis, Postmenopausal | 12.79 |
| PIGN | Osteoporosis, Postmenopausal | 12.79 |
| URB2 | Osteoporosis, Postmenopausal | 12.79 |
| EPHX2 | Osteoporosis, Postmenopausal | 12.78 |
| FAP | Osteoporosis, Postmenopausal | 12.78 |
| KRT8 | Osteoporosis, Postmenopausal | 12.78 |
| MAOB | Osteoporosis, Postmenopausal | 12.78 |
| CCDC88B | Osteoporosis, Postmenopausal | 12.77 |
| COA7 | Osteoporosis, Postmenopausal | 12.77 |
| CRB2 | Osteoporosis, Postmenopausal | 12.77 |
| FAM135A | Osteoporosis, Postmenopausal | 12.77 |
| FICD | Osteoporosis, Postmenopausal | 12.77 |
| MUC16 | Osteoporosis, Postmenopausal | 12.77 |
| RWDD2A | Osteoporosis, Postmenopausal | 12.77 |
| SNRPB2 | Osteoporosis, Postmenopausal | 12.77 |
| STX8 | Osteoporosis, Postmenopausal | 12.77 |
| SYNE3 | Osteoporosis, Postmenopausal | 12.77 |
| ACLY | Osteoporosis, Postmenopausal | 12.76 |
| APOL6 | Osteoporosis, Postmenopausal | 12.76 |
| BAG4 | Osteoporosis, Postmenopausal | 12.76 |
| CFAP251 | Osteoporosis, Postmenopausal | 12.76 |
| GALR2 | Osteoporosis, Postmenopausal | 12.76 |
| KLHL14 | Osteoporosis, Postmenopausal | 12.76 |
| POLR2B | Osteoporosis, Postmenopausal | 12.76 |
| VDR-B | Osteoporosis, Postmenopausal | 12.76 |
| SYT8 | Osteoporosis, Postmenopausal | 12.75 |
| CFLAR | Osteoporosis, Postmenopausal | 12.74 |
| SFTPA1 | Osteoporosis, Postmenopausal | 12.74 |
| TONSL | Osteoporosis, Postmenopausal | 12.74 |
| AKR1B10 | Osteoporosis, Postmenopausal | 12.73 |
| CREB3L4 | Osteoporosis, Postmenopausal | 12.73 |
| CSF3 | Osteoporosis, Postmenopausal | 12.73 |
| GLT1D1 | Osteoporosis, Postmenopausal | 12.73 |
| RAB26 | Osteoporosis, Postmenopausal | 12.73 |
| IGDCC3 | Osteoporosis, Postmenopausal | 12.72 |
| SCLT1 | Osteoporosis, Postmenopausal | 12.72 |
| TICAM2 | Osteoporosis, Postmenopausal | 12.72 |
| ZIC2 | Osteoporosis, Postmenopausal | 12.72 |
| CTSV | Osteoporosis, Postmenopausal | 12.71 |
| MSMB | Osteoporosis, Postmenopausal | 12.71 |
| NUP133 | Osteoporosis, Postmenopausal | 12.71 |
| SLC7A9 | Osteoporosis, Postmenopausal | 12.71 |
| CPXM2 | Osteoporosis, Postmenopausal | 12.7 |
| HAUS1 | Osteoporosis, Postmenopausal | 12.7 |
| LCT | Osteoporosis, Postmenopausal | 12.7 |
| LRRC15 | Osteoporosis, Postmenopausal | 12.7 |
| MIR200A | Osteoporosis, Postmenopausal | 12.7 |
| CACNA1S | Osteoporosis, Postmenopausal | 12.68 |
| CHPF | Osteoporosis, Postmenopausal | 12.68 |
| COX7A2L | Osteoporosis, Postmenopausal | 12.68 |
| CTTNBP2 | Osteoporosis, Postmenopausal | 12.68 |
| CYP2A6 | Osteoporosis, Postmenopausal | 12.68 |
| DLL3 | Osteoporosis, Postmenopausal | 12.68 |
| DPY19L3 | Osteoporosis, Postmenopausal | 12.68 |
| EID3 | Osteoporosis, Postmenopausal | 12.68 |
| KCNJ10 | Osteoporosis, Postmenopausal | 12.68 |
| MFSD4A | Osteoporosis, Postmenopausal | 12.68 |
| NOS1AP | Osteoporosis, Postmenopausal | 12.68 |
| PEX3 | Osteoporosis, Postmenopausal | 12.68 |
| SCTR | Osteoporosis, Postmenopausal | 12.68 |
| SEMA3D | Osteoporosis, Postmenopausal | 12.68 |
| SEPSECS | Osteoporosis, Postmenopausal | 12.68 |
| SLC5A9 | Osteoporosis, Postmenopausal | 12.68 |
| TSEN15 | Osteoporosis, Postmenopausal | 12.68 |
| ZNF608 | Osteoporosis, Postmenopausal | 12.68 |
| CCP110 | Osteoporosis, Postmenopausal | 12.67 |
| KIF7 | Osteoporosis, Postmenopausal | 12.67 |
| UNC5CL | Osteoporosis, Postmenopausal | 12.67 |
| CYP3A5 | Osteoporosis, Postmenopausal | 12.66 |
| FABP1 | Osteoporosis, Postmenopausal | 12.66 |
| NUP35 | Osteoporosis, Postmenopausal | 12.66 |
| ACSL1 | Osteoporosis, Postmenopausal | 12.65 |
| C15ORF48 | Osteoporosis, Postmenopausal | 12.65 |
| GRAMD1A | Osteoporosis, Postmenopausal | 12.65 |
| BAXA | Osteoporosis, Postmenopausal | 12.64 |
| BBS4 | Osteoporosis, Postmenopausal | 12.64 |
| KLHL2 | Osteoporosis, Postmenopausal | 12.64 |
| PHC2 | Osteoporosis, Postmenopausal | 12.64 |
| PLA2G12B | Osteoporosis, Postmenopausal | 12.64 |
| RILPL2 | Osteoporosis, Postmenopausal | 12.64 |
| ROS1 | Osteoporosis, Postmenopausal | 12.64 |
| SBSPON | Osteoporosis, Postmenopausal | 12.64 |
| SLC35F5 | Osteoporosis, Postmenopausal | 12.64 |
| STXBP5 | Osteoporosis, Postmenopausal | 12.64 |
| ZFP354A | Osteoporosis, Postmenopausal | 12.64 |
| CSNK1G3 | Osteoporosis, Postmenopausal | 12.63 |
| CYP19A1A | Osteoporosis, Postmenopausal | 12.63 |
| RASEF | Osteoporosis, Postmenopausal | 12.63 |
| TAGLN | Osteoporosis, Postmenopausal | 12.63 |
| CYP1A | Osteoporosis, Postmenopausal | 12.62 |
| MTMR6 | Osteoporosis, Postmenopausal | 12.62 |
| SCEL | Osteoporosis, Postmenopausal | 12.62 |
| APOB | Osteoporosis, Postmenopausal | 12.61 |
| HSPB9 | Osteoporosis, Postmenopausal | 12.61 |
| DGKE | Osteoporosis, Postmenopausal | 12.6 |
| FCHO2 | Osteoporosis, Postmenopausal | 12.6 |
| HNRNPUL2 | Osteoporosis, Postmenopausal | 12.6 |
| IL21R | Osteoporosis, Postmenopausal | 12.6 |
| INTS10 | Osteoporosis, Postmenopausal | 12.6 |
| KCNE1 | Osteoporosis, Postmenopausal | 12.6 |
| NIF3L1 | Osteoporosis, Postmenopausal | 12.6 |
| RNFT2 | Osteoporosis, Postmenopausal | 12.6 |
| RSPO1 | Osteoporosis, Postmenopausal | 12.6 |
| THUMPD1 | Osteoporosis, Postmenopausal | 12.6 |
| TRIM38 | Osteoporosis, Postmenopausal | 12.6 |
| TSC22D4 | Osteoporosis, Postmenopausal | 12.6 |
| IRAG2 | Osteoporosis, Postmenopausal | 12.59 |
| TMEM54 | Osteoporosis, Postmenopausal | 12.59 |
| TMEM67 | Osteoporosis, Postmenopausal | 12.59 |
| ADGRA3 | Osteoporosis, Postmenopausal | 12.58 |
| ELMO2 | Osteoporosis, Postmenopausal | 12.58 |
| NUP107 | Osteoporosis, Postmenopausal | 12.58 |
| OTULINL | Osteoporosis, Postmenopausal | 12.58 |
| PACSIN1 | Osteoporosis, Postmenopausal | 12.58 |
| PTK2 | Osteoporosis, Postmenopausal | 12.58 |
| PTPRH | Osteoporosis, Postmenopausal | 12.58 |
| RNF149 | Osteoporosis, Postmenopausal | 12.58 |
| SNN | Osteoporosis, Postmenopausal | 12.58 |
| ABCA12 | Osteoporosis, Postmenopausal | 12.57 |
| BCAS1 | Osteoporosis, Postmenopausal | 12.57 |
| FIG4 | Osteoporosis, Postmenopausal | 12.57 |
| GSTM3 | Osteoporosis, Postmenopausal | 12.57 |
| NOTCH1 | Osteoporosis, Postmenopausal | 12.57 |
| SNX3 | Osteoporosis, Postmenopausal | 12.57 |
| ANKRD22 | Osteoporosis, Postmenopausal | 12.56 |
| CDC73 | Osteoporosis, Postmenopausal | 12.55 |
| GALNT14 | Osteoporosis, Postmenopausal | 12.55 |
| HYLS1 | Osteoporosis, Postmenopausal | 12.55 |
| OSBPL11 | Osteoporosis, Postmenopausal | 12.55 |
| PRR15 | Osteoporosis, Postmenopausal | 12.55 |
| PYCR2 | Osteoporosis, Postmenopausal | 12.55 |
| CYB5R2 | Osteoporosis, Postmenopausal | 12.54 |
| MIB1 | Osteoporosis, Postmenopausal | 12.54 |
| MRPS12 | Osteoporosis, Postmenopausal | 12.54 |
| TOR1AIP2 | Osteoporosis, Postmenopausal | 12.54 |
| WDR72 | Osteoporosis, Postmenopausal | 12.54 |
| HSPA9 | Osteoporosis, Postmenopausal | 12.53 |
| TIAM2 | Osteoporosis, Postmenopausal | 12.53 |
| ASB13 | Osteoporosis, Postmenopausal | 12.51 |
| CDK10 | Osteoporosis, Postmenopausal | 12.51 |
| GPX3 | Osteoporosis, Postmenopausal | 12.51 |
| GRM3 | Osteoporosis, Postmenopausal | 12.51 |
| HSPA14 | Osteoporosis, Postmenopausal | 12.51 |
| MARCHF3 | Osteoporosis, Postmenopausal | 12.51 |
| NECTIN1 | Osteoporosis, Postmenopausal | 12.51 |
| PRPF6 | Osteoporosis, Postmenopausal | 12.51 |
| SEC11C | Osteoporosis, Postmenopausal | 12.51 |
| TF | Osteoporosis, Postmenopausal | 12.51 |
| TRDN | Osteoporosis, Postmenopausal | 12.51 |
| TSFM | Osteoporosis, Postmenopausal | 12.51 |
| ULBP1 | Osteoporosis, Postmenopausal | 12.51 |
| CALCA | Osteoporosis, Postmenopausal | 12.5 |
| CEP350 | Osteoporosis, Postmenopausal | 12.5 |
| FRK | Osteoporosis, Postmenopausal | 12.5 |
| PLA2G1B | Osteoporosis, Postmenopausal | 12.5 |
| UBE3C | Osteoporosis, Postmenopausal | 12.5 |
| UST | Osteoporosis, Postmenopausal | 12.5 |
| CLEC4E | Osteoporosis, Postmenopausal | 12.49 |
| KIF3C | Osteoporosis, Postmenopausal | 12.49 |
| SERPINB6 | Osteoporosis, Postmenopausal | 12.49 |
| ELF5 | Osteoporosis, Postmenopausal | 12.48 |
| PHTF1 | Osteoporosis, Postmenopausal | 12.48 |
| AMMECR1 | Osteoporosis, Postmenopausal | 12.47 |
| ATP6V0A4 | Osteoporosis, Postmenopausal | 12.47 |
| ELP1 | Osteoporosis, Postmenopausal | 12.47 |
| ERLIN2 | Osteoporosis, Postmenopausal | 12.47 |
| GLMP | Osteoporosis, Postmenopausal | 12.47 |
| GNG4 | Osteoporosis, Postmenopausal | 12.47 |
| KLHDC7A | Osteoporosis, Postmenopausal | 12.47 |
| SGCB | Osteoporosis, Postmenopausal | 12.47 |
| TMEM30B | Osteoporosis, Postmenopausal | 12.47 |
| TRIM5 | Osteoporosis, Postmenopausal | 12.47 |
| TSPYL2 | Osteoporosis, Postmenopausal | 12.47 |
| TWNK | Osteoporosis, Postmenopausal | 12.47 |
| UTP18 | Osteoporosis, Postmenopausal | 12.47 |
| DONSON | Osteoporosis, Postmenopausal | 12.46 |
| ID2 | Osteoporosis, Postmenopausal | 12.46 |
| MEN1 | Osteoporosis, Postmenopausal | 12.46 |
| PGLYRP1 | Osteoporosis, Postmenopausal | 12.46 |
| SRGAP1 | Osteoporosis, Postmenopausal | 12.46 |
| STX16 | Osteoporosis, Postmenopausal | 12.46 |
| TFAP4 | Osteoporosis, Postmenopausal | 12.46 |
| CBX2 | Osteoporosis, Postmenopausal | 12.45 |
| DLX | Osteoporosis, Postmenopausal | 12.45 |
| KRT18 | Osteoporosis, Postmenopausal | 12.45 |
| RGMB | Osteoporosis, Postmenopausal | 12.45 |
| CYP4A2 | Osteoporosis, Postmenopausal | 12.44 |
| PDE4C | Osteoporosis, Postmenopausal | 12.44 |
| RASA4 | Osteoporosis, Postmenopausal | 12.44 |
| THOC1 | Osteoporosis, Postmenopausal | 12.44 |
| BPNT2 | Osteoporosis, Postmenopausal | 12.43 |
| BSN | Osteoporosis, Postmenopausal | 12.43 |
| KLHDC2 | Osteoporosis, Postmenopausal | 12.43 |
| MBLAC2 | Osteoporosis, Postmenopausal | 12.43 |
| MICB | Osteoporosis, Postmenopausal | 12.43 |
| MIR200C | Osteoporosis, Postmenopausal | 12.43 |
| ORAI1 | Osteoporosis, Postmenopausal | 12.43 |
| PBDC1 | Osteoporosis, Postmenopausal | 12.43 |
| PRMT6 | Osteoporosis, Postmenopausal | 12.43 |
| PRR7 | Osteoporosis, Postmenopausal | 12.43 |
| RAVER2 | Osteoporosis, Postmenopausal | 12.43 |
| RGS7 | Osteoporosis, Postmenopausal | 12.43 |
| SNTA1 | Osteoporosis, Postmenopausal | 12.43 |
| TWIST2 | Osteoporosis, Postmenopausal | 12.43 |
| ZFYVE1 | Osteoporosis, Postmenopausal | 12.43 |
| ARHGAP25 | Osteoporosis, Postmenopausal | 12.42 |
| GRHL3 | Osteoporosis, Postmenopausal | 12.42 |
| HAVCR2 | Osteoporosis, Postmenopausal | 12.42 |
| KLK6 | Osteoporosis, Postmenopausal | 12.42 |
| MMS22L | Osteoporosis, Postmenopausal | 12.42 |
| TNS4 | Osteoporosis, Postmenopausal | 12.42 |
| GABPA | Osteoporosis, Postmenopausal | 12.41 |
| GATA1 | Osteoporosis, Postmenopausal | 12.41 |
| MGMT | Osteoporosis, Postmenopausal | 12.41 |
| NCF1 | Osteoporosis, Postmenopausal | 12.41 |
| SASS6 | Osteoporosis, Postmenopausal | 12.41 |
| PEX13 | Osteoporosis, Postmenopausal | 12.4 |
| SHC4 | Osteoporosis, Postmenopausal | 12.4 |
| ACAD10 | Osteoporosis, Postmenopausal | 12.39 |
| AQP2 | Osteoporosis, Postmenopausal | 12.39 |
| ARFGEF2 | Osteoporosis, Postmenopausal | 12.39 |
| BAG5 | Osteoporosis, Postmenopausal | 12.39 |
| BEST1 | Osteoporosis, Postmenopausal | 12.39 |
| CLDN10 | Osteoporosis, Postmenopausal | 12.39 |
| EPN3 | Osteoporosis, Postmenopausal | 12.39 |
| FAM171B | Osteoporosis, Postmenopausal | 12.39 |
| GSTT1 | Osteoporosis, Postmenopausal | 12.39 |
| PDE12 | Osteoporosis, Postmenopausal | 12.39 |
| SH3BGRL2 | Osteoporosis, Postmenopausal | 12.39 |
| SPDEF | Osteoporosis, Postmenopausal | 12.39 |
| SV2B | Osteoporosis, Postmenopausal | 12.39 |
| ZDHHC23 | Osteoporosis, Postmenopausal | 12.39 |
| CXCL6 | Osteoporosis, Postmenopausal | 12.38 |
| ECHDC3 | Osteoporosis, Postmenopausal | 12.38 |
| ENO1 | Osteoporosis, Postmenopausal | 12.38 |
| HOXA10 | Osteoporosis, Postmenopausal | 12.38 |
| HRG | Osteoporosis, Postmenopausal | 12.38 |
| MYBPC1 | Osteoporosis, Postmenopausal | 12.38 |
| PACS1 | Osteoporosis, Postmenopausal | 12.38 |
| TRPV1 | Osteoporosis, Postmenopausal | 12.38 |
| TUB | Osteoporosis, Postmenopausal | 12.38 |
| DOK4 | Osteoporosis, Postmenopausal | 12.37 |
| ARL3 | Osteoporosis, Postmenopausal | 12.36 |
| CEP128 | Osteoporosis, Postmenopausal | 12.36 |
| FOXD1 | Osteoporosis, Postmenopausal | 12.36 |
| GNB4 | Osteoporosis, Postmenopausal | 12.36 |
| PMS2 | Osteoporosis, Postmenopausal | 12.36 |
| POU2AF1 | Osteoporosis, Postmenopausal | 12.36 |
| PRSS35 | Osteoporosis, Postmenopausal | 12.36 |
| RFWD3 | Osteoporosis, Postmenopausal | 12.36 |
| CLASP2 | Osteoporosis, Postmenopausal | 12.35 |
| GABRE | Osteoporosis, Postmenopausal | 12.35 |
| HEATR3 | Osteoporosis, Postmenopausal | 12.35 |
| ME1 | Osteoporosis, Postmenopausal | 12.35 |
| PARG | Osteoporosis, Postmenopausal | 12.35 |
| PRRC2B | Osteoporosis, Postmenopausal | 12.35 |
| SLC33A1 | Osteoporosis, Postmenopausal | 12.35 |
| ALMS1 | Osteoporosis, Postmenopausal | 12.34 |
| HES2 | Osteoporosis, Postmenopausal | 12.34 |
| MOGAT2 | Osteoporosis, Postmenopausal | 12.34 |
| SH3D21 | Osteoporosis, Postmenopausal | 12.34 |
| ZBTB38 | Osteoporosis, Postmenopausal | 12.34 |
| ALDH2 | Osteoporosis, Postmenopausal | 12.33 |
| ABCA6 | Osteoporosis, Postmenopausal | 12.32 |
| KCNJ15 | Osteoporosis, Postmenopausal | 12.32 |
| PELI2 | Osteoporosis, Postmenopausal | 12.32 |
| TRPM6 | Osteoporosis, Postmenopausal | 12.32 |
| TRPV3 | Osteoporosis, Postmenopausal | 12.32 |
| TWIST1B | Osteoporosis, Postmenopausal | 12.32 |
| CAR8 | Osteoporosis, Postmenopausal | 12.31 |
| CHRNE | Osteoporosis, Postmenopausal | 12.31 |
| CMTM7 | Osteoporosis, Postmenopausal | 12.31 |
| DENND3 | Osteoporosis, Postmenopausal | 12.31 |
| EXOC6 | Osteoporosis, Postmenopausal | 12.31 |
| FAIM | Osteoporosis, Postmenopausal | 12.31 |
| MS4A1 | Osteoporosis, Postmenopausal | 12.31 |
| NR2C2 | Osteoporosis, Postmenopausal | 12.31 |
| RPL41 | Osteoporosis, Postmenopausal | 12.31 |
| SENP7 | Osteoporosis, Postmenopausal | 12.31 |
| SNX24 | Osteoporosis, Postmenopausal | 12.31 |
| STN1 | Osteoporosis, Postmenopausal | 12.31 |
| TBC1D9 | Osteoporosis, Postmenopausal | 12.31 |
| WDR36 | Osteoporosis, Postmenopausal | 12.31 |
| AK5 | Osteoporosis, Postmenopausal | 12.3 |
| DENND1B | Osteoporosis, Postmenopausal | 12.3 |
| DRD5 | Osteoporosis, Postmenopausal | 12.3 |
| EVA1B | Osteoporosis, Postmenopausal | 12.3 |
| NFASC | Osteoporosis, Postmenopausal | 12.3 |
| TRIM29 | Osteoporosis, Postmenopausal | 12.3 |
| AKR1C1 | Osteoporosis, Postmenopausal | 12.29 |
| SCN4A | Osteoporosis, Postmenopausal | 12.29 |
| THSD4 | Osteoporosis, Postmenopausal | 12.29 |
| CRHR2 | Osteoporosis, Postmenopausal | 12.28 |
| CRYBG1 | Osteoporosis, Postmenopausal | 12.28 |
| DVL1 | Osteoporosis, Postmenopausal | 12.28 |
| FAM117A | Osteoporosis, Postmenopausal | 12.28 |
| FRS2 | Osteoporosis, Postmenopausal | 12.28 |
| ILF2 | Osteoporosis, Postmenopausal | 12.28 |
| LIPH | Osteoporosis, Postmenopausal | 12.28 |
| MAPK4 | Osteoporosis, Postmenopausal | 12.28 |
| NOC2L | Osteoporosis, Postmenopausal | 12.28 |
| NUBPL | Osteoporosis, Postmenopausal | 12.28 |
| POLD2 | Osteoporosis, Postmenopausal | 12.28 |
| SCRIB | Osteoporosis, Postmenopausal | 12.28 |
| TMEM120A | Osteoporosis, Postmenopausal | 12.28 |
| TWF2 | Osteoporosis, Postmenopausal | 12.28 |
| INS2 | Osteoporosis, Postmenopausal | 12.27 |
| PDSS1 | Osteoporosis, Postmenopausal | 12.27 |
| RIN3 | Osteoporosis, Postmenopausal | 12.27 |
| RNF144A | Osteoporosis, Postmenopausal | 12.27 |
| SMG1 | Osteoporosis, Postmenopausal | 12.27 |
| TMC7 | Osteoporosis, Postmenopausal | 12.27 |
| UBL3 | Osteoporosis, Postmenopausal | 12.27 |
| TXLNA | Osteoporosis, Postmenopausal | 12.26 |
| ALG2 | Osteoporosis, Postmenopausal | 12.25 |
| BSP | Osteoporosis, Postmenopausal | 12.25 |
| CXCR5 | Osteoporosis, Postmenopausal | 12.25 |
| OR1F34 | Osteoporosis, Postmenopausal | 12.25 |
| PAQR5 | Osteoporosis, Postmenopausal | 12.25 |
| ADCY4 | Osteoporosis, Postmenopausal | 12.24 |
| ATP8B2 | Osteoporosis, Postmenopausal | 12.24 |
| BEX4 | Osteoporosis, Postmenopausal | 12.24 |
| C4BPB | Osteoporosis, Postmenopausal | 12.24 |
| CYP4F2 | Osteoporosis, Postmenopausal | 12.24 |
| DDX54 | Osteoporosis, Postmenopausal | 12.24 |
| DKK2 | Osteoporosis, Postmenopausal | 12.24 |
| DSN1 | Osteoporosis, Postmenopausal | 12.24 |
| FAM168A | Osteoporosis, Postmenopausal | 12.24 |
| IRF2BPL | Osteoporosis, Postmenopausal | 12.24 |
| PSTPIP2 | Osteoporosis, Postmenopausal | 12.24 |
| RALGAPA1 | Osteoporosis, Postmenopausal | 12.24 |
| LRATD2 | Osteoporosis, Postmenopausal | 12.23 |
| LRR1 | Osteoporosis, Postmenopausal | 12.23 |
| MICAL3 | Osteoporosis, Postmenopausal | 12.23 |
| CDK5RAP3 | Osteoporosis, Postmenopausal | 12.22 |
| COL9A2 | Osteoporosis, Postmenopausal | 12.22 |
| TMEM14A | Osteoporosis, Postmenopausal | 12.22 |
| TTR | Osteoporosis, Postmenopausal | 12.22 |
| ZMYM2 | Osteoporosis, Postmenopausal | 12.22 |
| ADGRD1 | Osteoporosis, Postmenopausal | 12.21 |
| CERK | Osteoporosis, Postmenopausal | 12.21 |
| ELOB | Osteoporosis, Postmenopausal | 12.21 |
| LHX6 | Osteoporosis, Postmenopausal | 12.21 |
| MIR141 | Osteoporosis, Postmenopausal | 12.21 |
| MKNK1 | Osteoporosis, Postmenopausal | 12.21 |
| TNNT3 | Osteoporosis, Postmenopausal | 12.21 |
| CENPQ | Osteoporosis, Postmenopausal | 12.2 |
| CES1C | Osteoporosis, Postmenopausal | 12.2 |
| FAM111B | Osteoporosis, Postmenopausal | 12.2 |
| FAM13B | Osteoporosis, Postmenopausal | 12.2 |
| FAM222A | Osteoporosis, Postmenopausal | 12.2 |
| FASTKD2 | Osteoporosis, Postmenopausal | 12.2 |
| GPD1L | Osteoporosis, Postmenopausal | 12.2 |
| IGSF1 | Osteoporosis, Postmenopausal | 12.2 |
| KCNS3 | Osteoporosis, Postmenopausal | 12.2 |
| MANEA | Osteoporosis, Postmenopausal | 12.2 |
| RAB2A | Osteoporosis, Postmenopausal | 12.2 |
| SLC38A6 | Osteoporosis, Postmenopausal | 12.2 |
| ABCB4 | Osteoporosis, Postmenopausal | 12.19 |
| PDCD5 | Osteoporosis, Postmenopausal | 12.19 |
| PHF14 | Osteoporosis, Postmenopausal | 12.19 |
| PTPA | Osteoporosis, Postmenopausal | 12.19 |
| EIF5A2 | Osteoporosis, Postmenopausal | 12.18 |
| MYL2 | Osteoporosis, Postmenopausal | 12.18 |
| C8G | Osteoporosis, Postmenopausal | 12.17 |
| CACNB3 | Osteoporosis, Postmenopausal | 12.17 |
| CHEK1 | Osteoporosis, Postmenopausal | 12.17 |
| DMXL1 | Osteoporosis, Postmenopausal | 12.17 |
| FAM110B | Osteoporosis, Postmenopausal | 12.17 |
| FGF13 | Osteoporosis, Postmenopausal | 12.17 |
| GSTP1 | Osteoporosis, Postmenopausal | 12.17 |
| HSF2 | Osteoporosis, Postmenopausal | 12.17 |
| IL17A | Osteoporosis, Postmenopausal | 12.17 |
| MAP4K2 | Osteoporosis, Postmenopausal | 12.17 |
| PCF11 | Osteoporosis, Postmenopausal | 12.17 |
| PRODH2 | Osteoporosis, Postmenopausal | 12.17 |
| REEP3 | Osteoporosis, Postmenopausal | 12.17 |
| SETD6 | Osteoporosis, Postmenopausal | 12.17 |
| TGIF2 | Osteoporosis, Postmenopausal | 12.17 |
| ERF | Osteoporosis, Postmenopausal | 12.16 |
| NOD2 | Osteoporosis, Postmenopausal | 12.16 |
| NOS1 | Osteoporosis, Postmenopausal | 12.16 |
| NRARP | Osteoporosis, Postmenopausal | 12.16 |
| RTN2 | Osteoporosis, Postmenopausal | 12.16 |
| VWA1 | Osteoporosis, Postmenopausal | 12.16 |
| XPO5 | Osteoporosis, Postmenopausal | 12.16 |
| HSP90AB5P | Osteoporosis, Postmenopausal | 12.15 |
| IL20RB | Osteoporosis, Postmenopausal | 12.15 |
| MIR4505 | Osteoporosis, Postmenopausal | 12.15 |
| RBBP7 | Osteoporosis, Postmenopausal | 12.15 |
| ADRB2 | Osteoporosis, Postmenopausal | 12.14 |
| AGO4 | Osteoporosis, Postmenopausal | 12.14 |
| F11 | Osteoporosis, Postmenopausal | 12.14 |
| PNOC | Osteoporosis, Postmenopausal | 12.14 |
| SMURF2 | Osteoporosis, Postmenopausal | 12.14 |
| UBTF | Osteoporosis, Postmenopausal | 12.14 |
| CBFA2T3 | Osteoporosis, Postmenopausal | 12.13 |
| CHD1L | Osteoporosis, Postmenopausal | 12.13 |
| FCMR | Osteoporosis, Postmenopausal | 12.13 |
| GLCE | Osteoporosis, Postmenopausal | 12.13 |
| KAT5 | Osteoporosis, Postmenopausal | 12.13 |
| MTMR4 | Osteoporosis, Postmenopausal | 12.13 |
| MYL12B | Osteoporosis, Postmenopausal | 12.13 |
| PARP8 | Osteoporosis, Postmenopausal | 12.13 |
| PCDH10 | Osteoporosis, Postmenopausal | 12.13 |
| PCDH8 | Osteoporosis, Postmenopausal | 12.13 |
| PSTPIP1 | Osteoporosis, Postmenopausal | 12.13 |
| SAC3D1 | Osteoporosis, Postmenopausal | 12.13 |
| SGPP2 | Osteoporosis, Postmenopausal | 12.13 |
| SH2D5 | Osteoporosis, Postmenopausal | 12.13 |
| SKA2 | Osteoporosis, Postmenopausal | 12.13 |
| SLC17A2 | Osteoporosis, Postmenopausal | 12.13 |
| TNKS | Osteoporosis, Postmenopausal | 12.13 |
| TRAPPC6A | Osteoporosis, Postmenopausal | 12.13 |
| UTP15 | Osteoporosis, Postmenopausal | 12.13 |
| ARHGEF28 | Osteoporosis, Postmenopausal | 12.12 |
| CRLS1 | Osteoporosis, Postmenopausal | 12.12 |
| CYP3A9 | Osteoporosis, Postmenopausal | 12.12 |
| NECTIN4 | Osteoporosis, Postmenopausal | 12.12 |
| CDK6 | Osteoporosis, Postmenopausal | 12.11 |
| ITPRIP | Osteoporosis, Postmenopausal | 12.11 |
| PELO | Osteoporosis, Postmenopausal | 12.11 |
| TLR8 | Osteoporosis, Postmenopausal | 12.11 |
| UBR2 | Osteoporosis, Postmenopausal | 12.11 |
| WDR62 | Osteoporosis, Postmenopausal | 12.11 |
| AATK | Osteoporosis, Postmenopausal | 12.1 |
| CTNND2 | Osteoporosis, Postmenopausal | 12.1 |
| EEIG2 | Osteoporosis, Postmenopausal | 12.1 |
| EYA2 | Osteoporosis, Postmenopausal | 12.1 |
| KRT20 | Osteoporosis, Postmenopausal | 12.1 |
| LMOD1 | Osteoporosis, Postmenopausal | 12.1 |
| MANSC1 | Osteoporosis, Postmenopausal | 12.1 |
| MTMR7 | Osteoporosis, Postmenopausal | 12.1 |
| MYOCD | Osteoporosis, Postmenopausal | 12.1 |
| NAB1 | Osteoporosis, Postmenopausal | 12.1 |
| NRAP | Osteoporosis, Postmenopausal | 12.1 |
| PPP1R18 | Osteoporosis, Postmenopausal | 12.1 |
| SEC16B | Osteoporosis, Postmenopausal | 12.1 |
| SLC6A14 | Osteoporosis, Postmenopausal | 12.1 |
| TMEM38B | Osteoporosis, Postmenopausal | 12.1 |
| TTF2 | Osteoporosis, Postmenopausal | 12.1 |
| ADAM15 | Osteoporosis, Postmenopausal | 12.09 |
| AGTPBP1 | Osteoporosis, Postmenopausal | 12.09 |
| B4GALT6 | Osteoporosis, Postmenopausal | 12.09 |
| CKLF | Osteoporosis, Postmenopausal | 12.09 |
| COL17A1 | Osteoporosis, Postmenopausal | 12.09 |
| DNA2 | Osteoporosis, Postmenopausal | 12.09 |
| KCNMB4 | Osteoporosis, Postmenopausal | 12.09 |
| KDM4A | Osteoporosis, Postmenopausal | 12.09 |
| MIR22HG | Osteoporosis, Postmenopausal | 12.09 |
| NKD2 | Osteoporosis, Postmenopausal | 12.09 |
| OLFML1 | Osteoporosis, Postmenopausal | 12.09 |
| PAK6 | Osteoporosis, Postmenopausal | 12.09 |
| PPP2R5A | Osteoporosis, Postmenopausal | 12.09 |
| SKA3 | Osteoporosis, Postmenopausal | 12.09 |
| SRPK2 | Osteoporosis, Postmenopausal | 12.09 |
| TTC9 | Osteoporosis, Postmenopausal | 12.09 |
| BTK | Osteoporosis, Postmenopausal | 12.08 |
| CDK8 | Osteoporosis, Postmenopausal | 12.08 |
| CHST3 | Osteoporosis, Postmenopausal | 12.08 |
| ANGPTL4 | Osteoporosis, Postmenopausal | 12.07 |
| AOPEP | Osteoporosis, Postmenopausal | 12.07 |
| ARID4A | Osteoporosis, Postmenopausal | 12.07 |
| RAB10 | Osteoporosis, Postmenopausal | 12.07 |
| CD3E | Osteoporosis, Postmenopausal | 12.06 |
| CLCN7 | Osteoporosis, Postmenopausal | 12.06 |
| CLEC11A | Osteoporosis, Postmenopausal | 12.06 |
| GDPD3 | Osteoporosis, Postmenopausal | 12.06 |
| HAS1 | Osteoporosis, Postmenopausal | 12.06 |
| MLF1 | Osteoporosis, Postmenopausal | 12.06 |
| MYH3 | Osteoporosis, Postmenopausal | 12.06 |
| OTUD1 | Osteoporosis, Postmenopausal | 12.06 |
| PBRM1 | Osteoporosis, Postmenopausal | 12.06 |
| PNPO | Osteoporosis, Postmenopausal | 12.06 |
| SCAF11 | Osteoporosis, Postmenopausal | 12.06 |
| SMYD3 | Osteoporosis, Postmenopausal | 12.06 |
| TDRD7 | Osteoporosis, Postmenopausal | 12.06 |
| TMEM50B | Osteoporosis, Postmenopausal | 12.06 |
| TRIM6 | Osteoporosis, Postmenopausal | 12.06 |
| TSPAN3 | Osteoporosis, Postmenopausal | 12.06 |
| CPEB3 | Osteoporosis, Postmenopausal | 12.05 |
| DMKN | Osteoporosis, Postmenopausal | 12.05 |
| JMY | Osteoporosis, Postmenopausal | 12.05 |
| PGM5 | Osteoporosis, Postmenopausal | 12.05 |
| HAUS4 | Osteoporosis, Postmenopausal | 12.04 |
| PGP | Osteoporosis, Postmenopausal | 12.04 |
| USP1 | Osteoporosis, Postmenopausal | 12.04 |
| ARFGEF1 | Osteoporosis, Postmenopausal | 12.03 |
| ERP27 | Osteoporosis, Postmenopausal | 12.03 |
| EYA4 | Osteoporosis, Postmenopausal | 12.03 |
| HOOK3 | Osteoporosis, Postmenopausal | 12.03 |
| LCP2 | Osteoporosis, Postmenopausal | 12.03 |
| PJA2 | Osteoporosis, Postmenopausal | 12.03 |
| STK17A | Osteoporosis, Postmenopausal | 12.03 |
| SURF4 | Osteoporosis, Postmenopausal | 12.03 |
| SYTL4 | Osteoporosis, Postmenopausal | 12.03 |
| TMEM135 | Osteoporosis, Postmenopausal | 12.03 |
| TRIM47 | Osteoporosis, Postmenopausal | 12.03 |
| WDFY1 | Osteoporosis, Postmenopausal | 12.03 |
| FAM110C | Osteoporosis, Postmenopausal | 12.02 |
| GIPC2 | Osteoporosis, Postmenopausal | 12.02 |
| GLRX2 | Osteoporosis, Postmenopausal | 12.02 |
| PPM1H | Osteoporosis, Postmenopausal | 12.02 |
| SERPINA5 | Osteoporosis, Postmenopausal | 12.02 |
| AEBP1 | Osteoporosis, Postmenopausal | 12.01 |
| ALPK1 | Osteoporosis, Postmenopausal | 12.01 |
| FCHSD2 | Osteoporosis, Postmenopausal | 12.01 |
| PSMD6 | Osteoporosis, Postmenopausal | 12 |
| PTPRR | Osteoporosis, Postmenopausal | 12 |
| CAND1 | Osteoporosis, Postmenopausal | 11.99 |
| CD5L | Osteoporosis, Postmenopausal | 11.99 |
| CLN3 | Osteoporosis, Postmenopausal | 11.99 |
| COPE | Osteoporosis, Postmenopausal | 11.99 |
| GABRP | Osteoporosis, Postmenopausal | 11.99 |
| GALNT15 | Osteoporosis, Postmenopausal | 11.99 |
| IGSF9 | Osteoporosis, Postmenopausal | 11.99 |
| LRIG3 | Osteoporosis, Postmenopausal | 11.99 |
| MALL | Osteoporosis, Postmenopausal | 11.99 |
| MMP | Osteoporosis, Postmenopausal | 11.99 |
| NMI | Osteoporosis, Postmenopausal | 11.99 |
| NUCB1 | Osteoporosis, Postmenopausal | 11.99 |
| PCP4 | Osteoporosis, Postmenopausal | 11.99 |
| PDS5B | Osteoporosis, Postmenopausal | 11.99 |
| PPP1R14C | Osteoporosis, Postmenopausal | 11.99 |
| ST3GAL4 | Osteoporosis, Postmenopausal | 11.99 |
| SUGCT | Osteoporosis, Postmenopausal | 11.99 |
| TCF7L1 | Osteoporosis, Postmenopausal | 11.99 |
| ADRA2C | Osteoporosis, Postmenopausal | 11.98 |
| CYSTM1 | Osteoporosis, Postmenopausal | 11.98 |
| SLC23A1 | Osteoporosis, Postmenopausal | 11.98 |
| TNK2 | Osteoporosis, Postmenopausal | 11.98 |
| UACA | Osteoporosis, Postmenopausal | 11.98 |
| COL10A1 | Osteoporosis, Postmenopausal | 11.97 |
| SHB | Osteoporosis, Postmenopausal | 11.97 |
| ALYREF | Osteoporosis, Postmenopausal | 11.96 |
| ARMCX3 | Osteoporosis, Postmenopausal | 11.96 |
| BMPR1B | Osteoporosis, Postmenopausal | 11.96 |
| CENPN | Osteoporosis, Postmenopausal | 11.96 |
| DMXL2 | Osteoporosis, Postmenopausal | 11.96 |
| FKBP10 | Osteoporosis, Postmenopausal | 11.96 |
| GOLM1 | Osteoporosis, Postmenopausal | 11.96 |
| HIRIP3 | Osteoporosis, Postmenopausal | 11.96 |
| IPO9 | Osteoporosis, Postmenopausal | 11.96 |
| NDUFA13 | Osteoporosis, Postmenopausal | 11.96 |
| NMNAT1 | Osteoporosis, Postmenopausal | 11.96 |
| PAN2 | Osteoporosis, Postmenopausal | 11.96 |
| PPP2R3A | Osteoporosis, Postmenopausal | 11.96 |
| SETD2 | Osteoporosis, Postmenopausal | 11.96 |
| SH3PXD2B | Osteoporosis, Postmenopausal | 11.96 |
| TAC1 | Osteoporosis, Postmenopausal | 11.96 |
| ACSS3 | Osteoporosis, Postmenopausal | 11.95 |
| ADM2 | Osteoporosis, Postmenopausal | 11.95 |
| AVIL | Osteoporosis, Postmenopausal | 11.95 |
| ISOC1 | Osteoporosis, Postmenopausal | 11.95 |
| SLC39A11 | Osteoporosis, Postmenopausal | 11.95 |
| SLIT1 | Osteoporosis, Postmenopausal | 11.95 |
| TNS2 | Osteoporosis, Postmenopausal | 11.95 |
| TOB2 | Osteoporosis, Postmenopausal | 11.95 |
| TRPM2 | Osteoporosis, Postmenopausal | 11.95 |
| TSC22D2 | Osteoporosis, Postmenopausal | 11.95 |
| FXYD2 | Osteoporosis, Postmenopausal | 11.94 |
| HBA-A1 | Osteoporosis, Postmenopausal | 11.94 |
| OLFML2A | Osteoporosis, Postmenopausal | 11.94 |
| RASGRF1 | Osteoporosis, Postmenopausal | 11.94 |
| SCGB1A1 | Osteoporosis, Postmenopausal | 11.94 |
| SFTPD | Osteoporosis, Postmenopausal | 11.94 |
| FIGN | Osteoporosis, Postmenopausal | 11.93 |
| KIF21A | Osteoporosis, Postmenopausal | 11.93 |
| LY6D | Osteoporosis, Postmenopausal | 11.93 |
| MASP2 | Osteoporosis, Postmenopausal | 11.93 |
| OGFR | Osteoporosis, Postmenopausal | 11.93 |
| PNPT1 | Osteoporosis, Postmenopausal | 11.93 |
| ZBTB21 | Osteoporosis, Postmenopausal | 11.93 |
| BBS2 | Osteoporosis, Postmenopausal | 11.92 |
| CD2AP | Osteoporosis, Postmenopausal | 11.92 |
| DCPS | Osteoporosis, Postmenopausal | 11.92 |
| DYNLT3 | Osteoporosis, Postmenopausal | 11.92 |
| KITL | Osteoporosis, Postmenopausal | 11.92 |
| LHFPL2 | Osteoporosis, Postmenopausal | 11.92 |
| MECR | Osteoporosis, Postmenopausal | 11.92 |
| PLOD1 | Osteoporosis, Postmenopausal | 11.92 |
| RNF128 | Osteoporosis, Postmenopausal | 11.92 |
| VDRA | Osteoporosis, Postmenopausal | 11.92 |
| CDC42BPB | Osteoporosis, Postmenopausal | 11.91 |
| CNBP | Osteoporosis, Postmenopausal | 11.91 |
| AHCYL1 | Osteoporosis, Postmenopausal | 11.9 |
| CPB2 | Osteoporosis, Postmenopausal | 11.9 |
| EDN3 | Osteoporosis, Postmenopausal | 11.9 |
| ARAF | Osteoporosis, Postmenopausal | 11.89 |
| BEX2 | Osteoporosis, Postmenopausal | 11.89 |
| DGKH | Osteoporosis, Postmenopausal | 11.89 |
| EVA1A | Osteoporosis, Postmenopausal | 11.89 |
| FBXO2 | Osteoporosis, Postmenopausal | 11.89 |
| GPSM1 | Osteoporosis, Postmenopausal | 11.89 |
| H4C8 | Osteoporosis, Postmenopausal | 11.89 |
| HINT3 | Osteoporosis, Postmenopausal | 11.89 |
| KDM5A | Osteoporosis, Postmenopausal | 11.89 |
| MBD4 | Osteoporosis, Postmenopausal | 11.89 |
| MYD88 | Osteoporosis, Postmenopausal | 11.89 |
| MYO18A | Osteoporosis, Postmenopausal | 11.89 |
| PFKFB2 | Osteoporosis, Postmenopausal | 11.89 |
| PNKD | Osteoporosis, Postmenopausal | 11.89 |
| RBM15 | Osteoporosis, Postmenopausal | 11.89 |
| RTN4R | Osteoporosis, Postmenopausal | 11.89 |
| SLC15A3 | Osteoporosis, Postmenopausal | 11.89 |
| SOX8 | Osteoporosis, Postmenopausal | 11.89 |
| SUV39H2 | Osteoporosis, Postmenopausal | 11.89 |
| THYN1 | Osteoporosis, Postmenopausal | 11.89 |
| ZC3H6 | Osteoporosis, Postmenopausal | 11.89 |
| MIS18BP1 | Osteoporosis, Postmenopausal | 11.88 |
| S100A13 | Osteoporosis, Postmenopausal | 11.88 |
| CERS6 | Osteoporosis, Postmenopausal | 11.87 |
| INPP5A | Osteoporosis, Postmenopausal | 11.87 |
| NTNG2A | Osteoporosis, Postmenopausal | 11.87 |
| BCKDK | Osteoporosis, Postmenopausal | 11.86 |
| CDK5RAP2 | Osteoporosis, Postmenopausal | 11.86 |
| CENPH | Osteoporosis, Postmenopausal | 11.86 |
| CYP2A4 | Osteoporosis, Postmenopausal | 11.86 |
| EPHB4 | Osteoporosis, Postmenopausal | 11.86 |
| FXYD3 | Osteoporosis, Postmenopausal | 11.86 |
| NUP58 | Osteoporosis, Postmenopausal | 11.86 |
| PAK3 | Osteoporosis, Postmenopausal | 11.86 |
| RFFL | Osteoporosis, Postmenopausal | 11.86 |
| SLC22A12 | Osteoporosis, Postmenopausal | 11.86 |
| SOX6 | Osteoporosis, Postmenopausal | 11.86 |
| SRD5A3 | Osteoporosis, Postmenopausal | 11.86 |
| TP53INP2 | Osteoporosis, Postmenopausal | 11.86 |
| CCN4 | Osteoporosis, Postmenopausal | 11.85 |
| SLC4A11 | Osteoporosis, Postmenopausal | 11.85 |
| CBX1 | Osteoporosis, Postmenopausal | 11.84 |
| MAP3K20 | Osteoporosis, Postmenopausal | 11.84 |
| MST1 | Osteoporosis, Postmenopausal | 11.84 |
| PPM1K | Osteoporosis, Postmenopausal | 11.84 |
| SEMA4B | Osteoporosis, Postmenopausal | 11.84 |
| SRXN1 | Osteoporosis, Postmenopausal | 11.84 |
| ABHD11 | Osteoporosis, Postmenopausal | 11.83 |
| ABHD14B | Osteoporosis, Postmenopausal | 11.83 |
| ACOX1 | Osteoporosis, Postmenopausal | 11.83 |
| AMPD2 | Osteoporosis, Postmenopausal | 11.83 |
| ANKH | Osteoporosis, Postmenopausal | 11.83 |
| APLP1 | Osteoporosis, Postmenopausal | 11.83 |
| ATXN2L | Osteoporosis, Postmenopausal | 11.83 |
| CCNC | Osteoporosis, Postmenopausal | 11.83 |
| COX6A1 | Osteoporosis, Postmenopausal | 11.83 |
| FZD5 | Osteoporosis, Postmenopausal | 11.83 |
| GINS3 | Osteoporosis, Postmenopausal | 11.83 |
| KCNJ5 | Osteoporosis, Postmenopausal | 11.83 |
| LACTB | Osteoporosis, Postmenopausal | 11.83 |
| MICALL1 | Osteoporosis, Postmenopausal | 11.83 |
| NDUFA3 | Osteoporosis, Postmenopausal | 11.83 |
| PKDCC | Osteoporosis, Postmenopausal | 11.83 |
| POU3F1 | Osteoporosis, Postmenopausal | 11.83 |
| PPP1R13L | Osteoporosis, Postmenopausal | 11.83 |
| SF3A2 | Osteoporosis, Postmenopausal | 11.83 |
| SP100 | Osteoporosis, Postmenopausal | 11.83 |
| TBC1D1 | Osteoporosis, Postmenopausal | 11.83 |
| TMEM176B | Osteoporosis, Postmenopausal | 11.83 |
| TTC39C | Osteoporosis, Postmenopausal | 11.83 |
| ACIN1 | Osteoporosis, Postmenopausal | 11.82 |
| MARCO | Osteoporosis, Postmenopausal | 11.82 |
| RCL1 | Osteoporosis, Postmenopausal | 11.82 |
| ADAM22 | Osteoporosis, Postmenopausal | 11.81 |
| EVI2A | Osteoporosis, Postmenopausal | 11.81 |
| PTP4A3 | Osteoporosis, Postmenopausal | 11.81 |
| ACAN | Osteoporosis, Postmenopausal | 11.8 |
| AIFM1 | Osteoporosis, Postmenopausal | 11.8 |
| CLYBL | Osteoporosis, Postmenopausal | 11.8 |
| DOCK5 | Osteoporosis, Postmenopausal | 11.8 |
| EHF | Osteoporosis, Postmenopausal | 11.8 |
| HNRNPH3 | Osteoporosis, Postmenopausal | 11.8 |
| HOXB9 | Osteoporosis, Postmenopausal | 11.8 |
| KPNA3 | Osteoporosis, Postmenopausal | 11.8 |
| MBD2 | Osteoporosis, Postmenopausal | 11.8 |
| MNS1 | Osteoporosis, Postmenopausal | 11.8 |
| MPZ | Osteoporosis, Postmenopausal | 11.8 |
| NME7 | Osteoporosis, Postmenopausal | 11.8 |
| NPNT | Osteoporosis, Postmenopausal | 11.8 |
| PAK1IP1 | Osteoporosis, Postmenopausal | 11.8 |
| PIPOX | Osteoporosis, Postmenopausal | 11.8 |
| REV3L | Osteoporosis, Postmenopausal | 11.8 |
| RNASET2 | Osteoporosis, Postmenopausal | 11.8 |
| SAMD9 | Osteoporosis, Postmenopausal | 11.8 |
| SRPRB | Osteoporosis, Postmenopausal | 11.8 |
| TYK2 | Osteoporosis, Postmenopausal | 11.8 |
| UBE2L3 | Osteoporosis, Postmenopausal | 11.8 |
| VIL1 | Osteoporosis, Postmenopausal | 11.8 |
| VPS13A | Osteoporosis, Postmenopausal | 11.8 |
| GNA15 | Osteoporosis, Postmenopausal | 11.79 |
| MKLN1 | Osteoporosis, Postmenopausal | 11.79 |
| PKD2 | Osteoporosis, Postmenopausal | 11.79 |
| TLE1 | Osteoporosis, Postmenopausal | 11.79 |
| ADAMTS7 | Osteoporosis, Postmenopausal | 11.77 |
| CDC42EP1 | Osteoporosis, Postmenopausal | 11.77 |
| COX3 | Osteoporosis, Postmenopausal | 11.77 |
| CYB561 | Osteoporosis, Postmenopausal | 11.77 |
| NUMB | Osteoporosis, Postmenopausal | 11.77 |
| PLBD1 | Osteoporosis, Postmenopausal | 11.77 |
| RAB11FIP1 | Osteoporosis, Postmenopausal | 11.77 |
| RNF4 | Osteoporosis, Postmenopausal | 11.77 |
| SOX11 | Osteoporosis, Postmenopausal | 11.77 |
| TCF21 | Osteoporosis, Postmenopausal | 11.77 |
| WDR3 | Osteoporosis, Postmenopausal | 11.77 |
| ASCC3 | Osteoporosis, Postmenopausal | 11.76 |
| ATF1 | Osteoporosis, Postmenopausal | 11.76 |
| CASR | Osteoporosis, Postmenopausal | 11.76 |
| CELSR3 | Osteoporosis, Postmenopausal | 11.76 |
| CLSTN1 | Osteoporosis, Postmenopausal | 11.76 |
| LSM4 | Osteoporosis, Postmenopausal | 11.76 |
| MXRA8 | Osteoporosis, Postmenopausal | 11.76 |
| PARP12 | Osteoporosis, Postmenopausal | 11.76 |
| PCYT1A | Osteoporosis, Postmenopausal | 11.76 |
| SLC66A3 | Osteoporosis, Postmenopausal | 11.76 |
| TNFRSF10D | Osteoporosis, Postmenopausal | 11.75 |
| CSNK2B | Osteoporosis, Postmenopausal | 11.74 |
| CUX2 | Osteoporosis, Postmenopausal | 11.74 |
| CXCL15 | Osteoporosis, Postmenopausal | 11.74 |
| GAB2 | Osteoporosis, Postmenopausal | 11.74 |
| JAG2 | Osteoporosis, Postmenopausal | 11.74 |
| KANK4 | Osteoporosis, Postmenopausal | 11.74 |
| KRTAP5-7 | Osteoporosis, Postmenopausal | 11.74 |
| MADD | Osteoporosis, Postmenopausal | 11.74 |
| MIR518F | Osteoporosis, Postmenopausal | 11.74 |
| MYCL | Osteoporosis, Postmenopausal | 11.74 |
| RASL11A | Osteoporosis, Postmenopausal | 11.74 |
| SFRP4 | Osteoporosis, Postmenopausal | 11.74 |
| TIMM9 | Osteoporosis, Postmenopausal | 11.74 |
| TNFRSF14 | Osteoporosis, Postmenopausal | 11.74 |
| WNT10B | Osteoporosis, Postmenopausal | 11.74 |
| ACOT13 | Osteoporosis, Postmenopausal | 11.73 |
| ENTPD2 | Osteoporosis, Postmenopausal | 11.73 |
| FANCG | Osteoporosis, Postmenopausal | 11.73 |
| FMNL2 | Osteoporosis, Postmenopausal | 11.73 |
| GCNT1 | Osteoporosis, Postmenopausal | 11.73 |
| ME2 | Osteoporosis, Postmenopausal | 11.73 |
| NADK2 | Osteoporosis, Postmenopausal | 11.73 |
| NFATC4 | Osteoporosis, Postmenopausal | 11.73 |
| RECQL | Osteoporosis, Postmenopausal | 11.73 |
| SBK1 | Osteoporosis, Postmenopausal | 11.73 |
| SPN | Osteoporosis, Postmenopausal | 11.73 |
| UROD | Osteoporosis, Postmenopausal | 11.73 |
| HEPH | Osteoporosis, Postmenopausal | 11.72 |
| MYEF2 | Osteoporosis, Postmenopausal | 11.72 |
| ANXA8 | Osteoporosis, Postmenopausal | 11.71 |
| CD3D | Osteoporosis, Postmenopausal | 11.71 |
| CDCP1 | Osteoporosis, Postmenopausal | 11.71 |
| CLCA1 | Osteoporosis, Postmenopausal | 11.71 |
| CSF3R | Osteoporosis, Postmenopausal | 11.71 |
| INSL3 | Osteoporosis, Postmenopausal | 11.71 |
| NLN | Osteoporosis, Postmenopausal | 11.71 |
| NRXN3 | Osteoporosis, Postmenopausal | 11.71 |
| PLEKHA6 | Osteoporosis, Postmenopausal | 11.71 |
| POLR3G | Osteoporosis, Postmenopausal | 11.71 |
| PPAN | Osteoporosis, Postmenopausal | 11.71 |
| RAMP2 | Osteoporosis, Postmenopausal | 11.71 |
| RAPGEF3 | Osteoporosis, Postmenopausal | 11.71 |
| SLC28A2 | Osteoporosis, Postmenopausal | 11.71 |
| TSPAN5 | Osteoporosis, Postmenopausal | 11.71 |
| VAV1 | Osteoporosis, Postmenopausal | 11.71 |
| ASNS | Osteoporosis, Postmenopausal | 11.7 |
| DLX2 | Osteoporosis, Postmenopausal | 11.7 |
| MAP3K4 | Osteoporosis, Postmenopausal | 11.7 |
| PPP1R1A | Osteoporosis, Postmenopausal | 11.7 |
| TIFA | Osteoporosis, Postmenopausal | 11.7 |
| TSHZ1 | Osteoporosis, Postmenopausal | 11.7 |
| SLCO4C1 | Osteoporosis, Postmenopausal | 11.69 |
| TRIB3 | Osteoporosis, Postmenopausal | 11.69 |
| CDS1 | Osteoporosis, Postmenopausal | 11.68 |
| ETNPPL | Osteoporosis, Postmenopausal | 11.68 |
| OSR2 | Osteoporosis, Postmenopausal | 11.68 |
| PLEKHA2 | Osteoporosis, Postmenopausal | 11.68 |
| POLD4 | Osteoporosis, Postmenopausal | 11.68 |
| RBP7 | Osteoporosis, Postmenopausal | 11.68 |
| THOC2 | Osteoporosis, Postmenopausal | 11.68 |
| UBAP2L | Osteoporosis, Postmenopausal | 11.68 |
| ATF7IP | Osteoporosis, Postmenopausal | 11.67 |
| CASP8AP2 | Osteoporosis, Postmenopausal | 11.67 |
| CD28 | Osteoporosis, Postmenopausal | 11.67 |
| FAM98A | Osteoporosis, Postmenopausal | 11.67 |
| GPRC5C | Osteoporosis, Postmenopausal | 11.67 |
| HPN | Osteoporosis, Postmenopausal | 11.67 |
| MND1 | Osteoporosis, Postmenopausal | 11.67 |
| NBL1 | Osteoporosis, Postmenopausal | 11.67 |
| NME4 | Osteoporosis, Postmenopausal | 11.67 |
| NTRK3 | Osteoporosis, Postmenopausal | 11.67 |
| PLOD3 | Osteoporosis, Postmenopausal | 11.67 |
| TNKS1BP1 | Osteoporosis, Postmenopausal | 11.67 |
| ADCYAP1 | Osteoporosis, Postmenopausal | 11.66 |
| PAPOLA | Osteoporosis, Postmenopausal | 11.66 |
| ARF4 | Osteoporosis, Postmenopausal | 11.65 |
| PCOLCE2 | Osteoporosis, Postmenopausal | 11.65 |
| PDCD11 | Osteoporosis, Postmenopausal | 11.65 |
| PIK3AP1 | Osteoporosis, Postmenopausal | 11.65 |
| PTPRZ1 | Osteoporosis, Postmenopausal | 11.65 |
| SELPLG | Osteoporosis, Postmenopausal | 11.65 |
| SLC25A22 | Osteoporosis, Postmenopausal | 11.65 |
| TMEM176A | Osteoporosis, Postmenopausal | 11.65 |
| TSPAN12 | Osteoporosis, Postmenopausal | 11.65 |
| ALS2 | Osteoporosis, Postmenopausal | 11.64 |
| CCDC92 | Osteoporosis, Postmenopausal | 11.64 |
| DNAJC9 | Osteoporosis, Postmenopausal | 11.64 |
| GRHL1 | Osteoporosis, Postmenopausal | 11.64 |
| HECTD1 | Osteoporosis, Postmenopausal | 11.64 |
| KHSRP | Osteoporosis, Postmenopausal | 11.64 |
| LOXL4 | Osteoporosis, Postmenopausal | 11.64 |
| NDE1 | Osteoporosis, Postmenopausal | 11.64 |
| OXR1 | Osteoporosis, Postmenopausal | 11.64 |
| RHOBTB3 | Osteoporosis, Postmenopausal | 11.64 |
| AP1S2 | Osteoporosis, Postmenopausal | 11.62 |
| C8A | Osteoporosis, Postmenopausal | 11.62 |
| PKP4 | Osteoporosis, Postmenopausal | 11.62 |
| PLCD1 | Osteoporosis, Postmenopausal | 11.62 |
| PRKCG | Osteoporosis, Postmenopausal | 11.62 |
| CLPX | Osteoporosis, Postmenopausal | 11.61 |
| DARS1 | Osteoporosis, Postmenopausal | 11.61 |
| ERN1 | Osteoporosis, Postmenopausal | 11.61 |
| LAT2 | Osteoporosis, Postmenopausal | 11.61 |
| MALT1 | Osteoporosis, Postmenopausal | 11.61 |
| NDUFA2 | Osteoporosis, Postmenopausal | 11.61 |
| NUAK1 | Osteoporosis, Postmenopausal | 11.61 |
| PDP1 | Osteoporosis, Postmenopausal | 11.61 |
| PDX1 | Osteoporosis, Postmenopausal | 11.61 |
| PTPN13 | Osteoporosis, Postmenopausal | 11.61 |
| SLC39A6 | Osteoporosis, Postmenopausal | 11.61 |
| STX1A | Osteoporosis, Postmenopausal | 11.61 |
| TRIM63 | Osteoporosis, Postmenopausal | 11.61 |
| XRCC3 | Osteoporosis, Postmenopausal | 11.61 |
| CES2A | Osteoporosis, Postmenopausal | 11.59 |
| CHGB | Osteoporosis, Postmenopausal | 11.59 |
| DAB1 | Osteoporosis, Postmenopausal | 11.59 |
| DTX4 | Osteoporosis, Postmenopausal | 11.59 |
| EIF4G2 | Osteoporosis, Postmenopausal | 11.59 |
| EXOSC2 | Osteoporosis, Postmenopausal | 11.59 |
| GRIN2D | Osteoporosis, Postmenopausal | 11.59 |
| KCNJ11 | Osteoporosis, Postmenopausal | 11.59 |
| KRT16 | Osteoporosis, Postmenopausal | 11.59 |
| MDN1 | Osteoporosis, Postmenopausal | 11.59 |
| NHP2 | Osteoporosis, Postmenopausal | 11.59 |
| PAMR1 | Osteoporosis, Postmenopausal | 11.59 |
| PAX8 | Osteoporosis, Postmenopausal | 11.59 |
| RAPGEF2 | Osteoporosis, Postmenopausal | 11.59 |
| SH3GLB1 | Osteoporosis, Postmenopausal | 11.59 |
| SLC3A1 | Osteoporosis, Postmenopausal | 11.59 |
| STAMBPL1 | Osteoporosis, Postmenopausal | 11.59 |
| SULT1A1 | Osteoporosis, Postmenopausal | 11.59 |
| FABP6 | Osteoporosis, Postmenopausal | 11.58 |
| GSTP2 | Osteoporosis, Postmenopausal | 11.58 |
| MMP11 | Osteoporosis, Postmenopausal | 11.58 |
| PCK1 | Osteoporosis, Postmenopausal | 11.58 |
| PLEK | Osteoporosis, Postmenopausal | 11.58 |
| TIGAR | Osteoporosis, Postmenopausal | 11.58 |
| ZCCHC7 | Osteoporosis, Postmenopausal | 11.58 |
| SF3B1 | Osteoporosis, Postmenopausal | 11.57 |
| ABI1 | Osteoporosis, Postmenopausal | 11.56 |
| ADAMTS2 | Osteoporosis, Postmenopausal | 11.56 |
| CYORF15A | Osteoporosis, Postmenopausal | 11.56 |
| GASK1B | Osteoporosis, Postmenopausal | 11.56 |
| GDF10 | Osteoporosis, Postmenopausal | 11.56 |
| HDAC7 | Osteoporosis, Postmenopausal | 11.56 |
| HJURP | Osteoporosis, Postmenopausal | 11.56 |
| IFRD2 | Osteoporosis, Postmenopausal | 11.56 |
| KCNN2 | Osteoporosis, Postmenopausal | 11.56 |
| LAD1 | Osteoporosis, Postmenopausal | 11.56 |
| LGALS2 | Osteoporosis, Postmenopausal | 11.56 |
| PHF20L1 | Osteoporosis, Postmenopausal | 11.56 |
| PRR5L | Osteoporosis, Postmenopausal | 11.56 |
| PRRG4 | Osteoporosis, Postmenopausal | 11.56 |
| SDS | Osteoporosis, Postmenopausal | 11.56 |
| SLC13A5 | Osteoporosis, Postmenopausal | 11.56 |
| SNTB1 | Osteoporosis, Postmenopausal | 11.56 |
| TNNT1 | Osteoporosis, Postmenopausal | 11.56 |
| TPBG | Osteoporosis, Postmenopausal | 11.56 |
| TNFRSF1A | Osteoporosis, Postmenopausal | 11.55 |
| ARHGAP29 | Osteoporosis, Postmenopausal | 11.53 |
| ATL2 | Osteoporosis, Postmenopausal | 11.53 |
| BCR | Osteoporosis, Postmenopausal | 11.53 |
| FLRT2 | Osteoporosis, Postmenopausal | 11.53 |
| GLA | Osteoporosis, Postmenopausal | 11.53 |
| GLIPR2 | Osteoporosis, Postmenopausal | 11.53 |
| HMGCL | Osteoporosis, Postmenopausal | 11.53 |
| KDM7A | Osteoporosis, Postmenopausal | 11.53 |
| LYAR | Osteoporosis, Postmenopausal | 11.53 |
| MICAL1 | Osteoporosis, Postmenopausal | 11.53 |
| PUS1 | Osteoporosis, Postmenopausal | 11.53 |
| RBFOX3 | Osteoporosis, Postmenopausal | 11.53 |
| S100A1 | Osteoporosis, Postmenopausal | 11.53 |
| SMIM14 | Osteoporosis, Postmenopausal | 11.53 |
| SNRPA | Osteoporosis, Postmenopausal | 11.53 |
| SSBP2 | Osteoporosis, Postmenopausal | 11.53 |
| STAU2 | Osteoporosis, Postmenopausal | 11.53 |
| TNNC2 | Osteoporosis, Postmenopausal | 11.53 |
| TOPBP1 | Osteoporosis, Postmenopausal | 11.53 |
| DCAKD | Osteoporosis, Postmenopausal | 11.51 |
| EPPK1 | Osteoporosis, Postmenopausal | 11.51 |
| MSX2 | Osteoporosis, Postmenopausal | 11.51 |
| AMOTL2 | Osteoporosis, Postmenopausal | 11.5 |
| ANKRD10 | Osteoporosis, Postmenopausal | 11.5 |
| CHRNA5 | Osteoporosis, Postmenopausal | 11.5 |
| EPHA3 | Osteoporosis, Postmenopausal | 11.5 |
| FOXC2 | Osteoporosis, Postmenopausal | 11.5 |
| NDUFA4 | Osteoporosis, Postmenopausal | 11.5 |
| PDP2 | Osteoporosis, Postmenopausal | 11.5 |
| PGM2 | Osteoporosis, Postmenopausal | 11.5 |
| PPFIBP2 | Osteoporosis, Postmenopausal | 11.5 |
| PPID | Osteoporosis, Postmenopausal | 11.5 |
| RCAN2 | Osteoporosis, Postmenopausal | 11.5 |
| RFC2 | Osteoporosis, Postmenopausal | 11.5 |
| SEC62 | Osteoporosis, Postmenopausal | 11.5 |
| SLFN5 | Osteoporosis, Postmenopausal | 11.5 |
| SMAD5 | Osteoporosis, Postmenopausal | 11.5 |
| SRGAP3 | Osteoporosis, Postmenopausal | 11.5 |
| TNNI2 | Osteoporosis, Postmenopausal | 11.5 |
| GSTA2 | Osteoporosis, Postmenopausal | 11.49 |
| AIG1 | Osteoporosis, Postmenopausal | 11.48 |
| EAF2 | Osteoporosis, Postmenopausal | 11.48 |
| PDK3 | Osteoporosis, Postmenopausal | 11.48 |
| RASL11B | Osteoporosis, Postmenopausal | 11.48 |
| SPARCL1 | Osteoporosis, Postmenopausal | 11.48 |
| CREB3L2 | Osteoporosis, Postmenopausal | 11.47 |
| EYA1 | Osteoporosis, Postmenopausal | 11.47 |
| FGR | Osteoporosis, Postmenopausal | 11.47 |
| PSMC3IP | Osteoporosis, Postmenopausal | 11.47 |
| RAB20 | Osteoporosis, Postmenopausal | 11.47 |
| SAA2 | Osteoporosis, Postmenopausal | 11.47 |
| STRBP | Osteoporosis, Postmenopausal | 11.47 |
| TAP2 | Osteoporosis, Postmenopausal | 11.47 |
| WNT11 | Osteoporosis, Postmenopausal | 11.47 |
| XRCC2 | Osteoporosis, Postmenopausal | 11.47 |
| DPYSL4 | Osteoporosis, Postmenopausal | 11.46 |
| FLT4 | Osteoporosis, Postmenopausal | 11.46 |
| P2RY1 | Osteoporosis, Postmenopausal | 11.46 |
| PCOLCE | Osteoporosis, Postmenopausal | 11.46 |
| ADCY9 | Osteoporosis, Postmenopausal | 11.45 |
| APOA5 | Osteoporosis, Postmenopausal | 11.45 |
| CENPM | Osteoporosis, Postmenopausal | 11.45 |
| DNER | Osteoporosis, Postmenopausal | 11.45 |
| FXR1 | Osteoporosis, Postmenopausal | 11.45 |
| GJA4 | Osteoporosis, Postmenopausal | 11.45 |
| GPR137B | Osteoporosis, Postmenopausal | 11.45 |
| MAGI1 | Osteoporosis, Postmenopausal | 11.45 |
| MYO1D | Osteoporosis, Postmenopausal | 11.45 |
| PAQR9 | Osteoporosis, Postmenopausal | 11.45 |
| TRIM16 | Osteoporosis, Postmenopausal | 11.45 |
| UGT2B17 | Osteoporosis, Postmenopausal | 11.45 |
| ANGPTL2 | Osteoporosis, Postmenopausal | 11.44 |
| EGR4 | Osteoporosis, Postmenopausal | 11.44 |
| FAM107A | Osteoporosis, Postmenopausal | 11.44 |
| NOP2 | Osteoporosis, Postmenopausal | 11.44 |
| PPA2 | Osteoporosis, Postmenopausal | 11.44 |
| RAB8B | Osteoporosis, Postmenopausal | 11.44 |
| SACS | Osteoporosis, Postmenopausal | 11.44 |
| CAMK1D | Osteoporosis, Postmenopausal | 11.43 |
| GAPDH | Osteoporosis, Postmenopausal | 11.43 |
| SP8B | Osteoporosis, Postmenopausal | 11.43 |
| CACNA1H | Osteoporosis, Postmenopausal | 11.42 |
| DUOX2 | Osteoporosis, Postmenopausal | 11.42 |
| FGD4 | Osteoporosis, Postmenopausal | 11.42 |
| FOXO4 | Osteoporosis, Postmenopausal | 11.42 |
| FUT8 | Osteoporosis, Postmenopausal | 11.42 |
| GPM6B | Osteoporosis, Postmenopausal | 11.42 |
| H2BC21 | Osteoporosis, Postmenopausal | 11.42 |
| MOCOS | Osteoporosis, Postmenopausal | 11.42 |
| NEIL3 | Osteoporosis, Postmenopausal | 11.42 |
| NMT2 | Osteoporosis, Postmenopausal | 11.42 |
| PHF19 | Osteoporosis, Postmenopausal | 11.42 |
| RGL1 | Osteoporosis, Postmenopausal | 11.42 |
| RPLP2 | Osteoporosis, Postmenopausal | 11.42 |
| SLC46A1 | Osteoporosis, Postmenopausal | 11.42 |
| SRPRA | Osteoporosis, Postmenopausal | 11.42 |
| AKR7A2P1 | Osteoporosis, Postmenopausal | 11.41 |
| BMP1 | Osteoporosis, Postmenopausal | 11.41 |
| DLL4 | Osteoporosis, Postmenopausal | 11.41 |
| GFOD1 | Osteoporosis, Postmenopausal | 11.41 |
| MIR518C | Osteoporosis, Postmenopausal | 11.41 |
| PROX1 | Osteoporosis, Postmenopausal | 11.41 |
| TFR2 | Osteoporosis, Postmenopausal | 11.41 |
| TLE3 | Osteoporosis, Postmenopausal | 11.41 |
| TUFM | Osteoporosis, Postmenopausal | 11.41 |
| ANK1 | Osteoporosis, Postmenopausal | 11.4 |
| DAP | Osteoporosis, Postmenopausal | 11.4 |
| LGR4 | Osteoporosis, Postmenopausal | 11.4 |
| SCARA3 | Osteoporosis, Postmenopausal | 11.4 |
| SCNN1B | Osteoporosis, Postmenopausal | 11.4 |
| SIRPA | Osteoporosis, Postmenopausal | 11.4 |
| ACY1 | Osteoporosis, Postmenopausal | 11.39 |
| CBX7 | Osteoporosis, Postmenopausal | 11.39 |
| CD8A | Osteoporosis, Postmenopausal | 11.39 |
| CHCHD10 | Osteoporosis, Postmenopausal | 11.39 |
| DSG2 | Osteoporosis, Postmenopausal | 11.39 |
| GRINA | Osteoporosis, Postmenopausal | 11.39 |
| IPO7 | Osteoporosis, Postmenopausal | 11.39 |
| MFAP2 | Osteoporosis, Postmenopausal | 11.39 |
| MID1 | Osteoporosis, Postmenopausal | 11.39 |
| MRPS6 | Osteoporosis, Postmenopausal | 11.39 |
| QDPR | Osteoporosis, Postmenopausal | 11.39 |
| QKI | Osteoporosis, Postmenopausal | 11.39 |
| RAD21 | Osteoporosis, Postmenopausal | 11.39 |
| RNF213 | Osteoporosis, Postmenopausal | 11.39 |
| TREM2 | Osteoporosis, Postmenopausal | 11.39 |
| AGL | Osteoporosis, Postmenopausal | 11.37 |
| BAZ1A | Osteoporosis, Postmenopausal | 11.37 |
| EEPD1 | Osteoporosis, Postmenopausal | 11.37 |
| FETUB | Osteoporosis, Postmenopausal | 11.37 |
| MPRIP | Osteoporosis, Postmenopausal | 11.37 |
| PELI1 | Osteoporosis, Postmenopausal | 11.37 |
| PYCR1 | Osteoporosis, Postmenopausal | 11.37 |
| TCF12 | Osteoporosis, Postmenopausal | 11.37 |
| TRAF4 | Osteoporosis, Postmenopausal | 11.37 |
| ACTN2 | Osteoporosis, Postmenopausal | 11.36 |
| ADAMTS4 | Osteoporosis, Postmenopausal | 11.36 |
| BZW1 | Osteoporosis, Postmenopausal | 11.36 |
| CPD | Osteoporosis, Postmenopausal | 11.36 |
| DAXX | Osteoporosis, Postmenopausal | 11.36 |
| EPB41L2 | Osteoporosis, Postmenopausal | 11.36 |
| GRK3 | Osteoporosis, Postmenopausal | 11.36 |
| HEATR1 | Osteoporosis, Postmenopausal | 11.36 |
| MMUT | Osteoporosis, Postmenopausal | 11.36 |
| NIFK | Osteoporosis, Postmenopausal | 11.36 |
| OSBPL3 | Osteoporosis, Postmenopausal | 11.36 |
| PAK2 | Osteoporosis, Postmenopausal | 11.36 |
| PSPC1 | Osteoporosis, Postmenopausal | 11.36 |
| RAD54B | Osteoporosis, Postmenopausal | 11.36 |
| SLC17A5 | Osteoporosis, Postmenopausal | 11.36 |
| SOBP | Osteoporosis, Postmenopausal | 11.36 |
| SP3 | Osteoporosis, Postmenopausal | 11.36 |
| SRI | Osteoporosis, Postmenopausal | 11.36 |
| CITED4 | Osteoporosis, Postmenopausal | 11.35 |
| CLCN3 | Osteoporosis, Postmenopausal | 11.35 |
| SPRR1A | Osteoporosis, Postmenopausal | 11.35 |
| COBLL1 | Osteoporosis, Postmenopausal | 11.34 |
| LGALS9 | Osteoporosis, Postmenopausal | 11.34 |
| MCCC2 | Osteoporosis, Postmenopausal | 11.34 |
| NFE2 | Osteoporosis, Postmenopausal | 11.34 |
| SEMA3A | Osteoporosis, Postmenopausal | 11.34 |
| SLC15A1 | Osteoporosis, Postmenopausal | 11.34 |
| SON | Osteoporosis, Postmenopausal | 11.34 |
| SSR3 | Osteoporosis, Postmenopausal | 11.34 |
| ST8SIA4 | Osteoporosis, Postmenopausal | 11.34 |
| SYTL2 | Osteoporosis, Postmenopausal | 11.34 |
| TACC1 | Osteoporosis, Postmenopausal | 11.34 |
| TRIM24 | Osteoporosis, Postmenopausal | 11.34 |
| ZMIZ1 | Osteoporosis, Postmenopausal | 11.34 |
| ADCY5 | Osteoporosis, Postmenopausal | 11.33 |
| FH | Osteoporosis, Postmenopausal | 11.33 |
| GALNT10 | Osteoporosis, Postmenopausal | 11.33 |
| GRM1 | Osteoporosis, Postmenopausal | 11.33 |
| PLXNB2 | Osteoporosis, Postmenopausal | 11.33 |
| PPP3R1 | Osteoporosis, Postmenopausal | 11.33 |
| PSMD14 | Osteoporosis, Postmenopausal | 11.33 |
| SMTN | Osteoporosis, Postmenopausal | 11.33 |
| TRPV2 | Osteoporosis, Postmenopausal | 11.33 |
| CLSPN | Osteoporosis, Postmenopausal | 11.32 |
| FRAS1 | Osteoporosis, Postmenopausal | 11.32 |
| FZD1 | Osteoporosis, Postmenopausal | 11.32 |
| MAD1L1 | Osteoporosis, Postmenopausal | 11.32 |
| MUC2 | Osteoporosis, Postmenopausal | 11.32 |
| NEXN | Osteoporosis, Postmenopausal | 11.32 |
| PLD3 | Osteoporosis, Postmenopausal | 11.32 |
| SLC15A2 | Osteoporosis, Postmenopausal | 11.32 |
| DSTN | Osteoporosis, Postmenopausal | 11.31 |
| MIR155 | Osteoporosis, Postmenopausal | 11.31 |
| NTN4 | Osteoporosis, Postmenopausal | 11.31 |
| RASA1 | Osteoporosis, Postmenopausal | 11.31 |
| SLC17A7 | Osteoporosis, Postmenopausal | 11.31 |
| TNFSF14 | Osteoporosis, Postmenopausal | 11.31 |
| TSPAN8 | Osteoporosis, Postmenopausal | 11.31 |
| ATOSA | Osteoporosis, Postmenopausal | 11.3 |
| HSPA13 | Osteoporosis, Postmenopausal | 11.3 |
| ADAMTS9 | Osteoporosis, Postmenopausal | 11.29 |
| ARHGAP5 | Osteoporosis, Postmenopausal | 11.29 |
| ARL4C | Osteoporosis, Postmenopausal | 11.29 |
| CUL3 | Osteoporosis, Postmenopausal | 11.29 |
| FAM107B | Osteoporosis, Postmenopausal | 11.29 |
| KLF11 | Osteoporosis, Postmenopausal | 11.29 |
| OAS3 | Osteoporosis, Postmenopausal | 11.29 |
| PLXDC2 | Osteoporosis, Postmenopausal | 11.29 |
| SGO1 | Osteoporosis, Postmenopausal | 11.29 |
| SH3PXD2A | Osteoporosis, Postmenopausal | 11.29 |
| SLC6A12 | Osteoporosis, Postmenopausal | 11.29 |
| SMPDL3A | Osteoporosis, Postmenopausal | 11.29 |
| TJP3 | Osteoporosis, Postmenopausal | 11.29 |
| ATP5PO | Osteoporosis, Postmenopausal | 11.28 |
| CORO1C | Osteoporosis, Postmenopausal | 11.28 |
| EI24 | Osteoporosis, Postmenopausal | 11.28 |
| PLAAT3 | Osteoporosis, Postmenopausal | 11.28 |
| PRKD3 | Osteoporosis, Postmenopausal | 11.28 |
| REEP5 | Osteoporosis, Postmenopausal | 11.28 |
| SERPIND1 | Osteoporosis, Postmenopausal | 11.28 |
| ZBTB10 | Osteoporosis, Postmenopausal | 11.28 |
| CENPK | Osteoporosis, Postmenopausal | 11.27 |
| CNN2 | Osteoporosis, Postmenopausal | 11.27 |
| EMP3 | Osteoporosis, Postmenopausal | 11.27 |
| FAM20C | Osteoporosis, Postmenopausal | 11.27 |
| KEAP1 | Osteoporosis, Postmenopausal | 11.27 |
| ORM2 | Osteoporosis, Postmenopausal | 11.27 |
| PDLIM2 | Osteoporosis, Postmenopausal | 11.27 |
| RECQL4 | Osteoporosis, Postmenopausal | 11.27 |
| TRIM25 | Osteoporosis, Postmenopausal | 11.27 |
| ABHD5 | Osteoporosis, Postmenopausal | 11.26 |
| BMP5 | Osteoporosis, Postmenopausal | 11.26 |
| CCKBR | Osteoporosis, Postmenopausal | 11.26 |
| CD53 | Osteoporosis, Postmenopausal | 11.26 |
| CSNK2A2 | Osteoporosis, Postmenopausal | 11.26 |
| ERCC5 | Osteoporosis, Postmenopausal | 11.26 |
| FBLIM1 | Osteoporosis, Postmenopausal | 11.26 |
| FZD4 | Osteoporosis, Postmenopausal | 11.26 |
| HBA1 | Osteoporosis, Postmenopausal | 11.26 |
| LIMK1 | Osteoporosis, Postmenopausal | 11.26 |
| MIA2 | Osteoporosis, Postmenopausal | 11.26 |
| PALMD | Osteoporosis, Postmenopausal | 11.26 |
| PDZD2 | Osteoporosis, Postmenopausal | 11.26 |
| RPS3A | Osteoporosis, Postmenopausal | 11.26 |
| SLK | Osteoporosis, Postmenopausal | 11.26 |
| SUV39H1 | Osteoporosis, Postmenopausal | 11.26 |
| TBK1 | Osteoporosis, Postmenopausal | 11.26 |
| TROAP | Osteoporosis, Postmenopausal | 11.26 |
| ZFHX3 | Osteoporosis, Postmenopausal | 11.26 |
| DHRS11 | Osteoporosis, Postmenopausal | 11.25 |
| DNM1 | Osteoporosis, Postmenopausal | 11.24 |
| OAS1 | Osteoporosis, Postmenopausal | 11.24 |
| PIK3C3 | Osteoporosis, Postmenopausal | 11.24 |
| RAD54L | Osteoporosis, Postmenopausal | 11.24 |
| STEAP3 | Osteoporosis, Postmenopausal | 11.24 |
| SYT1 | Osteoporosis, Postmenopausal | 11.24 |
| TACSTD2 | Osteoporosis, Postmenopausal | 11.24 |
| CXCL16 | Osteoporosis, Postmenopausal | 11.23 |
| GYS1 | Osteoporosis, Postmenopausal | 11.23 |
| KRT1 | Osteoporosis, Postmenopausal | 11.23 |
| LAMP3 | Osteoporosis, Postmenopausal | 11.23 |
| MIR3960 | Osteoporosis, Postmenopausal | 11.23 |
| NAA15 | Osteoporosis, Postmenopausal | 11.23 |
| SEC61A1 | Osteoporosis, Postmenopausal | 11.23 |
| SERPINA6 | Osteoporosis, Postmenopausal | 11.23 |
| SRD5A2 | Osteoporosis, Postmenopausal | 11.23 |
| ST13 | Osteoporosis, Postmenopausal | 11.23 |
| IGHG2A | Osteoporosis, Postmenopausal | 11.22 |
| MN1B | Osteoporosis, Postmenopausal | 11.22 |
| MR1 | Osteoporosis, Postmenopausal | 11.22 |
| TACC2 | Osteoporosis, Postmenopausal | 11.22 |
| ARHGAP18 | Osteoporosis, Postmenopausal | 11.21 |
| ATP6V1B2 | Osteoporosis, Postmenopausal | 11.21 |
| BMI1 | Osteoporosis, Postmenopausal | 11.21 |
| CCN5 | Osteoporosis, Postmenopausal | 11.21 |
| CKAP2L | Osteoporosis, Postmenopausal | 11.21 |
| CLTB | Osteoporosis, Postmenopausal | 11.21 |
| GFPT2 | Osteoporosis, Postmenopausal | 11.21 |
| HCAR2 | Osteoporosis, Postmenopausal | 11.21 |
| MAL2 | Osteoporosis, Postmenopausal | 11.21 |
| PLIN4 | Osteoporosis, Postmenopausal | 11.21 |
| SFXN2 | Osteoporosis, Postmenopausal | 11.21 |
| ADAM19 | Osteoporosis, Postmenopausal | 11.2 |
| BCL2L2 | Osteoporosis, Postmenopausal | 11.2 |
| CSNK1A1 | Osteoporosis, Postmenopausal | 11.2 |
| EPB41L5 | Osteoporosis, Postmenopausal | 11.2 |
| GCNT2 | Osteoporosis, Postmenopausal | 11.2 |
| PCTP | Osteoporosis, Postmenopausal | 11.2 |
| TRPM4 | Osteoporosis, Postmenopausal | 11.2 |
| ABLIM3 | Osteoporosis, Postmenopausal | 11.19 |
| ANP32E | Osteoporosis, Postmenopausal | 11.19 |
| C5AR1 | Osteoporosis, Postmenopausal | 11.19 |
| CHD7 | Osteoporosis, Postmenopausal | 11.19 |
| DLAT | Osteoporosis, Postmenopausal | 11.19 |
| HLF | Osteoporosis, Postmenopausal | 11.19 |
| LBR | Osteoporosis, Postmenopausal | 11.19 |
| NAV3 | Osteoporosis, Postmenopausal | 11.19 |
| SORBS2 | Osteoporosis, Postmenopausal | 11.19 |
| VMP1 | Osteoporosis, Postmenopausal | 11.19 |
| ABCD2 | Osteoporosis, Postmenopausal | 11.18 |
| AOC1 | Osteoporosis, Postmenopausal | 11.18 |
| CCR6 | Osteoporosis, Postmenopausal | 11.18 |
| CYP51A1 | Osteoporosis, Postmenopausal | 11.18 |
| ERBB4 | Osteoporosis, Postmenopausal | 11.18 |
| FOXN3 | Osteoporosis, Postmenopausal | 11.18 |
| HIVEP1 | Osteoporosis, Postmenopausal | 11.18 |
| IGF2BP3 | Osteoporosis, Postmenopausal | 11.18 |
| KMO | Osteoporosis, Postmenopausal | 11.18 |
| SERPINB8 | Osteoporosis, Postmenopausal | 11.18 |
| USP9X | Osteoporosis, Postmenopausal | 11.18 |
| YPEL5 | Osteoporosis, Postmenopausal | 11.18 |
| ZNF479 | Osteoporosis, Postmenopausal | 11.18 |
| NET1 | Osteoporosis, Postmenopausal | 11.17 |
| PLA2G6 | Osteoporosis, Postmenopausal | 11.17 |
| SLC16A7 | Osteoporosis, Postmenopausal | 11.17 |
| BDKRB1 | Osteoporosis, Postmenopausal | 11.16 |
| CSRP3 | Osteoporosis, Postmenopausal | 11.16 |
| DDAH2 | Osteoporosis, Postmenopausal | 11.16 |
| DOCK8 | Osteoporosis, Postmenopausal | 11.16 |
| H2AZ1 | Osteoporosis, Postmenopausal | 11.16 |
| ILF3 | Osteoporosis, Postmenopausal | 11.16 |
| MYO10 | Osteoporosis, Postmenopausal | 11.16 |
| PCSK6 | Osteoporosis, Postmenopausal | 11.16 |
| PLEKHF1 | Osteoporosis, Postmenopausal | 11.16 |
| PNN | Osteoporosis, Postmenopausal | 11.16 |
| RHOQ | Osteoporosis, Postmenopausal | 11.16 |
| ADAM8 | Osteoporosis, Postmenopausal | 11.15 |
| AKT1S1 | Osteoporosis, Postmenopausal | 11.15 |
| GSTA1 | Osteoporosis, Postmenopausal | 11.15 |
| INHA | Osteoporosis, Postmenopausal | 11.15 |
| NFE2L3 | Osteoporosis, Postmenopausal | 11.15 |
| SERPINA3 | Osteoporosis, Postmenopausal | 11.15 |
| ZDHHC2 | Osteoporosis, Postmenopausal | 11.15 |
| DDR1 | Osteoporosis, Postmenopausal | 11.14 |
| ENPEP | Osteoporosis, Postmenopausal | 11.14 |
| LPO | Osteoporosis, Postmenopausal | 11.14 |
| SERPINI1 | Osteoporosis, Postmenopausal | 11.14 |
| STARD13 | Osteoporosis, Postmenopausal | 11.14 |
| TENM3 | Osteoporosis, Postmenopausal | 11.14 |
| CNDP2 | Osteoporosis, Postmenopausal | 11.13 |
| EFHD2 | Osteoporosis, Postmenopausal | 11.13 |
| FNDC3B | Osteoporosis, Postmenopausal | 11.13 |
| FRY | Osteoporosis, Postmenopausal | 11.13 |
| GAST | Osteoporosis, Postmenopausal | 11.13 |
| HTR3E | Osteoporosis, Postmenopausal | 11.13 |
| IFI35 | Osteoporosis, Postmenopausal | 11.13 |
| IQGAP3 | Osteoporosis, Postmenopausal | 11.13 |
| MEOX1 | Osteoporosis, Postmenopausal | 11.13 |
| MIR29B | Osteoporosis, Postmenopausal | 11.13 |
| MIR576 | Osteoporosis, Postmenopausal | 11.13 |
| MMD | Osteoporosis, Postmenopausal | 11.13 |
| PCDH7 | Osteoporosis, Postmenopausal | 11.13 |
| PLA1A | Osteoporosis, Postmenopausal | 11.13 |
| PRAMEF5 | Osteoporosis, Postmenopausal | 11.13 |
| PRXL2A | Osteoporosis, Postmenopausal | 11.13 |
| SEC31A | Osteoporosis, Postmenopausal | 11.13 |
| UAP1 | Osteoporosis, Postmenopausal | 11.13 |
| VTG1 | Osteoporosis, Postmenopausal | 11.13 |
| AMACR | Osteoporosis, Postmenopausal | 11.12 |
| CMPK2 | Osteoporosis, Postmenopausal | 11.12 |
| CNN3 | Osteoporosis, Postmenopausal | 11.12 |
| FILIP1L | Osteoporosis, Postmenopausal | 11.12 |
| MECOM | Osteoporosis, Postmenopausal | 11.12 |
| PLCG2 | Osteoporosis, Postmenopausal | 11.12 |
| EFHD1 | Osteoporosis, Postmenopausal | 11.11 |
| HTR2B | Osteoporosis, Postmenopausal | 11.11 |
| KDELR3 | Osteoporosis, Postmenopausal | 11.11 |
| POLA2 | Osteoporosis, Postmenopausal | 11.11 |
| RRP12 | Osteoporosis, Postmenopausal | 11.11 |
| SEC24D | Osteoporosis, Postmenopausal | 11.11 |
| SF1 | Osteoporosis, Postmenopausal | 11.11 |
| SKIL | Osteoporosis, Postmenopausal | 11.11 |
| SUMO1 | Osteoporosis, Postmenopausal | 11.11 |
| UCP3 | Osteoporosis, Postmenopausal | 11.11 |
| LIMCH1 | Osteoporosis, Postmenopausal | 11.1 |
| PERP | Osteoporosis, Postmenopausal | 11.1 |
| PLIN3 | Osteoporosis, Postmenopausal | 11.1 |
| RNF144B | Osteoporosis, Postmenopausal | 11.1 |
| RPA3 | Osteoporosis, Postmenopausal | 11.1 |
| CACNA1D | Osteoporosis, Postmenopausal | 11.09 |
| DLC1 | Osteoporosis, Postmenopausal | 11.09 |
| ESR1.L | Osteoporosis, Postmenopausal | 11.09 |
| GNAO1 | Osteoporosis, Postmenopausal | 11.09 |
| GNB1 | Osteoporosis, Postmenopausal | 11.09 |
| KNTC1 | Osteoporosis, Postmenopausal | 11.09 |
| RFTN1 | Osteoporosis, Postmenopausal | 11.09 |
| ATP5F1E | Osteoporosis, Postmenopausal | 11.08 |
| ECI2 | Osteoporosis, Postmenopausal | 11.08 |
| LTBR | Osteoporosis, Postmenopausal | 11.08 |
| NR1H4 | Osteoporosis, Postmenopausal | 11.08 |
| NUP210 | Osteoporosis, Postmenopausal | 11.08 |
| PXDN | Osteoporosis, Postmenopausal | 11.08 |
| BCL10 | Osteoporosis, Postmenopausal | 11.07 |
| COLEC12 | Osteoporosis, Postmenopausal | 11.07 |
| PLP1 | Osteoporosis, Postmenopausal | 11.07 |
| STEAP1 | Osteoporosis, Postmenopausal | 11.07 |
| AK2 | Osteoporosis, Postmenopausal | 11.06 |
| CRHR1 | Osteoporosis, Postmenopausal | 11.06 |
| ENDOG | Osteoporosis, Postmenopausal | 11.06 |
| NFE2L1 | Osteoporosis, Postmenopausal | 11.06 |
| PIM3 | Osteoporosis, Postmenopausal | 11.06 |
| PRIM2 | Osteoporosis, Postmenopausal | 11.06 |
| SOS1 | Osteoporosis, Postmenopausal | 11.06 |
| UCK2 | Osteoporosis, Postmenopausal | 11.06 |
| AKAP1 | Osteoporosis, Postmenopausal | 11.05 |
| MAPK8 | Osteoporosis, Postmenopausal | 11.05 |
| NFKBIE | Osteoporosis, Postmenopausal | 11.05 |
| POR | Osteoporosis, Postmenopausal | 11.05 |
| GAS1 | Osteoporosis, Postmenopausal | 11.04 |
| IRF6 | Osteoporosis, Postmenopausal | 11.04 |
| PANK1 | Osteoporosis, Postmenopausal | 11.04 |
| SPDL1 | Osteoporosis, Postmenopausal | 11.04 |
| TXN2 | Osteoporosis, Postmenopausal | 11.04 |
| APOC2 | Osteoporosis, Postmenopausal | 11.03 |
| CPEB4 | Osteoporosis, Postmenopausal | 11.03 |
| DLL1 | Osteoporosis, Postmenopausal | 11.03 |
| FAM162A | Osteoporosis, Postmenopausal | 11.03 |
| ELL2 | Osteoporosis, Postmenopausal | 11.02 |
| POLE | Osteoporosis, Postmenopausal | 11.02 |
| PSPH | Osteoporosis, Postmenopausal | 11.02 |
| SPC24 | Osteoporosis, Postmenopausal | 11.02 |
| UBE2I | Osteoporosis, Postmenopausal | 11.02 |
| ARNT2 | Osteoporosis, Postmenopausal | 11.01 |
| ESR2.L | Osteoporosis, Postmenopausal | 11.01 |
| FRZB | Osteoporosis, Postmenopausal | 11.01 |
| GABBR2 | Osteoporosis, Postmenopausal | 11.01 |
| HACL1 | Osteoporosis, Postmenopausal | 11.01 |
| HDAC9 | Osteoporosis, Postmenopausal | 11.01 |
| ITGA2B | Osteoporosis, Postmenopausal | 11.01 |
| S1PR3 | Osteoporosis, Postmenopausal | 11.01 |
| SMAD1 | Osteoporosis, Postmenopausal | 11.01 |
| TP53BP1 | Osteoporosis, Postmenopausal | 11.01 |
| ATP2A1 | Osteoporosis, Postmenopausal | 11 |
| CDH11 | Osteoporosis, Postmenopausal | 11 |
| CSK | Osteoporosis, Postmenopausal | 11 |
| MAP4 | Osteoporosis, Postmenopausal | 11 |
| SLC7A8 | Osteoporosis, Postmenopausal | 11 |
| SMAD6 | Osteoporosis, Postmenopausal | 11 |
| TBXAS1 | Osteoporosis, Postmenopausal | 11 |
| APLP2 | Osteoporosis, Postmenopausal | 10.99 |
| NRP1A | Osteoporosis, Postmenopausal | 10.99 |
| PPP1CB | Osteoporosis, Postmenopausal | 10.99 |
| ST3GAL1 | Osteoporosis, Postmenopausal | 10.99 |
| STAT4 | Osteoporosis, Postmenopausal | 10.99 |
| B4GALT1 | Osteoporosis, Postmenopausal | 10.98 |
| BMPR2 | Osteoporosis, Postmenopausal | 10.98 |
| CLDN7 | Osteoporosis, Postmenopausal | 10.98 |
| GBP1 | Osteoporosis, Postmenopausal | 10.98 |
| LFNG | Osteoporosis, Postmenopausal | 10.98 |
| MYH11 | Osteoporosis, Postmenopausal | 10.98 |
| SPRY1 | Osteoporosis, Postmenopausal | 10.98 |
| CD163 | Osteoporosis, Postmenopausal | 10.97 |
| DAG1 | Osteoporosis, Postmenopausal | 10.97 |
| RPA2 | Osteoporosis, Postmenopausal | 10.97 |
| ABCC8 | Osteoporosis, Postmenopausal | 10.96 |
| FAM43A | Osteoporosis, Postmenopausal | 10.96 |
| HLA-B | Osteoporosis, Postmenopausal | 10.96 |
| HMBS | Osteoporosis, Postmenopausal | 10.96 |
| IFI30 | Osteoporosis, Postmenopausal | 10.96 |
| JDP2 | Osteoporosis, Postmenopausal | 10.96 |
| LAPTM5 | Osteoporosis, Postmenopausal | 10.96 |
| MFAP4 | Osteoporosis, Postmenopausal | 10.96 |
| RIDA | Osteoporosis, Postmenopausal | 10.96 |
| SUCLG2 | Osteoporosis, Postmenopausal | 10.96 |
| TGFBR3 | Osteoporosis, Postmenopausal | 10.96 |
| UGP2 | Osteoporosis, Postmenopausal | 10.96 |
| VEGFB | Osteoporosis, Postmenopausal | 10.96 |
| MAP3K6 | Osteoporosis, Postmenopausal | 10.95 |
| NGEF | Osteoporosis, Postmenopausal | 10.95 |
| PEG10 | Osteoporosis, Postmenopausal | 10.95 |
| RPS6KA5 | Osteoporosis, Postmenopausal | 10.95 |
| RRS1 | Osteoporosis, Postmenopausal | 10.95 |
| RYR1 | Osteoporosis, Postmenopausal | 10.95 |
| XRCC6 | Osteoporosis, Postmenopausal | 10.95 |
| CRIP1 | Osteoporosis, Postmenopausal | 10.94 |
| DLK1 | Osteoporosis, Postmenopausal | 10.94 |
| IARS1 | Osteoporosis, Postmenopausal | 10.94 |
| RECK | Osteoporosis, Postmenopausal | 10.94 |
| SEMA6D | Osteoporosis, Postmenopausal | 10.94 |
| ACACA | Osteoporosis, Postmenopausal | 10.93 |
| ADH4 | Osteoporosis, Postmenopausal | 10.93 |
| ARHGAP11A | Osteoporosis, Postmenopausal | 10.93 |
| AR.L | Osteoporosis, Postmenopausal | 10.93 |
| CBX5 | Osteoporosis, Postmenopausal | 10.93 |
| DICER1 | Osteoporosis, Postmenopausal | 10.93 |
| FARP1 | Osteoporosis, Postmenopausal | 10.93 |
| GABRB1 | Osteoporosis, Postmenopausal | 10.93 |
| RIPK2 | Osteoporosis, Postmenopausal | 10.93 |
| CAMK2N1 | Osteoporosis, Postmenopausal | 10.92 |
| DCK | Osteoporosis, Postmenopausal | 10.92 |
| FZD7 | Osteoporosis, Postmenopausal | 10.92 |
| HCK | Osteoporosis, Postmenopausal | 10.92 |
| MICAL2 | Osteoporosis, Postmenopausal | 10.92 |
| RANGAP1 | Osteoporosis, Postmenopausal | 10.92 |
| RAP1GAP | Osteoporosis, Postmenopausal | 10.92 |
| SARS1 | Osteoporosis, Postmenopausal | 10.92 |
| TIMELESS | Osteoporosis, Postmenopausal | 10.92 |
| ADCY7 | Osteoporosis, Postmenopausal | 10.91 |
| AEN | Osteoporosis, Postmenopausal | 10.91 |
| DDX39A | Osteoporosis, Postmenopausal | 10.91 |
| DHRS4 | Osteoporosis, Postmenopausal | 10.91 |
| NEAT1 | Osteoporosis, Postmenopausal | 10.91 |
| DNAJB11 | Osteoporosis, Postmenopausal | 10.9 |
| HNRNPAB | Osteoporosis, Postmenopausal | 10.9 |
| HOPX | Osteoporosis, Postmenopausal | 10.9 |
| KRTAP6-3 | Osteoporosis, Postmenopausal | 10.9 |
| OR10G2 | Osteoporosis, Postmenopausal | 10.9 |
| PPP1R10 | Osteoporosis, Postmenopausal | 10.9 |
| PTMS | Osteoporosis, Postmenopausal | 10.9 |
| ETFB | Osteoporosis, Postmenopausal | 10.89 |
| LPAR1 | Osteoporosis, Postmenopausal | 10.89 |
| SQOR | Osteoporosis, Postmenopausal | 10.89 |
| CCDC80 | Osteoporosis, Postmenopausal | 10.88 |
| INCENP | Osteoporosis, Postmenopausal | 10.88 |
| LAMA1 | Osteoporosis, Postmenopausal | 10.88 |
| MARCKSL1 | Osteoporosis, Postmenopausal | 10.88 |
| RYR2 | Osteoporosis, Postmenopausal | 10.88 |
| WIPI1 | Osteoporosis, Postmenopausal | 10.88 |
| YWHAQ | Osteoporosis, Postmenopausal | 10.88 |
| CARHSP1 | Osteoporosis, Postmenopausal | 10.87 |
| CHAF1B | Osteoporosis, Postmenopausal | 10.87 |
| E2F3 | Osteoporosis, Postmenopausal | 10.87 |
| PRSS8 | Osteoporosis, Postmenopausal | 10.87 |
| TENT5C | Osteoporosis, Postmenopausal | 10.87 |
| TLR1 | Osteoporosis, Postmenopausal | 10.87 |
| ABHD4 | Osteoporosis, Postmenopausal | 10.86 |
| DIAPH3 | Osteoporosis, Postmenopausal | 10.86 |
| ESM1 | Osteoporosis, Postmenopausal | 10.86 |
| MSR1 | Osteoporosis, Postmenopausal | 10.86 |
| PGM3 | Osteoporosis, Postmenopausal | 10.86 |
| ADRA1A | Osteoporosis, Postmenopausal | 10.85 |
| APLN | Osteoporosis, Postmenopausal | 10.85 |
| EPHX1 | Osteoporosis, Postmenopausal | 10.85 |
| HIGD1A | Osteoporosis, Postmenopausal | 10.85 |
| IVNS1ABP | Osteoporosis, Postmenopausal | 10.85 |
| LRP8 | Osteoporosis, Postmenopausal | 10.85 |
| MTR | Osteoporosis, Postmenopausal | 10.85 |
| TACR1 | Osteoporosis, Postmenopausal | 10.85 |
| ZNF137P | Osteoporosis, Postmenopausal | 10.85 |
| AGTR2 | Osteoporosis, Postmenopausal | 10.84 |
| SLC4A7 | Osteoporosis, Postmenopausal | 10.84 |
| SVIL | Osteoporosis, Postmenopausal | 10.84 |
| ABHD2 | Osteoporosis, Postmenopausal | 10.83 |
| BASP1 | Osteoporosis, Postmenopausal | 10.83 |
| CDK5 | Osteoporosis, Postmenopausal | 10.83 |
| COL14A1 | Osteoporosis, Postmenopausal | 10.83 |
| RRAGD | Osteoporosis, Postmenopausal | 10.83 |
| STOM | Osteoporosis, Postmenopausal | 10.83 |
| BCAR1 | Osteoporosis, Postmenopausal | 10.82 |
| DNAJB6 | Osteoporosis, Postmenopausal | 10.82 |
| HTR1B | Osteoporosis, Postmenopausal | 10.81 |
| NAV2 | Osteoporosis, Postmenopausal | 10.81 |
| ZYX | Osteoporosis, Postmenopausal | 10.81 |
| ANKRD37 | Osteoporosis, Postmenopausal | 10.8 |
| FBLN2 | Osteoporosis, Postmenopausal | 10.8 |
| HNRNPU | Osteoporosis, Postmenopausal | 10.8 |
| OR10AL6 | Osteoporosis, Postmenopausal | 10.8 |
| SLC38A4 | Osteoporosis, Postmenopausal | 10.8 |
| SPON2 | Osteoporosis, Postmenopausal | 10.8 |
| TM4SF1 | Osteoporosis, Postmenopausal | 10.8 |
| TMSB10 | Osteoporosis, Postmenopausal | 10.8 |
| BCL11BA | Osteoporosis, Postmenopausal | 10.79 |
| C1QC | Osteoporosis, Postmenopausal | 10.79 |
| EMR4 | Osteoporosis, Postmenopausal | 10.79 |
| NEDD4 | Osteoporosis, Postmenopausal | 10.79 |
| SLC7A5P1 | Osteoporosis, Postmenopausal | 10.79 |
| SNORA63 | Osteoporosis, Postmenopausal | 10.79 |
| TNS3 | Osteoporosis, Postmenopausal | 10.79 |
| ZFP469 | Osteoporosis, Postmenopausal | 10.79 |
| AMH | Osteoporosis, Postmenopausal | 10.78 |
| CHRNA3 | Osteoporosis, Postmenopausal | 10.78 |
| CRIP2 | Osteoporosis, Postmenopausal | 10.78 |
| CYP2K5 | Osteoporosis, Postmenopausal | 10.78 |
| FAM13A | Osteoporosis, Postmenopausal | 10.78 |
| LSP1 | Osteoporosis, Postmenopausal | 10.78 |
| MAP3K7 | Osteoporosis, Postmenopausal | 10.78 |
| PSMA2 | Osteoporosis, Postmenopausal | 10.78 |
| SLC25A10 | Osteoporosis, Postmenopausal | 10.78 |
| TREML3P | Osteoporosis, Postmenopausal | 10.78 |
| VEGFD | Osteoporosis, Postmenopausal | 10.78 |
| ECM1 | Osteoporosis, Postmenopausal | 10.77 |
| LRP5 | Osteoporosis, Postmenopausal | 10.77 |
| ST3GAL5 | Osteoporosis, Postmenopausal | 10.77 |
| CTNND1 | Osteoporosis, Postmenopausal | 10.76 |
| HADHB | Osteoporosis, Postmenopausal | 10.76 |
| ITGB6 | Osteoporosis, Postmenopausal | 10.76 |
| KRT14 | Osteoporosis, Postmenopausal | 10.76 |
| PSIP1 | Osteoporosis, Postmenopausal | 10.76 |
| STX3 | Osteoporosis, Postmenopausal | 10.76 |
| YWHAG | Osteoporosis, Postmenopausal | 10.76 |
| PNPLA3 | Osteoporosis, Postmenopausal | 10.75 |
| TIA1 | Osteoporosis, Postmenopausal | 10.75 |
| TRPC6 | Osteoporosis, Postmenopausal | 10.75 |
| GLS2 | Osteoporosis, Postmenopausal | 10.74 |
| MYBBP1A | Osteoporosis, Postmenopausal | 10.74 |
| RPS19 | Osteoporosis, Postmenopausal | 10.74 |
| TGFB1I1 | Osteoporosis, Postmenopausal | 10.74 |
| ALAS2 | Osteoporosis, Postmenopausal | 10.73 |
| CLCF1 | Osteoporosis, Postmenopausal | 10.73 |
| EPB41L3 | Osteoporosis, Postmenopausal | 10.73 |
| ITPR2 | Osteoporosis, Postmenopausal | 10.73 |
| PFN1 | Osteoporosis, Postmenopausal | 10.73 |
| STARD4 | Osteoporosis, Postmenopausal | 10.73 |
| ACTG2 | Osteoporosis, Postmenopausal | 10.72 |
| CD59 | Osteoporosis, Postmenopausal | 10.72 |
| FZD2 | Osteoporosis, Postmenopausal | 10.72 |
| NPAS2 | Osteoporosis, Postmenopausal | 10.72 |
| SLC22A4 | Osteoporosis, Postmenopausal | 10.72 |
| APOC1 | Osteoporosis, Postmenopausal | 10.71 |
| NOP58 | Osteoporosis, Postmenopausal | 10.71 |
| CAD | Osteoporosis, Postmenopausal | 10.7 |
| CD69 | Osteoporosis, Postmenopausal | 10.7 |
| COL7A1 | Osteoporosis, Postmenopausal | 10.7 |
| ERCC6 | Osteoporosis, Postmenopausal | 10.7 |
| ERP29 | Osteoporosis, Postmenopausal | 10.7 |
| FDXR | Osteoporosis, Postmenopausal | 10.7 |
| MLH1 | Osteoporosis, Postmenopausal | 10.7 |
| MTHFD1 | Osteoporosis, Postmenopausal | 10.7 |
| MTSS1 | Osteoporosis, Postmenopausal | 10.7 |
| SH3KBP1 | Osteoporosis, Postmenopausal | 10.7 |
| ATG12 | Osteoporosis, Postmenopausal | 10.69 |
| BARD1 | Osteoporosis, Postmenopausal | 10.69 |
| DUSP2 | Osteoporosis, Postmenopausal | 10.69 |
| FOXP3 | Osteoporosis, Postmenopausal | 10.69 |
| LINC02875 | Osteoporosis, Postmenopausal | 10.69 |
| MIR198 | Osteoporosis, Postmenopausal | 10.69 |
| PHLPP | Osteoporosis, Postmenopausal | 10.69 |
| POLD1 | Osteoporosis, Postmenopausal | 10.69 |
| PRB3 | Osteoporosis, Postmenopausal | 10.69 |
| SNORD34 | Osteoporosis, Postmenopausal | 10.69 |
| SNORD59A | Osteoporosis, Postmenopausal | 10.69 |
| ABCC9 | Osteoporosis, Postmenopausal | 10.68 |
| ADD3 | Osteoporosis, Postmenopausal | 10.68 |
| ATP7A | Osteoporosis, Postmenopausal | 10.68 |
| CDO1 | Osteoporosis, Postmenopausal | 10.68 |
| GLT6D1 | Osteoporosis, Postmenopausal | 10.67 |
| H19 | Osteoporosis, Postmenopausal | 10.67 |
| KRTAP10-7 | Osteoporosis, Postmenopausal | 10.67 |
| ADH1 | Osteoporosis, Postmenopausal | 10.66 |
| ELAVL1 | Osteoporosis, Postmenopausal | 10.66 |
| MIR687 | Osteoporosis, Postmenopausal | 10.66 |
| PNRC1 | Osteoporosis, Postmenopausal | 10.66 |
| PRDX5 | Osteoporosis, Postmenopausal | 10.66 |
| SLC39A10 | Osteoporosis, Postmenopausal | 10.66 |
| TEK | Osteoporosis, Postmenopausal | 10.66 |
| FLNC | Osteoporosis, Postmenopausal | 10.65 |
| GSTM5 | Osteoporosis, Postmenopausal | 10.65 |
| HNMT | Osteoporosis, Postmenopausal | 10.65 |
| KLRC4 | Osteoporosis, Postmenopausal | 10.65 |
| MYOF | Osteoporosis, Postmenopausal | 10.65 |
| PYGM | Osteoporosis, Postmenopausal | 10.65 |
| RRBP1 | Osteoporosis, Postmenopausal | 10.65 |
| SRRM2 | Osteoporosis, Postmenopausal | 10.65 |
| TRAF2 | Osteoporosis, Postmenopausal | 10.65 |
| VAT1 | Osteoporosis, Postmenopausal | 10.65 |
| ADIPOR2 | Osteoporosis, Postmenopausal | 10.64 |
| CGA | Osteoporosis, Postmenopausal | 10.64 |
| CYP4A10 | Osteoporosis, Postmenopausal | 10.64 |
| HDAC3 | Osteoporosis, Postmenopausal | 10.64 |
| LY6E | Osteoporosis, Postmenopausal | 10.64 |
| SHCBP1 | Osteoporosis, Postmenopausal | 10.64 |
| RASSF1 | Osteoporosis, Postmenopausal | 10.63 |
| RGS5 | Osteoporosis, Postmenopausal | 10.63 |
| ELOVL2 | Osteoporosis, Postmenopausal | 10.62 |
| MAPKAPK2 | Osteoporosis, Postmenopausal | 10.62 |
| PPIF | Osteoporosis, Postmenopausal | 10.62 |
| ETV5 | Osteoporosis, Postmenopausal | 10.61 |
| GLIPR1 | Osteoporosis, Postmenopausal | 10.61 |
| PDPK1 | Osteoporosis, Postmenopausal | 10.61 |
| TPO | Osteoporosis, Postmenopausal | 10.61 |
| AARS1 | Osteoporosis, Postmenopausal | 10.6 |
| IER5 | Osteoporosis, Postmenopausal | 10.6 |
| INHBAB | Osteoporosis, Postmenopausal | 10.6 |
| ITGA1 | Osteoporosis, Postmenopausal | 10.6 |
| MCP1 | Osteoporosis, Postmenopausal | 10.6 |
| PTGER2 | Osteoporosis, Postmenopausal | 10.6 |
| SRSF7 | Osteoporosis, Postmenopausal | 10.6 |
| SULT1B1 | Osteoporosis, Postmenopausal | 10.6 |
| DBN1 | Osteoporosis, Postmenopausal | 10.59 |
| G3BP1 | Osteoporosis, Postmenopausal | 10.59 |
| KLF15 | Osteoporosis, Postmenopausal | 10.59 |
| RACK1 | Osteoporosis, Postmenopausal | 10.59 |
| RPL6 | Osteoporosis, Postmenopausal | 10.59 |
| TRAP1 | Osteoporosis, Postmenopausal | 10.59 |
| AKT3 | Osteoporosis, Postmenopausal | 10.58 |
| CORO1A | Osteoporosis, Postmenopausal | 10.58 |
| ABCC6 | Osteoporosis, Postmenopausal | 10.57 |
| HNRNPDL | Osteoporosis, Postmenopausal | 10.57 |
| MDK | Osteoporosis, Postmenopausal | 10.57 |
| NABP1 | Osteoporosis, Postmenopausal | 10.57 |
| PA2G4 | Osteoporosis, Postmenopausal | 10.57 |
| PDLIM7 | Osteoporosis, Postmenopausal | 10.57 |
| EIF4A1 | Osteoporosis, Postmenopausal | 10.56 |
| LTB | Osteoporosis, Postmenopausal | 10.56 |
| MECP2 | Osteoporosis, Postmenopausal | 10.56 |
| NRP2 | Osteoporosis, Postmenopausal | 10.56 |
| TSKU | Osteoporosis, Postmenopausal | 10.56 |
| CAVIN2 | Osteoporosis, Postmenopausal | 10.55 |
| CYGB | Osteoporosis, Postmenopausal | 10.55 |
| DLD | Osteoporosis, Postmenopausal | 10.55 |
| SORBS1 | Osteoporosis, Postmenopausal | 10.55 |
| ARHGDIA | Osteoporosis, Postmenopausal | 10.54 |
| PRMT1 | Osteoporosis, Postmenopausal | 10.54 |
| DLG1 | Osteoporosis, Postmenopausal | 10.53 |
| FAT1 | Osteoporosis, Postmenopausal | 10.53 |
| LRRFIP1 | Osteoporosis, Postmenopausal | 10.53 |
| NOTCH3 | Osteoporosis, Postmenopausal | 10.53 |
| SRSF5 | Osteoporosis, Postmenopausal | 10.53 |
| ARRB2 | Osteoporosis, Postmenopausal | 10.52 |
| ATAD2 | Osteoporosis, Postmenopausal | 10.52 |
| EIF4G1 | Osteoporosis, Postmenopausal | 10.52 |
| HCG27 | Osteoporosis, Postmenopausal | 10.52 |
| HTR2C | Osteoporosis, Postmenopausal | 10.52 |
| MYH10 | Osteoporosis, Postmenopausal | 10.52 |
| SRM | Osteoporosis, Postmenopausal | 10.52 |
| SYN1 | Osteoporosis, Postmenopausal | 10.52 |
| ZRP | Osteoporosis, Postmenopausal | 10.52 |
| ANKRD1 | Osteoporosis, Postmenopausal | 10.51 |
| CD63 | Osteoporosis, Postmenopausal | 10.51 |
| CDC14C | Osteoporosis, Postmenopausal | 10.51 |
| MALAT1 | Osteoporosis, Postmenopausal | 10.51 |
| MIR3911 | Osteoporosis, Postmenopausal | 10.51 |
| RXRG | Osteoporosis, Postmenopausal | 10.51 |
| SFRP2 | Osteoporosis, Postmenopausal | 10.51 |
| SPPL2C | Osteoporosis, Postmenopausal | 10.51 |
| GPT2 | Osteoporosis, Postmenopausal | 10.5 |
| TARS1 | Osteoporosis, Postmenopausal | 10.5 |
| BAMBI | Osteoporosis, Postmenopausal | 10.49 |
| CMBL | Osteoporosis, Postmenopausal | 10.49 |
| IYD | Osteoporosis, Postmenopausal | 10.49 |
| LTBP1 | Osteoporosis, Postmenopausal | 10.49 |
| PAH | Osteoporosis, Postmenopausal | 10.49 |
| STAT2 | Osteoporosis, Postmenopausal | 10.49 |
| TRP53 | Osteoporosis, Postmenopausal | 10.49 |
| ADRA1B | Osteoporosis, Postmenopausal | 10.48 |
| CLDN3 | Osteoporosis, Postmenopausal | 10.48 |
| GUCY1A1 | Osteoporosis, Postmenopausal | 10.48 |
| L1CAM | Osteoporosis, Postmenopausal | 10.48 |
| PTMA | Osteoporosis, Postmenopausal | 10.48 |
| PTPRC | Osteoporosis, Postmenopausal | 10.48 |
| ANG | Osteoporosis, Postmenopausal | 10.47 |
| GSTZ1 | Osteoporosis, Postmenopausal | 10.47 |
| MTB | Osteoporosis, Postmenopausal | 10.47 |
| ZFP36L1 | Osteoporosis, Postmenopausal | 10.47 |
| C1QA | Osteoporosis, Postmenopausal | 10.46 |
| COL6A2 | Osteoporosis, Postmenopausal | 10.46 |
| RBM3 | Osteoporosis, Postmenopausal | 10.46 |
| SERPINA7 | Osteoporosis, Postmenopausal | 10.46 |
| GATA6 | Osteoporosis, Postmenopausal | 10.45 |
| MAPK10 | Osteoporosis, Postmenopausal | 10.45 |
| TUBB2B | Osteoporosis, Postmenopausal | 10.45 |
| AHRR | Osteoporosis, Postmenopausal | 10.44 |
| ATP1A2 | Osteoporosis, Postmenopausal | 10.44 |
| DCLK1 | Osteoporosis, Postmenopausal | 10.44 |
| DDX21 | Osteoporosis, Postmenopausal | 10.44 |
| FUT5 | Osteoporosis, Postmenopausal | 10.44 |
| KCNMA1 | Osteoporosis, Postmenopausal | 10.44 |
| PDYN | Osteoporosis, Postmenopausal | 10.44 |
| SCARB2 | Osteoporosis, Postmenopausal | 10.44 |
| TRH | Osteoporosis, Postmenopausal | 10.44 |
| TRIM43B | Osteoporosis, Postmenopausal | 10.44 |
| TSC2 | Osteoporosis, Postmenopausal | 10.44 |
| FIS1 | Osteoporosis, Postmenopausal | 10.43 |
| MAPK9 | Osteoporosis, Postmenopausal | 10.43 |
| SAA1 | Osteoporosis, Postmenopausal | 10.43 |
| BIK | Osteoporosis, Postmenopausal | 10.42 |
| CCL6 | Osteoporosis, Postmenopausal | 10.42 |
| GFRA1 | Osteoporosis, Postmenopausal | 10.42 |
| IFITM3 | Osteoporosis, Postmenopausal | 10.42 |
| LIMA1 | Osteoporosis, Postmenopausal | 10.42 |
| RETN | Osteoporosis, Postmenopausal | 10.42 |
| RTN4 | Osteoporosis, Postmenopausal | 10.42 |
| ADORA2B | Osteoporosis, Postmenopausal | 10.41 |
| CHI3L1 | Osteoporosis, Postmenopausal | 10.41 |
| HSD17B10 | Osteoporosis, Postmenopausal | 10.41 |
| IFNB1 | Osteoporosis, Postmenopausal | 10.41 |
| OR1D2 | Osteoporosis, Postmenopausal | 10.41 |
| PC | Osteoporosis, Postmenopausal | 10.41 |
| TM7SF2 | Osteoporosis, Postmenopausal | 10.41 |
| DRD3 | Osteoporosis, Postmenopausal | 10.4 |
| HIPK2 | Osteoporosis, Postmenopausal | 10.39 |
| AGTR1 | Osteoporosis, Postmenopausal | 10.38 |
| GSTT2 | Osteoporosis, Postmenopausal | 10.38 |
| MAP4K4 | Osteoporosis, Postmenopausal | 10.38 |
| PAICS | Osteoporosis, Postmenopausal | 10.38 |
| PRKACB | Osteoporosis, Postmenopausal | 10.38 |
| DUT | Osteoporosis, Postmenopausal | 10.37 |
| SLC39A14 | Osteoporosis, Postmenopausal | 10.37 |
| ZNF324B | Osteoporosis, Postmenopausal | 10.37 |
| ANGPT1 | Osteoporosis, Postmenopausal | 10.36 |
| APOC3 | Osteoporosis, Postmenopausal | 10.36 |
| CISH | Osteoporosis, Postmenopausal | 10.36 |
| PNP | Osteoporosis, Postmenopausal | 10.36 |
| SELENOV | Osteoporosis, Postmenopausal | 10.36 |
| C14ORF180 | Osteoporosis, Postmenopausal | 10.35 |
| GEM | Osteoporosis, Postmenopausal | 10.35 |
| KIFC1 | Osteoporosis, Postmenopausal | 10.35 |
| LINC02870 | Osteoporosis, Postmenopausal | 10.35 |
| LTF | Osteoporosis, Postmenopausal | 10.35 |
| SNORD46 | Osteoporosis, Postmenopausal | 10.35 |
| TRGV9 | Osteoporosis, Postmenopausal | 10.35 |
| ALDOB | Osteoporosis, Postmenopausal | 10.34 |
| GLDC | Osteoporosis, Postmenopausal | 10.34 |
| MYH9 | Osteoporosis, Postmenopausal | 10.34 |
| PTCH1 | Osteoporosis, Postmenopausal | 10.34 |
| TNFAIP2 | Osteoporosis, Postmenopausal | 10.34 |
| FANCD2 | Osteoporosis, Postmenopausal | 10.32 |
| ECI1 | Osteoporosis, Postmenopausal | 10.31 |
| FGF7 | Osteoporosis, Postmenopausal | 10.31 |
| MIR153 | Osteoporosis, Postmenopausal | 10.31 |
| TMEM97 | Osteoporosis, Postmenopausal | 10.31 |
| NDRG2 | Osteoporosis, Postmenopausal | 10.3 |
| DDX17 | Osteoporosis, Postmenopausal | 10.29 |
| HNRNPK | Osteoporosis, Postmenopausal | 10.29 |
| IFI44 | Osteoporosis, Postmenopausal | 10.29 |
| LGALS3BP | Osteoporosis, Postmenopausal | 10.29 |
| MSH6 | Osteoporosis, Postmenopausal | 10.29 |
| REL | Osteoporosis, Postmenopausal | 10.29 |
| RHOC | Osteoporosis, Postmenopausal | 10.29 |
| XPC | Osteoporosis, Postmenopausal | 10.29 |
| COL5A2 | Osteoporosis, Postmenopausal | 10.28 |
| EXO1 | Osteoporosis, Postmenopausal | 10.28 |
| TNNI3 | Osteoporosis, Postmenopausal | 10.28 |
| CCR5 | Osteoporosis, Postmenopausal | 10.27 |
| COX2 | Osteoporosis, Postmenopausal | 10.27 |
| CTTN | Osteoporosis, Postmenopausal | 10.27 |
| DCXR | Osteoporosis, Postmenopausal | 10.27 |
| GBE1 | Osteoporosis, Postmenopausal | 10.27 |
| GPAT3 | Osteoporosis, Postmenopausal | 10.27 |
| MT3 | Osteoporosis, Postmenopausal | 10.27 |
| NPR3 | Osteoporosis, Postmenopausal | 10.27 |
| SDC2 | Osteoporosis, Postmenopausal | 10.27 |
| CKAP2 | Osteoporosis, Postmenopausal | 10.26 |
| POSTN | Osteoporosis, Postmenopausal | 10.26 |
| SLC25A4 | Osteoporosis, Postmenopausal | 10.26 |
| TJP2 | Osteoporosis, Postmenopausal | 10.26 |
| WNT4 | Osteoporosis, Postmenopausal | 10.26 |
| CDCA8 | Osteoporosis, Postmenopausal | 10.25 |
| ECE1 | Osteoporosis, Postmenopausal | 10.25 |
| HYOU1 | Osteoporosis, Postmenopausal | 10.25 |
| KMT2A | Osteoporosis, Postmenopausal | 10.25 |
| PODXL | Osteoporosis, Postmenopausal | 10.25 |
| SDF2L1 | Osteoporosis, Postmenopausal | 10.25 |
| TFPI2 | Osteoporosis, Postmenopausal | 10.25 |
| UGCG | Osteoporosis, Postmenopausal | 10.25 |
| MAP1LC3B | Osteoporosis, Postmenopausal | 10.24 |
| MFN1 | Osteoporosis, Postmenopausal | 10.24 |
| TBX3 | Osteoporosis, Postmenopausal | 10.24 |
| TOP1 | Osteoporosis, Postmenopausal | 10.24 |
| BCAT1 | Osteoporosis, Postmenopausal | 10.22 |
| BCL2A1 | Osteoporosis, Postmenopausal | 10.22 |
| CTCFLOS | Osteoporosis, Postmenopausal | 10.22 |
| GRIA2 | Osteoporosis, Postmenopausal | 10.21 |
| PLTP | Osteoporosis, Postmenopausal | 10.21 |
| GNAI2 | Osteoporosis, Postmenopausal | 10.2 |
| IFIT2 | Osteoporosis, Postmenopausal | 10.2 |
| LCAT | Osteoporosis, Postmenopausal | 10.2 |
| MYCNOS | Osteoporosis, Postmenopausal | 10.2 |
| PART1 | Osteoporosis, Postmenopausal | 10.2 |
| SLCO1A4 | Osteoporosis, Postmenopausal | 10.2 |
| PDCD4-AS1 | Osteoporosis, Postmenopausal | 10.19 |
| LIG1 | Osteoporosis, Postmenopausal | 10.18 |
| MLKL | Osteoporosis, Postmenopausal | 10.18 |
| NANOG | Osteoporosis, Postmenopausal | 10.18 |
| BACE1 | Osteoporosis, Postmenopausal | 10.17 |
| LAMP1 | Osteoporosis, Postmenopausal | 10.17 |
| MCAM | Osteoporosis, Postmenopausal | 10.17 |
| CROCC2 | Osteoporosis, Postmenopausal | 10.16 |
| DPYSL2 | Osteoporosis, Postmenopausal | 10.16 |
| NCL | Osteoporosis, Postmenopausal | 10.16 |
| NTRK1 | Osteoporosis, Postmenopausal | 10.16 |
| PDE5A | Osteoporosis, Postmenopausal | 10.16 |
| PPIA | Osteoporosis, Postmenopausal | 10.16 |
| TLR3 | Osteoporosis, Postmenopausal | 10.16 |
| TUBA1C | Osteoporosis, Postmenopausal | 10.16 |
| ALOX15 | Osteoporosis, Postmenopausal | 10.15 |
| EIF2A | Osteoporosis, Postmenopausal | 10.15 |
| MVP | Osteoporosis, Postmenopausal | 10.15 |
| PSMB8 | Osteoporosis, Postmenopausal | 10.15 |
| SOCS1 | Osteoporosis, Postmenopausal | 10.15 |
| FBLN1 | Osteoporosis, Postmenopausal | 10.14 |
| GNRH1 | Osteoporosis, Postmenopausal | 10.14 |
| TES | Osteoporosis, Postmenopausal | 10.14 |
| ACSL5 | Osteoporosis, Postmenopausal | 10.13 |
| ATP7B | Osteoporosis, Postmenopausal | 10.13 |
| HPX | Osteoporosis, Postmenopausal | 10.13 |
| MYH6 | Osteoporosis, Postmenopausal | 10.13 |
| PTPRF | Osteoporosis, Postmenopausal | 10.13 |
| FN1A | Osteoporosis, Postmenopausal | 10.12 |
| PML | Osteoporosis, Postmenopausal | 10.12 |
| CCK | Osteoporosis, Postmenopausal | 10.11 |
| GATM | Osteoporosis, Postmenopausal | 10.11 |
| NAT1 | Osteoporosis, Postmenopausal | 10.11 |
| ESRRA | Osteoporosis, Postmenopausal | 10.1 |
| HDAC2 | Osteoporosis, Postmenopausal | 10.1 |
| PTK2B | Osteoporosis, Postmenopausal | 10.1 |
| TCRA | Osteoporosis, Postmenopausal | 10.1 |
| RGL4 | Osteoporosis, Postmenopausal | 10.09 |
| RGPD3 | Osteoporosis, Postmenopausal | 10.09 |
| RND3 | Osteoporosis, Postmenopausal | 10.09 |
| CALM1 | Osteoporosis, Postmenopausal | 10.08 |
| DECR1 | Osteoporosis, Postmenopausal | 10.08 |
| GK | Osteoporosis, Postmenopausal | 10.08 |
| F5 | Osteoporosis, Postmenopausal | 10.07 |
| ATF5 | Osteoporosis, Postmenopausal | 10.06 |
| CGB2 | Osteoporosis, Postmenopausal | 10.06 |
| CKM | Osteoporosis, Postmenopausal | 10.06 |
| PPP1R1B | Osteoporosis, Postmenopausal | 10.06 |
| RCAN1 | Osteoporosis, Postmenopausal | 10.06 |
| RGN | Osteoporosis, Postmenopausal | 10.06 |
| TEKTL1 | Osteoporosis, Postmenopausal | 10.06 |
| CDH5 | Osteoporosis, Postmenopausal | 10.05 |
| HSPA6 | Osteoporosis, Postmenopausal | 10.05 |
| MYCN | Osteoporosis, Postmenopausal | 10.05 |
| TRAF6 | Osteoporosis, Postmenopausal | 10.05 |
| CSRP1 | Osteoporosis, Postmenopausal | 10.04 |
| IGFBP6 | Osteoporosis, Postmenopausal | 10.04 |
| IL23A | Osteoporosis, Postmenopausal | 10.04 |
| PHB1 | Osteoporosis, Postmenopausal | 10.04 |
| ATR | Osteoporosis, Postmenopausal | 10.03 |
| DUSP10 | Osteoporosis, Postmenopausal | 10.03 |
| ENO3 | Osteoporosis, Postmenopausal | 10.03 |
| KCNJ5-AS1 | Osteoporosis, Postmenopausal | 10.03 |
| KLRI2 | Osteoporosis, Postmenopausal | 10.03 |
| MAFB | Osteoporosis, Postmenopausal | 10.03 |
| SPTAN1 | Osteoporosis, Postmenopausal | 10.03 |
| STAT6 | Osteoporosis, Postmenopausal | 10.03 |
| TRIM43 | Osteoporosis, Postmenopausal | 10.03 |
| SPHK1 | Osteoporosis, Postmenopausal | 10.02 |
| SFPQ | Osteoporosis, Postmenopausal | 10.01 |
| VASP | Osteoporosis, Postmenopausal | 10.01 |
| AK4 | Osteoporosis, Postmenopausal | 10 |
| BMF | Osteoporosis, Postmenopausal | 10 |
| CAMK2A | Osteoporosis, Postmenopausal | 10 |
| ITGA4 | Osteoporosis, Postmenopausal | 10 |
| VCP | Osteoporosis, Postmenopausal | 10 |
| ARHGAP11B | Osteoporosis, Postmenopausal | 9.99 |
| GSTA5 | Osteoporosis, Postmenopausal | 9.98 |
| MAGEA8 | Osteoporosis, Postmenopausal | 9.98 |
| PLPP3 | Osteoporosis, Postmenopausal | 9.98 |
| CHRNA7 | Osteoporosis, Postmenopausal | 9.97 |
| GUSB | Osteoporosis, Postmenopausal | 9.97 |
| HMGA1 | Osteoporosis, Postmenopausal | 9.96 |
| LAMP2 | Osteoporosis, Postmenopausal | 9.96 |
| RUNX1 | Osteoporosis, Postmenopausal | 9.96 |
| ERCC1 | Osteoporosis, Postmenopausal | 9.95 |
| IL2RA | Osteoporosis, Postmenopausal | 9.95 |
| LGALS9C | Osteoporosis, Postmenopausal | 9.95 |
| NME1 | Osteoporosis, Postmenopausal | 9.95 |
| C20ORF204 | Osteoporosis, Postmenopausal | 9.94 |
| MS4A6BL1 | Osteoporosis, Postmenopausal | 9.94 |
| SPACA1 | Osteoporosis, Postmenopausal | 9.94 |
| TMEM105 | Osteoporosis, Postmenopausal | 9.94 |
| CANX | Osteoporosis, Postmenopausal | 9.93 |
| CHRNB2 | Osteoporosis, Postmenopausal | 9.93 |
| HDC | Osteoporosis, Postmenopausal | 9.93 |
| ZEB2 | Osteoporosis, Postmenopausal | 9.93 |
| ABAT | Osteoporosis, Postmenopausal | 9.92 |
| ANPEP | Osteoporosis, Postmenopausal | 9.92 |
| SLC12A2 | Osteoporosis, Postmenopausal | 9.92 |
| SLC1A4 | Osteoporosis, Postmenopausal | 9.92 |
| ABCC13 | Osteoporosis, Postmenopausal | 9.91 |
| KLK1B5 | Osteoporosis, Postmenopausal | 9.91 |
| RGD1559600 | Osteoporosis, Postmenopausal | 9.91 |
| SERPINA1F | Osteoporosis, Postmenopausal | 9.91 |
| SLC7A5P2 | Osteoporosis, Postmenopausal | 9.91 |
| SPRR2I | Osteoporosis, Postmenopausal | 9.91 |
| VTG6 | Osteoporosis, Postmenopausal | 9.91 |
| ARC | Osteoporosis, Postmenopausal | 9.9 |
| HK1 | Osteoporosis, Postmenopausal | 9.9 |
| PGAM1 | Osteoporosis, Postmenopausal | 9.9 |
| SLC1A1 | Osteoporosis, Postmenopausal | 9.9 |
| RIPK3 | Osteoporosis, Postmenopausal | 9.89 |
| DES | Osteoporosis, Postmenopausal | 9.88 |
| MAP1B | Osteoporosis, Postmenopausal | 9.88 |
| PGPEP1L | Osteoporosis, Postmenopausal | 9.88 |
| PTGER4 | Osteoporosis, Postmenopausal | 9.88 |
| CD55 | Osteoporosis, Postmenopausal | 9.87 |
| FADS1 | Osteoporosis, Postmenopausal | 9.87 |
| GLB1 | Osteoporosis, Postmenopausal | 9.87 |
| H2AC16 | Osteoporosis, Postmenopausal | 9.87 |
| LITAF | Osteoporosis, Postmenopausal | 9.87 |
| RORA | Osteoporosis, Postmenopausal | 9.87 |
| TPM4 | Osteoporosis, Postmenopausal | 9.87 |
| WEE1 | Osteoporosis, Postmenopausal | 9.87 |
| C6ORF52 | Osteoporosis, Postmenopausal | 9.86 |
| MIR450A1 | Osteoporosis, Postmenopausal | 9.86 |
| VCL | Osteoporosis, Postmenopausal | 9.86 |
| HBB | Osteoporosis, Postmenopausal | 9.85 |
| TAP1 | Osteoporosis, Postmenopausal | 9.85 |
| ALDH1A2 | Osteoporosis, Postmenopausal | 9.84 |
| GPX4A | Osteoporosis, Postmenopausal | 9.84 |
| KYNU | Osteoporosis, Postmenopausal | 9.84 |
| STIP1 | Osteoporosis, Postmenopausal | 9.84 |
| TNNT2 | Osteoporosis, Postmenopausal | 9.84 |
| PROM1 | Osteoporosis, Postmenopausal | 9.83 |
| ANKRD36BP1 | Osteoporosis, Postmenopausal | 9.82 |
| CGB5 | Osteoporosis, Postmenopausal | 9.82 |
| DMRTC2 | Osteoporosis, Postmenopausal | 9.82 |
| GPR78 | Osteoporosis, Postmenopausal | 9.82 |
| NCR2 | Osteoporosis, Postmenopausal | 9.82 |
| RAN | Osteoporosis, Postmenopausal | 9.82 |
| SCARNA9 | Osteoporosis, Postmenopausal | 9.82 |
| SNORA64 | Osteoporosis, Postmenopausal | 9.82 |
| ZNF446 | Osteoporosis, Postmenopausal | 9.82 |
| STS | Osteoporosis, Postmenopausal | 9.81 |
| CFB | Osteoporosis, Postmenopausal | 9.8 |
| DNMT3AA | Osteoporosis, Postmenopausal | 9.8 |
| FOSL2 | Osteoporosis, Postmenopausal | 9.8 |
| MIR493 | Osteoporosis, Postmenopausal | 9.8 |
| SC5D | Osteoporosis, Postmenopausal | 9.8 |
| SMIM43 | Osteoporosis, Postmenopausal | 9.8 |
| PECAM1 | Osteoporosis, Postmenopausal | 9.79 |
| ACAT1 | Osteoporosis, Postmenopausal | 9.78 |
| MIR1915 | Osteoporosis, Postmenopausal | 9.78 |
| SLC6A6 | Osteoporosis, Postmenopausal | 9.78 |
| STC1 | Osteoporosis, Postmenopausal | 9.78 |
| TPM3 | Osteoporosis, Postmenopausal | 9.78 |
| XCL2 | Osteoporosis, Postmenopausal | 9.78 |
| FLNA | Osteoporosis, Postmenopausal | 9.77 |
| KRTAP2-4 | Osteoporosis, Postmenopausal | 9.77 |
| LRRC52 | Osteoporosis, Postmenopausal | 9.77 |
| SLC1A5 | Osteoporosis, Postmenopausal | 9.77 |
| TUBA4A | Osteoporosis, Postmenopausal | 9.77 |
| ALDH3A1 | Osteoporosis, Postmenopausal | 9.76 |
| ANXA4 | Osteoporosis, Postmenopausal | 9.76 |
| HSD3B2 | Osteoporosis, Postmenopausal | 9.76 |
| MIR1 | Osteoporosis, Postmenopausal | 9.76 |
| SRD5A1 | Osteoporosis, Postmenopausal | 9.76 |
| ATP5F1B | Osteoporosis, Postmenopausal | 9.75 |
| SLCO1A1 | Osteoporosis, Postmenopausal | 9.75 |
| UGT2B7 | Osteoporosis, Postmenopausal | 9.75 |
| UGDH | Osteoporosis, Postmenopausal | 9.74 |
| BHMT | Osteoporosis, Postmenopausal | 9.73 |
| CKS2 | Osteoporosis, Postmenopausal | 9.72 |
| CX3CL1 | Osteoporosis, Postmenopausal | 9.72 |
| DIABLO | Osteoporosis, Postmenopausal | 9.72 |
| SLC22A3 | Osteoporosis, Postmenopausal | 9.72 |
| MVD | Osteoporosis, Postmenopausal | 9.71 |
| SPRR4 | Osteoporosis, Postmenopausal | 9.71 |
| TCEAL6 | Osteoporosis, Postmenopausal | 9.71 |
| ULBP3 | Osteoporosis, Postmenopausal | 9.71 |
| CSKMT | Osteoporosis, Postmenopausal | 9.7 |
| G6PC1A.2 | Osteoporosis, Postmenopausal | 9.7 |
| VTG7 | Osteoporosis, Postmenopausal | 9.7 |
| CD83 | Osteoporosis, Postmenopausal | 9.69 |
| CTSC | Osteoporosis, Postmenopausal | 9.67 |
| PXDNL | Osteoporosis, Postmenopausal | 9.67 |
| ZNF440 | Osteoporosis, Postmenopausal | 9.67 |
| ACACB | Osteoporosis, Postmenopausal | 9.66 |
| FBN1 | Osteoporosis, Postmenopausal | 9.66 |
| POTEC | Osteoporosis, Postmenopausal | 9.66 |
| ATP5F1A | Osteoporosis, Postmenopausal | 9.65 |
| E2F8 | Osteoporosis, Postmenopausal | 9.65 |
| SFN | Osteoporosis, Postmenopausal | 9.64 |
| CYP51 | Osteoporosis, Postmenopausal | 9.63 |
| GSTO1 | Osteoporosis, Postmenopausal | 9.63 |
| XRCC1 | Osteoporosis, Postmenopausal | 9.63 |
| APOA4 | Osteoporosis, Postmenopausal | 9.62 |
| BMAL1 | Osteoporosis, Postmenopausal | 9.62 |
| CPT1B | Osteoporosis, Postmenopausal | 9.62 |
| CYP8B1 | Osteoporosis, Postmenopausal | 9.62 |
| HADH | Osteoporosis, Postmenopausal | 9.62 |
| GPR31 | Osteoporosis, Postmenopausal | 9.61 |
| KLRA2 | Osteoporosis, Postmenopausal | 9.61 |
| MUC12 | Osteoporosis, Postmenopausal | 9.61 |
| OR2B11 | Osteoporosis, Postmenopausal | 9.61 |
| PSORS1C1 | Osteoporosis, Postmenopausal | 9.61 |
| RNU11 | Osteoporosis, Postmenopausal | 9.61 |
| SPRR2C | Osteoporosis, Postmenopausal | 9.61 |
| SSTR5-AS1 | Osteoporosis, Postmenopausal | 9.61 |
| ST20 | Osteoporosis, Postmenopausal | 9.61 |
| WFDC13 | Osteoporosis, Postmenopausal | 9.61 |
| ZNF182 | Osteoporosis, Postmenopausal | 9.61 |
| GUSBP11 | Osteoporosis, Postmenopausal | 9.6 |
| LNP1 | Osteoporosis, Postmenopausal | 9.6 |
| NES | Osteoporosis, Postmenopausal | 9.6 |
| PHGR1 | Osteoporosis, Postmenopausal | 9.59 |
| IGF2R | Osteoporosis, Postmenopausal | 9.58 |
| MIR667 | Osteoporosis, Postmenopausal | 9.58 |
| NR4A3 | Osteoporosis, Postmenopausal | 9.58 |
| PGD | Osteoporosis, Postmenopausal | 9.58 |
| THBD | Osteoporosis, Postmenopausal | 9.58 |
| ZNF611 | Osteoporosis, Postmenopausal | 9.58 |
| ABCG1 | Osteoporosis, Postmenopausal | 9.57 |
| ERRFI1 | Osteoporosis, Postmenopausal | 9.56 |
| FYN | Osteoporosis, Postmenopausal | 9.56 |
| GDNF | Osteoporosis, Postmenopausal | 9.56 |
| NT5E | Osteoporosis, Postmenopausal | 9.56 |
| SERPINH1B | Osteoporosis, Postmenopausal | 9.56 |
| VEGFC | Osteoporosis, Postmenopausal | 9.56 |
| MTHFD2 | Osteoporosis, Postmenopausal | 9.55 |
| ADORA1 | Osteoporosis, Postmenopausal | 9.54 |
| COL1A1A | Osteoporosis, Postmenopausal | 9.54 |
| FABP3 | Osteoporosis, Postmenopausal | 9.54 |
| IL24 | Osteoporosis, Postmenopausal | 9.54 |
| NEFH | Osteoporosis, Postmenopausal | 9.54 |
| CREM | Osteoporosis, Postmenopausal | 9.53 |
| EGR2 | Osteoporosis, Postmenopausal | 9.53 |
| PKLR | Osteoporosis, Postmenopausal | 9.53 |
| STAT5B | Osteoporosis, Postmenopausal | 9.53 |
| CD55B | Osteoporosis, Postmenopausal | 9.52 |
| OL-VIT1 | Osteoporosis, Postmenopausal | 9.52 |
| PRKCDBP | Osteoporosis, Postmenopausal | 9.52 |
| APRG1 | Osteoporosis, Postmenopausal | 9.51 |
| CS | Osteoporosis, Postmenopausal | 9.51 |
| FAM86EP | Osteoporosis, Postmenopausal | 9.51 |
| IDH1 | Osteoporosis, Postmenopausal | 9.51 |
| LINC00052 | Osteoporosis, Postmenopausal | 9.51 |
| MAGEA9 | Osteoporosis, Postmenopausal | 9.51 |
| MOGAT3 | Osteoporosis, Postmenopausal | 9.51 |
| OR51Q1 | Osteoporosis, Postmenopausal | 9.51 |
| OSER1-DT | Osteoporosis, Postmenopausal | 9.51 |
| TCRB | Osteoporosis, Postmenopausal | 9.51 |
| TUBA3D | Osteoporosis, Postmenopausal | 9.51 |
| ZNF135 | Osteoporosis, Postmenopausal | 9.51 |
| ZNF700 | Osteoporosis, Postmenopausal | 9.51 |
| VPS24 | Osteoporosis, Postmenopausal | 9.5 |
| GABRG1 | Osteoporosis, Postmenopausal | 9.49 |
| MAPDA | Osteoporosis, Postmenopausal | 9.49 |
| MIR652 | Osteoporosis, Postmenopausal | 9.49 |
| MTHFR | Osteoporosis, Postmenopausal | 9.49 |
| SFRS3 | Osteoporosis, Postmenopausal | 9.49 |
| TTN.1 | Osteoporosis, Postmenopausal | 9.49 |
| ZNF576 | Osteoporosis, Postmenopausal | 9.49 |
| CES2 | Osteoporosis, Postmenopausal | 9.47 |
| FABP5 | Osteoporosis, Postmenopausal | 9.47 |
| SELENOP | Osteoporosis, Postmenopausal | 9.46 |
| CXCL9 | Osteoporosis, Postmenopausal | 9.44 |
| GATA3 | Osteoporosis, Postmenopausal | 9.44 |
| ARHGAP5-AS1 | Osteoporosis, Postmenopausal | 9.43 |
| CFAP206 | Osteoporosis, Postmenopausal | 9.43 |
| COX7B2 | Osteoporosis, Postmenopausal | 9.43 |
| MINAR1 | Osteoporosis, Postmenopausal | 9.43 |
| PRG3 | Osteoporosis, Postmenopausal | 9.43 |
| VTG4 | Osteoporosis, Postmenopausal | 9.43 |
| VTG5 | Osteoporosis, Postmenopausal | 9.43 |
| CCL16 | Osteoporosis, Postmenopausal | 9.42 |
| KRT33A | Osteoporosis, Postmenopausal | 9.42 |
| MIR16-1 | Osteoporosis, Postmenopausal | 9.42 |
| MIR302B | Osteoporosis, Postmenopausal | 9.42 |
| MIR434 | Osteoporosis, Postmenopausal | 9.42 |
| OR2H2 | Osteoporosis, Postmenopausal | 9.42 |
| RIPK1 | Osteoporosis, Postmenopausal | 9.42 |
| RNASE7 | Osteoporosis, Postmenopausal | 9.42 |
| SEC1 | Osteoporosis, Postmenopausal | 9.42 |
| ZNF222 | Osteoporosis, Postmenopausal | 9.42 |
| IL33 | Osteoporosis, Postmenopausal | 9.39 |
| CCL7 | Osteoporosis, Postmenopausal | 9.38 |
| DPYD | Osteoporosis, Postmenopausal | 9.38 |
| CDRT4 | Osteoporosis, Postmenopausal | 9.36 |
| EREG | Osteoporosis, Postmenopausal | 9.36 |
| FGB | Osteoporosis, Postmenopausal | 9.36 |
| ODAM | Osteoporosis, Postmenopausal | 9.36 |
| ACSL4 | Osteoporosis, Postmenopausal | 9.35 |
| KRT28 | Osteoporosis, Postmenopausal | 9.35 |
| OTULIN | Osteoporosis, Postmenopausal | 9.35 |
| ZNF530 | Osteoporosis, Postmenopausal | 9.35 |
| C8ORF82 | Osteoporosis, Postmenopausal | 9.34 |
| CST4 | Osteoporosis, Postmenopausal | 9.34 |
| MIR301A | Osteoporosis, Postmenopausal | 9.34 |
| MIR326 | Osteoporosis, Postmenopausal | 9.34 |
| MST1L | Osteoporosis, Postmenopausal | 9.34 |
| NFE2L2B | Osteoporosis, Postmenopausal | 9.34 |
| PLA2G4D | Osteoporosis, Postmenopausal | 9.34 |
| APEX1 | Osteoporosis, Postmenopausal | 9.33 |
| DIMT1L | Osteoporosis, Postmenopausal | 9.33 |
| MCM5 | Osteoporosis, Postmenopausal | 9.33 |
| FGA | Osteoporosis, Postmenopausal | 9.31 |
| ISG15 | Osteoporosis, Postmenopausal | 9.31 |
| MT2 | Osteoporosis, Postmenopausal | 9.3 |
| ABCB1B | Osteoporosis, Postmenopausal | 9.29 |
| ELOVL6 | Osteoporosis, Postmenopausal | 9.29 |
| IRF1 | Osteoporosis, Postmenopausal | 9.29 |
| ACADM | Osteoporosis, Postmenopausal | 9.27 |
| AGXTB | Osteoporosis, Postmenopausal | 9.27 |
| CLDN7A | Osteoporosis, Postmenopausal | 9.27 |
| FBXL9P | Osteoporosis, Postmenopausal | 9.27 |
| IGH-6 | Osteoporosis, Postmenopausal | 9.27 |
| KRT33B | Osteoporosis, Postmenopausal | 9.27 |
| LRFN2 | Osteoporosis, Postmenopausal | 9.27 |
| RLN3 | Osteoporosis, Postmenopausal | 9.27 |
| TBXT | Osteoporosis, Postmenopausal | 9.27 |
| C15ORF62 | Osteoporosis, Postmenopausal | 9.26 |
| C2ORF76 | Osteoporosis, Postmenopausal | 9.26 |
| FGG | Osteoporosis, Postmenopausal | 9.26 |
| LRRC37A3 | Osteoporosis, Postmenopausal | 9.26 |
| MIR203A | Osteoporosis, Postmenopausal | 9.26 |
| MIR369 | Osteoporosis, Postmenopausal | 9.26 |
| MRGPRX3 | Osteoporosis, Postmenopausal | 9.26 |
| OPN1MW2 | Osteoporosis, Postmenopausal | 9.26 |
| PLA2G2F | Osteoporosis, Postmenopausal | 9.26 |
| SIRPB2 | Osteoporosis, Postmenopausal | 9.25 |
| CHAC1 | Osteoporosis, Postmenopausal | 9.24 |
| CRH | Osteoporosis, Postmenopausal | 9.24 |
| RARB | Osteoporosis, Postmenopausal | 9.24 |
| C1ORF162 | Osteoporosis, Postmenopausal | 9.22 |
| DHFR | Osteoporosis, Postmenopausal | 9.22 |
| EFCAB8 | Osteoporosis, Postmenopausal | 9.21 |
| PHLDA1 | Osteoporosis, Postmenopausal | 9.21 |
| APOL7C | Osteoporosis, Postmenopausal | 9.2 |
| DHRS13A.2 | Osteoporosis, Postmenopausal | 9.2 |
| C2ORF72 | Osteoporosis, Postmenopausal | 9.19 |
| KLF6 | Osteoporosis, Postmenopausal | 9.19 |
| SLC35F4 | Osteoporosis, Postmenopausal | 9.19 |
| SPIN3 | Osteoporosis, Postmenopausal | 9.19 |
| THRSP | Osteoporosis, Postmenopausal | 9.19 |
| ZNF439 | Osteoporosis, Postmenopausal | 9.19 |
| AMY2B | Osteoporosis, Postmenopausal | 9.18 |
| KRT32 | Osteoporosis, Postmenopausal | 9.18 |
| MIR638 | Osteoporosis, Postmenopausal | 9.18 |
| MT1A | Osteoporosis, Postmenopausal | 9.18 |
| PROP1 | Osteoporosis, Postmenopausal | 9.18 |
| SLC52A1 | Osteoporosis, Postmenopausal | 9.18 |
| TMEM61 | Osteoporosis, Postmenopausal | 9.18 |
| ZNF776 | Osteoporosis, Postmenopausal | 9.18 |
| ALAD | Osteoporosis, Postmenopausal | 9.17 |
| NR4A2 | Osteoporosis, Postmenopausal | 9.17 |
| COX4 | Osteoporosis, Postmenopausal | 9.16 |
| PLA2G4A | Osteoporosis, Postmenopausal | 9.16 |
| ALT | Osteoporosis, Postmenopausal | 9.15 |
| SLC26A8 | Osteoporosis, Postmenopausal | 9.14 |
| ITPRID1 | Osteoporosis, Postmenopausal | 9.13 |
| MIR136 | Osteoporosis, Postmenopausal | 9.13 |
| THAP8 | Osteoporosis, Postmenopausal | 9.13 |
| C17ORF100 | Osteoporosis, Postmenopausal | 9.12 |
| GRIN1 | Osteoporosis, Postmenopausal | 9.12 |
| PINK1 | Osteoporosis, Postmenopausal | 9.11 |
| C18ORF54 | Osteoporosis, Postmenopausal | 9.1 |
| CES2H | Osteoporosis, Postmenopausal | 9.1 |
| ZNF420 | Osteoporosis, Postmenopausal | 9.1 |
| OGG1 | Osteoporosis, Postmenopausal | 9.09 |
| SREBF2 | Osteoporosis, Postmenopausal | 9.09 |
| CPT2 | Osteoporosis, Postmenopausal | 9.07 |
| SPRING1 | Osteoporosis, Postmenopausal | 9.07 |
| ALOX12E | Osteoporosis, Postmenopausal | 9.06 |
| CYCT | Osteoporosis, Postmenopausal | 9.06 |
| TRIM30D | Osteoporosis, Postmenopausal | 9.06 |
| VIT-6 | Osteoporosis, Postmenopausal | 9.06 |
| WDR93 | Osteoporosis, Postmenopausal | 9.06 |
| C11ORF52 | Osteoporosis, Postmenopausal | 9.05 |
| CCL15 | Osteoporosis, Postmenopausal | 9.05 |
| KCNH5 | Osteoporosis, Postmenopausal | 9.05 |
| PLAC8L1 | Osteoporosis, Postmenopausal | 9.05 |
| ZNF684 | Osteoporosis, Postmenopausal | 9.05 |
| ACTMAP | Osteoporosis, Postmenopausal | 9.03 |
| CATIP | Osteoporosis, Postmenopausal | 9.03 |
| KRT36 | Osteoporosis, Postmenopausal | 9.03 |
| MIR501 | Osteoporosis, Postmenopausal | 9.03 |
| TMC3 | Osteoporosis, Postmenopausal | 9.03 |
| TMEM215 | Osteoporosis, Postmenopausal | 9.03 |
| TMEM89 | Osteoporosis, Postmenopausal | 9.03 |
| ZDHHC8BP | Osteoporosis, Postmenopausal | 9.03 |
| ZNF264 | Osteoporosis, Postmenopausal | 9.03 |
| ZNF846 | Osteoporosis, Postmenopausal | 9.03 |
| CTSL | Osteoporosis, Postmenopausal | 9.02 |
| CYP2A7 | Osteoporosis, Postmenopausal | 9.01 |
| FAM72D | Osteoporosis, Postmenopausal | 9.01 |
| RFX8 | Osteoporosis, Postmenopausal | 9.01 |
| TCP11 | Osteoporosis, Postmenopausal | 9 |
| CCNG1 | Osteoporosis, Postmenopausal | 8.99 |
| LAIR2 | Osteoporosis, Postmenopausal | 8.99 |
| LGALS5 | Osteoporosis, Postmenopausal | 8.99 |
| LINGO4 | Osteoporosis, Postmenopausal | 8.99 |
| LUZP2 | Osteoporosis, Postmenopausal | 8.99 |
| MIR760 | Osteoporosis, Postmenopausal | 8.99 |
| PIK3R3A | Osteoporosis, Postmenopausal | 8.99 |
| STFA2 | Osteoporosis, Postmenopausal | 8.99 |
| ZG16B | Osteoporosis, Postmenopausal | 8.99 |
| ZNF225 | Osteoporosis, Postmenopausal | 8.99 |
| FDFT1 | Osteoporosis, Postmenopausal | 8.98 |
| CA7 | Osteoporosis, Postmenopausal | 8.97 |
| CPO | Osteoporosis, Postmenopausal | 8.97 |
| KRTAP17-1 | Osteoporosis, Postmenopausal | 8.97 |
| MIF | Osteoporosis, Postmenopausal | 8.97 |
| MIR503HG | Osteoporosis, Postmenopausal | 8.97 |
| MIR582 | Osteoporosis, Postmenopausal | 8.97 |
| OR8B8 | Osteoporosis, Postmenopausal | 8.97 |
| OTOP2 | Osteoporosis, Postmenopausal | 8.97 |
| PVRIG | Osteoporosis, Postmenopausal | 8.97 |
| ZNF524 | Osteoporosis, Postmenopausal | 8.97 |
| LINC00312 | Osteoporosis, Postmenopausal | 8.96 |
| BMX | Osteoporosis, Postmenopausal | 8.95 |
| FAM156A | Osteoporosis, Postmenopausal | 8.95 |
| NLRP5 | Osteoporosis, Postmenopausal | 8.95 |
| MIR337 | Osteoporosis, Postmenopausal | 8.94 |
| SERPINB12 | Osteoporosis, Postmenopausal | 8.94 |
| SPMIP8 | Osteoporosis, Postmenopausal | 8.94 |
| ABCC11 | Osteoporosis, Postmenopausal | 8.93 |
| APOBA | Osteoporosis, Postmenopausal | 8.93 |
| LY6G6E | Osteoporosis, Postmenopausal | 8.93 |
| P2RY11 | Osteoporosis, Postmenopausal | 8.93 |
| SPRR2E | Osteoporosis, Postmenopausal | 8.93 |
| ZNF552 | Osteoporosis, Postmenopausal | 8.93 |
| LSS | Osteoporosis, Postmenopausal | 8.92 |
| MIR411 | Osteoporosis, Postmenopausal | 8.92 |
| NICOL1 | Osteoporosis, Postmenopausal | 8.92 |
| OTOS | Osteoporosis, Postmenopausal | 8.92 |
| TMEM187 | Osteoporosis, Postmenopausal | 8.92 |
| ACTRT3 | Osteoporosis, Postmenopausal | 8.91 |
| BRDT | Osteoporosis, Postmenopausal | 8.91 |
| DHFR2 | Osteoporosis, Postmenopausal | 8.91 |
| MKNK2B | Osteoporosis, Postmenopausal | 8.91 |
| NFKBIAA | Osteoporosis, Postmenopausal | 8.91 |
| DNM1L | Osteoporosis, Postmenopausal | 8.9 |
| ERVW-1 | Osteoporosis, Postmenopausal | 8.9 |
| KRTAP3-1 | Osteoporosis, Postmenopausal | 8.9 |
| SH2D6 | Osteoporosis, Postmenopausal | 8.9 |
| SLC38A8 | Osteoporosis, Postmenopausal | 8.9 |
| ZNF426 | Osteoporosis, Postmenopausal | 8.9 |
| ZNF444 | Osteoporosis, Postmenopausal | 8.9 |
| CNTNAP4 | Osteoporosis, Postmenopausal | 8.89 |
| RLN2 | Osteoporosis, Postmenopausal | 8.89 |
| SLC46A2 | Osteoporosis, Postmenopausal | 8.89 |
| UGT1A6 | Osteoporosis, Postmenopausal | 8.89 |
| ZNF551 | Osteoporosis, Postmenopausal | 8.89 |
| ATP4B | Osteoporosis, Postmenopausal | 8.88 |
| BARHL2 | Osteoporosis, Postmenopausal | 8.88 |
| CATSPER4 | Osteoporosis, Postmenopausal | 8.88 |
| ANKRD1B | Osteoporosis, Postmenopausal | 8.87 |
| IGHG | Osteoporosis, Postmenopausal | 8.87 |
| KLRE1 | Osteoporosis, Postmenopausal | 8.87 |
| LRRC43 | Osteoporosis, Postmenopausal | 8.87 |
| NPIPB3 | Osteoporosis, Postmenopausal | 8.87 |
| TRMT61B | Osteoporosis, Postmenopausal | 8.87 |
| VSIG2 | Osteoporosis, Postmenopausal | 8.87 |
| ZFP275 | Osteoporosis, Postmenopausal | 8.87 |
| ZFP958 | Osteoporosis, Postmenopausal | 8.87 |
| AKR1B1 | Osteoporosis, Postmenopausal | 8.86 |
| CR1 | Osteoporosis, Postmenopausal | 8.86 |
| C3ORF70 | Osteoporosis, Postmenopausal | 8.84 |
| GLI4 | Osteoporosis, Postmenopausal | 8.84 |
| IL17C | Osteoporosis, Postmenopausal | 8.84 |
| MCM3AP-AS1 | Osteoporosis, Postmenopausal | 8.84 |
| MIR24-1 | Osteoporosis, Postmenopausal | 8.84 |
| RBBP8NL | Osteoporosis, Postmenopausal | 8.84 |
| ZNF658 | Osteoporosis, Postmenopausal | 8.84 |
| C9ORF85 | Osteoporosis, Postmenopausal | 8.83 |
| ANKRD53 | Osteoporosis, Postmenopausal | 8.82 |
| C3ORF38 | Osteoporosis, Postmenopausal | 8.82 |
| CALR | Osteoporosis, Postmenopausal | 8.82 |
| KIAA1586 | Osteoporosis, Postmenopausal | 8.82 |
| MGST1 | Osteoporosis, Postmenopausal | 8.82 |
| REG3A | Osteoporosis, Postmenopausal | 8.82 |
| ZNF775 | Osteoporosis, Postmenopausal | 8.82 |
| ALG10 | Osteoporosis, Postmenopausal | 8.81 |
| C1ORF216 | Osteoporosis, Postmenopausal | 8.81 |
| C9ORF152 | Osteoporosis, Postmenopausal | 8.81 |
| CD300C2 | Osteoporosis, Postmenopausal | 8.81 |
| CLDN16 | Osteoporosis, Postmenopausal | 8.81 |
| ENO4 | Osteoporosis, Postmenopausal | 8.81 |
| GARIN5A | Osteoporosis, Postmenopausal | 8.81 |
| GH | Osteoporosis, Postmenopausal | 8.81 |
| IGH-VJ558 | Osteoporosis, Postmenopausal | 8.81 |
| KRT27 | Osteoporosis, Postmenopausal | 8.81 |
| LRGUK | Osteoporosis, Postmenopausal | 8.81 |
| OAS1F | Osteoporosis, Postmenopausal | 8.81 |
| WAP | Osteoporosis, Postmenopausal | 8.81 |
| KIAA0513 | Osteoporosis, Postmenopausal | 8.8 |
| NRG1 | Osteoporosis, Postmenopausal | 8.8 |
| C4ORF33 | Osteoporosis, Postmenopausal | 8.79 |
| GPR63 | Osteoporosis, Postmenopausal | 8.79 |
| MIR139 | Osteoporosis, Postmenopausal | 8.79 |
| APOBEC3F | Osteoporosis, Postmenopausal | 8.78 |
| CCDC191 | Osteoporosis, Postmenopausal | 8.78 |
| CLTCL1 | Osteoporosis, Postmenopausal | 8.78 |
| CRYAB | Osteoporosis, Postmenopausal | 8.78 |
| GJD3 | Osteoporosis, Postmenopausal | 8.78 |
| INSYN2A | Osteoporosis, Postmenopausal | 8.78 |
| IZUMO1R | Osteoporosis, Postmenopausal | 8.78 |
| MIR615 | Osteoporosis, Postmenopausal | 8.78 |
| MS4A10 | Osteoporosis, Postmenopausal | 8.78 |
| OTUD7A | Osteoporosis, Postmenopausal | 8.78 |
| SERINC4 | Osteoporosis, Postmenopausal | 8.78 |
| SERPINB13 | Osteoporosis, Postmenopausal | 8.78 |
| ZNF511 | Osteoporosis, Postmenopausal | 8.78 |
| ALPG | Osteoporosis, Postmenopausal | 8.76 |
| CLEC4A2 | Osteoporosis, Postmenopausal | 8.76 |
| HTR1F | Osteoporosis, Postmenopausal | 8.76 |
| MIR351 | Osteoporosis, Postmenopausal | 8.76 |
| VTG3 | Osteoporosis, Postmenopausal | 8.76 |
| CACNG5 | Osteoporosis, Postmenopausal | 8.75 |
| METTL25B | Osteoporosis, Postmenopausal | 8.75 |
| ZFP69B | Osteoporosis, Postmenopausal | 8.75 |
| GPR143 | Osteoporosis, Postmenopausal | 8.74 |
| H2BC10 | Osteoporosis, Postmenopausal | 8.74 |
| OMG | Osteoporosis, Postmenopausal | 8.73 |
| PRP4K | Osteoporosis, Postmenopausal | 8.73 |
| COX8C | Osteoporosis, Postmenopausal | 8.72 |
| CYP2B10 | Osteoporosis, Postmenopausal | 8.72 |
| LINC01588 | Osteoporosis, Postmenopausal | 8.72 |
| MIR671 | Osteoporosis, Postmenopausal | 8.72 |
| PLIN2 | Osteoporosis, Postmenopausal | 8.72 |
| RAET1L | Osteoporosis, Postmenopausal | 8.72 |
| SANBR | Osteoporosis, Postmenopausal | 8.72 |
| GADD45BA | Osteoporosis, Postmenopausal | 8.71 |
| MIR133B | Osteoporosis, Postmenopausal | 8.71 |
| PLEKHS1 | Osteoporosis, Postmenopausal | 8.71 |
| ADGRG7 | Osteoporosis, Postmenopausal | 8.7 |
| CD209F | Osteoporosis, Postmenopausal | 8.7 |
| EIF4EBP3L | Osteoporosis, Postmenopausal | 8.7 |
| ER | Osteoporosis, Postmenopausal | 8.7 |
| IGH | Osteoporosis, Postmenopausal | 8.7 |
| PCDHB2 | Osteoporosis, Postmenopausal | 8.7 |
| PHAF1 | Osteoporosis, Postmenopausal | 8.7 |
| SECTM1A | Osteoporosis, Postmenopausal | 8.7 |
| SNHG4 | Osteoporosis, Postmenopausal | 8.7 |
| SSUH2 | Osteoporosis, Postmenopausal | 8.7 |
| VSTM2A | Osteoporosis, Postmenopausal | 8.7 |
| C9ORF40 | Osteoporosis, Postmenopausal | 8.69 |
| GAL3ST4 | Osteoporosis, Postmenopausal | 8.69 |
| TXN | Osteoporosis, Postmenopausal | 8.69 |
| ANOS1 | Osteoporosis, Postmenopausal | 8.68 |
| ZNF37A | Osteoporosis, Postmenopausal | 8.67 |
| HSP27 | Osteoporosis, Postmenopausal | 8.66 |
| LMAN1L | Osteoporosis, Postmenopausal | 8.66 |
| MIR339 | Osteoporosis, Postmenopausal | 8.66 |
| PILRB | Osteoporosis, Postmenopausal | 8.66 |
| SAMTOR | Osteoporosis, Postmenopausal | 8.66 |
| SOCS3A | Osteoporosis, Postmenopausal | 8.66 |
| TLCD3B | Osteoporosis, Postmenopausal | 8.66 |
| TMEM178 | Osteoporosis, Postmenopausal | 8.66 |
| TMEM198 | Osteoporosis, Postmenopausal | 8.66 |
| UCN2 | Osteoporosis, Postmenopausal | 8.66 |
| ZFP69 | Osteoporosis, Postmenopausal | 8.66 |
| ZNF121 | Osteoporosis, Postmenopausal | 8.66 |
| APOBB.1 | Osteoporosis, Postmenopausal | 8.65 |
| CABP4 | Osteoporosis, Postmenopausal | 8.65 |
| CLCN1 | Osteoporosis, Postmenopausal | 8.65 |
| DNAJB3 | Osteoporosis, Postmenopausal | 8.65 |
| HSF1 | Osteoporosis, Postmenopausal | 8.65 |
| IGLV1 | Osteoporosis, Postmenopausal | 8.65 |
| MCEMP1 | Osteoporosis, Postmenopausal | 8.65 |
| SIRPB1 | Osteoporosis, Postmenopausal | 8.65 |
| CXCL8A | Osteoporosis, Postmenopausal | 8.64 |
| OTUD3 | Osteoporosis, Postmenopausal | 8.64 |
| HHIPL2 | Osteoporosis, Postmenopausal | 8.63 |
| CRYGN | Osteoporosis, Postmenopausal | 8.62 |
| FBXL13 | Osteoporosis, Postmenopausal | 8.62 |
| FHIP2A | Osteoporosis, Postmenopausal | 8.62 |
| KLRC2 | Osteoporosis, Postmenopausal | 8.62 |
| NCMAP | Osteoporosis, Postmenopausal | 8.62 |
| TECPR2 | Osteoporosis, Postmenopausal | 8.62 |
| BLTP2 | Osteoporosis, Postmenopausal | 8.61 |
| C14ORF93 | Osteoporosis, Postmenopausal | 8.61 |
| CLDN19 | Osteoporosis, Postmenopausal | 8.61 |
| FBXW11 | Osteoporosis, Postmenopausal | 8.61 |
| GPR150 | Osteoporosis, Postmenopausal | 8.61 |
| MUCL1 | Osteoporosis, Postmenopausal | 8.61 |
| SAAL1 | Osteoporosis, Postmenopausal | 8.61 |
| C5ORF34 | Osteoporosis, Postmenopausal | 8.6 |
| CAR6 | Osteoporosis, Postmenopausal | 8.6 |
| DBF4B | Osteoporosis, Postmenopausal | 8.6 |
| FCNB | Osteoporosis, Postmenopausal | 8.6 |
| KCNA7 | Osteoporosis, Postmenopausal | 8.6 |
| KLRA22 | Osteoporosis, Postmenopausal | 8.6 |
| LIPF | Osteoporosis, Postmenopausal | 8.6 |
| PINLYP | Osteoporosis, Postmenopausal | 8.6 |
| STFA3 | Osteoporosis, Postmenopausal | 8.6 |
| C14ORF28 | Osteoporosis, Postmenopausal | 8.59 |
| CCDC62 | Osteoporosis, Postmenopausal | 8.59 |
| LINC00261 | Osteoporosis, Postmenopausal | 8.59 |
| NEU4 | Osteoporosis, Postmenopausal | 8.57 |
| SERPINA1E | Osteoporosis, Postmenopausal | 8.57 |
| SLC34A3 | Osteoporosis, Postmenopausal | 8.57 |
| ZKSCAN2 | Osteoporosis, Postmenopausal | 8.57 |
| DEFB4A | Osteoporosis, Postmenopausal | 8.56 |
| ENTREP2 | Osteoporosis, Postmenopausal | 8.56 |
| MIR1915HG | Osteoporosis, Postmenopausal | 8.56 |
| MIR542 | Osteoporosis, Postmenopausal | 8.56 |
| MIR92B | Osteoporosis, Postmenopausal | 8.56 |
| NKX1-2 | Osteoporosis, Postmenopausal | 8.56 |
| SOD-3 | Osteoporosis, Postmenopausal | 8.56 |
| UGT2B11 | Osteoporosis, Postmenopausal | 8.56 |
| WFDC12 | Osteoporosis, Postmenopausal | 8.56 |
| ZFP106 | Osteoporosis, Postmenopausal | 8.56 |
| ZNF33B | Osteoporosis, Postmenopausal | 8.56 |
| ZNF557 | Osteoporosis, Postmenopausal | 8.56 |
| BNIP3 | Osteoporosis, Postmenopausal | 8.55 |
| DND1 | Osteoporosis, Postmenopausal | 8.55 |
| ENAM | Osteoporosis, Postmenopausal | 8.55 |
| SAXO2 | Osteoporosis, Postmenopausal | 8.55 |
| SULT1C2A | Osteoporosis, Postmenopausal | 8.55 |
| CENPC | Osteoporosis, Postmenopausal | 8.54 |
| FBXO10 | Osteoporosis, Postmenopausal | 8.54 |
| NOP9 | Osteoporosis, Postmenopausal | 8.54 |
| ZNF711 | Osteoporosis, Postmenopausal | 8.54 |
| SHLD1 | Osteoporosis, Postmenopausal | 8.53 |
| DAF-16 | Osteoporosis, Postmenopausal | 8.52 |
| FAM131C | Osteoporosis, Postmenopausal | 8.52 |
| NKX2.5 | Osteoporosis, Postmenopausal | 8.52 |
| PRIMPOL | Osteoporosis, Postmenopausal | 8.52 |
| TMEM126B | Osteoporosis, Postmenopausal | 8.52 |
| EBF4 | Osteoporosis, Postmenopausal | 8.51 |
| FOXI2 | Osteoporosis, Postmenopausal | 8.51 |
| HBB-BS | Osteoporosis, Postmenopausal | 8.51 |
| KLRA1 | Osteoporosis, Postmenopausal | 8.51 |
| LCE3D | Osteoporosis, Postmenopausal | 8.51 |
| MIR149 | Osteoporosis, Postmenopausal | 8.51 |
| NSUN7 | Osteoporosis, Postmenopausal | 8.51 |
| RLIG1 | Osteoporosis, Postmenopausal | 8.51 |
| RNF135 | Osteoporosis, Postmenopausal | 8.51 |
| RNF146 | Osteoporosis, Postmenopausal | 8.51 |
| S100Z | Osteoporosis, Postmenopausal | 8.51 |
| TRABD2A | Osteoporosis, Postmenopausal | 8.51 |
| TRIM15 | Osteoporosis, Postmenopausal | 8.51 |
| ZMAT2 | Osteoporosis, Postmenopausal | 8.51 |
| ZNF397 | Osteoporosis, Postmenopausal | 8.51 |
| ZSCAN10 | Osteoporosis, Postmenopausal | 8.51 |
| GSTA4 | Osteoporosis, Postmenopausal | 8.5 |
| RGS22 | Osteoporosis, Postmenopausal | 8.5 |
| SPRR2B | Osteoporosis, Postmenopausal | 8.5 |
| SPRR2G | Osteoporosis, Postmenopausal | 8.5 |
| TCTE1 | Osteoporosis, Postmenopausal | 8.5 |
| TREML4 | Osteoporosis, Postmenopausal | 8.5 |
| YAE1 | Osteoporosis, Postmenopausal | 8.5 |
| ZBED2 | Osteoporosis, Postmenopausal | 8.5 |
| ZFP386 | Osteoporosis, Postmenopausal | 8.5 |
| CYP4F22 | Osteoporosis, Postmenopausal | 8.49 |
| GPR50 | Osteoporosis, Postmenopausal | 8.49 |
| SLA2 | Osteoporosis, Postmenopausal | 8.49 |
| TMEM91 | Osteoporosis, Postmenopausal | 8.49 |
| UCA1 | Osteoporosis, Postmenopausal | 8.49 |
| WDR5B | Osteoporosis, Postmenopausal | 8.49 |
| GDPGP1 | Osteoporosis, Postmenopausal | 8.48 |
| CIPC | Osteoporosis, Postmenopausal | 8.47 |
| LRRC25 | Osteoporosis, Postmenopausal | 8.47 |
| MIR342 | Osteoporosis, Postmenopausal | 8.47 |
| NOX4 | Osteoporosis, Postmenopausal | 8.47 |
| SNORC | Osteoporosis, Postmenopausal | 8.47 |
| TUBA3A | Osteoporosis, Postmenopausal | 8.47 |
| APOBEC2 | Osteoporosis, Postmenopausal | 8.46 |
| CAR12 | Osteoporosis, Postmenopausal | 8.46 |
| CARD16 | Osteoporosis, Postmenopausal | 8.46 |
| CHADL | Osteoporosis, Postmenopausal | 8.46 |
| CZIB | Osteoporosis, Postmenopausal | 8.46 |
| DEFA5 | Osteoporosis, Postmenopausal | 8.46 |
| FGF22 | Osteoporosis, Postmenopausal | 8.46 |
| IFI202B | Osteoporosis, Postmenopausal | 8.46 |
| LRTOMT | Osteoporosis, Postmenopausal | 8.46 |
| MIRLET7C | Osteoporosis, Postmenopausal | 8.46 |
| RIC1 | Osteoporosis, Postmenopausal | 8.46 |
| SPRR2F | Osteoporosis, Postmenopausal | 8.46 |
| TLX1 | Osteoporosis, Postmenopausal | 8.46 |
| TMSB15B | Osteoporosis, Postmenopausal | 8.46 |
| ZNF32 | Osteoporosis, Postmenopausal | 8.46 |
| ANKRD13C-DT | Osteoporosis, Postmenopausal | 8.45 |
| ARK2N | Osteoporosis, Postmenopausal | 8.45 |
| GSDMB | Osteoporosis, Postmenopausal | 8.45 |
| PGBD1 | Osteoporosis, Postmenopausal | 8.45 |
| PTGDR2 | Osteoporosis, Postmenopausal | 8.45 |
| CDH7 | Osteoporosis, Postmenopausal | 8.44 |
| CSMD2 | Osteoporosis, Postmenopausal | 8.44 |
| DYDC2 | Osteoporosis, Postmenopausal | 8.44 |
| NBPF1 | Osteoporosis, Postmenopausal | 8.44 |
| NBPF10 | Osteoporosis, Postmenopausal | 8.44 |
| TTLL10 | Osteoporosis, Postmenopausal | 8.44 |
| ARMC7 | Osteoporosis, Postmenopausal | 8.43 |
| IFI27L1 | Osteoporosis, Postmenopausal | 8.43 |
| IL5 | Osteoporosis, Postmenopausal | 8.43 |
| MIR181C | Osteoporosis, Postmenopausal | 8.43 |
| ZNF44 | Osteoporosis, Postmenopausal | 8.43 |
| C1ORF226 | Osteoporosis, Postmenopausal | 8.41 |
| CCDC40 | Osteoporosis, Postmenopausal | 8.41 |
| EEF1AKMT3 | Osteoporosis, Postmenopausal | 8.41 |
| FAM163B | Osteoporosis, Postmenopausal | 8.41 |
| GP6 | Osteoporosis, Postmenopausal | 8.41 |
| H2-D1 | Osteoporosis, Postmenopausal | 8.41 |
| KCNS1 | Osteoporosis, Postmenopausal | 8.41 |
| KRT25 | Osteoporosis, Postmenopausal | 8.41 |
| MORN3 | Osteoporosis, Postmenopausal | 8.41 |
| MUC15 | Osteoporosis, Postmenopausal | 8.41 |
| PDZD4 | Osteoporosis, Postmenopausal | 8.41 |
| PFKFB4B | Osteoporosis, Postmenopausal | 8.41 |
| RAB40C | Osteoporosis, Postmenopausal | 8.41 |
| RPPH1 | Osteoporosis, Postmenopausal | 8.41 |
| SMIM19 | Osteoporosis, Postmenopausal | 8.41 |
| SPRR2A | Osteoporosis, Postmenopausal | 8.41 |
| TBP | Osteoporosis, Postmenopausal | 8.41 |
| KCNK9 | Osteoporosis, Postmenopausal | 8.4 |
| PDK4 | Osteoporosis, Postmenopausal | 8.4 |
| RIT2 | Osteoporosis, Postmenopausal | 8.4 |
| ZNF641 | Osteoporosis, Postmenopausal | 8.4 |
| LHFPL4 | Osteoporosis, Postmenopausal | 8.39 |
| MIR324 | Osteoporosis, Postmenopausal | 8.39 |
| RBSN | Osteoporosis, Postmenopausal | 8.39 |
| SLC18A3 | Osteoporosis, Postmenopausal | 8.39 |
| TGTP1 | Osteoporosis, Postmenopausal | 8.39 |
| GCNA | Osteoporosis, Postmenopausal | 8.38 |
| ANKMY1 | Osteoporosis, Postmenopausal | 8.37 |
| APOL7A | Osteoporosis, Postmenopausal | 8.37 |
| CAPS | Osteoporosis, Postmenopausal | 8.37 |
| CYP4F1 | Osteoporosis, Postmenopausal | 8.37 |
| DMRT2 | Osteoporosis, Postmenopausal | 8.37 |
| DNTTIP1 | Osteoporosis, Postmenopausal | 8.37 |
| FUT9 | Osteoporosis, Postmenopausal | 8.37 |
| SHKBP1 | Osteoporosis, Postmenopausal | 8.37 |
| SMDT1 | Osteoporosis, Postmenopausal | 8.37 |
| CFAP69 | Osteoporosis, Postmenopausal | 8.36 |
| CNTD1 | Osteoporosis, Postmenopausal | 8.36 |
| DKK4 | Osteoporosis, Postmenopausal | 8.36 |
| EVX1 | Osteoporosis, Postmenopausal | 8.36 |
| FAM83B | Osteoporosis, Postmenopausal | 8.36 |
| FOXD4 | Osteoporosis, Postmenopausal | 8.36 |
| HMOX1A | Osteoporosis, Postmenopausal | 8.36 |
| HOXD11 | Osteoporosis, Postmenopausal | 8.36 |
| HPSE2 | Osteoporosis, Postmenopausal | 8.36 |
| KLK7 | Osteoporosis, Postmenopausal | 8.36 |
| KRT71 | Osteoporosis, Postmenopausal | 8.36 |
| MANEAL | Osteoporosis, Postmenopausal | 8.36 |
| MIR455 | Osteoporosis, Postmenopausal | 8.36 |
| POU1F1 | Osteoporosis, Postmenopausal | 8.36 |
| RAPSN | Osteoporosis, Postmenopausal | 8.36 |
| ZNF616 | Osteoporosis, Postmenopausal | 8.36 |
| FLT3LG | Osteoporosis, Postmenopausal | 8.35 |
| MDFI | Osteoporosis, Postmenopausal | 8.35 |
| CAAP1 | Osteoporosis, Postmenopausal | 8.34 |
| CCL4 | Osteoporosis, Postmenopausal | 8.34 |
| EEF1AKMT2 | Osteoporosis, Postmenopausal | 8.34 |
| NDRG1 | Osteoporosis, Postmenopausal | 8.34 |
| PI3 | Osteoporosis, Postmenopausal | 8.34 |
| ZFPL1 | Osteoporosis, Postmenopausal | 8.34 |
| ATG101 | Osteoporosis, Postmenopausal | 8.33 |
| CCDC184 | Osteoporosis, Postmenopausal | 8.33 |
| CYP2D1 | Osteoporosis, Postmenopausal | 8.33 |
| GIMAP7 | Osteoporosis, Postmenopausal | 8.33 |
| GPR20 | Osteoporosis, Postmenopausal | 8.33 |
| KCNMB1 | Osteoporosis, Postmenopausal | 8.33 |
| MIR98 | Osteoporosis, Postmenopausal | 8.33 |
| MLANA | Osteoporosis, Postmenopausal | 8.33 |
| MYT1L | Osteoporosis, Postmenopausal | 8.33 |
| NACAD | Osteoporosis, Postmenopausal | 8.33 |
| TMEM220 | Osteoporosis, Postmenopausal | 8.33 |
| AP4B1 | Osteoporosis, Postmenopausal | 8.32 |
| ARL6IP6 | Osteoporosis, Postmenopausal | 8.32 |
| C21ORF58 | Osteoporosis, Postmenopausal | 8.32 |
| CHST5 | Osteoporosis, Postmenopausal | 8.32 |
| CLRN3 | Osteoporosis, Postmenopausal | 8.32 |
| ERVFRD-1 | Osteoporosis, Postmenopausal | 8.32 |
| FAM185A | Osteoporosis, Postmenopausal | 8.32 |
| GPR173 | Osteoporosis, Postmenopausal | 8.32 |
| GSTP3 | Osteoporosis, Postmenopausal | 8.32 |
| MS4A4A | Osteoporosis, Postmenopausal | 8.32 |
| PRM2 | Osteoporosis, Postmenopausal | 8.32 |
| RMND5B | Osteoporosis, Postmenopausal | 8.32 |
| SNHG29 | Osteoporosis, Postmenopausal | 8.32 |
| PRSS27 | Osteoporosis, Postmenopausal | 8.31 |
| TMEM26 | Osteoporosis, Postmenopausal | 8.31 |
| TRAPPC8 | Osteoporosis, Postmenopausal | 8.31 |
| ZNF706 | Osteoporosis, Postmenopausal | 8.31 |
| CCDC152 | Osteoporosis, Postmenopausal | 8.3 |
| ICE1 | Osteoporosis, Postmenopausal | 8.3 |
| AARD | Osteoporosis, Postmenopausal | 8.29 |
| SCARB1 | Osteoporosis, Postmenopausal | 8.29 |
| WDR48 | Osteoporosis, Postmenopausal | 8.29 |
| ACSM2A | Osteoporosis, Postmenopausal | 8.28 |
| AGR3 | Osteoporosis, Postmenopausal | 8.28 |
| C21ORF91 | Osteoporosis, Postmenopausal | 8.28 |
| CAPN12 | Osteoporosis, Postmenopausal | 8.28 |
| CCDC9B | Osteoporosis, Postmenopausal | 8.28 |
| CPLX3 | Osteoporosis, Postmenopausal | 8.28 |
| CYP2G1 | Osteoporosis, Postmenopausal | 8.28 |
| ERN2 | Osteoporosis, Postmenopausal | 8.28 |
| FGF17 | Osteoporosis, Postmenopausal | 8.28 |
| HLA-DQA1 | Osteoporosis, Postmenopausal | 8.28 |
| HOXC8 | Osteoporosis, Postmenopausal | 8.28 |
| KANSL3 | Osteoporosis, Postmenopausal | 8.28 |
| NKPD1 | Osteoporosis, Postmenopausal | 8.28 |
| RIMBP3 | Osteoporosis, Postmenopausal | 8.28 |
| SDHAF4 | Osteoporosis, Postmenopausal | 8.28 |
| SLFN3 | Osteoporosis, Postmenopausal | 8.28 |
| STUM | Osteoporosis, Postmenopausal | 8.28 |
| TRIM56 | Osteoporosis, Postmenopausal | 8.28 |
| TSPAN10 | Osteoporosis, Postmenopausal | 8.28 |
| VSIG1 | Osteoporosis, Postmenopausal | 8.28 |
| ZNF57 | Osteoporosis, Postmenopausal | 8.28 |
| ANKRD36B | Osteoporosis, Postmenopausal | 8.27 |
| DIPK1C | Osteoporosis, Postmenopausal | 8.27 |
| GSTM2 | Osteoporosis, Postmenopausal | 8.27 |
| KLHDC7B | Osteoporosis, Postmenopausal | 8.27 |
| ZBTB12 | Osteoporosis, Postmenopausal | 8.27 |
| DLX6 | Osteoporosis, Postmenopausal | 8.26 |
| HEPACAM | Osteoporosis, Postmenopausal | 8.26 |
| ILRUN | Osteoporosis, Postmenopausal | 8.26 |
| KCNT2 | Osteoporosis, Postmenopausal | 8.26 |
| NHERF4 | Osteoporosis, Postmenopausal | 8.26 |
| RAB7 | Osteoporosis, Postmenopausal | 8.26 |
| TRIM16L | Osteoporosis, Postmenopausal | 8.26 |
| OTUD5 | Osteoporosis, Postmenopausal | 8.25 |
| RADX | Osteoporosis, Postmenopausal | 8.25 |
| AKR1C6 | Osteoporosis, Postmenopausal | 8.24 |
| BMP8A | Osteoporosis, Postmenopausal | 8.24 |
| BORCS7 | Osteoporosis, Postmenopausal | 8.24 |
| CARNMT1 | Osteoporosis, Postmenopausal | 8.24 |
| CFAP298 | Osteoporosis, Postmenopausal | 8.24 |
| HAUS3 | Osteoporosis, Postmenopausal | 8.24 |
| MAB21L4 | Osteoporosis, Postmenopausal | 8.24 |
| NAT14 | Osteoporosis, Postmenopausal | 8.24 |
| NLRP6 | Osteoporosis, Postmenopausal | 8.24 |
| PABIR1 | Osteoporosis, Postmenopausal | 8.24 |
| SCGB3A1 | Osteoporosis, Postmenopausal | 8.24 |
| TMEM222 | Osteoporosis, Postmenopausal | 8.24 |
| ADAMTS16 | Osteoporosis, Postmenopausal | 8.23 |
| AKR1C3 | Osteoporosis, Postmenopausal | 8.23 |
| B3GNT8 | Osteoporosis, Postmenopausal | 8.23 |
| CFAP57 | Osteoporosis, Postmenopausal | 8.23 |
| CIAO2B | Osteoporosis, Postmenopausal | 8.23 |
| FAM167B | Osteoporosis, Postmenopausal | 8.23 |
| FAM199X | Osteoporosis, Postmenopausal | 8.23 |
| FBXO17 | Osteoporosis, Postmenopausal | 8.23 |
| FOXL1 | Osteoporosis, Postmenopausal | 8.23 |
| GRIPAP1 | Osteoporosis, Postmenopausal | 8.23 |
| ITGB1BP2 | Osteoporosis, Postmenopausal | 8.23 |
| LRRC3 | Osteoporosis, Postmenopausal | 8.23 |
| MFAP3 | Osteoporosis, Postmenopausal | 8.23 |
| MROH6 | Osteoporosis, Postmenopausal | 8.23 |
| POLR2J | Osteoporosis, Postmenopausal | 8.23 |
| PROSER3 | Osteoporosis, Postmenopausal | 8.23 |
| SLC10A3 | Osteoporosis, Postmenopausal | 8.23 |
| SLC49A3 | Osteoporosis, Postmenopausal | 8.23 |
| TPSAB1 | Osteoporosis, Postmenopausal | 8.23 |
| UMOD | Osteoporosis, Postmenopausal | 8.23 |
| USH1G | Osteoporosis, Postmenopausal | 8.23 |
| GRPR | Osteoporosis, Postmenopausal | 8.22 |
| GSTA3 | Osteoporosis, Postmenopausal | 8.22 |
| TMEM252 | Osteoporosis, Postmenopausal | 8.22 |
| H2-DMA | Osteoporosis, Postmenopausal | 8.21 |
| APOBEC3G | Osteoporosis, Postmenopausal | 8.2 |
| B3GLCT | Osteoporosis, Postmenopausal | 8.2 |
| BRK1 | Osteoporosis, Postmenopausal | 8.2 |
| CCR9 | Osteoporosis, Postmenopausal | 8.2 |
| CHST13 | Osteoporosis, Postmenopausal | 8.2 |
| EPB42 | Osteoporosis, Postmenopausal | 8.2 |
| GPR34 | Osteoporosis, Postmenopausal | 8.2 |
| GPR37L1 | Osteoporosis, Postmenopausal | 8.2 |
| LEMD1 | Osteoporosis, Postmenopausal | 8.2 |
| MAP3K21 | Osteoporosis, Postmenopausal | 8.2 |
| MYL10 | Osteoporosis, Postmenopausal | 8.2 |
| MZT1 | Osteoporosis, Postmenopausal | 8.2 |
| PAX9 | Osteoporosis, Postmenopausal | 8.2 |
| PLAC1 | Osteoporosis, Postmenopausal | 8.2 |
| POC1B | Osteoporosis, Postmenopausal | 8.2 |
| PRXL2C | Osteoporosis, Postmenopausal | 8.2 |
| RBMXL1 | Osteoporosis, Postmenopausal | 8.2 |
| UPK3B | Osteoporosis, Postmenopausal | 8.2 |
| AICDA | Osteoporosis, Postmenopausal | 8.19 |
| ARMT1 | Osteoporosis, Postmenopausal | 8.19 |
| BTN2A2 | Osteoporosis, Postmenopausal | 8.19 |
| CYP1C1 | Osteoporosis, Postmenopausal | 8.19 |
| DNAL1 | Osteoporosis, Postmenopausal | 8.19 |
| DYNC2I1 | Osteoporosis, Postmenopausal | 8.19 |
| GABRR1 | Osteoporosis, Postmenopausal | 8.19 |
| H2BC15 | Osteoporosis, Postmenopausal | 8.19 |
| IGIP | Osteoporosis, Postmenopausal | 8.19 |
| KATNIP | Osteoporosis, Postmenopausal | 8.19 |
| LRRC4 | Osteoporosis, Postmenopausal | 8.19 |
| LRTM1 | Osteoporosis, Postmenopausal | 8.19 |
| OPTC | Osteoporosis, Postmenopausal | 8.19 |
| PODNL1 | Osteoporosis, Postmenopausal | 8.19 |
| SAMD10 | Osteoporosis, Postmenopausal | 8.19 |
| SCML1 | Osteoporosis, Postmenopausal | 8.19 |
| SMIM24 | Osteoporosis, Postmenopausal | 8.19 |
| SULT1C1 | Osteoporosis, Postmenopausal | 8.18 |
| GPR87 | Osteoporosis, Postmenopausal | 8.17 |
| HROB | Osteoporosis, Postmenopausal | 8.17 |
| IFITM10 | Osteoporosis, Postmenopausal | 8.17 |
| IQUB | Osteoporosis, Postmenopausal | 8.17 |
| KRT86 | Osteoporosis, Postmenopausal | 8.17 |
| MUC20 | Osteoporosis, Postmenopausal | 8.17 |
| NKX6-2 | Osteoporosis, Postmenopausal | 8.17 |
| PLXNB3 | Osteoporosis, Postmenopausal | 8.17 |
| POMK | Osteoporosis, Postmenopausal | 8.17 |
| RASSF10 | Osteoporosis, Postmenopausal | 8.17 |
| RLN1 | Osteoporosis, Postmenopausal | 8.17 |
| SIGLEC10 | Osteoporosis, Postmenopausal | 8.17 |
| TIMD4 | Osteoporosis, Postmenopausal | 8.17 |
| TRPC5 | Osteoporosis, Postmenopausal | 8.17 |
| BRMS1L | Osteoporosis, Postmenopausal | 8.16 |
| CCDC14 | Osteoporosis, Postmenopausal | 8.16 |
| CYP3A25 | Osteoporosis, Postmenopausal | 8.16 |
| MSL1 | Osteoporosis, Postmenopausal | 8.16 |
| PRKAG3 | Osteoporosis, Postmenopausal | 8.16 |
| RPRD1B | Osteoporosis, Postmenopausal | 8.16 |
| TRIM62 | Osteoporosis, Postmenopausal | 8.16 |
| ZNF93 | Osteoporosis, Postmenopausal | 8.16 |
| AGBL4 | Osteoporosis, Postmenopausal | 8.15 |
| C2CD4C | Osteoporosis, Postmenopausal | 8.15 |
| CNTN5 | Osteoporosis, Postmenopausal | 8.15 |
| CYP2B1 | Osteoporosis, Postmenopausal | 8.15 |
| FBXO45 | Osteoporosis, Postmenopausal | 8.15 |
| FOXI1 | Osteoporosis, Postmenopausal | 8.15 |
| GPX4 | Osteoporosis, Postmenopausal | 8.15 |
| IL20 | Osteoporosis, Postmenopausal | 8.15 |
| MED11 | Osteoporosis, Postmenopausal | 8.15 |
| MIR574 | Osteoporosis, Postmenopausal | 8.15 |
| SERPINA3C | Osteoporosis, Postmenopausal | 8.15 |
| PITRM1 | Osteoporosis, Postmenopausal | 8.14 |
| ADISSP | Osteoporosis, Postmenopausal | 8.13 |
| AHSP | Osteoporosis, Postmenopausal | 8.13 |
| CRLF3 | Osteoporosis, Postmenopausal | 8.13 |
| HSD3B | Osteoporosis, Postmenopausal | 8.13 |
| IL17RE | Osteoporosis, Postmenopausal | 8.13 |
| JCAD | Osteoporosis, Postmenopausal | 8.13 |
| KLHL25 | Osteoporosis, Postmenopausal | 8.13 |
| SLAMF8 | Osteoporosis, Postmenopausal | 8.13 |
| STX5 | Osteoporosis, Postmenopausal | 8.13 |
| SULT1C3 | Osteoporosis, Postmenopausal | 8.13 |
| URAH | Osteoporosis, Postmenopausal | 8.13 |
| XXYLT1 | Osteoporosis, Postmenopausal | 8.13 |
| ENTR1 | Osteoporosis, Postmenopausal | 8.12 |
| FTH1 | Osteoporosis, Postmenopausal | 8.12 |
| GCOM1 | Osteoporosis, Postmenopausal | 8.12 |
| KRT2 | Osteoporosis, Postmenopausal | 8.12 |
| MTIF3 | Osteoporosis, Postmenopausal | 8.12 |
| NFATC2IP | Osteoporosis, Postmenopausal | 8.12 |
| PODN | Osteoporosis, Postmenopausal | 8.12 |
| TADA1 | Osteoporosis, Postmenopausal | 8.12 |
| TGM5 | Osteoporosis, Postmenopausal | 8.12 |
| CACNG2 | Osteoporosis, Postmenopausal | 8.11 |
| CYP2C39 | Osteoporosis, Postmenopausal | 8.11 |
| DCUN1D2 | Osteoporosis, Postmenopausal | 8.11 |
| KCNA3 | Osteoporosis, Postmenopausal | 8.11 |
| MIOS | Osteoporosis, Postmenopausal | 8.11 |
| MIR532 | Osteoporosis, Postmenopausal | 8.11 |
| NAT9 | Osteoporosis, Postmenopausal | 8.11 |
| NOM1 | Osteoporosis, Postmenopausal | 8.11 |
| ODR4 | Osteoporosis, Postmenopausal | 8.11 |
| SFR1 | Osteoporosis, Postmenopausal | 8.11 |
| UBE2G1 | Osteoporosis, Postmenopausal | 8.11 |
| CALML3 | Osteoporosis, Postmenopausal | 8.1 |
| CSN1S1 | Osteoporosis, Postmenopausal | 8.1 |
| CYP2C7 | Osteoporosis, Postmenopausal | 8.1 |
| DBNDD1 | Osteoporosis, Postmenopausal | 8.1 |
| DCLRE1A | Osteoporosis, Postmenopausal | 8.1 |
| HSPA8 | Osteoporosis, Postmenopausal | 8.1 |
| SAMD13 | Osteoporosis, Postmenopausal | 8.1 |
| SLC39A2 | Osteoporosis, Postmenopausal | 8.1 |
| SRRM3 | Osteoporosis, Postmenopausal | 8.1 |
| USP20 | Osteoporosis, Postmenopausal | 8.1 |
| ATP8A2 | Osteoporosis, Postmenopausal | 8.09 |
| CRACDL | Osteoporosis, Postmenopausal | 8.09 |
| FIRRM | Osteoporosis, Postmenopausal | 8.09 |
| GIP | Osteoporosis, Postmenopausal | 8.09 |
| IGSF5 | Osteoporosis, Postmenopausal | 8.09 |
| KHDRBS2 | Osteoporosis, Postmenopausal | 8.09 |
| LY6C | Osteoporosis, Postmenopausal | 8.09 |
| MS4A6C | Osteoporosis, Postmenopausal | 8.09 |
| MZB1 | Osteoporosis, Postmenopausal | 8.09 |
| SDR39U1 | Osteoporosis, Postmenopausal | 8.09 |
| STX18 | Osteoporosis, Postmenopausal | 8.09 |
| TGM3 | Osteoporosis, Postmenopausal | 8.09 |
| TLL2 | Osteoporosis, Postmenopausal | 8.09 |
| TMPRSS13 | Osteoporosis, Postmenopausal | 8.09 |
| CYB561D1 | Osteoporosis, Postmenopausal | 8.08 |
| CYSLTR2 | Osteoporosis, Postmenopausal | 8.08 |
| DBR1 | Osteoporosis, Postmenopausal | 8.08 |
| MIR212 | Osteoporosis, Postmenopausal | 8.08 |
| MIR25 | Osteoporosis, Postmenopausal | 8.08 |
| PIGZ | Osteoporosis, Postmenopausal | 8.08 |
| TMEM126A | Osteoporosis, Postmenopausal | 8.08 |
| TRMT11 | Osteoporosis, Postmenopausal | 8.08 |
| TYW3 | Osteoporosis, Postmenopausal | 8.08 |
| UPK3A | Osteoporosis, Postmenopausal | 8.08 |
| WFDC18 | Osteoporosis, Postmenopausal | 8.08 |
| C1QL1 | Osteoporosis, Postmenopausal | 8.07 |
| CCDC149 | Osteoporosis, Postmenopausal | 8.07 |
| CNOT6 | Osteoporosis, Postmenopausal | 8.07 |
| EME2 | Osteoporosis, Postmenopausal | 8.07 |
| KLHDC1 | Osteoporosis, Postmenopausal | 8.07 |
| KLRG2 | Osteoporosis, Postmenopausal | 8.07 |
| MIR100 | Osteoporosis, Postmenopausal | 8.07 |
| PLA2G2E | Osteoporosis, Postmenopausal | 8.07 |
| RAET1E | Osteoporosis, Postmenopausal | 8.07 |
| RNF208 | Osteoporosis, Postmenopausal | 8.07 |
| THEM5 | Osteoporosis, Postmenopausal | 8.07 |
| VGLL1 | Osteoporosis, Postmenopausal | 8.07 |
| ZNF467 | Osteoporosis, Postmenopausal | 8.07 |
| ATXN7L2 | Osteoporosis, Postmenopausal | 8.06 |
| CASTOR1 | Osteoporosis, Postmenopausal | 8.06 |
| CCDC121 | Osteoporosis, Postmenopausal | 8.06 |
| CD180 | Osteoporosis, Postmenopausal | 8.06 |
| CNNM4 | Osteoporosis, Postmenopausal | 8.06 |
| CRTAM | Osteoporosis, Postmenopausal | 8.06 |
| FBXO8 | Osteoporosis, Postmenopausal | 8.06 |
| FMNL1 | Osteoporosis, Postmenopausal | 8.06 |
| INTS6L | Osteoporosis, Postmenopausal | 8.06 |
| STYK1 | Osteoporosis, Postmenopausal | 8.06 |
| TNK1 | Osteoporosis, Postmenopausal | 8.06 |
| CCSER1 | Osteoporosis, Postmenopausal | 8.05 |
| CD96 | Osteoporosis, Postmenopausal | 8.05 |
| CELA2A | Osteoporosis, Postmenopausal | 8.05 |
| CRACD | Osteoporosis, Postmenopausal | 8.05 |
| EDAR | Osteoporosis, Postmenopausal | 8.05 |
| EFHC2 | Osteoporosis, Postmenopausal | 8.05 |
| EPB41L4A-AS1 | Osteoporosis, Postmenopausal | 8.05 |
| FBXL2 | Osteoporosis, Postmenopausal | 8.05 |
| FCER1A | Osteoporosis, Postmenopausal | 8.05 |
| GORAB | Osteoporosis, Postmenopausal | 8.05 |
| H4C1 | Osteoporosis, Postmenopausal | 8.05 |
| HAMP2 | Osteoporosis, Postmenopausal | 8.05 |
| KBTBD6 | Osteoporosis, Postmenopausal | 8.05 |
| TDP2 | Osteoporosis, Postmenopausal | 8.05 |
| TRAM1L1 | Osteoporosis, Postmenopausal | 8.05 |
| RBAK | Osteoporosis, Postmenopausal | 8.04 |
| ADAT1 | Osteoporosis, Postmenopausal | 8.03 |
| ADGRF4 | Osteoporosis, Postmenopausal | 8.03 |
| BTNL9 | Osteoporosis, Postmenopausal | 8.03 |
| CLEC2B | Osteoporosis, Postmenopausal | 8.03 |
| DDX55 | Osteoporosis, Postmenopausal | 8.03 |
| DRC3 | Osteoporosis, Postmenopausal | 8.03 |
| GRHL2 | Osteoporosis, Postmenopausal | 8.03 |
| IGFLR1 | Osteoporosis, Postmenopausal | 8.03 |
| LRRC26 | Osteoporosis, Postmenopausal | 8.03 |
| LRRN4CL | Osteoporosis, Postmenopausal | 8.03 |
| NCCRP1 | Osteoporosis, Postmenopausal | 8.03 |
| NOL9 | Osteoporosis, Postmenopausal | 8.03 |
| TNFRSF18 | Osteoporosis, Postmenopausal | 8.03 |
| UBXN1 | Osteoporosis, Postmenopausal | 8.03 |
| ZNF83 | Osteoporosis, Postmenopausal | 8.03 |
| ATP9B | Osteoporosis, Postmenopausal | 8.02 |
| BEAN1 | Osteoporosis, Postmenopausal | 8.02 |
| BTLA | Osteoporosis, Postmenopausal | 8.02 |
| CACNA2D4 | Osteoporosis, Postmenopausal | 8.02 |
| CFAP70 | Osteoporosis, Postmenopausal | 8.02 |
| CTRC | Osteoporosis, Postmenopausal | 8.02 |
| DSCAM | Osteoporosis, Postmenopausal | 8.02 |
| GSPT2 | Osteoporosis, Postmenopausal | 8.02 |
| KCNG2 | Osteoporosis, Postmenopausal | 8.02 |
| NXNL2 | Osteoporosis, Postmenopausal | 8.02 |
| POT1 | Osteoporosis, Postmenopausal | 8.02 |
| PRDM6 | Osteoporosis, Postmenopausal | 8.02 |
| RDH13 | Osteoporosis, Postmenopausal | 8.02 |
| SLC26A7 | Osteoporosis, Postmenopausal | 8.02 |
| TTC9C | Osteoporosis, Postmenopausal | 8.02 |
| AP5M1 | Osteoporosis, Postmenopausal | 8.01 |
| CFAP74 | Osteoporosis, Postmenopausal | 8.01 |
| CUEDC1 | Osteoporosis, Postmenopausal | 8.01 |
| DSPP | Osteoporosis, Postmenopausal | 8.01 |
| NUP188 | Osteoporosis, Postmenopausal | 8.01 |
| PHYHIPL | Osteoporosis, Postmenopausal | 8.01 |
| PPP2R3C | Osteoporosis, Postmenopausal | 8.01 |
| SETDB2 | Osteoporosis, Postmenopausal | 8.01 |
| SPRTN | Osteoporosis, Postmenopausal | 8.01 |
| TFPT | Osteoporosis, Postmenopausal | 8.01 |
| VSIG10L | Osteoporosis, Postmenopausal | 8.01 |
| AKR1C18 | Osteoporosis, Postmenopausal | 8 |
| CARD14 | Osteoporosis, Postmenopausal | 8 |
| CDHR2 | Osteoporosis, Postmenopausal | 8 |
| CMC2 | Osteoporosis, Postmenopausal | 8 |
| F2RL3 | Osteoporosis, Postmenopausal | 8 |
| FGF6 | Osteoporosis, Postmenopausal | 8 |
| KCNH8 | Osteoporosis, Postmenopausal | 8 |
| MIR10B | Osteoporosis, Postmenopausal | 8 |
| MYLPF | Osteoporosis, Postmenopausal | 8 |
| RCCD1 | Osteoporosis, Postmenopausal | 8 |
| TRIM72 | Osteoporosis, Postmenopausal | 8 |
| USB1 | Osteoporosis, Postmenopausal | 8 |
| ZNF22 | Osteoporosis, Postmenopausal | 8 |
| ADAMDEC1 | Osteoporosis, Postmenopausal | 7.99 |
| DNAJC17 | Osteoporosis, Postmenopausal | 7.99 |
| FAM216A | Osteoporosis, Postmenopausal | 7.99 |
| GZMM | Osteoporosis, Postmenopausal | 7.99 |
| KREMEN1 | Osteoporosis, Postmenopausal | 7.99 |
| LEPROT | Osteoporosis, Postmenopausal | 7.99 |
| LIX1 | Osteoporosis, Postmenopausal | 7.99 |
| LRRN2 | Osteoporosis, Postmenopausal | 7.99 |
| MEX3A | Osteoporosis, Postmenopausal | 7.99 |
| MYO18B | Osteoporosis, Postmenopausal | 7.99 |
| PDE6H | Osteoporosis, Postmenopausal | 7.99 |
| PHF24 | Osteoporosis, Postmenopausal | 7.99 |
| PLPPR2 | Osteoporosis, Postmenopausal | 7.99 |
| RNF139 | Osteoporosis, Postmenopausal | 7.99 |
| ST6GALNAC1 | Osteoporosis, Postmenopausal | 7.99 |
| TMEM182 | Osteoporosis, Postmenopausal | 7.99 |
| TRP53INP2 | Osteoporosis, Postmenopausal | 7.99 |
| CCNJL | Osteoporosis, Postmenopausal | 7.98 |
| KBTBD3 | Osteoporosis, Postmenopausal | 7.98 |
| MOS | Osteoporosis, Postmenopausal | 7.98 |
| NSRP1 | Osteoporosis, Postmenopausal | 7.98 |
| RBMS2 | Osteoporosis, Postmenopausal | 7.98 |
| RNF130 | Osteoporosis, Postmenopausal | 7.98 |
| SLC7A6OS | Osteoporosis, Postmenopausal | 7.98 |
| TARBP1 | Osteoporosis, Postmenopausal | 7.98 |
| TMEM131L | Osteoporosis, Postmenopausal | 7.98 |
| ABCB5 | Osteoporosis, Postmenopausal | 7.97 |
| ALKBH2 | Osteoporosis, Postmenopausal | 7.97 |
| AVPR2 | Osteoporosis, Postmenopausal | 7.97 |
| IQCB1 | Osteoporosis, Postmenopausal | 7.97 |
| L3MBTL1 | Osteoporosis, Postmenopausal | 7.97 |
| NKAP | Osteoporosis, Postmenopausal | 7.97 |
| SWT1 | Osteoporosis, Postmenopausal | 7.97 |
| ZGRF1 | Osteoporosis, Postmenopausal | 7.97 |
| BICRA | Osteoporosis, Postmenopausal | 7.96 |
| BIRC7 | Osteoporosis, Postmenopausal | 7.96 |
| CCDC138 | Osteoporosis, Postmenopausal | 7.96 |
| CCDC97 | Osteoporosis, Postmenopausal | 7.96 |
| CEP68 | Osteoporosis, Postmenopausal | 7.96 |
| DSG1 | Osteoporosis, Postmenopausal | 7.96 |
| FBXO34 | Osteoporosis, Postmenopausal | 7.96 |
| FBXO46 | Osteoporosis, Postmenopausal | 7.96 |
| FOXD2 | Osteoporosis, Postmenopausal | 7.96 |
| IZUMO4 | Osteoporosis, Postmenopausal | 7.96 |
| MED25 | Osteoporosis, Postmenopausal | 7.96 |
| MYL3 | Osteoporosis, Postmenopausal | 7.96 |
| NETO1 | Osteoporosis, Postmenopausal | 7.96 |
| SPOPL | Osteoporosis, Postmenopausal | 7.96 |
| AAGAB | Osteoporosis, Postmenopausal | 7.95 |
| ADIRF | Osteoporosis, Postmenopausal | 7.95 |
| ANKRD55 | Osteoporosis, Postmenopausal | 7.95 |
| APOBEC3C | Osteoporosis, Postmenopausal | 7.95 |
| CASTOR2 | Osteoporosis, Postmenopausal | 7.95 |
| CCNYL1 | Osteoporosis, Postmenopausal | 7.95 |
| GDAP1L1 | Osteoporosis, Postmenopausal | 7.95 |
| GGT6 | Osteoporosis, Postmenopausal | 7.95 |
| GJB4 | Osteoporosis, Postmenopausal | 7.95 |
| GLMN | Osteoporosis, Postmenopausal | 7.95 |
| GPN1 | Osteoporosis, Postmenopausal | 7.95 |
| HSBP1L1 | Osteoporosis, Postmenopausal | 7.95 |
| HYAL3 | Osteoporosis, Postmenopausal | 7.95 |
| IMMP1L | Osteoporosis, Postmenopausal | 7.95 |
| LILRA5 | Osteoporosis, Postmenopausal | 7.95 |
| NDP | Osteoporosis, Postmenopausal | 7.95 |
| PDCD7 | Osteoporosis, Postmenopausal | 7.95 |
| PIWIL2 | Osteoporosis, Postmenopausal | 7.95 |
| REM2 | Osteoporosis, Postmenopausal | 7.95 |
| UNC50 | Osteoporosis, Postmenopausal | 7.95 |
| WDR70 | Osteoporosis, Postmenopausal | 7.95 |
| CCDC93 | Osteoporosis, Postmenopausal | 7.94 |
| CYP2D2 | Osteoporosis, Postmenopausal | 7.94 |
| FBXL16 | Osteoporosis, Postmenopausal | 7.94 |
| H3C2 | Osteoporosis, Postmenopausal | 7.94 |
| ICAM4 | Osteoporosis, Postmenopausal | 7.94 |
| KLK8 | Osteoporosis, Postmenopausal | 7.94 |
| LMX1B | Osteoporosis, Postmenopausal | 7.94 |
| PELI3 | Osteoporosis, Postmenopausal | 7.94 |
| TANGO2 | Osteoporosis, Postmenopausal | 7.94 |
| ARHGAP33 | Osteoporosis, Postmenopausal | 7.93 |
| ATRNL1 | Osteoporosis, Postmenopausal | 7.93 |
| C2CD3 | Osteoporosis, Postmenopausal | 7.93 |
| CACNG8 | Osteoporosis, Postmenopausal | 7.93 |
| CCDC106 | Osteoporosis, Postmenopausal | 7.93 |
| CDR2L | Osteoporosis, Postmenopausal | 7.93 |
| DNAAF9 | Osteoporosis, Postmenopausal | 7.93 |
| DSC1 | Osteoporosis, Postmenopausal | 7.93 |
| IFT22 | Osteoporosis, Postmenopausal | 7.93 |
| IL36RN | Osteoporosis, Postmenopausal | 7.93 |
| ILDR1 | Osteoporosis, Postmenopausal | 7.93 |
| MOSPD2 | Osteoporosis, Postmenopausal | 7.93 |
| NAA16 | Osteoporosis, Postmenopausal | 7.93 |
| NUP62CL | Osteoporosis, Postmenopausal | 7.93 |
| ORC5 | Osteoporosis, Postmenopausal | 7.93 |
| RBM10 | Osteoporosis, Postmenopausal | 7.93 |
| TAF1B | Osteoporosis, Postmenopausal | 7.93 |
| TLX2 | Osteoporosis, Postmenopausal | 7.93 |
| TMPRSS6 | Osteoporosis, Postmenopausal | 7.93 |
| UBE3D | Osteoporosis, Postmenopausal | 7.93 |
| USP46 | Osteoporosis, Postmenopausal | 7.93 |
| WDSUB1 | Osteoporosis, Postmenopausal | 7.93 |
| ZBTB34 | Osteoporosis, Postmenopausal | 7.93 |
| ANKRD16 | Osteoporosis, Postmenopausal | 7.92 |
| ARL5C | Osteoporosis, Postmenopausal | 7.92 |
| ARMH4 | Osteoporosis, Postmenopausal | 7.92 |
| ATP5MC2 | Osteoporosis, Postmenopausal | 7.92 |
| EBAG9 | Osteoporosis, Postmenopausal | 7.92 |
| EDC4 | Osteoporosis, Postmenopausal | 7.92 |
| FAM171A2 | Osteoporosis, Postmenopausal | 7.92 |
| FBXO41 | Osteoporosis, Postmenopausal | 7.92 |
| HEATR6 | Osteoporosis, Postmenopausal | 7.92 |
| LCMT2 | Osteoporosis, Postmenopausal | 7.92 |
| PRRT2 | Osteoporosis, Postmenopausal | 7.92 |
| REG3B | Osteoporosis, Postmenopausal | 7.92 |
| RNF169 | Osteoporosis, Postmenopausal | 7.92 |
| SLC27A6 | Osteoporosis, Postmenopausal | 7.92 |
| SPATA2L | Osteoporosis, Postmenopausal | 7.92 |
| USP33 | Osteoporosis, Postmenopausal | 7.92 |
| BLTP1 | Osteoporosis, Postmenopausal | 7.91 |
| BRINP2 | Osteoporosis, Postmenopausal | 7.91 |
| DNAJB14 | Osteoporosis, Postmenopausal | 7.91 |
| FAN1 | Osteoporosis, Postmenopausal | 7.91 |
| MRNIP | Osteoporosis, Postmenopausal | 7.91 |
| NOL12 | Osteoporosis, Postmenopausal | 7.91 |
| RBIS | Osteoporosis, Postmenopausal | 7.91 |
| RT1-BB | Osteoporosis, Postmenopausal | 7.91 |
| TP53RK | Osteoporosis, Postmenopausal | 7.91 |
| TP53TG1 | Osteoporosis, Postmenopausal | 7.91 |
| UBE3B | Osteoporosis, Postmenopausal | 7.91 |
| WIZ | Osteoporosis, Postmenopausal | 7.91 |
| AUNIP | Osteoporosis, Postmenopausal | 7.9 |
| CNKSR1 | Osteoporosis, Postmenopausal | 7.9 |
| PLEKHG6 | Osteoporosis, Postmenopausal | 7.9 |
| PRPF38A | Osteoporosis, Postmenopausal | 7.9 |
| STAC | Osteoporosis, Postmenopausal | 7.9 |
| VSTM2L | Osteoporosis, Postmenopausal | 7.9 |
| ZNF185 | Osteoporosis, Postmenopausal | 7.9 |
| ARMC10 | Osteoporosis, Postmenopausal | 7.89 |
| BAALC | Osteoporosis, Postmenopausal | 7.89 |
| CHTOP | Osteoporosis, Postmenopausal | 7.89 |
| CMTR1 | Osteoporosis, Postmenopausal | 7.89 |
| CPSF7 | Osteoporosis, Postmenopausal | 7.89 |
| CXCR6 | Osteoporosis, Postmenopausal | 7.89 |
| FAM184B | Osteoporosis, Postmenopausal | 7.89 |
| FAM221A | Osteoporosis, Postmenopausal | 7.89 |
| FAM83G | Osteoporosis, Postmenopausal | 7.89 |
| FCNA | Osteoporosis, Postmenopausal | 7.89 |
| FKBPL | Osteoporosis, Postmenopausal | 7.89 |
| GALR1 | Osteoporosis, Postmenopausal | 7.89 |
| GDPD2 | Osteoporosis, Postmenopausal | 7.89 |
| GUCD1 | Osteoporosis, Postmenopausal | 7.89 |
| IFT43 | Osteoporosis, Postmenopausal | 7.89 |
| KEL | Osteoporosis, Postmenopausal | 7.89 |
| KIAA1549L | Osteoporosis, Postmenopausal | 7.89 |
| MAP7D3 | Osteoporosis, Postmenopausal | 7.89 |
| MIR99B | Osteoporosis, Postmenopausal | 7.89 |
| MRGPRF | Osteoporosis, Postmenopausal | 7.89 |
| MS4A6D | Osteoporosis, Postmenopausal | 7.89 |
| MUP5 | Osteoporosis, Postmenopausal | 7.89 |
| MYEOV | Osteoporosis, Postmenopausal | 7.89 |
| NVL | Osteoporosis, Postmenopausal | 7.89 |
| NXPH1 | Osteoporosis, Postmenopausal | 7.89 |
| OBP2A | Osteoporosis, Postmenopausal | 7.89 |
| POC5 | Osteoporosis, Postmenopausal | 7.89 |
| REG4 | Osteoporosis, Postmenopausal | 7.89 |
| RRAGA | Osteoporosis, Postmenopausal | 7.89 |
| TASOR2 | Osteoporosis, Postmenopausal | 7.89 |
| TM2D2 | Osteoporosis, Postmenopausal | 7.89 |
| TUBGCP2 | Osteoporosis, Postmenopausal | 7.89 |
| USF1 | Osteoporosis, Postmenopausal | 7.89 |
| ZIC5 | Osteoporosis, Postmenopausal | 7.89 |
| ZMYND11 | Osteoporosis, Postmenopausal | 7.89 |
| BBS5 | Osteoporosis, Postmenopausal | 7.88 |
| CEP63 | Osteoporosis, Postmenopausal | 7.88 |
| CEP89 | Osteoporosis, Postmenopausal | 7.88 |
| CYBRD1 | Osteoporosis, Postmenopausal | 7.88 |
| DHRS13 | Osteoporosis, Postmenopausal | 7.88 |
| DOP1A | Osteoporosis, Postmenopausal | 7.88 |
| EXO5 | Osteoporosis, Postmenopausal | 7.88 |
| GUF1 | Osteoporosis, Postmenopausal | 7.88 |
| IFIT5 | Osteoporosis, Postmenopausal | 7.88 |
| INTS13 | Osteoporosis, Postmenopausal | 7.88 |
| INTS8 | Osteoporosis, Postmenopausal | 7.88 |
| MIR192 | Osteoporosis, Postmenopausal | 7.88 |
| MOB1B | Osteoporosis, Postmenopausal | 7.88 |
| PDIK1L | Osteoporosis, Postmenopausal | 7.88 |
| PGC | Osteoporosis, Postmenopausal | 7.88 |
| PLD4 | Osteoporosis, Postmenopausal | 7.88 |
| PPIP5K2 | Osteoporosis, Postmenopausal | 7.88 |
| RHOT1 | Osteoporosis, Postmenopausal | 7.88 |
| SYNE4 | Osteoporosis, Postmenopausal | 7.88 |
| TMCO3 | Osteoporosis, Postmenopausal | 7.88 |
| TMEM138 | Osteoporosis, Postmenopausal | 7.88 |
| TMEM168 | Osteoporosis, Postmenopausal | 7.88 |
| TMEM60 | Osteoporosis, Postmenopausal | 7.88 |
| VPS39 | Osteoporosis, Postmenopausal | 7.88 |
| WDR41 | Osteoporosis, Postmenopausal | 7.88 |
| ZSCAN31 | Osteoporosis, Postmenopausal | 7.88 |
| BCL7C | Osteoporosis, Postmenopausal | 7.87 |
| C1QTNF6 | Osteoporosis, Postmenopausal | 7.87 |
| MACO1 | Osteoporosis, Postmenopausal | 7.87 |
| RUBCNL | Osteoporosis, Postmenopausal | 7.87 |
| SCARF2 | Osteoporosis, Postmenopausal | 7.87 |
| VSTM4 | Osteoporosis, Postmenopausal | 7.87 |
| ATF7 | Osteoporosis, Postmenopausal | 7.86 |
| BBS1 | Osteoporosis, Postmenopausal | 7.86 |
| BMP15 | Osteoporosis, Postmenopausal | 7.86 |
| CBR2 | Osteoporosis, Postmenopausal | 7.86 |
| COL21A1 | Osteoporosis, Postmenopausal | 7.86 |
| DGUOK | Osteoporosis, Postmenopausal | 7.86 |
| DNAAF3 | Osteoporosis, Postmenopausal | 7.86 |
| DZIP1L | Osteoporosis, Postmenopausal | 7.86 |
| EDRF1 | Osteoporosis, Postmenopausal | 7.86 |
| GLRB | Osteoporosis, Postmenopausal | 7.86 |
| HOXA4 | Osteoporosis, Postmenopausal | 7.86 |
| KCTD20 | Osteoporosis, Postmenopausal | 7.86 |
| KRT75 | Osteoporosis, Postmenopausal | 7.86 |
| MIR106B | Osteoporosis, Postmenopausal | 7.86 |
| MIR34B | Osteoporosis, Postmenopausal | 7.86 |
| NAALADL2 | Osteoporosis, Postmenopausal | 7.86 |
| POLR3F | Osteoporosis, Postmenopausal | 7.86 |
| RTN4RL2 | Osteoporosis, Postmenopausal | 7.86 |
| SELENOK | Osteoporosis, Postmenopausal | 7.86 |
| SUSD5 | Osteoporosis, Postmenopausal | 7.86 |
| TAS1R1 | Osteoporosis, Postmenopausal | 7.86 |
| VMO1 | Osteoporosis, Postmenopausal | 7.86 |
| WDR82 | Osteoporosis, Postmenopausal | 7.86 |
| DNAJC22 | Osteoporosis, Postmenopausal | 7.85 |
| ETL4 | Osteoporosis, Postmenopausal | 7.85 |
| FBXL6 | Osteoporosis, Postmenopausal | 7.85 |
| GPR135 | Osteoporosis, Postmenopausal | 7.85 |
| ICAM3 | Osteoporosis, Postmenopausal | 7.85 |
| ISG20L2 | Osteoporosis, Postmenopausal | 7.85 |
| LRRC51 | Osteoporosis, Postmenopausal | 7.85 |
| MED24 | Osteoporosis, Postmenopausal | 7.85 |
| MED26 | Osteoporosis, Postmenopausal | 7.85 |
| OLFM2 | Osteoporosis, Postmenopausal | 7.85 |
| PBLD1 | Osteoporosis, Postmenopausal | 7.85 |
| PIP4K2C | Osteoporosis, Postmenopausal | 7.85 |
| SLC30A6 | Osteoporosis, Postmenopausal | 7.85 |
| SLC44A5 | Osteoporosis, Postmenopausal | 7.85 |
| TMEM170B | Osteoporosis, Postmenopausal | 7.85 |
| TRIM27 | Osteoporosis, Postmenopausal | 7.85 |
| ADAM28 | Osteoporosis, Postmenopausal | 7.84 |
| BBS10 | Osteoporosis, Postmenopausal | 7.84 |
| CYB5R4 | Osteoporosis, Postmenopausal | 7.84 |
| DDX50 | Osteoporosis, Postmenopausal | 7.84 |
| EVI2B | Osteoporosis, Postmenopausal | 7.84 |
| ORAI3 | Osteoporosis, Postmenopausal | 7.84 |
| SMG7 | Osteoporosis, Postmenopausal | 7.84 |
| ABCD4 | Osteoporosis, Postmenopausal | 7.83 |
| ABHD8 | Osteoporosis, Postmenopausal | 7.83 |
| BICRAL | Osteoporosis, Postmenopausal | 7.83 |
| CFAP44 | Osteoporosis, Postmenopausal | 7.83 |
| CHRNG | Osteoporosis, Postmenopausal | 7.83 |
| CNFN | Osteoporosis, Postmenopausal | 7.83 |
| DNAJC16 | Osteoporosis, Postmenopausal | 7.83 |
| DUSP18 | Osteoporosis, Postmenopausal | 7.83 |
| GDF2 | Osteoporosis, Postmenopausal | 7.83 |
| IFNAR1 | Osteoporosis, Postmenopausal | 7.83 |
| ISY1 | Osteoporosis, Postmenopausal | 7.83 |
| MARCHF4 | Osteoporosis, Postmenopausal | 7.83 |
| MCRIP1 | Osteoporosis, Postmenopausal | 7.83 |
| METTL27 | Osteoporosis, Postmenopausal | 7.83 |
| NYNRIN | Osteoporosis, Postmenopausal | 7.83 |
| OVAL | Osteoporosis, Postmenopausal | 7.83 |
| RANBP3 | Osteoporosis, Postmenopausal | 7.83 |
| SEC22C | Osteoporosis, Postmenopausal | 7.83 |
| SF3B6 | Osteoporosis, Postmenopausal | 7.83 |
| SS18 | Osteoporosis, Postmenopausal | 7.83 |
| TMEM132D | Osteoporosis, Postmenopausal | 7.83 |
| TMEM199 | Osteoporosis, Postmenopausal | 7.83 |
| UBE2QL1 | Osteoporosis, Postmenopausal | 7.83 |
| ADARB2 | Osteoporosis, Postmenopausal | 7.82 |
| ANKEF1 | Osteoporosis, Postmenopausal | 7.82 |
| CYP2C12 | Osteoporosis, Postmenopausal | 7.82 |
| GCFC2 | Osteoporosis, Postmenopausal | 7.82 |
| GYG2 | Osteoporosis, Postmenopausal | 7.82 |
| HES4 | Osteoporosis, Postmenopausal | 7.82 |
| HEXD | Osteoporosis, Postmenopausal | 7.82 |
| HOXD8 | Osteoporosis, Postmenopausal | 7.82 |
| ITGA10 | Osteoporosis, Postmenopausal | 7.82 |
| KATNAL2 | Osteoporosis, Postmenopausal | 7.82 |
| LTB4R | Osteoporosis, Postmenopausal | 7.82 |
| LYPD6B | Osteoporosis, Postmenopausal | 7.82 |
| LZTR1 | Osteoporosis, Postmenopausal | 7.82 |
| MIR214 | Osteoporosis, Postmenopausal | 7.82 |
| MKX | Osteoporosis, Postmenopausal | 7.82 |
| MRPL16 | Osteoporosis, Postmenopausal | 7.82 |
| NEK9 | Osteoporosis, Postmenopausal | 7.82 |
| OXNAD1 | Osteoporosis, Postmenopausal | 7.82 |
| PHACTR3 | Osteoporosis, Postmenopausal | 7.82 |
| RNPC3 | Osteoporosis, Postmenopausal | 7.82 |
| SEC14L3 | Osteoporosis, Postmenopausal | 7.82 |
| SENP3 | Osteoporosis, Postmenopausal | 7.82 |
| TREM1 | Osteoporosis, Postmenopausal | 7.82 |
| TTC33 | Osteoporosis, Postmenopausal | 7.82 |
| BRI3 | Osteoporosis, Postmenopausal | 7.81 |
| CYP4X1 | Osteoporosis, Postmenopausal | 7.81 |
| IL18RAP | Osteoporosis, Postmenopausal | 7.81 |
| KRT6B | Osteoporosis, Postmenopausal | 7.81 |
| PARVG | Osteoporosis, Postmenopausal | 7.81 |
| PLPP1 | Osteoporosis, Postmenopausal | 7.81 |
| TMEM79 | Osteoporosis, Postmenopausal | 7.81 |
| COQ2 | Osteoporosis, Postmenopausal | 7.8 |
| FUT11 | Osteoporosis, Postmenopausal | 7.8 |
| GOLGA5 | Osteoporosis, Postmenopausal | 7.8 |
| LYRM7 | Osteoporosis, Postmenopausal | 7.8 |
| PITX3 | Osteoporosis, Postmenopausal | 7.8 |
| PSMD10 | Osteoporosis, Postmenopausal | 7.8 |
| SCMH1 | Osteoporosis, Postmenopausal | 7.8 |
| SLC6A18 | Osteoporosis, Postmenopausal | 7.8 |
| USP37 | Osteoporosis, Postmenopausal | 7.8 |
| ASIC3 | Osteoporosis, Postmenopausal | 7.79 |
| ATP6V1B1 | Osteoporosis, Postmenopausal | 7.79 |
| CCHCR1 | Osteoporosis, Postmenopausal | 7.79 |
| CLCNKB | Osteoporosis, Postmenopausal | 7.79 |
| CRHBP | Osteoporosis, Postmenopausal | 7.79 |
| DACT3 | Osteoporosis, Postmenopausal | 7.79 |
| DCDC2 | Osteoporosis, Postmenopausal | 7.79 |
| DCLRE1B | Osteoporosis, Postmenopausal | 7.79 |
| ENKUR | Osteoporosis, Postmenopausal | 7.79 |
| EVPL | Osteoporosis, Postmenopausal | 7.79 |
| EXOC5 | Osteoporosis, Postmenopausal | 7.79 |
| FOXS1 | Osteoporosis, Postmenopausal | 7.79 |
| FUT2 | Osteoporosis, Postmenopausal | 7.79 |
| GPR27 | Osteoporosis, Postmenopausal | 7.79 |
| HOXC6 | Osteoporosis, Postmenopausal | 7.79 |
| HSF2BP | Osteoporosis, Postmenopausal | 7.79 |
| KIF26A | Osteoporosis, Postmenopausal | 7.79 |
| LGI2 | Osteoporosis, Postmenopausal | 7.79 |
| MRPL27 | Osteoporosis, Postmenopausal | 7.79 |
| NTAN1 | Osteoporosis, Postmenopausal | 7.79 |
| OSCAR | Osteoporosis, Postmenopausal | 7.79 |
| RAB6A | Osteoporosis, Postmenopausal | 7.79 |
| RNF34 | Osteoporosis, Postmenopausal | 7.79 |
| SLC9A6 | Osteoporosis, Postmenopausal | 7.79 |
| SMG9 | Osteoporosis, Postmenopausal | 7.79 |
| SOCS4 | Osteoporosis, Postmenopausal | 7.79 |
| TAF5L | Osteoporosis, Postmenopausal | 7.79 |
| TBC1D7 | Osteoporosis, Postmenopausal | 7.79 |
| TEP1 | Osteoporosis, Postmenopausal | 7.79 |
| TEX9 | Osteoporosis, Postmenopausal | 7.79 |
| TNFRSF10C | Osteoporosis, Postmenopausal | 7.79 |
| TRIM45 | Osteoporosis, Postmenopausal | 7.79 |
| TUBD1 | Osteoporosis, Postmenopausal | 7.79 |
| UFL1 | Osteoporosis, Postmenopausal | 7.79 |
| UQCC1 | Osteoporosis, Postmenopausal | 7.79 |
| USP8 | Osteoporosis, Postmenopausal | 7.79 |
| WRNIP1 | Osteoporosis, Postmenopausal | 7.79 |
| ALPK3 | Osteoporosis, Postmenopausal | 7.78 |
| CNGB1 | Osteoporosis, Postmenopausal | 7.78 |
| LONRF1 | Osteoporosis, Postmenopausal | 7.78 |
| LORICRIN | Osteoporosis, Postmenopausal | 7.78 |
| MMP28 | Osteoporosis, Postmenopausal | 7.78 |
| N4BP3 | Osteoporosis, Postmenopausal | 7.78 |
| NUDT11 | Osteoporosis, Postmenopausal | 7.78 |
| SHISA4 | Osteoporosis, Postmenopausal | 7.78 |
| SPTSSB | Osteoporosis, Postmenopausal | 7.78 |
| ALG13 | Osteoporosis, Postmenopausal | 7.77 |
| APOF | Osteoporosis, Postmenopausal | 7.77 |
| ASB7 | Osteoporosis, Postmenopausal | 7.77 |
| B3GNT3 | Osteoporosis, Postmenopausal | 7.77 |
| CABP1 | Osteoporosis, Postmenopausal | 7.77 |
| CHCHD1 | Osteoporosis, Postmenopausal | 7.77 |
| LMX1A | Osteoporosis, Postmenopausal | 7.77 |
| PTOV1 | Osteoporosis, Postmenopausal | 7.77 |
| SERPINA11 | Osteoporosis, Postmenopausal | 7.77 |
| SLC25A46 | Osteoporosis, Postmenopausal | 7.77 |
| SYAP1 | Osteoporosis, Postmenopausal | 7.77 |
| BIVM | Osteoporosis, Postmenopausal | 7.76 |
| CD226 | Osteoporosis, Postmenopausal | 7.76 |
| CHST6 | Osteoporosis, Postmenopausal | 7.76 |
| CNOT8 | Osteoporosis, Postmenopausal | 7.76 |
| CTSW | Osteoporosis, Postmenopausal | 7.76 |
| DEDD | Osteoporosis, Postmenopausal | 7.76 |
| DIP2A | Osteoporosis, Postmenopausal | 7.76 |
| EFL1 | Osteoporosis, Postmenopausal | 7.76 |
| ERICH1 | Osteoporosis, Postmenopausal | 7.76 |
| FAM193B | Osteoporosis, Postmenopausal | 7.76 |
| GDAP2 | Osteoporosis, Postmenopausal | 7.76 |
| H2AC11 | Osteoporosis, Postmenopausal | 7.76 |
| HCST | Osteoporosis, Postmenopausal | 7.76 |
| HECW1 | Osteoporosis, Postmenopausal | 7.76 |
| HOXA11 | Osteoporosis, Postmenopausal | 7.76 |
| LCLAT1 | Osteoporosis, Postmenopausal | 7.76 |
| LIME1 | Osteoporosis, Postmenopausal | 7.76 |
| LYRM9 | Osteoporosis, Postmenopausal | 7.76 |
| MED4 | Osteoporosis, Postmenopausal | 7.76 |
| MFSD10 | Osteoporosis, Postmenopausal | 7.76 |
| MYBPC2 | Osteoporosis, Postmenopausal | 7.76 |
| PLB1 | Osteoporosis, Postmenopausal | 7.76 |
| RAB36 | Osteoporosis, Postmenopausal | 7.76 |
| RETNLG | Osteoporosis, Postmenopausal | 7.76 |
| SENP5 | Osteoporosis, Postmenopausal | 7.76 |
| SLC38A9 | Osteoporosis, Postmenopausal | 7.76 |
| SLC39A9 | Osteoporosis, Postmenopausal | 7.76 |
| SVIP | Osteoporosis, Postmenopausal | 7.76 |
| TBX18 | Osteoporosis, Postmenopausal | 7.76 |
| TNFRSF13B | Osteoporosis, Postmenopausal | 7.76 |
| TOMM70A | Osteoporosis, Postmenopausal | 7.76 |
| VIT | Osteoporosis, Postmenopausal | 7.76 |
| VPS37D | Osteoporosis, Postmenopausal | 7.76 |
| ZBTB22 | Osteoporosis, Postmenopausal | 7.76 |
| ZMYND12 | Osteoporosis, Postmenopausal | 7.76 |
| ZNRF3 | Osteoporosis, Postmenopausal | 7.76 |
| ACBD7 | Osteoporosis, Postmenopausal | 7.75 |
| ACY3 | Osteoporosis, Postmenopausal | 7.75 |
| CCNB1IP1 | Osteoporosis, Postmenopausal | 7.75 |
| CHRD | Osteoporosis, Postmenopausal | 7.75 |
| CHST7 | Osteoporosis, Postmenopausal | 7.75 |
| DENND1C | Osteoporosis, Postmenopausal | 7.75 |
| SPOCK3 | Osteoporosis, Postmenopausal | 7.75 |
| SREK1IP1 | Osteoporosis, Postmenopausal | 7.75 |
| STAP2 | Osteoporosis, Postmenopausal | 7.75 |
| UIMC1 | Osteoporosis, Postmenopausal | 7.75 |
| XKR5 | Osteoporosis, Postmenopausal | 7.75 |
| CIC | Osteoporosis, Postmenopausal | 7.74 |
| COX19 | Osteoporosis, Postmenopausal | 7.74 |
| CRYBG3 | Osteoporosis, Postmenopausal | 7.74 |
| CYP4A12A | Osteoporosis, Postmenopausal | 7.74 |
| ERCC8 | Osteoporosis, Postmenopausal | 7.74 |
| HLA-DMB | Osteoporosis, Postmenopausal | 7.74 |
| LSM14B | Osteoporosis, Postmenopausal | 7.74 |
| MCMBP | Osteoporosis, Postmenopausal | 7.74 |
| MED27 | Osteoporosis, Postmenopausal | 7.74 |
| MGME1 | Osteoporosis, Postmenopausal | 7.74 |
| MIR29A | Osteoporosis, Postmenopausal | 7.74 |
| MRE11A | Osteoporosis, Postmenopausal | 7.74 |
| NECAP2 | Osteoporosis, Postmenopausal | 7.74 |
| NEUROG1 | Osteoporosis, Postmenopausal | 7.74 |
| NPY2R | Osteoporosis, Postmenopausal | 7.74 |
| POLDIP2 | Osteoporosis, Postmenopausal | 7.74 |
| RELL2 | Osteoporosis, Postmenopausal | 7.74 |
| RPGRIP1L | Osteoporosis, Postmenopausal | 7.74 |
| SHPRH | Osteoporosis, Postmenopausal | 7.74 |
| SLC18B1 | Osteoporosis, Postmenopausal | 7.74 |
| SV2C | Osteoporosis, Postmenopausal | 7.74 |
| TEDC2 | Osteoporosis, Postmenopausal | 7.74 |
| ABCF3 | Osteoporosis, Postmenopausal | 7.73 |
| ADAMTS18 | Osteoporosis, Postmenopausal | 7.73 |
| AK9 | Osteoporosis, Postmenopausal | 7.73 |
| CAMK2N2 | Osteoporosis, Postmenopausal | 7.73 |
| CHMP5 | Osteoporosis, Postmenopausal | 7.73 |
| DCUN1D5 | Osteoporosis, Postmenopausal | 7.73 |
| DOK6 | Osteoporosis, Postmenopausal | 7.73 |
| ESYT3 | Osteoporosis, Postmenopausal | 7.73 |
| FAM3B | Osteoporosis, Postmenopausal | 7.73 |
| FAR2 | Osteoporosis, Postmenopausal | 7.73 |
| FMC1 | Osteoporosis, Postmenopausal | 7.73 |
| GPKOW | Osteoporosis, Postmenopausal | 7.73 |
| GSC | Osteoporosis, Postmenopausal | 7.73 |
| H2AC8 | Osteoporosis, Postmenopausal | 7.73 |
| HEXIM2 | Osteoporosis, Postmenopausal | 7.73 |
| IGBP1 | Osteoporosis, Postmenopausal | 7.73 |
| IL36B | Osteoporosis, Postmenopausal | 7.73 |
| INO80B | Osteoporosis, Postmenopausal | 7.73 |
| KY | Osteoporosis, Postmenopausal | 7.73 |
| LRRC49 | Osteoporosis, Postmenopausal | 7.73 |
| MIR142 | Osteoporosis, Postmenopausal | 7.73 |
| MYG1 | Osteoporosis, Postmenopausal | 7.73 |
| MYOZ1 | Osteoporosis, Postmenopausal | 7.73 |
| NEMF | Osteoporosis, Postmenopausal | 7.73 |
| OTUD6B | Osteoporosis, Postmenopausal | 7.73 |
| PCDH19 | Osteoporosis, Postmenopausal | 7.73 |
| PDCD10 | Osteoporosis, Postmenopausal | 7.73 |
| PI15 | Osteoporosis, Postmenopausal | 7.73 |
| RNF138 | Osteoporosis, Postmenopausal | 7.73 |
| RPRD2 | Osteoporosis, Postmenopausal | 7.73 |
| SERPINA4 | Osteoporosis, Postmenopausal | 7.73 |
| SLC25A21 | Osteoporosis, Postmenopausal | 7.73 |
| SLC6A20 | Osteoporosis, Postmenopausal | 7.73 |
| SNHG7 | Osteoporosis, Postmenopausal | 7.73 |
| SPRR1B | Osteoporosis, Postmenopausal | 7.73 |
| TAF1A | Osteoporosis, Postmenopausal | 7.73 |
| TELO2 | Osteoporosis, Postmenopausal | 7.73 |
| TRAPPC4 | Osteoporosis, Postmenopausal | 7.73 |
| ZDHHC17 | Osteoporosis, Postmenopausal | 7.73 |
| ZFYVE19 | Osteoporosis, Postmenopausal | 7.73 |
| CSDC2 | Osteoporosis, Postmenopausal | 7.72 |
| FBXO4 | Osteoporosis, Postmenopausal | 7.72 |
| ISM1 | Osteoporosis, Postmenopausal | 7.72 |
| LYPD3 | Osteoporosis, Postmenopausal | 7.72 |
| MAEA | Osteoporosis, Postmenopausal | 7.72 |
| PGBD5 | Osteoporosis, Postmenopausal | 7.72 |
| PIGW | Osteoporosis, Postmenopausal | 7.72 |
| SERAC1 | Osteoporosis, Postmenopausal | 7.72 |
| STK24 | Osteoporosis, Postmenopausal | 7.72 |
| TCEAL1 | Osteoporosis, Postmenopausal | 7.72 |
| TINAG | Osteoporosis, Postmenopausal | 7.72 |
| AFG2A | Osteoporosis, Postmenopausal | 7.71 |
| CABIN1 | Osteoporosis, Postmenopausal | 7.71 |
| ELMOD2 | Osteoporosis, Postmenopausal | 7.71 |
| HOXA3 | Osteoporosis, Postmenopausal | 7.71 |
| MTREX | Osteoporosis, Postmenopausal | 7.71 |
| PPWD1 | Osteoporosis, Postmenopausal | 7.71 |
| WASHC5 | Osteoporosis, Postmenopausal | 7.71 |
| AMY1 | Osteoporosis, Postmenopausal | 7.7 |
| ANO4 | Osteoporosis, Postmenopausal | 7.7 |
| AOAH | Osteoporosis, Postmenopausal | 7.7 |
| AOC2 | Osteoporosis, Postmenopausal | 7.7 |
| COL22A1 | Osteoporosis, Postmenopausal | 7.7 |
| CWC25 | Osteoporosis, Postmenopausal | 7.7 |
| DIPK2A | Osteoporosis, Postmenopausal | 7.7 |
| FABP10A | Osteoporosis, Postmenopausal | 7.7 |
| FAM161B | Osteoporosis, Postmenopausal | 7.7 |
| FANCE | Osteoporosis, Postmenopausal | 7.7 |
| GAREM1 | Osteoporosis, Postmenopausal | 7.7 |
| GLT8D2 | Osteoporosis, Postmenopausal | 7.7 |
| H3-3B | Osteoporosis, Postmenopausal | 7.7 |
| ICOS | Osteoporosis, Postmenopausal | 7.7 |
| KISS1R | Osteoporosis, Postmenopausal | 7.7 |
| LHX4 | Osteoporosis, Postmenopausal | 7.7 |
| LRRC58 | Osteoporosis, Postmenopausal | 7.7 |
| MZF1 | Osteoporosis, Postmenopausal | 7.7 |
| NHS | Osteoporosis, Postmenopausal | 7.7 |
| NIM1K | Osteoporosis, Postmenopausal | 7.7 |
| OSGEPL1 | Osteoporosis, Postmenopausal | 7.7 |
| P2RX3 | Osteoporosis, Postmenopausal | 7.7 |
| PIGL | Osteoporosis, Postmenopausal | 7.7 |
| PNLIPRP1 | Osteoporosis, Postmenopausal | 7.7 |
| PSENEN | Osteoporosis, Postmenopausal | 7.7 |
| RSPO2 | Osteoporosis, Postmenopausal | 7.7 |
| SEC22A | Osteoporosis, Postmenopausal | 7.7 |
| SIDT1 | Osteoporosis, Postmenopausal | 7.7 |
| SLC6A17 | Osteoporosis, Postmenopausal | 7.7 |
| SNAPC4 | Osteoporosis, Postmenopausal | 7.7 |
| SPNS1 | Osteoporosis, Postmenopausal | 7.7 |
| TRIP11 | Osteoporosis, Postmenopausal | 7.7 |
| ZDHHC7 | Osteoporosis, Postmenopausal | 7.7 |
| ZFAND4 | Osteoporosis, Postmenopausal | 7.7 |
| ZNF217 | Osteoporosis, Postmenopausal | 7.7 |
| ZSCAN26 | Osteoporosis, Postmenopausal | 7.7 |
| ASB9 | Osteoporosis, Postmenopausal | 7.69 |
| IL36G | Osteoporosis, Postmenopausal | 7.69 |
| MAP3K9 | Osteoporosis, Postmenopausal | 7.69 |
| PLPPR1 | Osteoporosis, Postmenopausal | 7.69 |
| PROK2 | Osteoporosis, Postmenopausal | 7.69 |
| RAB12 | Osteoporosis, Postmenopausal | 7.69 |
| SLC17A9 | Osteoporosis, Postmenopausal | 7.69 |
| TSPYL4 | Osteoporosis, Postmenopausal | 7.69 |
| BAIAP3 | Osteoporosis, Postmenopausal | 7.68 |
| BTRC | Osteoporosis, Postmenopausal | 7.68 |
| CASQ1 | Osteoporosis, Postmenopausal | 7.68 |
| IGSF8 | Osteoporosis, Postmenopausal | 7.68 |
| KATNBL1 | Osteoporosis, Postmenopausal | 7.68 |
| KLHL41 | Osteoporosis, Postmenopausal | 7.68 |
| MIR205 | Osteoporosis, Postmenopausal | 7.68 |
| NTNG2 | Osteoporosis, Postmenopausal | 7.68 |
| NUDCD2 | Osteoporosis, Postmenopausal | 7.68 |
| PKP3 | Osteoporosis, Postmenopausal | 7.68 |
| PWP1 | Osteoporosis, Postmenopausal | 7.68 |
| RIOX1 | Osteoporosis, Postmenopausal | 7.68 |
| SLC37A1 | Osteoporosis, Postmenopausal | 7.68 |
| SNX18 | Osteoporosis, Postmenopausal | 7.68 |
| TBC1D14 | Osteoporosis, Postmenopausal | 7.68 |
| TMEM41B | Osteoporosis, Postmenopausal | 7.68 |
| AMIGO1 | Osteoporosis, Postmenopausal | 7.67 |
| AP4E1 | Osteoporosis, Postmenopausal | 7.67 |
| ARHGEF25 | Osteoporosis, Postmenopausal | 7.67 |
| BLTP3A | Osteoporosis, Postmenopausal | 7.67 |
| C5AR2 | Osteoporosis, Postmenopausal | 7.67 |
| CLCN6 | Osteoporosis, Postmenopausal | 7.67 |
| DEF8 | Osteoporosis, Postmenopausal | 7.67 |
| DSC3 | Osteoporosis, Postmenopausal | 7.67 |
| FAM161A | Osteoporosis, Postmenopausal | 7.67 |
| FAM219A | Osteoporosis, Postmenopausal | 7.67 |
| GGNBP2 | Osteoporosis, Postmenopausal | 7.67 |
| IFT46 | Osteoporosis, Postmenopausal | 7.67 |
| KIFAP3 | Osteoporosis, Postmenopausal | 7.67 |
| KLF1 | Osteoporosis, Postmenopausal | 7.67 |
| L3HYPDH | Osteoporosis, Postmenopausal | 7.67 |
| LNPK | Osteoporosis, Postmenopausal | 7.67 |
| MNDA | Osteoporosis, Postmenopausal | 7.67 |
| MSANTD3 | Osteoporosis, Postmenopausal | 7.67 |
| PLXNA3 | Osteoporosis, Postmenopausal | 7.67 |
| POU2F3 | Osteoporosis, Postmenopausal | 7.67 |
| PRR16 | Osteoporosis, Postmenopausal | 7.67 |
| PSMF1 | Osteoporosis, Postmenopausal | 7.67 |
| PYM1 | Osteoporosis, Postmenopausal | 7.67 |
| RESF1 | Osteoporosis, Postmenopausal | 7.67 |
| RHNO1 | Osteoporosis, Postmenopausal | 7.67 |
| SFRP5 | Osteoporosis, Postmenopausal | 7.67 |
| SLC4A3 | Osteoporosis, Postmenopausal | 7.67 |
| SNAI3 | Osteoporosis, Postmenopausal | 7.67 |
| SYDE1 | Osteoporosis, Postmenopausal | 7.67 |
| TATDN2 | Osteoporosis, Postmenopausal | 7.67 |
| THAP2 | Osteoporosis, Postmenopausal | 7.67 |
| TPRN | Osteoporosis, Postmenopausal | 7.67 |
| TRNT1 | Osteoporosis, Postmenopausal | 7.67 |
| ZNF277 | Osteoporosis, Postmenopausal | 7.67 |
| ADAM33 | Osteoporosis, Postmenopausal | 7.66 |
| ASH2L | Osteoporosis, Postmenopausal | 7.66 |
| C9ORF72 | Osteoporosis, Postmenopausal | 7.66 |
| FAM241B | Osteoporosis, Postmenopausal | 7.66 |
| GPIHBP1 | Osteoporosis, Postmenopausal | 7.66 |
| HEATR5A | Osteoporosis, Postmenopausal | 7.66 |
| KCNC1 | Osteoporosis, Postmenopausal | 7.66 |
| MGAM | Osteoporosis, Postmenopausal | 7.66 |
| SUGT1 | Osteoporosis, Postmenopausal | 7.66 |
| ZFAS1 | Osteoporosis, Postmenopausal | 7.66 |
| ANGPTL1 | Osteoporosis, Postmenopausal | 7.65 |
| DOHH | Osteoporosis, Postmenopausal | 7.65 |
| ENDOU | Osteoporosis, Postmenopausal | 7.65 |
| GPAA1 | Osteoporosis, Postmenopausal | 7.65 |
| GTF2H4 | Osteoporosis, Postmenopausal | 7.65 |
| LEFTY1 | Osteoporosis, Postmenopausal | 7.65 |
| LRRC34 | Osteoporosis, Postmenopausal | 7.65 |
| MEP1B | Osteoporosis, Postmenopausal | 7.65 |
| MMRN1 | Osteoporosis, Postmenopausal | 7.65 |
| NEK1 | Osteoporosis, Postmenopausal | 7.65 |
| A1CF | Osteoporosis, Postmenopausal | 7.64 |
| ADGRB2 | Osteoporosis, Postmenopausal | 7.64 |
| CHST4 | Osteoporosis, Postmenopausal | 7.64 |
| DISP2 | Osteoporosis, Postmenopausal | 7.64 |
| EPS15L1 | Osteoporosis, Postmenopausal | 7.64 |
| F13B | Osteoporosis, Postmenopausal | 7.64 |
| FBRSL1 | Osteoporosis, Postmenopausal | 7.64 |
| IGFBPL1 | Osteoporosis, Postmenopausal | 7.64 |
| IL20RA | Osteoporosis, Postmenopausal | 7.64 |
| INAVA | Osteoporosis, Postmenopausal | 7.64 |
| KCNJ1 | Osteoporosis, Postmenopausal | 7.64 |
| KCTD13 | Osteoporosis, Postmenopausal | 7.64 |
| KLK10 | Osteoporosis, Postmenopausal | 7.64 |
| MRPL48 | Osteoporosis, Postmenopausal | 7.64 |
| ND4 | Osteoporosis, Postmenopausal | 7.64 |
| NHLRC3 | Osteoporosis, Postmenopausal | 7.64 |
| POLR1F | Osteoporosis, Postmenopausal | 7.64 |
| RYK | Osteoporosis, Postmenopausal | 7.64 |
| SLC30A9 | Osteoporosis, Postmenopausal | 7.64 |
| SLC52A3 | Osteoporosis, Postmenopausal | 7.64 |
| SUDS3 | Osteoporosis, Postmenopausal | 7.64 |
| TMEM125 | Osteoporosis, Postmenopausal | 7.64 |
| ZC3H7A | Osteoporosis, Postmenopausal | 7.64 |
| ZMYM6 | Osteoporosis, Postmenopausal | 7.64 |
| ZNF148 | Osteoporosis, Postmenopausal | 7.64 |
| ZNRF2 | Osteoporosis, Postmenopausal | 7.64 |
| ANKS4B | Osteoporosis, Postmenopausal | 7.63 |
| ATP13A1 | Osteoporosis, Postmenopausal | 7.63 |
| BRD7 | Osteoporosis, Postmenopausal | 7.63 |
| CEP83 | Osteoporosis, Postmenopausal | 7.63 |
| LDAH | Osteoporosis, Postmenopausal | 7.63 |
| LSM11 | Osteoporosis, Postmenopausal | 7.63 |
| MRPS31 | Osteoporosis, Postmenopausal | 7.63 |
| PTPMT1 | Osteoporosis, Postmenopausal | 7.63 |
| RAI2 | Osteoporosis, Postmenopausal | 7.63 |
| RTCA | Osteoporosis, Postmenopausal | 7.63 |
| TIMM21 | Osteoporosis, Postmenopausal | 7.63 |
| TRMT10A | Osteoporosis, Postmenopausal | 7.63 |
| ZBTB2 | Osteoporosis, Postmenopausal | 7.63 |
| ABTB1 | Osteoporosis, Postmenopausal | 7.62 |
| ARHGAP19 | Osteoporosis, Postmenopausal | 7.62 |
| ARL5A | Osteoporosis, Postmenopausal | 7.62 |
| BTBD10 | Osteoporosis, Postmenopausal | 7.62 |
| CD300LF | Osteoporosis, Postmenopausal | 7.62 |
| DENND4C | Osteoporosis, Postmenopausal | 7.62 |
| EXOSC9 | Osteoporosis, Postmenopausal | 7.62 |
| GALNT13 | Osteoporosis, Postmenopausal | 7.62 |
| HLA-DRA | Osteoporosis, Postmenopausal | 7.62 |
| PDE6C | Osteoporosis, Postmenopausal | 7.62 |
| PLXNA4 | Osteoporosis, Postmenopausal | 7.62 |
| SNX33 | Osteoporosis, Postmenopausal | 7.62 |
| SYNC | Osteoporosis, Postmenopausal | 7.62 |
| TDH | Osteoporosis, Postmenopausal | 7.62 |
| TMPRSS4 | Osteoporosis, Postmenopausal | 7.62 |
| TNPO2 | Osteoporosis, Postmenopausal | 7.62 |
| UBIAD1 | Osteoporosis, Postmenopausal | 7.62 |
| CASKIN1 | Osteoporosis, Postmenopausal | 7.61 |
| CNIH3 | Osteoporosis, Postmenopausal | 7.61 |
| DESI1 | Osteoporosis, Postmenopausal | 7.61 |
| ECEL1 | Osteoporosis, Postmenopausal | 7.61 |
| EXOC1 | Osteoporosis, Postmenopausal | 7.61 |
| FIBCD1 | Osteoporosis, Postmenopausal | 7.61 |
| FLAD1 | Osteoporosis, Postmenopausal | 7.61 |
| FUBP3 | Osteoporosis, Postmenopausal | 7.61 |
| GEN1 | Osteoporosis, Postmenopausal | 7.61 |
| HS3ST6 | Osteoporosis, Postmenopausal | 7.61 |
| IQCK | Osteoporosis, Postmenopausal | 7.61 |
| KLHL12 | Osteoporosis, Postmenopausal | 7.61 |
| MPHOSPH8 | Osteoporosis, Postmenopausal | 7.61 |
| MYBPH | Osteoporosis, Postmenopausal | 7.61 |
| NSMCE2 | Osteoporosis, Postmenopausal | 7.61 |
| OIT3 | Osteoporosis, Postmenopausal | 7.61 |
| PLGRKT | Osteoporosis, Postmenopausal | 7.61 |
| PRAF2 | Osteoporosis, Postmenopausal | 7.61 |
| SIX5 | Osteoporosis, Postmenopausal | 7.61 |
| SLC35A1 | Osteoporosis, Postmenopausal | 7.61 |
| SMTNL2 | Osteoporosis, Postmenopausal | 7.61 |
| SPOCD1 | Osteoporosis, Postmenopausal | 7.61 |
| SYF2 | Osteoporosis, Postmenopausal | 7.61 |
| TCF15 | Osteoporosis, Postmenopausal | 7.61 |
| TEX30 | Osteoporosis, Postmenopausal | 7.61 |
| THOC6 | Osteoporosis, Postmenopausal | 7.61 |
| TRABD | Osteoporosis, Postmenopausal | 7.61 |
| ZSWIM5 | Osteoporosis, Postmenopausal | 7.61 |
| ALG6 | Osteoporosis, Postmenopausal | 7.6 |
| BMPR1A | Osteoporosis, Postmenopausal | 7.6 |
| BTF3L4 | Osteoporosis, Postmenopausal | 7.6 |
| CEP135 | Osteoporosis, Postmenopausal | 7.6 |
| DLEU2 | Osteoporosis, Postmenopausal | 7.6 |
| PDSS2 | Osteoporosis, Postmenopausal | 7.6 |
| SCN9A | Osteoporosis, Postmenopausal | 7.6 |
| TOMM22 | Osteoporosis, Postmenopausal | 7.6 |
| AGBL2 | Osteoporosis, Postmenopausal | 7.59 |
| EDC3 | Osteoporosis, Postmenopausal | 7.59 |
| EIF4EBP3 | Osteoporosis, Postmenopausal | 7.59 |
| FCRLA | Osteoporosis, Postmenopausal | 7.59 |
| FFAR4 | Osteoporosis, Postmenopausal | 7.59 |
| FRAT1 | Osteoporosis, Postmenopausal | 7.59 |
| GDF3 | Osteoporosis, Postmenopausal | 7.59 |
| GJB5 | Osteoporosis, Postmenopausal | 7.59 |
| H2BC14 | Osteoporosis, Postmenopausal | 7.59 |
| KHNYN | Osteoporosis, Postmenopausal | 7.59 |
| MAGEH1 | Osteoporosis, Postmenopausal | 7.59 |
| NKG7 | Osteoporosis, Postmenopausal | 7.59 |
| PITX1 | Osteoporosis, Postmenopausal | 7.59 |
| PRR5 | Osteoporosis, Postmenopausal | 7.59 |
| SLC25A34 | Osteoporosis, Postmenopausal | 7.59 |
| SPAG4 | Osteoporosis, Postmenopausal | 7.59 |
| TAB3 | Osteoporosis, Postmenopausal | 7.59 |
| TCTA | Osteoporosis, Postmenopausal | 7.59 |
| THNSL1 | Osteoporosis, Postmenopausal | 7.59 |
| UBE2D2 | Osteoporosis, Postmenopausal | 7.59 |
| ZBED3 | Osteoporosis, Postmenopausal | 7.59 |
| CENPS | Osteoporosis, Postmenopausal | 7.58 |
| CTR9 | Osteoporosis, Postmenopausal | 7.58 |
| DCTN4 | Osteoporosis, Postmenopausal | 7.58 |
| DHX33 | Osteoporosis, Postmenopausal | 7.58 |
| EBF2 | Osteoporosis, Postmenopausal | 7.58 |
| FREM1 | Osteoporosis, Postmenopausal | 7.58 |
| LAS1L | Osteoporosis, Postmenopausal | 7.58 |
| LRRFIP2 | Osteoporosis, Postmenopausal | 7.58 |
| LSM14A | Osteoporosis, Postmenopausal | 7.58 |
| MAK16 | Osteoporosis, Postmenopausal | 7.58 |
| MC1R | Osteoporosis, Postmenopausal | 7.58 |
| MEFV | Osteoporosis, Postmenopausal | 7.58 |
| MLH3 | Osteoporosis, Postmenopausal | 7.58 |
| MRPL47 | Osteoporosis, Postmenopausal | 7.58 |
| NDUFB1 | Osteoporosis, Postmenopausal | 7.58 |
| NOP14 | Osteoporosis, Postmenopausal | 7.58 |
| PIGU | Osteoporosis, Postmenopausal | 7.58 |
| RFX2 | Osteoporosis, Postmenopausal | 7.58 |
| SLAMF9 | Osteoporosis, Postmenopausal | 7.58 |
| SOX7 | Osteoporosis, Postmenopausal | 7.58 |
| TBX6 | Osteoporosis, Postmenopausal | 7.58 |
| TLL1 | Osteoporosis, Postmenopausal | 7.58 |
| TSPAN31 | Osteoporosis, Postmenopausal | 7.58 |
| VPS54 | Osteoporosis, Postmenopausal | 7.58 |
| AMN1 | Osteoporosis, Postmenopausal | 7.57 |
| AVEN | Osteoporosis, Postmenopausal | 7.57 |
| BICDL1 | Osteoporosis, Postmenopausal | 7.57 |
| CHMP2B | Osteoporosis, Postmenopausal | 7.57 |
| CLIC2 | Osteoporosis, Postmenopausal | 7.57 |
| COPS7A | Osteoporosis, Postmenopausal | 7.57 |
| IRAG1 | Osteoporosis, Postmenopausal | 7.57 |
| JAKMIP1 | Osteoporosis, Postmenopausal | 7.57 |
| LILRB4 | Osteoporosis, Postmenopausal | 7.57 |
| MTCL1 | Osteoporosis, Postmenopausal | 7.57 |
| NPHS2 | Osteoporosis, Postmenopausal | 7.57 |
| PI4KB | Osteoporosis, Postmenopausal | 7.57 |
| SGCD | Osteoporosis, Postmenopausal | 7.57 |
| ST6GALNAC5 | Osteoporosis, Postmenopausal | 7.57 |
| TMEM218 | Osteoporosis, Postmenopausal | 7.57 |
| TTL | Osteoporosis, Postmenopausal | 7.57 |
| TUBE1 | Osteoporosis, Postmenopausal | 7.57 |
| URB1 | Osteoporosis, Postmenopausal | 7.57 |
| ARTN | Osteoporosis, Postmenopausal | 7.56 |
| BEND3 | Osteoporosis, Postmenopausal | 7.56 |
| BHMT2 | Osteoporosis, Postmenopausal | 7.56 |
| BRD8 | Osteoporosis, Postmenopausal | 7.56 |
| CALCRL | Osteoporosis, Postmenopausal | 7.56 |
| CARS2 | Osteoporosis, Postmenopausal | 7.56 |
| CDC23 | Osteoporosis, Postmenopausal | 7.56 |
| COG3 | Osteoporosis, Postmenopausal | 7.56 |
| DNAAF2 | Osteoporosis, Postmenopausal | 7.56 |
| FXYD7 | Osteoporosis, Postmenopausal | 7.56 |
| HASPIN | Osteoporosis, Postmenopausal | 7.56 |
| MCOLN1 | Osteoporosis, Postmenopausal | 7.56 |
| MEDAG | Osteoporosis, Postmenopausal | 7.56 |
| MIR34C | Osteoporosis, Postmenopausal | 7.56 |
| MRPS18A | Osteoporosis, Postmenopausal | 7.56 |
| MYNN | Osteoporosis, Postmenopausal | 7.56 |
| NEDD8 | Osteoporosis, Postmenopausal | 7.56 |
| OTX1 | Osteoporosis, Postmenopausal | 7.56 |
| RPP40 | Osteoporosis, Postmenopausal | 7.56 |
| SEMA6C | Osteoporosis, Postmenopausal | 7.56 |
| SLC22A9 | Osteoporosis, Postmenopausal | 7.56 |
| SLC25A47 | Osteoporosis, Postmenopausal | 7.56 |
| STAM2 | Osteoporosis, Postmenopausal | 7.56 |
| TMEM130 | Osteoporosis, Postmenopausal | 7.56 |
| TRNP1 | Osteoporosis, Postmenopausal | 7.56 |
| TUBB1 | Osteoporosis, Postmenopausal | 7.56 |
| UBR4 | Osteoporosis, Postmenopausal | 7.56 |
| UNC13C | Osteoporosis, Postmenopausal | 7.56 |
| VSX1 | Osteoporosis, Postmenopausal | 7.56 |
| XRN2 | Osteoporosis, Postmenopausal | 7.56 |
| ZFHX2 | Osteoporosis, Postmenopausal | 7.56 |
| ADRA2B | Osteoporosis, Postmenopausal | 7.55 |
| ATP9A | Osteoporosis, Postmenopausal | 7.55 |
| CCR4 | Osteoporosis, Postmenopausal | 7.55 |
| GABARAP | Osteoporosis, Postmenopausal | 7.55 |
| ITPKC | Osteoporosis, Postmenopausal | 7.55 |
| MBD6 | Osteoporosis, Postmenopausal | 7.55 |
| MIR125A | Osteoporosis, Postmenopausal | 7.55 |
| NEDD1 | Osteoporosis, Postmenopausal | 7.55 |
| ORC2 | Osteoporosis, Postmenopausal | 7.55 |
| SULT1D1 | Osteoporosis, Postmenopausal | 7.55 |
| WDR47 | Osteoporosis, Postmenopausal | 7.55 |
| ALS2CL | Osteoporosis, Postmenopausal | 7.54 |
| ARHGAP4 | Osteoporosis, Postmenopausal | 7.54 |
| ARSJ | Osteoporosis, Postmenopausal | 7.54 |
| CD200R1 | Osteoporosis, Postmenopausal | 7.54 |
| CEACAM6 | Osteoporosis, Postmenopausal | 7.54 |
| EXTL3 | Osteoporosis, Postmenopausal | 7.54 |
| FITM1 | Osteoporosis, Postmenopausal | 7.54 |
| GALC | Osteoporosis, Postmenopausal | 7.54 |
| HAUS8 | Osteoporosis, Postmenopausal | 7.54 |
| IL18R1 | Osteoporosis, Postmenopausal | 7.54 |
| LHX1 | Osteoporosis, Postmenopausal | 7.54 |
| MED20 | Osteoporosis, Postmenopausal | 7.54 |
| NT5DC3 | Osteoporosis, Postmenopausal | 7.54 |
| NUP54 | Osteoporosis, Postmenopausal | 7.54 |
| PRRG1 | Osteoporosis, Postmenopausal | 7.54 |
| PTGDR | Osteoporosis, Postmenopausal | 7.54 |
| RP2 | Osteoporosis, Postmenopausal | 7.54 |
| RPP25 | Osteoporosis, Postmenopausal | 7.54 |
| SEMA4A | Osteoporosis, Postmenopausal | 7.54 |
| SFXN4 | Osteoporosis, Postmenopausal | 7.54 |
| SHC2 | Osteoporosis, Postmenopausal | 7.54 |
| SPTA1 | Osteoporosis, Postmenopausal | 7.54 |
| XBP1 | Osteoporosis, Postmenopausal | 7.54 |
| ZNF503 | Osteoporosis, Postmenopausal | 7.54 |
| ZP3 | Osteoporosis, Postmenopausal | 7.54 |
| ACVR1 | Osteoporosis, Postmenopausal | 7.53 |
| ADAMTS14 | Osteoporosis, Postmenopausal | 7.53 |
| ALG8 | Osteoporosis, Postmenopausal | 7.53 |
| ANKRD28 | Osteoporosis, Postmenopausal | 7.53 |
| APOO | Osteoporosis, Postmenopausal | 7.53 |
| ARHGAP27 | Osteoporosis, Postmenopausal | 7.53 |
| BNIP1 | Osteoporosis, Postmenopausal | 7.53 |
| BRWD3 | Osteoporosis, Postmenopausal | 7.53 |
| CACNA1E | Osteoporosis, Postmenopausal | 7.53 |
| CACNG6 | Osteoporosis, Postmenopausal | 7.53 |
| CARF | Osteoporosis, Postmenopausal | 7.53 |
| CNOT7 | Osteoporosis, Postmenopausal | 7.53 |
| COX11 | Osteoporosis, Postmenopausal | 7.53 |
| DDX52 | Osteoporosis, Postmenopausal | 7.53 |
| DYNC2I2 | Osteoporosis, Postmenopausal | 7.53 |
| ENSA | Osteoporosis, Postmenopausal | 7.53 |
| FGF11 | Osteoporosis, Postmenopausal | 7.53 |
| GBP3 | Osteoporosis, Postmenopausal | 7.53 |
| GBX2 | Osteoporosis, Postmenopausal | 7.53 |
| H3C10 | Osteoporosis, Postmenopausal | 7.53 |
| HOXD1 | Osteoporosis, Postmenopausal | 7.53 |
| MIGA1 | Osteoporosis, Postmenopausal | 7.53 |
| MTERF3 | Osteoporosis, Postmenopausal | 7.53 |
| NOVA2 | Osteoporosis, Postmenopausal | 7.53 |
| NUP85 | Osteoporosis, Postmenopausal | 7.53 |
| PAQR3 | Osteoporosis, Postmenopausal | 7.53 |
| PARP16 | Osteoporosis, Postmenopausal | 7.53 |
| PLD5 | Osteoporosis, Postmenopausal | 7.53 |
| PPHLN1 | Osteoporosis, Postmenopausal | 7.53 |
| PSD | Osteoporosis, Postmenopausal | 7.53 |
| QTRT2 | Osteoporosis, Postmenopausal | 7.53 |
| RBM22 | Osteoporosis, Postmenopausal | 7.53 |
| RSRC1 | Osteoporosis, Postmenopausal | 7.53 |
| SEC23IP | Osteoporosis, Postmenopausal | 7.53 |
| SETD1A | Osteoporosis, Postmenopausal | 7.53 |
| SF3B5 | Osteoporosis, Postmenopausal | 7.53 |
| SMAP1 | Osteoporosis, Postmenopausal | 7.53 |
| TMEM268 | Osteoporosis, Postmenopausal | 7.53 |
| VBP1 | Osteoporosis, Postmenopausal | 7.53 |
| VPS36 | Osteoporosis, Postmenopausal | 7.53 |
| YME1L1 | Osteoporosis, Postmenopausal | 7.53 |
| YOD1 | Osteoporosis, Postmenopausal | 7.53 |
| ZBTB41 | Osteoporosis, Postmenopausal | 7.53 |
| ACOT11 | Osteoporosis, Postmenopausal | 7.52 |
| ADTRP | Osteoporosis, Postmenopausal | 7.52 |
| C2CD5 | Osteoporosis, Postmenopausal | 7.52 |
| CWC22 | Osteoporosis, Postmenopausal | 7.52 |
| DISP1 | Osteoporosis, Postmenopausal | 7.52 |
| ESRRB | Osteoporosis, Postmenopausal | 7.52 |
| GREB1L | Osteoporosis, Postmenopausal | 7.52 |
| HDAC11 | Osteoporosis, Postmenopausal | 7.52 |
| INSC | Osteoporosis, Postmenopausal | 7.52 |
| LILRB3 | Osteoporosis, Postmenopausal | 7.52 |
| MAST2 | Osteoporosis, Postmenopausal | 7.52 |
| RAB37 | Osteoporosis, Postmenopausal | 7.52 |
| RHEBL1 | Osteoporosis, Postmenopausal | 7.52 |
| SIGLEC1 | Osteoporosis, Postmenopausal | 7.52 |
| SLC22A17 | Osteoporosis, Postmenopausal | 7.52 |
| SLITRK6 | Osteoporosis, Postmenopausal | 7.52 |
| SNAPC1 | Osteoporosis, Postmenopausal | 7.52 |
| A1BG | Osteoporosis, Postmenopausal | 7.51 |
| ACER3 | Osteoporosis, Postmenopausal | 7.51 |
| CACNA2D2 | Osteoporosis, Postmenopausal | 7.51 |
| CEP170 | Osteoporosis, Postmenopausal | 7.51 |
| COIL | Osteoporosis, Postmenopausal | 7.51 |
| GDF9 | Osteoporosis, Postmenopausal | 7.51 |
| GFRA3 | Osteoporosis, Postmenopausal | 7.51 |
| HAUS6 | Osteoporosis, Postmenopausal | 7.51 |
| HNF4G | Osteoporosis, Postmenopausal | 7.51 |
| IMP3 | Osteoporosis, Postmenopausal | 7.51 |
| INPP5B | Osteoporosis, Postmenopausal | 7.51 |
| MYBPC3 | Osteoporosis, Postmenopausal | 7.51 |
| NDFIP2 | Osteoporosis, Postmenopausal | 7.51 |
| RBKS | Osteoporosis, Postmenopausal | 7.51 |
| SLC25A39 | Osteoporosis, Postmenopausal | 7.51 |
| SLCO5A1 | Osteoporosis, Postmenopausal | 7.51 |
| WFDC1 | Osteoporosis, Postmenopausal | 7.51 |
| BDP1 | Osteoporosis, Postmenopausal | 7.5 |
| CDH15 | Osteoporosis, Postmenopausal | 7.5 |
| DCAF4 | Osteoporosis, Postmenopausal | 7.5 |
| E2F6 | Osteoporosis, Postmenopausal | 7.5 |
| ELOVL4 | Osteoporosis, Postmenopausal | 7.5 |
| FAM72A | Osteoporosis, Postmenopausal | 7.5 |
| GYPA | Osteoporosis, Postmenopausal | 7.5 |
| MED6 | Osteoporosis, Postmenopausal | 7.5 |
| OGFOD1 | Osteoporosis, Postmenopausal | 7.5 |
| ORMDL2 | Osteoporosis, Postmenopausal | 7.5 |
| PKN2 | Osteoporosis, Postmenopausal | 7.5 |
| PPP2R2D | Osteoporosis, Postmenopausal | 7.5 |
| PRRT1 | Osteoporosis, Postmenopausal | 7.5 |
| RAMP3 | Osteoporosis, Postmenopausal | 7.5 |
| RASL10B | Osteoporosis, Postmenopausal | 7.5 |
| SAMD5 | Osteoporosis, Postmenopausal | 7.5 |
| SAP30L | Osteoporosis, Postmenopausal | 7.5 |
| SCAMP4 | Osteoporosis, Postmenopausal | 7.5 |
| SHROOM4 | Osteoporosis, Postmenopausal | 7.5 |
| SLC35G2 | Osteoporosis, Postmenopausal | 7.5 |
| SUN1 | Osteoporosis, Postmenopausal | 7.5 |
| TBX5 | Osteoporosis, Postmenopausal | 7.5 |
| TMEM120B | Osteoporosis, Postmenopausal | 7.5 |
| TNNI1 | Osteoporosis, Postmenopausal | 7.5 |
| VRK2 | Osteoporosis, Postmenopausal | 7.5 |
| XPNPEP3 | Osteoporosis, Postmenopausal | 7.5 |
| ZFP90 | Osteoporosis, Postmenopausal | 7.5 |
| ZIC3 | Osteoporosis, Postmenopausal | 7.5 |
| ARHGEF37 | Osteoporosis, Postmenopausal | 7.49 |
| ATOSB | Osteoporosis, Postmenopausal | 7.49 |
| BRIX1 | Osteoporosis, Postmenopausal | 7.49 |
| CACNA1B | Osteoporosis, Postmenopausal | 7.49 |
| CENPL | Osteoporosis, Postmenopausal | 7.49 |
| CYRIA | Osteoporosis, Postmenopausal | 7.49 |
| DDX11 | Osteoporosis, Postmenopausal | 7.49 |
| ELP4 | Osteoporosis, Postmenopausal | 7.49 |
| GNA11 | Osteoporosis, Postmenopausal | 7.49 |
| HOXB2 | Osteoporosis, Postmenopausal | 7.49 |
| HVCN1 | Osteoporosis, Postmenopausal | 7.49 |
| ITCH | Osteoporosis, Postmenopausal | 7.49 |
| KLHL4 | Osteoporosis, Postmenopausal | 7.49 |
| MELTF | Osteoporosis, Postmenopausal | 7.49 |
| NAP1L5 | Osteoporosis, Postmenopausal | 7.49 |
| NUDT6 | Osteoporosis, Postmenopausal | 7.49 |
| ORC4 | Osteoporosis, Postmenopausal | 7.49 |
| PAQR7 | Osteoporosis, Postmenopausal | 7.49 |
| POLRMT | Osteoporosis, Postmenopausal | 7.49 |
| S100A3 | Osteoporosis, Postmenopausal | 7.49 |
| SMAGP | Osteoporosis, Postmenopausal | 7.49 |
| TNFAIP8L3 | Osteoporosis, Postmenopausal | 7.49 |
| ARHGEF5 | Osteoporosis, Postmenopausal | 7.48 |
| CAPRIN2 | Osteoporosis, Postmenopausal | 7.48 |
| CASD1 | Osteoporosis, Postmenopausal | 7.48 |
| CCNK | Osteoporosis, Postmenopausal | 7.48 |
| CENPX | Osteoporosis, Postmenopausal | 7.48 |
| CRYZL1 | Osteoporosis, Postmenopausal | 7.48 |
| CSTF2 | Osteoporosis, Postmenopausal | 7.48 |
| EOGT | Osteoporosis, Postmenopausal | 7.48 |
| GJA3 | Osteoporosis, Postmenopausal | 7.48 |
| KLC2 | Osteoporosis, Postmenopausal | 7.48 |
| MIA | Osteoporosis, Postmenopausal | 7.48 |
| MICALL2 | Osteoporosis, Postmenopausal | 7.48 |
| NINJ2 | Osteoporosis, Postmenopausal | 7.48 |
| NUFIP2 | Osteoporosis, Postmenopausal | 7.48 |
| POMP | Osteoporosis, Postmenopausal | 7.48 |
| SAFB2 | Osteoporosis, Postmenopausal | 7.48 |
| SIAH1 | Osteoporosis, Postmenopausal | 7.48 |
| SNHG8 | Osteoporosis, Postmenopausal | 7.48 |
| USP4 | Osteoporosis, Postmenopausal | 7.48 |
| ACAP2 | Osteoporosis, Postmenopausal | 7.47 |
| ALG3 | Osteoporosis, Postmenopausal | 7.47 |
| ARID3B | Osteoporosis, Postmenopausal | 7.47 |
| BABAM2 | Osteoporosis, Postmenopausal | 7.47 |
| BARX2 | Osteoporosis, Postmenopausal | 7.47 |
| BCL11B | Osteoporosis, Postmenopausal | 7.47 |
| CCDC141 | Osteoporosis, Postmenopausal | 7.47 |
| CCDC85A | Osteoporosis, Postmenopausal | 7.47 |
| CCL21 | Osteoporosis, Postmenopausal | 7.47 |
| CDCA4 | Osteoporosis, Postmenopausal | 7.47 |
| CMSS1 | Osteoporosis, Postmenopausal | 7.47 |
| DNAJB13 | Osteoporosis, Postmenopausal | 7.47 |
| EFCAB2 | Osteoporosis, Postmenopausal | 7.47 |
| EIF3K | Osteoporosis, Postmenopausal | 7.47 |
| ENPP4 | Osteoporosis, Postmenopausal | 7.47 |
| FERMT3 | Osteoporosis, Postmenopausal | 7.47 |
| FGD5 | Osteoporosis, Postmenopausal | 7.47 |
| FOXK1 | Osteoporosis, Postmenopausal | 7.47 |
| HOXA9 | Osteoporosis, Postmenopausal | 7.47 |
| KNOP1 | Osteoporosis, Postmenopausal | 7.47 |
| LAG3 | Osteoporosis, Postmenopausal | 7.47 |
| LDLRAP1 | Osteoporosis, Postmenopausal | 7.47 |
| MFSD6 | Osteoporosis, Postmenopausal | 7.47 |
| MIR200B | Osteoporosis, Postmenopausal | 7.47 |
| MIR93 | Osteoporosis, Postmenopausal | 7.47 |
| MOK | Osteoporosis, Postmenopausal | 7.47 |
| MOSPD3 | Osteoporosis, Postmenopausal | 7.47 |
| MRAP2 | Osteoporosis, Postmenopausal | 7.47 |
| NOL10 | Osteoporosis, Postmenopausal | 7.47 |
| NOL4L | Osteoporosis, Postmenopausal | 7.47 |
| PFDN5 | Osteoporosis, Postmenopausal | 7.47 |
| PHF11 | Osteoporosis, Postmenopausal | 7.47 |
| PLEKHB2 | Osteoporosis, Postmenopausal | 7.47 |
| PPP1R12B | Osteoporosis, Postmenopausal | 7.47 |
| RBP2 | Osteoporosis, Postmenopausal | 7.47 |
| SAMSN1 | Osteoporosis, Postmenopausal | 7.47 |
| SHISA5 | Osteoporosis, Postmenopausal | 7.47 |
| SLC25A28 | Osteoporosis, Postmenopausal | 7.47 |
| SSH2 | Osteoporosis, Postmenopausal | 7.47 |
| TMEM164 | Osteoporosis, Postmenopausal | 7.47 |
| TMEM201 | Osteoporosis, Postmenopausal | 7.47 |
| UBAC1 | Osteoporosis, Postmenopausal | 7.47 |
| UROC1 | Osteoporosis, Postmenopausal | 7.47 |
| ABCB11 | Osteoporosis, Postmenopausal | 7.46 |
| ACSM5 | Osteoporosis, Postmenopausal | 7.46 |
| AMT | Osteoporosis, Postmenopausal | 7.46 |
| ART4 | Osteoporosis, Postmenopausal | 7.46 |
| CLDN15 | Osteoporosis, Postmenopausal | 7.46 |
| CPNE3 | Osteoporosis, Postmenopausal | 7.46 |
| FPR2 | Osteoporosis, Postmenopausal | 7.46 |
| GPR35 | Osteoporosis, Postmenopausal | 7.46 |
| H2-AB1 | Osteoporosis, Postmenopausal | 7.46 |
| HECTD2 | Osteoporosis, Postmenopausal | 7.46 |
| HPS5 | Osteoporosis, Postmenopausal | 7.46 |
| KIFC2 | Osteoporosis, Postmenopausal | 7.46 |
| ONECUT2 | Osteoporosis, Postmenopausal | 7.46 |
| PKD1 | Osteoporosis, Postmenopausal | 7.46 |
| RALY | Osteoporosis, Postmenopausal | 7.46 |
| RNF8 | Osteoporosis, Postmenopausal | 7.46 |
| SCFD2 | Osteoporosis, Postmenopausal | 7.46 |
| SFTPC | Osteoporosis, Postmenopausal | 7.46 |
| SMC5 | Osteoporosis, Postmenopausal | 7.46 |
| TMEFF1 | Osteoporosis, Postmenopausal | 7.46 |
| TRPC1 | Osteoporosis, Postmenopausal | 7.46 |
| ALG14 | Osteoporosis, Postmenopausal | 7.45 |
| ANKRD26 | Osteoporosis, Postmenopausal | 7.45 |
| BICD2 | Osteoporosis, Postmenopausal | 7.45 |
| EMC9 | Osteoporosis, Postmenopausal | 7.45 |
| FANCL | Osteoporosis, Postmenopausal | 7.45 |
| FBXL3 | Osteoporosis, Postmenopausal | 7.45 |
| FEM1B | Osteoporosis, Postmenopausal | 7.45 |
| FGFR1OP2 | Osteoporosis, Postmenopausal | 7.45 |
| GPR17 | Osteoporosis, Postmenopausal | 7.45 |
| KCNK12 | Osteoporosis, Postmenopausal | 7.45 |
| KLF16 | Osteoporosis, Postmenopausal | 7.45 |
| MAP4K3 | Osteoporosis, Postmenopausal | 7.45 |
| METTL8 | Osteoporosis, Postmenopausal | 7.45 |
| MMADHC | Osteoporosis, Postmenopausal | 7.45 |
| MRPS2 | Osteoporosis, Postmenopausal | 7.45 |
| MS4A7 | Osteoporosis, Postmenopausal | 7.45 |
| NMD3 | Osteoporosis, Postmenopausal | 7.45 |
| PITPNA | Osteoporosis, Postmenopausal | 7.45 |
| POLR3B | Osteoporosis, Postmenopausal | 7.45 |
| PYGO2 | Osteoporosis, Postmenopausal | 7.45 |
| SMYD5 | Osteoporosis, Postmenopausal | 7.45 |
| SOX3 | Osteoporosis, Postmenopausal | 7.45 |
| UNC5D | Osteoporosis, Postmenopausal | 7.45 |
| VPS8 | Osteoporosis, Postmenopausal | 7.45 |
| CLCN2 | Osteoporosis, Postmenopausal | 7.44 |
| CNPY2 | Osteoporosis, Postmenopausal | 7.44 |
| CYTH1 | Osteoporosis, Postmenopausal | 7.44 |
| DTWD1 | Osteoporosis, Postmenopausal | 7.44 |
| GNAT2 | Osteoporosis, Postmenopausal | 7.44 |
| GNG8 | Osteoporosis, Postmenopausal | 7.44 |
| GRM8 | Osteoporosis, Postmenopausal | 7.44 |
| LMO3 | Osteoporosis, Postmenopausal | 7.44 |
| MAN2A2 | Osteoporosis, Postmenopausal | 7.44 |
| NDRG3 | Osteoporosis, Postmenopausal | 7.44 |
| NUDT19 | Osteoporosis, Postmenopausal | 7.44 |
| PRG2 | Osteoporosis, Postmenopausal | 7.44 |
| RNF150 | Osteoporosis, Postmenopausal | 7.44 |
| SLC9A2 | Osteoporosis, Postmenopausal | 7.44 |
| TRF | Osteoporosis, Postmenopausal | 7.44 |
| AGMAT | Osteoporosis, Postmenopausal | 7.43 |
| AKIRIN2 | Osteoporosis, Postmenopausal | 7.43 |
| AMDHD1 | Osteoporosis, Postmenopausal | 7.43 |
| ASIC1 | Osteoporosis, Postmenopausal | 7.43 |
| BCL9 | Osteoporosis, Postmenopausal | 7.43 |
| GNPTAB | Osteoporosis, Postmenopausal | 7.43 |
| KRIT1 | Osteoporosis, Postmenopausal | 7.43 |
| MED21 | Osteoporosis, Postmenopausal | 7.43 |
| MRTFB | Osteoporosis, Postmenopausal | 7.43 |
| NAALAD2 | Osteoporosis, Postmenopausal | 7.43 |
| PRELID2 | Osteoporosis, Postmenopausal | 7.43 |
| RIPPLY3 | Osteoporosis, Postmenopausal | 7.43 |
| UGT1A2 | Osteoporosis, Postmenopausal | 7.43 |
| USP13 | Osteoporosis, Postmenopausal | 7.43 |
| ABCB7 | Osteoporosis, Postmenopausal | 7.42 |
| AKAP10 | Osteoporosis, Postmenopausal | 7.42 |
| B3GALT1 | Osteoporosis, Postmenopausal | 7.42 |
| BCS1L | Osteoporosis, Postmenopausal | 7.42 |
| CHRNA1 | Osteoporosis, Postmenopausal | 7.42 |
| CYYR1 | Osteoporosis, Postmenopausal | 7.42 |
| DBNDD2 | Osteoporosis, Postmenopausal | 7.42 |
| DLX3 | Osteoporosis, Postmenopausal | 7.42 |
| DOCK2 | Osteoporosis, Postmenopausal | 7.42 |
| FCGR2A | Osteoporosis, Postmenopausal | 7.42 |
| GGT5 | Osteoporosis, Postmenopausal | 7.42 |
| GXYLT2 | Osteoporosis, Postmenopausal | 7.42 |
| HM13 | Osteoporosis, Postmenopausal | 7.42 |
| LGALS7 | Osteoporosis, Postmenopausal | 7.42 |
| LYPLA1 | Osteoporosis, Postmenopausal | 7.42 |
| MIR145 | Osteoporosis, Postmenopausal | 7.42 |
| MRPL11 | Osteoporosis, Postmenopausal | 7.42 |
| MRPL40 | Osteoporosis, Postmenopausal | 7.42 |
| MYH2 | Osteoporosis, Postmenopausal | 7.42 |
| PACC1 | Osteoporosis, Postmenopausal | 7.42 |
| POGLUT1 | Osteoporosis, Postmenopausal | 7.42 |
| RAB11FIP2 | Osteoporosis, Postmenopausal | 7.42 |
| RCBTB2 | Osteoporosis, Postmenopausal | 7.42 |
| ROBO3 | Osteoporosis, Postmenopausal | 7.42 |
| SBF1 | Osteoporosis, Postmenopausal | 7.42 |
| SLC5A8 | Osteoporosis, Postmenopausal | 7.42 |
| STON1 | Osteoporosis, Postmenopausal | 7.42 |
| SUSD1 | Osteoporosis, Postmenopausal | 7.42 |
| TPRKB | Osteoporosis, Postmenopausal | 7.42 |
| TSN | Osteoporosis, Postmenopausal | 7.42 |
| USP25 | Osteoporosis, Postmenopausal | 7.42 |
| ZC3H12C | Osteoporosis, Postmenopausal | 7.42 |
| AHCYL2 | Osteoporosis, Postmenopausal | 7.41 |
| CCNO | Osteoporosis, Postmenopausal | 7.41 |
| DNASE1 | Osteoporosis, Postmenopausal | 7.41 |
| EFNA4 | Osteoporosis, Postmenopausal | 7.41 |
| ELAVL3 | Osteoporosis, Postmenopausal | 7.41 |
| H2BC8 | Osteoporosis, Postmenopausal | 7.41 |
| KIF26B | Osteoporosis, Postmenopausal | 7.41 |
| KLHL7 | Osteoporosis, Postmenopausal | 7.41 |
| MAP3K13 | Osteoporosis, Postmenopausal | 7.41 |
| MARS2 | Osteoporosis, Postmenopausal | 7.41 |
| PHF3 | Osteoporosis, Postmenopausal | 7.41 |
| PHKA2 | Osteoporosis, Postmenopausal | 7.41 |
| RNPEP | Osteoporosis, Postmenopausal | 7.41 |
| SLC19A3 | Osteoporosis, Postmenopausal | 7.41 |
| TRPC4 | Osteoporosis, Postmenopausal | 7.41 |
| UBA3 | Osteoporosis, Postmenopausal | 7.41 |
| ZDHHC21 | Osteoporosis, Postmenopausal | 7.41 |
| ZNF652 | Osteoporosis, Postmenopausal | 7.41 |
| ADAMTSL1 | Osteoporosis, Postmenopausal | 7.4 |
| CAB39 | Osteoporosis, Postmenopausal | 7.4 |
| CCDC18 | Osteoporosis, Postmenopausal | 7.4 |
| CTF1 | Osteoporosis, Postmenopausal | 7.4 |
| DNMBP | Osteoporosis, Postmenopausal | 7.4 |
| EIF3L | Osteoporosis, Postmenopausal | 7.4 |
| GNPNAT1 | Osteoporosis, Postmenopausal | 7.4 |
| GPR161 | Osteoporosis, Postmenopausal | 7.4 |
| GPR19 | Osteoporosis, Postmenopausal | 7.4 |
| HAUS2 | Osteoporosis, Postmenopausal | 7.4 |
| HLA-E | Osteoporosis, Postmenopausal | 7.4 |
| IFT25 | Osteoporosis, Postmenopausal | 7.4 |
| MED23 | Osteoporosis, Postmenopausal | 7.4 |
| NDUFA4L2 | Osteoporosis, Postmenopausal | 7.4 |
| NSMCE4A | Osteoporosis, Postmenopausal | 7.4 |
| RABGEF1 | Osteoporosis, Postmenopausal | 7.4 |
| RASSF7 | Osteoporosis, Postmenopausal | 7.4 |
| RBM34 | Osteoporosis, Postmenopausal | 7.4 |
| SDCCAG8 | Osteoporosis, Postmenopausal | 7.4 |
| SLITRK5 | Osteoporosis, Postmenopausal | 7.4 |
| SNCAIP | Osteoporosis, Postmenopausal | 7.4 |
| SNHG12 | Osteoporosis, Postmenopausal | 7.4 |
| SNTB2 | Osteoporosis, Postmenopausal | 7.4 |
| STOML2 | Osteoporosis, Postmenopausal | 7.4 |
| TMEM132A | Osteoporosis, Postmenopausal | 7.4 |
| TOM1 | Osteoporosis, Postmenopausal | 7.4 |
| UHRF2 | Osteoporosis, Postmenopausal | 7.4 |
| VTCN1 | Osteoporosis, Postmenopausal | 7.4 |
| ZFAND6 | Osteoporosis, Postmenopausal | 7.4 |
| AP3M2 | Osteoporosis, Postmenopausal | 7.39 |
| ARK2C | Osteoporosis, Postmenopausal | 7.39 |
| CNOT2 | Osteoporosis, Postmenopausal | 7.39 |
| CTSO | Osteoporosis, Postmenopausal | 7.39 |
| FPR1 | Osteoporosis, Postmenopausal | 7.39 |
| FRMD5 | Osteoporosis, Postmenopausal | 7.39 |
| GABRA3 | Osteoporosis, Postmenopausal | 7.39 |
| GSTM6 | Osteoporosis, Postmenopausal | 7.39 |
| H1F2 | Osteoporosis, Postmenopausal | 7.39 |
| HYI | Osteoporosis, Postmenopausal | 7.39 |
| IRAK1BP1 | Osteoporosis, Postmenopausal | 7.39 |
| MBOAT7 | Osteoporosis, Postmenopausal | 7.39 |
| MRM3 | Osteoporosis, Postmenopausal | 7.39 |
| PI16 | Osteoporosis, Postmenopausal | 7.39 |
| PLAG1 | Osteoporosis, Postmenopausal | 7.39 |
| POLR1G | Osteoporosis, Postmenopausal | 7.39 |
| RASSF6 | Osteoporosis, Postmenopausal | 7.39 |
| RIT1 | Osteoporosis, Postmenopausal | 7.39 |
| RYR3 | Osteoporosis, Postmenopausal | 7.39 |
| SIX4 | Osteoporosis, Postmenopausal | 7.39 |
| SNX7 | Osteoporosis, Postmenopausal | 7.39 |
| SOCS6 | Osteoporosis, Postmenopausal | 7.39 |
| TM7SF3 | Osteoporosis, Postmenopausal | 7.39 |
| VWCE | Osteoporosis, Postmenopausal | 7.39 |
| AHR2 | Osteoporosis, Postmenopausal | 7.38 |
| AP1M2 | Osteoporosis, Postmenopausal | 7.38 |
| CTNNA2 | Osteoporosis, Postmenopausal | 7.38 |
| DYNC1LI2 | Osteoporosis, Postmenopausal | 7.38 |
| HS6ST2 | Osteoporosis, Postmenopausal | 7.38 |
| KRT6A | Osteoporosis, Postmenopausal | 7.38 |
| PARP2 | Osteoporosis, Postmenopausal | 7.38 |
| PAX5 | Osteoporosis, Postmenopausal | 7.38 |
| RDM1 | Osteoporosis, Postmenopausal | 7.38 |
| TOR1AIP1 | Osteoporosis, Postmenopausal | 7.38 |
| A4GALT | Osteoporosis, Postmenopausal | 7.37 |
| ADAT2 | Osteoporosis, Postmenopausal | 7.37 |
| ATF4 | Osteoporosis, Postmenopausal | 7.37 |
| ATP6V1C2 | Osteoporosis, Postmenopausal | 7.37 |
| BOLA3 | Osteoporosis, Postmenopausal | 7.37 |
| CCDC47 | Osteoporosis, Postmenopausal | 7.37 |
| CHRND | Osteoporosis, Postmenopausal | 7.37 |
| CYSLTR1 | Osteoporosis, Postmenopausal | 7.37 |
| DDX27 | Osteoporosis, Postmenopausal | 7.37 |
| DOK7 | Osteoporosis, Postmenopausal | 7.37 |
| ELOA | Osteoporosis, Postmenopausal | 7.37 |
| EPHA8 | Osteoporosis, Postmenopausal | 7.37 |
| GPATCH2 | Osteoporosis, Postmenopausal | 7.37 |
| HS3ST2 | Osteoporosis, Postmenopausal | 7.37 |
| KRR1 | Osteoporosis, Postmenopausal | 7.37 |
| MARCHF7 | Osteoporosis, Postmenopausal | 7.37 |
| MCRIP2 | Osteoporosis, Postmenopausal | 7.37 |
| MIR17HG | Osteoporosis, Postmenopausal | 7.37 |
| MLX | Osteoporosis, Postmenopausal | 7.37 |
| MMP17 | Osteoporosis, Postmenopausal | 7.37 |
| NCKAP1 | Osteoporosis, Postmenopausal | 7.37 |
| NOP53 | Osteoporosis, Postmenopausal | 7.37 |
| P2RY12 | Osteoporosis, Postmenopausal | 7.37 |
| PDCD1 | Osteoporosis, Postmenopausal | 7.37 |
| PINX1 | Osteoporosis, Postmenopausal | 7.37 |
| PLPP5 | Osteoporosis, Postmenopausal | 7.37 |
| RAB25 | Osteoporosis, Postmenopausal | 7.37 |
| ROBO4 | Osteoporosis, Postmenopausal | 7.37 |
| SLC41A3 | Osteoporosis, Postmenopausal | 7.37 |
| SSBP4 | Osteoporosis, Postmenopausal | 7.37 |
| TOMM34 | Osteoporosis, Postmenopausal | 7.37 |
| TXNL4B | Osteoporosis, Postmenopausal | 7.37 |
| VWA5A | Osteoporosis, Postmenopausal | 7.37 |
| WIPF3 | Osteoporosis, Postmenopausal | 7.37 |
| ZCCHC12 | Osteoporosis, Postmenopausal | 7.37 |
| AIMP1 | Osteoporosis, Postmenopausal | 7.36 |
| ARHGEF18 | Osteoporosis, Postmenopausal | 7.36 |
| BLZF1 | Osteoporosis, Postmenopausal | 7.36 |
| CDIP1 | Osteoporosis, Postmenopausal | 7.36 |
| DAB2IP | Osteoporosis, Postmenopausal | 7.36 |
| DPY30 | Osteoporosis, Postmenopausal | 7.36 |
| HNRNPLL | Osteoporosis, Postmenopausal | 7.36 |
| IRAK3 | Osteoporosis, Postmenopausal | 7.36 |
| LNX2 | Osteoporosis, Postmenopausal | 7.36 |
| MRE11 | Osteoporosis, Postmenopausal | 7.36 |
| MYH1 | Osteoporosis, Postmenopausal | 7.36 |
| PKNOX2 | Osteoporosis, Postmenopausal | 7.36 |
| PLEKHA7 | Osteoporosis, Postmenopausal | 7.36 |
| RNF39 | Osteoporosis, Postmenopausal | 7.36 |
| SLC25A27 | Osteoporosis, Postmenopausal | 7.36 |
| SUFU | Osteoporosis, Postmenopausal | 7.36 |
| TBC1D8B | Osteoporosis, Postmenopausal | 7.36 |
| TRIM21 | Osteoporosis, Postmenopausal | 7.36 |
| TUBGCP3 | Osteoporosis, Postmenopausal | 7.36 |
| WDR54 | Osteoporosis, Postmenopausal | 7.36 |
| WNT9A | Osteoporosis, Postmenopausal | 7.36 |
| ABCB9 | Osteoporosis, Postmenopausal | 7.35 |
| ADGRL1 | Osteoporosis, Postmenopausal | 7.35 |
| AKAP6 | Osteoporosis, Postmenopausal | 7.35 |
| ANKRD50 | Osteoporosis, Postmenopausal | 7.35 |
| ARHGAP8 | Osteoporosis, Postmenopausal | 7.35 |
| ARPC3 | Osteoporosis, Postmenopausal | 7.35 |
| ARPC5 | Osteoporosis, Postmenopausal | 7.35 |
| AXIN1 | Osteoporosis, Postmenopausal | 7.35 |
| CAMTA1 | Osteoporosis, Postmenopausal | 7.35 |
| CBARP | Osteoporosis, Postmenopausal | 7.35 |
| CD72 | Osteoporosis, Postmenopausal | 7.35 |
| CES2E | Osteoporosis, Postmenopausal | 7.35 |
| CPLX1 | Osteoporosis, Postmenopausal | 7.35 |
| CRTAC1 | Osteoporosis, Postmenopausal | 7.35 |
| CTNNBL1 | Osteoporosis, Postmenopausal | 7.35 |
| DAZL | Osteoporosis, Postmenopausal | 7.35 |
| DLX4 | Osteoporosis, Postmenopausal | 7.35 |
| ETV3 | Osteoporosis, Postmenopausal | 7.35 |
| HLA-F | Osteoporosis, Postmenopausal | 7.35 |
| HSD17B14 | Osteoporosis, Postmenopausal | 7.35 |
| KCNIP3 | Osteoporosis, Postmenopausal | 7.35 |
| MESP1 | Osteoporosis, Postmenopausal | 7.35 |
| MRPS18C | Osteoporosis, Postmenopausal | 7.35 |
| MTM1 | Osteoporosis, Postmenopausal | 7.35 |
| NAA50 | Osteoporosis, Postmenopausal | 7.35 |
| NDUFC1 | Osteoporosis, Postmenopausal | 7.35 |
| NINL | Osteoporosis, Postmenopausal | 7.35 |
| NUBP1 | Osteoporosis, Postmenopausal | 7.35 |
| OAZ2 | Osteoporosis, Postmenopausal | 7.35 |
| PARP3 | Osteoporosis, Postmenopausal | 7.35 |
| POLR1E | Osteoporosis, Postmenopausal | 7.35 |
| PPM1J | Osteoporosis, Postmenopausal | 7.35 |
| RAB15 | Osteoporosis, Postmenopausal | 7.35 |
| RNASEH2B | Osteoporosis, Postmenopausal | 7.35 |
| ROPN1L | Osteoporosis, Postmenopausal | 7.35 |
| SNAPIN | Osteoporosis, Postmenopausal | 7.35 |
| SNCG | Osteoporosis, Postmenopausal | 7.35 |
| SNW1 | Osteoporosis, Postmenopausal | 7.35 |
| TMEM52 | Osteoporosis, Postmenopausal | 7.35 |
| TRIL | Osteoporosis, Postmenopausal | 7.35 |
| XIRP1 | Osteoporosis, Postmenopausal | 7.35 |
| YIPF5 | Osteoporosis, Postmenopausal | 7.35 |
| ZFYVE28 | Osteoporosis, Postmenopausal | 7.35 |
| ADH6 | Osteoporosis, Postmenopausal | 7.34 |
| ARVCF | Osteoporosis, Postmenopausal | 7.34 |
| CERS5 | Osteoporosis, Postmenopausal | 7.34 |
| DSE | Osteoporosis, Postmenopausal | 7.34 |
| EIF3F | Osteoporosis, Postmenopausal | 7.34 |
| ELF2 | Osteoporosis, Postmenopausal | 7.34 |
| FMO4 | Osteoporosis, Postmenopausal | 7.34 |
| LAMTOR3 | Osteoporosis, Postmenopausal | 7.34 |
| MROH1 | Osteoporosis, Postmenopausal | 7.34 |
| MRPS22 | Osteoporosis, Postmenopausal | 7.34 |
| PYROXD2 | Osteoporosis, Postmenopausal | 7.34 |
| WAPL | Osteoporosis, Postmenopausal | 7.34 |
| YARS2 | Osteoporosis, Postmenopausal | 7.34 |
| ABTB3 | Osteoporosis, Postmenopausal | 7.33 |
| CARD6 | Osteoporosis, Postmenopausal | 7.33 |
| COMP | Osteoporosis, Postmenopausal | 7.33 |
| CREB3L1 | Osteoporosis, Postmenopausal | 7.33 |
| GRP | Osteoporosis, Postmenopausal | 7.33 |
| IRX4 | Osteoporosis, Postmenopausal | 7.33 |
| KCNAB2 | Osteoporosis, Postmenopausal | 7.33 |
| MUG1 | Osteoporosis, Postmenopausal | 7.33 |
| NFS1 | Osteoporosis, Postmenopausal | 7.33 |
| RHOBTB2 | Osteoporosis, Postmenopausal | 7.33 |
| SCOC | Osteoporosis, Postmenopausal | 7.33 |
| SCUBE2 | Osteoporosis, Postmenopausal | 7.33 |
| XCL1 | Osteoporosis, Postmenopausal | 7.33 |
| ZBTB7A | Osteoporosis, Postmenopausal | 7.33 |
| AMFR | Osteoporosis, Postmenopausal | 7.32 |
| APOL3 | Osteoporosis, Postmenopausal | 7.32 |
| ARSA | Osteoporosis, Postmenopausal | 7.32 |
| CARM1 | Osteoporosis, Postmenopausal | 7.32 |
| CD46 | Osteoporosis, Postmenopausal | 7.32 |
| CNDP1 | Osteoporosis, Postmenopausal | 7.32 |
| CNST | Osteoporosis, Postmenopausal | 7.32 |
| CNTFR | Osteoporosis, Postmenopausal | 7.32 |
| DDX20 | Osteoporosis, Postmenopausal | 7.32 |
| DUSP9 | Osteoporosis, Postmenopausal | 7.32 |
| FBXO11 | Osteoporosis, Postmenopausal | 7.32 |
| FKBP8 | Osteoporosis, Postmenopausal | 7.32 |
| GAS2L1 | Osteoporosis, Postmenopausal | 7.32 |
| GDF11 | Osteoporosis, Postmenopausal | 7.32 |
| HCLS1 | Osteoporosis, Postmenopausal | 7.32 |
| KLHDC8A | Osteoporosis, Postmenopausal | 7.32 |
| MSC | Osteoporosis, Postmenopausal | 7.32 |
| MTHFD2L | Osteoporosis, Postmenopausal | 7.32 |
| NOXO1 | Osteoporosis, Postmenopausal | 7.32 |
| NUS1 | Osteoporosis, Postmenopausal | 7.32 |
| OSER1 | Osteoporosis, Postmenopausal | 7.32 |
| PCMTD2 | Osteoporosis, Postmenopausal | 7.32 |
| PEAR1 | Osteoporosis, Postmenopausal | 7.32 |
| POLR1H | Osteoporosis, Postmenopausal | 7.32 |
| PPM1E | Osteoporosis, Postmenopausal | 7.32 |
| PRTG | Osteoporosis, Postmenopausal | 7.32 |
| RNF44 | Osteoporosis, Postmenopausal | 7.32 |
| RNF7 | Osteoporosis, Postmenopausal | 7.32 |
| SGCG | Osteoporosis, Postmenopausal | 7.32 |
| SLC25A32 | Osteoporosis, Postmenopausal | 7.32 |
| SLC39A5 | Osteoporosis, Postmenopausal | 7.32 |
| SMC6 | Osteoporosis, Postmenopausal | 7.32 |
| TPK1 | Osteoporosis, Postmenopausal | 7.32 |
| UBE2E2 | Osteoporosis, Postmenopausal | 7.32 |
| UBR1 | Osteoporosis, Postmenopausal | 7.32 |
| UNC5A | Osteoporosis, Postmenopausal | 7.32 |
| WRN | Osteoporosis, Postmenopausal | 7.32 |
| XPO6 | Osteoporosis, Postmenopausal | 7.32 |
| YES1 | Osteoporosis, Postmenopausal | 7.32 |
| ZBTB44 | Osteoporosis, Postmenopausal | 7.32 |
| ZC3H8 | Osteoporosis, Postmenopausal | 7.32 |
| ZMPSTE24 | Osteoporosis, Postmenopausal | 7.32 |
| BSPRY | Osteoporosis, Postmenopausal | 7.31 |
| CCDC69 | Osteoporosis, Postmenopausal | 7.31 |
| CHMP4C | Osteoporosis, Postmenopausal | 7.31 |
| COPZ2 | Osteoporosis, Postmenopausal | 7.31 |
| ITGA11 | Osteoporosis, Postmenopausal | 7.31 |
| KDM6A | Osteoporosis, Postmenopausal | 7.31 |
| LRRC8A | Osteoporosis, Postmenopausal | 7.31 |
| PNLIP | Osteoporosis, Postmenopausal | 7.31 |
| RAB17 | Osteoporosis, Postmenopausal | 7.31 |
| RAG1 | Osteoporosis, Postmenopausal | 7.31 |
| RNASE1 | Osteoporosis, Postmenopausal | 7.31 |
| STARD10 | Osteoporosis, Postmenopausal | 7.31 |
| ACOT8 | Osteoporosis, Postmenopausal | 7.3 |
| APBA1 | Osteoporosis, Postmenopausal | 7.3 |
| CCNT1 | Osteoporosis, Postmenopausal | 7.3 |
| CITED1 | Osteoporosis, Postmenopausal | 7.3 |
| CKMT1 | Osteoporosis, Postmenopausal | 7.3 |
| CLDND1 | Osteoporosis, Postmenopausal | 7.3 |
| CLIC6 | Osteoporosis, Postmenopausal | 7.3 |
| COPS5 | Osteoporosis, Postmenopausal | 7.3 |
| CPVL | Osteoporosis, Postmenopausal | 7.3 |
| CTDSPL2 | Osteoporosis, Postmenopausal | 7.3 |
| DDX42 | Osteoporosis, Postmenopausal | 7.3 |
| FAM171A1 | Osteoporosis, Postmenopausal | 7.3 |
| FBXL7 | Osteoporosis, Postmenopausal | 7.3 |
| FGF4 | Osteoporosis, Postmenopausal | 7.3 |
| FOXL2 | Osteoporosis, Postmenopausal | 7.3 |
| GDF6 | Osteoporosis, Postmenopausal | 7.3 |
| GPAT4 | Osteoporosis, Postmenopausal | 7.3 |
| GPR160 | Osteoporosis, Postmenopausal | 7.3 |
| IBTK | Osteoporosis, Postmenopausal | 7.3 |
| KCNE2 | Osteoporosis, Postmenopausal | 7.3 |
| KCNJ16 | Osteoporosis, Postmenopausal | 7.3 |
| KSR2 | Osteoporosis, Postmenopausal | 7.3 |
| LRP10 | Osteoporosis, Postmenopausal | 7.3 |
| MFNG | Osteoporosis, Postmenopausal | 7.3 |
| MRPS33 | Osteoporosis, Postmenopausal | 7.3 |
| MTF2 | Osteoporosis, Postmenopausal | 7.3 |
| MYOM1 | Osteoporosis, Postmenopausal | 7.3 |
| NACC1 | Osteoporosis, Postmenopausal | 7.3 |
| NPTXR | Osteoporosis, Postmenopausal | 7.3 |
| NTNG1 | Osteoporosis, Postmenopausal | 7.3 |
| P2RX1 | Osteoporosis, Postmenopausal | 7.3 |
| PLEKHH1 | Osteoporosis, Postmenopausal | 7.3 |
| PRPF3 | Osteoporosis, Postmenopausal | 7.3 |
| PRPF4 | Osteoporosis, Postmenopausal | 7.3 |
| PRSS1 | Osteoporosis, Postmenopausal | 7.3 |
| PTPRA | Osteoporosis, Postmenopausal | 7.3 |
| RAB1B | Osteoporosis, Postmenopausal | 7.3 |
| RAB3IL1 | Osteoporosis, Postmenopausal | 7.3 |
| RAPGEF6 | Osteoporosis, Postmenopausal | 7.3 |
| RBM38 | Osteoporosis, Postmenopausal | 7.3 |
| REPIN1 | Osteoporosis, Postmenopausal | 7.3 |
| RIMS1 | Osteoporosis, Postmenopausal | 7.3 |
| RLIM | Osteoporosis, Postmenopausal | 7.3 |
| RRP15 | Osteoporosis, Postmenopausal | 7.3 |
| SEH1L | Osteoporosis, Postmenopausal | 7.3 |
| SFT2D2 | Osteoporosis, Postmenopausal | 7.3 |
| SH3RF2 | Osteoporosis, Postmenopausal | 7.3 |
| SLC18A1 | Osteoporosis, Postmenopausal | 7.3 |
| SLC25A45 | Osteoporosis, Postmenopausal | 7.3 |
| SLN | Osteoporosis, Postmenopausal | 7.3 |
| TBC1D17 | Osteoporosis, Postmenopausal | 7.3 |
| TCEA1 | Osteoporosis, Postmenopausal | 7.3 |
| TRAV5 | Osteoporosis, Postmenopausal | 7.3 |
| TRIM13 | Osteoporosis, Postmenopausal | 7.3 |
| TSG101 | Osteoporosis, Postmenopausal | 7.3 |
| TSTD1 | Osteoporosis, Postmenopausal | 7.3 |
| ZFPM2 | Osteoporosis, Postmenopausal | 7.3 |
| CAB39L | Osteoporosis, Postmenopausal | 7.29 |
| CHDH | Osteoporosis, Postmenopausal | 7.29 |
| DTNB | Osteoporosis, Postmenopausal | 7.29 |
| MBTD1 | Osteoporosis, Postmenopausal | 7.29 |
| TIMM17A | Osteoporosis, Postmenopausal | 7.29 |
| ASH1L | Osteoporosis, Postmenopausal | 7.28 |
| ATE1 | Osteoporosis, Postmenopausal | 7.28 |
| BCAS2 | Osteoporosis, Postmenopausal | 7.28 |
| BLOC1S2 | Osteoporosis, Postmenopausal | 7.28 |
| CASQ2 | Osteoporosis, Postmenopausal | 7.28 |
| COMMD10 | Osteoporosis, Postmenopausal | 7.28 |
| DPP6 | Osteoporosis, Postmenopausal | 7.28 |
| GNL2 | Osteoporosis, Postmenopausal | 7.28 |
| GP1BB | Osteoporosis, Postmenopausal | 7.28 |
| IFNA1 | Osteoporosis, Postmenopausal | 7.28 |
| ILVBL | Osteoporosis, Postmenopausal | 7.28 |
| INTS3 | Osteoporosis, Postmenopausal | 7.28 |
| KAZALD1 | Osteoporosis, Postmenopausal | 7.28 |
| MAPK15 | Osteoporosis, Postmenopausal | 7.28 |
| METTL14 | Osteoporosis, Postmenopausal | 7.28 |
| NAAA | Osteoporosis, Postmenopausal | 7.28 |
| P2RX5 | Osteoporosis, Postmenopausal | 7.28 |
| P3H3 | Osteoporosis, Postmenopausal | 7.28 |
| PDCD2 | Osteoporosis, Postmenopausal | 7.28 |
| PLPBP | Osteoporosis, Postmenopausal | 7.28 |
| POLR2L | Osteoporosis, Postmenopausal | 7.28 |
| PXK | Osteoporosis, Postmenopausal | 7.28 |
| RAE1 | Osteoporosis, Postmenopausal | 7.28 |
| SARM1 | Osteoporosis, Postmenopausal | 7.28 |
| SCFD1 | Osteoporosis, Postmenopausal | 7.28 |
| SLMAP | Osteoporosis, Postmenopausal | 7.28 |
| TBC1D24 | Osteoporosis, Postmenopausal | 7.28 |
| TMX4 | Osteoporosis, Postmenopausal | 7.28 |
| TRIM36 | Osteoporosis, Postmenopausal | 7.28 |
| UPK1A | Osteoporosis, Postmenopausal | 7.28 |
| WDR5 | Osteoporosis, Postmenopausal | 7.28 |
| ZFP91 | Osteoporosis, Postmenopausal | 7.28 |
| ZFPM1 | Osteoporosis, Postmenopausal | 7.28 |
| ARFGAP1 | Osteoporosis, Postmenopausal | 7.27 |
| ATP10A | Osteoporosis, Postmenopausal | 7.27 |
| CRADD | Osteoporosis, Postmenopausal | 7.27 |
| GINS4 | Osteoporosis, Postmenopausal | 7.27 |
| GRIK3 | Osteoporosis, Postmenopausal | 7.27 |
| ITIH1 | Osteoporosis, Postmenopausal | 7.27 |
| LARP4B | Osteoporosis, Postmenopausal | 7.27 |
| MREG | Osteoporosis, Postmenopausal | 7.27 |
| NIPAL2 | Osteoporosis, Postmenopausal | 7.27 |
| PSMG1 | Osteoporosis, Postmenopausal | 7.27 |
| RAP1GAP2 | Osteoporosis, Postmenopausal | 7.27 |
| SH3GL3 | Osteoporosis, Postmenopausal | 7.27 |
| TMEM100 | Osteoporosis, Postmenopausal | 7.27 |
| TMEM255A | Osteoporosis, Postmenopausal | 7.27 |
| TREX1 | Osteoporosis, Postmenopausal | 7.27 |
| ADORA3 | Osteoporosis, Postmenopausal | 7.26 |
| ATP6V0A2 | Osteoporosis, Postmenopausal | 7.26 |
| CDH22 | Osteoporosis, Postmenopausal | 7.26 |
| CLPTM1 | Osteoporosis, Postmenopausal | 7.26 |
| COQ7 | Osteoporosis, Postmenopausal | 7.26 |
| CPXM1 | Osteoporosis, Postmenopausal | 7.26 |
| E2F4 | Osteoporosis, Postmenopausal | 7.26 |
| GPR183 | Osteoporosis, Postmenopausal | 7.26 |
| LNX1 | Osteoporosis, Postmenopausal | 7.26 |
| POLI | Osteoporosis, Postmenopausal | 7.26 |
| RAB7B | Osteoporosis, Postmenopausal | 7.26 |
| SHC3 | Osteoporosis, Postmenopausal | 7.26 |
| STX11 | Osteoporosis, Postmenopausal | 7.26 |
| TRAK2 | Osteoporosis, Postmenopausal | 7.26 |
| USP15 | Osteoporosis, Postmenopausal | 7.26 |
| AFDN | Osteoporosis, Postmenopausal | 7.25 |
| AGTRAP | Osteoporosis, Postmenopausal | 7.25 |
| CDK13 | Osteoporosis, Postmenopausal | 7.25 |
| COMMD3 | Osteoporosis, Postmenopausal | 7.25 |
| DLX1 | Osteoporosis, Postmenopausal | 7.25 |
| DPM3 | Osteoporosis, Postmenopausal | 7.25 |
| FHDC1 | Osteoporosis, Postmenopausal | 7.25 |
| FRMD3 | Osteoporosis, Postmenopausal | 7.25 |
| GAPVD1 | Osteoporosis, Postmenopausal | 7.25 |
| GEMIN6 | Osteoporosis, Postmenopausal | 7.25 |
| IARS2 | Osteoporosis, Postmenopausal | 7.25 |
| IMPDH1 | Osteoporosis, Postmenopausal | 7.25 |
| LACC1 | Osteoporosis, Postmenopausal | 7.25 |
| LSM10 | Osteoporosis, Postmenopausal | 7.25 |
| LST1 | Osteoporosis, Postmenopausal | 7.25 |
| NAGS | Osteoporosis, Postmenopausal | 7.25 |
| NDUFB7 | Osteoporosis, Postmenopausal | 7.25 |
| NDUFB9 | Osteoporosis, Postmenopausal | 7.25 |
| NSMCE1 | Osteoporosis, Postmenopausal | 7.25 |
| NTPCR | Osteoporosis, Postmenopausal | 7.25 |
| PHYHD1 | Osteoporosis, Postmenopausal | 7.25 |
| PLCXD2 | Osteoporosis, Postmenopausal | 7.25 |
| RAB43 | Osteoporosis, Postmenopausal | 7.25 |
| RAC3 | Osteoporosis, Postmenopausal | 7.25 |
| RNF26 | Osteoporosis, Postmenopausal | 7.25 |
| SETD1B | Osteoporosis, Postmenopausal | 7.25 |
| SH3BGR | Osteoporosis, Postmenopausal | 7.25 |
| SLIRP | Osteoporosis, Postmenopausal | 7.25 |
| SMARCB1 | Osteoporosis, Postmenopausal | 7.25 |
| SPTLC3 | Osteoporosis, Postmenopausal | 7.25 |
| STAB2 | Osteoporosis, Postmenopausal | 7.25 |
| TET2 | Osteoporosis, Postmenopausal | 7.25 |
| TM6SF2 | Osteoporosis, Postmenopausal | 7.25 |
| TSR1 | Osteoporosis, Postmenopausal | 7.25 |
| UGT2B4 | Osteoporosis, Postmenopausal | 7.25 |
| WNT10A | Osteoporosis, Postmenopausal | 7.25 |
| XYLT1 | Osteoporosis, Postmenopausal | 7.25 |
| AP1S3 | Osteoporosis, Postmenopausal | 7.24 |
| DDX3Y | Osteoporosis, Postmenopausal | 7.24 |
| MUSTN1 | Osteoporosis, Postmenopausal | 7.24 |
| NELL1 | Osteoporosis, Postmenopausal | 7.24 |
| RAB18 | Osteoporosis, Postmenopausal | 7.24 |
| SBNO1 | Osteoporosis, Postmenopausal | 7.24 |
| ST3GAL2 | Osteoporosis, Postmenopausal | 7.24 |
| TMEM205 | Osteoporosis, Postmenopausal | 7.24 |
| ZBTB43 | Osteoporosis, Postmenopausal | 7.24 |
| AAMDC | Osteoporosis, Postmenopausal | 7.23 |
| AKIRIN1 | Osteoporosis, Postmenopausal | 7.23 |
| BEX1 | Osteoporosis, Postmenopausal | 7.23 |
| BSCL2 | Osteoporosis, Postmenopausal | 7.23 |
| C4BPA | Osteoporosis, Postmenopausal | 7.23 |
| CEP85L | Osteoporosis, Postmenopausal | 7.23 |
| ERG28 | Osteoporosis, Postmenopausal | 7.23 |
| FNIP2 | Osteoporosis, Postmenopausal | 7.23 |
| GAL3ST1 | Osteoporosis, Postmenopausal | 7.23 |
| GMFG | Osteoporosis, Postmenopausal | 7.23 |
| GSAP | Osteoporosis, Postmenopausal | 7.23 |
| HIPK1 | Osteoporosis, Postmenopausal | 7.23 |
| L3MBTL3 | Osteoporosis, Postmenopausal | 7.23 |
| LRRC40 | Osteoporosis, Postmenopausal | 7.23 |
| MAN1A1 | Osteoporosis, Postmenopausal | 7.23 |
| MAN1C1 | Osteoporosis, Postmenopausal | 7.23 |
| MAP3K3 | Osteoporosis, Postmenopausal | 7.23 |
| MGAT4A | Osteoporosis, Postmenopausal | 7.23 |
| NDUFB4 | Osteoporosis, Postmenopausal | 7.23 |
| NEGR1 | Osteoporosis, Postmenopausal | 7.23 |
| NELFE | Osteoporosis, Postmenopausal | 7.23 |
| NFXL1 | Osteoporosis, Postmenopausal | 7.23 |
| PARVB | Osteoporosis, Postmenopausal | 7.23 |
| PLSCR4 | Osteoporosis, Postmenopausal | 7.23 |
| PRRX2 | Osteoporosis, Postmenopausal | 7.23 |
| PTK7 | Osteoporosis, Postmenopausal | 7.23 |
| RALGPS2 | Osteoporosis, Postmenopausal | 7.23 |
| RPGR | Osteoporosis, Postmenopausal | 7.23 |
| RRN3 | Osteoporosis, Postmenopausal | 7.23 |
| SNHG11 | Osteoporosis, Postmenopausal | 7.23 |
| SPATA7 | Osteoporosis, Postmenopausal | 7.23 |
| ST7 | Osteoporosis, Postmenopausal | 7.23 |
| STAB1 | Osteoporosis, Postmenopausal | 7.23 |
| STIM1 | Osteoporosis, Postmenopausal | 7.23 |
| SYN2 | Osteoporosis, Postmenopausal | 7.23 |
| TCAF1 | Osteoporosis, Postmenopausal | 7.23 |
| TLCD4 | Osteoporosis, Postmenopausal | 7.23 |
| TMT1B | Osteoporosis, Postmenopausal | 7.23 |
| TNFSF13 | Osteoporosis, Postmenopausal | 7.23 |
| TPPP | Osteoporosis, Postmenopausal | 7.23 |
| UBE2G2 | Osteoporosis, Postmenopausal | 7.23 |
| UTP6 | Osteoporosis, Postmenopausal | 7.23 |
| ARL1 | Osteoporosis, Postmenopausal | 7.22 |
| ATAD5 | Osteoporosis, Postmenopausal | 7.22 |
| CD27 | Osteoporosis, Postmenopausal | 7.22 |
| CRYAA | Osteoporosis, Postmenopausal | 7.22 |
| DNAJC21 | Osteoporosis, Postmenopausal | 7.22 |
| FAM149A | Osteoporosis, Postmenopausal | 7.22 |
| GABRA5 | Osteoporosis, Postmenopausal | 7.22 |
| IRX1 | Osteoporosis, Postmenopausal | 7.22 |
| KLHL13 | Osteoporosis, Postmenopausal | 7.22 |
| MYOM2 | Osteoporosis, Postmenopausal | 7.22 |
| MYRIP | Osteoporosis, Postmenopausal | 7.22 |
| NAT8L | Osteoporosis, Postmenopausal | 7.22 |
| NDUFC2 | Osteoporosis, Postmenopausal | 7.22 |
| NOD1 | Osteoporosis, Postmenopausal | 7.22 |
| PALM | Osteoporosis, Postmenopausal | 7.22 |
| PGAM5 | Osteoporosis, Postmenopausal | 7.22 |
| PYY | Osteoporosis, Postmenopausal | 7.22 |
| TMC5 | Osteoporosis, Postmenopausal | 7.22 |
| VGLL3 | Osteoporosis, Postmenopausal | 7.22 |
| WNT6 | Osteoporosis, Postmenopausal | 7.22 |
| ADARB1 | Osteoporosis, Postmenopausal | 7.21 |
| ADCY2 | Osteoporosis, Postmenopausal | 7.21 |
| AQP11 | Osteoporosis, Postmenopausal | 7.21 |
| CCDC88C | Osteoporosis, Postmenopausal | 7.21 |
| CDC14A | Osteoporosis, Postmenopausal | 7.21 |
| CHIC2 | Osteoporosis, Postmenopausal | 7.21 |
| CLDN14 | Osteoporosis, Postmenopausal | 7.21 |
| DERL1 | Osteoporosis, Postmenopausal | 7.21 |
| DHRS1 | Osteoporosis, Postmenopausal | 7.21 |
| EDEM3 | Osteoporosis, Postmenopausal | 7.21 |
| EVI5 | Osteoporosis, Postmenopausal | 7.21 |
| FUCA2 | Osteoporosis, Postmenopausal | 7.21 |
| FUT4 | Osteoporosis, Postmenopausal | 7.21 |
| GALNT18 | Osteoporosis, Postmenopausal | 7.21 |
| GLRA1 | Osteoporosis, Postmenopausal | 7.21 |
| GOPC | Osteoporosis, Postmenopausal | 7.21 |
| IPCEF1 | Osteoporosis, Postmenopausal | 7.21 |
| KCNT1 | Osteoporosis, Postmenopausal | 7.21 |
| KLC4 | Osteoporosis, Postmenopausal | 7.21 |
| LRRC17 | Osteoporosis, Postmenopausal | 7.21 |
| MCUB | Osteoporosis, Postmenopausal | 7.21 |
| MRPL13 | Osteoporosis, Postmenopausal | 7.21 |
| MRPS17 | Osteoporosis, Postmenopausal | 7.21 |
| MTFP1 | Osteoporosis, Postmenopausal | 7.21 |
| OTUD4 | Osteoporosis, Postmenopausal | 7.21 |
| PAQR8 | Osteoporosis, Postmenopausal | 7.21 |
| PTGR2 | Osteoporosis, Postmenopausal | 7.21 |
| RIN1 | Osteoporosis, Postmenopausal | 7.21 |
| ROR2 | Osteoporosis, Postmenopausal | 7.21 |
| SCX | Osteoporosis, Postmenopausal | 7.21 |
| SEC22B | Osteoporosis, Postmenopausal | 7.21 |
| SEMA3G | Osteoporosis, Postmenopausal | 7.21 |
| SLC22A15 | Osteoporosis, Postmenopausal | 7.21 |
| STX2 | Osteoporosis, Postmenopausal | 7.21 |
| SYTL1 | Osteoporosis, Postmenopausal | 7.21 |
| TTC27 | Osteoporosis, Postmenopausal | 7.21 |
| UBL4A | Osteoporosis, Postmenopausal | 7.21 |
| UPF1 | Osteoporosis, Postmenopausal | 7.21 |
| ZC3H11A | Osteoporosis, Postmenopausal | 7.21 |
| CAPN3 | Osteoporosis, Postmenopausal | 7.2 |
| LYVE1 | Osteoporosis, Postmenopausal | 7.2 |
| MAPK6 | Osteoporosis, Postmenopausal | 7.2 |
| NRM | Osteoporosis, Postmenopausal | 7.2 |
| PDZK1IP1 | Osteoporosis, Postmenopausal | 7.2 |
| PLCD4 | Osteoporosis, Postmenopausal | 7.2 |
| RRAGC | Osteoporosis, Postmenopausal | 7.2 |
| TNFSF15 | Osteoporosis, Postmenopausal | 7.2 |
| ADD2 | Osteoporosis, Postmenopausal | 7.19 |
| ANKRA2 | Osteoporosis, Postmenopausal | 7.19 |
| ARHGAP15 | Osteoporosis, Postmenopausal | 7.19 |
| ARMC9 | Osteoporosis, Postmenopausal | 7.19 |
| ASB2 | Osteoporosis, Postmenopausal | 7.19 |
| BCAP31 | Osteoporosis, Postmenopausal | 7.19 |
| BTBD3 | Osteoporosis, Postmenopausal | 7.19 |
| BUB3 | Osteoporosis, Postmenopausal | 7.19 |
| CCDC50 | Osteoporosis, Postmenopausal | 7.19 |
| CCDC77 | Osteoporosis, Postmenopausal | 7.19 |
| CERS4 | Osteoporosis, Postmenopausal | 7.19 |
| COQ3 | Osteoporosis, Postmenopausal | 7.19 |
| CPED1 | Osteoporosis, Postmenopausal | 7.19 |
| CXCR3 | Osteoporosis, Postmenopausal | 7.19 |
| CYP2A13 | Osteoporosis, Postmenopausal | 7.19 |
| CYP3A13 | Osteoporosis, Postmenopausal | 7.19 |
| DGKZ | Osteoporosis, Postmenopausal | 7.19 |
| ESF1 | Osteoporosis, Postmenopausal | 7.19 |
| FTSJ3 | Osteoporosis, Postmenopausal | 7.19 |
| GRPEL2 | Osteoporosis, Postmenopausal | 7.19 |
| HCN2 | Osteoporosis, Postmenopausal | 7.19 |
| HOXB5 | Osteoporosis, Postmenopausal | 7.19 |
| ICE2 | Osteoporosis, Postmenopausal | 7.19 |
| IFT80 | Osteoporosis, Postmenopausal | 7.19 |
| KCNA2 | Osteoporosis, Postmenopausal | 7.19 |
| L2HGDH | Osteoporosis, Postmenopausal | 7.19 |
| LIMD2 | Osteoporosis, Postmenopausal | 7.19 |
| LZTFL1 | Osteoporosis, Postmenopausal | 7.19 |
| MAPK8IP1 | Osteoporosis, Postmenopausal | 7.19 |
| MARK2 | Osteoporosis, Postmenopausal | 7.19 |
| MATK | Osteoporosis, Postmenopausal | 7.19 |
| MGAT5 | Osteoporosis, Postmenopausal | 7.19 |
| NSMF | Osteoporosis, Postmenopausal | 7.19 |
| PRMT2 | Osteoporosis, Postmenopausal | 7.19 |
| PSMD5 | Osteoporosis, Postmenopausal | 7.19 |
| RALGAPA2 | Osteoporosis, Postmenopausal | 7.19 |
| RASAL1 | Osteoporosis, Postmenopausal | 7.19 |
| REEP4 | Osteoporosis, Postmenopausal | 7.19 |
| RSPO3 | Osteoporosis, Postmenopausal | 7.19 |
| SALL2 | Osteoporosis, Postmenopausal | 7.19 |
| SF3B3 | Osteoporosis, Postmenopausal | 7.19 |
| SHROOM3 | Osteoporosis, Postmenopausal | 7.19 |
| SLC52A2 | Osteoporosis, Postmenopausal | 7.19 |
| SNRNP25 | Osteoporosis, Postmenopausal | 7.19 |
| SPA17 | Osteoporosis, Postmenopausal | 7.19 |
| TCEA2 | Osteoporosis, Postmenopausal | 7.19 |
| TLK1 | Osteoporosis, Postmenopausal | 7.19 |
| TTPAL | Osteoporosis, Postmenopausal | 7.19 |
| UQCR10 | Osteoporosis, Postmenopausal | 7.19 |
| ZHX2 | Osteoporosis, Postmenopausal | 7.19 |
| RPL39 | Osteoporosis, Postmenopausal | 7.18 |
| ZCCHC24 | Osteoporosis, Postmenopausal | 7.18 |
| B4GALNT1 | Osteoporosis, Postmenopausal | 7.17 |
| C1GALT1C1 | Osteoporosis, Postmenopausal | 7.17 |
| C1QTNF3 | Osteoporosis, Postmenopausal | 7.17 |
| CEBPZ | Osteoporosis, Postmenopausal | 7.17 |
| CHST14 | Osteoporosis, Postmenopausal | 7.17 |
| CLUH | Osteoporosis, Postmenopausal | 7.17 |
| COPS2 | Osteoporosis, Postmenopausal | 7.17 |
| CORO2B | Osteoporosis, Postmenopausal | 7.17 |
| CXCR1 | Osteoporosis, Postmenopausal | 7.17 |
| FUT1 | Osteoporosis, Postmenopausal | 7.17 |
| GCA | Osteoporosis, Postmenopausal | 7.17 |
| GNG5 | Osteoporosis, Postmenopausal | 7.17 |
| KCNH2 | Osteoporosis, Postmenopausal | 7.17 |
| LDB3 | Osteoporosis, Postmenopausal | 7.17 |
| LY6A | Osteoporosis, Postmenopausal | 7.17 |
| MAS1 | Osteoporosis, Postmenopausal | 7.17 |
| MRPL23 | Osteoporosis, Postmenopausal | 7.17 |
| NETO2 | Osteoporosis, Postmenopausal | 7.17 |
| NOVA1 | Osteoporosis, Postmenopausal | 7.17 |
| PALS2 | Osteoporosis, Postmenopausal | 7.17 |
| POC1A | Osteoporosis, Postmenopausal | 7.17 |
| RPL38 | Osteoporosis, Postmenopausal | 7.17 |
| SLC35G1 | Osteoporosis, Postmenopausal | 7.17 |
| TCF25 | Osteoporosis, Postmenopausal | 7.17 |
| THEM6 | Osteoporosis, Postmenopausal | 7.17 |
| TMEM106A | Osteoporosis, Postmenopausal | 7.17 |
| ZC3H13 | Osteoporosis, Postmenopausal | 7.17 |
| ZCCHC2 | Osteoporosis, Postmenopausal | 7.17 |
| AMOTL1 | Osteoporosis, Postmenopausal | 7.16 |
| ARHGAP9 | Osteoporosis, Postmenopausal | 7.16 |
| BNIP2 | Osteoporosis, Postmenopausal | 7.16 |
| CDKL5 | Osteoporosis, Postmenopausal | 7.16 |
| EIF2B1 | Osteoporosis, Postmenopausal | 7.16 |
| FAIM2 | Osteoporosis, Postmenopausal | 7.16 |
| INTS7 | Osteoporosis, Postmenopausal | 7.16 |
| MAP4K5 | Osteoporosis, Postmenopausal | 7.16 |
| MRPL4 | Osteoporosis, Postmenopausal | 7.16 |
| NPDC1 | Osteoporosis, Postmenopausal | 7.16 |
| PAPLN | Osteoporosis, Postmenopausal | 7.16 |
| PCBP3 | Osteoporosis, Postmenopausal | 7.16 |
| PNRC2 | Osteoporosis, Postmenopausal | 7.16 |
| RABEP1 | Osteoporosis, Postmenopausal | 7.16 |
| RCOR1 | Osteoporosis, Postmenopausal | 7.16 |
| SH3RF3 | Osteoporosis, Postmenopausal | 7.16 |
| TEAD4 | Osteoporosis, Postmenopausal | 7.16 |
| TMEM87A | Osteoporosis, Postmenopausal | 7.16 |
| USPL1 | Osteoporosis, Postmenopausal | 7.16 |
| ZKSCAN1 | Osteoporosis, Postmenopausal | 7.16 |
| ACSL6 | Osteoporosis, Postmenopausal | 7.15 |
| ARHGEF6 | Osteoporosis, Postmenopausal | 7.15 |
| ART3 | Osteoporosis, Postmenopausal | 7.15 |
| CIAPIN1 | Osteoporosis, Postmenopausal | 7.15 |
| CIDEB | Osteoporosis, Postmenopausal | 7.15 |
| DGCR8 | Osteoporosis, Postmenopausal | 7.15 |
| DUSP7 | Osteoporosis, Postmenopausal | 7.15 |
| EME1 | Osteoporosis, Postmenopausal | 7.15 |
| EXTL1 | Osteoporosis, Postmenopausal | 7.15 |
| GPR176 | Osteoporosis, Postmenopausal | 7.15 |
| HAPLN1 | Osteoporosis, Postmenopausal | 7.15 |
| HEY2 | Osteoporosis, Postmenopausal | 7.15 |
| JPH1 | Osteoporosis, Postmenopausal | 7.15 |
| KAT14 | Osteoporosis, Postmenopausal | 7.15 |
| LDLRAD4 | Osteoporosis, Postmenopausal | 7.15 |
| LMO2 | Osteoporosis, Postmenopausal | 7.15 |
| LTBP3 | Osteoporosis, Postmenopausal | 7.15 |
| MCOLN2 | Osteoporosis, Postmenopausal | 7.15 |
| MSRB3 | Osteoporosis, Postmenopausal | 7.15 |
| SART1 | Osteoporosis, Postmenopausal | 7.15 |
| SLC35D1 | Osteoporosis, Postmenopausal | 7.15 |
| SNED1 | Osteoporosis, Postmenopausal | 7.15 |
| SNX9 | Osteoporosis, Postmenopausal | 7.15 |
| SRPX2 | Osteoporosis, Postmenopausal | 7.15 |
| ST6GALNAC2 | Osteoporosis, Postmenopausal | 7.15 |
| TMEM163 | Osteoporosis, Postmenopausal | 7.15 |
| TRAM2 | Osteoporosis, Postmenopausal | 7.15 |
| TRRAP | Osteoporosis, Postmenopausal | 7.15 |
| TSPAN33 | Osteoporosis, Postmenopausal | 7.15 |
| TUSC3 | Osteoporosis, Postmenopausal | 7.15 |
| TXNDC16 | Osteoporosis, Postmenopausal | 7.15 |
| ZAP70 | Osteoporosis, Postmenopausal | 7.15 |
| ZFYVE16 | Osteoporosis, Postmenopausal | 7.15 |
| AGFG2 | Osteoporosis, Postmenopausal | 7.14 |
| B4GALT2 | Osteoporosis, Postmenopausal | 7.14 |
| EIF3J | Osteoporosis, Postmenopausal | 7.14 |
| FNDC3A | Osteoporosis, Postmenopausal | 7.14 |
| GUCY1A2 | Osteoporosis, Postmenopausal | 7.14 |
| GYG1 | Osteoporosis, Postmenopausal | 7.14 |
| IFNGR2 | Osteoporosis, Postmenopausal | 7.14 |
| IGSF6 | Osteoporosis, Postmenopausal | 7.14 |
| KPNA1 | Osteoporosis, Postmenopausal | 7.14 |
| LSAMP | Osteoporosis, Postmenopausal | 7.14 |
| LSM3 | Osteoporosis, Postmenopausal | 7.14 |
| NANOS1 | Osteoporosis, Postmenopausal | 7.14 |
| NMRAL1 | Osteoporosis, Postmenopausal | 7.14 |
| NUDT12 | Osteoporosis, Postmenopausal | 7.14 |
| PLXDC1 | Osteoporosis, Postmenopausal | 7.14 |
| PUF60 | Osteoporosis, Postmenopausal | 7.14 |
| RND2 | Osteoporosis, Postmenopausal | 7.14 |
| SERPINA3K | Osteoporosis, Postmenopausal | 7.14 |
| TMF1 | Osteoporosis, Postmenopausal | 7.14 |
| USP47 | Osteoporosis, Postmenopausal | 7.14 |
| XPO4 | Osteoporosis, Postmenopausal | 7.14 |
| ATP6V1C1 | Osteoporosis, Postmenopausal | 7.13 |
| BCKDHA | Osteoporosis, Postmenopausal | 7.13 |
| C1GALT1 | Osteoporosis, Postmenopausal | 7.13 |
| CBX3 | Osteoporosis, Postmenopausal | 7.13 |
| CLEC2D | Osteoporosis, Postmenopausal | 7.13 |
| DEDD2 | Osteoporosis, Postmenopausal | 7.13 |
| DLG5 | Osteoporosis, Postmenopausal | 7.13 |
| EBF3 | Osteoporosis, Postmenopausal | 7.13 |
| EFEMP2 | Osteoporosis, Postmenopausal | 7.13 |
| EPB41L4B | Osteoporosis, Postmenopausal | 7.13 |
| FAM117B | Osteoporosis, Postmenopausal | 7.13 |
| FGF5 | Osteoporosis, Postmenopausal | 7.13 |
| KDM4B | Osteoporosis, Postmenopausal | 7.13 |
| KLHL42 | Osteoporosis, Postmenopausal | 7.13 |
| LEAP2 | Osteoporosis, Postmenopausal | 7.13 |
| MPC2 | Osteoporosis, Postmenopausal | 7.13 |
| PRICKLE2 | Osteoporosis, Postmenopausal | 7.13 |
| RAB4A | Osteoporosis, Postmenopausal | 7.13 |
| RBM19 | Osteoporosis, Postmenopausal | 7.13 |
| RFLNB | Osteoporosis, Postmenopausal | 7.13 |
| RGS20 | Osteoporosis, Postmenopausal | 7.13 |
| RNF14 | Osteoporosis, Postmenopausal | 7.13 |
| S1PR5 | Osteoporosis, Postmenopausal | 7.13 |
| SEC61B | Osteoporosis, Postmenopausal | 7.13 |
| SIRT6 | Osteoporosis, Postmenopausal | 7.13 |
| SLC12A1 | Osteoporosis, Postmenopausal | 7.13 |
| SLC44A3 | Osteoporosis, Postmenopausal | 7.13 |
| STUB1 | Osteoporosis, Postmenopausal | 7.13 |
| VAMP5 | Osteoporosis, Postmenopausal | 7.13 |
| WNK4 | Osteoporosis, Postmenopausal | 7.13 |
| ADAMTS12 | Osteoporosis, Postmenopausal | 7.12 |
| ANKFY1 | Osteoporosis, Postmenopausal | 7.12 |
| CENPI | Osteoporosis, Postmenopausal | 7.12 |
| CLCN4 | Osteoporosis, Postmenopausal | 7.12 |
| CLIP2 | Osteoporosis, Postmenopausal | 7.12 |
| CSNK1G1 | Osteoporosis, Postmenopausal | 7.12 |
| CYP2D22 | Osteoporosis, Postmenopausal | 7.12 |
| DEGS1 | Osteoporosis, Postmenopausal | 7.12 |
| FNBP1L | Osteoporosis, Postmenopausal | 7.12 |
| HSD3B5 | Osteoporosis, Postmenopausal | 7.12 |
| MAFA | Osteoporosis, Postmenopausal | 7.12 |
| MAP3K2 | Osteoporosis, Postmenopausal | 7.12 |
| MARS1 | Osteoporosis, Postmenopausal | 7.12 |
| MYO1C | Osteoporosis, Postmenopausal | 7.12 |
| PPTC7 | Osteoporosis, Postmenopausal | 7.12 |
| REEP2 | Osteoporosis, Postmenopausal | 7.12 |
| RSL24D1 | Osteoporosis, Postmenopausal | 7.12 |
| RUFY3 | Osteoporosis, Postmenopausal | 7.12 |
| SAMM50 | Osteoporosis, Postmenopausal | 7.12 |
| SAT2 | Osteoporosis, Postmenopausal | 7.12 |
| SPSB4 | Osteoporosis, Postmenopausal | 7.12 |
| SYTL5 | Osteoporosis, Postmenopausal | 7.12 |
| TFB1M | Osteoporosis, Postmenopausal | 7.12 |
| TMEM106C | Osteoporosis, Postmenopausal | 7.12 |
| UFM1 | Osteoporosis, Postmenopausal | 7.12 |
| UPF3B | Osteoporosis, Postmenopausal | 7.12 |
| VIP | Osteoporosis, Postmenopausal | 7.12 |
| ARHGEF19 | Osteoporosis, Postmenopausal | 7.11 |
| ATP2C1 | Osteoporosis, Postmenopausal | 7.11 |
| BHLHA15 | Osteoporosis, Postmenopausal | 7.11 |
| BICD1 | Osteoporosis, Postmenopausal | 7.11 |
| CCDC68 | Osteoporosis, Postmenopausal | 7.11 |
| CLK3 | Osteoporosis, Postmenopausal | 7.11 |
| CSPP1 | Osteoporosis, Postmenopausal | 7.11 |
| CUBN | Osteoporosis, Postmenopausal | 7.11 |
| DAPK2 | Osteoporosis, Postmenopausal | 7.11 |
| FCGBP | Osteoporosis, Postmenopausal | 7.11 |
| GRIP1 | Osteoporosis, Postmenopausal | 7.11 |
| MAP4K1 | Osteoporosis, Postmenopausal | 7.11 |
| MLC1 | Osteoporosis, Postmenopausal | 7.11 |
| NLRC5 | Osteoporosis, Postmenopausal | 7.11 |
| OAS1A | Osteoporosis, Postmenopausal | 7.11 |
| OBSCN | Osteoporosis, Postmenopausal | 7.11 |
| PHACTR1 | Osteoporosis, Postmenopausal | 7.11 |
| POPDC2 | Osteoporosis, Postmenopausal | 7.11 |
| PROSER2 | Osteoporosis, Postmenopausal | 7.11 |
| RAB40B | Osteoporosis, Postmenopausal | 7.11 |
| RBM12 | Osteoporosis, Postmenopausal | 7.11 |
| RIMS3 | Osteoporosis, Postmenopausal | 7.11 |
| RNF145 | Osteoporosis, Postmenopausal | 7.11 |
| RNF19A | Osteoporosis, Postmenopausal | 7.11 |
| SAPCD2 | Osteoporosis, Postmenopausal | 7.11 |
| SH2D4A | Osteoporosis, Postmenopausal | 7.11 |
| SLC16A14 | Osteoporosis, Postmenopausal | 7.11 |
| STAG1 | Osteoporosis, Postmenopausal | 7.11 |
| THEMIS2 | Osteoporosis, Postmenopausal | 7.11 |
| TMEM45A | Osteoporosis, Postmenopausal | 7.11 |
| TNFA | Osteoporosis, Postmenopausal | 7.11 |
| ARPC4 | Osteoporosis, Postmenopausal | 7.1 |
| BOC | Osteoporosis, Postmenopausal | 7.1 |
| BRWD1 | Osteoporosis, Postmenopausal | 7.1 |
| BYSL | Osteoporosis, Postmenopausal | 7.1 |
| EIF2B3 | Osteoporosis, Postmenopausal | 7.1 |
| EPS15 | Osteoporosis, Postmenopausal | 7.1 |
| FNDC4 | Osteoporosis, Postmenopausal | 7.1 |
| H1-10 | Osteoporosis, Postmenopausal | 7.1 |
| HAX1 | Osteoporosis, Postmenopausal | 7.1 |
| HLTF | Osteoporosis, Postmenopausal | 7.1 |
| IDH3B | Osteoporosis, Postmenopausal | 7.1 |
| MLLT6 | Osteoporosis, Postmenopausal | 7.1 |
| NDUFB3 | Osteoporosis, Postmenopausal | 7.1 |
| PLXND1 | Osteoporosis, Postmenopausal | 7.1 |
| POLG2 | Osteoporosis, Postmenopausal | 7.1 |
| PRMT7 | Osteoporosis, Postmenopausal | 7.1 |
| PUM3 | Osteoporosis, Postmenopausal | 7.1 |
| RASD2 | Osteoporosis, Postmenopausal | 7.1 |
| RXRB | Osteoporosis, Postmenopausal | 7.1 |
| SEC61G | Osteoporosis, Postmenopausal | 7.1 |
| SEMA5B | Osteoporosis, Postmenopausal | 7.1 |
| SETD7 | Osteoporosis, Postmenopausal | 7.1 |
| SNRPD3 | Osteoporosis, Postmenopausal | 7.1 |
| STRN | Osteoporosis, Postmenopausal | 7.1 |
| TNKS2 | Osteoporosis, Postmenopausal | 7.1 |
| TRMT6 | Osteoporosis, Postmenopausal | 7.1 |
| TRPC3 | Osteoporosis, Postmenopausal | 7.1 |
| ACBD4 | Osteoporosis, Postmenopausal | 7.09 |
| ANKHD1 | Osteoporosis, Postmenopausal | 7.09 |
| BNC2 | Osteoporosis, Postmenopausal | 7.09 |
| CAPNS1 | Osteoporosis, Postmenopausal | 7.09 |
| CCDC34 | Osteoporosis, Postmenopausal | 7.09 |
| CHN1 | Osteoporosis, Postmenopausal | 7.09 |
| DNAJB5 | Osteoporosis, Postmenopausal | 7.09 |
| DOCK11 | Osteoporosis, Postmenopausal | 7.09 |
| EPHB6 | Osteoporosis, Postmenopausal | 7.09 |
| ERMP1 | Osteoporosis, Postmenopausal | 7.09 |
| FGD3 | Osteoporosis, Postmenopausal | 7.09 |
| GEMIN4 | Osteoporosis, Postmenopausal | 7.09 |
| HMGN5 | Osteoporosis, Postmenopausal | 7.09 |
| KRT13 | Osteoporosis, Postmenopausal | 7.09 |
| LARP6 | Osteoporosis, Postmenopausal | 7.09 |
| LENG8 | Osteoporosis, Postmenopausal | 7.09 |
| MOGAT1 | Osteoporosis, Postmenopausal | 7.09 |
| NIT2 | Osteoporosis, Postmenopausal | 7.09 |
| NOL6 | Osteoporosis, Postmenopausal | 7.09 |
| PBX3 | Osteoporosis, Postmenopausal | 7.09 |
| PEX2 | Osteoporosis, Postmenopausal | 7.09 |
| PLEKHO1 | Osteoporosis, Postmenopausal | 7.09 |
| SASH1 | Osteoporosis, Postmenopausal | 7.09 |
| SERPINA10 | Osteoporosis, Postmenopausal | 7.09 |
| SLC45A4 | Osteoporosis, Postmenopausal | 7.09 |
| SOAT2 | Osteoporosis, Postmenopausal | 7.09 |
| SPTB | Osteoporosis, Postmenopausal | 7.09 |
| TAOK1 | Osteoporosis, Postmenopausal | 7.09 |
| TICRR | Osteoporosis, Postmenopausal | 7.09 |
| TNFRSF8 | Osteoporosis, Postmenopausal | 7.09 |
| TRANK1 | Osteoporosis, Postmenopausal | 7.09 |
| TTC39A | Osteoporosis, Postmenopausal | 7.09 |
| ADSL | Osteoporosis, Postmenopausal | 7.08 |
| ANKRD44 | Osteoporosis, Postmenopausal | 7.08 |
| ARID5A | Osteoporosis, Postmenopausal | 7.08 |
| ARPC5L | Osteoporosis, Postmenopausal | 7.08 |
| BET1 | Osteoporosis, Postmenopausal | 7.08 |
| CCDC88A | Osteoporosis, Postmenopausal | 7.08 |
| CDC27 | Osteoporosis, Postmenopausal | 7.08 |
| CDKAL1 | Osteoporosis, Postmenopausal | 7.08 |
| CEP85 | Osteoporosis, Postmenopausal | 7.08 |
| CKMT2 | Osteoporosis, Postmenopausal | 7.08 |
| CTBS | Osteoporosis, Postmenopausal | 7.08 |
| CTRB1 | Osteoporosis, Postmenopausal | 7.08 |
| ERC2 | Osteoporosis, Postmenopausal | 7.08 |
| GIGYF2 | Osteoporosis, Postmenopausal | 7.08 |
| GMPPB | Osteoporosis, Postmenopausal | 7.08 |
| GNL3L | Osteoporosis, Postmenopausal | 7.08 |
| HECW2 | Osteoporosis, Postmenopausal | 7.08 |
| KRT80 | Osteoporosis, Postmenopausal | 7.08 |
| LUC7L2 | Osteoporosis, Postmenopausal | 7.08 |
| N4BP2 | Osteoporosis, Postmenopausal | 7.08 |
| NNAT | Osteoporosis, Postmenopausal | 7.08 |
| NSD3 | Osteoporosis, Postmenopausal | 7.08 |
| NT5C2 | Osteoporosis, Postmenopausal | 7.08 |
| PDXP | Osteoporosis, Postmenopausal | 7.08 |
| PELP1 | Osteoporosis, Postmenopausal | 7.08 |
| RAB5C | Osteoporosis, Postmenopausal | 7.08 |
| RPRM | Osteoporosis, Postmenopausal | 7.08 |
| RSF1 | Osteoporosis, Postmenopausal | 7.08 |
| SEZ6 | Osteoporosis, Postmenopausal | 7.08 |
| SIRT5 | Osteoporosis, Postmenopausal | 7.08 |
| SYNGR1 | Osteoporosis, Postmenopausal | 7.08 |
| TIMM10B | Osteoporosis, Postmenopausal | 7.08 |
| TIMM13 | Osteoporosis, Postmenopausal | 7.08 |
| TRAF5 | Osteoporosis, Postmenopausal | 7.08 |
| TRAIP | Osteoporosis, Postmenopausal | 7.08 |
| BAP1 | Osteoporosis, Postmenopausal | 7.07 |
| FBXO31 | Osteoporosis, Postmenopausal | 7.07 |
| FZD6 | Osteoporosis, Postmenopausal | 7.07 |
| ITPK1 | Osteoporosis, Postmenopausal | 7.07 |
| KBTBD8 | Osteoporosis, Postmenopausal | 7.07 |
| KLHL29 | Osteoporosis, Postmenopausal | 7.07 |
| MYO7A | Osteoporosis, Postmenopausal | 7.07 |
| NAPEPLD | Osteoporosis, Postmenopausal | 7.07 |
| NRTN | Osteoporosis, Postmenopausal | 7.07 |
| PLCB3 | Osteoporosis, Postmenopausal | 7.07 |
| PLEKHG3 | Osteoporosis, Postmenopausal | 7.07 |
| POLQ | Osteoporosis, Postmenopausal | 7.07 |
| RAPGEF5 | Osteoporosis, Postmenopausal | 7.07 |
| RASGEF1B | Osteoporosis, Postmenopausal | 7.07 |
| RGS19 | Osteoporosis, Postmenopausal | 7.07 |
| SCN1A | Osteoporosis, Postmenopausal | 7.07 |
| SCN3B | Osteoporosis, Postmenopausal | 7.07 |
| SGPP1 | Osteoporosis, Postmenopausal | 7.07 |
| SH3D19 | Osteoporosis, Postmenopausal | 7.07 |
| SLC17A6 | Osteoporosis, Postmenopausal | 7.07 |
| SMARCA5 | Osteoporosis, Postmenopausal | 7.07 |
| SPOCK1 | Osteoporosis, Postmenopausal | 7.07 |
| UBASH3B | Osteoporosis, Postmenopausal | 7.07 |
| WDR4 | Osteoporosis, Postmenopausal | 7.07 |
| ATL3 | Osteoporosis, Postmenopausal | 7.06 |
| CARD11 | Osteoporosis, Postmenopausal | 7.06 |
| CCNT2 | Osteoporosis, Postmenopausal | 7.06 |
| CLEC10A | Osteoporosis, Postmenopausal | 7.06 |
| CMTM3 | Osteoporosis, Postmenopausal | 7.06 |
| CPZ | Osteoporosis, Postmenopausal | 7.06 |
| CSDE1 | Osteoporosis, Postmenopausal | 7.06 |
| CYP46A1 | Osteoporosis, Postmenopausal | 7.06 |
| EEIG1 | Osteoporosis, Postmenopausal | 7.06 |
| ELAC2 | Osteoporosis, Postmenopausal | 7.06 |
| FITM2 | Osteoporosis, Postmenopausal | 7.06 |
| FOLR2 | Osteoporosis, Postmenopausal | 7.06 |
| FOXJ1 | Osteoporosis, Postmenopausal | 7.06 |
| FOXP4 | Osteoporosis, Postmenopausal | 7.06 |
| GJB3 | Osteoporosis, Postmenopausal | 7.06 |
| GSTM1 | Osteoporosis, Postmenopausal | 7.06 |
| IL10RB | Osteoporosis, Postmenopausal | 7.06 |
| MON2 | Osteoporosis, Postmenopausal | 7.06 |
| MXD3 | Osteoporosis, Postmenopausal | 7.06 |
| PDE1C | Osteoporosis, Postmenopausal | 7.06 |
| PPP3CB | Osteoporosis, Postmenopausal | 7.06 |
| SOSTDC1 | Osteoporosis, Postmenopausal | 7.06 |
| TRIP12 | Osteoporosis, Postmenopausal | 7.06 |
| TTLL12 | Osteoporosis, Postmenopausal | 7.06 |
| WDR75 | Osteoporosis, Postmenopausal | 7.06 |
| ASAP2 | Osteoporosis, Postmenopausal | 7.05 |
| DNAJA4 | Osteoporosis, Postmenopausal | 7.05 |
| ELF4 | Osteoporosis, Postmenopausal | 7.05 |
| GPLD1 | Osteoporosis, Postmenopausal | 7.05 |
| KRCC1 | Osteoporosis, Postmenopausal | 7.05 |
| LYSMD2 | Osteoporosis, Postmenopausal | 7.05 |
| MAP3K12 | Osteoporosis, Postmenopausal | 7.05 |
| PEX19 | Osteoporosis, Postmenopausal | 7.05 |
| PGAP1 | Osteoporosis, Postmenopausal | 7.05 |
| SLC16A4 | Osteoporosis, Postmenopausal | 7.05 |
| STEAP2 | Osteoporosis, Postmenopausal | 7.05 |
| ZC3HAV1 | Osteoporosis, Postmenopausal | 7.05 |
| ACMSD | Osteoporosis, Postmenopausal | 7.04 |
| AGFG1 | Osteoporosis, Postmenopausal | 7.04 |
| AOX3 | Osteoporosis, Postmenopausal | 7.04 |
| ARSG | Osteoporosis, Postmenopausal | 7.04 |
| ASTN2 | Osteoporosis, Postmenopausal | 7.04 |
| ATAD3A | Osteoporosis, Postmenopausal | 7.04 |
| CLCN5 | Osteoporosis, Postmenopausal | 7.04 |
| COL4A5 | Osteoporosis, Postmenopausal | 7.04 |
| DENND2D | Osteoporosis, Postmenopausal | 7.04 |
| EIF2AK4 | Osteoporosis, Postmenopausal | 7.04 |
| FURIN | Osteoporosis, Postmenopausal | 7.04 |
| GALNT7 | Osteoporosis, Postmenopausal | 7.04 |
| HLCS | Osteoporosis, Postmenopausal | 7.04 |
| KCNJ12 | Osteoporosis, Postmenopausal | 7.04 |
| KPNA4 | Osteoporosis, Postmenopausal | 7.04 |
| LTN1 | Osteoporosis, Postmenopausal | 7.04 |
| MARCHF6 | Osteoporosis, Postmenopausal | 7.04 |
| MCCC1 | Osteoporosis, Postmenopausal | 7.04 |
| MIOX | Osteoporosis, Postmenopausal | 7.04 |
| MPEG1 | Osteoporosis, Postmenopausal | 7.04 |
| NDUFB5 | Osteoporosis, Postmenopausal | 7.04 |
| NMT1 | Osteoporosis, Postmenopausal | 7.04 |
| NOP16 | Osteoporosis, Postmenopausal | 7.04 |
| NRAMP-ALPHA | Osteoporosis, Postmenopausal | 7.04 |
| NUCKS1 | Osteoporosis, Postmenopausal | 7.04 |
| PITPNB | Osteoporosis, Postmenopausal | 7.04 |
| PLEKHO2 | Osteoporosis, Postmenopausal | 7.04 |
| PPP2R2C | Osteoporosis, Postmenopausal | 7.04 |
| RAB11A | Osteoporosis, Postmenopausal | 7.04 |
| RRP9 | Osteoporosis, Postmenopausal | 7.04 |
| RTN3 | Osteoporosis, Postmenopausal | 7.04 |
| SLC26A1 | Osteoporosis, Postmenopausal | 7.04 |
| SPI1 | Osteoporosis, Postmenopausal | 7.04 |
| STRN3 | Osteoporosis, Postmenopausal | 7.04 |
| TNFSF13B | Osteoporosis, Postmenopausal | 7.04 |
| TRMT1 | Osteoporosis, Postmenopausal | 7.04 |
| UPF2 | Osteoporosis, Postmenopausal | 7.04 |
| ZPR1 | Osteoporosis, Postmenopausal | 7.04 |
| CDK14 | Osteoporosis, Postmenopausal | 7.03 |
| DNTTIP2 | Osteoporosis, Postmenopausal | 7.03 |
| E2F5 | Osteoporosis, Postmenopausal | 7.03 |
| EMG1 | Osteoporosis, Postmenopausal | 7.03 |
| GMDS | Osteoporosis, Postmenopausal | 7.03 |
| HMGN1 | Osteoporosis, Postmenopausal | 7.03 |
| N4BP2L2 | Osteoporosis, Postmenopausal | 7.03 |
| ND2 | Osteoporosis, Postmenopausal | 7.03 |
| PRPF8 | Osteoporosis, Postmenopausal | 7.03 |
| SLC38A5 | Osteoporosis, Postmenopausal | 7.03 |
| SPATA13 | Osteoporosis, Postmenopausal | 7.03 |
| STK38 | Osteoporosis, Postmenopausal | 7.03 |
| TCEAL8 | Osteoporosis, Postmenopausal | 7.03 |
| ATP13A3 | Osteoporosis, Postmenopausal | 7.02 |
| ATP1B2 | Osteoporosis, Postmenopausal | 7.02 |
| ATP5MC3 | Osteoporosis, Postmenopausal | 7.02 |
| CASZ1 | Osteoporosis, Postmenopausal | 7.02 |
| CETN3 | Osteoporosis, Postmenopausal | 7.02 |
| CPA4 | Osteoporosis, Postmenopausal | 7.02 |
| DHX30 | Osteoporosis, Postmenopausal | 7.02 |
| FEZ2 | Osteoporosis, Postmenopausal | 7.02 |
| HDAC8 | Osteoporosis, Postmenopausal | 7.02 |
| KBTBD11 | Osteoporosis, Postmenopausal | 7.02 |
| LCOR | Osteoporosis, Postmenopausal | 7.02 |
| MAN2B1 | Osteoporosis, Postmenopausal | 7.02 |
| MARVELD1 | Osteoporosis, Postmenopausal | 7.02 |
| MCFD2 | Osteoporosis, Postmenopausal | 7.02 |
| MCM8 | Osteoporosis, Postmenopausal | 7.02 |
| NEO1 | Osteoporosis, Postmenopausal | 7.02 |
| OLIG2 | Osteoporosis, Postmenopausal | 7.02 |
| OPRL1 | Osteoporosis, Postmenopausal | 7.02 |
| PCDH1 | Osteoporosis, Postmenopausal | 7.02 |
| PLLP | Osteoporosis, Postmenopausal | 7.02 |
| PPP1R16B | Osteoporosis, Postmenopausal | 7.02 |
| PRXL2B | Osteoporosis, Postmenopausal | 7.02 |
| PTPRB | Osteoporosis, Postmenopausal | 7.02 |
| RHO | Osteoporosis, Postmenopausal | 7.02 |
| RPIA | Osteoporosis, Postmenopausal | 7.02 |
| SALL4 | Osteoporosis, Postmenopausal | 7.02 |
| SCIN | Osteoporosis, Postmenopausal | 7.02 |
| SLC16A5 | Osteoporosis, Postmenopausal | 7.02 |
| SLC6A1 | Osteoporosis, Postmenopausal | 7.02 |
| SNRPA1 | Osteoporosis, Postmenopausal | 7.02 |
| SRSF4 | Osteoporosis, Postmenopausal | 7.02 |
| TBC1D5 | Osteoporosis, Postmenopausal | 7.02 |
| CDC42EP3 | Osteoporosis, Postmenopausal | 7.01 |
| DEPDC7 | Osteoporosis, Postmenopausal | 7.01 |
| GTF2B | Osteoporosis, Postmenopausal | 7.01 |
| H4C3 | Osteoporosis, Postmenopausal | 7.01 |
| KCNK2 | Osteoporosis, Postmenopausal | 7.01 |
| MFHAS1 | Osteoporosis, Postmenopausal | 7.01 |
| NIN | Osteoporosis, Postmenopausal | 7.01 |
| NUDC | Osteoporosis, Postmenopausal | 7.01 |
| POLH | Osteoporosis, Postmenopausal | 7.01 |
| PSCA | Osteoporosis, Postmenopausal | 7.01 |
| SLC29A3 | Osteoporosis, Postmenopausal | 7.01 |
| TINF2 | Osteoporosis, Postmenopausal | 7.01 |
| TRMT61A | Osteoporosis, Postmenopausal | 7.01 |
| ACBD5 | Osteoporosis, Postmenopausal | 7 |
| AHCTF1 | Osteoporosis, Postmenopausal | 7 |
| AKNA | Osteoporosis, Postmenopausal | 7 |
| APCDD1 | Osteoporosis, Postmenopausal | 7 |
| ATP5PB | Osteoporosis, Postmenopausal | 7 |
| B3GNT2 | Osteoporosis, Postmenopausal | 7 |
| CHD1 | Osteoporosis, Postmenopausal | 7 |
| EPHA7 | Osteoporosis, Postmenopausal | 7 |
| HKDC1 | Osteoporosis, Postmenopausal | 7 |
| IL27RA | Osteoporosis, Postmenopausal | 7 |
| INPP1 | Osteoporosis, Postmenopausal | 7 |
| KCNE3 | Osteoporosis, Postmenopausal | 7 |
| LATS2 | Osteoporosis, Postmenopausal | 7 |
| MBD3 | Osteoporosis, Postmenopausal | 7 |
| NIPBL | Osteoporosis, Postmenopausal | 7 |
| NTHL1 | Osteoporosis, Postmenopausal | 7 |
| NXPH3 | Osteoporosis, Postmenopausal | 7 |
| PIM2 | Osteoporosis, Postmenopausal | 7 |
| RHPN2 | Osteoporosis, Postmenopausal | 7 |
| RIPK4 | Osteoporosis, Postmenopausal | 7 |
| RPTOR | Osteoporosis, Postmenopausal | 7 |
| SCN1B | Osteoporosis, Postmenopausal | 7 |
| SEC14L2 | Osteoporosis, Postmenopausal | 7 |
| SLC16A11 | Osteoporosis, Postmenopausal | 7 |
| SLC26A6 | Osteoporosis, Postmenopausal | 7 |
| SNRPB | Osteoporosis, Postmenopausal | 7 |
| SPNS2 | Osteoporosis, Postmenopausal | 7 |
| SSPN | Osteoporosis, Postmenopausal | 7 |
| TENM2 | Osteoporosis, Postmenopausal | 7 |
| TMX1 | Osteoporosis, Postmenopausal | 7 |
| TRERF1 | Osteoporosis, Postmenopausal | 7 |
| WNT2 | Osteoporosis, Postmenopausal | 7 |
| ADAMTSL4 | Osteoporosis, Postmenopausal | 6.99 |
| ARFGAP3 | Osteoporosis, Postmenopausal | 6.99 |
| ASPN | Osteoporosis, Postmenopausal | 6.99 |
| ATP8A1 | Osteoporosis, Postmenopausal | 6.99 |
| DMGDH | Osteoporosis, Postmenopausal | 6.99 |
| FLVCR2 | Osteoporosis, Postmenopausal | 6.99 |
| GSE1 | Osteoporosis, Postmenopausal | 6.99 |
| GYPC | Osteoporosis, Postmenopausal | 6.99 |
| HLA-DRB1 | Osteoporosis, Postmenopausal | 6.99 |
| KMT2D | Osteoporosis, Postmenopausal | 6.99 |
| LIMS1 | Osteoporosis, Postmenopausal | 6.99 |
| MLLT3 | Osteoporosis, Postmenopausal | 6.99 |
| NACA | Osteoporosis, Postmenopausal | 6.99 |
| PDZRN3 | Osteoporosis, Postmenopausal | 6.99 |
| PIEZO2 | Osteoporosis, Postmenopausal | 6.99 |
| SH3RF1 | Osteoporosis, Postmenopausal | 6.99 |
| SLC25A24 | Osteoporosis, Postmenopausal | 6.99 |
| SRRM1 | Osteoporosis, Postmenopausal | 6.99 |
| TRIM22 | Osteoporosis, Postmenopausal | 6.99 |
| ADH1B | Osteoporosis, Postmenopausal | 6.98 |
| ADRA1D | Osteoporosis, Postmenopausal | 6.98 |
| AGPAT4 | Osteoporosis, Postmenopausal | 6.98 |
| AP3D1 | Osteoporosis, Postmenopausal | 6.98 |
| C1QTNF1 | Osteoporosis, Postmenopausal | 6.98 |
| CACNB2 | Osteoporosis, Postmenopausal | 6.98 |
| COPB1 | Osteoporosis, Postmenopausal | 6.98 |
| DIAPH2 | Osteoporosis, Postmenopausal | 6.98 |
| DPP9 | Osteoporosis, Postmenopausal | 6.98 |
| ITIH2 | Osteoporosis, Postmenopausal | 6.98 |
| LIMK2 | Osteoporosis, Postmenopausal | 6.98 |
| NHERF2 | Osteoporosis, Postmenopausal | 6.98 |
| NR2F6 | Osteoporosis, Postmenopausal | 6.98 |
| PRKX | Osteoporosis, Postmenopausal | 6.98 |
| RPL19 | Osteoporosis, Postmenopausal | 6.98 |
| SDCBP2 | Osteoporosis, Postmenopausal | 6.98 |
| SFXN3 | Osteoporosis, Postmenopausal | 6.98 |
| TBC1D31 | Osteoporosis, Postmenopausal | 6.98 |
| CAMK2G | Osteoporosis, Postmenopausal | 6.97 |
| CNTRL | Osteoporosis, Postmenopausal | 6.97 |
| ECHDC1 | Osteoporosis, Postmenopausal | 6.97 |
| FHOD3 | Osteoporosis, Postmenopausal | 6.97 |
| GPR146 | Osteoporosis, Postmenopausal | 6.97 |
| HEG1 | Osteoporosis, Postmenopausal | 6.97 |
| MBNL3 | Osteoporosis, Postmenopausal | 6.97 |
| MMAB | Osteoporosis, Postmenopausal | 6.97 |
| PRKAR1B | Osteoporosis, Postmenopausal | 6.97 |
| PTGES3 | Osteoporosis, Postmenopausal | 6.97 |
| SAP18 | Osteoporosis, Postmenopausal | 6.97 |
| SLC31A2 | Osteoporosis, Postmenopausal | 6.97 |
| TBC1D2 | Osteoporosis, Postmenopausal | 6.97 |
| TOX3 | Osteoporosis, Postmenopausal | 6.97 |
| WDR12 | Osteoporosis, Postmenopausal | 6.97 |
| ARL5B | Osteoporosis, Postmenopausal | 6.96 |
| ATP6V0E2 | Osteoporosis, Postmenopausal | 6.96 |
| BATF3 | Osteoporosis, Postmenopausal | 6.96 |
| BRI3BP | Osteoporosis, Postmenopausal | 6.96 |
| CORO6 | Osteoporosis, Postmenopausal | 6.96 |
| COX7A1 | Osteoporosis, Postmenopausal | 6.96 |
| ERC1 | Osteoporosis, Postmenopausal | 6.96 |
| GALK1 | Osteoporosis, Postmenopausal | 6.96 |
| GMPR | Osteoporosis, Postmenopausal | 6.96 |
| GOLIM4 | Osteoporosis, Postmenopausal | 6.96 |
| HERC3 | Osteoporosis, Postmenopausal | 6.96 |
| HSD17B8 | Osteoporosis, Postmenopausal | 6.96 |
| IMPACT | Osteoporosis, Postmenopausal | 6.96 |
| ITPA | Osteoporosis, Postmenopausal | 6.96 |
| KANK2 | Osteoporosis, Postmenopausal | 6.96 |
| LRRC8D | Osteoporosis, Postmenopausal | 6.96 |
| LTC4S | Osteoporosis, Postmenopausal | 6.96 |
| MAGOH | Osteoporosis, Postmenopausal | 6.96 |
| MYL12A | Osteoporosis, Postmenopausal | 6.96 |
| NSUN2 | Osteoporosis, Postmenopausal | 6.96 |
| PCDH9 | Osteoporosis, Postmenopausal | 6.96 |
| PDHX | Osteoporosis, Postmenopausal | 6.96 |
| QPRT | Osteoporosis, Postmenopausal | 6.96 |
| RICTOR | Osteoporosis, Postmenopausal | 6.96 |
| RYBP | Osteoporosis, Postmenopausal | 6.96 |
| SH3BP2 | Osteoporosis, Postmenopausal | 6.96 |
| SMPDL3B | Osteoporosis, Postmenopausal | 6.96 |
| SPATS2L | Osteoporosis, Postmenopausal | 6.96 |
| STK38L | Osteoporosis, Postmenopausal | 6.96 |
| TC2N | Osteoporosis, Postmenopausal | 6.96 |
| TMCC1 | Osteoporosis, Postmenopausal | 6.96 |
| VPS13D | Osteoporosis, Postmenopausal | 6.96 |
| YRDC | Osteoporosis, Postmenopausal | 6.96 |
| AFF1 | Osteoporosis, Postmenopausal | 6.95 |
| CELSR1 | Osteoporosis, Postmenopausal | 6.95 |
| CHRNA2 | Osteoporosis, Postmenopausal | 6.95 |
| CHST11 | Osteoporosis, Postmenopausal | 6.95 |
| CHSY1 | Osteoporosis, Postmenopausal | 6.95 |
| DROSHA | Osteoporosis, Postmenopausal | 6.95 |
| GNB2 | Osteoporosis, Postmenopausal | 6.95 |
| IL2RG | Osteoporosis, Postmenopausal | 6.95 |
| KCND2 | Osteoporosis, Postmenopausal | 6.95 |
| KCNJ3 | Osteoporosis, Postmenopausal | 6.95 |
| MACROD1 | Osteoporosis, Postmenopausal | 6.95 |
| MIR122 | Osteoporosis, Postmenopausal | 6.95 |
| MUC5B | Osteoporosis, Postmenopausal | 6.95 |
| PDLIM3 | Osteoporosis, Postmenopausal | 6.95 |
| PLIN5 | Osteoporosis, Postmenopausal | 6.95 |
| SHISA2 | Osteoporosis, Postmenopausal | 6.95 |
| STXBP6 | Osteoporosis, Postmenopausal | 6.95 |
| SVEP1 | Osteoporosis, Postmenopausal | 6.95 |
| TMTC2 | Osteoporosis, Postmenopausal | 6.95 |
| ACOT9 | Osteoporosis, Postmenopausal | 6.94 |
| AIMP2 | Osteoporosis, Postmenopausal | 6.94 |
| B3GALNT1 | Osteoporosis, Postmenopausal | 6.94 |
| BCL6B | Osteoporosis, Postmenopausal | 6.94 |
| BEX3 | Osteoporosis, Postmenopausal | 6.94 |
| BORA | Osteoporosis, Postmenopausal | 6.94 |
| CPM | Osteoporosis, Postmenopausal | 6.94 |
| DDHD1 | Osteoporosis, Postmenopausal | 6.94 |
| DDX10 | Osteoporosis, Postmenopausal | 6.94 |
| DERA | Osteoporosis, Postmenopausal | 6.94 |
| EZH1 | Osteoporosis, Postmenopausal | 6.94 |
| FASTK | Osteoporosis, Postmenopausal | 6.94 |
| FSTL3 | Osteoporosis, Postmenopausal | 6.94 |
| GJA5 | Osteoporosis, Postmenopausal | 6.94 |
| GOLGB1 | Osteoporosis, Postmenopausal | 6.94 |
| HTR7 | Osteoporosis, Postmenopausal | 6.94 |
| LAT | Osteoporosis, Postmenopausal | 6.94 |
| LYPD1 | Osteoporosis, Postmenopausal | 6.94 |
| MBOAT1 | Osteoporosis, Postmenopausal | 6.94 |
| MIR146A | Osteoporosis, Postmenopausal | 6.94 |
| MT1M | Osteoporosis, Postmenopausal | 6.94 |
| PALM2AKAP2 | Osteoporosis, Postmenopausal | 6.94 |
| PLEKHA5 | Osteoporosis, Postmenopausal | 6.94 |
| RBM8A | Osteoporosis, Postmenopausal | 6.94 |
| RFX7 | Osteoporosis, Postmenopausal | 6.94 |
| SEMA4G | Osteoporosis, Postmenopausal | 6.94 |
| SSX2IP | Osteoporosis, Postmenopausal | 6.94 |
| VPS35 | Osteoporosis, Postmenopausal | 6.94 |
| AFF3 | Osteoporosis, Postmenopausal | 6.93 |
| AKTIP | Osteoporosis, Postmenopausal | 6.93 |
| EXOSC8 | Osteoporosis, Postmenopausal | 6.93 |
| FGF12 | Osteoporosis, Postmenopausal | 6.93 |
| MIS18A | Osteoporosis, Postmenopausal | 6.93 |
| RPS29 | Osteoporosis, Postmenopausal | 6.93 |
| SDC3 | Osteoporosis, Postmenopausal | 6.93 |
| SYT12 | Osteoporosis, Postmenopausal | 6.93 |
| TFCP2L1 | Osteoporosis, Postmenopausal | 6.93 |
| BCAS3 | Osteoporosis, Postmenopausal | 6.92 |
| CDR2 | Osteoporosis, Postmenopausal | 6.92 |
| CST6 | Osteoporosis, Postmenopausal | 6.92 |
| DDOST | Osteoporosis, Postmenopausal | 6.92 |
| DOCK9 | Osteoporosis, Postmenopausal | 6.92 |
| ECPAS | Osteoporosis, Postmenopausal | 6.92 |
| FBXL20 | Osteoporosis, Postmenopausal | 6.92 |
| FGFBP1 | Osteoporosis, Postmenopausal | 6.92 |
| FIBIN | Osteoporosis, Postmenopausal | 6.92 |
| FXYD1 | Osteoporosis, Postmenopausal | 6.92 |
| HEBP2 | Osteoporosis, Postmenopausal | 6.92 |
| IDS | Osteoporosis, Postmenopausal | 6.92 |
| IGSF3 | Osteoporosis, Postmenopausal | 6.92 |
| KIF5B | Osteoporosis, Postmenopausal | 6.92 |
| KMT5B | Osteoporosis, Postmenopausal | 6.92 |
| LPCAT2 | Osteoporosis, Postmenopausal | 6.92 |
| LYST | Osteoporosis, Postmenopausal | 6.92 |
| MYO5C | Osteoporosis, Postmenopausal | 6.92 |
| NXF1 | Osteoporosis, Postmenopausal | 6.92 |
| OGFRL1 | Osteoporosis, Postmenopausal | 6.92 |
| OLFML2B | Osteoporosis, Postmenopausal | 6.92 |
| P4HA2 | Osteoporosis, Postmenopausal | 6.92 |
| PANX2 | Osteoporosis, Postmenopausal | 6.92 |
| PPP1CA | Osteoporosis, Postmenopausal | 6.92 |
| PXYLP1 | Osteoporosis, Postmenopausal | 6.92 |
| QARS1 | Osteoporosis, Postmenopausal | 6.92 |
| RHBG | Osteoporosis, Postmenopausal | 6.92 |
| SDAD1 | Osteoporosis, Postmenopausal | 6.92 |
| SIN3A | Osteoporosis, Postmenopausal | 6.92 |
| SNRK | Osteoporosis, Postmenopausal | 6.92 |
| SNRPF | Osteoporosis, Postmenopausal | 6.92 |
| STT3A | Osteoporosis, Postmenopausal | 6.92 |
| THRAP3 | Osteoporosis, Postmenopausal | 6.92 |
| TLE2 | Osteoporosis, Postmenopausal | 6.92 |
| TMED3 | Osteoporosis, Postmenopausal | 6.92 |
| TMEM140 | Osteoporosis, Postmenopausal | 6.92 |
| TWF1 | Osteoporosis, Postmenopausal | 6.92 |
| VAPB | Osteoporosis, Postmenopausal | 6.92 |
| ABCF2 | Osteoporosis, Postmenopausal | 6.91 |
| ARF1 | Osteoporosis, Postmenopausal | 6.91 |
| DDX18 | Osteoporosis, Postmenopausal | 6.91 |
| GREM2 | Osteoporosis, Postmenopausal | 6.91 |
| HSPB7 | Osteoporosis, Postmenopausal | 6.91 |
| IRX5 | Osteoporosis, Postmenopausal | 6.91 |
| MRPS27 | Osteoporosis, Postmenopausal | 6.91 |
| NIP7 | Osteoporosis, Postmenopausal | 6.91 |
| NKTR | Osteoporosis, Postmenopausal | 6.91 |
| OSBPL6 | Osteoporosis, Postmenopausal | 6.91 |
| OSR1 | Osteoporosis, Postmenopausal | 6.91 |
| POLR1C | Osteoporosis, Postmenopausal | 6.91 |
| PPP1R2 | Osteoporosis, Postmenopausal | 6.91 |
| PTRH2 | Osteoporosis, Postmenopausal | 6.91 |
| RAB3D | Osteoporosis, Postmenopausal | 6.91 |
| RETREG1 | Osteoporosis, Postmenopausal | 6.91 |
| SCN8A | Osteoporosis, Postmenopausal | 6.91 |
| SLC45A3 | Osteoporosis, Postmenopausal | 6.91 |
| SPEN | Osteoporosis, Postmenopausal | 6.91 |
| ZMYM3 | Osteoporosis, Postmenopausal | 6.91 |
| ABCA3 | Osteoporosis, Postmenopausal | 6.9 |
| ABHD6 | Osteoporosis, Postmenopausal | 6.9 |
| ARID2 | Osteoporosis, Postmenopausal | 6.9 |
| CAP2 | Osteoporosis, Postmenopausal | 6.9 |
| CHD9 | Osteoporosis, Postmenopausal | 6.9 |
| NLE1 | Osteoporosis, Postmenopausal | 6.9 |
| NSFL1C | Osteoporosis, Postmenopausal | 6.9 |
| PIAS1 | Osteoporosis, Postmenopausal | 6.9 |
| SIPA1L1 | Osteoporosis, Postmenopausal | 6.9 |
| TMCC3 | Osteoporosis, Postmenopausal | 6.9 |
| TRIOBP | Osteoporosis, Postmenopausal | 6.9 |
| XPNPEP2 | Osteoporosis, Postmenopausal | 6.9 |
| ADGRG6 | Osteoporosis, Postmenopausal | 6.89 |
| ADIPOR1 | Osteoporosis, Postmenopausal | 6.89 |
| ADNP | Osteoporosis, Postmenopausal | 6.89 |
| AFAP1 | Osteoporosis, Postmenopausal | 6.89 |
| AIF1L | Osteoporosis, Postmenopausal | 6.89 |
| API5 | Osteoporosis, Postmenopausal | 6.89 |
| CCL24 | Osteoporosis, Postmenopausal | 6.89 |
| CDH17 | Osteoporosis, Postmenopausal | 6.89 |
| CELA1 | Osteoporosis, Postmenopausal | 6.89 |
| CEP57 | Osteoporosis, Postmenopausal | 6.89 |
| CHD2 | Osteoporosis, Postmenopausal | 6.89 |
| DNAJB2 | Osteoporosis, Postmenopausal | 6.89 |
| FADS3 | Osteoporosis, Postmenopausal | 6.89 |
| FAM114A1 | Osteoporosis, Postmenopausal | 6.89 |
| GALNT16 | Osteoporosis, Postmenopausal | 6.89 |
| IKBIP | Osteoporosis, Postmenopausal | 6.89 |
| LIG4 | Osteoporosis, Postmenopausal | 6.89 |
| MAP1A | Osteoporosis, Postmenopausal | 6.89 |
| NFYA | Osteoporosis, Postmenopausal | 6.89 |
| NUP50 | Osteoporosis, Postmenopausal | 6.89 |
| PARVA | Osteoporosis, Postmenopausal | 6.89 |
| PHLDB1 | Osteoporosis, Postmenopausal | 6.89 |
| PXMP4 | Osteoporosis, Postmenopausal | 6.89 |
| RAB14 | Osteoporosis, Postmenopausal | 6.89 |
| RPS26 | Osteoporosis, Postmenopausal | 6.89 |
| SLC16A12 | Osteoporosis, Postmenopausal | 6.89 |
| SLC37A2 | Osteoporosis, Postmenopausal | 6.89 |
| SOX5 | Osteoporosis, Postmenopausal | 6.89 |
| TIMM10 | Osteoporosis, Postmenopausal | 6.89 |
| TMEM33 | Osteoporosis, Postmenopausal | 6.89 |
| VAV2 | Osteoporosis, Postmenopausal | 6.89 |
| WLS | Osteoporosis, Postmenopausal | 6.89 |
| ABCA5 | Osteoporosis, Postmenopausal | 6.88 |
| ARHGAP22 | Osteoporosis, Postmenopausal | 6.88 |
| ARHGEF26 | Osteoporosis, Postmenopausal | 6.88 |
| C3AR1 | Osteoporosis, Postmenopausal | 6.88 |
| CGN | Osteoporosis, Postmenopausal | 6.88 |
| COL13A1 | Osteoporosis, Postmenopausal | 6.88 |
| CYP2C6 | Osteoporosis, Postmenopausal | 6.88 |
| CYP4A1 | Osteoporosis, Postmenopausal | 6.88 |
| DLG2 | Osteoporosis, Postmenopausal | 6.88 |
| ELF1 | Osteoporosis, Postmenopausal | 6.88 |
| EPSTI1 | Osteoporosis, Postmenopausal | 6.88 |
| GCGR | Osteoporosis, Postmenopausal | 6.88 |
| IFNA2 | Osteoporosis, Postmenopausal | 6.88 |
| KCNK5 | Osteoporosis, Postmenopausal | 6.88 |
| LARP1B | Osteoporosis, Postmenopausal | 6.88 |
| MAST4 | Osteoporosis, Postmenopausal | 6.88 |
| MCTP1 | Osteoporosis, Postmenopausal | 6.88 |
| PAG1 | Osteoporosis, Postmenopausal | 6.88 |
| PLD2 | Osteoporosis, Postmenopausal | 6.88 |
| PRG4 | Osteoporosis, Postmenopausal | 6.88 |
| PTGFR | Osteoporosis, Postmenopausal | 6.88 |
| RASSF5 | Osteoporosis, Postmenopausal | 6.88 |
| RPS25 | Osteoporosis, Postmenopausal | 6.88 |
| SERINC2 | Osteoporosis, Postmenopausal | 6.88 |
| WNT7A | Osteoporosis, Postmenopausal | 6.88 |
| ADPGK | Osteoporosis, Postmenopausal | 6.87 |
| AOC3 | Osteoporosis, Postmenopausal | 6.87 |
| APOLD1 | Osteoporosis, Postmenopausal | 6.87 |
| BLVRA | Osteoporosis, Postmenopausal | 6.87 |
| CAPZB | Osteoporosis, Postmenopausal | 6.87 |
| DPT | Osteoporosis, Postmenopausal | 6.87 |
| EFNA5 | Osteoporosis, Postmenopausal | 6.87 |
| EP400 | Osteoporosis, Postmenopausal | 6.87 |
| GPRC5B | Osteoporosis, Postmenopausal | 6.87 |
| GSTO2 | Osteoporosis, Postmenopausal | 6.87 |
| KLKB1 | Osteoporosis, Postmenopausal | 6.87 |
| LUC7L | Osteoporosis, Postmenopausal | 6.87 |
| MAMDC2 | Osteoporosis, Postmenopausal | 6.87 |
| MRTO4 | Osteoporosis, Postmenopausal | 6.87 |
| PHKB | Osteoporosis, Postmenopausal | 6.87 |
| PLEKHG5 | Osteoporosis, Postmenopausal | 6.87 |
| PRAG1 | Osteoporosis, Postmenopausal | 6.87 |
| PRKCI | Osteoporosis, Postmenopausal | 6.87 |
| PRMT3 | Osteoporosis, Postmenopausal | 6.87 |
| RNF13 | Osteoporosis, Postmenopausal | 6.87 |
| RRP1B | Osteoporosis, Postmenopausal | 6.87 |
| SEL1L3 | Osteoporosis, Postmenopausal | 6.87 |
| SREK1 | Osteoporosis, Postmenopausal | 6.87 |
| SRGN | Osteoporosis, Postmenopausal | 6.87 |
| TUBA8 | Osteoporosis, Postmenopausal | 6.87 |
| WIPF1 | Osteoporosis, Postmenopausal | 6.87 |
| CCN3 | Osteoporosis, Postmenopausal | 6.86 |
| CDON | Osteoporosis, Postmenopausal | 6.86 |
| GULO | Osteoporosis, Postmenopausal | 6.86 |
| JCHAIN | Osteoporosis, Postmenopausal | 6.86 |
| PAFAH1B3 | Osteoporosis, Postmenopausal | 6.86 |
| RNF125 | Osteoporosis, Postmenopausal | 6.86 |
| TMED10 | Osteoporosis, Postmenopausal | 6.86 |
| UOX | Osteoporosis, Postmenopausal | 6.86 |
| AKR1C14 | Osteoporosis, Postmenopausal | 6.85 |
| APPL2 | Osteoporosis, Postmenopausal | 6.85 |
| ASGR1 | Osteoporosis, Postmenopausal | 6.85 |
| B3GNT5 | Osteoporosis, Postmenopausal | 6.85 |
| CAPN6 | Osteoporosis, Postmenopausal | 6.85 |
| CD52 | Osteoporosis, Postmenopausal | 6.85 |
| CTPS2 | Osteoporosis, Postmenopausal | 6.85 |
| ELK4 | Osteoporosis, Postmenopausal | 6.85 |
| EML2 | Osteoporosis, Postmenopausal | 6.85 |
| FKBP14 | Osteoporosis, Postmenopausal | 6.85 |
| FTL1 | Osteoporosis, Postmenopausal | 6.85 |
| GABRA4 | Osteoporosis, Postmenopausal | 6.85 |
| GRB7 | Osteoporosis, Postmenopausal | 6.85 |
| HJV | Osteoporosis, Postmenopausal | 6.85 |
| HLA-A | Osteoporosis, Postmenopausal | 6.85 |
| KYAT3 | Osteoporosis, Postmenopausal | 6.85 |
| LARP1 | Osteoporosis, Postmenopausal | 6.85 |
| MLLT11 | Osteoporosis, Postmenopausal | 6.85 |
| MMP19 | Osteoporosis, Postmenopausal | 6.85 |
| MYO5B | Osteoporosis, Postmenopausal | 6.85 |
| NANS | Osteoporosis, Postmenopausal | 6.85 |
| PNO1 | Osteoporosis, Postmenopausal | 6.85 |
| RPL28 | Osteoporosis, Postmenopausal | 6.85 |
| SEPTIN11 | Osteoporosis, Postmenopausal | 6.85 |
| SLC25A12 | Osteoporosis, Postmenopausal | 6.85 |
| TMEM106B | Osteoporosis, Postmenopausal | 6.85 |
| WNT5B | Osteoporosis, Postmenopausal | 6.85 |
| WNT7B | Osteoporosis, Postmenopausal | 6.85 |
| ARPC2 | Osteoporosis, Postmenopausal | 6.84 |
| ATP5MF | Osteoporosis, Postmenopausal | 6.84 |
| C9 | Osteoporosis, Postmenopausal | 6.84 |
| COX6B1 | Osteoporosis, Postmenopausal | 6.84 |
| DACT2 | Osteoporosis, Postmenopausal | 6.84 |
| GALNT1 | Osteoporosis, Postmenopausal | 6.84 |
| IL2RB | Osteoporosis, Postmenopausal | 6.84 |
| LAP3 | Osteoporosis, Postmenopausal | 6.84 |
| NRXN1 | Osteoporosis, Postmenopausal | 6.84 |
| PCBP2 | Osteoporosis, Postmenopausal | 6.84 |
| PIK3C2B | Osteoporosis, Postmenopausal | 6.84 |
| PPBP | Osteoporosis, Postmenopausal | 6.84 |
| RAI1 | Osteoporosis, Postmenopausal | 6.84 |
| RGS9 | Osteoporosis, Postmenopausal | 6.84 |
| ROR1 | Osteoporosis, Postmenopausal | 6.84 |
| SLC2A12 | Osteoporosis, Postmenopausal | 6.84 |
| TFDP2 | Osteoporosis, Postmenopausal | 6.84 |
| TMEM37 | Osteoporosis, Postmenopausal | 6.84 |
| VHL | Osteoporosis, Postmenopausal | 6.84 |
| ATP5MG | Osteoporosis, Postmenopausal | 6.83 |
| CAVIN1 | Osteoporosis, Postmenopausal | 6.83 |
| COX7A2 | Osteoporosis, Postmenopausal | 6.83 |
| CPNE8 | Osteoporosis, Postmenopausal | 6.83 |
| CYP2C23 | Osteoporosis, Postmenopausal | 6.83 |
| EMILIN2 | Osteoporosis, Postmenopausal | 6.83 |
| IL4RA | Osteoporosis, Postmenopausal | 6.83 |
| NDUFA1 | Osteoporosis, Postmenopausal | 6.83 |
| NRIP3 | Osteoporosis, Postmenopausal | 6.83 |
| OBSL1 | Osteoporosis, Postmenopausal | 6.83 |
| PCSK1 | Osteoporosis, Postmenopausal | 6.83 |
| PEG3 | Osteoporosis, Postmenopausal | 6.83 |
| RPS9 | Osteoporosis, Postmenopausal | 6.83 |
| S100A14 | Osteoporosis, Postmenopausal | 6.83 |
| SUSD2 | Osteoporosis, Postmenopausal | 6.83 |
| TMOD2 | Osteoporosis, Postmenopausal | 6.83 |
| TMTC1 | Osteoporosis, Postmenopausal | 6.83 |
| TPCN1 | Osteoporosis, Postmenopausal | 6.83 |
| TRIM14 | Osteoporosis, Postmenopausal | 6.83 |
| UQCRB | Osteoporosis, Postmenopausal | 6.83 |
| ANKRD11 | Osteoporosis, Postmenopausal | 6.82 |
| APBB2 | Osteoporosis, Postmenopausal | 6.82 |
| AQP4 | Osteoporosis, Postmenopausal | 6.82 |
| AUTS2 | Osteoporosis, Postmenopausal | 6.82 |
| CD109 | Osteoporosis, Postmenopausal | 6.82 |
| CFL2 | Osteoporosis, Postmenopausal | 6.82 |
| DTNA | Osteoporosis, Postmenopausal | 6.82 |
| HIF3A | Osteoporosis, Postmenopausal | 6.82 |
| HMGN3 | Osteoporosis, Postmenopausal | 6.82 |
| MTARC1 | Osteoporosis, Postmenopausal | 6.82 |
| MYO6 | Osteoporosis, Postmenopausal | 6.82 |
| N4BP2L1 | Osteoporosis, Postmenopausal | 6.82 |
| NDUFV1 | Osteoporosis, Postmenopausal | 6.82 |
| PSMD2 | Osteoporosis, Postmenopausal | 6.82 |
| RASGRP2 | Osteoporosis, Postmenopausal | 6.82 |
| RB1CC1 | Osteoporosis, Postmenopausal | 6.82 |
| RPL34 | Osteoporosis, Postmenopausal | 6.82 |
| SCN4B | Osteoporosis, Postmenopausal | 6.82 |
| SELENOM | Osteoporosis, Postmenopausal | 6.82 |
| SGPL1 | Osteoporosis, Postmenopausal | 6.82 |
| SLC7A6 | Osteoporosis, Postmenopausal | 6.82 |
| SYT7 | Osteoporosis, Postmenopausal | 6.82 |
| TIMP4 | Osteoporosis, Postmenopausal | 6.82 |
| TINAGL1 | Osteoporosis, Postmenopausal | 6.82 |
| TMED5 | Osteoporosis, Postmenopausal | 6.82 |
| TNFSF4 | Osteoporosis, Postmenopausal | 6.82 |
| UQCRH | Osteoporosis, Postmenopausal | 6.82 |
| ATG3 | Osteoporosis, Postmenopausal | 6.81 |
| ATP5PD | Osteoporosis, Postmenopausal | 6.81 |
| BTF3 | Osteoporosis, Postmenopausal | 6.81 |
| COL16A1 | Osteoporosis, Postmenopausal | 6.81 |
| CYP2B9 | Osteoporosis, Postmenopausal | 6.81 |
| GLG1 | Osteoporosis, Postmenopausal | 6.81 |
| IKZF1 | Osteoporosis, Postmenopausal | 6.81 |
| PCDH17 | Osteoporosis, Postmenopausal | 6.81 |
| PHEX | Osteoporosis, Postmenopausal | 6.81 |
| PIK3IP1 | Osteoporosis, Postmenopausal | 6.81 |
| SEMA3E | Osteoporosis, Postmenopausal | 6.81 |
| SETBP1 | Osteoporosis, Postmenopausal | 6.81 |
| SLC44A1 | Osteoporosis, Postmenopausal | 6.81 |
| SLC5A6 | Osteoporosis, Postmenopausal | 6.81 |
| SORCS2 | Osteoporosis, Postmenopausal | 6.81 |
| ASPG | Osteoporosis, Postmenopausal | 6.8 |
| CD151 | Osteoporosis, Postmenopausal | 6.8 |
| DAO | Osteoporosis, Postmenopausal | 6.8 |
| EMX2 | Osteoporosis, Postmenopausal | 6.8 |
| FGF10 | Osteoporosis, Postmenopausal | 6.8 |
| HSD3B7 | Osteoporosis, Postmenopausal | 6.8 |
| KLF3 | Osteoporosis, Postmenopausal | 6.8 |
| MEF2A | Osteoporosis, Postmenopausal | 6.8 |
| MPDZ | Osteoporosis, Postmenopausal | 6.8 |
| MSI2 | Osteoporosis, Postmenopausal | 6.8 |
| PARM1 | Osteoporosis, Postmenopausal | 6.8 |
| PRRC2C | Osteoporosis, Postmenopausal | 6.8 |
| RAD18 | Osteoporosis, Postmenopausal | 6.8 |
| SRSF3 | Osteoporosis, Postmenopausal | 6.8 |
| TRIB2 | Osteoporosis, Postmenopausal | 6.8 |
| VIPR1 | Osteoporosis, Postmenopausal | 6.8 |
| ARL4A | Osteoporosis, Postmenopausal | 6.79 |
| BCOR | Osteoporosis, Postmenopausal | 6.79 |
| BPGM | Osteoporosis, Postmenopausal | 6.79 |
| CAPN5 | Osteoporosis, Postmenopausal | 6.79 |
| CASK | Osteoporosis, Postmenopausal | 6.79 |
| CCDC86 | Osteoporosis, Postmenopausal | 6.79 |
| DOCK10 | Osteoporosis, Postmenopausal | 6.79 |
| HFE | Osteoporosis, Postmenopausal | 6.79 |
| HS3ST3B1 | Osteoporosis, Postmenopausal | 6.79 |
| ME3 | Osteoporosis, Postmenopausal | 6.79 |
| MEG3 | Osteoporosis, Postmenopausal | 6.79 |
| PFAS | Osteoporosis, Postmenopausal | 6.79 |
| PRPS1 | Osteoporosis, Postmenopausal | 6.79 |
| PZP | Osteoporosis, Postmenopausal | 6.79 |
| RASA3 | Osteoporosis, Postmenopausal | 6.79 |
| RPS11 | Osteoporosis, Postmenopausal | 6.79 |
| RUVBL2 | Osteoporosis, Postmenopausal | 6.79 |
| TBX2 | Osteoporosis, Postmenopausal | 6.79 |
| TMEM45B | Osteoporosis, Postmenopausal | 6.79 |
| TRIM28 | Osteoporosis, Postmenopausal | 6.79 |
| TSC1 | Osteoporosis, Postmenopausal | 6.79 |
| WDR1 | Osteoporosis, Postmenopausal | 6.79 |
| ARID1B | Osteoporosis, Postmenopausal | 6.78 |
| BCL2L13 | Osteoporosis, Postmenopausal | 6.78 |
| CLK4 | Osteoporosis, Postmenopausal | 6.78 |
| DLGAP4 | Osteoporosis, Postmenopausal | 6.78 |
| HTT | Osteoporosis, Postmenopausal | 6.78 |
| INF2 | Osteoporosis, Postmenopausal | 6.78 |
| IRF4 | Osteoporosis, Postmenopausal | 6.78 |
| ITIH5 | Osteoporosis, Postmenopausal | 6.78 |
| LPCAT3 | Osteoporosis, Postmenopausal | 6.78 |
| MAP2K7 | Osteoporosis, Postmenopausal | 6.78 |
| MYOG | Osteoporosis, Postmenopausal | 6.78 |
| NPR2 | Osteoporosis, Postmenopausal | 6.78 |
| PDCD6IP | Osteoporosis, Postmenopausal | 6.78 |
| PREX1 | Osteoporosis, Postmenopausal | 6.78 |
| PTER | Osteoporosis, Postmenopausal | 6.78 |
| RBM25 | Osteoporosis, Postmenopausal | 6.78 |
| RPS16 | Osteoporosis, Postmenopausal | 6.78 |
| RPS21 | Osteoporosis, Postmenopausal | 6.78 |
| S1PR2 | Osteoporosis, Postmenopausal | 6.78 |
| SNRNP70 | Osteoporosis, Postmenopausal | 6.78 |
| TMTC4 | Osteoporosis, Postmenopausal | 6.78 |
| TOB1 | Osteoporosis, Postmenopausal | 6.78 |
| TRAM1 | Osteoporosis, Postmenopausal | 6.78 |
| TUBB4A | Osteoporosis, Postmenopausal | 6.78 |
| USP36 | Osteoporosis, Postmenopausal | 6.78 |
| ADI1 | Osteoporosis, Postmenopausal | 6.77 |
| AK3 | Osteoporosis, Postmenopausal | 6.77 |
| DNAJC2 | Osteoporosis, Postmenopausal | 6.77 |
| FNBP4 | Osteoporosis, Postmenopausal | 6.77 |
| GAS2 | Osteoporosis, Postmenopausal | 6.77 |
| GCKR | Osteoporosis, Postmenopausal | 6.77 |
| KCNH1 | Osteoporosis, Postmenopausal | 6.77 |
| KHDRBS1 | Osteoporosis, Postmenopausal | 6.77 |
| MEX3B | Osteoporosis, Postmenopausal | 6.77 |
| NOC3L | Osteoporosis, Postmenopausal | 6.77 |
| SCNN1G | Osteoporosis, Postmenopausal | 6.77 |
| ST3GAL6 | Osteoporosis, Postmenopausal | 6.77 |
| TMEM47 | Osteoporosis, Postmenopausal | 6.77 |
| TRPV4 | Osteoporosis, Postmenopausal | 6.77 |
| ADGRV1 | Osteoporosis, Postmenopausal | 6.76 |
| ADRB3 | Osteoporosis, Postmenopausal | 6.76 |
| CORO2A | Osteoporosis, Postmenopausal | 6.76 |
| DAD1 | Osteoporosis, Postmenopausal | 6.76 |
| DPEP1 | Osteoporosis, Postmenopausal | 6.76 |
| RPS6KA2 | Osteoporosis, Postmenopausal | 6.76 |
| ABTB2 | Osteoporosis, Postmenopausal | 6.75 |
| ACER2 | Osteoporosis, Postmenopausal | 6.75 |
| ALDH8A1 | Osteoporosis, Postmenopausal | 6.75 |
| ARL6IP5 | Osteoporosis, Postmenopausal | 6.75 |
| BOP1 | Osteoporosis, Postmenopausal | 6.75 |
| CES3 | Osteoporosis, Postmenopausal | 6.75 |
| COBL | Osteoporosis, Postmenopausal | 6.75 |
| CSPG4 | Osteoporosis, Postmenopausal | 6.75 |
| CYP2J2 | Osteoporosis, Postmenopausal | 6.75 |
| ELOVL7 | Osteoporosis, Postmenopausal | 6.75 |
| EPM2AIP1 | Osteoporosis, Postmenopausal | 6.75 |
| FGF18 | Osteoporosis, Postmenopausal | 6.75 |
| FRMD4B | Osteoporosis, Postmenopausal | 6.75 |
| GCAT | Osteoporosis, Postmenopausal | 6.75 |
| GNA12 | Osteoporosis, Postmenopausal | 6.75 |
| HGD | Osteoporosis, Postmenopausal | 6.75 |
| IGF2BP2 | Osteoporosis, Postmenopausal | 6.75 |
| MEIS1 | Osteoporosis, Postmenopausal | 6.75 |
| PDE3B | Osteoporosis, Postmenopausal | 6.75 |
| PGAM2 | Osteoporosis, Postmenopausal | 6.75 |
| PLAA | Osteoporosis, Postmenopausal | 6.75 |
| RASGRP3 | Osteoporosis, Postmenopausal | 6.75 |
| RBM33 | Osteoporosis, Postmenopausal | 6.75 |
| RPL22L1 | Osteoporosis, Postmenopausal | 6.75 |
| RPN2 | Osteoporosis, Postmenopausal | 6.75 |
| SRF | Osteoporosis, Postmenopausal | 6.75 |
| SRSF6 | Osteoporosis, Postmenopausal | 6.75 |
| TIMM8B | Osteoporosis, Postmenopausal | 6.75 |
| TM4SF4 | Osteoporosis, Postmenopausal | 6.75 |
| TNIK | Osteoporosis, Postmenopausal | 6.75 |
| TSPAN4 | Osteoporosis, Postmenopausal | 6.75 |
| WASL | Osteoporosis, Postmenopausal | 6.75 |
| WDR77 | Osteoporosis, Postmenopausal | 6.75 |
| ADSS1 | Osteoporosis, Postmenopausal | 6.74 |
| ARAP2 | Osteoporosis, Postmenopausal | 6.74 |
| CHRM4 | Osteoporosis, Postmenopausal | 6.74 |
| CSE1L | Osteoporosis, Postmenopausal | 6.74 |
| DRD4 | Osteoporosis, Postmenopausal | 6.74 |
| EMB | Osteoporosis, Postmenopausal | 6.74 |
| GZMA | Osteoporosis, Postmenopausal | 6.74 |
| MYCBP2 | Osteoporosis, Postmenopausal | 6.74 |
| QPCT | Osteoporosis, Postmenopausal | 6.74 |
| SH2B3 | Osteoporosis, Postmenopausal | 6.74 |
| SH3BGRL3 | Osteoporosis, Postmenopausal | 6.74 |
| SLC38A3 | Osteoporosis, Postmenopausal | 6.74 |
| SMC3 | Osteoporosis, Postmenopausal | 6.74 |
| SRPX | Osteoporosis, Postmenopausal | 6.74 |
| TRA2A | Osteoporosis, Postmenopausal | 6.74 |
| UBE2D3 | Osteoporosis, Postmenopausal | 6.74 |
| ASCL1 | Osteoporosis, Postmenopausal | 6.73 |
| BAIAP2 | Osteoporosis, Postmenopausal | 6.73 |
| BIN1 | Osteoporosis, Postmenopausal | 6.73 |
| CACNA2D1 | Osteoporosis, Postmenopausal | 6.73 |
| CAP1 | Osteoporosis, Postmenopausal | 6.73 |
| CAR2 | Osteoporosis, Postmenopausal | 6.73 |
| CCT4 | Osteoporosis, Postmenopausal | 6.73 |
| CTSZ | Osteoporosis, Postmenopausal | 6.73 |
| EHD2 | Osteoporosis, Postmenopausal | 6.73 |
| EPS8L2 | Osteoporosis, Postmenopausal | 6.73 |
| FAM210B | Osteoporosis, Postmenopausal | 6.73 |
| FASL | Osteoporosis, Postmenopausal | 6.73 |
| FJX1 | Osteoporosis, Postmenopausal | 6.73 |
| IL10RA | Osteoporosis, Postmenopausal | 6.73 |
| KIF18B | Osteoporosis, Postmenopausal | 6.73 |
| KIFC3 | Osteoporosis, Postmenopausal | 6.73 |
| MAG | Osteoporosis, Postmenopausal | 6.73 |
| MAPK7 | Osteoporosis, Postmenopausal | 6.73 |
| NAV1 | Osteoporosis, Postmenopausal | 6.73 |
| ND1 | Osteoporosis, Postmenopausal | 6.73 |
| PFKFB1 | Osteoporosis, Postmenopausal | 6.73 |
| PHB2 | Osteoporosis, Postmenopausal | 6.73 |
| PLP2 | Osteoporosis, Postmenopausal | 6.73 |
| SHANK3 | Osteoporosis, Postmenopausal | 6.73 |
| SUCLA2 | Osteoporosis, Postmenopausal | 6.73 |
| TIAM1 | Osteoporosis, Postmenopausal | 6.73 |
| TMED2 | Osteoporosis, Postmenopausal | 6.73 |
| TNFAIP8L1 | Osteoporosis, Postmenopausal | 6.73 |
| TRA2B | Osteoporosis, Postmenopausal | 6.73 |
| USP10 | Osteoporosis, Postmenopausal | 6.73 |
| AADAT | Osteoporosis, Postmenopausal | 6.72 |
| ACOT4 | Osteoporosis, Postmenopausal | 6.72 |
| ADAM12 | Osteoporosis, Postmenopausal | 6.72 |
| ADGRG2 | Osteoporosis, Postmenopausal | 6.72 |
| ALDH1A7 | Osteoporosis, Postmenopausal | 6.72 |
| CDC42BPA | Osteoporosis, Postmenopausal | 6.72 |
| CFP | Osteoporosis, Postmenopausal | 6.72 |
| FERMT1 | Osteoporosis, Postmenopausal | 6.72 |
| FLG | Osteoporosis, Postmenopausal | 6.72 |
| KCNB1 | Osteoporosis, Postmenopausal | 6.72 |
| KIF5C | Osteoporosis, Postmenopausal | 6.72 |
| MBD1 | Osteoporosis, Postmenopausal | 6.72 |
| ONECUT1 | Osteoporosis, Postmenopausal | 6.72 |
| PARD3 | Osteoporosis, Postmenopausal | 6.72 |
| PSMD4 | Osteoporosis, Postmenopausal | 6.72 |
| SRRT | Osteoporosis, Postmenopausal | 6.72 |
| TLR5 | Osteoporosis, Postmenopausal | 6.72 |
| APOH | Osteoporosis, Postmenopausal | 6.71 |
| COMTD1 | Osteoporosis, Postmenopausal | 6.71 |
| COX6A2 | Osteoporosis, Postmenopausal | 6.71 |
| EMP2 | Osteoporosis, Postmenopausal | 6.71 |
| GABBR1 | Osteoporosis, Postmenopausal | 6.71 |
| GABRA2 | Osteoporosis, Postmenopausal | 6.71 |
| GPM6A | Osteoporosis, Postmenopausal | 6.71 |
| GTPBP2 | Osteoporosis, Postmenopausal | 6.71 |
| KALRN | Osteoporosis, Postmenopausal | 6.71 |
| KANK1 | Osteoporosis, Postmenopausal | 6.71 |
| LMAN1 | Osteoporosis, Postmenopausal | 6.71 |
| LTBP4 | Osteoporosis, Postmenopausal | 6.71 |
| MSX1 | Osteoporosis, Postmenopausal | 6.71 |
| NUDT4 | Osteoporosis, Postmenopausal | 6.71 |
| PSMA3 | Osteoporosis, Postmenopausal | 6.71 |
| RBBP4 | Osteoporosis, Postmenopausal | 6.71 |
| SCG5 | Osteoporosis, Postmenopausal | 6.71 |
| SUOX | Osteoporosis, Postmenopausal | 6.71 |
| TOMM40 | Osteoporosis, Postmenopausal | 6.71 |
| ATOH8 | Osteoporosis, Postmenopausal | 6.7 |
| CAVIN3 | Osteoporosis, Postmenopausal | 6.7 |
| EBNA1BP2 | Osteoporosis, Postmenopausal | 6.7 |
| HS3ST1 | Osteoporosis, Postmenopausal | 6.7 |
| ILK | Osteoporosis, Postmenopausal | 6.7 |
| MBNL1 | Osteoporosis, Postmenopausal | 6.7 |
| MYL6 | Osteoporosis, Postmenopausal | 6.7 |
| NCALD | Osteoporosis, Postmenopausal | 6.7 |
| PCCB | Osteoporosis, Postmenopausal | 6.7 |
| PDPN | Osteoporosis, Postmenopausal | 6.7 |
| RAB32 | Osteoporosis, Postmenopausal | 6.7 |
| RANBP1 | Osteoporosis, Postmenopausal | 6.7 |
| RCC2 | Osteoporosis, Postmenopausal | 6.7 |
| REV1 | Osteoporosis, Postmenopausal | 6.7 |
| RPF2 | Osteoporosis, Postmenopausal | 6.7 |
| SLCO1B3 | Osteoporosis, Postmenopausal | 6.7 |
| ST14 | Osteoporosis, Postmenopausal | 6.7 |
| TECR | Osteoporosis, Postmenopausal | 6.7 |
| UBE3A | Osteoporosis, Postmenopausal | 6.7 |
| GNA13 | Osteoporosis, Postmenopausal | 6.69 |
| GPC3 | Osteoporosis, Postmenopausal | 6.69 |
| HSP70 | Osteoporosis, Postmenopausal | 6.69 |
| LAMA2 | Osteoporosis, Postmenopausal | 6.69 |
| PPP1R9A | Osteoporosis, Postmenopausal | 6.69 |
| PSD3 | Osteoporosis, Postmenopausal | 6.69 |
| SERPINF2 | Osteoporosis, Postmenopausal | 6.69 |
| AGXT | Osteoporosis, Postmenopausal | 6.68 |
| CD93 | Osteoporosis, Postmenopausal | 6.68 |
| DPP7 | Osteoporosis, Postmenopausal | 6.68 |
| EWSR1 | Osteoporosis, Postmenopausal | 6.68 |
| FOXP2 | Osteoporosis, Postmenopausal | 6.68 |
| GSPT1 | Osteoporosis, Postmenopausal | 6.68 |
| OTC | Osteoporosis, Postmenopausal | 6.68 |
| PAM | Osteoporosis, Postmenopausal | 6.68 |
| PHIP | Osteoporosis, Postmenopausal | 6.68 |
| PSMA6 | Osteoporosis, Postmenopausal | 6.68 |
| REST | Osteoporosis, Postmenopausal | 6.68 |
| RUVBL1 | Osteoporosis, Postmenopausal | 6.68 |
| SATB2 | Osteoporosis, Postmenopausal | 6.68 |
| SLBP | Osteoporosis, Postmenopausal | 6.68 |
| SLC27A4 | Osteoporosis, Postmenopausal | 6.68 |
| SLC47A2 | Osteoporosis, Postmenopausal | 6.68 |
| SMPD1 | Osteoporosis, Postmenopausal | 6.68 |
| SRP72 | Osteoporosis, Postmenopausal | 6.68 |
| SRP9 | Osteoporosis, Postmenopausal | 6.68 |
| SUCLG1 | Osteoporosis, Postmenopausal | 6.68 |
| TMBIM1 | Osteoporosis, Postmenopausal | 6.68 |
| ACSS1 | Osteoporosis, Postmenopausal | 6.67 |
| ADHFE1 | Osteoporosis, Postmenopausal | 6.67 |
| C2 | Osteoporosis, Postmenopausal | 6.67 |
| COX7B | Osteoporosis, Postmenopausal | 6.67 |
| COX7C | Osteoporosis, Postmenopausal | 6.67 |
| DHODH | Osteoporosis, Postmenopausal | 6.67 |
| ENTPD1 | Osteoporosis, Postmenopausal | 6.67 |
| FUCA1 | Osteoporosis, Postmenopausal | 6.67 |
| GPATCH4 | Osteoporosis, Postmenopausal | 6.67 |
| IPO4 | Osteoporosis, Postmenopausal | 6.67 |
| MMP15 | Osteoporosis, Postmenopausal | 6.67 |
| NBR1 | Osteoporosis, Postmenopausal | 6.67 |
| NRGN | Osteoporosis, Postmenopausal | 6.67 |
| PLEKHB1 | Osteoporosis, Postmenopausal | 6.67 |
| PRSS12 | Osteoporosis, Postmenopausal | 6.67 |
| YPEL3 | Osteoporosis, Postmenopausal | 6.67 |
| ENPP3 | Osteoporosis, Postmenopausal | 6.66 |
| GRPEL1 | Osteoporosis, Postmenopausal | 6.66 |
| MEIS2 | Osteoporosis, Postmenopausal | 6.66 |
| OAF | Osteoporosis, Postmenopausal | 6.66 |
| PDGFC | Osteoporosis, Postmenopausal | 6.66 |
| PNISR | Osteoporosis, Postmenopausal | 6.66 |
| PRKAB2 | Osteoporosis, Postmenopausal | 6.66 |
| PTBP1 | Osteoporosis, Postmenopausal | 6.66 |
| RCN1 | Osteoporosis, Postmenopausal | 6.66 |
| SLC22A18 | Osteoporosis, Postmenopausal | 6.66 |
| SLC25A30 | Osteoporosis, Postmenopausal | 6.66 |
| TENT5A | Osteoporosis, Postmenopausal | 6.66 |
| XAF1 | Osteoporosis, Postmenopausal | 6.66 |
| CD4 | Osteoporosis, Postmenopausal | 6.65 |
| CD48 | Osteoporosis, Postmenopausal | 6.65 |
| COX6C | Osteoporosis, Postmenopausal | 6.65 |
| DDX60 | Osteoporosis, Postmenopausal | 6.65 |
| DNM2 | Osteoporosis, Postmenopausal | 6.65 |
| EEF1B2 | Osteoporosis, Postmenopausal | 6.65 |
| HDLBP | Osteoporosis, Postmenopausal | 6.65 |
| HPD | Osteoporosis, Postmenopausal | 6.65 |
| LONRF3 | Osteoporosis, Postmenopausal | 6.65 |
| NDRG4 | Osteoporosis, Postmenopausal | 6.65 |
| PPIB | Osteoporosis, Postmenopausal | 6.65 |
| RAB27B | Osteoporosis, Postmenopausal | 6.65 |
| RPN1 | Osteoporosis, Postmenopausal | 6.65 |
| WFS1 | Osteoporosis, Postmenopausal | 6.65 |
| ADH7 | Osteoporosis, Postmenopausal | 6.64 |
| AGPAT3 | Osteoporosis, Postmenopausal | 6.64 |
| ANK2 | Osteoporosis, Postmenopausal | 6.64 |
| ATP5F1C | Osteoporosis, Postmenopausal | 6.64 |
| BACE2 | Osteoporosis, Postmenopausal | 6.64 |
| COX5A | Osteoporosis, Postmenopausal | 6.64 |
| EHD1 | Osteoporosis, Postmenopausal | 6.64 |
| FTCD | Osteoporosis, Postmenopausal | 6.64 |
| GTPBP4 | Osteoporosis, Postmenopausal | 6.64 |
| HMGN2 | Osteoporosis, Postmenopausal | 6.64 |
| HSPB6 | Osteoporosis, Postmenopausal | 6.64 |
| IL13RA2 | Osteoporosis, Postmenopausal | 6.64 |
| NKD1 | Osteoporosis, Postmenopausal | 6.64 |
| SLC22A23 | Osteoporosis, Postmenopausal | 6.64 |
| AGPAT2 | Osteoporosis, Postmenopausal | 6.63 |
| ARID5B | Osteoporosis, Postmenopausal | 6.63 |
| B4GALT5 | Osteoporosis, Postmenopausal | 6.63 |
| CDK7 | Osteoporosis, Postmenopausal | 6.63 |
| COQ10B | Osteoporosis, Postmenopausal | 6.63 |
| CYTB | Osteoporosis, Postmenopausal | 6.63 |
| DNAJC12 | Osteoporosis, Postmenopausal | 6.63 |
| EHD4 | Osteoporosis, Postmenopausal | 6.63 |
| ERCC2 | Osteoporosis, Postmenopausal | 6.63 |
| FANCA | Osteoporosis, Postmenopausal | 6.63 |
| FXYD5 | Osteoporosis, Postmenopausal | 6.63 |
| NAB2 | Osteoporosis, Postmenopausal | 6.63 |
| NCF4 | Osteoporosis, Postmenopausal | 6.63 |
| PDE7B | Osteoporosis, Postmenopausal | 6.63 |
| PEA15 | Osteoporosis, Postmenopausal | 6.63 |
| RAD23A | Osteoporosis, Postmenopausal | 6.63 |
| SLC13A2 | Osteoporosis, Postmenopausal | 6.63 |
| STXBP1 | Osteoporosis, Postmenopausal | 6.63 |
| BCAT2 | Osteoporosis, Postmenopausal | 6.62 |
| BLNK | Osteoporosis, Postmenopausal | 6.62 |
| CDC34 | Osteoporosis, Postmenopausal | 6.62 |
| CDV3 | Osteoporosis, Postmenopausal | 6.62 |
| FRMD6 | Osteoporosis, Postmenopausal | 6.62 |
| GPR155 | Osteoporosis, Postmenopausal | 6.62 |
| KLHL21 | Osteoporosis, Postmenopausal | 6.62 |
| LPXN | Osteoporosis, Postmenopausal | 6.62 |
| PAX3 | Osteoporosis, Postmenopausal | 6.62 |
| PTPN6 | Osteoporosis, Postmenopausal | 6.62 |
| SCARA5 | Osteoporosis, Postmenopausal | 6.62 |
| UBA7 | Osteoporosis, Postmenopausal | 6.62 |
| YBX1 | Osteoporosis, Postmenopausal | 6.62 |
| CNTN1 | Osteoporosis, Postmenopausal | 6.61 |
| CTSA | Osteoporosis, Postmenopausal | 6.61 |
| FNDC5 | Osteoporosis, Postmenopausal | 6.61 |
| INA | Osteoporosis, Postmenopausal | 6.61 |
| MAPRE1 | Osteoporosis, Postmenopausal | 6.61 |
| MT1H | Osteoporosis, Postmenopausal | 6.61 |
| MXD4 | Osteoporosis, Postmenopausal | 6.61 |
| PPP1R14B | Osteoporosis, Postmenopausal | 6.61 |
| RFC5 | Osteoporosis, Postmenopausal | 6.61 |
| TTC3 | Osteoporosis, Postmenopausal | 6.61 |
| TUBG1 | Osteoporosis, Postmenopausal | 6.61 |
| CREBRF | Osteoporosis, Postmenopausal | 6.6 |
| IP6K2 | Osteoporosis, Postmenopausal | 6.6 |
| OAS2 | Osteoporosis, Postmenopausal | 6.6 |
| POLR2A | Osteoporosis, Postmenopausal | 6.6 |
| RHBDF2 | Osteoporosis, Postmenopausal | 6.6 |
| APRT | Osteoporosis, Postmenopausal | 6.59 |
| ATOX1 | Osteoporosis, Postmenopausal | 6.59 |
| BDH2 | Osteoporosis, Postmenopausal | 6.59 |
| BRIP1 | Osteoporosis, Postmenopausal | 6.59 |
| GRB14 | Osteoporosis, Postmenopausal | 6.59 |
| ICAM2 | Osteoporosis, Postmenopausal | 6.59 |
| METAP2 | Osteoporosis, Postmenopausal | 6.59 |
| MPZL2 | Osteoporosis, Postmenopausal | 6.59 |
| NARS1 | Osteoporosis, Postmenopausal | 6.59 |
| PARP9 | Osteoporosis, Postmenopausal | 6.59 |
| SET | Osteoporosis, Postmenopausal | 6.59 |
| SLC6A13 | Osteoporosis, Postmenopausal | 6.59 |
| TLR7 | Osteoporosis, Postmenopausal | 6.59 |
| ADRM1 | Osteoporosis, Postmenopausal | 6.58 |
| AMD1 | Osteoporosis, Postmenopausal | 6.58 |
| CALB2 | Osteoporosis, Postmenopausal | 6.58 |
| CELF2 | Osteoporosis, Postmenopausal | 6.58 |
| CGB3 | Osteoporosis, Postmenopausal | 6.58 |
| CHGA | Osteoporosis, Postmenopausal | 6.58 |
| CUX1 | Osteoporosis, Postmenopausal | 6.58 |
| CYC1 | Osteoporosis, Postmenopausal | 6.58 |
| ETFDH | Osteoporosis, Postmenopausal | 6.58 |
| FAM111A | Osteoporosis, Postmenopausal | 6.58 |
| HOMER1 | Osteoporosis, Postmenopausal | 6.58 |
| KLF13 | Osteoporosis, Postmenopausal | 6.58 |
| MTF1 | Osteoporosis, Postmenopausal | 6.58 |
| PADI2 | Osteoporosis, Postmenopausal | 6.58 |
| PTAFR | Osteoporosis, Postmenopausal | 6.58 |
| RBPJ | Osteoporosis, Postmenopausal | 6.58 |
| RPL27A | Osteoporosis, Postmenopausal | 6.58 |
| SYNM | Osteoporosis, Postmenopausal | 6.58 |
| GNG11 | Osteoporosis, Postmenopausal | 6.57 |
| ITIH3 | Osteoporosis, Postmenopausal | 6.57 |
| PDIA5 | Osteoporosis, Postmenopausal | 6.57 |
| RPS20 | Osteoporosis, Postmenopausal | 6.57 |
| SEL1L | Osteoporosis, Postmenopausal | 6.57 |
| YWHAB | Osteoporosis, Postmenopausal | 6.57 |
| AP1S1 | Osteoporosis, Postmenopausal | 6.56 |
| CYP2C87 | Osteoporosis, Postmenopausal | 6.56 |
| GULP1 | Osteoporosis, Postmenopausal | 6.56 |
| GYS2 | Osteoporosis, Postmenopausal | 6.56 |
| JMJD1C | Osteoporosis, Postmenopausal | 6.56 |
| MYL1 | Osteoporosis, Postmenopausal | 6.56 |
| NEBL | Osteoporosis, Postmenopausal | 6.56 |
| PAPSS1 | Osteoporosis, Postmenopausal | 6.56 |
| PNPLA8 | Osteoporosis, Postmenopausal | 6.56 |
| PYGB | Osteoporosis, Postmenopausal | 6.56 |
| RNF19B | Osteoporosis, Postmenopausal | 6.56 |
| SAA3 | Osteoporosis, Postmenopausal | 6.56 |
| SDCBP | Osteoporosis, Postmenopausal | 6.56 |
| SLC25A1 | Osteoporosis, Postmenopausal | 6.56 |
| SOAT1 | Osteoporosis, Postmenopausal | 6.56 |
| SOX18 | Osteoporosis, Postmenopausal | 6.56 |
| SPAG9 | Osteoporosis, Postmenopausal | 6.56 |
| TBC1D4 | Osteoporosis, Postmenopausal | 6.56 |
| TBX21 | Osteoporosis, Postmenopausal | 6.56 |
| WDR43 | Osteoporosis, Postmenopausal | 6.56 |
| ATP6 | Osteoporosis, Postmenopausal | 6.55 |
| CD276 | Osteoporosis, Postmenopausal | 6.55 |
| COQ8A | Osteoporosis, Postmenopausal | 6.55 |
| CPQ | Osteoporosis, Postmenopausal | 6.55 |
| FNBP1 | Osteoporosis, Postmenopausal | 6.55 |
| MRPL12 | Osteoporosis, Postmenopausal | 6.55 |
| PPAT | Osteoporosis, Postmenopausal | 6.55 |
| PRDM16 | Osteoporosis, Postmenopausal | 6.55 |
| PRKAB1 | Osteoporosis, Postmenopausal | 6.55 |
| PTN | Osteoporosis, Postmenopausal | 6.55 |
| RALA | Osteoporosis, Postmenopausal | 6.55 |
| RBBP6 | Osteoporosis, Postmenopausal | 6.55 |
| RIF1 | Osteoporosis, Postmenopausal | 6.55 |
| RPL10 | Osteoporosis, Postmenopausal | 6.55 |
| RPL32 | Osteoporosis, Postmenopausal | 6.55 |
| SLC25A5 | Osteoporosis, Postmenopausal | 6.55 |
| SLC46A3 | Osteoporosis, Postmenopausal | 6.55 |
| TBL1XR1 | Osteoporosis, Postmenopausal | 6.55 |
| ADRA2A | Osteoporosis, Postmenopausal | 6.54 |
| ATIC | Osteoporosis, Postmenopausal | 6.54 |
| GPCPD1 | Osteoporosis, Postmenopausal | 6.54 |
| ITGB8 | Osteoporosis, Postmenopausal | 6.54 |
| MSLN | Osteoporosis, Postmenopausal | 6.54 |
| RGS3 | Osteoporosis, Postmenopausal | 6.54 |
| ATP5IF1 | Osteoporosis, Postmenopausal | 6.53 |
| CLEC7A | Osteoporosis, Postmenopausal | 6.53 |
| DDIT4L | Osteoporosis, Postmenopausal | 6.53 |
| FDX1 | Osteoporosis, Postmenopausal | 6.53 |
| HNRNPC | Osteoporosis, Postmenopausal | 6.53 |
| PRF1 | Osteoporosis, Postmenopausal | 6.53 |
| PSMC6 | Osteoporosis, Postmenopausal | 6.53 |
| PVALB | Osteoporosis, Postmenopausal | 6.53 |
| RAB3B | Osteoporosis, Postmenopausal | 6.53 |
| SLIT3 | Osteoporosis, Postmenopausal | 6.53 |
| ACSF2 | Osteoporosis, Postmenopausal | 6.52 |
| ANTXR1 | Osteoporosis, Postmenopausal | 6.52 |
| ARID1A | Osteoporosis, Postmenopausal | 6.52 |
| ARL4D | Osteoporosis, Postmenopausal | 6.52 |
| ASL | Osteoporosis, Postmenopausal | 6.52 |
| CBLB | Osteoporosis, Postmenopausal | 6.52 |
| CD34 | Osteoporosis, Postmenopausal | 6.52 |
| CDH3 | Osteoporosis, Postmenopausal | 6.52 |
| GPX7 | Osteoporosis, Postmenopausal | 6.52 |
| GSK3A | Osteoporosis, Postmenopausal | 6.52 |
| HIBADH | Osteoporosis, Postmenopausal | 6.52 |
| LPIN2 | Osteoporosis, Postmenopausal | 6.52 |
| NEFM | Osteoporosis, Postmenopausal | 6.52 |
| PLS1 | Osteoporosis, Postmenopausal | 6.52 |
| PRKG1 | Osteoporosis, Postmenopausal | 6.52 |
| RPL3 | Osteoporosis, Postmenopausal | 6.52 |
| SCN5A | Osteoporosis, Postmenopausal | 6.52 |
| SLC25A13 | Osteoporosis, Postmenopausal | 6.52 |
| SRSF11 | Osteoporosis, Postmenopausal | 6.52 |
| TRAF3 | Osteoporosis, Postmenopausal | 6.52 |
| AASS | Osteoporosis, Postmenopausal | 6.51 |
| ALDH9A1 | Osteoporosis, Postmenopausal | 6.51 |
| GPI | Osteoporosis, Postmenopausal | 6.51 |
| MASP1 | Osteoporosis, Postmenopausal | 6.51 |
| SAMHD1 | Osteoporosis, Postmenopausal | 6.51 |
| SLC27A1 | Osteoporosis, Postmenopausal | 6.51 |
| ADAM10 | Osteoporosis, Postmenopausal | 6.5 |
| ALDH5A1 | Osteoporosis, Postmenopausal | 6.5 |
| ATP2B4 | Osteoporosis, Postmenopausal | 6.5 |
| ETNK1 | Osteoporosis, Postmenopausal | 6.5 |
| FOXQ1 | Osteoporosis, Postmenopausal | 6.5 |
| GART | Osteoporosis, Postmenopausal | 6.5 |
| MRPS18B | Osteoporosis, Postmenopausal | 6.5 |
| NDUFB8 | Osteoporosis, Postmenopausal | 6.5 |
| NME2 | Osteoporosis, Postmenopausal | 6.5 |
| PDE3A | Osteoporosis, Postmenopausal | 6.5 |
| SLC16A10 | Osteoporosis, Postmenopausal | 6.5 |
| UBE2L6 | Osteoporosis, Postmenopausal | 6.5 |
| ACOT7 | Osteoporosis, Postmenopausal | 6.49 |
| AFF4 | Osteoporosis, Postmenopausal | 6.49 |
| AZIN1 | Osteoporosis, Postmenopausal | 6.49 |
| BAAT | Osteoporosis, Postmenopausal | 6.49 |
| C6 | Osteoporosis, Postmenopausal | 6.49 |
| CCT5 | Osteoporosis, Postmenopausal | 6.49 |
| CDH13 | Osteoporosis, Postmenopausal | 6.49 |
| CHST15 | Osteoporosis, Postmenopausal | 6.49 |
| COL27A1 | Osteoporosis, Postmenopausal | 6.49 |
| CRK | Osteoporosis, Postmenopausal | 6.49 |
| EIF3A | Osteoporosis, Postmenopausal | 6.49 |
| ETHE1 | Osteoporosis, Postmenopausal | 6.49 |
| HSP70A | Osteoporosis, Postmenopausal | 6.49 |
| NDUFS4 | Osteoporosis, Postmenopausal | 6.49 |
| OGDH | Osteoporosis, Postmenopausal | 6.49 |
| PSMC3 | Osteoporosis, Postmenopausal | 6.49 |
| SNX10 | Osteoporosis, Postmenopausal | 6.49 |
| TAX1BP3 | Osteoporosis, Postmenopausal | 6.49 |
| ADH5 | Osteoporosis, Postmenopausal | 6.48 |
| AKR7A2 | Osteoporosis, Postmenopausal | 6.48 |
| ENTPD5 | Osteoporosis, Postmenopausal | 6.48 |
| FMR1 | Osteoporosis, Postmenopausal | 6.48 |
| GFPT1 | Osteoporosis, Postmenopausal | 6.48 |
| HSD17B6 | Osteoporosis, Postmenopausal | 6.48 |
| LAMB2 | Osteoporosis, Postmenopausal | 6.48 |
| MERTK | Osteoporosis, Postmenopausal | 6.48 |
| SLC34A2 | Osteoporosis, Postmenopausal | 6.48 |
| WWTR1 | Osteoporosis, Postmenopausal | 6.48 |
| ANK3 | Osteoporosis, Postmenopausal | 6.47 |
| ARRDC4 | Osteoporosis, Postmenopausal | 6.47 |
| CEMIP2 | Osteoporosis, Postmenopausal | 6.47 |
| EIF6 | Osteoporosis, Postmenopausal | 6.47 |
| ESCO2 | Osteoporosis, Postmenopausal | 6.47 |
| HSDL2 | Osteoporosis, Postmenopausal | 6.47 |
| P2RX4 | Osteoporosis, Postmenopausal | 6.47 |
| PLCB4 | Osteoporosis, Postmenopausal | 6.47 |
| UTRN | Osteoporosis, Postmenopausal | 6.47 |
| ZMYND8 | Osteoporosis, Postmenopausal | 6.47 |
| ESRRG | Osteoporosis, Postmenopausal | 6.46 |
| JAK3 | Osteoporosis, Postmenopausal | 6.46 |
| LTA | Osteoporosis, Postmenopausal | 6.46 |
| PDHB | Osteoporosis, Postmenopausal | 6.46 |
| PIK3CG | Osteoporosis, Postmenopausal | 6.46 |
| PSMB6 | Osteoporosis, Postmenopausal | 6.46 |
| RAB30 | Osteoporosis, Postmenopausal | 6.46 |
| TCP11L2 | Osteoporosis, Postmenopausal | 6.46 |
| ARPP19 | Osteoporosis, Postmenopausal | 6.45 |
| AVPR1A | Osteoporosis, Postmenopausal | 6.45 |
| FGL2 | Osteoporosis, Postmenopausal | 6.45 |
| FKBP1A | Osteoporosis, Postmenopausal | 6.45 |
| MID1IP1 | Osteoporosis, Postmenopausal | 6.45 |
| NFIC | Osteoporosis, Postmenopausal | 6.45 |
| PDE2A | Osteoporosis, Postmenopausal | 6.45 |
| PHYH | Osteoporosis, Postmenopausal | 6.45 |
| SARDH | Osteoporosis, Postmenopausal | 6.45 |
| TKFC | Osteoporosis, Postmenopausal | 6.45 |
| TNIP1 | Osteoporosis, Postmenopausal | 6.45 |
| ANTXR2 | Osteoporosis, Postmenopausal | 6.44 |
| FMO2 | Osteoporosis, Postmenopausal | 6.44 |
| GHRL | Osteoporosis, Postmenopausal | 6.44 |
| IMPA2 | Osteoporosis, Postmenopausal | 6.44 |
| NPC2 | Osteoporosis, Postmenopausal | 6.44 |
| PRKCH | Osteoporosis, Postmenopausal | 6.44 |
| BTG3 | Osteoporosis, Postmenopausal | 6.43 |
| CBL | Osteoporosis, Postmenopausal | 6.43 |
| CLIP1 | Osteoporosis, Postmenopausal | 6.43 |
| CRIM1 | Osteoporosis, Postmenopausal | 6.43 |
| CRYL1 | Osteoporosis, Postmenopausal | 6.43 |
| CRYM | Osteoporosis, Postmenopausal | 6.43 |
| ENG | Osteoporosis, Postmenopausal | 6.43 |
| GRIN2C | Osteoporosis, Postmenopausal | 6.43 |
| HAT1 | Osteoporosis, Postmenopausal | 6.43 |
| LRAT | Osteoporosis, Postmenopausal | 6.43 |
| PEPD | Osteoporosis, Postmenopausal | 6.43 |
| PRODH | Osteoporosis, Postmenopausal | 6.43 |
| ROBO1 | Osteoporosis, Postmenopausal | 6.43 |
| TRIO | Osteoporosis, Postmenopausal | 6.43 |
| ACTN4 | Osteoporosis, Postmenopausal | 6.42 |
| BBOX1 | Osteoporosis, Postmenopausal | 6.42 |
| CALU | Osteoporosis, Postmenopausal | 6.42 |
| COL15A1 | Osteoporosis, Postmenopausal | 6.42 |
| JADE1 | Osteoporosis, Postmenopausal | 6.42 |
| MAL | Osteoporosis, Postmenopausal | 6.42 |
| RSRC2 | Osteoporosis, Postmenopausal | 6.42 |
| SH3BP5 | Osteoporosis, Postmenopausal | 6.42 |
| TRIM2 | Osteoporosis, Postmenopausal | 6.42 |
| VGF | Osteoporosis, Postmenopausal | 6.42 |
| WNT3A | Osteoporosis, Postmenopausal | 6.42 |
| ACTC1 | Osteoporosis, Postmenopausal | 6.41 |
| AKAP9 | Osteoporosis, Postmenopausal | 6.41 |
| ANKRD12 | Osteoporosis, Postmenopausal | 6.41 |
| APCS | Osteoporosis, Postmenopausal | 6.41 |
| CACNA1C | Osteoporosis, Postmenopausal | 6.41 |
| FKBP11 | Osteoporosis, Postmenopausal | 6.41 |
| IGFALS | Osteoporosis, Postmenopausal | 6.41 |
| IRF8 | Osteoporosis, Postmenopausal | 6.41 |
| KIF22 | Osteoporosis, Postmenopausal | 6.41 |
| PPP2R2B | Osteoporosis, Postmenopausal | 6.41 |
| SLC13A3 | Osteoporosis, Postmenopausal | 6.41 |
| CSAD | Osteoporosis, Postmenopausal | 6.4 |
| CXCR2 | Osteoporosis, Postmenopausal | 6.4 |
| HMOX2 | Osteoporosis, Postmenopausal | 6.4 |
| LMO7 | Osteoporosis, Postmenopausal | 6.4 |
| MT1G | Osteoporosis, Postmenopausal | 6.4 |
| NCEH1 | Osteoporosis, Postmenopausal | 6.4 |
| RARRES2 | Osteoporosis, Postmenopausal | 6.4 |
| SLC2A5 | Osteoporosis, Postmenopausal | 6.4 |
| AACS | Osteoporosis, Postmenopausal | 6.39 |
| DKK3 | Osteoporosis, Postmenopausal | 6.39 |
| MPST | Osteoporosis, Postmenopausal | 6.39 |
| NECTIN2 | Osteoporosis, Postmenopausal | 6.39 |
| SIRT2 | Osteoporosis, Postmenopausal | 6.39 |
| BHLHE41 | Osteoporosis, Postmenopausal | 6.38 |
| CREB5 | Osteoporosis, Postmenopausal | 6.38 |
| CXCL13 | Osteoporosis, Postmenopausal | 6.38 |
| GSTT3 | Osteoporosis, Postmenopausal | 6.38 |
| IRF2BP2 | Osteoporosis, Postmenopausal | 6.38 |
| MFGE8 | Osteoporosis, Postmenopausal | 6.38 |
| PDK2 | Osteoporosis, Postmenopausal | 6.38 |
| PSMB1 | Osteoporosis, Postmenopausal | 6.38 |
| PTPRK | Osteoporosis, Postmenopausal | 6.38 |
| RPL23 | Osteoporosis, Postmenopausal | 6.38 |
| SLC16A2 | Osteoporosis, Postmenopausal | 6.38 |
| TCP1 | Osteoporosis, Postmenopausal | 6.38 |
| TPMT | Osteoporosis, Postmenopausal | 6.38 |
| XPA | Osteoporosis, Postmenopausal | 6.38 |
| CCT8 | Osteoporosis, Postmenopausal | 6.37 |
| CXCL11 | Osteoporosis, Postmenopausal | 6.37 |
| GATA2 | Osteoporosis, Postmenopausal | 6.37 |
| MSRA | Osteoporosis, Postmenopausal | 6.37 |
| GABRB3 | Osteoporosis, Postmenopausal | 6.36 |
| GSDME | Osteoporosis, Postmenopausal | 6.36 |
| HILPDA | Osteoporosis, Postmenopausal | 6.36 |
| PAWR | Osteoporosis, Postmenopausal | 6.36 |
| PDE4DIP | Osteoporosis, Postmenopausal | 6.36 |
| HDAC4 | Osteoporosis, Postmenopausal | 6.35 |
| KDM6B | Osteoporosis, Postmenopausal | 6.35 |
| KRT10 | Osteoporosis, Postmenopausal | 6.35 |
| LIPA | Osteoporosis, Postmenopausal | 6.35 |
| LOXL1 | Osteoporosis, Postmenopausal | 6.35 |
| PRKAR2B | Osteoporosis, Postmenopausal | 6.35 |
| RASSF4 | Osteoporosis, Postmenopausal | 6.35 |
| SLC25A37 | Osteoporosis, Postmenopausal | 6.35 |
| SULF1 | Osteoporosis, Postmenopausal | 6.35 |
| TSLP | Osteoporosis, Postmenopausal | 6.35 |
| ANGPT2 | Osteoporosis, Postmenopausal | 6.34 |
| CAPRIN1 | Osteoporosis, Postmenopausal | 6.34 |
| DYNC1H1 | Osteoporosis, Postmenopausal | 6.34 |
| HHEX | Osteoporosis, Postmenopausal | 6.34 |
| IRF9 | Osteoporosis, Postmenopausal | 6.34 |
| LCK | Osteoporosis, Postmenopausal | 6.34 |
| PMVK | Osteoporosis, Postmenopausal | 6.34 |
| SMC1A | Osteoporosis, Postmenopausal | 6.34 |
| ARHGEF2 | Osteoporosis, Postmenopausal | 6.33 |
| ARRB1 | Osteoporosis, Postmenopausal | 6.33 |
| CALM2 | Osteoporosis, Postmenopausal | 6.33 |
| FERMT2 | Osteoporosis, Postmenopausal | 6.33 |
| GCHFR | Osteoporosis, Postmenopausal | 6.33 |
| GREM1 | Osteoporosis, Postmenopausal | 6.33 |
| OXCT1 | Osteoporosis, Postmenopausal | 6.33 |
| PSMD12 | Osteoporosis, Postmenopausal | 6.33 |
| SMOC1 | Osteoporosis, Postmenopausal | 6.33 |
| SRSF2 | Osteoporosis, Postmenopausal | 6.33 |
| UMPS | Osteoporosis, Postmenopausal | 6.33 |
| BACH1 | Osteoporosis, Postmenopausal | 6.32 |
| CKAP4 | Osteoporosis, Postmenopausal | 6.32 |
| CYB5B | Osteoporosis, Postmenopausal | 6.32 |
| DUSP8 | Osteoporosis, Postmenopausal | 6.32 |
| FOLR1 | Osteoporosis, Postmenopausal | 6.32 |
| HDAC6 | Osteoporosis, Postmenopausal | 6.32 |
| INMT | Osteoporosis, Postmenopausal | 6.32 |
| PDXK | Osteoporosis, Postmenopausal | 6.32 |
| DSP | Osteoporosis, Postmenopausal | 6.31 |
| PF4 | Osteoporosis, Postmenopausal | 6.31 |
| BRD2 | Osteoporosis, Postmenopausal | 6.3 |
| CCNL1 | Osteoporosis, Postmenopausal | 6.3 |
| MACF1 | Osteoporosis, Postmenopausal | 6.3 |
| REEP6 | Osteoporosis, Postmenopausal | 6.3 |
| SYNE1 | Osteoporosis, Postmenopausal | 6.3 |
| TMSB4X | Osteoporosis, Postmenopausal | 6.3 |
| BCLAF1 | Osteoporosis, Postmenopausal | 6.29 |
| BOK | Osteoporosis, Postmenopausal | 6.29 |
| CADM1 | Osteoporosis, Postmenopausal | 6.29 |
| CCL9 | Osteoporosis, Postmenopausal | 6.29 |
| LAMA5 | Osteoporosis, Postmenopausal | 6.29 |
| PRKAR1A | Osteoporosis, Postmenopausal | 6.29 |
| RAD23B | Osteoporosis, Postmenopausal | 6.29 |
| RIGI | Osteoporosis, Postmenopausal | 6.29 |
| SLC6A2 | Osteoporosis, Postmenopausal | 6.29 |
| ACADS | Osteoporosis, Postmenopausal | 6.28 |
| CAMK4 | Osteoporosis, Postmenopausal | 6.28 |
| EIF4B | Osteoporosis, Postmenopausal | 6.28 |
| GNAI1 | Osteoporosis, Postmenopausal | 6.28 |
| MFSD2A | Osteoporosis, Postmenopausal | 6.28 |
| NNMT | Osteoporosis, Postmenopausal | 6.28 |
| PRDX4 | Osteoporosis, Postmenopausal | 6.28 |
| TPM2 | Osteoporosis, Postmenopausal | 6.28 |
| GLI2 | Osteoporosis, Postmenopausal | 6.27 |
| NCOA7 | Osteoporosis, Postmenopausal | 6.27 |
| TPT1 | Osteoporosis, Postmenopausal | 6.27 |
| DPYSL3 | Osteoporosis, Postmenopausal | 6.26 |
| ELN | Osteoporosis, Postmenopausal | 6.26 |
| FBN2 | Osteoporosis, Postmenopausal | 6.26 |
| MANF | Osteoporosis, Postmenopausal | 6.26 |
| C4B | Osteoporosis, Postmenopausal | 6.25 |
| CLDN2 | Osteoporosis, Postmenopausal | 6.25 |
| CSRNP1 | Osteoporosis, Postmenopausal | 6.25 |
| IFI27 | Osteoporosis, Postmenopausal | 6.25 |
| NT5DC2 | Osteoporosis, Postmenopausal | 6.25 |
| PROCR | Osteoporosis, Postmenopausal | 6.25 |
| VTN | Osteoporosis, Postmenopausal | 6.25 |
| ZMAT3 | Osteoporosis, Postmenopausal | 6.25 |
| CEBPG | Osteoporosis, Postmenopausal | 6.24 |
| OPRD1 | Osteoporosis, Postmenopausal | 6.24 |
| PARK7 | Osteoporosis, Postmenopausal | 6.24 |
| PARP14 | Osteoporosis, Postmenopausal | 6.24 |
| UQCRC2 | Osteoporosis, Postmenopausal | 6.24 |
| CAV2 | Osteoporosis, Postmenopausal | 6.23 |
| CD81 | Osteoporosis, Postmenopausal | 6.23 |
| CSTB | Osteoporosis, Postmenopausal | 6.23 |
| DKC1 | Osteoporosis, Postmenopausal | 6.23 |
| FAH | Osteoporosis, Postmenopausal | 6.23 |
| FLCN | Osteoporosis, Postmenopausal | 6.23 |
| HES6 | Osteoporosis, Postmenopausal | 6.23 |
| HEY1 | Osteoporosis, Postmenopausal | 6.23 |
| ST6GAL1 | Osteoporosis, Postmenopausal | 6.23 |
| ACO2 | Osteoporosis, Postmenopausal | 6.22 |
| CSNK2A1 | Osteoporosis, Postmenopausal | 6.22 |
| FCGR2B | Osteoporosis, Postmenopausal | 6.22 |
| ITGAL | Osteoporosis, Postmenopausal | 6.22 |
| LY96 | Osteoporosis, Postmenopausal | 6.22 |
| MAT1A | Osteoporosis, Postmenopausal | 6.22 |
| PGM1 | Osteoporosis, Postmenopausal | 6.22 |
| PSMB9 | Osteoporosis, Postmenopausal | 6.22 |
| PSMC5 | Osteoporosis, Postmenopausal | 6.22 |
| RDX | Osteoporosis, Postmenopausal | 6.22 |
| TYMP | Osteoporosis, Postmenopausal | 6.22 |
| AKR1D1 | Osteoporosis, Postmenopausal | 6.21 |
| CRABP2 | Osteoporosis, Postmenopausal | 6.21 |
| EEF2K | Osteoporosis, Postmenopausal | 6.21 |
| KRT7 | Osteoporosis, Postmenopausal | 6.21 |
| NOP56 | Osteoporosis, Postmenopausal | 6.21 |
| PSMB5 | Osteoporosis, Postmenopausal | 6.21 |
| SDHB | Osteoporosis, Postmenopausal | 6.21 |
| SHANK2 | Osteoporosis, Postmenopausal | 6.21 |
| TXNL1 | Osteoporosis, Postmenopausal | 6.21 |
| CHAT | Osteoporosis, Postmenopausal | 6.2 |
| DDX5 | Osteoporosis, Postmenopausal | 6.2 |
| NR1D2 | Osteoporosis, Postmenopausal | 6.2 |
| SLC16A6 | Osteoporosis, Postmenopausal | 6.2 |
| OGA | Osteoporosis, Postmenopausal | 6.19 |
| OSMR | Osteoporosis, Postmenopausal | 6.19 |
| CHKA | Osteoporosis, Postmenopausal | 6.18 |
| COL4A2 | Osteoporosis, Postmenopausal | 6.18 |
| GLO1 | Osteoporosis, Postmenopausal | 6.18 |
| MAP2K4 | Osteoporosis, Postmenopausal | 6.18 |
| SHMT1 | Osteoporosis, Postmenopausal | 6.18 |
| ALDH18A1 | Osteoporosis, Postmenopausal | 6.17 |
| FBLN5 | Osteoporosis, Postmenopausal | 6.17 |
| FSHR | Osteoporosis, Postmenopausal | 6.17 |
| NR5A2 | Osteoporosis, Postmenopausal | 6.17 |
| DDX3X | Osteoporosis, Postmenopausal | 6.16 |
| LAMA3 | Osteoporosis, Postmenopausal | 6.16 |
| PFKM | Osteoporosis, Postmenopausal | 6.16 |
| SERTAD1 | Osteoporosis, Postmenopausal | 6.16 |
| UBD | Osteoporosis, Postmenopausal | 6.16 |
| ACADVL | Osteoporosis, Postmenopausal | 6.15 |
| ERO1A | Osteoporosis, Postmenopausal | 6.15 |
| PEX11A | Osteoporosis, Postmenopausal | 6.15 |
| SLC4A4 | Osteoporosis, Postmenopausal | 6.15 |
| TST | Osteoporosis, Postmenopausal | 6.15 |
| FABP7 | Osteoporosis, Postmenopausal | 6.14 |
| HDAC5 | Osteoporosis, Postmenopausal | 6.14 |
| IKBKG | Osteoporosis, Postmenopausal | 6.14 |
| KLF10 | Osteoporosis, Postmenopausal | 6.13 |
| MTHFD1L | Osteoporosis, Postmenopausal | 6.13 |
| OGT | Osteoporosis, Postmenopausal | 6.13 |
| PTPN11 | Osteoporosis, Postmenopausal | 6.13 |
| SMAD4 | Osteoporosis, Postmenopausal | 6.13 |
| FOXP1 | Osteoporosis, Postmenopausal | 6.12 |
| IGFBP7 | Osteoporosis, Postmenopausal | 6.12 |
| PSEN1 | Osteoporosis, Postmenopausal | 6.12 |
| RETSAT | Osteoporosis, Postmenopausal | 6.12 |
| AGRN | Osteoporosis, Postmenopausal | 6.11 |
| GPD2 | Osteoporosis, Postmenopausal | 6.11 |
| PID1 | Osteoporosis, Postmenopausal | 6.11 |
| SIK1 | Osteoporosis, Postmenopausal | 6.11 |
| TAT | Osteoporosis, Postmenopausal | 6.11 |
| ADAM17 | Osteoporosis, Postmenopausal | 6.1 |
| ALDH1L2 | Osteoporosis, Postmenopausal | 6.1 |
| APOA2 | Osteoporosis, Postmenopausal | 6.1 |
| FGFR4 | Osteoporosis, Postmenopausal | 6.1 |
| MX2 | Osteoporosis, Postmenopausal | 6.1 |
| PAPSS2 | Osteoporosis, Postmenopausal | 6.1 |
| PTP4A1 | Osteoporosis, Postmenopausal | 6.1 |
| YARS1 | Osteoporosis, Postmenopausal | 6.1 |
| ADGRE1 | Osteoporosis, Postmenopausal | 6.09 |
| CLDN5 | Osteoporosis, Postmenopausal | 6.09 |
| CROT | Osteoporosis, Postmenopausal | 6.09 |
| NFIB | Osteoporosis, Postmenopausal | 6.09 |
| CA-ALPHA1D | Osteoporosis, Postmenopausal | 6.08 |
| CYP2C11 | Osteoporosis, Postmenopausal | 6.08 |
| HTR1A | Osteoporosis, Postmenopausal | 6.08 |
| NCAM1 | Osteoporosis, Postmenopausal | 6.08 |
| PEBP1 | Osteoporosis, Postmenopausal | 6.08 |
| SP120 | Osteoporosis, Postmenopausal | 6.08 |
| GARS1 | Osteoporosis, Postmenopausal | 6.07 |
| MGST2 | Osteoporosis, Postmenopausal | 6.07 |
| NOLC1 | Osteoporosis, Postmenopausal | 6.07 |
| PDLIM1 | Osteoporosis, Postmenopausal | 6.07 |
| BST2 | Osteoporosis, Postmenopausal | 6.06 |
| RNASE4 | Osteoporosis, Postmenopausal | 6.06 |
| TGIF1 | Osteoporosis, Postmenopausal | 6.06 |
| CHRM2 | Osteoporosis, Postmenopausal | 6.05 |
| CIRBP | Osteoporosis, Postmenopausal | 6.05 |
| FMO3 | Osteoporosis, Postmenopausal | 6.05 |
| YAP1 | Osteoporosis, Postmenopausal | 6.04 |
| ZFAND2A | Osteoporosis, Postmenopausal | 6.04 |
| CARS1 | Osteoporosis, Postmenopausal | 6.03 |
| NPC1 | Osteoporosis, Postmenopausal | 6.03 |
| ACADL | Osteoporosis, Postmenopausal | 6.02 |
| AKR1B8 | Osteoporosis, Postmenopausal | 6.02 |
| DBP | Osteoporosis, Postmenopausal | 6.02 |
| IL15 | Osteoporosis, Postmenopausal | 6.02 |
| NFIX | Osteoporosis, Postmenopausal | 6.02 |
| SHMT2 | Osteoporosis, Postmenopausal | 6.02 |
| C3-3 | Osteoporosis, Postmenopausal | 6.01 |
| ITGB4 | Osteoporosis, Postmenopausal | 6.01 |
| SIRT3 | Osteoporosis, Postmenopausal | 6 |
| USP18 | Osteoporosis, Postmenopausal | 6 |
| TUBB5 | Osteoporosis, Postmenopausal | 5.98 |
| UGT2B1 | Osteoporosis, Postmenopausal | 5.98 |
| CRY1 | Osteoporosis, Postmenopausal | 5.97 |
| RPSA | Osteoporosis, Postmenopausal | 5.97 |
| CIDEA | Osteoporosis, Postmenopausal | 5.96 |
| DIO3 | Osteoporosis, Postmenopausal | 5.96 |
| HSD17B3 | Osteoporosis, Postmenopausal | 5.96 |
| ALDH6A1 | Osteoporosis, Postmenopausal | 5.95 |
| GJB1 | Osteoporosis, Postmenopausal | 5.95 |
| MB | Osteoporosis, Postmenopausal | 5.95 |
| MUC5AC | Osteoporosis, Postmenopausal | 5.95 |
| OPRM1 | Osteoporosis, Postmenopausal | 5.95 |
| ATRX | Osteoporosis, Postmenopausal | 5.94 |
| CD274 | Osteoporosis, Postmenopausal | 5.94 |
| CFH | Osteoporosis, Postmenopausal | 5.94 |
| COL4A1 | Osteoporosis, Postmenopausal | 5.94 |
| GSDMD | Osteoporosis, Postmenopausal | 5.94 |
| ACTG1 | Osteoporosis, Postmenopausal | 5.93 |
| IDH3A | Osteoporosis, Postmenopausal | 5.93 |
| RAC2 | Osteoporosis, Postmenopausal | 5.93 |
| CBR3 | Osteoporosis, Postmenopausal | 5.92 |
| SLC7A1 | Osteoporosis, Postmenopausal | 5.92 |
| EPHA2 | Osteoporosis, Postmenopausal | 5.91 |
| MEF2C | Osteoporosis, Postmenopausal | 5.91 |
| S100A10 | Osteoporosis, Postmenopausal | 5.91 |
| KLF2 | Osteoporosis, Postmenopausal | 5.9 |
| RELN | Osteoporosis, Postmenopausal | 5.9 |
| SLC47A1 | Osteoporosis, Postmenopausal | 5.9 |
| VCAN | Osteoporosis, Postmenopausal | 5.9 |
| MIR3076 | Osteoporosis, Postmenopausal | 5.89 |
| PLG | Osteoporosis, Postmenopausal | 5.89 |
| CYP2B2 | Osteoporosis, Postmenopausal | 5.87 |
| DAB2 | Osteoporosis, Postmenopausal | 5.86 |
| AHCY | Osteoporosis, Postmenopausal | 5.85 |
| AKR7A3 | Osteoporosis, Postmenopausal | 5.85 |
| AXL | Osteoporosis, Postmenopausal | 5.85 |
| BRAF | Osteoporosis, Postmenopausal | 5.85 |
| TUBB4B | Osteoporosis, Postmenopausal | 5.85 |
| GLRX | Osteoporosis, Postmenopausal | 5.84 |
| LAMC2 | Osteoporosis, Postmenopausal | 5.84 |
| ZBTB16 | Osteoporosis, Postmenopausal | 5.84 |
| FOSB | Osteoporosis, Postmenopausal | 5.83 |
| MAT2A | Osteoporosis, Postmenopausal | 5.83 |
| MX1 | Osteoporosis, Postmenopausal | 5.82 |
| MHC1LDA | Osteoporosis, Postmenopausal | 5.8 |
| PONZR2 | Osteoporosis, Postmenopausal | 5.8 |
| SLC22A7 | Osteoporosis, Postmenopausal | 5.8 |
| TMEM79B | Osteoporosis, Postmenopausal | 5.8 |
| S100A11 | Osteoporosis, Postmenopausal | 5.79 |
| CST3 | Osteoporosis, Postmenopausal | 5.78 |
| HSD17B7 | Osteoporosis, Postmenopausal | 5.78 |
| DLG4 | Osteoporosis, Postmenopausal | 5.76 |
| MDH1 | Osteoporosis, Postmenopausal | 5.76 |
| MRC1 | Osteoporosis, Postmenopausal | 5.76 |
| ACOT1 | Osteoporosis, Postmenopausal | 5.75 |
| CNR2 | Osteoporosis, Postmenopausal | 5.74 |
| ETS1 | Osteoporosis, Postmenopausal | 5.74 |
| PIR | Osteoporosis, Postmenopausal | 5.74 |
| ACO1 | Osteoporosis, Postmenopausal | 5.73 |
| LPR | Osteoporosis, Postmenopausal | 5.73 |
| SDC4 | Osteoporosis, Postmenopausal | 5.73 |
| SORD | Osteoporosis, Postmenopausal | 5.73 |
| ACTN1 | Osteoporosis, Postmenopausal | 5.72 |
| FGF21 | Osteoporosis, Postmenopausal | 5.72 |
| GATA4 | Osteoporosis, Postmenopausal | 5.72 |
| GRN | Osteoporosis, Postmenopausal | 5.72 |
| PDE4B | Osteoporosis, Postmenopausal | 5.72 |
| PRKAA2 | Osteoporosis, Postmenopausal | 5.72 |
| PDIA4 | Osteoporosis, Postmenopausal | 5.69 |
| MTTP | Osteoporosis, Postmenopausal | 5.67 |
| PENK | Osteoporosis, Postmenopausal | 5.66 |
| LRP1 | Osteoporosis, Postmenopausal | 5.65 |
| EZR | Osteoporosis, Postmenopausal | 5.63 |
| ACAT2 | Osteoporosis, Postmenopausal | 5.61 |
| GCK | Osteoporosis, Postmenopausal | 5.6 |
| CCR2 | Osteoporosis, Postmenopausal | 5.57 |
| PDGFA | Osteoporosis, Postmenopausal | 5.55 |
| GRIN2A | Osteoporosis, Postmenopausal | 5.54 |
| SYP | Osteoporosis, Postmenopausal | 5.54 |
| CAC | Osteoporosis, Postmenopausal | 5.53 |
| MFN2 | Osteoporosis, Postmenopausal | 5.53 |
| CYP2B6B | Osteoporosis, Postmenopausal | 5.52 |
| MME | Osteoporosis, Postmenopausal | 5.5 |
| GCH1 | Osteoporosis, Postmenopausal | 5.49 |
| LPIN1 | Osteoporosis, Postmenopausal | 5.47 |
| A2M | Osteoporosis, Postmenopausal | 5.46 |
| EPAS1 | Osteoporosis, Postmenopausal | 5.46 |
| FADS2 | Osteoporosis, Postmenopausal | 5.45 |
| GHRHRL | Osteoporosis, Postmenopausal | 5.45 |
| HRH2B | Osteoporosis, Postmenopausal | 5.45 |
| NRN1LB | Osteoporosis, Postmenopausal | 5.45 |
| ODF2B | Osteoporosis, Postmenopausal | 5.45 |
| VDRALPHA | Osteoporosis, Postmenopausal | 5.44 |
| OAT | Osteoporosis, Postmenopausal | 5.42 |
| RUNX2A | Osteoporosis, Postmenopausal | 5.4 |
| PTGR1 | Osteoporosis, Postmenopausal | 5.39 |
| ACE2 | Osteoporosis, Postmenopausal | 5.38 |
| MAP2 | Osteoporosis, Postmenopausal | 5.37 |
| MBP | Osteoporosis, Postmenopausal | 5.37 |
| MIR679 | Osteoporosis, Postmenopausal | 5.34 |
| GRIN2B | Osteoporosis, Postmenopausal | 5.3 |
| NPY | Osteoporosis, Postmenopausal | 5.3 |
| PHGDH | Osteoporosis, Postmenopausal | 5.3 |
| SLC10A1 | Osteoporosis, Postmenopausal | 5.29 |
| NTRK2 | Osteoporosis, Postmenopausal | 5.26 |
| CD28L | Osteoporosis, Postmenopausal | 5.25 |
| FTR01 | Osteoporosis, Postmenopausal | 5.25 |
| GPSM1A | Osteoporosis, Postmenopausal | 5.25 |
| MTCL3A | Osteoporosis, Postmenopausal | 5.25 |
| PPP2R2BB | Osteoporosis, Postmenopausal | 5.25 |
| BTNL7-PS | Osteoporosis, Postmenopausal | 5.23 |
| CCPN-PS | Osteoporosis, Postmenopausal | 5.23 |
| ECRG4B | Osteoporosis, Postmenopausal | 5.23 |
| IGHV1-31 | Osteoporosis, Postmenopausal | 5.23 |
| IGHV4-2 | Osteoporosis, Postmenopausal | 5.23 |
| IGHV5-12-4 | Osteoporosis, Postmenopausal | 5.23 |
| IGKVK33-85 | Osteoporosis, Postmenopausal | 5.23 |
| OLFR257 | Osteoporosis, Postmenopausal | 5.23 |
| PIMR115 | Osteoporosis, Postmenopausal | 5.23 |
| PIMR191 | Osteoporosis, Postmenopausal | 5.23 |
| PMIS2 | Osteoporosis, Postmenopausal | 5.23 |
| TEX13C3 | Osteoporosis, Postmenopausal | 5.23 |
| TRDV2-2 | Osteoporosis, Postmenopausal | 5.23 |
| ZFA-PS | Osteoporosis, Postmenopausal | 5.23 |
| ZNF1043 | Osteoporosis, Postmenopausal | 5.23 |
| MIR3081 | Osteoporosis, Postmenopausal | 5.2 |
| BIB | Osteoporosis, Postmenopausal | 5.18 |
| SULT1ST6 | Osteoporosis, Postmenopausal | 5.18 |
| AKR1C2 | Osteoporosis, Postmenopausal | 5.17 |
| THRA.L | Osteoporosis, Postmenopausal | 5.17 |
| UXS1 | Osteoporosis, Postmenopausal | 5.16 |
| ICN | Osteoporosis, Postmenopausal | 5.15 |
| ABCB1A | Osteoporosis, Postmenopausal | 5.13 |
| CNR1 | Osteoporosis, Postmenopausal | 5.13 |
| PYCARD | Osteoporosis, Postmenopausal | 5.11 |
| ACY3.2 | Osteoporosis, Postmenopausal | 5.1 |
| ADNPB | Osteoporosis, Postmenopausal | 5.1 |
| GPR158A | Osteoporosis, Postmenopausal | 5.1 |
| LMBR1L | Osteoporosis, Postmenopausal | 5.1 |
| MTNR1AL | Osteoporosis, Postmenopausal | 5.1 |
| RN18S | Osteoporosis, Postmenopausal | 5.1 |
| SDK2B | Osteoporosis, Postmenopausal | 5.1 |
| SLC34A1A | Osteoporosis, Postmenopausal | 5.1 |
| VIML | Osteoporosis, Postmenopausal | 5.1 |
| WHRNB | Osteoporosis, Postmenopausal | 5.1 |
| DUX4L17 | Osteoporosis, Postmenopausal | 5.09 |
| DUX4L9 | Osteoporosis, Postmenopausal | 5.09 |
| MIR1A-1 | Osteoporosis, Postmenopausal | 5.09 |
| MIR5132 | Osteoporosis, Postmenopausal | 5.09 |
| PRAC2 | Osteoporosis, Postmenopausal | 5.09 |
| TRPL | Osteoporosis, Postmenopausal | 5.04 |
| ENTPD7 | Osteoporosis, Postmenopausal | 5 |
| GID8A | Osteoporosis, Postmenopausal | 4.99 |
| H19-ICR | Osteoporosis, Postmenopausal | 4.99 |
| HOXC6A | Osteoporosis, Postmenopausal | 4.99 |
| KCNG4A | Osteoporosis, Postmenopausal | 4.99 |
| MARK2A | Osteoporosis, Postmenopausal | 4.99 |
| MIR3074-1 | Osteoporosis, Postmenopausal | 4.99 |
| MIR3085 | Osteoporosis, Postmenopausal | 4.99 |
| MIR712 | Osteoporosis, Postmenopausal | 4.99 |
| MPZL2B | Osteoporosis, Postmenopausal | 4.99 |
| SLBP2 | Osteoporosis, Postmenopausal | 4.99 |
| ABU-2 | Osteoporosis, Postmenopausal | 4.98 |
| PQN-76 | Osteoporosis, Postmenopausal | 4.98 |
| PQN-91 | Osteoporosis, Postmenopausal | 4.98 |
| MIR195A | Osteoporosis, Postmenopausal | 4.92 |
| PRAC1 | Osteoporosis, Postmenopausal | 4.92 |
| CADM1A | Osteoporosis, Postmenopausal | 4.9 |
| FBXW11B | Osteoporosis, Postmenopausal | 4.9 |
| HNRNPH1L | Osteoporosis, Postmenopausal | 4.9 |
| NR1D2B | Osteoporosis, Postmenopausal | 4.9 |
| SHHA | Osteoporosis, Postmenopausal | 4.9 |
| ANKRD50L | Osteoporosis, Postmenopausal | 4.89 |
| CDK5R1B | Osteoporosis, Postmenopausal | 4.89 |
| CSMD2OS | Osteoporosis, Postmenopausal | 4.89 |
| DVR1 | Osteoporosis, Postmenopausal | 4.89 |
| FBXW22 | Osteoporosis, Postmenopausal | 4.89 |
| FBXW27 | Osteoporosis, Postmenopausal | 4.89 |
| IGKV2-116 | Osteoporosis, Postmenopausal | 4.89 |
| IGKV4-51 | Osteoporosis, Postmenopausal | 4.89 |
| IGKV8-16 | Osteoporosis, Postmenopausal | 4.89 |
| IGKV8-34 | Osteoporosis, Postmenopausal | 4.89 |
| KLRA20 | Osteoporosis, Postmenopausal | 4.89 |
| LYPD9 | Osteoporosis, Postmenopausal | 4.89 |
| MIR3576 | Osteoporosis, Postmenopausal | 4.89 |
| OR6Z3 | Osteoporosis, Postmenopausal | 4.89 |
| OR8B101D | Osteoporosis, Postmenopausal | 4.89 |
| OR8B51B | Osteoporosis, Postmenopausal | 4.89 |
| OR8C16C | Osteoporosis, Postmenopausal | 4.89 |
| PIMR213 | Osteoporosis, Postmenopausal | 4.89 |
| PIMR62 | Osteoporosis, Postmenopausal | 4.89 |
| PRDM16OS | Osteoporosis, Postmenopausal | 4.89 |
| RHOX7 | Osteoporosis, Postmenopausal | 4.89 |
| RPL23AP37 | Osteoporosis, Postmenopausal | 4.89 |
| RSF1OS2 | Osteoporosis, Postmenopausal | 4.89 |
| SERPINA3L-PS | Osteoporosis, Postmenopausal | 4.89 |
| SSRP1B | Osteoporosis, Postmenopausal | 4.89 |
| UBTD1B | Osteoporosis, Postmenopausal | 4.89 |
| VMN2R71 | Osteoporosis, Postmenopausal | 4.89 |
| KIF13A | Osteoporosis, Postmenopausal | 4.88 |
| F8A | Osteoporosis, Postmenopausal | 4.87 |
| FIT1 | Osteoporosis, Postmenopausal | 4.87 |
| IGKV13-84 | Osteoporosis, Postmenopausal | 4.87 |
| IGKV7-33 | Osteoporosis, Postmenopausal | 4.87 |
| MSTNA | Osteoporosis, Postmenopausal | 4.87 |
| MIR295 | Osteoporosis, Postmenopausal | 4.85 |
| MIR3472 | Osteoporosis, Postmenopausal | 4.85 |
| HTR4 | Osteoporosis, Postmenopausal | 4.83 |
| KIF20BA | Osteoporosis, Postmenopausal | 4.82 |
| LRATB.1 | Osteoporosis, Postmenopausal | 4.82 |
| OR1F45 | Osteoporosis, Postmenopausal | 4.82 |
| ANKRD34BA | Osteoporosis, Postmenopausal | 4.8 |
| ANXA14 | Osteoporosis, Postmenopausal | 4.8 |
| ATP5MJ-PS1 | Osteoporosis, Postmenopausal | 4.8 |
| BAZ2B-AS1 | Osteoporosis, Postmenopausal | 4.8 |
| C17H5ORF22 | Osteoporosis, Postmenopausal | 4.8 |
| C1H19ORF67 | Osteoporosis, Postmenopausal | 4.8 |
| C1H22ORF23 | Osteoporosis, Postmenopausal | 4.8 |
| C21HXORF21 | Osteoporosis, Postmenopausal | 4.8 |
| C24H6ORF58 | Osteoporosis, Postmenopausal | 4.8 |
| CAPSLB | Osteoporosis, Postmenopausal | 4.8 |
| CDCP1B | Osteoporosis, Postmenopausal | 4.8 |
| CTSSL | Osteoporosis, Postmenopausal | 4.8 |
| CYP2B6 | Osteoporosis, Postmenopausal | 4.8 |
| DRAXINA | Osteoporosis, Postmenopausal | 4.8 |
| DSG2L | Osteoporosis, Postmenopausal | 4.8 |
| FUT9C | Osteoporosis, Postmenopausal | 4.8 |
| GJB9B | Osteoporosis, Postmenopausal | 4.8 |
| HID1B | Osteoporosis, Postmenopausal | 4.8 |
| HMGA1P7 | Osteoporosis, Postmenopausal | 4.8 |
| HSF | Osteoporosis, Postmenopausal | 4.8 |
| IRX1A | Osteoporosis, Postmenopausal | 4.8 |
| JUPA | Osteoporosis, Postmenopausal | 4.8 |
| LHX6A | Osteoporosis, Postmenopausal | 4.8 |
| LINC00559 | Osteoporosis, Postmenopausal | 4.8 |
| LMX1BB | Osteoporosis, Postmenopausal | 4.8 |
| LRRC18B | Osteoporosis, Postmenopausal | 4.8 |
| MAGOH-DT | Osteoporosis, Postmenopausal | 4.8 |
| MIR4280 | Osteoporosis, Postmenopausal | 4.8 |
| MIR4701 | Osteoporosis, Postmenopausal | 4.8 |
| MIR519D | Osteoporosis, Postmenopausal | 4.8 |
| MIR520C | Osteoporosis, Postmenopausal | 4.8 |
| MIR6765 | Osteoporosis, Postmenopausal | 4.8 |
| MIR6820 | Osteoporosis, Postmenopausal | 4.8 |
| MIR7843 | Osteoporosis, Postmenopausal | 4.8 |
| NHR-119 | Osteoporosis, Postmenopausal | 4.8 |
| NHR-171 | Osteoporosis, Postmenopausal | 4.8 |
| NHR-194 | Osteoporosis, Postmenopausal | 4.8 |
| NHR-210 | Osteoporosis, Postmenopausal | 4.8 |
| NHR-246 | Osteoporosis, Postmenopausal | 4.8 |
| NHR-261 | Osteoporosis, Postmenopausal | 4.8 |
| OR8U3-PS | Osteoporosis, Postmenopausal | 4.8 |
| PGR-AS1 | Osteoporosis, Postmenopausal | 4.8 |
| PLXDC2B | Osteoporosis, Postmenopausal | 4.8 |
| PPT2B | Osteoporosis, Postmenopausal | 4.8 |
| RALBA | Osteoporosis, Postmenopausal | 4.8 |
| RASSF10B | Osteoporosis, Postmenopausal | 4.8 |
| RSL24D1P3 | Osteoporosis, Postmenopausal | 4.8 |
| SUV39H1-PS1 | Osteoporosis, Postmenopausal | 4.8 |
| UNC-55 | Osteoporosis, Postmenopausal | 4.8 |
| V165-D-J-C MU | Osteoporosis, Postmenopausal | 4.8 |
| VLC | Osteoporosis, Postmenopausal | 4.8 |
| ZDHHC12-DT | Osteoporosis, Postmenopausal | 4.8 |
| MIR2861 | Osteoporosis, Postmenopausal | 4.79 |
| SCD1 | Osteoporosis, Postmenopausal | 4.78 |
| OAMB | Osteoporosis, Postmenopausal | 4.76 |
| PTENB | Osteoporosis, Postmenopausal | 4.76 |
| U2AF2B | Osteoporosis, Postmenopausal | 4.76 |
| ARL8 | Osteoporosis, Postmenopausal | 4.75 |
| ELK2AP | Osteoporosis, Postmenopausal | 4.75 |
| RPL10P8 | Osteoporosis, Postmenopausal | 4.75 |
| MIR5099 | Osteoporosis, Postmenopausal | 4.74 |
| MIR5121 | Osteoporosis, Postmenopausal | 4.74 |
| CYLC2 | Osteoporosis, Postmenopausal | 4.7 |
| MSMP | Osteoporosis, Postmenopausal | 4.69 |
| ACSBG3 | Osteoporosis, Postmenopausal | 4.68 |
| ASTX6 | Osteoporosis, Postmenopausal | 4.68 |
| CBP | Osteoporosis, Postmenopausal | 4.68 |
| FBXW26 | Osteoporosis, Postmenopausal | 4.68 |
| IGHV14-4 | Osteoporosis, Postmenopausal | 4.68 |
| IGHV1-47 | Osteoporosis, Postmenopausal | 4.68 |
| IGHV1-56 | Osteoporosis, Postmenopausal | 4.68 |
| IGHV1-62-1 | Osteoporosis, Postmenopausal | 4.68 |
| IGHV1-66 | Osteoporosis, Postmenopausal | 4.68 |
| KCNMB4OS2 | Osteoporosis, Postmenopausal | 4.68 |
| KLRA18 | Osteoporosis, Postmenopausal | 4.68 |
| KRTAP4-16 | Osteoporosis, Postmenopausal | 4.68 |
| MAGEB5 | Osteoporosis, Postmenopausal | 4.68 |
| MIRLET7D | Osteoporosis, Postmenopausal | 4.68 |
| OR1F27 | Osteoporosis, Postmenopausal | 4.68 |
| OR2AA1 | Osteoporosis, Postmenopausal | 4.68 |
| OR8B43 | Osteoporosis, Postmenopausal | 4.68 |
| OR94A1 | Osteoporosis, Postmenopausal | 4.68 |
| PIMR188 | Osteoporosis, Postmenopausal | 4.68 |
| SCGB2B26 | Osteoporosis, Postmenopausal | 4.68 |
| ST3GAL3B | Osteoporosis, Postmenopausal | 4.68 |
| VMN1R53 | Osteoporosis, Postmenopausal | 4.68 |
| ZDHHC20B | Osteoporosis, Postmenopausal | 4.68 |
| ZNF1065 | Osteoporosis, Postmenopausal | 4.68 |
| DEFA2 | Osteoporosis, Postmenopausal | 4.67 |
| DOP-2 | Osteoporosis, Postmenopausal | 4.67 |
| UNC-43 | Osteoporosis, Postmenopausal | 4.67 |
| EWSR1B | Osteoporosis, Postmenopausal | 4.65 |
| FAM177B | Osteoporosis, Postmenopausal | 4.65 |
| GML2 | Osteoporosis, Postmenopausal | 4.65 |
| IGSF22 | Osteoporosis, Postmenopausal | 4.65 |
| ACT1 | Osteoporosis, Postmenopausal | 4.64 |
| ABU-3 | Osteoporosis, Postmenopausal | 4.63 |
| ABU-5 | Osteoporosis, Postmenopausal | 4.63 |
| AKTS1 | Osteoporosis, Postmenopausal | 4.63 |
| ATP6V0CP1 | Osteoporosis, Postmenopausal | 4.63 |
| CYP-13A6 | Osteoporosis, Postmenopausal | 4.63 |
| FTH1P19 | Osteoporosis, Postmenopausal | 4.63 |
| KCTD9P2 | Osteoporosis, Postmenopausal | 4.63 |
| LINC00315 | Osteoporosis, Postmenopausal | 4.63 |
| MIR105-1 | Osteoporosis, Postmenopausal | 4.63 |
| MIR3185 | Osteoporosis, Postmenopausal | 4.63 |
| MIR4433A | Osteoporosis, Postmenopausal | 4.63 |
| MIR569 | Osteoporosis, Postmenopausal | 4.63 |
| MIR657 | Osteoporosis, Postmenopausal | 4.63 |
| MIR7110 | Osteoporosis, Postmenopausal | 4.63 |
| OR7E19P | Osteoporosis, Postmenopausal | 4.63 |
| PQN-78 | Osteoporosis, Postmenopausal | 4.63 |
| PRX6A | Osteoporosis, Postmenopausal | 4.63 |
| PRX6B | Osteoporosis, Postmenopausal | 4.63 |
| RPL10P12 | Osteoporosis, Postmenopausal | 4.63 |
| RPL27P2 | Osteoporosis, Postmenopausal | 4.63 |
| SLPR | Osteoporosis, Postmenopausal | 4.63 |
| CLK2A | Osteoporosis, Postmenopausal | 4.6 |
| COX8H | Osteoporosis, Postmenopausal | 4.6 |
| UBTFL | Osteoporosis, Postmenopausal | 4.6 |
| MIR194-1 | Osteoporosis, Postmenopausal | 4.57 |
| BAI3 | Osteoporosis, Postmenopausal | 4.54 |
| C3H20ORF144 | Osteoporosis, Postmenopausal | 4.54 |
| C7H12ORF56 | Osteoporosis, Postmenopausal | 4.54 |
| CST | Osteoporosis, Postmenopausal | 4.54 |
| CYP19 | Osteoporosis, Postmenopausal | 4.54 |
| FAM228A | Osteoporosis, Postmenopausal | 4.54 |
| FOXD2OS | Osteoporosis, Postmenopausal | 4.54 |
| GTPBP1L | Osteoporosis, Postmenopausal | 4.54 |
| HEPN1 | Osteoporosis, Postmenopausal | 4.54 |
| HSTRPA | Osteoporosis, Postmenopausal | 4.54 |
| KLK1B11 | Osteoporosis, Postmenopausal | 4.54 |
| MIR492 | Osteoporosis, Postmenopausal | 4.54 |
| OOG1 | Osteoporosis, Postmenopausal | 4.54 |
| OR4F57 | Osteoporosis, Postmenopausal | 4.54 |
| OR5D18 | Osteoporosis, Postmenopausal | 4.54 |
| OR7R1 | Osteoporosis, Postmenopausal | 4.54 |
| OR8B43B | Osteoporosis, Postmenopausal | 4.54 |
| OR8C13 | Osteoporosis, Postmenopausal | 4.54 |
| PARGL | Osteoporosis, Postmenopausal | 4.54 |
| PIMR60 | Osteoporosis, Postmenopausal | 4.54 |
| RHOUB | Osteoporosis, Postmenopausal | 4.54 |
| RPZ5 | Osteoporosis, Postmenopausal | 4.54 |
| RUNX1-IT1 | Osteoporosis, Postmenopausal | 4.54 |
| SYNGR1B | Osteoporosis, Postmenopausal | 4.54 |
| TAS2R130 | Osteoporosis, Postmenopausal | 4.54 |
| TIEG3 | Osteoporosis, Postmenopausal | 4.54 |
| TMEM79A | Osteoporosis, Postmenopausal | 4.54 |
| VDRB | Osteoporosis, Postmenopausal | 4.54 |
| VIT-3 | Osteoporosis, Postmenopausal | 4.54 |
| VMN1R227 | Osteoporosis, Postmenopausal | 4.54 |
| VMN2R43 | Osteoporosis, Postmenopausal | 4.54 |
| VOM2R38 | Osteoporosis, Postmenopausal | 4.54 |
| WBSCR25 | Osteoporosis, Postmenopausal | 4.54 |
| ZFP974 | Osteoporosis, Postmenopausal | 4.54 |
| ZNF106A | Osteoporosis, Postmenopausal | 4.54 |
| ZP2.1 | Osteoporosis, Postmenopausal | 4.54 |
| ZP2.6 | Osteoporosis, Postmenopausal | 4.54 |
| CSTF2T | Osteoporosis, Postmenopausal | 4.53 |
| AIR | Osteoporosis, Postmenopausal | 4.52 |
| MOD2 | Osteoporosis, Postmenopausal | 4.52 |
| SQSTM | Osteoporosis, Postmenopausal | 4.52 |
| TARP | Osteoporosis, Postmenopausal | 4.52 |
| TUBBP5 | Osteoporosis, Postmenopausal | 4.52 |
| UBE2E1 | Osteoporosis, Postmenopausal | 4.52 |
| WNT2BB | Osteoporosis, Postmenopausal | 4.52 |
| LTB4R1 | Osteoporosis, Postmenopausal | 4.51 |
| NR1H5 | Osteoporosis, Postmenopausal | 4.51 |
| TAFAZZIN | Osteoporosis, Postmenopausal | 4.51 |
| CYP4A3 | Osteoporosis, Postmenopausal | 4.49 |
| C17ORF67 | Osteoporosis, Postmenopausal | 4.48 |
| TPTEP1 | Osteoporosis, Postmenopausal | 4.48 |
| FOXD4L3 | Osteoporosis, Postmenopausal | 4.47 |
| POTEH | Osteoporosis, Postmenopausal | 4.47 |
| SPANXN3 | Osteoporosis, Postmenopausal | 4.47 |
| TRIM48 | Osteoporosis, Postmenopausal | 4.47 |
| ADGRG7.1 | Osteoporosis, Postmenopausal | 4.45 |
| AMH.S | Osteoporosis, Postmenopausal | 4.45 |
| APODA.1 | Osteoporosis, Postmenopausal | 4.45 |
| AQP5-AS1 | Osteoporosis, Postmenopausal | 4.45 |
| ARA | Osteoporosis, Postmenopausal | 4.45 |
| ARID6 | Osteoporosis, Postmenopausal | 4.45 |
| BTBD11B | Osteoporosis, Postmenopausal | 4.45 |
| BTF3P12 | Osteoporosis, Postmenopausal | 4.45 |
| C1H4ORF33 | Osteoporosis, Postmenopausal | 4.45 |
| C1QTNF6B | Osteoporosis, Postmenopausal | 4.45 |
| C2H4ORF3 | Osteoporosis, Postmenopausal | 4.45 |
| C3H15ORF48 | Osteoporosis, Postmenopausal | 4.45 |
| CAPZA1-PS1 | Osteoporosis, Postmenopausal | 4.45 |
| CLMNB | Osteoporosis, Postmenopausal | 4.45 |
| CLMPB | Osteoporosis, Postmenopausal | 4.45 |
| CYSSL3 | Osteoporosis, Postmenopausal | 4.45 |
| DTX2-PS1 | Osteoporosis, Postmenopausal | 4.45 |
| DVL1B | Osteoporosis, Postmenopausal | 4.45 |
| ENO1-PS6 | Osteoporosis, Postmenopausal | 4.45 |
| ERB2 | Osteoporosis, Postmenopausal | 4.45 |
| FAM205A | Osteoporosis, Postmenopausal | 4.45 |
| FAM87A | Osteoporosis, Postmenopausal | 4.45 |
| FGF8B | Osteoporosis, Postmenopausal | 4.45 |
| FOXL2.S | Osteoporosis, Postmenopausal | 4.45 |
| GCSHL1 | Osteoporosis, Postmenopausal | 4.45 |
| GLIS2-AS1 | Osteoporosis, Postmenopausal | 4.45 |
| GRHL2A | Osteoporosis, Postmenopausal | 4.45 |
| HEPACAMA | Osteoporosis, Postmenopausal | 4.45 |
| HMGN2-PS4 | Osteoporosis, Postmenopausal | 4.45 |
| HOXB13A | Osteoporosis, Postmenopausal | 4.45 |
| HOXB9A | Osteoporosis, Postmenopausal | 4.45 |
| HYMAI | Osteoporosis, Postmenopausal | 4.45 |
| IGKV1OR15-118 | Osteoporosis, Postmenopausal | 4.45 |
| IKBE | Osteoporosis, Postmenopausal | 4.45 |
| JAM3A | Osteoporosis, Postmenopausal | 4.45 |
| K6PP | Osteoporosis, Postmenopausal | 4.45 |
| KISS1RB | Osteoporosis, Postmenopausal | 4.45 |
| KLC1B | Osteoporosis, Postmenopausal | 4.45 |
| LCDR | Osteoporosis, Postmenopausal | 4.45 |
| LINC00160 | Osteoporosis, Postmenopausal | 4.45 |
| LINC01523 | Osteoporosis, Postmenopausal | 4.45 |
| LINC02568 | Osteoporosis, Postmenopausal | 4.45 |
| MAPRE3B | Osteoporosis, Postmenopausal | 4.45 |
| MIR512 | Osteoporosis, Postmenopausal | 4.45 |
| MIR520H | Osteoporosis, Postmenopausal | 4.45 |
| MIR524 | Osteoporosis, Postmenopausal | 4.45 |
| MIR526B | Osteoporosis, Postmenopausal | 4.45 |
| MIR6753 | Osteoporosis, Postmenopausal | 4.45 |
| MIR6769A | Osteoporosis, Postmenopausal | 4.45 |
| MIR6779 | Osteoporosis, Postmenopausal | 4.45 |
| MIR6865 | Osteoporosis, Postmenopausal | 4.45 |
| MIR876 | Osteoporosis, Postmenopausal | 4.45 |
| MLLT4 | Osteoporosis, Postmenopausal | 4.45 |
| MMP25A | Osteoporosis, Postmenopausal | 4.45 |
| MYADMB | Osteoporosis, Postmenopausal | 4.45 |
| NHR-140 | Osteoporosis, Postmenopausal | 4.45 |
| NHR-47 | Osteoporosis, Postmenopausal | 4.45 |
| ODF2A | Osteoporosis, Postmenopausal | 4.45 |
| OR2Y3 | Osteoporosis, Postmenopausal | 4.45 |
| OR51B4B | Osteoporosis, Postmenopausal | 4.45 |
| OR6C213C | Osteoporosis, Postmenopausal | 4.45 |
| OSCP1B | Osteoporosis, Postmenopausal | 4.45 |
| OXCT1-AS1 | Osteoporosis, Postmenopausal | 4.45 |
| PANK3-PS1 | Osteoporosis, Postmenopausal | 4.45 |
| PIP5K1BA | Osteoporosis, Postmenopausal | 4.45 |
| PLA2G4AB | Osteoporosis, Postmenopausal | 4.45 |
| PLBD1A | Osteoporosis, Postmenopausal | 4.45 |
| PLD7 | Osteoporosis, Postmenopausal | 4.45 |
| PLEKHG5B | Osteoporosis, Postmenopausal | 4.45 |
| PRANCR | Osteoporosis, Postmenopausal | 4.45 |
| PTCHD3A | Osteoporosis, Postmenopausal | 4.45 |
| RANBP1L1 | Osteoporosis, Postmenopausal | 4.45 |
| RNF149L1 | Osteoporosis, Postmenopausal | 4.45 |
| RPL27L7 | Osteoporosis, Postmenopausal | 4.45 |
| RPS14-PS7 | Osteoporosis, Postmenopausal | 4.45 |
| RPS28-PS3 | Osteoporosis, Postmenopausal | 4.45 |
| S1PR3A | Osteoporosis, Postmenopausal | 4.45 |
| SERPINA14 | Osteoporosis, Postmenopausal | 4.45 |
| SLC8A4B | Osteoporosis, Postmenopausal | 4.45 |
| SMTNA | Osteoporosis, Postmenopausal | 4.45 |
| SNORD88C | Osteoporosis, Postmenopausal | 4.45 |
| SOCS9 | Osteoporosis, Postmenopausal | 4.45 |
| SOX8A | Osteoporosis, Postmenopausal | 4.45 |
| SOX9.L | Osteoporosis, Postmenopausal | 4.45 |
| SPIN2BL1 | Osteoporosis, Postmenopausal | 4.45 |
| SPINK1L | Osteoporosis, Postmenopausal | 4.45 |
| TMEM119A | Osteoporosis, Postmenopausal | 4.45 |
| TOMM5-PS1 | Osteoporosis, Postmenopausal | 4.45 |
| TPBG1B | Osteoporosis, Postmenopausal | 4.45 |
| TSHZ3B | Osteoporosis, Postmenopausal | 4.45 |
| VIG1 | Osteoporosis, Postmenopausal | 4.45 |
| YPEL2B | Osteoporosis, Postmenopausal | 4.45 |
| ZFHX3B | Osteoporosis, Postmenopausal | 4.45 |
| ZNF230-DT | Osteoporosis, Postmenopausal | 4.45 |
| ZPLD1A | Osteoporosis, Postmenopausal | 4.45 |
| CRY3B | Osteoporosis, Postmenopausal | 4.44 |
| ABHD17AP4 | Osteoporosis, Postmenopausal | 4.43 |
| CT47B1 | Osteoporosis, Postmenopausal | 4.43 |
| DLSTP1 | Osteoporosis, Postmenopausal | 4.43 |
| DPPA3P2 | Osteoporosis, Postmenopausal | 4.43 |
| FAM138D | Osteoporosis, Postmenopausal | 4.43 |
| FBXW14 | Osteoporosis, Postmenopausal | 4.43 |
| FER1L6-AS2 | Osteoporosis, Postmenopausal | 4.43 |
| FIP1L1A | Osteoporosis, Postmenopausal | 4.43 |
| GAGE12B | Osteoporosis, Postmenopausal | 4.43 |
| GZME | Osteoporosis, Postmenopausal | 4.43 |
| HOXAAS2 | Osteoporosis, Postmenopausal | 4.43 |
| KHDC1B | Osteoporosis, Postmenopausal | 4.43 |
| KIRREL3-AS3 | Osteoporosis, Postmenopausal | 4.43 |
| KLRA6 | Osteoporosis, Postmenopausal | 4.43 |
| KMT2BB | Osteoporosis, Postmenopausal | 4.43 |
| LINC01620 | Osteoporosis, Postmenopausal | 4.43 |
| MAFGA | Osteoporosis, Postmenopausal | 4.43 |
| MIR4689 | Osteoporosis, Postmenopausal | 4.43 |
| MIR507 | Osteoporosis, Postmenopausal | 4.43 |
| MIR518B | Osteoporosis, Postmenopausal | 4.43 |
| MIR519B | Osteoporosis, Postmenopausal | 4.43 |
| MIR525 | Osteoporosis, Postmenopausal | 4.43 |
| MIR548V | Osteoporosis, Postmenopausal | 4.43 |
| MIR631 | Osteoporosis, Postmenopausal | 4.43 |
| MIR639 | Osteoporosis, Postmenopausal | 4.43 |
| MIR645 | Osteoporosis, Postmenopausal | 4.43 |
| MIR664B | Osteoporosis, Postmenopausal | 4.43 |
| OR10D3 | Osteoporosis, Postmenopausal | 4.43 |
| OR2T2 | Osteoporosis, Postmenopausal | 4.43 |
| OR2T33 | Osteoporosis, Postmenopausal | 4.43 |
| OR5B101 | Osteoporosis, Postmenopausal | 4.43 |
| OR6J1 | Osteoporosis, Postmenopausal | 4.43 |
| OR7E125P | Osteoporosis, Postmenopausal | 4.43 |
| OR8A1B | Osteoporosis, Postmenopausal | 4.43 |
| PLECB | Osteoporosis, Postmenopausal | 4.43 |
| PPY2P | Osteoporosis, Postmenopausal | 4.43 |
| PRR23E | Osteoporosis, Postmenopausal | 4.43 |
| PSG27 | Osteoporosis, Postmenopausal | 4.43 |
| RPS2P35 | Osteoporosis, Postmenopausal | 4.43 |
| SCGB2B27 | Osteoporosis, Postmenopausal | 4.43 |
| SCN1BB | Osteoporosis, Postmenopausal | 4.43 |
| SNORD103C | Osteoporosis, Postmenopausal | 4.43 |
| SNORD14C | Osteoporosis, Postmenopausal | 4.43 |
| TRDV3 | Osteoporosis, Postmenopausal | 4.43 |
| VN1R10P | Osteoporosis, Postmenopausal | 4.43 |
| WBP11P1 | Osteoporosis, Postmenopausal | 4.43 |
| ZNF6 | Osteoporosis, Postmenopausal | 4.43 |
| ZNF740B | Osteoporosis, Postmenopausal | 4.43 |
| ZP3.2 | Osteoporosis, Postmenopausal | 4.43 |
| ANXA2R | Osteoporosis, Postmenopausal | 4.42 |
| CRIPTO | Osteoporosis, Postmenopausal | 4.41 |
| PLK | Osteoporosis, Postmenopausal | 4.41 |
| TEFA | Osteoporosis, Postmenopausal | 4.41 |
| DBNL | Osteoporosis, Postmenopausal | 4.4 |
| RFPL1 | Osteoporosis, Postmenopausal | 4.4 |
| MIR377 | Osteoporosis, Postmenopausal | 4.39 |
| MIR702 | Osteoporosis, Postmenopausal | 4.39 |
| STK4 | Osteoporosis, Postmenopausal | 4.39 |
| CRY1A | Osteoporosis, Postmenopausal | 4.38 |
| LY6B | Osteoporosis, Postmenopausal | 4.38 |
| USP50 | Osteoporosis, Postmenopausal | 4.38 |
| TMEM174 | Osteoporosis, Postmenopausal | 4.37 |
| UBA2 | Osteoporosis, Postmenopausal | 4.37 |
| SP2 | Osteoporosis, Postmenopausal | 4.36 |
| APRIN | Osteoporosis, Postmenopausal | 4.34 |
| HOXAAS3 | Osteoporosis, Postmenopausal | 4.34 |
| IGHV1-77 | Osteoporosis, Postmenopausal | 4.34 |
| IGKV1-132 | Osteoporosis, Postmenopausal | 4.34 |
| KRT8P12 | Osteoporosis, Postmenopausal | 4.34 |
| LINC00470 | Osteoporosis, Postmenopausal | 4.34 |
| NLRP4B | Osteoporosis, Postmenopausal | 4.34 |
| NXF5 | Osteoporosis, Postmenopausal | 4.34 |
| OR11I1 | Osteoporosis, Postmenopausal | 4.34 |
| OR4C108 | Osteoporosis, Postmenopausal | 4.34 |
| OR7D11B-PS1 | Osteoporosis, Postmenopausal | 4.34 |
| OR7G32 | Osteoporosis, Postmenopausal | 4.34 |
| POTEB | Osteoporosis, Postmenopausal | 4.34 |
| PRSS28 | Osteoporosis, Postmenopausal | 4.34 |
| PSG22 | Osteoporosis, Postmenopausal | 4.34 |
| RPTOROS | Osteoporosis, Postmenopausal | 4.34 |
| SLCO4A1-AS1 | Osteoporosis, Postmenopausal | 4.34 |
| SLCO6C1 | Osteoporosis, Postmenopausal | 4.34 |
| TMEM191B | Osteoporosis, Postmenopausal | 4.34 |
| TRIM35-34 | Osteoporosis, Postmenopausal | 4.34 |
| SLC2A11L | Osteoporosis, Postmenopausal | 4.33 |
| ZNF554 | Osteoporosis, Postmenopausal | 4.33 |
| CYCB | Osteoporosis, Postmenopausal | 4.32 |
| MIR4271 | Osteoporosis, Postmenopausal | 4.32 |
| OR9S15 | Osteoporosis, Postmenopausal | 4.32 |
| TCIMA | Osteoporosis, Postmenopausal | 4.3 |
| ABU-15 | Osteoporosis, Postmenopausal | 4.29 |
| ABU-7 | Osteoporosis, Postmenopausal | 4.29 |
| ADH5P4 | Osteoporosis, Postmenopausal | 4.29 |
| C15ORF32 | Osteoporosis, Postmenopausal | 4.29 |
| CTAGE7P | Osteoporosis, Postmenopausal | 4.29 |
| DEFB125 | Osteoporosis, Postmenopausal | 4.29 |
| DMBT1L1 | Osteoporosis, Postmenopausal | 4.29 |
| EGR | Osteoporosis, Postmenopausal | 4.29 |
| FAM47A | Osteoporosis, Postmenopausal | 4.29 |
| FAM87B | Osteoporosis, Postmenopausal | 4.29 |
| IL17R | Osteoporosis, Postmenopausal | 4.29 |
| IL21 | Osteoporosis, Postmenopausal | 4.29 |
| ILP5 | Osteoporosis, Postmenopausal | 4.29 |
| KRTAP12-4 | Osteoporosis, Postmenopausal | 4.29 |
| MAGEB6B | Osteoporosis, Postmenopausal | 4.29 |
| MIR103A1 | Osteoporosis, Postmenopausal | 4.29 |
| MIR1902 | Osteoporosis, Postmenopausal | 4.29 |
| MIR196A1 | Osteoporosis, Postmenopausal | 4.29 |
| MIR218 | Osteoporosis, Postmenopausal | 4.29 |
| MIR4674 | Osteoporosis, Postmenopausal | 4.29 |
| MIR4707 | Osteoporosis, Postmenopausal | 4.29 |
| MIR4741 | Osteoporosis, Postmenopausal | 4.29 |
| MIR584 | Osteoporosis, Postmenopausal | 4.29 |
| MIR888 | Osteoporosis, Postmenopausal | 4.29 |
| NOS | Osteoporosis, Postmenopausal | 4.29 |
| NRDC | Osteoporosis, Postmenopausal | 4.29 |
| OR1K1 | Osteoporosis, Postmenopausal | 4.29 |
| OR2G6 | Osteoporosis, Postmenopausal | 4.29 |
| OR2T35 | Osteoporosis, Postmenopausal | 4.29 |
| OR3A4P | Osteoporosis, Postmenopausal | 4.29 |
| OR4K17 | Osteoporosis, Postmenopausal | 4.29 |
| PAK6-AS1 | Osteoporosis, Postmenopausal | 4.29 |
| PRAMEF15 | Osteoporosis, Postmenopausal | 4.29 |
| PRAMEF19 | Osteoporosis, Postmenopausal | 4.29 |
| PRAMEF22 | Osteoporosis, Postmenopausal | 4.29 |
| PRAMEF7 | Osteoporosis, Postmenopausal | 4.29 |
| PUM2 | Osteoporosis, Postmenopausal | 4.29 |
| RGPD4 | Osteoporosis, Postmenopausal | 4.29 |
| RPL29P15 | Osteoporosis, Postmenopausal | 4.29 |
| SSX4 | Osteoporosis, Postmenopausal | 4.29 |
| TTTY13 | Osteoporosis, Postmenopausal | 4.29 |
| ULE-3 | Osteoporosis, Postmenopausal | 4.29 |
| ZBTB17 | Osteoporosis, Postmenopausal | 4.29 |
| MIR21A | Osteoporosis, Postmenopausal | 4.28 |
| CART1 | Osteoporosis, Postmenopausal | 4.26 |
| CNBPA | Osteoporosis, Postmenopausal | 4.26 |
| DMBX1A | Osteoporosis, Postmenopausal | 4.26 |
| EPB41B | Osteoporosis, Postmenopausal | 4.26 |
| GSDMC2 | Osteoporosis, Postmenopausal | 4.26 |
| IGKV14-111 | Osteoporosis, Postmenopausal | 4.26 |
| IGKV19-93 | Osteoporosis, Postmenopausal | 4.26 |
| KLRA13-PS | Osteoporosis, Postmenopausal | 4.26 |
| MAT2AL | Osteoporosis, Postmenopausal | 4.26 |
| MIR1188 | Osteoporosis, Postmenopausal | 4.26 |
| NGS | Osteoporosis, Postmenopausal | 4.26 |
| NLRP5-PS | Osteoporosis, Postmenopausal | 4.26 |
| OR11G1 | Osteoporosis, Postmenopausal | 4.26 |
| OR12K7 | Osteoporosis, Postmenopausal | 4.26 |
| OR4X13 | Osteoporosis, Postmenopausal | 4.26 |
| OR5AC19 | Osteoporosis, Postmenopausal | 4.26 |
| OR5T5 | Osteoporosis, Postmenopausal | 4.26 |
| PCDH1GB9 | Osteoporosis, Postmenopausal | 4.26 |
| PIRA1 | Osteoporosis, Postmenopausal | 4.26 |
| RPS27.2 | Osteoporosis, Postmenopausal | 4.26 |
| SIGLECF | Osteoporosis, Postmenopausal | 4.26 |
| SSU2 | Osteoporosis, Postmenopausal | 4.26 |
| SYNE2A | Osteoporosis, Postmenopausal | 4.26 |
| A2MP1 | Osteoporosis, Postmenopausal | 4.25 |
| ABHD8B | Osteoporosis, Postmenopausal | 4.25 |
| AK7B | Osteoporosis, Postmenopausal | 4.25 |
| ANKEF1A | Osteoporosis, Postmenopausal | 4.25 |
| ARK2CA | Osteoporosis, Postmenopausal | 4.25 |
| BGLAPL | Osteoporosis, Postmenopausal | 4.25 |
| BPIFA4P | Osteoporosis, Postmenopausal | 4.25 |
| BZW1-AS1 | Osteoporosis, Postmenopausal | 4.25 |
| C3-1 | Osteoporosis, Postmenopausal | 4.25 |
| C3-2 | Osteoporosis, Postmenopausal | 4.25 |
| CAMK1B | Osteoporosis, Postmenopausal | 4.25 |
| CART4 | Osteoporosis, Postmenopausal | 4.25 |
| CCSAPA | Osteoporosis, Postmenopausal | 4.25 |
| CHL1B | Osteoporosis, Postmenopausal | 4.25 |
| CKS1L | Osteoporosis, Postmenopausal | 4.25 |
| COL10A1B | Osteoporosis, Postmenopausal | 4.25 |
| COL18A1A | Osteoporosis, Postmenopausal | 4.25 |
| COL7A1L | Osteoporosis, Postmenopausal | 4.25 |
| CRACR2AA | Osteoporosis, Postmenopausal | 4.25 |
| CRB2B | Osteoporosis, Postmenopausal | 4.25 |
| CUZD1.2 | Osteoporosis, Postmenopausal | 4.25 |
| CXADRP1 | Osteoporosis, Postmenopausal | 4.25 |
| CYP17A1.L | Osteoporosis, Postmenopausal | 4.25 |
| CYP3A40 | Osteoporosis, Postmenopausal | 4.25 |
| DCAF13P3 | Osteoporosis, Postmenopausal | 4.25 |
| DDR2L | Osteoporosis, Postmenopausal | 4.25 |
| DENND5B-AS1 | Osteoporosis, Postmenopausal | 4.25 |
| DMRT1.S | Osteoporosis, Postmenopausal | 4.25 |
| ECE2B | Osteoporosis, Postmenopausal | 4.25 |
| EFR3BB | Osteoporosis, Postmenopausal | 4.25 |
| FAM167AB | Osteoporosis, Postmenopausal | 4.25 |
| GAJ | Osteoporosis, Postmenopausal | 4.25 |
| GATD3.L | Osteoporosis, Postmenopausal | 4.25 |
| GFRA4B | Osteoporosis, Postmenopausal | 4.25 |
| GGT5A | Osteoporosis, Postmenopausal | 4.25 |
| GIPC3 | Osteoporosis, Postmenopausal | 4.25 |
| GPER | Osteoporosis, Postmenopausal | 4.25 |
| GRM1B | Osteoporosis, Postmenopausal | 4.25 |
| GRM5B | Osteoporosis, Postmenopausal | 4.25 |
| GRM6A | Osteoporosis, Postmenopausal | 4.25 |
| HAAF | Osteoporosis, Postmenopausal | 4.25 |
| KIF5AB | Osteoporosis, Postmenopausal | 4.25 |
| KMT5AL3 | Osteoporosis, Postmenopausal | 4.25 |
| KRT16P6 | Osteoporosis, Postmenopausal | 4.25 |
| KRT98 | Osteoporosis, Postmenopausal | 4.25 |
| L1CAM-AS1 | Osteoporosis, Postmenopausal | 4.25 |
| LINC00304 | Osteoporosis, Postmenopausal | 4.25 |
| LINC00410 | Osteoporosis, Postmenopausal | 4.25 |
| LINC00994 | Osteoporosis, Postmenopausal | 4.25 |
| LINC01749 | Osteoporosis, Postmenopausal | 4.25 |
| LINC01951 | Osteoporosis, Postmenopausal | 4.25 |
| LINC02728 | Osteoporosis, Postmenopausal | 4.25 |
| MARVELD2B | Osteoporosis, Postmenopausal | 4.25 |
| MIR3679 | Osteoporosis, Postmenopausal | 4.25 |
| MIR518E | Osteoporosis, Postmenopausal | 4.25 |
| MIR519C | Osteoporosis, Postmenopausal | 4.25 |
| MIR579 | Osteoporosis, Postmenopausal | 4.25 |
| MIR6085 | Osteoporosis, Postmenopausal | 4.25 |
| MIR624 | Osteoporosis, Postmenopausal | 4.25 |
| MIR6795 | Osteoporosis, Postmenopausal | 4.25 |
| MTHFD2P7 | Osteoporosis, Postmenopausal | 4.25 |
| MYCLP1 | Osteoporosis, Postmenopausal | 4.25 |
| MYLKB | Osteoporosis, Postmenopausal | 4.25 |
| MYRIPB | Osteoporosis, Postmenopausal | 4.25 |
| NF2B | Osteoporosis, Postmenopausal | 4.25 |
| NHR-214 | Osteoporosis, Postmenopausal | 4.25 |
| NHR-59 | Osteoporosis, Postmenopausal | 4.25 |
| NMDAR1 | Osteoporosis, Postmenopausal | 4.25 |
| NOMO | Osteoporosis, Postmenopausal | 4.25 |
| OLR1500 | Osteoporosis, Postmenopausal | 4.25 |
| OR2N1G | Osteoporosis, Postmenopausal | 4.25 |
| OR51Q1D | Osteoporosis, Postmenopausal | 4.25 |
| OR51Q1E | Osteoporosis, Postmenopausal | 4.25 |
| PAG | Osteoporosis, Postmenopausal | 4.25 |
| PALMDB | Osteoporosis, Postmenopausal | 4.25 |
| PAPLNA | Osteoporosis, Postmenopausal | 4.25 |
| PAQR5B | Osteoporosis, Postmenopausal | 4.25 |
| PBOV1 | Osteoporosis, Postmenopausal | 4.25 |
| PILRB2L1 | Osteoporosis, Postmenopausal | 4.25 |
| PNPT1P1 | Osteoporosis, Postmenopausal | 4.25 |
| PORA | Osteoporosis, Postmenopausal | 4.25 |
| PTK7B | Osteoporosis, Postmenopausal | 4.25 |
| QUO | Osteoporosis, Postmenopausal | 4.25 |
| RC3H1B | Osteoporosis, Postmenopausal | 4.25 |
| RNU6-807P | Osteoporosis, Postmenopausal | 4.25 |
| RPL23AL1 | Osteoporosis, Postmenopausal | 4.25 |
| SALL1A | Osteoporosis, Postmenopausal | 4.25 |
| SEPSECS-AS1 | Osteoporosis, Postmenopausal | 4.25 |
| SEPTIN5A | Osteoporosis, Postmenopausal | 4.25 |
| SKINT8 | Osteoporosis, Postmenopausal | 4.25 |
| SLC13A5B | Osteoporosis, Postmenopausal | 4.25 |
| SNORD2 | Osteoporosis, Postmenopausal | 4.25 |
| SNORD88B | Osteoporosis, Postmenopausal | 4.25 |
| SOWAHCA | Osteoporosis, Postmenopausal | 4.25 |
| SPACA4L | Osteoporosis, Postmenopausal | 4.25 |
| THRIL | Osteoporosis, Postmenopausal | 4.25 |
| TLN2B | Osteoporosis, Postmenopausal | 4.25 |
| TRIM107 | Osteoporosis, Postmenopausal | 4.25 |
| TRPD52L3 | Osteoporosis, Postmenopausal | 4.25 |
| VOM2R-PS24 | Osteoporosis, Postmenopausal | 4.25 |
| ZNF213-AS1 | Osteoporosis, Postmenopausal | 4.25 |
| ZNF594-DT | Osteoporosis, Postmenopausal | 4.25 |
| CCR10 | Osteoporosis, Postmenopausal | 4.24 |
| CDC2L1 | Osteoporosis, Postmenopausal | 4.24 |
| GGTLC1 | Osteoporosis, Postmenopausal | 4.24 |
| GSTT1L | Osteoporosis, Postmenopausal | 4.24 |
| IIGP1 | Osteoporosis, Postmenopausal | 4.24 |
| LNK | Osteoporosis, Postmenopausal | 4.24 |
| PIT54 | Osteoporosis, Postmenopausal | 4.24 |
| SEK-1 | Osteoporosis, Postmenopausal | 4.24 |
| SSX2 | Osteoporosis, Postmenopausal | 4.24 |
| TMEM131 | Osteoporosis, Postmenopausal | 4.24 |
| TRPC7 | Osteoporosis, Postmenopausal | 4.23 |
| ARL13A | Osteoporosis, Postmenopausal | 4.21 |
| ADGRE3 | Osteoporosis, Postmenopausal | 4.2 |
| CD300LD3 | Osteoporosis, Postmenopausal | 4.2 |
| CIPCB | Osteoporosis, Postmenopausal | 4.2 |
| COPG2OS2 | Osteoporosis, Postmenopausal | 4.2 |
| D7ERTD443E | Osteoporosis, Postmenopausal | 4.2 |
| ELDR | Osteoporosis, Postmenopausal | 4.2 |
| FPR-RS6 | Osteoporosis, Postmenopausal | 4.2 |
| GGTLC2 | Osteoporosis, Postmenopausal | 4.2 |
| HDLBPA | Osteoporosis, Postmenopausal | 4.2 |
| HYAL6 | Osteoporosis, Postmenopausal | 4.2 |
| IGHV1-72 | Osteoporosis, Postmenopausal | 4.2 |
| IGKV4-63 | Osteoporosis, Postmenopausal | 4.2 |
| KRT37 | Osteoporosis, Postmenopausal | 4.2 |
| KRT87 | Osteoporosis, Postmenopausal | 4.2 |
| MELA | Osteoporosis, Postmenopausal | 4.2 |
| MIR540 | Osteoporosis, Postmenopausal | 4.2 |
| MIR673 | Osteoporosis, Postmenopausal | 4.2 |
| OLFR197 | Osteoporosis, Postmenopausal | 4.2 |
| OR1F26 | Osteoporosis, Postmenopausal | 4.2 |
| PRODHA | Osteoporosis, Postmenopausal | 4.2 |
| TCL1B5 | Osteoporosis, Postmenopausal | 4.2 |
| TOP2 | Osteoporosis, Postmenopausal | 4.2 |
| ZP2.2 | Osteoporosis, Postmenopausal | 4.2 |
| DAND5 | Osteoporosis, Postmenopausal | 4.19 |
| DNAJC5B | Osteoporosis, Postmenopausal | 4.19 |
| ABU-6 | Osteoporosis, Postmenopausal | 4.18 |
| C10ORF120 | Osteoporosis, Postmenopausal | 4.18 |
| C20ORF181 | Osteoporosis, Postmenopausal | 4.18 |
| CELF2-AS1 | Osteoporosis, Postmenopausal | 4.18 |
| CSNK2A3 | Osteoporosis, Postmenopausal | 4.18 |
| CYP2C1 | Osteoporosis, Postmenopausal | 4.18 |
| DKK1B | Osteoporosis, Postmenopausal | 4.18 |
| DULLARD | Osteoporosis, Postmenopausal | 4.18 |
| FAM83A-AS1 | Osteoporosis, Postmenopausal | 4.18 |
| GPR148 | Osteoporosis, Postmenopausal | 4.18 |
| ILP3 | Osteoporosis, Postmenopausal | 4.18 |
| KHDC3L | Osteoporosis, Postmenopausal | 4.18 |
| KRTAP10-1 | Osteoporosis, Postmenopausal | 4.18 |
| KRTAP10-12 | Osteoporosis, Postmenopausal | 4.18 |
| KRTAP10-2 | Osteoporosis, Postmenopausal | 4.18 |
| KRTAP4-11 | Osteoporosis, Postmenopausal | 4.18 |
| KRTAP4-5 | Osteoporosis, Postmenopausal | 4.18 |
| KRTAP5-11 | Osteoporosis, Postmenopausal | 4.18 |
| LBX2-AS1 | Osteoporosis, Postmenopausal | 4.18 |
| LCE5A | Osteoporosis, Postmenopausal | 4.18 |
| LINC00326 | Osteoporosis, Postmenopausal | 4.18 |
| LINC01565 | Osteoporosis, Postmenopausal | 4.18 |
| LINC01600 | Osteoporosis, Postmenopausal | 4.18 |
| MIR103A2 | Osteoporosis, Postmenopausal | 4.18 |
| MIR378H | Osteoporosis, Postmenopausal | 4.18 |
| MIR570 | Osteoporosis, Postmenopausal | 4.18 |
| MIR632 | Osteoporosis, Postmenopausal | 4.18 |
| MIR7-3 | Osteoporosis, Postmenopausal | 4.18 |
| OR2T3 | Osteoporosis, Postmenopausal | 4.18 |
| OR2T34 | Osteoporosis, Postmenopausal | 4.18 |
| OR52I2 | Osteoporosis, Postmenopausal | 4.18 |
| OR5H14 | Osteoporosis, Postmenopausal | 4.18 |
| OR5L1 | Osteoporosis, Postmenopausal | 4.18 |
| OR6C4 | Osteoporosis, Postmenopausal | 4.18 |
| OR6Q1 | Osteoporosis, Postmenopausal | 4.18 |
| POM121L10P | Osteoporosis, Postmenopausal | 4.18 |
| PPP1R3F | Osteoporosis, Postmenopausal | 4.18 |
| PQN-54 | Osteoporosis, Postmenopausal | 4.18 |
| PRB2 | Osteoporosis, Postmenopausal | 4.18 |
| PRR23B | Osteoporosis, Postmenopausal | 4.18 |
| PRSS42P | Osteoporosis, Postmenopausal | 4.18 |
| PRSS45P | Osteoporosis, Postmenopausal | 4.18 |
| PRY | Osteoporosis, Postmenopausal | 4.18 |
| REXO1L2P | Osteoporosis, Postmenopausal | 4.18 |
| RPL21P44 | Osteoporosis, Postmenopausal | 4.18 |
| RRN3P3 | Osteoporosis, Postmenopausal | 4.18 |
| SPATA31D1 | Osteoporosis, Postmenopausal | 4.18 |
| SSBP3-AS1 | Osteoporosis, Postmenopausal | 4.18 |
| SYNJ2BP-COX16 | Osteoporosis, Postmenopausal | 4.18 |
| TBC1D28 | Osteoporosis, Postmenopausal | 4.18 |
| TCRD | Osteoporosis, Postmenopausal | 4.18 |
| TPI | Osteoporosis, Postmenopausal | 4.18 |
| ARRDC3B | Osteoporosis, Postmenopausal | 4.17 |
| ANKRD36 | Osteoporosis, Postmenopausal | 4.16 |
| C6ORF118 | Osteoporosis, Postmenopausal | 4.16 |
| CCR8 | Osteoporosis, Postmenopausal | 4.16 |
| NOA1 | Osteoporosis, Postmenopausal | 4.16 |
| PBX2 | Osteoporosis, Postmenopausal | 4.16 |
| POU6F1 | Osteoporosis, Postmenopausal | 4.16 |
| P2RY4 | Osteoporosis, Postmenopausal | 4.15 |
| SH2D3A | Osteoporosis, Postmenopausal | 4.15 |
| ANTXR2A | Osteoporosis, Postmenopausal | 4.14 |
| CHI3L4 | Osteoporosis, Postmenopausal | 4.14 |
| CLK4A | Osteoporosis, Postmenopausal | 4.14 |
| CSTDC4 | Osteoporosis, Postmenopausal | 4.14 |
| IGKV3-12 | Osteoporosis, Postmenopausal | 4.14 |
| IGKV4-55 | Osteoporosis, Postmenopausal | 4.14 |
| IGKV5-43 | Osteoporosis, Postmenopausal | 4.14 |
| KLRA12 | Osteoporosis, Postmenopausal | 4.14 |
| KRTAP2-2 | Osteoporosis, Postmenopausal | 4.14 |
| OPRPN | Osteoporosis, Postmenopausal | 4.14 |
| OR51AA5 | Osteoporosis, Postmenopausal | 4.14 |
| PCDHGA3 | Osteoporosis, Postmenopausal | 4.14 |
| PYCR1B | Osteoporosis, Postmenopausal | 4.14 |
| RANGAP1B | Osteoporosis, Postmenopausal | 4.14 |
| SMR2 | Osteoporosis, Postmenopausal | 4.14 |
| UBAP2B | Osteoporosis, Postmenopausal | 4.14 |
| VEGP2 | Osteoporosis, Postmenopausal | 4.14 |
| VOM2R37 | Osteoporosis, Postmenopausal | 4.14 |
| ZFP943 | Osteoporosis, Postmenopausal | 4.14 |
| ZP3R | Osteoporosis, Postmenopausal | 4.14 |
| GUCY2D | Osteoporosis, Postmenopausal | 4.13 |
| SLC8A2 | Osteoporosis, Postmenopausal | 4.13 |
| ARIH1 | Osteoporosis, Postmenopausal | 4.12 |
| CACNA1F | Osteoporosis, Postmenopausal | 4.12 |
| LPAL2 | Osteoporosis, Postmenopausal | 4.12 |
| NOL1 | Osteoporosis, Postmenopausal | 4.12 |
| PER1B | Osteoporosis, Postmenopausal | 4.12 |
| POTEG | Osteoporosis, Postmenopausal | 4.12 |
| SNORA25 | Osteoporosis, Postmenopausal | 4.12 |
| TEPSIN | Osteoporosis, Postmenopausal | 4.12 |
| ZNF654 | Osteoporosis, Postmenopausal | 4.12 |
| ABO3 | Osteoporosis, Postmenopausal | 4.11 |
| ADGRF3B | Osteoporosis, Postmenopausal | 4.11 |
| ANTXR1C | Osteoporosis, Postmenopausal | 4.11 |
| APOA-I-1 | Osteoporosis, Postmenopausal | 4.11 |
| ARL3B | Osteoporosis, Postmenopausal | 4.11 |
| BCL11BB | Osteoporosis, Postmenopausal | 4.11 |
| BUB1B-PAK6 | Osteoporosis, Postmenopausal | 4.11 |
| C22ORF15 | Osteoporosis, Postmenopausal | 4.11 |
| CALML3-AS1 | Osteoporosis, Postmenopausal | 4.11 |
| CBX8B | Osteoporosis, Postmenopausal | 4.11 |
| CCKBRA | Osteoporosis, Postmenopausal | 4.11 |
| CCT6A-PS2 | Osteoporosis, Postmenopausal | 4.11 |
| CIPCA | Osteoporosis, Postmenopausal | 4.11 |
| COL18A1-AS1 | Osteoporosis, Postmenopausal | 4.11 |
| CPEB2-DT | Osteoporosis, Postmenopausal | 4.11 |
| CREB1A | Osteoporosis, Postmenopausal | 4.11 |
| CRIM1-DT | Osteoporosis, Postmenopausal | 4.11 |
| CYP19A1.L | Osteoporosis, Postmenopausal | 4.11 |
| CYP3C2 | Osteoporosis, Postmenopausal | 4.11 |
| CYP4F24P | Osteoporosis, Postmenopausal | 4.11 |
| DCHS1B | Osteoporosis, Postmenopausal | 4.11 |
| DGCR11 | Osteoporosis, Postmenopausal | 4.11 |
| EIF3LP3 | Osteoporosis, Postmenopausal | 4.11 |
| EPHA2B | Osteoporosis, Postmenopausal | 4.11 |
| EPS8L3A | Osteoporosis, Postmenopausal | 4.11 |
| FAM21EP | Osteoporosis, Postmenopausal | 4.11 |
| FBN2B | Osteoporosis, Postmenopausal | 4.11 |
| FOLR | Osteoporosis, Postmenopausal | 4.11 |
| FPGS | Osteoporosis, Postmenopausal | 4.11 |
| GDF10A | Osteoporosis, Postmenopausal | 4.11 |
| GLTSCR1L | Osteoporosis, Postmenopausal | 4.11 |
| GRIA1B | Osteoporosis, Postmenopausal | 4.11 |
| GUSBP5 | Osteoporosis, Postmenopausal | 4.11 |
| HAR1A | Osteoporosis, Postmenopausal | 4.11 |
| HDAC7A | Osteoporosis, Postmenopausal | 4.11 |
| HERC2P7 | Osteoporosis, Postmenopausal | 4.11 |
| HINT1-PS1 | Osteoporosis, Postmenopausal | 4.11 |
| IGKV28 | Osteoporosis, Postmenopausal | 4.11 |
| INDO | Osteoporosis, Postmenopausal | 4.11 |
| IRX5A | Osteoporosis, Postmenopausal | 4.11 |
| ITGB3A | Osteoporosis, Postmenopausal | 4.11 |
| JPH1A | Osteoporosis, Postmenopausal | 4.11 |
| LAMB2L | Osteoporosis, Postmenopausal | 4.11 |
| LILRP2 | Osteoporosis, Postmenopausal | 4.11 |
| LIMD1-AS1 | Osteoporosis, Postmenopausal | 4.11 |
| LINC00598 | Osteoporosis, Postmenopausal | 4.11 |
| LINC01016 | Osteoporosis, Postmenopausal | 4.11 |
| LINC01586 | Osteoporosis, Postmenopausal | 4.11 |
| LTB4R2A | Osteoporosis, Postmenopausal | 4.11 |
| MEGF6B | Osteoporosis, Postmenopausal | 4.11 |
| MIR3620 | Osteoporosis, Postmenopausal | 4.11 |
| MIR5008 | Osteoporosis, Postmenopausal | 4.11 |
| MIR502 | Osteoporosis, Postmenopausal | 4.11 |
| MIR519E | Osteoporosis, Postmenopausal | 4.11 |
| MIR548M | Osteoporosis, Postmenopausal | 4.11 |
| MIR618 | Osteoporosis, Postmenopausal | 4.11 |
| MIR6733 | Osteoporosis, Postmenopausal | 4.11 |
| MMP13B | Osteoporosis, Postmenopausal | 4.11 |
| MUT | Osteoporosis, Postmenopausal | 4.11 |
| NEO1A | Osteoporosis, Postmenopausal | 4.11 |
| OR2AG16 | Osteoporosis, Postmenopausal | 4.11 |
| OR51K7 | Osteoporosis, Postmenopausal | 4.11 |
| P4HA2-AS1 | Osteoporosis, Postmenopausal | 4.11 |
| PCYT1BB | Osteoporosis, Postmenopausal | 4.11 |
| PELI1B | Osteoporosis, Postmenopausal | 4.11 |
| PICK1 | Osteoporosis, Postmenopausal | 4.11 |
| PILRB-PS6 | Osteoporosis, Postmenopausal | 4.11 |
| PLCD4B | Osteoporosis, Postmenopausal | 4.11 |
| PPM1BB | Osteoporosis, Postmenopausal | 4.11 |
| PRAMEF11 | Osteoporosis, Postmenopausal | 4.11 |
| PRKAG3B | Osteoporosis, Postmenopausal | 4.11 |
| PROSC | Osteoporosis, Postmenopausal | 4.11 |
| PRPH2LB | Osteoporosis, Postmenopausal | 4.11 |
| PTP4A1L1 | Osteoporosis, Postmenopausal | 4.11 |
| RABL6B | Osteoporosis, Postmenopausal | 4.11 |
| RAD23AA | Osteoporosis, Postmenopausal | 4.11 |
| RGD2301395 | Osteoporosis, Postmenopausal | 4.11 |
| RPGRB | Osteoporosis, Postmenopausal | 4.11 |
| RPL13AP17 | Osteoporosis, Postmenopausal | 4.11 |
| RPL23P8 | Osteoporosis, Postmenopausal | 4.11 |
| RPL5P29 | Osteoporosis, Postmenopausal | 4.11 |
| RPSAP28 | Osteoporosis, Postmenopausal | 4.11 |
| RPSAP3 | Osteoporosis, Postmenopausal | 4.11 |
| RREB1B | Osteoporosis, Postmenopausal | 4.11 |
| S1PR5B | Osteoporosis, Postmenopausal | 4.11 |
| SCN1BA | Osteoporosis, Postmenopausal | 4.11 |
| SEMA3BL | Osteoporosis, Postmenopausal | 4.11 |
| SEPTIN4B | Osteoporosis, Postmenopausal | 4.11 |
| SERPINA3L | Osteoporosis, Postmenopausal | 4.11 |
| SLC17A7A | Osteoporosis, Postmenopausal | 4.11 |
| SLC22A13B | Osteoporosis, Postmenopausal | 4.11 |
| SNORD81 | Osteoporosis, Postmenopausal | 4.11 |
| SUMO1P1 | Osteoporosis, Postmenopausal | 4.11 |
| TGFB5 | Osteoporosis, Postmenopausal | 4.11 |
| TM6SF2A | Osteoporosis, Postmenopausal | 4.11 |
| TMEM106BA | Osteoporosis, Postmenopausal | 4.11 |
| TMTC2B | Osteoporosis, Postmenopausal | 4.11 |
| TNNI4B.3 | Osteoporosis, Postmenopausal | 4.11 |
| TSPAN3B | Osteoporosis, Postmenopausal | 4.11 |
| UBE2G1A | Osteoporosis, Postmenopausal | 4.11 |
| UGT5B2 | Osteoporosis, Postmenopausal | 4.11 |
| VGLL4L | Osteoporosis, Postmenopausal | 4.11 |
| VMN1R73 | Osteoporosis, Postmenopausal | 4.11 |
| VTGB1.L | Osteoporosis, Postmenopausal | 4.11 |
| GOT1L1 | Osteoporosis, Postmenopausal | 4.1 |
| GPAT2 | Osteoporosis, Postmenopausal | 4.1 |
| ABU-11 | Osteoporosis, Postmenopausal | 4.09 |
| CDRT15P3 | Osteoporosis, Postmenopausal | 4.09 |
| CLCA3P | Osteoporosis, Postmenopausal | 4.09 |
| CPS1-IT1 | Osteoporosis, Postmenopausal | 4.09 |
| DEFB108B | Osteoporosis, Postmenopausal | 4.09 |
| GIMAP2 | Osteoporosis, Postmenopausal | 4.09 |
| HRH4 | Osteoporosis, Postmenopausal | 4.09 |
| KIAA0087 | Osteoporosis, Postmenopausal | 4.09 |
| KRTAP10-4 | Osteoporosis, Postmenopausal | 4.09 |
| KRTAP12-3 | Osteoporosis, Postmenopausal | 4.09 |
| KRTAP13-3 | Osteoporosis, Postmenopausal | 4.09 |
| KRTAP13-4 | Osteoporosis, Postmenopausal | 4.09 |
| KRTAP20-4 | Osteoporosis, Postmenopausal | 4.09 |
| KRTAP5-10 | Osteoporosis, Postmenopausal | 4.09 |
| KRTAP9-8 | Osteoporosis, Postmenopausal | 4.09 |
| LGALS9B | Osteoporosis, Postmenopausal | 4.09 |
| LINC00523 | Osteoporosis, Postmenopausal | 4.09 |
| LINC01555 | Osteoporosis, Postmenopausal | 4.09 |
| LRRC53 | Osteoporosis, Postmenopausal | 4.09 |
| LYPLA2P1 | Osteoporosis, Postmenopausal | 4.09 |
| MIR1181 | Osteoporosis, Postmenopausal | 4.09 |
| MIR1231 | Osteoporosis, Postmenopausal | 4.09 |
| MIR135A | Osteoporosis, Postmenopausal | 4.09 |
| MIR1983 | Osteoporosis, Postmenopausal | 4.09 |
| MIR3175 | Osteoporosis, Postmenopausal | 4.09 |
| MIR323A | Osteoporosis, Postmenopausal | 4.09 |
| MIR352 | Osteoporosis, Postmenopausal | 4.09 |
| MIR3916 | Osteoporosis, Postmenopausal | 4.09 |
| MIR466D | Osteoporosis, Postmenopausal | 4.09 |
| MIR517A | Osteoporosis, Postmenopausal | 4.09 |
| MIR571 | Osteoporosis, Postmenopausal | 4.09 |
| MIR601 | Osteoporosis, Postmenopausal | 4.09 |
| MIR6789 | Osteoporosis, Postmenopausal | 4.09 |
| MIR6850 | Osteoporosis, Postmenopausal | 4.09 |
| MIR92A2 | Osteoporosis, Postmenopausal | 4.09 |
| MUP14 | Osteoporosis, Postmenopausal | 4.09 |
| OR10G7 | Osteoporosis, Postmenopausal | 4.09 |
| OR10H2 | Osteoporosis, Postmenopausal | 4.09 |
| OR10H5 | Osteoporosis, Postmenopausal | 4.09 |
| OR2M1P | Osteoporosis, Postmenopausal | 4.09 |
| OR2S2 | Osteoporosis, Postmenopausal | 4.09 |
| OR6K3 | Osteoporosis, Postmenopausal | 4.09 |
| PDL1 | Osteoporosis, Postmenopausal | 4.09 |
| PRAMEF20 | Osteoporosis, Postmenopausal | 4.09 |
| PRX6C | Osteoporosis, Postmenopausal | 4.09 |
| PTTG2 | Osteoporosis, Postmenopausal | 4.09 |
| PVALEF | Osteoporosis, Postmenopausal | 4.09 |
| RGPD1 | Osteoporosis, Postmenopausal | 4.09 |
| RNF5P1 | Osteoporosis, Postmenopausal | 4.09 |
| RTP5 | Osteoporosis, Postmenopausal | 4.09 |
| SNORA29 | Osteoporosis, Postmenopausal | 4.09 |
| SNORA38B | Osteoporosis, Postmenopausal | 4.09 |
| SNORA80A | Osteoporosis, Postmenopausal | 4.09 |
| SNORD32B | Osteoporosis, Postmenopausal | 4.09 |
| SNORD54 | Osteoporosis, Postmenopausal | 4.09 |
| SPATA8 | Osteoporosis, Postmenopausal | 4.09 |
| STARD4-AS1 | Osteoporosis, Postmenopausal | 4.09 |
| TAAR8 | Osteoporosis, Postmenopausal | 4.09 |
| TAK1 | Osteoporosis, Postmenopausal | 4.09 |
| TRPS1 | Osteoporosis, Postmenopausal | 4.09 |
| VN1R3 | Osteoporosis, Postmenopausal | 4.09 |
| D3ERTD751E | Osteoporosis, Postmenopausal | 4.08 |
| DEFA-RS4 | Osteoporosis, Postmenopausal | 4.08 |
| GJA6 | Osteoporosis, Postmenopausal | 4.08 |
| KHDC1A | Osteoporosis, Postmenopausal | 4.08 |
| LINC00899 | Osteoporosis, Postmenopausal | 4.08 |
| OR2O1 | Osteoporosis, Postmenopausal | 4.08 |
| PRL3C1 | Osteoporosis, Postmenopausal | 4.08 |
| TMEM210 | Osteoporosis, Postmenopausal | 4.08 |
| TMEM249 | Osteoporosis, Postmenopausal | 4.08 |
| USP2A | Osteoporosis, Postmenopausal | 4.08 |
| ZP3B | Osteoporosis, Postmenopausal | 4.08 |
| CNPPD1 | Osteoporosis, Postmenopausal | 4.06 |
| PRSS30P | Osteoporosis, Postmenopausal | 4.06 |
| BPIFB5 | Osteoporosis, Postmenopausal | 4.04 |
| GZMF | Osteoporosis, Postmenopausal | 4.04 |
| H4C2 | Osteoporosis, Postmenopausal | 4.04 |
| LY6G6F | Osteoporosis, Postmenopausal | 4.04 |
| MAPK12 | Osteoporosis, Postmenopausal | 4.04 |
| MYST2 | Osteoporosis, Postmenopausal | 4.04 |
| NRXN3A | Osteoporosis, Postmenopausal | 4.04 |
| NT5C2L1 | Osteoporosis, Postmenopausal | 4.04 |
| OR3A4 | Osteoporosis, Postmenopausal | 4.04 |
| OR7G29 | Osteoporosis, Postmenopausal | 4.04 |
| PDCL2 | Osteoporosis, Postmenopausal | 4.04 |
| SPEER4D | Osteoporosis, Postmenopausal | 4.04 |
| TCL1A | Osteoporosis, Postmenopausal | 4.04 |
| VEGF | Osteoporosis, Postmenopausal | 4.04 |
| AXDND1 | Osteoporosis, Postmenopausal | 4.03 |
| C1ORF54 | Osteoporosis, Postmenopausal | 4.03 |
| EMC7 | Osteoporosis, Postmenopausal | 4.03 |
| EP400P1 | Osteoporosis, Postmenopausal | 4.03 |
| GPBAR1 | Osteoporosis, Postmenopausal | 4.03 |
| NGB | Osteoporosis, Postmenopausal | 4.03 |
| AGE-1 | Osteoporosis, Postmenopausal | 4.02 |
| MIR216A | Osteoporosis, Postmenopausal | 4.02 |
| RNF41 | Osteoporosis, Postmenopausal | 4.02 |
| SNORA68 | Osteoporosis, Postmenopausal | 4.02 |
| ABU-8 | Osteoporosis, Postmenopausal | 4.01 |
| APOL5 | Osteoporosis, Postmenopausal | 4.01 |
| C11ORF16 | Osteoporosis, Postmenopausal | 4.01 |
| C16ORF92 | Osteoporosis, Postmenopausal | 4.01 |
| C19ORF67 | Osteoporosis, Postmenopausal | 4.01 |
| DDX53 | Osteoporosis, Postmenopausal | 4.01 |
| DEFB136 | Osteoporosis, Postmenopausal | 4.01 |
| DIAPH2-AS1 | Osteoporosis, Postmenopausal | 4.01 |
| DSTNP2 | Osteoporosis, Postmenopausal | 4.01 |
| DUXA | Osteoporosis, Postmenopausal | 4.01 |
| ELOA3BP | Osteoporosis, Postmenopausal | 4.01 |
| ELOA3P | Osteoporosis, Postmenopausal | 4.01 |
| FAM215A | Osteoporosis, Postmenopausal | 4.01 |
| H2BW1 | Osteoporosis, Postmenopausal | 4.01 |
| KRTAP4-12 | Osteoporosis, Postmenopausal | 4.01 |
| KRTAP4-4 | Osteoporosis, Postmenopausal | 4.01 |
| LINC00528 | Osteoporosis, Postmenopausal | 4.01 |
| MIR124-3 | Osteoporosis, Postmenopausal | 4.01 |
| MIR572 | Osteoporosis, Postmenopausal | 4.01 |
| MIR659 | Osteoporosis, Postmenopausal | 4.01 |
| MRGPRX4 | Osteoporosis, Postmenopausal | 4.01 |
| NFE4 | Osteoporosis, Postmenopausal | 4.01 |
| NLRP13 | Osteoporosis, Postmenopausal | 4.01 |
| NPBWR2 | Osteoporosis, Postmenopausal | 4.01 |
| NUTM2F | Osteoporosis, Postmenopausal | 4.01 |
| OR10K1 | Osteoporosis, Postmenopausal | 4.01 |
| OR1D4 | Osteoporosis, Postmenopausal | 4.01 |
| OR2A2 | Osteoporosis, Postmenopausal | 4.01 |
| OR2T11 | Osteoporosis, Postmenopausal | 4.01 |
| OR2Z1 | Osteoporosis, Postmenopausal | 4.01 |
| OR51V1 | Osteoporosis, Postmenopausal | 4.01 |
| OR52N1 | Osteoporosis, Postmenopausal | 4.01 |
| OR56A1 | Osteoporosis, Postmenopausal | 4.01 |
| OR5M10 | Osteoporosis, Postmenopausal | 4.01 |
| PIWIL3 | Osteoporosis, Postmenopausal | 4.01 |
| POM121L12 | Osteoporosis, Postmenopausal | 4.01 |
| RFPL4B | Osteoporosis, Postmenopausal | 4.01 |
| SAA | Osteoporosis, Postmenopausal | 4.01 |
| SCARNA16 | Osteoporosis, Postmenopausal | 4.01 |
| SLC35G6 | Osteoporosis, Postmenopausal | 4.01 |
| SMIM45 | Osteoporosis, Postmenopausal | 4.01 |
| SNORD38B | Osteoporosis, Postmenopausal | 4.01 |
| SNORD73A | Osteoporosis, Postmenopausal | 4.01 |
| SPANXA2-OT1 | Osteoporosis, Postmenopausal | 4.01 |
| SSX8P | Osteoporosis, Postmenopausal | 4.01 |
| TAS2R1 | Osteoporosis, Postmenopausal | 4.01 |
| TBC1D26 | Osteoporosis, Postmenopausal | 4.01 |
| TMEM132E-DT | Osteoporosis, Postmenopausal | 4.01 |
| VCY | Osteoporosis, Postmenopausal | 4.01 |
| ZNF718 | Osteoporosis, Postmenopausal | 4 |
| ACOT16 | Osteoporosis, Postmenopausal | 3.99 |
| ADD3B | Osteoporosis, Postmenopausal | 3.99 |
| ADORA2AB | Osteoporosis, Postmenopausal | 3.99 |
| AES | Osteoporosis, Postmenopausal | 3.99 |
| ALOX8 | Osteoporosis, Postmenopausal | 3.99 |
| ANKHA | Osteoporosis, Postmenopausal | 3.99 |
| APOL7B-PS1 | Osteoporosis, Postmenopausal | 3.99 |
| ARF3A | Osteoporosis, Postmenopausal | 3.99 |
| ATP5D | Osteoporosis, Postmenopausal | 3.99 |
| ATP6AP1-DT | Osteoporosis, Postmenopausal | 3.99 |
| BIC | Osteoporosis, Postmenopausal | 3.99 |
| BMB | Osteoporosis, Postmenopausal | 3.99 |
| C13H1ORF105 | Osteoporosis, Postmenopausal | 3.99 |
| C1ORF220 | Osteoporosis, Postmenopausal | 3.99 |
| CABP5A | Osteoporosis, Postmenopausal | 3.99 |
| CAPRIN1B | Osteoporosis, Postmenopausal | 3.99 |
| CCDC189 | Osteoporosis, Postmenopausal | 3.99 |
| CCL20B | Osteoporosis, Postmenopausal | 3.99 |
| CD248A | Osteoporosis, Postmenopausal | 3.99 |
| CDX1A | Osteoporosis, Postmenopausal | 3.99 |
| CEACAM15 | Osteoporosis, Postmenopausal | 3.99 |
| CFBL | Osteoporosis, Postmenopausal | 3.99 |
| CHMP5A | Osteoporosis, Postmenopausal | 3.99 |
| CLUHA | Osteoporosis, Postmenopausal | 3.99 |
| COL12A1B | Osteoporosis, Postmenopausal | 3.99 |
| COL28A2A | Osteoporosis, Postmenopausal | 3.99 |
| CPDA | Osteoporosis, Postmenopausal | 3.99 |
| CYP2P9 | Osteoporosis, Postmenopausal | 3.99 |
| DEFA4 | Osteoporosis, Postmenopausal | 3.99 |
| DLX4B | Osteoporosis, Postmenopausal | 3.99 |
| DLX6A | Osteoporosis, Postmenopausal | 3.99 |
| EB1 | Osteoporosis, Postmenopausal | 3.99 |
| ELNA | Osteoporosis, Postmenopausal | 3.99 |
| FAM111A-DT | Osteoporosis, Postmenopausal | 3.99 |
| FAM209 | Osteoporosis, Postmenopausal | 3.99 |
| FGF6A | Osteoporosis, Postmenopausal | 3.99 |
| FGF7P3 | Osteoporosis, Postmenopausal | 3.99 |
| FOXJ1A | Osteoporosis, Postmenopausal | 3.99 |
| FRG1HP | Osteoporosis, Postmenopausal | 3.99 |
| FT | Osteoporosis, Postmenopausal | 3.99 |
| GAPDHL10 | Osteoporosis, Postmenopausal | 3.99 |
| GATA6-AS1 | Osteoporosis, Postmenopausal | 3.99 |
| GHRLOS | Osteoporosis, Postmenopausal | 3.99 |
| GPR98 | Osteoporosis, Postmenopausal | 3.99 |
| GSTM.3 | Osteoporosis, Postmenopausal | 3.99 |
| GZMD | Osteoporosis, Postmenopausal | 3.99 |
| HOXB-AS1 | Osteoporosis, Postmenopausal | 3.99 |
| HOXC12A | Osteoporosis, Postmenopausal | 3.99 |
| HTR2CL1 | Osteoporosis, Postmenopausal | 3.99 |
| IGK-V1 | Osteoporosis, Postmenopausal | 3.99 |
| IGKV3-4 | Osteoporosis, Postmenopausal | 3.99 |
| IGKV4-86 | Osteoporosis, Postmenopausal | 3.99 |
| IGKV6-14 | Osteoporosis, Postmenopausal | 3.99 |
| IGKVL15 | Osteoporosis, Postmenopausal | 3.99 |
| IRX3A | Osteoporosis, Postmenopausal | 3.99 |
| KLK1C4 | Osteoporosis, Postmenopausal | 3.99 |
| KLK5L1 | Osteoporosis, Postmenopausal | 3.99 |
| KNG2L1 | Osteoporosis, Postmenopausal | 3.99 |
| LGALS8-AS1 | Osteoporosis, Postmenopausal | 3.99 |
| LINC01023 | Osteoporosis, Postmenopausal | 3.99 |
| LINC01954 | Osteoporosis, Postmenopausal | 3.99 |
| LINC01962 | Osteoporosis, Postmenopausal | 3.99 |
| LY6L | Osteoporosis, Postmenopausal | 3.99 |
| MALINC1 | Osteoporosis, Postmenopausal | 3.99 |
| MAPK12A | Osteoporosis, Postmenopausal | 3.99 |
| MAT2AL1 | Osteoporosis, Postmenopausal | 3.99 |
| MFAP1AL1 | Osteoporosis, Postmenopausal | 3.99 |
| MGST3L1 | Osteoporosis, Postmenopausal | 3.99 |
| MINPP1B | Osteoporosis, Postmenopausal | 3.99 |
| MIR448 | Osteoporosis, Postmenopausal | 3.99 |
| MIR4488 | Osteoporosis, Postmenopausal | 3.99 |
| MIR4664 | Osteoporosis, Postmenopausal | 3.99 |
| MIR4787 | Osteoporosis, Postmenopausal | 3.99 |
| MIR548B | Osteoporosis, Postmenopausal | 3.99 |
| MIR563 | Osteoporosis, Postmenopausal | 3.99 |
| MIR564 | Osteoporosis, Postmenopausal | 3.99 |
| MIR595 | Osteoporosis, Postmenopausal | 3.99 |
| MIR627 | Osteoporosis, Postmenopausal | 3.99 |
| MIR647 | Osteoporosis, Postmenopausal | 3.99 |
| MIR95 | Osteoporosis, Postmenopausal | 3.99 |
| MUP20 | Osteoporosis, Postmenopausal | 3.99 |
| NDST2A | Osteoporosis, Postmenopausal | 3.99 |
| NRP2A | Osteoporosis, Postmenopausal | 3.99 |
| OR10V1 | Osteoporosis, Postmenopausal | 3.99 |
| OR1F21 | Osteoporosis, Postmenopausal | 3.99 |
| OR51K1 | Osteoporosis, Postmenopausal | 3.99 |
| OR5T16 | Osteoporosis, Postmenopausal | 3.99 |
| OR9S14B | Osteoporosis, Postmenopausal | 3.99 |
| PATE4 | Osteoporosis, Postmenopausal | 3.99 |
| PCGEM1 | Osteoporosis, Postmenopausal | 3.99 |
| PDE6GB | Osteoporosis, Postmenopausal | 3.99 |
| PILRB2L3 | Osteoporosis, Postmenopausal | 3.99 |
| PKDCCB | Osteoporosis, Postmenopausal | 3.99 |
| PPP1R3AB | Osteoporosis, Postmenopausal | 3.99 |
| PR | Osteoporosis, Postmenopausal | 3.99 |
| PRKCBB | Osteoporosis, Postmenopausal | 3.99 |
| RNASE1L2 | Osteoporosis, Postmenopausal | 3.99 |
| RPL27AL2 | Osteoporosis, Postmenopausal | 3.99 |
| SEMA6BB | Osteoporosis, Postmenopausal | 3.99 |
| SERPINB1L1 | Osteoporosis, Postmenopausal | 3.99 |
| SIX7 | Osteoporosis, Postmenopausal | 3.99 |
| SLC12A5B | Osteoporosis, Postmenopausal | 3.99 |
| SNORA15 | Osteoporosis, Postmenopausal | 3.99 |
| SNORA81 | Osteoporosis, Postmenopausal | 3.99 |
| SNORD110 | Osteoporosis, Postmenopausal | 3.99 |
| SNORD76 | Osteoporosis, Postmenopausal | 3.99 |
| SPG1 | Osteoporosis, Postmenopausal | 3.99 |
| SRSF6B | Osteoporosis, Postmenopausal | 3.99 |
| TGFBR1A | Osteoporosis, Postmenopausal | 3.99 |
| TNK2A | Osteoporosis, Postmenopausal | 3.99 |
| TNNI1D | Osteoporosis, Postmenopausal | 3.99 |
| TRAF4A | Osteoporosis, Postmenopausal | 3.99 |
| TRBV14 | Osteoporosis, Postmenopausal | 3.99 |
| WFDC10A | Osteoporosis, Postmenopausal | 3.99 |
| ZCCHC11 | Osteoporosis, Postmenopausal | 3.99 |
| ZFP109 | Osteoporosis, Postmenopausal | 3.99 |
| ZFP534L2 | Osteoporosis, Postmenopausal | 3.99 |
| ZNF818P | Osteoporosis, Postmenopausal | 3.99 |
| LYSMD4 | Osteoporosis, Postmenopausal | 3.98 |
| SLC8A3 | Osteoporosis, Postmenopausal | 3.98 |
| CELF6 | Osteoporosis, Postmenopausal | 3.97 |
| DAT-1 | Osteoporosis, Postmenopausal | 3.97 |
| EQTN | Osteoporosis, Postmenopausal | 3.97 |
| HMOX | Osteoporosis, Postmenopausal | 3.97 |
| MIR298 | Osteoporosis, Postmenopausal | 3.97 |
| QNG1 | Osteoporosis, Postmenopausal | 3.97 |
| TRPM8 | Osteoporosis, Postmenopausal | 3.97 |
| ZNF565 | Osteoporosis, Postmenopausal | 3.97 |
| KCNN3 | Osteoporosis, Postmenopausal | 3.96 |
| ZNF473 | Osteoporosis, Postmenopausal | 3.96 |
| ATG4A | Osteoporosis, Postmenopausal | 3.95 |
| CCDC174 | Osteoporosis, Postmenopausal | 3.95 |
| CEACAM2 | Osteoporosis, Postmenopausal | 3.95 |
| CREMA | Osteoporosis, Postmenopausal | 3.95 |
| CYP19B | Osteoporosis, Postmenopausal | 3.95 |
| IGFL3 | Osteoporosis, Postmenopausal | 3.95 |
| IGKV1-135 | Osteoporosis, Postmenopausal | 3.95 |
| KLRA3 | Osteoporosis, Postmenopausal | 3.95 |
| KLRB1F | Osteoporosis, Postmenopausal | 3.95 |
| MRGPRB2 | Osteoporosis, Postmenopausal | 3.95 |
| OR2Y14 | Osteoporosis, Postmenopausal | 3.95 |
| RHOX8 | Osteoporosis, Postmenopausal | 3.95 |
| SERPINA3I | Osteoporosis, Postmenopausal | 3.95 |
| SERPINB1C | Osteoporosis, Postmenopausal | 3.95 |
| SPON1A | Osteoporosis, Postmenopausal | 3.95 |
| ZFP950 | Osteoporosis, Postmenopausal | 3.95 |
| ASCL5 | Osteoporosis, Postmenopausal | 3.94 |
| BPESC1 | Osteoporosis, Postmenopausal | 3.94 |
| CIR | Osteoporosis, Postmenopausal | 3.94 |
| CSNK1G2-AS1 | Osteoporosis, Postmenopausal | 3.94 |
| DEFB123 | Osteoporosis, Postmenopausal | 3.94 |
| DIP2C-AS1 | Osteoporosis, Postmenopausal | 3.94 |
| DMRTC1 | Osteoporosis, Postmenopausal | 3.94 |
| GSN-AS1 | Osteoporosis, Postmenopausal | 3.94 |
| HOXB13 | Osteoporosis, Postmenopausal | 3.94 |
| KRTAP10-10 | Osteoporosis, Postmenopausal | 3.94 |
| KRTAP10-6 | Osteoporosis, Postmenopausal | 3.94 |
| KRTAP10-8 | Osteoporosis, Postmenopausal | 3.94 |
| KRTAP4-8 | Osteoporosis, Postmenopausal | 3.94 |
| KRTAP4-9 | Osteoporosis, Postmenopausal | 3.94 |
| LINC00474 | Osteoporosis, Postmenopausal | 3.94 |
| MAS1L | Osteoporosis, Postmenopausal | 3.94 |
| MIRLET7C-1 | Osteoporosis, Postmenopausal | 3.94 |
| NKAIN3 | Osteoporosis, Postmenopausal | 3.94 |
| OR1I1 | Osteoporosis, Postmenopausal | 3.94 |
| OR5AR1 | Osteoporosis, Postmenopausal | 3.94 |
| OR5K2 | Osteoporosis, Postmenopausal | 3.94 |
| OR5T2 | Osteoporosis, Postmenopausal | 3.94 |
| OVOS2 | Osteoporosis, Postmenopausal | 3.94 |
| RAB40A | Osteoporosis, Postmenopausal | 3.94 |
| RNF113B | Osteoporosis, Postmenopausal | 3.94 |
| SIR-2.1 | Osteoporosis, Postmenopausal | 3.94 |
| SLC35G5 | Osteoporosis, Postmenopausal | 3.94 |
| SNORA72 | Osteoporosis, Postmenopausal | 3.94 |
| SNORD38A | Osteoporosis, Postmenopausal | 3.94 |
| SNORD4A | Osteoporosis, Postmenopausal | 3.94 |
| SNORD61 | Osteoporosis, Postmenopausal | 3.94 |
| SPATA31E1 | Osteoporosis, Postmenopausal | 3.94 |
| TBC1D29P | Osteoporosis, Postmenopausal | 3.94 |
| AP4M1 | Osteoporosis, Postmenopausal | 3.93 |
| DDX26B | Osteoporosis, Postmenopausal | 3.93 |
| FAM209A | Osteoporosis, Postmenopausal | 3.93 |
| MATCAP1 | Osteoporosis, Postmenopausal | 3.93 |
| NEXN-AS1 | Osteoporosis, Postmenopausal | 3.93 |
| RFPL3S | Osteoporosis, Postmenopausal | 3.93 |
| WASH2P | Osteoporosis, Postmenopausal | 3.93 |
| ABHD16B | Osteoporosis, Postmenopausal | 3.92 |
| BHLHA9 | Osteoporosis, Postmenopausal | 3.92 |
| BPIFA2 | Osteoporosis, Postmenopausal | 3.92 |
| CLEC4B1 | Osteoporosis, Postmenopausal | 3.92 |
| EAR10 | Osteoporosis, Postmenopausal | 3.92 |
| EMC3 | Osteoporosis, Postmenopausal | 3.92 |
| FPR3 | Osteoporosis, Postmenopausal | 3.92 |
| HIST2H2AA2 | Osteoporosis, Postmenopausal | 3.92 |
| HSP90AB2P | Osteoporosis, Postmenopausal | 3.92 |
| IGHV1-64 | Osteoporosis, Postmenopausal | 3.92 |
| IGKV1-133 | Osteoporosis, Postmenopausal | 3.92 |
| JDP2B | Osteoporosis, Postmenopausal | 3.92 |
| NXPE5 | Osteoporosis, Postmenopausal | 3.92 |
| OR2H1 | Osteoporosis, Postmenopausal | 3.92 |
| PTGIR | Osteoporosis, Postmenopausal | 3.92 |
| STM | Osteoporosis, Postmenopausal | 3.92 |
| TXNIPB | Osteoporosis, Postmenopausal | 3.92 |
| MRRF | Osteoporosis, Postmenopausal | 3.91 |
| ABCA15P | Osteoporosis, Postmenopausal | 3.9 |
| ABCC6A | Osteoporosis, Postmenopausal | 3.9 |
| ACOD1LB.L | Osteoporosis, Postmenopausal | 3.9 |
| ANKRD20A1 | Osteoporosis, Postmenopausal | 3.9 |
| ASAP2B | Osteoporosis, Postmenopausal | 3.9 |
| ASB14B | Osteoporosis, Postmenopausal | 3.9 |
| ATOH1A | Osteoporosis, Postmenopausal | 3.9 |
| BUB1BA | Osteoporosis, Postmenopausal | 3.9 |
| C17ORF75 | Osteoporosis, Postmenopausal | 3.9 |
| CAB39L1 | Osteoporosis, Postmenopausal | 3.9 |
| CCRN4L | Osteoporosis, Postmenopausal | 3.9 |
| CGM4 | Osteoporosis, Postmenopausal | 3.9 |
| CH25HL1.1 | Osteoporosis, Postmenopausal | 3.9 |
| CHRNA2B | Osteoporosis, Postmenopausal | 3.9 |
| CLCA5 | Osteoporosis, Postmenopausal | 3.9 |
| CLOCKA | Osteoporosis, Postmenopausal | 3.9 |
| CPLX4A | Osteoporosis, Postmenopausal | 3.9 |
| CREB3L3A | Osteoporosis, Postmenopausal | 3.9 |
| CSDC2A | Osteoporosis, Postmenopausal | 3.9 |
| CYP2P7 | Osteoporosis, Postmenopausal | 3.9 |
| DNM3OS | Osteoporosis, Postmenopausal | 3.9 |
| ENTPD5A | Osteoporosis, Postmenopausal | 3.9 |
| EVPLB | Osteoporosis, Postmenopausal | 3.9 |
| EZRB | Osteoporosis, Postmenopausal | 3.9 |
| FAAH2A | Osteoporosis, Postmenopausal | 3.9 |
| FABP1A | Osteoporosis, Postmenopausal | 3.9 |
| FAM60A | Osteoporosis, Postmenopausal | 3.9 |
| FNDC3BA | Osteoporosis, Postmenopausal | 3.9 |
| GHR1 | Osteoporosis, Postmenopausal | 3.9 |
| GNRH-R2 | Osteoporosis, Postmenopausal | 3.9 |
| GRM1A | Osteoporosis, Postmenopausal | 3.9 |
| H2BC3 | Osteoporosis, Postmenopausal | 3.9 |
| H2BW2 | Osteoporosis, Postmenopausal | 3.9 |
| HMGB2L2 | Osteoporosis, Postmenopausal | 3.9 |
| IDH1-AS1 | Osteoporosis, Postmenopausal | 3.9 |
| JAG1B | Osteoporosis, Postmenopausal | 3.9 |
| KISS1RA | Osteoporosis, Postmenopausal | 3.9 |
| KISS2 | Osteoporosis, Postmenopausal | 3.9 |
| KLK1B1 | Osteoporosis, Postmenopausal | 3.9 |
| KRTAP16-5 | Osteoporosis, Postmenopausal | 3.9 |
| LDB2A | Osteoporosis, Postmenopausal | 3.9 |
| LHX8A | Osteoporosis, Postmenopausal | 3.9 |
| LINC00513 | Osteoporosis, Postmenopausal | 3.9 |
| LINC00689 | Osteoporosis, Postmenopausal | 3.9 |
| LY49I7 | Osteoporosis, Postmenopausal | 3.9 |
| LY49S3 | Osteoporosis, Postmenopausal | 3.9 |
| MIR1229 | Osteoporosis, Postmenopausal | 3.9 |
| MIR26A2 | Osteoporosis, Postmenopausal | 3.9 |
| MIR3178 | Osteoporosis, Postmenopausal | 3.9 |
| MIR323B | Osteoporosis, Postmenopausal | 3.9 |
| MIR3936 | Osteoporosis, Postmenopausal | 3.9 |
| MIR4530 | Osteoporosis, Postmenopausal | 3.9 |
| MIR520B | Osteoporosis, Postmenopausal | 3.9 |
| MIRLET7A2 | Osteoporosis, Postmenopausal | 3.9 |
| MMP15A | Osteoporosis, Postmenopausal | 3.9 |
| MYB-AS1 | Osteoporosis, Postmenopausal | 3.9 |
| MYCLB | Osteoporosis, Postmenopausal | 3.9 |
| MYO6A | Osteoporosis, Postmenopausal | 3.9 |
| NBPF4 | Osteoporosis, Postmenopausal | 3.9 |
| OLR35 | Osteoporosis, Postmenopausal | 3.9 |
| OR52E15C | Osteoporosis, Postmenopausal | 3.9 |
| OR52X1 | Osteoporosis, Postmenopausal | 3.9 |
| PANE1 | Osteoporosis, Postmenopausal | 3.9 |
| PKMB | Osteoporosis, Postmenopausal | 3.9 |
| PLECA | Osteoporosis, Postmenopausal | 3.9 |
| PNP6 | Osteoporosis, Postmenopausal | 3.9 |
| PPP1R14AA | Osteoporosis, Postmenopausal | 3.9 |
| RBM39A | Osteoporosis, Postmenopausal | 3.9 |
| RPSAP31 | Osteoporosis, Postmenopausal | 3.9 |
| RT1-CE6 | Osteoporosis, Postmenopausal | 3.9 |
| RT1-DB2 | Osteoporosis, Postmenopausal | 3.9 |
| SEMA3FA | Osteoporosis, Postmenopausal | 3.9 |
| SNORD27 | Osteoporosis, Postmenopausal | 3.9 |
| SNORD45A | Osteoporosis, Postmenopausal | 3.9 |
| SNORD77 | Osteoporosis, Postmenopausal | 3.9 |
| SNORD78 | Osteoporosis, Postmenopausal | 3.9 |
| SNORD80 | Osteoporosis, Postmenopausal | 3.9 |
| SYN2B | Osteoporosis, Postmenopausal | 3.9 |
| TAAR3 | Osteoporosis, Postmenopausal | 3.9 |
| TCEB2 | Osteoporosis, Postmenopausal | 3.9 |
| THBS1B | Osteoporosis, Postmenopausal | 3.9 |
| THBS4B | Osteoporosis, Postmenopausal | 3.9 |
| TJP1A | Osteoporosis, Postmenopausal | 3.9 |
| TMCC1B | Osteoporosis, Postmenopausal | 3.9 |
| TRGC2 | Osteoporosis, Postmenopausal | 3.9 |
| UGT5A5 | Osteoporosis, Postmenopausal | 3.9 |
| UNGA | Osteoporosis, Postmenopausal | 3.9 |
| VASH1-AS1 | Osteoporosis, Postmenopausal | 3.9 |
| WNT7AA | Osteoporosis, Postmenopausal | 3.9 |
| ZBED10P | Osteoporosis, Postmenopausal | 3.9 |
| CACNA2D3 | Osteoporosis, Postmenopausal | 3.89 |
| ANKRD30A | Osteoporosis, Postmenopausal | 3.88 |
| APOL10B | Osteoporosis, Postmenopausal | 3.88 |
| AQP10 | Osteoporosis, Postmenopausal | 3.88 |
| C11ORF42 | Osteoporosis, Postmenopausal | 3.88 |
| CCND2A | Osteoporosis, Postmenopausal | 3.88 |
| CD209G | Osteoporosis, Postmenopausal | 3.88 |
| CRYBB1L1 | Osteoporosis, Postmenopausal | 3.88 |
| CSAG2 | Osteoporosis, Postmenopausal | 3.88 |
| CT55 | Osteoporosis, Postmenopausal | 3.88 |
| DEFB113 | Osteoporosis, Postmenopausal | 3.88 |
| DEFB41 | Osteoporosis, Postmenopausal | 3.88 |
| DLEU2L | Osteoporosis, Postmenopausal | 3.88 |
| DSCR10 | Osteoporosis, Postmenopausal | 3.88 |
| ERCC6L2-AS1 | Osteoporosis, Postmenopausal | 3.88 |
| EWSAT1 | Osteoporosis, Postmenopausal | 3.88 |
| FOXO3B | Osteoporosis, Postmenopausal | 3.88 |
| GAS-1 | Osteoporosis, Postmenopausal | 3.88 |
| GPD1A | Osteoporosis, Postmenopausal | 3.88 |
| GPR142 | Osteoporosis, Postmenopausal | 3.88 |
| HERC2P4 | Osteoporosis, Postmenopausal | 3.88 |
| HGC6.3 | Osteoporosis, Postmenopausal | 3.88 |
| KLRA23 | Osteoporosis, Postmenopausal | 3.88 |
| KRTAP10-11 | Osteoporosis, Postmenopausal | 3.88 |
| KRTAP19-3 | Osteoporosis, Postmenopausal | 3.88 |
| KRTAP4-2 | Osteoporosis, Postmenopausal | 3.88 |
| KRTAP9-3 | Osteoporosis, Postmenopausal | 3.88 |
| LCE2A | Osteoporosis, Postmenopausal | 3.88 |
| LCE4A | Osteoporosis, Postmenopausal | 3.88 |
| LINC00242 | Osteoporosis, Postmenopausal | 3.88 |
| LINC00319 | Osteoporosis, Postmenopausal | 3.88 |
| LINC00574 | Osteoporosis, Postmenopausal | 3.88 |
| LINC00671 | Osteoporosis, Postmenopausal | 3.88 |
| LINC01121 | Osteoporosis, Postmenopausal | 3.88 |
| LINCR | Osteoporosis, Postmenopausal | 3.88 |
| MCPT10 | Osteoporosis, Postmenopausal | 3.88 |
| MIR3613 | Osteoporosis, Postmenopausal | 3.88 |
| MIR5189 | Osteoporosis, Postmenopausal | 3.88 |
| MIR600HG | Osteoporosis, Postmenopausal | 3.88 |
| MIR770 | Osteoporosis, Postmenopausal | 3.88 |
| MYCS | Osteoporosis, Postmenopausal | 3.88 |
| NBPF15 | Osteoporosis, Postmenopausal | 3.88 |
| NUTM2A | Osteoporosis, Postmenopausal | 3.88 |
| OR1A2 | Osteoporosis, Postmenopausal | 3.88 |
| OR2T6 | Osteoporosis, Postmenopausal | 3.88 |
| OR5M11 | Osteoporosis, Postmenopausal | 3.88 |
| OR9A4 | Osteoporosis, Postmenopausal | 3.88 |
| OTOL1 | Osteoporosis, Postmenopausal | 3.88 |
| PAGE5 | Osteoporosis, Postmenopausal | 3.88 |
| PDZPH1 | Osteoporosis, Postmenopausal | 3.88 |
| PRAMEF12 | Osteoporosis, Postmenopausal | 3.88 |
| PRSS21 | Osteoporosis, Postmenopausal | 3.88 |
| PRSS58 | Osteoporosis, Postmenopausal | 3.88 |
| PYY2 | Osteoporosis, Postmenopausal | 3.88 |
| REXO1L1P | Osteoporosis, Postmenopausal | 3.88 |
| SCARNA12 | Osteoporosis, Postmenopausal | 3.88 |
| SCARNA9L | Osteoporosis, Postmenopausal | 3.88 |
| SNORA38 | Osteoporosis, Postmenopausal | 3.88 |
| SNORD101 | Osteoporosis, Postmenopausal | 3.88 |
| SNORD50A | Osteoporosis, Postmenopausal | 3.88 |
| SNORD56 | Osteoporosis, Postmenopausal | 3.88 |
| TESCL | Osteoporosis, Postmenopausal | 3.88 |
| TEX28 | Osteoporosis, Postmenopausal | 3.88 |
| TEX44 | Osteoporosis, Postmenopausal | 3.88 |
| THEM7 | Osteoporosis, Postmenopausal | 3.88 |
| TP53TG5 | Osteoporosis, Postmenopausal | 3.88 |
| ZFP984 | Osteoporosis, Postmenopausal | 3.88 |
| ZCCHC8 | Osteoporosis, Postmenopausal | 3.87 |
| ASB16-AS1 | Osteoporosis, Postmenopausal | 3.86 |
| ATG13 | Osteoporosis, Postmenopausal | 3.86 |
| DBH-AS1 | Osteoporosis, Postmenopausal | 3.86 |
| DEFA1 | Osteoporosis, Postmenopausal | 3.86 |
| FAM209B | Osteoporosis, Postmenopausal | 3.86 |
| FBXL21P | Osteoporosis, Postmenopausal | 3.86 |
| GML | Osteoporosis, Postmenopausal | 3.86 |
| GRK6 | Osteoporosis, Postmenopausal | 3.86 |
| PCSK7 | Osteoporosis, Postmenopausal | 3.86 |
| UBE2D2A | Osteoporosis, Postmenopausal | 3.86 |
| C8ORF34 | Osteoporosis, Postmenopausal | 3.85 |
| CLN8 | Osteoporosis, Postmenopausal | 3.85 |
| KLK1B8 | Osteoporosis, Postmenopausal | 3.85 |
| KRT26 | Osteoporosis, Postmenopausal | 3.85 |
| PCDHGA5 | Osteoporosis, Postmenopausal | 3.85 |
| PRSS29 | Osteoporosis, Postmenopausal | 3.85 |
| RBM46 | Osteoporosis, Postmenopausal | 3.85 |
| SERPINA3A | Osteoporosis, Postmenopausal | 3.85 |
| STFA1 | Osteoporosis, Postmenopausal | 3.85 |
| TRIML1 | Osteoporosis, Postmenopausal | 3.85 |
| ZFP941 | Osteoporosis, Postmenopausal | 3.85 |
| ZNF674 | Osteoporosis, Postmenopausal | 3.85 |
| ZNF695 | Osteoporosis, Postmenopausal | 3.85 |
| FKBP7 | Osteoporosis, Postmenopausal | 3.84 |
| ISL2 | Osteoporosis, Postmenopausal | 3.84 |
| USP19 | Osteoporosis, Postmenopausal | 3.84 |
| ADAMTS15A | Osteoporosis, Postmenopausal | 3.83 |
| ADGRF2P | Osteoporosis, Postmenopausal | 3.83 |
| AGO3B | Osteoporosis, Postmenopausal | 3.83 |
| ALDH9A1B | Osteoporosis, Postmenopausal | 3.83 |
| ALG1L1P | Osteoporosis, Postmenopausal | 3.83 |
| AMY2 | Osteoporosis, Postmenopausal | 3.83 |
| ANGPTL7 | Osteoporosis, Postmenopausal | 3.83 |
| APOBEC2A | Osteoporosis, Postmenopausal | 3.83 |
| AQP12A | Osteoporosis, Postmenopausal | 3.83 |
| ARNTL2 | Osteoporosis, Postmenopausal | 3.83 |
| C11ORF91 | Osteoporosis, Postmenopausal | 3.83 |
| C1QTNF8 | Osteoporosis, Postmenopausal | 3.83 |
| C1S-PS1 | Osteoporosis, Postmenopausal | 3.83 |
| C2CD4D-AS1 | Osteoporosis, Postmenopausal | 3.83 |
| C2H5ORF34 | Osteoporosis, Postmenopausal | 3.83 |
| C2ORF66 | Osteoporosis, Postmenopausal | 3.83 |
| C8ORF74 | Osteoporosis, Postmenopausal | 3.83 |
| C9ORF78 | Osteoporosis, Postmenopausal | 3.83 |
| CATSPERG2 | Osteoporosis, Postmenopausal | 3.83 |
| CCDC182 | Osteoporosis, Postmenopausal | 3.83 |
| CCT8L1 | Osteoporosis, Postmenopausal | 3.83 |
| COL12A1A | Osteoporosis, Postmenopausal | 3.83 |
| CSAG1 | Osteoporosis, Postmenopausal | 3.83 |
| CSAP1 | Osteoporosis, Postmenopausal | 3.83 |
| CTAG1A | Osteoporosis, Postmenopausal | 3.83 |
| DUSP21 | Osteoporosis, Postmenopausal | 3.83 |
| ECEL1P2 | Osteoporosis, Postmenopausal | 3.83 |
| EFCAB1 | Osteoporosis, Postmenopausal | 3.83 |
| EMILIN1A | Osteoporosis, Postmenopausal | 3.83 |
| ERBB2IP | Osteoporosis, Postmenopausal | 3.83 |
| F13A1A.1 | Osteoporosis, Postmenopausal | 3.83 |
| FAM182A | Osteoporosis, Postmenopausal | 3.83 |
| FAM228B | Osteoporosis, Postmenopausal | 3.83 |
| FAM86JP | Osteoporosis, Postmenopausal | 3.83 |
| FHL2A | Osteoporosis, Postmenopausal | 3.83 |
| FRMPD2 | Osteoporosis, Postmenopausal | 3.83 |
| GPR125 | Osteoporosis, Postmenopausal | 3.83 |
| GRIA1A | Osteoporosis, Postmenopausal | 3.83 |
| GRIA2B | Osteoporosis, Postmenopausal | 3.83 |
| GSTS2 | Osteoporosis, Postmenopausal | 3.83 |
| H4F3 | Osteoporosis, Postmenopausal | 3.83 |
| HN1 | Osteoporosis, Postmenopausal | 3.83 |
| HPXB | Osteoporosis, Postmenopausal | 3.83 |
| HSP90B2P | Osteoporosis, Postmenopausal | 3.83 |
| IGFBP5A | Osteoporosis, Postmenopausal | 3.83 |
| IGLJ3 | Osteoporosis, Postmenopausal | 3.83 |
| ING5A | Osteoporosis, Postmenopausal | 3.83 |
| IRBP | Osteoporosis, Postmenopausal | 3.83 |
| KDM4A-AS1 | Osteoporosis, Postmenopausal | 3.83 |
| KHDC1L | Osteoporosis, Postmenopausal | 3.83 |
| KLF5A | Osteoporosis, Postmenopausal | 3.83 |
| KLK1C6 | Osteoporosis, Postmenopausal | 3.83 |
| KPLCE | Osteoporosis, Postmenopausal | 3.83 |
| KRTAP20-2 | Osteoporosis, Postmenopausal | 3.83 |
| KRTAP4-1 | Osteoporosis, Postmenopausal | 3.83 |
| KRTAP4-3 | Osteoporosis, Postmenopausal | 3.83 |
| KRTAP5-1 | Osteoporosis, Postmenopausal | 3.83 |
| KRTAP7-1 | Osteoporosis, Postmenopausal | 3.83 |
| LINC00863 | Osteoporosis, Postmenopausal | 3.83 |
| LINC01508 | Osteoporosis, Postmenopausal | 3.83 |
| LY49I9 | Osteoporosis, Postmenopausal | 3.83 |
| LY49S6 | Osteoporosis, Postmenopausal | 3.83 |
| LY49SI1 | Osteoporosis, Postmenopausal | 3.83 |
| MACO1A | Osteoporosis, Postmenopausal | 3.83 |
| MIR1226 | Osteoporosis, Postmenopausal | 3.83 |
| MIR1910 | Osteoporosis, Postmenopausal | 3.83 |
| MIR4324 | Osteoporosis, Postmenopausal | 3.83 |
| MIR4508 | Osteoporosis, Postmenopausal | 3.83 |
| MIR612 | Osteoporosis, Postmenopausal | 3.83 |
| MIR656 | Osteoporosis, Postmenopausal | 3.83 |
| MIR9-1HG | Osteoporosis, Postmenopausal | 3.83 |
| MMP23A | Osteoporosis, Postmenopausal | 3.83 |
| MSTO2P | Osteoporosis, Postmenopausal | 3.83 |
| MTMR2 | Osteoporosis, Postmenopausal | 3.83 |
| NPAP1 | Osteoporosis, Postmenopausal | 3.83 |
| OPN4.1 | Osteoporosis, Postmenopausal | 3.83 |
| OR4D11 | Osteoporosis, Postmenopausal | 3.83 |
| OR51F2 | Osteoporosis, Postmenopausal | 3.83 |
| OR5W8 | Osteoporosis, Postmenopausal | 3.83 |
| OR6C69 | Osteoporosis, Postmenopausal | 3.83 |
| PAPLNB | Osteoporosis, Postmenopausal | 3.83 |
| PBX3B | Osteoporosis, Postmenopausal | 3.83 |
| PEX39 | Osteoporosis, Postmenopausal | 3.83 |
| PGAM1A | Osteoporosis, Postmenopausal | 3.83 |
| PNMA6A | Osteoporosis, Postmenopausal | 3.83 |
| PPP4R3C | Osteoporosis, Postmenopausal | 3.83 |
| RGD1559575 | Osteoporosis, Postmenopausal | 3.83 |
| RNA18SN4 | Osteoporosis, Postmenopausal | 3.83 |
| RNA28SN4 | Osteoporosis, Postmenopausal | 3.83 |
| ROCK2A | Osteoporosis, Postmenopausal | 3.83 |
| RPS2-PS10 | Osteoporosis, Postmenopausal | 3.83 |
| RPSAP12 | Osteoporosis, Postmenopausal | 3.83 |
| RT1-CE11 | Osteoporosis, Postmenopausal | 3.83 |
| SEPT5-GP1BB | Osteoporosis, Postmenopausal | 3.83 |
| SNORA4 | Osteoporosis, Postmenopausal | 3.83 |
| SNORA8 | Osteoporosis, Postmenopausal | 3.83 |
| SNORD116-21 | Osteoporosis, Postmenopausal | 3.83 |
| SNORD57 | Osteoporosis, Postmenopausal | 3.83 |
| SNORD59B | Osteoporosis, Postmenopausal | 3.83 |
| SNORD79 | Osteoporosis, Postmenopausal | 3.83 |
| SNORD87 | Osteoporosis, Postmenopausal | 3.83 |
| SNX10B | Osteoporosis, Postmenopausal | 3.83 |
| TAX1BP1B | Osteoporosis, Postmenopausal | 3.83 |
| TBC1D3G | Osteoporosis, Postmenopausal | 3.83 |
| TCAM1P | Osteoporosis, Postmenopausal | 3.83 |
| TCP11X2 | Osteoporosis, Postmenopausal | 3.83 |
| TDRD10 | Osteoporosis, Postmenopausal | 3.83 |
| TDRG1 | Osteoporosis, Postmenopausal | 3.83 |
| TEX55 | Osteoporosis, Postmenopausal | 3.83 |
| TIPARP-AS1 | Osteoporosis, Postmenopausal | 3.83 |
| TRAM2-AS1 | Osteoporosis, Postmenopausal | 3.83 |
| TTYH3B | Osteoporosis, Postmenopausal | 3.83 |
| UCKL1-AS1 | Osteoporosis, Postmenopausal | 3.83 |
| UGT2B9*2 | Osteoporosis, Postmenopausal | 3.83 |
| UGT5E1 | Osteoporosis, Postmenopausal | 3.83 |
| UPF3A | Osteoporosis, Postmenopausal | 3.83 |
| USMG5 | Osteoporosis, Postmenopausal | 3.83 |
| USP27X-DT | Osteoporosis, Postmenopausal | 3.83 |
| VOM2R16 | Osteoporosis, Postmenopausal | 3.83 |
| VOM2R73 | Osteoporosis, Postmenopausal | 3.83 |
| ZNF107 | Osteoporosis, Postmenopausal | 3.83 |
| ZNF454 | Osteoporosis, Postmenopausal | 3.83 |
| ZNF577 | Osteoporosis, Postmenopausal | 3.83 |
| ZNF835 | Osteoporosis, Postmenopausal | 3.83 |
| CES1A | Osteoporosis, Postmenopausal | 3.82 |
| GALNTL5 | Osteoporosis, Postmenopausal | 3.82 |
| H2AP | Osteoporosis, Postmenopausal | 3.82 |
| HORMAD2 | Osteoporosis, Postmenopausal | 3.82 |
| KPRP | Osteoporosis, Postmenopausal | 3.82 |
| KRTAP3-2 | Osteoporosis, Postmenopausal | 3.82 |
| MCU | Osteoporosis, Postmenopausal | 3.82 |
| PLA2 | Osteoporosis, Postmenopausal | 3.82 |
| PRL3A1 | Osteoporosis, Postmenopausal | 3.82 |
| RBMXL2 | Osteoporosis, Postmenopausal | 3.82 |
| TRIM30B | Osteoporosis, Postmenopausal | 3.82 |
| MARCHF11 | Osteoporosis, Postmenopausal | 3.81 |
| TASP1 | Osteoporosis, Postmenopausal | 3.81 |
| TMEM267 | Osteoporosis, Postmenopausal | 3.81 |
| ZMYM5 | Osteoporosis, Postmenopausal | 3.81 |
| C19ORF18 | Osteoporosis, Postmenopausal | 3.8 |
| C19ORF25 | Osteoporosis, Postmenopausal | 3.8 |
| CATSPER3 | Osteoporosis, Postmenopausal | 3.8 |
| DAF-2 | Osteoporosis, Postmenopausal | 3.8 |
| MIR33 | Osteoporosis, Postmenopausal | 3.8 |
| RPL32P3 | Osteoporosis, Postmenopausal | 3.8 |
| TAOK2 | Osteoporosis, Postmenopausal | 3.8 |
| TOP6BL | Osteoporosis, Postmenopausal | 3.8 |
| ZNF789 | Osteoporosis, Postmenopausal | 3.8 |
| CHI3L3 | Osteoporosis, Postmenopausal | 3.79 |
| GSTT4 | Osteoporosis, Postmenopausal | 3.79 |
| HRASLS | Osteoporosis, Postmenopausal | 3.79 |
| IFNL1 | Osteoporosis, Postmenopausal | 3.79 |
| LRRC63 | Osteoporosis, Postmenopausal | 3.79 |
| MBD3L2 | Osteoporosis, Postmenopausal | 3.79 |
| MIR665 | Osteoporosis, Postmenopausal | 3.79 |
| PTPN4 | Osteoporosis, Postmenopausal | 3.79 |
| BTNL3 | Osteoporosis, Postmenopausal | 3.78 |
| C12ORF50 | Osteoporosis, Postmenopausal | 3.78 |
| CCDC24 | Osteoporosis, Postmenopausal | 3.78 |
| CYLC1 | Osteoporosis, Postmenopausal | 3.78 |
| DIRC1 | Osteoporosis, Postmenopausal | 3.78 |
| DUX4 | Osteoporosis, Postmenopausal | 3.78 |
| HRH3 | Osteoporosis, Postmenopausal | 3.78 |
| INOS | Osteoporosis, Postmenopausal | 3.78 |
| KCNQ1DN | Osteoporosis, Postmenopausal | 3.78 |
| KRTAP12-2 | Osteoporosis, Postmenopausal | 3.78 |
| LCE2C | Osteoporosis, Postmenopausal | 3.78 |
| LINC00477 | Osteoporosis, Postmenopausal | 3.78 |
| LINC00652 | Osteoporosis, Postmenopausal | 3.78 |
| LINC01558 | Osteoporosis, Postmenopausal | 3.78 |
| MIR1-1HG | Osteoporosis, Postmenopausal | 3.78 |
| MIR26A1 | Osteoporosis, Postmenopausal | 3.78 |
| MIR450B | Osteoporosis, Postmenopausal | 3.78 |
| MIR625 | Osteoporosis, Postmenopausal | 3.78 |
| MTERF1A | Osteoporosis, Postmenopausal | 3.78 |
| NUTM2D | Osteoporosis, Postmenopausal | 3.78 |
| OR51A7 | Osteoporosis, Postmenopausal | 3.78 |
| OR52L1 | Osteoporosis, Postmenopausal | 3.78 |
| OR52W1 | Osteoporosis, Postmenopausal | 3.78 |
| PRR35 | Osteoporosis, Postmenopausal | 3.78 |
| PYDC1 | Osteoporosis, Postmenopausal | 3.78 |
| RAB41 | Osteoporosis, Postmenopausal | 3.78 |
| SNORA20 | Osteoporosis, Postmenopausal | 3.78 |
| SNORA54 | Osteoporosis, Postmenopausal | 3.78 |
| SNORD21 | Osteoporosis, Postmenopausal | 3.78 |
| SNORD50B | Osteoporosis, Postmenopausal | 3.78 |
| SPDYE4 | Osteoporosis, Postmenopausal | 3.78 |
| TRIM60 | Osteoporosis, Postmenopausal | 3.78 |
| C13H1ORF115 | Osteoporosis, Postmenopausal | 3.77 |
| GLRX3 | Osteoporosis, Postmenopausal | 3.77 |
| PRORSD1P | Osteoporosis, Postmenopausal | 3.77 |
| SRARP | Osteoporosis, Postmenopausal | 3.77 |
| ZSCAN4 | Osteoporosis, Postmenopausal | 3.77 |
| ARAP1-AS2 | Osteoporosis, Postmenopausal | 3.76 |
| ATP1B2B | Osteoporosis, Postmenopausal | 3.76 |
| ATP5IF1B | Osteoporosis, Postmenopausal | 3.76 |
| BGNB | Osteoporosis, Postmenopausal | 3.76 |
| C10H17ORF75 | Osteoporosis, Postmenopausal | 3.76 |
| C20H6ORF136 | Osteoporosis, Postmenopausal | 3.76 |
| CA15B | Osteoporosis, Postmenopausal | 3.76 |
| CAHM | Osteoporosis, Postmenopausal | 3.76 |
| CAM | Osteoporosis, Postmenopausal | 3.76 |
| CELSR1A | Osteoporosis, Postmenopausal | 3.76 |
| CYP3C4 | Osteoporosis, Postmenopausal | 3.76 |
| DEFA10 | Osteoporosis, Postmenopausal | 3.76 |
| DEFB6 | Osteoporosis, Postmenopausal | 3.76 |
| EGFEM1 | Osteoporosis, Postmenopausal | 3.76 |
| EMSLR | Osteoporosis, Postmenopausal | 3.76 |
| EPHB3A | Osteoporosis, Postmenopausal | 3.76 |
| FAM181A | Osteoporosis, Postmenopausal | 3.76 |
| FAM26F | Osteoporosis, Postmenopausal | 3.76 |
| FGF13A | Osteoporosis, Postmenopausal | 3.76 |
| FGFRL1A | Osteoporosis, Postmenopausal | 3.76 |
| FTR83 | Osteoporosis, Postmenopausal | 3.76 |
| GCN1L1 | Osteoporosis, Postmenopausal | 3.76 |
| GKN3 | Osteoporosis, Postmenopausal | 3.76 |
| GLIS3-AS1 | Osteoporosis, Postmenopausal | 3.76 |
| HIF1A-AS2 | Osteoporosis, Postmenopausal | 3.76 |
| HSP47 | Osteoporosis, Postmenopausal | 3.76 |
| ITGB1A | Osteoporosis, Postmenopausal | 3.76 |
| ITGB1B.2 | Osteoporosis, Postmenopausal | 3.76 |
| JAM3B | Osteoporosis, Postmenopausal | 3.76 |
| KLK5 | Osteoporosis, Postmenopausal | 3.76 |
| KLRA15 | Osteoporosis, Postmenopausal | 3.76 |
| LINC00852 | Osteoporosis, Postmenopausal | 3.76 |
| LINC01341 | Osteoporosis, Postmenopausal | 3.76 |
| LIPOGENIN | Osteoporosis, Postmenopausal | 3.76 |
| MFSD2AA | Osteoporosis, Postmenopausal | 3.76 |
| MIR1296 | Osteoporosis, Postmenopausal | 3.76 |
| MIR1305 | Osteoporosis, Postmenopausal | 3.76 |
| MRPL45 | Osteoporosis, Postmenopausal | 3.76 |
| MSX2B | Osteoporosis, Postmenopausal | 3.76 |
| MTNB | Osteoporosis, Postmenopausal | 3.76 |
| NADKB | Osteoporosis, Postmenopausal | 3.76 |
| NEFLB | Osteoporosis, Postmenopausal | 3.76 |
| NFIL3-2 | Osteoporosis, Postmenopausal | 3.76 |
| NIBAN2A | Osteoporosis, Postmenopausal | 3.76 |
| NID2A | Osteoporosis, Postmenopausal | 3.76 |
| NPIPB7 | Osteoporosis, Postmenopausal | 3.76 |
| OR10K2 | Osteoporosis, Postmenopausal | 3.76 |
| OR2Y13 | Osteoporosis, Postmenopausal | 3.76 |
| OR7E13P | Osteoporosis, Postmenopausal | 3.76 |
| PAX7A | Osteoporosis, Postmenopausal | 3.76 |
| PDHA1A | Osteoporosis, Postmenopausal | 3.76 |
| PKP3A | Osteoporosis, Postmenopausal | 3.76 |
| PRR27 | Osteoporosis, Postmenopausal | 3.76 |
| PTBP1A | Osteoporosis, Postmenopausal | 3.76 |
| QRFP | Osteoporosis, Postmenopausal | 3.76 |
| RGD1564480 | Osteoporosis, Postmenopausal | 3.76 |
| RHCGB | Osteoporosis, Postmenopausal | 3.76 |
| RNASE1L1 | Osteoporosis, Postmenopausal | 3.76 |
| RORCB | Osteoporosis, Postmenopausal | 3.76 |
| RPL13AP6 | Osteoporosis, Postmenopausal | 3.76 |
| SNAI1B | Osteoporosis, Postmenopausal | 3.76 |
| SNORA6 | Osteoporosis, Postmenopausal | 3.76 |
| SNORA67 | Osteoporosis, Postmenopausal | 3.76 |
| SNORD44 | Osteoporosis, Postmenopausal | 3.76 |
| SOX4A | Osteoporosis, Postmenopausal | 3.76 |
| TEX47 | Osteoporosis, Postmenopausal | 3.76 |
| TMEM56 | Osteoporosis, Postmenopausal | 3.76 |
| TMPRSS11B | Osteoporosis, Postmenopausal | 3.76 |
| TMPRSS4A | Osteoporosis, Postmenopausal | 3.76 |
| TRIM6-TRIM34 | Osteoporosis, Postmenopausal | 3.76 |
| UHMK1L1 | Osteoporosis, Postmenopausal | 3.76 |
| ZFP300 | Osteoporosis, Postmenopausal | 3.76 |
| ZNF177 | Osteoporosis, Postmenopausal | 3.76 |
| DCAF16 | Osteoporosis, Postmenopausal | 3.75 |
| IFNA4 | Osteoporosis, Postmenopausal | 3.75 |
| LINC00526 | Osteoporosis, Postmenopausal | 3.75 |
| SNHG20 | Osteoporosis, Postmenopausal | 3.75 |
| ZNF788P | Osteoporosis, Postmenopausal | 3.75 |
| ZNF875 | Osteoporosis, Postmenopausal | 3.75 |
| ANKRD20A8P | Osteoporosis, Postmenopausal | 3.74 |
| ANKRD60 | Osteoporosis, Postmenopausal | 3.74 |
| ATF5B | Osteoporosis, Postmenopausal | 3.74 |
| C6ORF15 | Osteoporosis, Postmenopausal | 3.74 |
| CELP | Osteoporosis, Postmenopausal | 3.74 |
| CSHL1 | Osteoporosis, Postmenopausal | 3.74 |
| CYCSB | Osteoporosis, Postmenopausal | 3.74 |
| DEFB132 | Osteoporosis, Postmenopausal | 3.74 |
| EGL-1 | Osteoporosis, Postmenopausal | 3.74 |
| EPHB1 | Osteoporosis, Postmenopausal | 3.74 |
| FAM170A | Osteoporosis, Postmenopausal | 3.74 |
| H1-9P | Osteoporosis, Postmenopausal | 3.74 |
| IGHG3 | Osteoporosis, Postmenopausal | 3.74 |
| ITIH6 | Osteoporosis, Postmenopausal | 3.74 |
| KLK1B3 | Osteoporosis, Postmenopausal | 3.74 |
| KRTAP1-1 | Osteoporosis, Postmenopausal | 3.74 |
| KRTAP5-5 | Osteoporosis, Postmenopausal | 3.74 |
| KRTAP6-1 | Osteoporosis, Postmenopausal | 3.74 |
| KRTAP6-2 | Osteoporosis, Postmenopausal | 3.74 |
| LCE1A | Osteoporosis, Postmenopausal | 3.74 |
| LINC00479 | Osteoporosis, Postmenopausal | 3.74 |
| MAK10 | Osteoporosis, Postmenopausal | 3.74 |
| MEPE | Osteoporosis, Postmenopausal | 3.74 |
| MIR350 | Osteoporosis, Postmenopausal | 3.74 |
| MIR622 | Osteoporosis, Postmenopausal | 3.74 |
| MS4A3 | Osteoporosis, Postmenopausal | 3.74 |
| OR5AU1 | Osteoporosis, Postmenopausal | 3.74 |
| PRL3B1 | Osteoporosis, Postmenopausal | 3.74 |
| RAB6C | Osteoporosis, Postmenopausal | 3.74 |
| SCGB1D4 | Osteoporosis, Postmenopausal | 3.74 |
| SKOR2 | Osteoporosis, Postmenopausal | 3.74 |
| SLC25A31 | Osteoporosis, Postmenopausal | 3.74 |
| TADA3L | Osteoporosis, Postmenopausal | 3.74 |
| TMPRSS11E | Osteoporosis, Postmenopausal | 3.74 |
| TPRXL | Osteoporosis, Postmenopausal | 3.74 |
| TTC24 | Osteoporosis, Postmenopausal | 3.74 |
| UBL4B | Osteoporosis, Postmenopausal | 3.74 |
| ZNF66 | Osteoporosis, Postmenopausal | 3.74 |
| ZNF843 | Osteoporosis, Postmenopausal | 3.74 |
| ADAMTSL3 | Osteoporosis, Postmenopausal | 3.73 |
| NAT8B | Osteoporosis, Postmenopausal | 3.73 |
| POTEM | Osteoporosis, Postmenopausal | 3.73 |
| PPP2R3B | Osteoporosis, Postmenopausal | 3.73 |
| SNX16 | Osteoporosis, Postmenopausal | 3.73 |
| ZPBP | Osteoporosis, Postmenopausal | 3.73 |
| ADGRG4 | Osteoporosis, Postmenopausal | 3.72 |
| ASCL1A | Osteoporosis, Postmenopausal | 3.72 |
| HPDA | Osteoporosis, Postmenopausal | 3.72 |
| KLRA5 | Osteoporosis, Postmenopausal | 3.72 |
| MAP3K11 | Osteoporosis, Postmenopausal | 3.72 |
| MIR125B1 | Osteoporosis, Postmenopausal | 3.72 |
| MIR431 | Osteoporosis, Postmenopausal | 3.72 |
| SERPINI2 | Osteoporosis, Postmenopausal | 3.72 |
| SLC16A9B | Osteoporosis, Postmenopausal | 3.72 |
| SLC5A4B | Osteoporosis, Postmenopausal | 3.72 |
| SPACA5 | Osteoporosis, Postmenopausal | 3.72 |
| TDRD12 | Osteoporosis, Postmenopausal | 3.72 |
| TMEM114 | Osteoporosis, Postmenopausal | 3.72 |
| TMEM273 | Osteoporosis, Postmenopausal | 3.72 |
| ZFP146 | Osteoporosis, Postmenopausal | 3.72 |
| ZNF112 | Osteoporosis, Postmenopausal | 3.72 |
| ELL | Osteoporosis, Postmenopausal | 3.71 |
| PPP1R13B | Osteoporosis, Postmenopausal | 3.71 |
| TMEM102 | Osteoporosis, Postmenopausal | 3.71 |
| ALDH2.1 | Osteoporosis, Postmenopausal | 3.7 |
| ASF1BA | Osteoporosis, Postmenopausal | 3.7 |
| B430306N03RIKL | Osteoporosis, Postmenopausal | 3.7 |
| BPIFB3 | Osteoporosis, Postmenopausal | 3.7 |
| C17ORF107 | Osteoporosis, Postmenopausal | 3.7 |
| C2H4ORF33 | Osteoporosis, Postmenopausal | 3.7 |
| C3H9ORF50 | Osteoporosis, Postmenopausal | 3.7 |
| C3ORF22 | Osteoporosis, Postmenopausal | 3.7 |
| CACNA1DA | Osteoporosis, Postmenopausal | 3.7 |
| CACNG2A | Osteoporosis, Postmenopausal | 3.7 |
| CAMK2D1 | Osteoporosis, Postmenopausal | 3.7 |
| CD74B | Osteoporosis, Postmenopausal | 3.7 |
| CGB8 | Osteoporosis, Postmenopausal | 3.7 |
| CIMIP4 | Osteoporosis, Postmenopausal | 3.7 |
| CRYBB1L3 | Osteoporosis, Postmenopausal | 3.7 |
| CYP3C3 | Osteoporosis, Postmenopausal | 3.7 |
| ELOVL6L | Osteoporosis, Postmenopausal | 3.7 |
| ELTD1 | Osteoporosis, Postmenopausal | 3.7 |
| EVPLL | Osteoporosis, Postmenopausal | 3.7 |
| FAM86C2P | Osteoporosis, Postmenopausal | 3.7 |
| FTH1B | Osteoporosis, Postmenopausal | 3.7 |
| GAS8-AS1 | Osteoporosis, Postmenopausal | 3.7 |
| GCSHP3 | Osteoporosis, Postmenopausal | 3.7 |
| GNG14 | Osteoporosis, Postmenopausal | 3.7 |
| GPR89 | Osteoporosis, Postmenopausal | 3.7 |
| GRAMD3 | Osteoporosis, Postmenopausal | 3.7 |
| GRIN1A | Osteoporosis, Postmenopausal | 3.7 |
| H1-8 | Osteoporosis, Postmenopausal | 3.7 |
| HAND2-AS1 | Osteoporosis, Postmenopausal | 3.7 |
| HIBADHB | Osteoporosis, Postmenopausal | 3.7 |
| HSD17B12B | Osteoporosis, Postmenopausal | 3.7 |
| HSP68 | Osteoporosis, Postmenopausal | 3.7 |
| HSPA4B | Osteoporosis, Postmenopausal | 3.7 |
| KCNIP2 | Osteoporosis, Postmenopausal | 3.7 |
| KMT2E-AS1 | Osteoporosis, Postmenopausal | 3.7 |
| KNCN | Osteoporosis, Postmenopausal | 3.7 |
| KRTAP2-1 | Osteoporosis, Postmenopausal | 3.7 |
| LCN10 | Osteoporosis, Postmenopausal | 3.7 |
| LINC00460 | Osteoporosis, Postmenopausal | 3.7 |
| LMNTD1 | Osteoporosis, Postmenopausal | 3.7 |
| LOH12CR2 | Osteoporosis, Postmenopausal | 3.7 |
| LYVE1A | Osteoporosis, Postmenopausal | 3.7 |
| MEF2C-AS1 | Osteoporosis, Postmenopausal | 3.7 |
| MIR133A1 | Osteoporosis, Postmenopausal | 3.7 |
| MIR302D | Osteoporosis, Postmenopausal | 3.7 |
| MIR365A | Osteoporosis, Postmenopausal | 3.7 |
| MIR4497 | Osteoporosis, Postmenopausal | 3.7 |
| MIR504 | Osteoporosis, Postmenopausal | 3.7 |
| MIR766 | Osteoporosis, Postmenopausal | 3.7 |
| MKRN4 | Osteoporosis, Postmenopausal | 3.7 |
| MROH5 | Osteoporosis, Postmenopausal | 3.7 |
| MS4A12 | Osteoporosis, Postmenopausal | 3.7 |
| MS4A4E | Osteoporosis, Postmenopausal | 3.7 |
| MT1DP | Osteoporosis, Postmenopausal | 3.7 |
| NEFLA | Osteoporosis, Postmenopausal | 3.7 |
| OCLNA | Osteoporosis, Postmenopausal | 3.7 |
| OCLNB | Osteoporosis, Postmenopausal | 3.7 |
| OR11G2 | Osteoporosis, Postmenopausal | 3.7 |
| OR13J1 | Osteoporosis, Postmenopausal | 3.7 |
| OR4F15 | Osteoporosis, Postmenopausal | 3.7 |
| OR51B17 | Osteoporosis, Postmenopausal | 3.7 |
| OR51S1 | Osteoporosis, Postmenopausal | 3.7 |
| OR7E91P | Osteoporosis, Postmenopausal | 3.7 |
| OR9G4 | Osteoporosis, Postmenopausal | 3.7 |
| PCOTH | Osteoporosis, Postmenopausal | 3.7 |
| PRDM16-DT | Osteoporosis, Postmenopausal | 3.7 |
| PRKG1A | Osteoporosis, Postmenopausal | 3.7 |
| PRSS54 | Osteoporosis, Postmenopausal | 3.7 |
| PSMG3-AS1 | Osteoporosis, Postmenopausal | 3.7 |
| PTBP1B | Osteoporosis, Postmenopausal | 3.7 |
| PTX4 | Osteoporosis, Postmenopausal | 3.7 |
| RAB40AL | Osteoporosis, Postmenopausal | 3.7 |
| RAET1G | Osteoporosis, Postmenopausal | 3.7 |
| RNASE13 | Osteoporosis, Postmenopausal | 3.7 |
| RNU6ATAC | Osteoporosis, Postmenopausal | 3.7 |
| RUNX2B | Osteoporosis, Postmenopausal | 3.7 |
| SBPL | Osteoporosis, Postmenopausal | 3.7 |
| SCARNA2 | Osteoporosis, Postmenopausal | 3.7 |
| SCGB1D1 | Osteoporosis, Postmenopausal | 3.7 |
| SCINLB | Osteoporosis, Postmenopausal | 3.7 |
| SEMA3AA | Osteoporosis, Postmenopausal | 3.7 |
| SLC1A2B | Osteoporosis, Postmenopausal | 3.7 |
| SLC25A41 | Osteoporosis, Postmenopausal | 3.7 |
| SNORA9 | Osteoporosis, Postmenopausal | 3.7 |
| SSR4P1 | Osteoporosis, Postmenopausal | 3.7 |
| STX11B.1 | Osteoporosis, Postmenopausal | 3.7 |
| SUZ12P1 | Osteoporosis, Postmenopausal | 3.7 |
| TAAR2 | Osteoporosis, Postmenopausal | 3.7 |
| TAS2R38 | Osteoporosis, Postmenopausal | 3.7 |
| TGM1L1 | Osteoporosis, Postmenopausal | 3.7 |
| THAP10 | Osteoporosis, Postmenopausal | 3.7 |
| THOC4 | Osteoporosis, Postmenopausal | 3.7 |
| TIGD1 | Osteoporosis, Postmenopausal | 3.7 |
| TNIKA | Osteoporosis, Postmenopausal | 3.7 |
| TTC35 | Osteoporosis, Postmenopausal | 3.7 |
| TUBA2 | Osteoporosis, Postmenopausal | 3.7 |
| TYMSOS | Osteoporosis, Postmenopausal | 3.7 |
| USP2-AS1 | Osteoporosis, Postmenopausal | 3.7 |
| WDR87 | Osteoporosis, Postmenopausal | 3.7 |
| XPO1B | Osteoporosis, Postmenopausal | 3.7 |
| ZNF280A | Osteoporosis, Postmenopausal | 3.7 |
| ZNF285 | Osteoporosis, Postmenopausal | 3.7 |
| ZNF594 | Osteoporosis, Postmenopausal | 3.7 |
| DIO3OS | Osteoporosis, Postmenopausal | 3.69 |
| FAM169B | Osteoporosis, Postmenopausal | 3.69 |
| GADD45BB | Osteoporosis, Postmenopausal | 3.69 |
| MIR370 | Osteoporosis, Postmenopausal | 3.69 |
| MTNA | Osteoporosis, Postmenopausal | 3.69 |
| SMIM6 | Osteoporosis, Postmenopausal | 3.69 |
| CACNA1A | Osteoporosis, Postmenopausal | 3.68 |
| SCD4 | Osteoporosis, Postmenopausal | 3.68 |
| BTN3A3 | Osteoporosis, Postmenopausal | 3.67 |
| C12ORF60 | Osteoporosis, Postmenopausal | 3.67 |
| C17ORF58 | Osteoporosis, Postmenopausal | 3.67 |
| CATSPER1 | Osteoporosis, Postmenopausal | 3.67 |
| COL6A4 | Osteoporosis, Postmenopausal | 3.67 |
| CYP1A3 | Osteoporosis, Postmenopausal | 3.67 |
| DESI2 | Osteoporosis, Postmenopausal | 3.67 |
| FUT6 | Osteoporosis, Postmenopausal | 3.67 |
| GPR82 | Osteoporosis, Postmenopausal | 3.67 |
| PROL1 | Osteoporosis, Postmenopausal | 3.67 |
| PRSS32 | Osteoporosis, Postmenopausal | 3.67 |
| RP9P | Osteoporosis, Postmenopausal | 3.67 |
| ZFP819 | Osteoporosis, Postmenopausal | 3.67 |
| ZNF75A | Osteoporosis, Postmenopausal | 3.67 |
| ZNF850 | Osteoporosis, Postmenopausal | 3.67 |
| ANKRD33 | Osteoporosis, Postmenopausal | 3.66 |
| ATP11AUN | Osteoporosis, Postmenopausal | 3.66 |
| BANF2 | Osteoporosis, Postmenopausal | 3.66 |
| CCDC27 | Osteoporosis, Postmenopausal | 3.66 |
| CD200R1L | Osteoporosis, Postmenopausal | 3.66 |
| CED-4 | Osteoporosis, Postmenopausal | 3.66 |
| CED-9 | Osteoporosis, Postmenopausal | 3.66 |
| CLPSL2 | Osteoporosis, Postmenopausal | 3.66 |
| CSNK1A1P1 | Osteoporosis, Postmenopausal | 3.66 |
| CTRB2 | Osteoporosis, Postmenopausal | 3.66 |
| DGAT2L6 | Osteoporosis, Postmenopausal | 3.66 |
| GARIN5B | Osteoporosis, Postmenopausal | 3.66 |
| GPR152 | Osteoporosis, Postmenopausal | 3.66 |
| HERC2P2 | Osteoporosis, Postmenopausal | 3.66 |
| LCE3E | Osteoporosis, Postmenopausal | 3.66 |
| LELP1 | Osteoporosis, Postmenopausal | 3.66 |
| MAGEA4 | Osteoporosis, Postmenopausal | 3.66 |
| MIR181A2 | Osteoporosis, Postmenopausal | 3.66 |
| MIR323 | Osteoporosis, Postmenopausal | 3.66 |
| MIR9-1 | Osteoporosis, Postmenopausal | 3.66 |
| MUC21 | Osteoporosis, Postmenopausal | 3.66 |
| OR10J3 | Osteoporosis, Postmenopausal | 3.66 |
| OR52E4 | Osteoporosis, Postmenopausal | 3.66 |
| OR6N1 | Osteoporosis, Postmenopausal | 3.66 |
| SLFN12L | Osteoporosis, Postmenopausal | 3.66 |
| SNORD15A | Osteoporosis, Postmenopausal | 3.66 |
| SPDYE8 | Osteoporosis, Postmenopausal | 3.66 |
| TUBB7P | Osteoporosis, Postmenopausal | 3.66 |
| ABRAA | Osteoporosis, Postmenopausal | 3.65 |
| ADAM2 | Osteoporosis, Postmenopausal | 3.65 |
| AGR1 | Osteoporosis, Postmenopausal | 3.65 |
| ANKRD18B | Osteoporosis, Postmenopausal | 3.65 |
| ATP1A2A | Osteoporosis, Postmenopausal | 3.65 |
| C10H17ORF49 | Osteoporosis, Postmenopausal | 3.65 |
| C3ORF33 | Osteoporosis, Postmenopausal | 3.65 |
| C5H1ORF174 | Osteoporosis, Postmenopausal | 3.65 |
| CCL34A.4 | Osteoporosis, Postmenopausal | 3.65 |
| CLDN15LA | Osteoporosis, Postmenopausal | 3.65 |
| CLDNK | Osteoporosis, Postmenopausal | 3.65 |
| COL10A1A | Osteoporosis, Postmenopausal | 3.65 |
| CPT1AA | Osteoporosis, Postmenopausal | 3.65 |
| CRXOS1 | Osteoporosis, Postmenopausal | 3.65 |
| DNAJA3A | Osteoporosis, Postmenopausal | 3.65 |
| FDXACB1 | Osteoporosis, Postmenopausal | 3.65 |
| FKBP1C | Osteoporosis, Postmenopausal | 3.65 |
| FV1 | Osteoporosis, Postmenopausal | 3.65 |
| GALCB | Osteoporosis, Postmenopausal | 3.65 |
| GATA3-AS1 | Osteoporosis, Postmenopausal | 3.65 |
| GSTA3L1 | Osteoporosis, Postmenopausal | 3.65 |
| HPDB | Osteoporosis, Postmenopausal | 3.65 |
| HPXA | Osteoporosis, Postmenopausal | 3.65 |
| IFIT1BL | Osteoporosis, Postmenopausal | 3.65 |
| IL17RA1A | Osteoporosis, Postmenopausal | 3.65 |
| IQCF1 | Osteoporosis, Postmenopausal | 3.65 |
| KLK13 | Osteoporosis, Postmenopausal | 3.65 |
| KLK4 | Osteoporosis, Postmenopausal | 3.65 |
| KRT91 | Osteoporosis, Postmenopausal | 3.65 |
| LINC01235 | Osteoporosis, Postmenopausal | 3.65 |
| LINC01305 | Osteoporosis, Postmenopausal | 3.65 |
| LY6CL1 | Osteoporosis, Postmenopausal | 3.65 |
| MEIS3P1 | Osteoporosis, Postmenopausal | 3.65 |
| MIR499A | Osteoporosis, Postmenopausal | 3.65 |
| MIR505 | Osteoporosis, Postmenopausal | 3.65 |
| MIR616 | Osteoporosis, Postmenopausal | 3.65 |
| MIR675 | Osteoporosis, Postmenopausal | 3.65 |
| MIR765 | Osteoporosis, Postmenopausal | 3.65 |
| MSANTD1 | Osteoporosis, Postmenopausal | 3.65 |
| NR2F5 | Osteoporosis, Postmenopausal | 3.65 |
| NT5C2B | Osteoporosis, Postmenopausal | 3.65 |
| OR51I1 | Osteoporosis, Postmenopausal | 3.65 |
| OR5B21 | Osteoporosis, Postmenopausal | 3.65 |
| OR9S14 | Osteoporosis, Postmenopausal | 3.65 |
| PCDH10A | Osteoporosis, Postmenopausal | 3.65 |
| PDGFA-DT | Osteoporosis, Postmenopausal | 3.65 |
| PEBP4 | Osteoporosis, Postmenopausal | 3.65 |
| PVRL3 | Osteoporosis, Postmenopausal | 3.65 |
| RNASEKA | Osteoporosis, Postmenopausal | 3.65 |
| RNF148 | Osteoporosis, Postmenopausal | 3.65 |
| RPL13AP20 | Osteoporosis, Postmenopausal | 3.65 |
| RPL13AP3 | Osteoporosis, Postmenopausal | 3.65 |
| RPL34-DT | Osteoporosis, Postmenopausal | 3.65 |
| SCAND3 | Osteoporosis, Postmenopausal | 3.65 |
| SLC1A9 | Osteoporosis, Postmenopausal | 3.65 |
| SNORD35A | Osteoporosis, Postmenopausal | 3.65 |
| SNORD47 | Osteoporosis, Postmenopausal | 3.65 |
| SUGT1P1 | Osteoporosis, Postmenopausal | 3.65 |
| SULT1C2AL1 | Osteoporosis, Postmenopausal | 3.65 |
| SVS3A | Osteoporosis, Postmenopausal | 3.65 |
| TCL1B | Osteoporosis, Postmenopausal | 3.65 |
| TMEM220-AS1 | Osteoporosis, Postmenopausal | 3.65 |
| TMEM51-AS1 | Osteoporosis, Postmenopausal | 3.65 |
| TOLLIP-DT | Osteoporosis, Postmenopausal | 3.65 |
| TPM4B | Osteoporosis, Postmenopausal | 3.65 |
| VWA5AL1 | Osteoporosis, Postmenopausal | 3.65 |
| WHAMMP2 | Osteoporosis, Postmenopausal | 3.65 |
| ZEB2OS | Osteoporosis, Postmenopausal | 3.65 |
| ZFP133 | Osteoporosis, Postmenopausal | 3.65 |
| ZFP383 | Osteoporosis, Postmenopausal | 3.65 |
| ZKSCAN8P1 | Osteoporosis, Postmenopausal | 3.65 |
| ZNF316 | Osteoporosis, Postmenopausal | 3.65 |
| ZNF708 | Osteoporosis, Postmenopausal | 3.65 |
| MOSMO | Osteoporosis, Postmenopausal | 3.64 |
| PAX1 | Osteoporosis, Postmenopausal | 3.64 |
| SLFN11 | Osteoporosis, Postmenopausal | 3.64 |
| ARR3 | Osteoporosis, Postmenopausal | 3.63 |
| BARX1 | Osteoporosis, Postmenopausal | 3.63 |
| C8ORF44 | Osteoporosis, Postmenopausal | 3.63 |
| CABP5 | Osteoporosis, Postmenopausal | 3.63 |
| CAR10 | Osteoporosis, Postmenopausal | 3.63 |
| CED-3 | Osteoporosis, Postmenopausal | 3.63 |
| CFAP107 | Osteoporosis, Postmenopausal | 3.63 |
| CFAP221 | Osteoporosis, Postmenopausal | 3.63 |
| CFAP54 | Osteoporosis, Postmenopausal | 3.63 |
| CHCT1 | Osteoporosis, Postmenopausal | 3.63 |
| CYP3A59 | Osteoporosis, Postmenopausal | 3.63 |
| CYTIP | Osteoporosis, Postmenopausal | 3.63 |
| DTR | Osteoporosis, Postmenopausal | 3.63 |
| FAM170B | Osteoporosis, Postmenopausal | 3.63 |
| GPR139 | Osteoporosis, Postmenopausal | 3.63 |
| HMGB1P1 | Osteoporosis, Postmenopausal | 3.63 |
| LINC00482 | Osteoporosis, Postmenopausal | 3.63 |
| LLCFC1 | Osteoporosis, Postmenopausal | 3.63 |
| MAGEA12 | Osteoporosis, Postmenopausal | 3.63 |
| MAGEB2 | Osteoporosis, Postmenopausal | 3.63 |
| MIR129-2 | Osteoporosis, Postmenopausal | 3.63 |
| MIR208A | Osteoporosis, Postmenopausal | 3.63 |
| MIR433 | Osteoporosis, Postmenopausal | 3.63 |
| MRGPRX1 | Osteoporosis, Postmenopausal | 3.63 |
| MYL11 | Osteoporosis, Postmenopausal | 3.63 |
| NEK7 | Osteoporosis, Postmenopausal | 3.63 |
| NUP160 | Osteoporosis, Postmenopausal | 3.63 |
| OR10Z1 | Osteoporosis, Postmenopausal | 3.63 |
| OR1F1 | Osteoporosis, Postmenopausal | 3.63 |
| OR4C3 | Osteoporosis, Postmenopausal | 3.63 |
| OR51G1 | Osteoporosis, Postmenopausal | 3.63 |
| RCOR3 | Osteoporosis, Postmenopausal | 3.63 |
| RCVRN | Osteoporosis, Postmenopausal | 3.63 |
| RDH2 | Osteoporosis, Postmenopausal | 3.63 |
| SNORA5A | Osteoporosis, Postmenopausal | 3.63 |
| SP140L | Osteoporosis, Postmenopausal | 3.63 |
| SPAM1 | Osteoporosis, Postmenopausal | 3.63 |
| SPANXD | Osteoporosis, Postmenopausal | 3.63 |
| TAAR5 | Osteoporosis, Postmenopausal | 3.63 |
| TEX29 | Osteoporosis, Postmenopausal | 3.63 |
| TMSB4Y | Osteoporosis, Postmenopausal | 3.63 |
| TRIM52 | Osteoporosis, Postmenopausal | 3.63 |
| TSSK4 | Osteoporosis, Postmenopausal | 3.63 |
| TTN.2 | Osteoporosis, Postmenopausal | 3.63 |
| TYSND1 | Osteoporosis, Postmenopausal | 3.63 |
| WFDC15B | Osteoporosis, Postmenopausal | 3.63 |
| ZNF468 | Osteoporosis, Postmenopausal | 3.63 |
| ZNF831 | Osteoporosis, Postmenopausal | 3.63 |
| TRAF3IP2 | Osteoporosis, Postmenopausal | 3.62 |
| AFDN-DT | Osteoporosis, Postmenopausal | 3.61 |
| AOX4 | Osteoporosis, Postmenopausal | 3.61 |
| BPIFB2 | Osteoporosis, Postmenopausal | 3.61 |
| LRRC20 | Osteoporosis, Postmenopausal | 3.61 |
| OR51E1 | Osteoporosis, Postmenopausal | 3.61 |
| PGBD3 | Osteoporosis, Postmenopausal | 3.61 |
| TMEM213 | Osteoporosis, Postmenopausal | 3.61 |
| ZNF184 | Osteoporosis, Postmenopausal | 3.61 |
| ACCSL | Osteoporosis, Postmenopausal | 3.6 |
| ADKA | Osteoporosis, Postmenopausal | 3.6 |
| ALMS1-IT1 | Osteoporosis, Postmenopausal | 3.6 |
| APOA4A | Osteoporosis, Postmenopausal | 3.6 |
| ARHGAP27P1 | Osteoporosis, Postmenopausal | 3.6 |
| ATP5F1EP2 | Osteoporosis, Postmenopausal | 3.6 |
| BCL6AA | Osteoporosis, Postmenopausal | 3.6 |
| BIRC5A | Osteoporosis, Postmenopausal | 3.6 |
| BNIP3LB | Osteoporosis, Postmenopausal | 3.6 |
| C16ORF54 | Osteoporosis, Postmenopausal | 3.6 |
| C16ORF86 | Osteoporosis, Postmenopausal | 3.6 |
| C16ORF89 | Osteoporosis, Postmenopausal | 3.6 |
| C9ORF50 | Osteoporosis, Postmenopausal | 3.6 |
| CAVIN1B | Osteoporosis, Postmenopausal | 3.6 |
| CCDC162P | Osteoporosis, Postmenopausal | 3.6 |
| CGB1 | Osteoporosis, Postmenopausal | 3.6 |
| CHIA.1 | Osteoporosis, Postmenopausal | 3.6 |
| CLCA4L | Osteoporosis, Postmenopausal | 3.6 |
| CRABP1A | Osteoporosis, Postmenopausal | 3.6 |
| CSH2 | Osteoporosis, Postmenopausal | 3.6 |
| DNAJB6B | Osteoporosis, Postmenopausal | 3.6 |
| EPB41L4A-DT | Osteoporosis, Postmenopausal | 3.6 |
| EXOC3L | Osteoporosis, Postmenopausal | 3.6 |
| FAM86HP | Osteoporosis, Postmenopausal | 3.6 |
| FGF7P6 | Osteoporosis, Postmenopausal | 3.6 |
| GNB3B | Osteoporosis, Postmenopausal | 3.6 |
| GZMH | Osteoporosis, Postmenopausal | 3.6 |
| H1-10-AS1 | Osteoporosis, Postmenopausal | 3.6 |
| HOXA3A | Osteoporosis, Postmenopausal | 3.6 |
| HTR1E | Osteoporosis, Postmenopausal | 3.6 |
| IGLL3P | Osteoporosis, Postmenopausal | 3.6 |
| IGSF7L1 | Osteoporosis, Postmenopausal | 3.6 |
| IRX2-DT | Osteoporosis, Postmenopausal | 3.6 |
| KA11 | Osteoporosis, Postmenopausal | 3.6 |
| KCNA10 | Osteoporosis, Postmenopausal | 3.6 |
| KRT74 | Osteoporosis, Postmenopausal | 3.6 |
| KRTAP19-1 | Osteoporosis, Postmenopausal | 3.6 |
| LDLRAD2 | Osteoporosis, Postmenopausal | 3.6 |
| LGALS7B | Osteoporosis, Postmenopausal | 3.6 |
| LINC01133 | Osteoporosis, Postmenopausal | 3.6 |
| LINC02035 | Osteoporosis, Postmenopausal | 3.6 |
| LINC02910 | Osteoporosis, Postmenopausal | 3.6 |
| LIPCA | Osteoporosis, Postmenopausal | 3.6 |
| MAFBA | Osteoporosis, Postmenopausal | 3.6 |
| MAFBB | Osteoporosis, Postmenopausal | 3.6 |
| MELTF-AS1 | Osteoporosis, Postmenopausal | 3.6 |
| MIR1228 | Osteoporosis, Postmenopausal | 3.6 |
| MIR1260B | Osteoporosis, Postmenopausal | 3.6 |
| MIR181B1 | Osteoporosis, Postmenopausal | 3.6 |
| MIR486-1 | Osteoporosis, Postmenopausal | 3.6 |
| MIR489 | Osteoporosis, Postmenopausal | 3.6 |
| MIR498 | Osteoporosis, Postmenopausal | 3.6 |
| MIR653 | Osteoporosis, Postmenopausal | 3.6 |
| MKL2 | Osteoporosis, Postmenopausal | 3.6 |
| MYH11A | Osteoporosis, Postmenopausal | 3.6 |
| NFIL3-5 | Osteoporosis, Postmenopausal | 3.6 |
| NR1D4A | Osteoporosis, Postmenopausal | 3.6 |
| NSFA | Osteoporosis, Postmenopausal | 3.6 |
| OR10AA1 | Osteoporosis, Postmenopausal | 3.6 |
| OR5C1 | Osteoporosis, Postmenopausal | 3.6 |
| PIRA12 | Osteoporosis, Postmenopausal | 3.6 |
| PRICKLE1A | Osteoporosis, Postmenopausal | 3.6 |
| PRKACG | Osteoporosis, Postmenopausal | 3.6 |
| PRLH | Osteoporosis, Postmenopausal | 3.6 |
| RGL3A | Osteoporosis, Postmenopausal | 3.6 |
| RNF212B | Osteoporosis, Postmenopausal | 3.6 |
| SFTA2 | Osteoporosis, Postmenopausal | 3.6 |
| SMG1P1 | Osteoporosis, Postmenopausal | 3.6 |
| SMG1P3 | Osteoporosis, Postmenopausal | 3.6 |
| SMIM10 | Osteoporosis, Postmenopausal | 3.6 |
| SULT1ST3 | Osteoporosis, Postmenopausal | 3.6 |
| TEFB | Osteoporosis, Postmenopausal | 3.6 |
| TGM2B | Osteoporosis, Postmenopausal | 3.6 |
| TIMM23B | Osteoporosis, Postmenopausal | 3.6 |
| TOB2P1 | Osteoporosis, Postmenopausal | 3.6 |
| VPS9D1-AS1 | Osteoporosis, Postmenopausal | 3.6 |
| VTGA2.L | Osteoporosis, Postmenopausal | 3.6 |
| VTNA | Osteoporosis, Postmenopausal | 3.6 |
| WEE2-AS1 | Osteoporosis, Postmenopausal | 3.6 |
| ZNF497 | Osteoporosis, Postmenopausal | 3.6 |
| ASB12 | Osteoporosis, Postmenopausal | 3.59 |
| CCDC63 | Osteoporosis, Postmenopausal | 3.59 |
| DIDO1 | Osteoporosis, Postmenopausal | 3.59 |
| H2-T10 | Osteoporosis, Postmenopausal | 3.59 |
| H2-T22 | Osteoporosis, Postmenopausal | 3.59 |
| HRCT1 | Osteoporosis, Postmenopausal | 3.59 |
| KRT31 | Osteoporosis, Postmenopausal | 3.59 |
| MIR127 | Osteoporosis, Postmenopausal | 3.59 |
| MIR484 | Osteoporosis, Postmenopausal | 3.59 |
| PMCH | Osteoporosis, Postmenopausal | 3.59 |
| RFK | Osteoporosis, Postmenopausal | 3.59 |
| SNHG10 | Osteoporosis, Postmenopausal | 3.59 |
| SPATA21 | Osteoporosis, Postmenopausal | 3.59 |
| SULT3A1 | Osteoporosis, Postmenopausal | 3.59 |
| TMIGD1 | Osteoporosis, Postmenopausal | 3.59 |
| TPBGL | Osteoporosis, Postmenopausal | 3.59 |
| ZNF441 | Osteoporosis, Postmenopausal | 3.59 |
| ZNF777 | Osteoporosis, Postmenopausal | 3.59 |
| ALG10B | Osteoporosis, Postmenopausal | 3.58 |
| C1ORF21 | Osteoporosis, Postmenopausal | 3.58 |
| DUSP16 | Osteoporosis, Postmenopausal | 3.58 |
| GCG | Osteoporosis, Postmenopausal | 3.58 |
| MICOS10 | Osteoporosis, Postmenopausal | 3.58 |
| ANKRD34C | Osteoporosis, Postmenopausal | 3.57 |
| CCDC87 | Osteoporosis, Postmenopausal | 3.57 |
| COPRS | Osteoporosis, Postmenopausal | 3.57 |
| CTU2 | Osteoporosis, Postmenopausal | 3.57 |
| DENND6B | Osteoporosis, Postmenopausal | 3.57 |
| ELOVL4B | Osteoporosis, Postmenopausal | 3.57 |
| EOLA1 | Osteoporosis, Postmenopausal | 3.57 |
| FAM27E3 | Osteoporosis, Postmenopausal | 3.57 |
| GIMAP3 | Osteoporosis, Postmenopausal | 3.57 |
| GOLGA8IP | Osteoporosis, Postmenopausal | 3.57 |
| LCE2B | Osteoporosis, Postmenopausal | 3.57 |
| LINC01140 | Osteoporosis, Postmenopausal | 3.57 |
| MIR129-1 | Osteoporosis, Postmenopausal | 3.57 |
| MIR539 | Osteoporosis, Postmenopausal | 3.57 |
| NOPCHAP1 | Osteoporosis, Postmenopausal | 3.57 |
| PCDHGA4 | Osteoporosis, Postmenopausal | 3.57 |
| PMS2P5 | Osteoporosis, Postmenopausal | 3.57 |
| PPP3R2 | Osteoporosis, Postmenopausal | 3.57 |
| PRB1 | Osteoporosis, Postmenopausal | 3.57 |
| RGPD5 | Osteoporosis, Postmenopausal | 3.57 |
| RXFP4 | Osteoporosis, Postmenopausal | 3.57 |
| SDHAP1 | Osteoporosis, Postmenopausal | 3.57 |
| SERPINA1D | Osteoporosis, Postmenopausal | 3.57 |
| SRSF8 | Osteoporosis, Postmenopausal | 3.57 |
| STAT5 | Osteoporosis, Postmenopausal | 3.57 |
| SUN5 | Osteoporosis, Postmenopausal | 3.57 |
| TTTY14 | Osteoporosis, Postmenopausal | 3.57 |
| WDR95 | Osteoporosis, Postmenopausal | 3.57 |
| XAGE2 | Osteoporosis, Postmenopausal | 3.57 |
| ZFP707 | Osteoporosis, Postmenopausal | 3.57 |
| ZFP740 | Osteoporosis, Postmenopausal | 3.57 |
| ZFP750 | Osteoporosis, Postmenopausal | 3.57 |
| ZNF445 | Osteoporosis, Postmenopausal | 3.57 |
| ZNF513 | Osteoporosis, Postmenopausal | 3.57 |
| ZNF81 | Osteoporosis, Postmenopausal | 3.57 |
| ANG4 | Osteoporosis, Postmenopausal | 3.56 |
| ASZ1 | Osteoporosis, Postmenopausal | 3.56 |
| ATP1A1-AS1 | Osteoporosis, Postmenopausal | 3.56 |
| BCO1L | Osteoporosis, Postmenopausal | 3.56 |
| C1ORF53 | Osteoporosis, Postmenopausal | 3.56 |
| C2CD6 | Osteoporosis, Postmenopausal | 3.56 |
| CEACAM7 | Osteoporosis, Postmenopausal | 3.56 |
| CGB7 | Osteoporosis, Postmenopausal | 3.56 |
| CYR61 | Osteoporosis, Postmenopausal | 3.56 |
| DGCR5 | Osteoporosis, Postmenopausal | 3.56 |
| DNMT3BA | Osteoporosis, Postmenopausal | 3.56 |
| ERO1L | Osteoporosis, Postmenopausal | 3.56 |
| FAM35A | Osteoporosis, Postmenopausal | 3.56 |
| GSTA6 | Osteoporosis, Postmenopausal | 3.56 |
| H2-T24 | Osteoporosis, Postmenopausal | 3.56 |
| HLA-H | Osteoporosis, Postmenopausal | 3.56 |
| ICK | Osteoporosis, Postmenopausal | 3.56 |
| KLK1B22 | Osteoporosis, Postmenopausal | 3.56 |
| KLRI1 | Osteoporosis, Postmenopausal | 3.56 |
| KRT18P55 | Osteoporosis, Postmenopausal | 3.56 |
| KRTAP1-3 | Osteoporosis, Postmenopausal | 3.56 |
| LILRC2 | Osteoporosis, Postmenopausal | 3.56 |
| LINC00624 | Osteoporosis, Postmenopausal | 3.56 |
| LINC00847 | Osteoporosis, Postmenopausal | 3.56 |
| MIR660 | Osteoporosis, Postmenopausal | 3.56 |
| NKX2-9 | Osteoporosis, Postmenopausal | 3.56 |
| NMUR2 | Osteoporosis, Postmenopausal | 3.56 |
| NPHP3-ACAD11 | Osteoporosis, Postmenopausal | 3.56 |
| OAS1K | Osteoporosis, Postmenopausal | 3.56 |
| OR3A1 | Osteoporosis, Postmenopausal | 3.56 |
| OR51B6 | Osteoporosis, Postmenopausal | 3.56 |
| OR5AS1 | Osteoporosis, Postmenopausal | 3.56 |
| PFN1P2 | Osteoporosis, Postmenopausal | 3.56 |
| PLA2G2C | Osteoporosis, Postmenopausal | 3.56 |
| PNP5B | Osteoporosis, Postmenopausal | 3.56 |
| POM121L8P | Osteoporosis, Postmenopausal | 3.56 |
| PRICKLE2-AS3 | Osteoporosis, Postmenopausal | 3.56 |
| RHEX | Osteoporosis, Postmenopausal | 3.56 |
| RHOXF1 | Osteoporosis, Postmenopausal | 3.56 |
| RLBP1A | Osteoporosis, Postmenopausal | 3.56 |
| RNF224 | Osteoporosis, Postmenopausal | 3.56 |
| RPL13AP5 | Osteoporosis, Postmenopausal | 3.56 |
| SERPINF2B | Osteoporosis, Postmenopausal | 3.56 |
| SLC37A4A | Osteoporosis, Postmenopausal | 3.56 |
| SLC6A1A | Osteoporosis, Postmenopausal | 3.56 |
| SLFN9 | Osteoporosis, Postmenopausal | 3.56 |
| SMCO2 | Osteoporosis, Postmenopausal | 3.56 |
| SPAG11A | Osteoporosis, Postmenopausal | 3.56 |
| STFA2L2 | Osteoporosis, Postmenopausal | 3.56 |
| TRAPPC3L | Osteoporosis, Postmenopausal | 3.56 |
| WDR32 | Osteoporosis, Postmenopausal | 3.56 |
| ZFP496 | Osteoporosis, Postmenopausal | 3.56 |
| ZNF793 | Osteoporosis, Postmenopausal | 3.56 |
| ACVRL1 | Osteoporosis, Postmenopausal | 3.55 |
| C16ORF87 | Osteoporosis, Postmenopausal | 3.55 |
| CXCL17 | Osteoporosis, Postmenopausal | 3.55 |
| HPR | Osteoporosis, Postmenopausal | 3.55 |
| RBM23 | Osteoporosis, Postmenopausal | 3.55 |
| SLITRK3 | Osteoporosis, Postmenopausal | 3.55 |
| ACTL9 | Osteoporosis, Postmenopausal | 3.54 |
| ARSL | Osteoporosis, Postmenopausal | 3.54 |
| BSX | Osteoporosis, Postmenopausal | 3.54 |
| CAR5B | Osteoporosis, Postmenopausal | 3.54 |
| CCDC70 | Osteoporosis, Postmenopausal | 3.54 |
| CCNP | Osteoporosis, Postmenopausal | 3.54 |
| CIB3 | Osteoporosis, Postmenopausal | 3.54 |
| CLRN1 | Osteoporosis, Postmenopausal | 3.54 |
| DPPA4 | Osteoporosis, Postmenopausal | 3.54 |
| ERICH2 | Osteoporosis, Postmenopausal | 3.54 |
| FCER2A | Osteoporosis, Postmenopausal | 3.54 |
| FRMD1 | Osteoporosis, Postmenopausal | 3.54 |
| GARIN3 | Osteoporosis, Postmenopausal | 3.54 |
| KCNH6 | Osteoporosis, Postmenopausal | 3.54 |
| KRTAP5-4 | Osteoporosis, Postmenopausal | 3.54 |
| MIAT | Osteoporosis, Postmenopausal | 3.54 |
| MIR101B | Osteoporosis, Postmenopausal | 3.54 |
| MIR155HG | Osteoporosis, Postmenopausal | 3.54 |
| MIR590 | Osteoporosis, Postmenopausal | 3.54 |
| NAIP5 | Osteoporosis, Postmenopausal | 3.54 |
| NT5DC4 | Osteoporosis, Postmenopausal | 3.54 |
| PAX6 | Osteoporosis, Postmenopausal | 3.54 |
| PBLD2 | Osteoporosis, Postmenopausal | 3.54 |
| PCDHB6 | Osteoporosis, Postmenopausal | 3.54 |
| PRMT9 | Osteoporosis, Postmenopausal | 3.54 |
| RAX2 | Osteoporosis, Postmenopausal | 3.54 |
| RNF222 | Osteoporosis, Postmenopausal | 3.54 |
| RSPO4 | Osteoporosis, Postmenopausal | 3.54 |
| S100PBP | Osteoporosis, Postmenopausal | 3.54 |
| SNORA28 | Osteoporosis, Postmenopausal | 3.54 |
| SPANXC | Osteoporosis, Postmenopausal | 3.54 |
| SPATA3 | Osteoporosis, Postmenopausal | 3.54 |
| TAC2 | Osteoporosis, Postmenopausal | 3.54 |
| TRPA1 | Osteoporosis, Postmenopausal | 3.54 |
| WDR88 | Osteoporosis, Postmenopausal | 3.54 |
| WNT8A | Osteoporosis, Postmenopausal | 3.54 |
| ZNF248 | Osteoporosis, Postmenopausal | 3.54 |
| ZNF337 | Osteoporosis, Postmenopausal | 3.54 |
| ZNF555 | Osteoporosis, Postmenopausal | 3.54 |
| ZNF639 | Osteoporosis, Postmenopausal | 3.54 |
| ZNG1C | Osteoporosis, Postmenopausal | 3.54 |
| F12 | Osteoporosis, Postmenopausal | 3.53 |
| ACAT2L1 | Osteoporosis, Postmenopausal | 3.52 |
| ACTA1A | Osteoporosis, Postmenopausal | 3.52 |
| AGAP4 | Osteoporosis, Postmenopausal | 3.52 |
| AGAP6 | Osteoporosis, Postmenopausal | 3.52 |
| ALDOAB | Osteoporosis, Postmenopausal | 3.52 |
| ALKAL2 | Osteoporosis, Postmenopausal | 3.52 |
| APEG3 | Osteoporosis, Postmenopausal | 3.52 |
| APOBB.2 | Osteoporosis, Postmenopausal | 3.52 |
| COL9A1A | Osteoporosis, Postmenopausal | 3.52 |
| CYP11B | Osteoporosis, Postmenopausal | 3.52 |
| DAO.3 | Osteoporosis, Postmenopausal | 3.52 |
| DGKK | Osteoporosis, Postmenopausal | 3.52 |
| DLX3B | Osteoporosis, Postmenopausal | 3.52 |
| DOPEY2 | Osteoporosis, Postmenopausal | 3.52 |
| FAM160A1 | Osteoporosis, Postmenopausal | 3.52 |
| FAM198A | Osteoporosis, Postmenopausal | 3.52 |
| FAM208A | Osteoporosis, Postmenopausal | 3.52 |
| FLVCR2B | Osteoporosis, Postmenopausal | 3.52 |
| GAPT | Osteoporosis, Postmenopausal | 3.52 |
| GPIB | Osteoporosis, Postmenopausal | 3.52 |
| GRHPRB | Osteoporosis, Postmenopausal | 3.52 |
| GZMC | Osteoporosis, Postmenopausal | 3.52 |
| H1F6 | Osteoporosis, Postmenopausal | 3.52 |
| H2-M2 | Osteoporosis, Postmenopausal | 3.52 |
| HAUS5 | Osteoporosis, Postmenopausal | 3.52 |
| HLA-J | Osteoporosis, Postmenopausal | 3.52 |
| HOXA11-AS | Osteoporosis, Postmenopausal | 3.52 |
| IFI205 | Osteoporosis, Postmenopausal | 3.52 |
| IFNG1 | Osteoporosis, Postmenopausal | 3.52 |
| IGKV2-112L2 | Osteoporosis, Postmenopausal | 3.52 |
| KIR2DL3 | Osteoporosis, Postmenopausal | 3.52 |
| KRT24 | Osteoporosis, Postmenopausal | 3.52 |
| KRT42 | Osteoporosis, Postmenopausal | 3.52 |
| LILRB5 | Osteoporosis, Postmenopausal | 3.52 |
| LINC01559 | Osteoporosis, Postmenopausal | 3.52 |
| LINC02593 | Osteoporosis, Postmenopausal | 3.52 |
| LRCOL1 | Osteoporosis, Postmenopausal | 3.52 |
| MACROH2A1 | Osteoporosis, Postmenopausal | 3.52 |
| MAGED4 | Osteoporosis, Postmenopausal | 3.52 |
| MGL2 | Osteoporosis, Postmenopausal | 3.52 |
| MK1 | Osteoporosis, Postmenopausal | 3.52 |
| NAIP6 | Osteoporosis, Postmenopausal | 3.52 |
| NAT8F3 | Osteoporosis, Postmenopausal | 3.52 |
| NBPF20 | Osteoporosis, Postmenopausal | 3.52 |
| NBPF8 | Osteoporosis, Postmenopausal | 3.52 |
| NOMO2 | Osteoporosis, Postmenopausal | 3.52 |
| NPIPB15 | Osteoporosis, Postmenopausal | 3.52 |
| OR9S13 | Osteoporosis, Postmenopausal | 3.52 |
| PABPC1A | Osteoporosis, Postmenopausal | 3.52 |
| PITPNAA | Osteoporosis, Postmenopausal | 3.52 |
| PPEF1 | Osteoporosis, Postmenopausal | 3.52 |
| RAB11B-AS1 | Osteoporosis, Postmenopausal | 3.52 |
| RAD21L1 | Osteoporosis, Postmenopausal | 3.52 |
| RBP4L | Osteoporosis, Postmenopausal | 3.52 |
| RGSL2H | Osteoporosis, Postmenopausal | 3.52 |
| RHCGA | Osteoporosis, Postmenopausal | 3.52 |
| RNF103-CHMP3 | Osteoporosis, Postmenopausal | 3.52 |
| RT1-M2 | Osteoporosis, Postmenopausal | 3.52 |
| SERPINB11 | Osteoporosis, Postmenopausal | 3.52 |
| SERPINB6C | Osteoporosis, Postmenopausal | 3.52 |
| SERTM1 | Osteoporosis, Postmenopausal | 3.52 |
| SETSIP | Osteoporosis, Postmenopausal | 3.52 |
| SKIA | Osteoporosis, Postmenopausal | 3.52 |
| SLCO1A8 | Osteoporosis, Postmenopausal | 3.52 |
| TAFA4 | Osteoporosis, Postmenopausal | 3.52 |
| TAL2 | Osteoporosis, Postmenopausal | 3.52 |
| TMPRSS11G | Osteoporosis, Postmenopausal | 3.52 |
| TRGC1 | Osteoporosis, Postmenopausal | 3.52 |
| TRP73 | Osteoporosis, Postmenopausal | 3.52 |
| WDR53 | Osteoporosis, Postmenopausal | 3.52 |
| ZFP330 | Osteoporosis, Postmenopausal | 3.52 |
| ZNF138 | Osteoporosis, Postmenopausal | 3.52 |
| ZNF227 | Osteoporosis, Postmenopausal | 3.52 |
| ZNF730 | Osteoporosis, Postmenopausal | 3.52 |
| ZNF783 | Osteoporosis, Postmenopausal | 3.52 |
| CAPN11 | Osteoporosis, Postmenopausal | 3.51 |
| F8A1 | Osteoporosis, Postmenopausal | 3.51 |
| GADD45AA | Osteoporosis, Postmenopausal | 3.51 |
| GJD4 | Osteoporosis, Postmenopausal | 3.51 |
| GTF2IRD2B | Osteoporosis, Postmenopausal | 3.51 |
| HNRNPCL1 | Osteoporosis, Postmenopausal | 3.51 |
| IFNL2 | Osteoporosis, Postmenopausal | 3.51 |
| LINC01587 | Osteoporosis, Postmenopausal | 3.51 |
| MAGEA3 | Osteoporosis, Postmenopausal | 3.51 |
| MIR100HG | Osteoporosis, Postmenopausal | 3.51 |
| NR2C2AP | Osteoporosis, Postmenopausal | 3.51 |
| OCEL1 | Osteoporosis, Postmenopausal | 3.51 |
| OR8B12 | Osteoporosis, Postmenopausal | 3.51 |
| PPFIA2 | Osteoporosis, Postmenopausal | 3.51 |
| PPP2R1A | Osteoporosis, Postmenopausal | 3.51 |
| RABL2B | Osteoporosis, Postmenopausal | 3.51 |
| RGD1309362 | Osteoporosis, Postmenopausal | 3.51 |
| SCAND2P | Osteoporosis, Postmenopausal | 3.51 |
| SEPTIN14 | Osteoporosis, Postmenopausal | 3.51 |
| SERPINA1B | Osteoporosis, Postmenopausal | 3.51 |
| SMCO1 | Osteoporosis, Postmenopausal | 3.51 |
| SOHLH2 | Osteoporosis, Postmenopausal | 3.51 |
| SSPOP | Osteoporosis, Postmenopausal | 3.51 |
| STOML1 | Osteoporosis, Postmenopausal | 3.51 |
| STRA6L | Osteoporosis, Postmenopausal | 3.51 |
| SUMO4 | Osteoporosis, Postmenopausal | 3.51 |
| TNFRSF26 | Osteoporosis, Postmenopausal | 3.51 |
| TTC6 | Osteoporosis, Postmenopausal | 3.51 |
| WRAP53 | Osteoporosis, Postmenopausal | 3.51 |
| ZKSCAN7 | Osteoporosis, Postmenopausal | 3.51 |
| ZNF322 | Osteoporosis, Postmenopausal | 3.51 |
| ZNF41 | Osteoporosis, Postmenopausal | 3.51 |
| ZNF461 | Osteoporosis, Postmenopausal | 3.51 |
| ZNF541 | Osteoporosis, Postmenopausal | 3.51 |
| ZNF558 | Osteoporosis, Postmenopausal | 3.51 |
| ZNF626 | Osteoporosis, Postmenopausal | 3.51 |
| ZNF75D | Osteoporosis, Postmenopausal | 3.51 |
| ZNF841 | Osteoporosis, Postmenopausal | 3.51 |
| ASNSD1 | Osteoporosis, Postmenopausal | 3.5 |
| C4ORF3 | Osteoporosis, Postmenopausal | 3.5 |
| CRYGS | Osteoporosis, Postmenopausal | 3.5 |
| FAM43B | Osteoporosis, Postmenopausal | 3.5 |
| FOCAD | Osteoporosis, Postmenopausal | 3.5 |
| RIPOR3 | Osteoporosis, Postmenopausal | 3.5 |
| SMIM20 | Osteoporosis, Postmenopausal | 3.5 |
| SPATA33 | Osteoporosis, Postmenopausal | 3.5 |
| SYNPO2L | Osteoporosis, Postmenopausal | 3.5 |
| TGFB1A | Osteoporosis, Postmenopausal | 3.5 |
| ZNF581 | Osteoporosis, Postmenopausal | 3.5 |
| ZNF600 | Osteoporosis, Postmenopausal | 3.5 |
| ADCK1 | Osteoporosis, Postmenopausal | 3.49 |
| CD160 | Osteoporosis, Postmenopausal | 3.49 |
| CFAP276 | Osteoporosis, Postmenopausal | 3.49 |
| CLEC12B | Osteoporosis, Postmenopausal | 3.49 |
| CRISP2 | Osteoporosis, Postmenopausal | 3.49 |
| FAAP24 | Osteoporosis, Postmenopausal | 3.49 |
| GARIN4 | Osteoporosis, Postmenopausal | 3.49 |
| GCM2 | Osteoporosis, Postmenopausal | 3.49 |
| GJA10 | Osteoporosis, Postmenopausal | 3.49 |
| GSC2 | Osteoporosis, Postmenopausal | 3.49 |
| GUSBP4 | Osteoporosis, Postmenopausal | 3.49 |
| HSP-16.2 | Osteoporosis, Postmenopausal | 3.49 |
| IGFL1 | Osteoporosis, Postmenopausal | 3.49 |
| KCNK16 | Osteoporosis, Postmenopausal | 3.49 |
| KIF24 | Osteoporosis, Postmenopausal | 3.49 |
| KLK15 | Osteoporosis, Postmenopausal | 3.49 |
| LCN15 | Osteoporosis, Postmenopausal | 3.49 |
| MIR301B | Osteoporosis, Postmenopausal | 3.49 |
| MIR30C1 | Osteoporosis, Postmenopausal | 3.49 |
| MIR30C2 | Osteoporosis, Postmenopausal | 3.49 |
| MIR376B | Osteoporosis, Postmenopausal | 3.49 |
| MROH8 | Osteoporosis, Postmenopausal | 3.49 |
| NPIPA1 | Osteoporosis, Postmenopausal | 3.49 |
| PLPP4 | Osteoporosis, Postmenopausal | 3.49 |
| PTPRV | Osteoporosis, Postmenopausal | 3.49 |
| RNF225 | Osteoporosis, Postmenopausal | 3.49 |
| SCD3 | Osteoporosis, Postmenopausal | 3.49 |
| SHOX | Osteoporosis, Postmenopausal | 3.49 |
| SLC35G3 | Osteoporosis, Postmenopausal | 3.49 |
| SMCO3 | Osteoporosis, Postmenopausal | 3.49 |
| SPMIP9 | Osteoporosis, Postmenopausal | 3.49 |
| SULT2A8 | Osteoporosis, Postmenopausal | 3.49 |
| SVOPL | Osteoporosis, Postmenopausal | 3.49 |
| TSPO2 | Osteoporosis, Postmenopausal | 3.49 |
| TULP2 | Osteoporosis, Postmenopausal | 3.49 |
| ZFP704 | Osteoporosis, Postmenopausal | 3.49 |
| ZNF181 | Osteoporosis, Postmenopausal | 3.49 |
| ZNF211 | Osteoporosis, Postmenopausal | 3.49 |
| ZNF252P | Osteoporosis, Postmenopausal | 3.49 |
| ZNF699 | Osteoporosis, Postmenopausal | 3.49 |
| ZNF763 | Osteoporosis, Postmenopausal | 3.49 |
| ACTL8 | Osteoporosis, Postmenopausal | 3.48 |
| ANKUB1 | Osteoporosis, Postmenopausal | 3.48 |
| ANXA1C | Osteoporosis, Postmenopausal | 3.48 |
| ANXA5B | Osteoporosis, Postmenopausal | 3.48 |
| APOA4B.2 | Osteoporosis, Postmenopausal | 3.48 |
| ARNTL | Osteoporosis, Postmenopausal | 3.48 |
| BTBD18 | Osteoporosis, Postmenopausal | 3.48 |
| CBLN2 | Osteoporosis, Postmenopausal | 3.48 |
| CCDC185 | Osteoporosis, Postmenopausal | 3.48 |
| CEP57L1 | Osteoporosis, Postmenopausal | 3.48 |
| COX4I1L | Osteoporosis, Postmenopausal | 3.48 |
| ECT2L | Osteoporosis, Postmenopausal | 3.48 |
| EEF1AKMT4 | Osteoporosis, Postmenopausal | 3.48 |
| GADD45AB | Osteoporosis, Postmenopausal | 3.48 |
| GSTR | Osteoporosis, Postmenopausal | 3.48 |
| HHLA2 | Osteoporosis, Postmenopausal | 3.48 |
| HLA-DRB3 | Osteoporosis, Postmenopausal | 3.48 |
| IFFO1 | Osteoporosis, Postmenopausal | 3.48 |
| LINC00842 | Osteoporosis, Postmenopausal | 3.48 |
| LRRC37A | Osteoporosis, Postmenopausal | 3.48 |
| LY49SI2 | Osteoporosis, Postmenopausal | 3.48 |
| MIBP | Osteoporosis, Postmenopausal | 3.48 |
| MIR383 | Osteoporosis, Postmenopausal | 3.48 |
| MUC17 | Osteoporosis, Postmenopausal | 3.48 |
| MUM1L1 | Osteoporosis, Postmenopausal | 3.48 |
| NAPSB | Osteoporosis, Postmenopausal | 3.48 |
| NIFK-AS1 | Osteoporosis, Postmenopausal | 3.48 |
| NME8 | Osteoporosis, Postmenopausal | 3.48 |
| NOMO3 | Osteoporosis, Postmenopausal | 3.48 |
| OBP1F | Osteoporosis, Postmenopausal | 3.48 |
| PITX1-AS1 | Osteoporosis, Postmenopausal | 3.48 |
| PMFBP1 | Osteoporosis, Postmenopausal | 3.48 |
| PRKAG2-AS1 | Osteoporosis, Postmenopausal | 3.48 |
| RNF133 | Osteoporosis, Postmenopausal | 3.48 |
| S100A10A | Osteoporosis, Postmenopausal | 3.48 |
| SBDSP1 | Osteoporosis, Postmenopausal | 3.48 |
| SCGB2A1 | Osteoporosis, Postmenopausal | 3.48 |
| SIRPD | Osteoporosis, Postmenopausal | 3.48 |
| SLC22A24 | Osteoporosis, Postmenopausal | 3.48 |
| SNORA14B | Osteoporosis, Postmenopausal | 3.48 |
| SPRR2J-PS | Osteoporosis, Postmenopausal | 3.48 |
| SRD5A2A | Osteoporosis, Postmenopausal | 3.48 |
| STMN1A | Osteoporosis, Postmenopausal | 3.48 |
| STMP1 | Osteoporosis, Postmenopausal | 3.48 |
| TDO2A | Osteoporosis, Postmenopausal | 3.48 |
| TEDDM3 | Osteoporosis, Postmenopausal | 3.48 |
| TMPPE | Osteoporosis, Postmenopausal | 3.48 |
| TNFRSF17 | Osteoporosis, Postmenopausal | 3.48 |
| TUBA3C | Osteoporosis, Postmenopausal | 3.48 |
| VWA3B | Osteoporosis, Postmenopausal | 3.48 |
| WFIKKN2 | Osteoporosis, Postmenopausal | 3.48 |
| WISP2 | Osteoporosis, Postmenopausal | 3.48 |
| ZP2.3 | Osteoporosis, Postmenopausal | 3.48 |
| C12ORF75 | Osteoporosis, Postmenopausal | 3.47 |
| CCDC81 | Osteoporosis, Postmenopausal | 3.47 |
| CD200 | Osteoporosis, Postmenopausal | 3.47 |
| ECHS1 | Osteoporosis, Postmenopausal | 3.47 |
| IQCA1 | Osteoporosis, Postmenopausal | 3.47 |
| KIAA1671 | Osteoporosis, Postmenopausal | 3.47 |
| KRTCAP3 | Osteoporosis, Postmenopausal | 3.47 |
| LIN9 | Osteoporosis, Postmenopausal | 3.47 |
| NUDT18 | Osteoporosis, Postmenopausal | 3.47 |
| PSMA1 | Osteoporosis, Postmenopausal | 3.47 |
| SLC39A3 | Osteoporosis, Postmenopausal | 3.47 |
| UBAP1L | Osteoporosis, Postmenopausal | 3.47 |
| USP5 | Osteoporosis, Postmenopausal | 3.47 |
| ZNF260 | Osteoporosis, Postmenopausal | 3.47 |
| ZNF37BP | Osteoporosis, Postmenopausal | 3.47 |
| ZXDB | Osteoporosis, Postmenopausal | 3.47 |
| ACTL10 | Osteoporosis, Postmenopausal | 3.46 |
| ANKRD18A | Osteoporosis, Postmenopausal | 3.46 |
| C10ORF88 | Osteoporosis, Postmenopausal | 3.46 |
| C11ORF87 | Osteoporosis, Postmenopausal | 3.46 |
| C1ORF50 | Osteoporosis, Postmenopausal | 3.46 |
| C1ORF56 | Osteoporosis, Postmenopausal | 3.46 |
| CCL3L1 | Osteoporosis, Postmenopausal | 3.46 |
| CIMAP1D | Osteoporosis, Postmenopausal | 3.46 |
| CPSF4L | Osteoporosis, Postmenopausal | 3.46 |
| CYP2A12 | Osteoporosis, Postmenopausal | 3.46 |
| DUSP28 | Osteoporosis, Postmenopausal | 3.46 |
| HELT | Osteoporosis, Postmenopausal | 3.46 |
| IFITM5 | Osteoporosis, Postmenopausal | 3.46 |
| IFNA | Osteoporosis, Postmenopausal | 3.46 |
| KCTD8 | Osteoporosis, Postmenopausal | 3.46 |
| KLHL33 | Osteoporosis, Postmenopausal | 3.46 |
| LHFPL3 | Osteoporosis, Postmenopausal | 3.46 |
| MEI1 | Osteoporosis, Postmenopausal | 3.46 |
| MIR421 | Osteoporosis, Postmenopausal | 3.46 |
| MIR487B | Osteoporosis, Postmenopausal | 3.46 |
| MMP23B | Osteoporosis, Postmenopausal | 3.46 |
| NLRP7 | Osteoporosis, Postmenopausal | 3.46 |
| OR2L13 | Osteoporosis, Postmenopausal | 3.46 |
| PKD1L3 | Osteoporosis, Postmenopausal | 3.46 |
| SFTPA2 | Osteoporosis, Postmenopausal | 3.46 |
| SLAMF6 | Osteoporosis, Postmenopausal | 3.46 |
| SLC2A7 | Osteoporosis, Postmenopausal | 3.46 |
| SRP54A | Osteoporosis, Postmenopausal | 3.46 |
| SYNGR4 | Osteoporosis, Postmenopausal | 3.46 |
| TREML1 | Osteoporosis, Postmenopausal | 3.46 |
| UBTD1 | Osteoporosis, Postmenopausal | 3.46 |
| ZNF250 | Osteoporosis, Postmenopausal | 3.46 |
| ZNF575 | Osteoporosis, Postmenopausal | 3.46 |
| ZNF771 | Osteoporosis, Postmenopausal | 3.46 |
| ZNF813 | Osteoporosis, Postmenopausal | 3.46 |
| ACP5A | Osteoporosis, Postmenopausal | 3.45 |
| ACTB2 | Osteoporosis, Postmenopausal | 3.45 |
| ADH6A | Osteoporosis, Postmenopausal | 3.45 |
| ADH8A | Osteoporosis, Postmenopausal | 3.45 |
| APOA4B.3 | Osteoporosis, Postmenopausal | 3.45 |
| ATP2B1-AS1 | Osteoporosis, Postmenopausal | 3.45 |
| AWAT2 | Osteoporosis, Postmenopausal | 3.45 |
| BRS3 | Osteoporosis, Postmenopausal | 3.45 |
| CFAP99 | Osteoporosis, Postmenopausal | 3.45 |
| CHS1 | Osteoporosis, Postmenopausal | 3.45 |
| CLPS | Osteoporosis, Postmenopausal | 3.45 |
| CTXN3 | Osteoporosis, Postmenopausal | 3.45 |
| CYP4F5 | Osteoporosis, Postmenopausal | 3.45 |
| DRC12 | Osteoporosis, Postmenopausal | 3.45 |
| ENO1B | Osteoporosis, Postmenopausal | 3.45 |
| FAM110A | Osteoporosis, Postmenopausal | 3.45 |
| FAM64A | Osteoporosis, Postmenopausal | 3.45 |
| FGFBP2 | Osteoporosis, Postmenopausal | 3.45 |
| FNDC3C1 | Osteoporosis, Postmenopausal | 3.45 |
| FOXB1 | Osteoporosis, Postmenopausal | 3.45 |
| GRIN1B | Osteoporosis, Postmenopausal | 3.45 |
| HLA-DRB6 | Osteoporosis, Postmenopausal | 3.45 |
| HTR3A | Osteoporosis, Postmenopausal | 3.45 |
| IDI2 | Osteoporosis, Postmenopausal | 3.45 |
| IFI214 | Osteoporosis, Postmenopausal | 3.45 |
| IFIT1BL1 | Osteoporosis, Postmenopausal | 3.45 |
| KIAA0408 | Osteoporosis, Postmenopausal | 3.45 |
| KLK11 | Osteoporosis, Postmenopausal | 3.45 |
| LEPA | Osteoporosis, Postmenopausal | 3.45 |
| MS4A6E | Osteoporosis, Postmenopausal | 3.45 |
| MST1P2 | Osteoporosis, Postmenopausal | 3.45 |
| PPARAA | Osteoporosis, Postmenopausal | 3.45 |
| RARRES3 | Osteoporosis, Postmenopausal | 3.45 |
| SCGB1C1 | Osteoporosis, Postmenopausal | 3.45 |
| SDR16C5 | Osteoporosis, Postmenopausal | 3.45 |
| SLC66A1 | Osteoporosis, Postmenopausal | 3.45 |
| SPACA4 | Osteoporosis, Postmenopausal | 3.45 |
| SPINK7 | Osteoporosis, Postmenopausal | 3.45 |
| SPINT3 | Osteoporosis, Postmenopausal | 3.45 |
| TAS2R119 | Osteoporosis, Postmenopausal | 3.45 |
| TMEM235 | Osteoporosis, Postmenopausal | 3.45 |
| TSIX | Osteoporosis, Postmenopausal | 3.45 |
| TSPOAP1-AS1 | Osteoporosis, Postmenopausal | 3.45 |
| UNC5B-AS1 | Osteoporosis, Postmenopausal | 3.45 |
| VSIG4 | Osteoporosis, Postmenopausal | 3.45 |
| ZFP521 | Osteoporosis, Postmenopausal | 3.45 |
| ZNF774 | Osteoporosis, Postmenopausal | 3.45 |
| ADAMTS20 | Osteoporosis, Postmenopausal | 3.44 |
| AMZ2P1 | Osteoporosis, Postmenopausal | 3.44 |
| BOD1L | Osteoporosis, Postmenopausal | 3.44 |
| C7ORF50 | Osteoporosis, Postmenopausal | 3.44 |
| CRNN | Osteoporosis, Postmenopausal | 3.44 |
| EOLA2 | Osteoporosis, Postmenopausal | 3.44 |
| FAM81A | Osteoporosis, Postmenopausal | 3.44 |
| FTHL17 | Osteoporosis, Postmenopausal | 3.44 |
| GRAMD4 | Osteoporosis, Postmenopausal | 3.44 |
| HEATR4 | Osteoporosis, Postmenopausal | 3.44 |
| HOXD12 | Osteoporosis, Postmenopausal | 3.44 |
| INAFM1 | Osteoporosis, Postmenopausal | 3.44 |
| LBX1 | Osteoporosis, Postmenopausal | 3.44 |
| LRRIQ3 | Osteoporosis, Postmenopausal | 3.44 |
| MIR106A | Osteoporosis, Postmenopausal | 3.44 |
| OR51E2 | Osteoporosis, Postmenopausal | 3.44 |
| PPIP5K1 | Osteoporosis, Postmenopausal | 3.44 |
| PRKY | Osteoporosis, Postmenopausal | 3.44 |
| PTH2 | Osteoporosis, Postmenopausal | 3.44 |
| RT1-DMA | Osteoporosis, Postmenopausal | 3.44 |
| SELENOO | Osteoporosis, Postmenopausal | 3.44 |
| TRIM4 | Osteoporosis, Postmenopausal | 3.44 |
| UBR7 | Osteoporosis, Postmenopausal | 3.44 |
| YJU2B | Osteoporosis, Postmenopausal | 3.44 |
| ZNF212 | Osteoporosis, Postmenopausal | 3.44 |
| ZNF30 | Osteoporosis, Postmenopausal | 3.44 |
| ZNF318 | Osteoporosis, Postmenopausal | 3.44 |
| ZNF320 | Osteoporosis, Postmenopausal | 3.44 |
| ZNF347 | Osteoporosis, Postmenopausal | 3.44 |
| ZNF649 | Osteoporosis, Postmenopausal | 3.44 |
| ZNF747 | Osteoporosis, Postmenopausal | 3.44 |
| ADAM7 | Osteoporosis, Postmenopausal | 3.43 |
| HAUS7 | Osteoporosis, Postmenopausal | 3.43 |
| HEMGN | Osteoporosis, Postmenopausal | 3.43 |
| LRRC45 | Osteoporosis, Postmenopausal | 3.43 |
| NLRP10 | Osteoporosis, Postmenopausal | 3.43 |
| PCNX4 | Osteoporosis, Postmenopausal | 3.43 |
| PIH1D2 | Osteoporosis, Postmenopausal | 3.43 |
| SLC39A13 | Osteoporosis, Postmenopausal | 3.43 |
| SMIM29 | Osteoporosis, Postmenopausal | 3.43 |
| ACTR8 | Osteoporosis, Postmenopausal | 3.42 |
| ADGRF3 | Osteoporosis, Postmenopausal | 3.42 |
| ATOH7 | Osteoporosis, Postmenopausal | 3.42 |
| CCSAP | Osteoporosis, Postmenopausal | 3.42 |
| CRYGA | Osteoporosis, Postmenopausal | 3.42 |
| CSPRS | Osteoporosis, Postmenopausal | 3.42 |
| CYB561D2 | Osteoporosis, Postmenopausal | 3.42 |
| EEF1AKMT1 | Osteoporosis, Postmenopausal | 3.42 |
| EVC | Osteoporosis, Postmenopausal | 3.42 |
| FEZF1 | Osteoporosis, Postmenopausal | 3.42 |
| GSX1 | Osteoporosis, Postmenopausal | 3.42 |
| HIPK4 | Osteoporosis, Postmenopausal | 3.42 |
| IGF2-AS | Osteoporosis, Postmenopausal | 3.42 |
| ITPRIPL1 | Osteoporosis, Postmenopausal | 3.42 |
| KLHL40 | Osteoporosis, Postmenopausal | 3.42 |
| LHX5 | Osteoporosis, Postmenopausal | 3.42 |
| LIPT1 | Osteoporosis, Postmenopausal | 3.42 |
| MFSD14B | Osteoporosis, Postmenopausal | 3.42 |
| MUP21 | Osteoporosis, Postmenopausal | 3.42 |
| OPN5 | Osteoporosis, Postmenopausal | 3.42 |
| PRSS41 | Osteoporosis, Postmenopausal | 3.42 |
| PTCHD1 | Osteoporosis, Postmenopausal | 3.42 |
| RHAG | Osteoporosis, Postmenopausal | 3.42 |
| RIPOR1 | Osteoporosis, Postmenopausal | 3.42 |
| RIPPLY1 | Osteoporosis, Postmenopausal | 3.42 |
| RTBDN | Osteoporosis, Postmenopausal | 3.42 |
| SHE | Osteoporosis, Postmenopausal | 3.42 |
| SLX4IP | Osteoporosis, Postmenopausal | 3.42 |
| TCP10L | Osteoporosis, Postmenopausal | 3.42 |
| TEX13B | Osteoporosis, Postmenopausal | 3.42 |
| THSD7B | Osteoporosis, Postmenopausal | 3.42 |
| TMPRSS7 | Osteoporosis, Postmenopausal | 3.42 |
| ZNF189 | Osteoporosis, Postmenopausal | 3.42 |
| ZNF23 | Osteoporosis, Postmenopausal | 3.42 |
| ZNF423 | Osteoporosis, Postmenopausal | 3.42 |
| ZNF561 | Osteoporosis, Postmenopausal | 3.42 |
| ZNF740 | Osteoporosis, Postmenopausal | 3.42 |
| ZNF816 | Osteoporosis, Postmenopausal | 3.42 |
| ZNRF4 | Osteoporosis, Postmenopausal | 3.42 |
| AADACL3 | Osteoporosis, Postmenopausal | 3.41 |
| ALDOAA | Osteoporosis, Postmenopausal | 3.41 |
| ANPEPB.1 | Osteoporosis, Postmenopausal | 3.41 |
| AQP8A.1 | Osteoporosis, Postmenopausal | 3.41 |
| CALHM4 | Osteoporosis, Postmenopausal | 3.41 |
| CATSPERD | Osteoporosis, Postmenopausal | 3.41 |
| CATSPERE | Osteoporosis, Postmenopausal | 3.41 |
| CCL3L3 | Osteoporosis, Postmenopausal | 3.41 |
| CHMP4BL1 | Osteoporosis, Postmenopausal | 3.41 |
| CYLD | Osteoporosis, Postmenopausal | 3.41 |
| CYP2D5 | Osteoporosis, Postmenopausal | 3.41 |
| DAO.1 | Osteoporosis, Postmenopausal | 3.41 |
| DGAT1A | Osteoporosis, Postmenopausal | 3.41 |
| DHRS3B | Osteoporosis, Postmenopausal | 3.41 |
| DPYDB | Osteoporosis, Postmenopausal | 3.41 |
| FAM69C | Osteoporosis, Postmenopausal | 3.41 |
| FBXO39 | Osteoporosis, Postmenopausal | 3.41 |
| FUT8-AS1 | Osteoporosis, Postmenopausal | 3.41 |
| G6PC2 | Osteoporosis, Postmenopausal | 3.41 |
| GSG1L | Osteoporosis, Postmenopausal | 3.41 |
| KLK1B4 | Osteoporosis, Postmenopausal | 3.41 |
| LARS1B | Osteoporosis, Postmenopausal | 3.41 |
| LAYN | Osteoporosis, Postmenopausal | 3.41 |
| LINC00515 | Osteoporosis, Postmenopausal | 3.41 |
| MGST3B | Osteoporosis, Postmenopausal | 3.41 |
| MIR16 | Osteoporosis, Postmenopausal | 3.41 |
| MS4A18 | Osteoporosis, Postmenopausal | 3.41 |
| NID1A | Osteoporosis, Postmenopausal | 3.41 |
| NOLA2 | Osteoporosis, Postmenopausal | 3.41 |
| NOP14-AS1 | Osteoporosis, Postmenopausal | 3.41 |
| OAZ3 | Osteoporosis, Postmenopausal | 3.41 |
| OR6C74 | Osteoporosis, Postmenopausal | 3.41 |
| PAK5 | Osteoporosis, Postmenopausal | 3.41 |
| PPP2R5B | Osteoporosis, Postmenopausal | 3.41 |
| RT1-DOB | Osteoporosis, Postmenopausal | 3.41 |
| SDR42E2 | Osteoporosis, Postmenopausal | 3.41 |
| SHOC1 | Osteoporosis, Postmenopausal | 3.41 |
| SIGLEC15 | Osteoporosis, Postmenopausal | 3.41 |
| SIX6 | Osteoporosis, Postmenopausal | 3.41 |
| SLC16A12B | Osteoporosis, Postmenopausal | 3.41 |
| SNHG18 | Osteoporosis, Postmenopausal | 3.41 |
| SNORD49A | Osteoporosis, Postmenopausal | 3.41 |
| SULT1ST2 | Osteoporosis, Postmenopausal | 3.41 |
| TNFRSFA | Osteoporosis, Postmenopausal | 3.41 |
| TSSK3 | Osteoporosis, Postmenopausal | 3.41 |
| VOF16 | Osteoporosis, Postmenopausal | 3.41 |
| ZNF620 | Osteoporosis, Postmenopausal | 3.41 |
| A | Osteoporosis, Postmenopausal | 3.4 |
| AGAP3 | Osteoporosis, Postmenopausal | 3.4 |
| APBA2 | Osteoporosis, Postmenopausal | 3.4 |
| ARMCX5 | Osteoporosis, Postmenopausal | 3.4 |
| C11ORF86 | Osteoporosis, Postmenopausal | 3.4 |
| C14ORF119 | Osteoporosis, Postmenopausal | 3.4 |
| C18ORF21 | Osteoporosis, Postmenopausal | 3.4 |
| C3ORF62 | Osteoporosis, Postmenopausal | 3.4 |
| C7ORF57 | Osteoporosis, Postmenopausal | 3.4 |
| CD8B | Osteoporosis, Postmenopausal | 3.4 |
| CDCP2 | Osteoporosis, Postmenopausal | 3.4 |
| CHIA | Osteoporosis, Postmenopausal | 3.4 |
| FAM177A1 | Osteoporosis, Postmenopausal | 3.4 |
| GPR153 | Osteoporosis, Postmenopausal | 3.4 |
| HBQ1 | Osteoporosis, Postmenopausal | 3.4 |
| MANBAL | Osteoporosis, Postmenopausal | 3.4 |
| MIR202 | Osteoporosis, Postmenopausal | 3.4 |
| MIR340 | Osteoporosis, Postmenopausal | 3.4 |
| MT1IP | Osteoporosis, Postmenopausal | 3.4 |
| MUP9 | Osteoporosis, Postmenopausal | 3.4 |
| MYO1A | Osteoporosis, Postmenopausal | 3.4 |
| PIP | Osteoporosis, Postmenopausal | 3.4 |
| PLA2G4F | Osteoporosis, Postmenopausal | 3.4 |
| PLK5 | Osteoporosis, Postmenopausal | 3.4 |
| PSG5 | Osteoporosis, Postmenopausal | 3.4 |
| REXO5 | Osteoporosis, Postmenopausal | 3.4 |
| RXFP2 | Osteoporosis, Postmenopausal | 3.4 |
| SLC22A10 | Osteoporosis, Postmenopausal | 3.4 |
| SPATC1 | Osteoporosis, Postmenopausal | 3.4 |
| SPEM1 | Osteoporosis, Postmenopausal | 3.4 |
| STKLD1 | Osteoporosis, Postmenopausal | 3.4 |
| SYTL3 | Osteoporosis, Postmenopausal | 3.4 |
| TDRKH | Osteoporosis, Postmenopausal | 3.4 |
| TMEM132E | Osteoporosis, Postmenopausal | 3.4 |
| TPPP2 | Osteoporosis, Postmenopausal | 3.4 |
| USP6 | Osteoporosis, Postmenopausal | 3.4 |
| WDR24 | Osteoporosis, Postmenopausal | 3.4 |
| ZNF204P | Osteoporosis, Postmenopausal | 3.4 |
| ZNF585B | Osteoporosis, Postmenopausal | 3.4 |
| ZP4 | Osteoporosis, Postmenopausal | 3.4 |
| ARL2BP | Osteoporosis, Postmenopausal | 3.39 |
| CAPN7 | Osteoporosis, Postmenopausal | 3.39 |
| EGLN2 | Osteoporosis, Postmenopausal | 3.39 |
| FOLH1 | Osteoporosis, Postmenopausal | 3.39 |
| GIMAP8 | Osteoporosis, Postmenopausal | 3.39 |
| GPANK1 | Osteoporosis, Postmenopausal | 3.39 |
| GSKIP | Osteoporosis, Postmenopausal | 3.39 |
| HDDC2 | Osteoporosis, Postmenopausal | 3.39 |
| HYCC1 | Osteoporosis, Postmenopausal | 3.39 |
| KTI12 | Osteoporosis, Postmenopausal | 3.39 |
| LIM2 | Osteoporosis, Postmenopausal | 3.39 |
| MAP3K10 | Osteoporosis, Postmenopausal | 3.39 |
| MARCHF1 | Osteoporosis, Postmenopausal | 3.39 |
| RNLS | Osteoporosis, Postmenopausal | 3.39 |
| TMEM87B | Osteoporosis, Postmenopausal | 3.39 |
| TNP1 | Osteoporosis, Postmenopausal | 3.39 |
| ABCG3L3 | Osteoporosis, Postmenopausal | 3.38 |
| ANO2 | Osteoporosis, Postmenopausal | 3.38 |
| ARHGAP30 | Osteoporosis, Postmenopausal | 3.38 |
| ATP6V1E2 | Osteoporosis, Postmenopausal | 3.38 |
| B3GALT4 | Osteoporosis, Postmenopausal | 3.38 |
| BTNL10 | Osteoporosis, Postmenopausal | 3.38 |
| CALML5 | Osteoporosis, Postmenopausal | 3.38 |
| CATSPERB | Osteoporosis, Postmenopausal | 3.38 |
| CATSPERG1 | Osteoporosis, Postmenopausal | 3.38 |
| CFAP126 | Osteoporosis, Postmenopausal | 3.38 |
| CLEC4B2 | Osteoporosis, Postmenopausal | 3.38 |
| CYGB1 | Osteoporosis, Postmenopausal | 3.38 |
| CYP17 | Osteoporosis, Postmenopausal | 3.38 |
| CYP2J10 | Osteoporosis, Postmenopausal | 3.38 |
| CYP2Y3 | Osteoporosis, Postmenopausal | 3.38 |
| DAO.2 | Osteoporosis, Postmenopausal | 3.38 |
| DIRC3 | Osteoporosis, Postmenopausal | 3.38 |
| DIS3L2 | Osteoporosis, Postmenopausal | 3.38 |
| DSEL | Osteoporosis, Postmenopausal | 3.38 |
| EPDL2 | Osteoporosis, Postmenopausal | 3.38 |
| EPS8L1 | Osteoporosis, Postmenopausal | 3.38 |
| ERV3-1 | Osteoporosis, Postmenopausal | 3.38 |
| FAM105A | Osteoporosis, Postmenopausal | 3.38 |
| FAM83H | Osteoporosis, Postmenopausal | 3.38 |
| FAM86DP | Osteoporosis, Postmenopausal | 3.38 |
| FCGBPL1 | Osteoporosis, Postmenopausal | 3.38 |
| FOXF2 | Osteoporosis, Postmenopausal | 3.38 |
| GAREM | Osteoporosis, Postmenopausal | 3.38 |
| GNB3A | Osteoporosis, Postmenopausal | 3.38 |
| GPHA2 | Osteoporosis, Postmenopausal | 3.38 |
| GSTT1A | Osteoporosis, Postmenopausal | 3.38 |
| HYAL4 | Osteoporosis, Postmenopausal | 3.38 |
| ITLN2 | Osteoporosis, Postmenopausal | 3.38 |
| KRBA2 | Osteoporosis, Postmenopausal | 3.38 |
| KRBOX4 | Osteoporosis, Postmenopausal | 3.38 |
| LRP1B | Osteoporosis, Postmenopausal | 3.38 |
| LSMEM2 | Osteoporosis, Postmenopausal | 3.38 |
| LY6I | Osteoporosis, Postmenopausal | 3.38 |
| LYPD8 | Osteoporosis, Postmenopausal | 3.38 |
| MAJIN | Osteoporosis, Postmenopausal | 3.38 |
| MEP1A.1 | Osteoporosis, Postmenopausal | 3.38 |
| MIR551B | Osteoporosis, Postmenopausal | 3.38 |
| MRVI1 | Osteoporosis, Postmenopausal | 3.38 |
| MYO15A | Osteoporosis, Postmenopausal | 3.38 |
| NDNL2 | Osteoporosis, Postmenopausal | 3.38 |
| NKIRAS2 | Osteoporosis, Postmenopausal | 3.38 |
| NP4 | Osteoporosis, Postmenopausal | 3.38 |
| NTMT2 | Osteoporosis, Postmenopausal | 3.38 |
| OR51K2 | Osteoporosis, Postmenopausal | 3.38 |
| PCDHA13 | Osteoporosis, Postmenopausal | 3.38 |
| PCDHGB4 | Osteoporosis, Postmenopausal | 3.38 |
| PCDHGC5 | Osteoporosis, Postmenopausal | 3.38 |
| PPP1R17 | Osteoporosis, Postmenopausal | 3.38 |
| PPP1R1C | Osteoporosis, Postmenopausal | 3.38 |
| PWAR5 | Osteoporosis, Postmenopausal | 3.38 |
| QRICH2 | Osteoporosis, Postmenopausal | 3.38 |
| RNF2 | Osteoporosis, Postmenopausal | 3.38 |
| RNF223 | Osteoporosis, Postmenopausal | 3.38 |
| RT1-T24-3 | Osteoporosis, Postmenopausal | 3.38 |
| SGK2B | Osteoporosis, Postmenopausal | 3.38 |
| SLPI-PS1 | Osteoporosis, Postmenopausal | 3.38 |
| SMIM38 | Osteoporosis, Postmenopausal | 3.38 |
| TOB1-AS1 | Osteoporosis, Postmenopausal | 3.38 |
| TUBA3E | Osteoporosis, Postmenopausal | 3.38 |
| UGT2B34L1 | Osteoporosis, Postmenopausal | 3.38 |
| ZC2HC1C | Osteoporosis, Postmenopausal | 3.38 |
| ZFP169 | Osteoporosis, Postmenopausal | 3.38 |
| ZFP287 | Osteoporosis, Postmenopausal | 3.38 |
| ZNF366 | Osteoporosis, Postmenopausal | 3.38 |
| ZNF573 | Osteoporosis, Postmenopausal | 3.38 |
| ZNF609 | Osteoporosis, Postmenopausal | 3.38 |
| ZNF676 | Osteoporosis, Postmenopausal | 3.38 |
| BXDC2 | Osteoporosis, Postmenopausal | 3.37 |
| C1QL2 | Osteoporosis, Postmenopausal | 3.37 |
| C5ORF22 | Osteoporosis, Postmenopausal | 3.37 |
| CIMIP2A | Osteoporosis, Postmenopausal | 3.37 |
| DOK5 | Osteoporosis, Postmenopausal | 3.37 |
| ERVMER34-1 | Osteoporosis, Postmenopausal | 3.37 |
| HENMT1 | Osteoporosis, Postmenopausal | 3.37 |
| IFNL3 | Osteoporosis, Postmenopausal | 3.37 |
| JAKMIP3 | Osteoporosis, Postmenopausal | 3.37 |
| KLF14 | Osteoporosis, Postmenopausal | 3.37 |
| KRT78 | Osteoporosis, Postmenopausal | 3.37 |
| M1AP | Osteoporosis, Postmenopausal | 3.37 |
| MGAT4C | Osteoporosis, Postmenopausal | 3.37 |
| MPND | Osteoporosis, Postmenopausal | 3.37 |
| NPFFR1 | Osteoporosis, Postmenopausal | 3.37 |
| OPN1SW | Osteoporosis, Postmenopausal | 3.37 |
| PGCP | Osteoporosis, Postmenopausal | 3.37 |
| POU6F2 | Osteoporosis, Postmenopausal | 3.37 |
| SLFNL1 | Osteoporosis, Postmenopausal | 3.37 |
| TECTA | Osteoporosis, Postmenopausal | 3.37 |
| TMEM121 | Osteoporosis, Postmenopausal | 3.37 |
| TVP23C | Osteoporosis, Postmenopausal | 3.37 |
| UBE2U | Osteoporosis, Postmenopausal | 3.37 |
| ZNF223 | Osteoporosis, Postmenopausal | 3.37 |
| ZNF304 | Osteoporosis, Postmenopausal | 3.37 |
| ZNF484 | Osteoporosis, Postmenopausal | 3.37 |
| ZNG1A | Osteoporosis, Postmenopausal | 3.37 |
| 1-SF | Osteoporosis, Postmenopausal | 3.36 |
| ABCB10 | Osteoporosis, Postmenopausal | 3.36 |
| APOA4B.1 | Osteoporosis, Postmenopausal | 3.36 |
| ARSD | Osteoporosis, Postmenopausal | 3.36 |
| C3ORF80 | Osteoporosis, Postmenopausal | 3.36 |
| C5ORF15 | Osteoporosis, Postmenopausal | 3.36 |
| CADM3 | Osteoporosis, Postmenopausal | 3.36 |
| CLUL1 | Osteoporosis, Postmenopausal | 3.36 |
| CRYBGX | Osteoporosis, Postmenopausal | 3.36 |
| CRYGC | Osteoporosis, Postmenopausal | 3.36 |
| DNMT3BB.1 | Osteoporosis, Postmenopausal | 3.36 |
| DPM2 | Osteoporosis, Postmenopausal | 3.36 |
| DPY19L2P2 | Osteoporosis, Postmenopausal | 3.36 |
| ERMARD | Osteoporosis, Postmenopausal | 3.36 |
| FAM86B3P | Osteoporosis, Postmenopausal | 3.36 |
| FCN3 | Osteoporosis, Postmenopausal | 3.36 |
| HMSD | Osteoporosis, Postmenopausal | 3.36 |
| HS3ST4 | Osteoporosis, Postmenopausal | 3.36 |
| HSP-16.1 | Osteoporosis, Postmenopausal | 3.36 |
| KRT1-19E | Osteoporosis, Postmenopausal | 3.36 |
| LGALS1L1 | Osteoporosis, Postmenopausal | 3.36 |
| LGALS2A | Osteoporosis, Postmenopausal | 3.36 |
| LGSN | Osteoporosis, Postmenopausal | 3.36 |
| LINC00339 | Osteoporosis, Postmenopausal | 3.36 |
| LRRC10 | Osteoporosis, Postmenopausal | 3.36 |
| MCIDAS | Osteoporosis, Postmenopausal | 3.36 |
| MINOS1 | Osteoporosis, Postmenopausal | 3.36 |
| MIR302A | Osteoporosis, Postmenopausal | 3.36 |
| MIR378A | Osteoporosis, Postmenopausal | 3.36 |
| MIR885 | Osteoporosis, Postmenopausal | 3.36 |
| MITD1 | Osteoporosis, Postmenopausal | 3.36 |
| MYEOV2 | Osteoporosis, Postmenopausal | 3.36 |
| NAT16 | Osteoporosis, Postmenopausal | 3.36 |
| NEPN | Osteoporosis, Postmenopausal | 3.36 |
| OLMALINC | Osteoporosis, Postmenopausal | 3.36 |
| PGAM4 | Osteoporosis, Postmenopausal | 3.36 |
| PLCH1 | Osteoporosis, Postmenopausal | 3.36 |
| PLCZ1 | Osteoporosis, Postmenopausal | 3.36 |
| PPP1R14D | Osteoporosis, Postmenopausal | 3.36 |
| PRR4 | Osteoporosis, Postmenopausal | 3.36 |
| RAB9 | Osteoporosis, Postmenopausal | 3.36 |
| RDH10B | Osteoporosis, Postmenopausal | 3.36 |
| RGS11 | Osteoporosis, Postmenopausal | 3.36 |
| RLBP1B | Osteoporosis, Postmenopausal | 3.36 |
| RT1-T24-1 | Osteoporosis, Postmenopausal | 3.36 |
| SCART1 | Osteoporosis, Postmenopausal | 3.36 |
| SHCBP1L | Osteoporosis, Postmenopausal | 3.36 |
| SLC26A3.2 | Osteoporosis, Postmenopausal | 3.36 |
| SLC35D3 | Osteoporosis, Postmenopausal | 3.36 |
| SMIM10L2B | Osteoporosis, Postmenopausal | 3.36 |
| SPMIP7 | Osteoporosis, Postmenopausal | 3.36 |
| ST1A8 | Osteoporosis, Postmenopausal | 3.36 |
| TBC1D22B | Osteoporosis, Postmenopausal | 3.36 |
| TEX19 | Osteoporosis, Postmenopausal | 3.36 |
| TMEM14B | Osteoporosis, Postmenopausal | 3.36 |
| TRBC2 | Osteoporosis, Postmenopausal | 3.36 |
| TRMT112 | Osteoporosis, Postmenopausal | 3.36 |
| TTYH1 | Osteoporosis, Postmenopausal | 3.36 |
| TUBA5 | Osteoporosis, Postmenopausal | 3.36 |
| UCMA | Osteoporosis, Postmenopausal | 3.36 |
| WFDC8 | Osteoporosis, Postmenopausal | 3.36 |
| ZBTB33 | Osteoporosis, Postmenopausal | 3.36 |
| ZFP236 | Osteoporosis, Postmenopausal | 3.36 |
| ZFP420 | Osteoporosis, Postmenopausal | 3.36 |
| ZNF230 | Osteoporosis, Postmenopausal | 3.36 |
| ZNF25 | Osteoporosis, Postmenopausal | 3.36 |
| ZNF26 | Osteoporosis, Postmenopausal | 3.36 |
| ZNF431 | Osteoporosis, Postmenopausal | 3.36 |
| ZNF442 | Osteoporosis, Postmenopausal | 3.36 |
| ZNF543 | Osteoporosis, Postmenopausal | 3.36 |
| ZNF549 | Osteoporosis, Postmenopausal | 3.36 |
| ZNF559 | Osteoporosis, Postmenopausal | 3.36 |
| ZNF610 | Osteoporosis, Postmenopausal | 3.36 |
| ZNF696 | Osteoporosis, Postmenopausal | 3.36 |
| ZNF761 | Osteoporosis, Postmenopausal | 3.36 |
| ZNF837 | Osteoporosis, Postmenopausal | 3.36 |
| ZNG1E | Osteoporosis, Postmenopausal | 3.36 |
| AKR1E2 | Osteoporosis, Postmenopausal | 3.35 |
| BTBD17 | Osteoporosis, Postmenopausal | 3.35 |
| CEACAM10 | Osteoporosis, Postmenopausal | 3.35 |
| CEP44 | Osteoporosis, Postmenopausal | 3.35 |
| CLBA1 | Osteoporosis, Postmenopausal | 3.35 |
| CMTR2 | Osteoporosis, Postmenopausal | 3.35 |
| CREB3 | Osteoporosis, Postmenopausal | 3.35 |
| DRP2 | Osteoporosis, Postmenopausal | 3.35 |
| MORN1 | Osteoporosis, Postmenopausal | 3.35 |
| MTBP | Osteoporosis, Postmenopausal | 3.35 |
| MTURN | Osteoporosis, Postmenopausal | 3.35 |
| MYO19 | Osteoporosis, Postmenopausal | 3.35 |
| RINT1 | Osteoporosis, Postmenopausal | 3.35 |
| RUSF1 | Osteoporosis, Postmenopausal | 3.35 |
| SLC6A7 | Osteoporosis, Postmenopausal | 3.35 |
| SPINK2 | Osteoporosis, Postmenopausal | 3.35 |
| TEFM | Osteoporosis, Postmenopausal | 3.35 |
| TMEM128 | Osteoporosis, Postmenopausal | 3.35 |
| TXNDC15 | Osteoporosis, Postmenopausal | 3.35 |
| ZNF117 | Osteoporosis, Postmenopausal | 3.35 |
| A2ML1 | Osteoporosis, Postmenopausal | 3.34 |
| ARMH3 | Osteoporosis, Postmenopausal | 3.34 |
| C2ORF69 | Osteoporosis, Postmenopausal | 3.34 |
| C4BP | Osteoporosis, Postmenopausal | 3.34 |
| CES3B | Osteoporosis, Postmenopausal | 3.34 |
| CIBAR2 | Osteoporosis, Postmenopausal | 3.34 |
| CYP4A32 | Osteoporosis, Postmenopausal | 3.34 |
| DDRGK1 | Osteoporosis, Postmenopausal | 3.34 |
| DPF1 | Osteoporosis, Postmenopausal | 3.34 |
| ECRG4 | Osteoporosis, Postmenopausal | 3.34 |
| FOXO3A | Osteoporosis, Postmenopausal | 3.34 |
| GUCA1B | Osteoporosis, Postmenopausal | 3.34 |
| HHATL | Osteoporosis, Postmenopausal | 3.34 |
| HOXA7 | Osteoporosis, Postmenopausal | 3.34 |
| IL17B | Osteoporosis, Postmenopausal | 3.34 |
| KCTD2 | Osteoporosis, Postmenopausal | 3.34 |
| MINPP1 | Osteoporosis, Postmenopausal | 3.34 |
| NEUROD4 | Osteoporosis, Postmenopausal | 3.34 |
| OPN4 | Osteoporosis, Postmenopausal | 3.34 |
| PAGE4 | Osteoporosis, Postmenopausal | 3.34 |
| PPP1R37 | Osteoporosis, Postmenopausal | 3.34 |
| PRLHR | Osteoporosis, Postmenopausal | 3.34 |
| PRORP | Osteoporosis, Postmenopausal | 3.34 |
| RT1-DMB | Osteoporosis, Postmenopausal | 3.34 |
| SAYSD1 | Osteoporosis, Postmenopausal | 3.34 |
| SLC10A7 | Osteoporosis, Postmenopausal | 3.34 |
| SLC22A11 | Osteoporosis, Postmenopausal | 3.34 |
| TBC1D21 | Osteoporosis, Postmenopausal | 3.34 |
| TEX101 | Osteoporosis, Postmenopausal | 3.34 |
| TRAC | Osteoporosis, Postmenopausal | 3.34 |
| TSPYL5 | Osteoporosis, Postmenopausal | 3.34 |
| USP35 | Osteoporosis, Postmenopausal | 3.34 |
| USP40 | Osteoporosis, Postmenopausal | 3.34 |
| ZNF14 | Osteoporosis, Postmenopausal | 3.34 |
| ZNF615 | Osteoporosis, Postmenopausal | 3.34 |
| ZNF623 | Osteoporosis, Postmenopausal | 3.34 |
| ZNF624 | Osteoporosis, Postmenopausal | 3.34 |
| ADAM30 | Osteoporosis, Postmenopausal | 3.33 |
| AKR7A5 | Osteoporosis, Postmenopausal | 3.33 |
| AMTN | Osteoporosis, Postmenopausal | 3.33 |
| APOA1B | Osteoporosis, Postmenopausal | 3.33 |
| ASB16 | Osteoporosis, Postmenopausal | 3.33 |
| CALR3B | Osteoporosis, Postmenopausal | 3.33 |
| CD300LE | Osteoporosis, Postmenopausal | 3.33 |
| CESL1 | Osteoporosis, Postmenopausal | 3.33 |
| CLEC4M | Osteoporosis, Postmenopausal | 3.33 |
| CMAHP | Osteoporosis, Postmenopausal | 3.33 |
| CNPY1 | Osteoporosis, Postmenopausal | 3.33 |
| CROCC | Osteoporosis, Postmenopausal | 3.33 |
| CST5 | Osteoporosis, Postmenopausal | 3.33 |
| CTSG | Osteoporosis, Postmenopausal | 3.33 |
| CYP3C1 | Osteoporosis, Postmenopausal | 3.33 |
| DCP1B | Osteoporosis, Postmenopausal | 3.33 |
| DNAJB8 | Osteoporosis, Postmenopausal | 3.33 |
| DTD2 | Osteoporosis, Postmenopausal | 3.33 |
| EID2B | Osteoporosis, Postmenopausal | 3.33 |
| EIF4E1B | Osteoporosis, Postmenopausal | 3.33 |
| FAM229A | Osteoporosis, Postmenopausal | 3.33 |
| FAM86B1 | Osteoporosis, Postmenopausal | 3.33 |
| FNDC9 | Osteoporosis, Postmenopausal | 3.33 |
| GPR39 | Osteoporosis, Postmenopausal | 3.33 |
| GSTA.1 | Osteoporosis, Postmenopausal | 3.33 |
| KLLN | Osteoporosis, Postmenopausal | 3.33 |
| LINC02210 | Osteoporosis, Postmenopausal | 3.33 |
| LRRC74A | Osteoporosis, Postmenopausal | 3.33 |
| MALSU1 | Osteoporosis, Postmenopausal | 3.33 |
| MBIP | Osteoporosis, Postmenopausal | 3.33 |
| MIR1275 | Osteoporosis, Postmenopausal | 3.33 |
| MIR654 | Osteoporosis, Postmenopausal | 3.33 |
| MMP20 | Osteoporosis, Postmenopausal | 3.33 |
| MPHOSPH6 | Osteoporosis, Postmenopausal | 3.33 |
| MS4A2 | Osteoporosis, Postmenopausal | 3.33 |
| MYL5 | Osteoporosis, Postmenopausal | 3.33 |
| NAIP1 | Osteoporosis, Postmenopausal | 3.33 |
| NKX2-8 | Osteoporosis, Postmenopausal | 3.33 |
| NRBP1 | Osteoporosis, Postmenopausal | 3.33 |
| PAPOLB | Osteoporosis, Postmenopausal | 3.33 |
| PFKMA | Osteoporosis, Postmenopausal | 3.33 |
| PIRB | Osteoporosis, Postmenopausal | 3.33 |
| PNMA1 | Osteoporosis, Postmenopausal | 3.33 |
| PPAP2B | Osteoporosis, Postmenopausal | 3.33 |
| RGSL1 | Osteoporosis, Postmenopausal | 3.33 |
| RPE65A | Osteoporosis, Postmenopausal | 3.33 |
| RTKN2 | Osteoporosis, Postmenopausal | 3.33 |
| RTTN | Osteoporosis, Postmenopausal | 3.33 |
| SEPW1 | Osteoporosis, Postmenopausal | 3.33 |
| SERTAD4-AS1 | Osteoporosis, Postmenopausal | 3.33 |
| SLC13A1 | Osteoporosis, Postmenopausal | 3.33 |
| SLC2A15B | Osteoporosis, Postmenopausal | 3.33 |
| SLC7A14 | Osteoporosis, Postmenopausal | 3.33 |
| SOX10 | Osteoporosis, Postmenopausal | 3.33 |
| SPECC1 | Osteoporosis, Postmenopausal | 3.33 |
| SYBL1 | Osteoporosis, Postmenopausal | 3.33 |
| TBPL2 | Osteoporosis, Postmenopausal | 3.33 |
| TBR1 | Osteoporosis, Postmenopausal | 3.33 |
| TMEM81 | Osteoporosis, Postmenopausal | 3.33 |
| TMPO-AS1 | Osteoporosis, Postmenopausal | 3.33 |
| TNN | Osteoporosis, Postmenopausal | 3.33 |
| TPD52L3 | Osteoporosis, Postmenopausal | 3.33 |
| ZNF778 | Osteoporosis, Postmenopausal | 3.33 |
| ARHGAP10 | Osteoporosis, Postmenopausal | 3.32 |
| ARL13B | Osteoporosis, Postmenopausal | 3.32 |
| ASB15 | Osteoporosis, Postmenopausal | 3.32 |
| B3GNT4 | Osteoporosis, Postmenopausal | 3.32 |
| CA13 | Osteoporosis, Postmenopausal | 3.32 |
| CMKLR2 | Osteoporosis, Postmenopausal | 3.32 |
| CYP2B13 | Osteoporosis, Postmenopausal | 3.32 |
| CYP2C50 | Osteoporosis, Postmenopausal | 3.32 |
| CYP4F14 | Osteoporosis, Postmenopausal | 3.32 |
| ENTREP1 | Osteoporosis, Postmenopausal | 3.32 |
| ESPNL | Osteoporosis, Postmenopausal | 3.32 |
| FFAR3 | Osteoporosis, Postmenopausal | 3.32 |
| FLVCR1 | Osteoporosis, Postmenopausal | 3.32 |
| GJD2 | Osteoporosis, Postmenopausal | 3.32 |
| KDELR1 | Osteoporosis, Postmenopausal | 3.32 |
| LDAF1 | Osteoporosis, Postmenopausal | 3.32 |
| MAP1LC3C | Osteoporosis, Postmenopausal | 3.32 |
| METTL13 | Osteoporosis, Postmenopausal | 3.32 |
| MIR187 | Osteoporosis, Postmenopausal | 3.32 |
| MIR211 | Osteoporosis, Postmenopausal | 3.32 |
| MIR296 | Osteoporosis, Postmenopausal | 3.32 |
| MIR363 | Osteoporosis, Postmenopausal | 3.32 |
| MIR4435-2HG | Osteoporosis, Postmenopausal | 3.32 |
| MSL3L2 | Osteoporosis, Postmenopausal | 3.32 |
| NEXMIF | Osteoporosis, Postmenopausal | 3.32 |
| NUDT10 | Osteoporosis, Postmenopausal | 3.32 |
| PRORSD1 | Osteoporosis, Postmenopausal | 3.32 |
| SKIDA1 | Osteoporosis, Postmenopausal | 3.32 |
| SOHLH1 | Osteoporosis, Postmenopausal | 3.32 |
| SSC4D | Osteoporosis, Postmenopausal | 3.32 |
| STRA8 | Osteoporosis, Postmenopausal | 3.32 |
| SYT15 | Osteoporosis, Postmenopausal | 3.32 |
| TRAPPC11 | Osteoporosis, Postmenopausal | 3.32 |
| TRAPPC2 | Osteoporosis, Postmenopausal | 3.32 |
| UFSP2 | Osteoporosis, Postmenopausal | 3.32 |
| VPREB1 | Osteoporosis, Postmenopausal | 3.32 |
| ZFP41 | Osteoporosis, Postmenopausal | 3.32 |
| ZNF436-AS1 | Osteoporosis, Postmenopausal | 3.32 |
| ZNF550 | Osteoporosis, Postmenopausal | 3.32 |
| ZNF780B | Osteoporosis, Postmenopausal | 3.32 |
| ZNF85 | Osteoporosis, Postmenopausal | 3.32 |
| ZSCAN21 | Osteoporosis, Postmenopausal | 3.32 |
| CES4A | Osteoporosis, Postmenopausal | 3.31 |
| DNLZ | Osteoporosis, Postmenopausal | 3.31 |
| EPHA6 | Osteoporosis, Postmenopausal | 3.31 |
| ESS2 | Osteoporosis, Postmenopausal | 3.31 |
| FBXO25 | Osteoporosis, Postmenopausal | 3.31 |
| FBXO42 | Osteoporosis, Postmenopausal | 3.31 |
| HOXC9 | Osteoporosis, Postmenopausal | 3.31 |
| IFI203 | Osteoporosis, Postmenopausal | 3.31 |
| PPP1R3D | Osteoporosis, Postmenopausal | 3.31 |
| RASSF3 | Osteoporosis, Postmenopausal | 3.31 |
| SASH3 | Osteoporosis, Postmenopausal | 3.31 |
| SGCA | Osteoporosis, Postmenopausal | 3.31 |
| SH3TC1 | Osteoporosis, Postmenopausal | 3.31 |
| SLC30A5 | Osteoporosis, Postmenopausal | 3.31 |
| SNX25 | Osteoporosis, Postmenopausal | 3.31 |
| SORCS3 | Osteoporosis, Postmenopausal | 3.31 |
| TUBGCP6 | Osteoporosis, Postmenopausal | 3.31 |
| ACTR3C | Osteoporosis, Postmenopausal | 3.3 |
| ADIG | Osteoporosis, Postmenopausal | 3.3 |
| AFG3L1 | Osteoporosis, Postmenopausal | 3.3 |
| AKR1A1A | Osteoporosis, Postmenopausal | 3.3 |
| AND2 | Osteoporosis, Postmenopausal | 3.3 |
| ANKRD66 | Osteoporosis, Postmenopausal | 3.3 |
| APOA1A | Osteoporosis, Postmenopausal | 3.3 |
| AQP6 | Osteoporosis, Postmenopausal | 3.3 |
| AVD | Osteoporosis, Postmenopausal | 3.3 |
| AVPR1B | Osteoporosis, Postmenopausal | 3.3 |
| BCYRN1 | Osteoporosis, Postmenopausal | 3.3 |
| C15ORF61 | Osteoporosis, Postmenopausal | 3.3 |
| C18ORF32 | Osteoporosis, Postmenopausal | 3.3 |
| CALM4 | Osteoporosis, Postmenopausal | 3.3 |
| CAMKMT | Osteoporosis, Postmenopausal | 3.3 |
| CAR13 | Osteoporosis, Postmenopausal | 3.3 |
| CBLL1 | Osteoporosis, Postmenopausal | 3.3 |
| CBY2 | Osteoporosis, Postmenopausal | 3.3 |
| CES2B | Osteoporosis, Postmenopausal | 3.3 |
| CLDN12 | Osteoporosis, Postmenopausal | 3.3 |
| CNBD2 | Osteoporosis, Postmenopausal | 3.3 |
| CYP1C2 | Osteoporosis, Postmenopausal | 3.3 |
| DCD | Osteoporosis, Postmenopausal | 3.3 |
| DPY19L1 | Osteoporosis, Postmenopausal | 3.3 |
| ETV2 | Osteoporosis, Postmenopausal | 3.3 |
| FAM49A | Osteoporosis, Postmenopausal | 3.3 |
| FAM69A | Osteoporosis, Postmenopausal | 3.3 |
| FBXW10B | Osteoporosis, Postmenopausal | 3.3 |
| FOLH1B | Osteoporosis, Postmenopausal | 3.3 |
| G6PD2 | Osteoporosis, Postmenopausal | 3.3 |
| HMX3 | Osteoporosis, Postmenopausal | 3.3 |
| HSD17B12A | Osteoporosis, Postmenopausal | 3.3 |
| HSF5 | Osteoporosis, Postmenopausal | 3.3 |
| IGFL2 | Osteoporosis, Postmenopausal | 3.3 |
| INCA1 | Osteoporosis, Postmenopausal | 3.3 |
| KCNA4 | Osteoporosis, Postmenopausal | 3.3 |
| KCNV2 | Osteoporosis, Postmenopausal | 3.3 |
| LHFPL1 | Osteoporosis, Postmenopausal | 3.3 |
| LINC00673 | Osteoporosis, Postmenopausal | 3.3 |
| LYRM1 | Osteoporosis, Postmenopausal | 3.3 |
| MARCHF9 | Osteoporosis, Postmenopausal | 3.3 |
| MBL1 | Osteoporosis, Postmenopausal | 3.3 |
| MIR346 | Osteoporosis, Postmenopausal | 3.3 |
| MIRLET7B | Osteoporosis, Postmenopausal | 3.3 |
| MUC3A | Osteoporosis, Postmenopausal | 3.3 |
| NANOS2 | Osteoporosis, Postmenopausal | 3.3 |
| NCKAP5 | Osteoporosis, Postmenopausal | 3.3 |
| NOTCH1A | Osteoporosis, Postmenopausal | 3.3 |
| OAS1I | Osteoporosis, Postmenopausal | 3.3 |
| OPHN1 | Osteoporosis, Postmenopausal | 3.3 |
| PCGF6 | Osteoporosis, Postmenopausal | 3.3 |
| PPIAL4A | Osteoporosis, Postmenopausal | 3.3 |
| PPP6R2 | Osteoporosis, Postmenopausal | 3.3 |
| PRP2L1 | Osteoporosis, Postmenopausal | 3.3 |
| PRPH2 | Osteoporosis, Postmenopausal | 3.3 |
| PTTG3P | Osteoporosis, Postmenopausal | 3.3 |
| QRICH1 | Osteoporosis, Postmenopausal | 3.3 |
| RASGEF1C | Osteoporosis, Postmenopausal | 3.3 |
| RT1-CE10 | Osteoporosis, Postmenopausal | 3.3 |
| SERPINA9 | Osteoporosis, Postmenopausal | 3.3 |
| SLC35A2 | Osteoporosis, Postmenopausal | 3.3 |
| SLC35E2A | Osteoporosis, Postmenopausal | 3.3 |
| SNORA70 | Osteoporosis, Postmenopausal | 3.3 |
| SNORA74A | Osteoporosis, Postmenopausal | 3.3 |
| SOX9B | Osteoporosis, Postmenopausal | 3.3 |
| SPACDR | Osteoporosis, Postmenopausal | 3.3 |
| SPRR2A1 | Osteoporosis, Postmenopausal | 3.3 |
| STEEP1 | Osteoporosis, Postmenopausal | 3.3 |
| STPG2 | Osteoporosis, Postmenopausal | 3.3 |
| TMEM245 | Osteoporosis, Postmenopausal | 3.3 |
| TMIE | Osteoporosis, Postmenopausal | 3.3 |
| TREX2 | Osteoporosis, Postmenopausal | 3.3 |
| VAX1 | Osteoporosis, Postmenopausal | 3.3 |
| WDR38 | Osteoporosis, Postmenopausal | 3.3 |
| ZNF419 | Osteoporosis, Postmenopausal | 3.3 |
| ZNF518A | Osteoporosis, Postmenopausal | 3.3 |
| ZNF579 | Osteoporosis, Postmenopausal | 3.3 |
| ZNF587 | Osteoporosis, Postmenopausal | 3.3 |
| ZNF880 | Osteoporosis, Postmenopausal | 3.3 |
| ABCB8 | Osteoporosis, Postmenopausal | 3.29 |
| ABRAXAS1 | Osteoporosis, Postmenopausal | 3.29 |
| B3GAT3 | Osteoporosis, Postmenopausal | 3.29 |
| CHTF8 | Osteoporosis, Postmenopausal | 3.29 |
| DNAH9 | Osteoporosis, Postmenopausal | 3.29 |
| FAM180A | Osteoporosis, Postmenopausal | 3.29 |
| HS2ST1 | Osteoporosis, Postmenopausal | 3.29 |
| HSD3B6 | Osteoporosis, Postmenopausal | 3.29 |
| KIAA1217 | Osteoporosis, Postmenopausal | 3.29 |
| MTMR12 | Osteoporosis, Postmenopausal | 3.29 |
| MTMR9 | Osteoporosis, Postmenopausal | 3.29 |
| NPAT | Osteoporosis, Postmenopausal | 3.29 |
| PPIH | Osteoporosis, Postmenopausal | 3.29 |
| RNASEL | Osteoporosis, Postmenopausal | 3.29 |
| TADA3 | Osteoporosis, Postmenopausal | 3.29 |
| TBX15 | Osteoporosis, Postmenopausal | 3.29 |
| TMEM192 | Osteoporosis, Postmenopausal | 3.29 |
| TMEM68 | Osteoporosis, Postmenopausal | 3.29 |
| XPO7 | Osteoporosis, Postmenopausal | 3.29 |
| ABITRAM | Osteoporosis, Postmenopausal | 3.28 |
| ACR | Osteoporosis, Postmenopausal | 3.28 |
| ADM5 | Osteoporosis, Postmenopausal | 3.28 |
| AFP4 | Osteoporosis, Postmenopausal | 3.28 |
| ANKDD1B | Osteoporosis, Postmenopausal | 3.28 |
| ARHGEF15 | Osteoporosis, Postmenopausal | 3.28 |
| ATAD3B | Osteoporosis, Postmenopausal | 3.28 |
| C1ORF210 | Osteoporosis, Postmenopausal | 3.28 |
| C1QL4 | Osteoporosis, Postmenopausal | 3.28 |
| CCDC154 | Osteoporosis, Postmenopausal | 3.28 |
| CCDC17 | Osteoporosis, Postmenopausal | 3.28 |
| CDYL | Osteoporosis, Postmenopausal | 3.28 |
| CIMAP1C | Osteoporosis, Postmenopausal | 3.28 |
| DPPA5 | Osteoporosis, Postmenopausal | 3.28 |
| FAM134B | Osteoporosis, Postmenopausal | 3.28 |
| FAM187B | Osteoporosis, Postmenopausal | 3.28 |
| FAM214A | Osteoporosis, Postmenopausal | 3.28 |
| FBP1B | Osteoporosis, Postmenopausal | 3.28 |
| FIGLA | Osteoporosis, Postmenopausal | 3.28 |
| GBP8 | Osteoporosis, Postmenopausal | 3.28 |
| GGCTB | Osteoporosis, Postmenopausal | 3.28 |
| GNGT2B | Osteoporosis, Postmenopausal | 3.28 |
| GOLGA8B | Osteoporosis, Postmenopausal | 3.28 |
| GPALPP1 | Osteoporosis, Postmenopausal | 3.28 |
| H4C6 | Osteoporosis, Postmenopausal | 3.28 |
| HIST3H2BA | Osteoporosis, Postmenopausal | 3.28 |
| HS3ST5 | Osteoporosis, Postmenopausal | 3.28 |
| IGSF21 | Osteoporosis, Postmenopausal | 3.28 |
| KBTBD4 | Osteoporosis, Postmenopausal | 3.28 |
| KBTBD7 | Osteoporosis, Postmenopausal | 3.28 |
| KLRA17 | Osteoporosis, Postmenopausal | 3.28 |
| KRT85 | Osteoporosis, Postmenopausal | 3.28 |
| KRTAP1-5 | Osteoporosis, Postmenopausal | 3.28 |
| LCE1C | Osteoporosis, Postmenopausal | 3.28 |
| LCE1D | Osteoporosis, Postmenopausal | 3.28 |
| LILRB4B | Osteoporosis, Postmenopausal | 3.28 |
| LRRC18 | Osteoporosis, Postmenopausal | 3.28 |
| LY6AL | Osteoporosis, Postmenopausal | 3.28 |
| MCPT2 | Osteoporosis, Postmenopausal | 3.28 |
| MIR188 | Osteoporosis, Postmenopausal | 3.28 |
| MIR28 | Osteoporosis, Postmenopausal | 3.28 |
| MIR7-3HG | Osteoporosis, Postmenopausal | 3.28 |
| MUP3 | Osteoporosis, Postmenopausal | 3.28 |
| NKAPD1 | Osteoporosis, Postmenopausal | 3.28 |
| ODAPH | Osteoporosis, Postmenopausal | 3.28 |
| PANX3 | Osteoporosis, Postmenopausal | 3.28 |
| PCDHGC4 | Osteoporosis, Postmenopausal | 3.28 |
| PCNX3 | Osteoporosis, Postmenopausal | 3.28 |
| PHOSPHO2 | Osteoporosis, Postmenopausal | 3.28 |
| PONZR3 | Osteoporosis, Postmenopausal | 3.28 |
| PSME3IP1 | Osteoporosis, Postmenopausal | 3.28 |
| RBM45 | Osteoporosis, Postmenopausal | 3.28 |
| RNF32-DT | Osteoporosis, Postmenopausal | 3.28 |
| RP1L1 | Osteoporosis, Postmenopausal | 3.28 |
| RT1-CE4 | Osteoporosis, Postmenopausal | 3.28 |
| RWDD2B | Osteoporosis, Postmenopausal | 3.28 |
| SLC22A7A | Osteoporosis, Postmenopausal | 3.28 |
| SMKR1 | Osteoporosis, Postmenopausal | 3.28 |
| SPATA46 | Osteoporosis, Postmenopausal | 3.28 |
| SPATS1 | Osteoporosis, Postmenopausal | 3.28 |
| TAPT1-AS1 | Osteoporosis, Postmenopausal | 3.28 |
| TASL | Osteoporosis, Postmenopausal | 3.28 |
| TBATA | Osteoporosis, Postmenopausal | 3.28 |
| THOC5 | Osteoporosis, Postmenopausal | 3.28 |
| TIMD2 | Osteoporosis, Postmenopausal | 3.28 |
| TIMM29 | Osteoporosis, Postmenopausal | 3.28 |
| TMED7-TICAM2 | Osteoporosis, Postmenopausal | 3.28 |
| TMEM173 | Osteoporosis, Postmenopausal | 3.28 |
| TMEM9B | Osteoporosis, Postmenopausal | 3.28 |
| TRHR | Osteoporosis, Postmenopausal | 3.28 |
| TUBA1B | Osteoporosis, Postmenopausal | 3.28 |
| TUBGCP5 | Osteoporosis, Postmenopausal | 3.28 |
| TXK | Osteoporosis, Postmenopausal | 3.28 |
| WFDC16 | Osteoporosis, Postmenopausal | 3.28 |
| WFDC3 | Osteoporosis, Postmenopausal | 3.28 |
| ZFP770 | Osteoporosis, Postmenopausal | 3.28 |
| ZNF7 | Osteoporosis, Postmenopausal | 3.28 |
| ACTR1B | Osteoporosis, Postmenopausal | 3.27 |
| APBA3 | Osteoporosis, Postmenopausal | 3.27 |
| BTN1A1 | Osteoporosis, Postmenopausal | 3.27 |
| CD247 | Osteoporosis, Postmenopausal | 3.27 |
| CHMP4A | Osteoporosis, Postmenopausal | 3.27 |
| CHRNA10 | Osteoporosis, Postmenopausal | 3.27 |
| CRYBA2 | Osteoporosis, Postmenopausal | 3.27 |
| CUTA | Osteoporosis, Postmenopausal | 3.27 |
| DLGAP1 | Osteoporosis, Postmenopausal | 3.27 |
| EML6 | Osteoporosis, Postmenopausal | 3.27 |
| FAM50A | Osteoporosis, Postmenopausal | 3.27 |
| FCGR3A | Osteoporosis, Postmenopausal | 3.27 |
| GTPBP8 | Osteoporosis, Postmenopausal | 3.27 |
| H3C1 | Osteoporosis, Postmenopausal | 3.27 |
| HSD3B4 | Osteoporosis, Postmenopausal | 3.27 |
| IL31 | Osteoporosis, Postmenopausal | 3.27 |
| LIN28B | Osteoporosis, Postmenopausal | 3.27 |
| MAPKAPK5 | Osteoporosis, Postmenopausal | 3.27 |
| MFSD11 | Osteoporosis, Postmenopausal | 3.27 |
| MIR137 | Osteoporosis, Postmenopausal | 3.27 |
| MSANTD2 | Osteoporosis, Postmenopausal | 3.27 |
| NIPSNAP3A | Osteoporosis, Postmenopausal | 3.27 |
| NXPH2 | Osteoporosis, Postmenopausal | 3.27 |
| PCNX2 | Osteoporosis, Postmenopausal | 3.27 |
| POLR3C | Osteoporosis, Postmenopausal | 3.27 |
| PPP1R21 | Osteoporosis, Postmenopausal | 3.27 |
| RPH3A | Osteoporosis, Postmenopausal | 3.27 |
| RPRML | Osteoporosis, Postmenopausal | 3.27 |
| RTL8B | Osteoporosis, Postmenopausal | 3.27 |
| SOS2 | Osteoporosis, Postmenopausal | 3.27 |
| TAF3 | Osteoporosis, Postmenopausal | 3.27 |
| TMEM72 | Osteoporosis, Postmenopausal | 3.27 |
| TRIM67 | Osteoporosis, Postmenopausal | 3.27 |
| TRMU | Osteoporosis, Postmenopausal | 3.27 |
| URGCP | Osteoporosis, Postmenopausal | 3.27 |
| USP53 | Osteoporosis, Postmenopausal | 3.27 |
| ZNF235 | Osteoporosis, Postmenopausal | 3.27 |
| ZNF670 | Osteoporosis, Postmenopausal | 3.27 |
| ZNF791 | Osteoporosis, Postmenopausal | 3.27 |
| ANXA2P3 | Osteoporosis, Postmenopausal | 3.26 |
| AP1AR | Osteoporosis, Postmenopausal | 3.26 |
| ATAD3C | Osteoporosis, Postmenopausal | 3.26 |
| ATF7IP2 | Osteoporosis, Postmenopausal | 3.26 |
| BBS12 | Osteoporosis, Postmenopausal | 3.26 |
| BCAP29 | Osteoporosis, Postmenopausal | 3.26 |
| C11ORF71 | Osteoporosis, Postmenopausal | 3.26 |
| C1H11ORF24 | Osteoporosis, Postmenopausal | 3.26 |
| C8ORF48 | Osteoporosis, Postmenopausal | 3.26 |
| CCDC183 | Osteoporosis, Postmenopausal | 3.26 |
| DIABLOA | Osteoporosis, Postmenopausal | 3.26 |
| DNASE1L1 | Osteoporosis, Postmenopausal | 3.26 |
| GPS1 | Osteoporosis, Postmenopausal | 3.26 |
| GSTA | Osteoporosis, Postmenopausal | 3.26 |
| KDM2B | Osteoporosis, Postmenopausal | 3.26 |
| LZTS1 | Osteoporosis, Postmenopausal | 3.26 |
| MBPB | Osteoporosis, Postmenopausal | 3.26 |
| MCM3AP | Osteoporosis, Postmenopausal | 3.26 |
| MIXL1 | Osteoporosis, Postmenopausal | 3.26 |
| MKRN2OS | Osteoporosis, Postmenopausal | 3.26 |
| NMNAT2 | Osteoporosis, Postmenopausal | 3.26 |
| NR2F1-AS1 | Osteoporosis, Postmenopausal | 3.26 |
| NUP37 | Osteoporosis, Postmenopausal | 3.26 |
| PFKMB | Osteoporosis, Postmenopausal | 3.26 |
| PPP1R16A | Osteoporosis, Postmenopausal | 3.26 |
| PTGS2B | Osteoporosis, Postmenopausal | 3.26 |
| RPAP1 | Osteoporosis, Postmenopausal | 3.26 |
| RSAD1 | Osteoporosis, Postmenopausal | 3.26 |
| RT1-N3 | Osteoporosis, Postmenopausal | 3.26 |
| SH2D1B | Osteoporosis, Postmenopausal | 3.26 |
| SHROOM2 | Osteoporosis, Postmenopausal | 3.26 |
| SLC25A43 | Osteoporosis, Postmenopausal | 3.26 |
| SLC39A1 | Osteoporosis, Postmenopausal | 3.26 |
| SPMAP2 | Osteoporosis, Postmenopausal | 3.26 |
| ST8SIA1 | Osteoporosis, Postmenopausal | 3.26 |
| TAFA5 | Osteoporosis, Postmenopausal | 3.26 |
| TEAD3 | Osteoporosis, Postmenopausal | 3.26 |
| TMEM115 | Osteoporosis, Postmenopausal | 3.26 |
| TOR2A | Osteoporosis, Postmenopausal | 3.26 |
| XLR3A | Osteoporosis, Postmenopausal | 3.26 |
| ZC3H10 | Osteoporosis, Postmenopausal | 3.26 |
| ZCCHC6 | Osteoporosis, Postmenopausal | 3.26 |
| ZFP212 | Osteoporosis, Postmenopausal | 3.26 |
| ZFP217 | Osteoporosis, Postmenopausal | 3.26 |
| ZFP281 | Osteoporosis, Postmenopausal | 3.26 |
| ZFP692 | Osteoporosis, Postmenopausal | 3.26 |
| ZFP775 | Osteoporosis, Postmenopausal | 3.26 |
| ZFP92 | Osteoporosis, Postmenopausal | 3.26 |
| ZNF35 | Osteoporosis, Postmenopausal | 3.26 |
| ZNF358 | Osteoporosis, Postmenopausal | 3.26 |
| ZNF773 | Osteoporosis, Postmenopausal | 3.26 |
| ZNF853 | Osteoporosis, Postmenopausal | 3.26 |
| ZNF862 | Osteoporosis, Postmenopausal | 3.26 |
| ADAM32 | Osteoporosis, Postmenopausal | 3.25 |
| ADGB | Osteoporosis, Postmenopausal | 3.25 |
| ADPRM | Osteoporosis, Postmenopausal | 3.25 |
| AK6 | Osteoporosis, Postmenopausal | 3.25 |
| BTBD8 | Osteoporosis, Postmenopausal | 3.25 |
| C1ORF52 | Osteoporosis, Postmenopausal | 3.25 |
| COPS8 | Osteoporosis, Postmenopausal | 3.25 |
| CYP4F4 | Osteoporosis, Postmenopausal | 3.25 |
| DHH | Osteoporosis, Postmenopausal | 3.25 |
| DNAH2 | Osteoporosis, Postmenopausal | 3.25 |
| EPPIN | Osteoporosis, Postmenopausal | 3.25 |
| EXTL2 | Osteoporosis, Postmenopausal | 3.25 |
| FAM98B | Osteoporosis, Postmenopausal | 3.25 |
| FER1L4 | Osteoporosis, Postmenopausal | 3.25 |
| FPGT | Osteoporosis, Postmenopausal | 3.25 |
| FRRS1L | Osteoporosis, Postmenopausal | 3.25 |
| IGFBP1A | Osteoporosis, Postmenopausal | 3.25 |
| KIAA0930 | Osteoporosis, Postmenopausal | 3.25 |
| KIAA1191 | Osteoporosis, Postmenopausal | 3.25 |
| KIAA1614 | Osteoporosis, Postmenopausal | 3.25 |
| LCE1F | Osteoporosis, Postmenopausal | 3.25 |
| LENG1 | Osteoporosis, Postmenopausal | 3.25 |
| LRRC2 | Osteoporosis, Postmenopausal | 3.25 |
| MIR328 | Osteoporosis, Postmenopausal | 3.25 |
| MIR345 | Osteoporosis, Postmenopausal | 3.25 |
| MYO16 | Osteoporosis, Postmenopausal | 3.25 |
| NKAIN4 | Osteoporosis, Postmenopausal | 3.25 |
| NT5C1A | Osteoporosis, Postmenopausal | 3.25 |
| PCYT1B | Osteoporosis, Postmenopausal | 3.25 |
| PHF1 | Osteoporosis, Postmenopausal | 3.25 |
| PRUNE1 | Osteoporosis, Postmenopausal | 3.25 |
| RFXAP | Osteoporosis, Postmenopausal | 3.25 |
| RIIAD1 | Osteoporosis, Postmenopausal | 3.25 |
| RTF2 | Osteoporosis, Postmenopausal | 3.25 |
| SPSB2 | Osteoporosis, Postmenopausal | 3.25 |
| SRBD1 | Osteoporosis, Postmenopausal | 3.25 |
| TENM1 | Osteoporosis, Postmenopausal | 3.25 |
| UNC79 | Osteoporosis, Postmenopausal | 3.25 |
| UTY | Osteoporosis, Postmenopausal | 3.25 |
| XKR8 | Osteoporosis, Postmenopausal | 3.25 |
| ZBTB14 | Osteoporosis, Postmenopausal | 3.25 |
| ZC3HC1 | Osteoporosis, Postmenopausal | 3.25 |
| ZFY | Osteoporosis, Postmenopausal | 3.25 |
| ZNF432 | Osteoporosis, Postmenopausal | 3.25 |
| ZNF628 | Osteoporosis, Postmenopausal | 3.25 |
| AASDH | Osteoporosis, Postmenopausal | 3.24 |
| ACVR2A | Osteoporosis, Postmenopausal | 3.24 |
| ASTN1 | Osteoporosis, Postmenopausal | 3.24 |
| ATG2A | Osteoporosis, Postmenopausal | 3.24 |
| CBFA2T2 | Osteoporosis, Postmenopausal | 3.24 |
| CERS1 | Osteoporosis, Postmenopausal | 3.24 |
| CTNNA3 | Osteoporosis, Postmenopausal | 3.24 |
| CYP4A31 | Osteoporosis, Postmenopausal | 3.24 |
| F2RL2 | Osteoporosis, Postmenopausal | 3.24 |
| FBXO28 | Osteoporosis, Postmenopausal | 3.24 |
| FHAD1 | Osteoporosis, Postmenopausal | 3.24 |
| GGN | Osteoporosis, Postmenopausal | 3.24 |
| GUCA2A | Osteoporosis, Postmenopausal | 3.24 |
| KCNK3 | Osteoporosis, Postmenopausal | 3.24 |
| KDF1 | Osteoporosis, Postmenopausal | 3.24 |
| KDM5D | Osteoporosis, Postmenopausal | 3.24 |
| LINGO3 | Osteoporosis, Postmenopausal | 3.24 |
| LRRC71 | Osteoporosis, Postmenopausal | 3.24 |
| LSM12 | Osteoporosis, Postmenopausal | 3.24 |
| MED7 | Osteoporosis, Postmenopausal | 3.24 |
| MIR374B | Osteoporosis, Postmenopausal | 3.24 |
| MYL7 | Osteoporosis, Postmenopausal | 3.24 |
| MYOZ3 | Osteoporosis, Postmenopausal | 3.24 |
| NCKIPSD | Osteoporosis, Postmenopausal | 3.24 |
| NEUROG3 | Osteoporosis, Postmenopausal | 3.24 |
| NSA2 | Osteoporosis, Postmenopausal | 3.24 |
| NTAQ1 | Osteoporosis, Postmenopausal | 3.24 |
| NYAP2 | Osteoporosis, Postmenopausal | 3.24 |
| NYX | Osteoporosis, Postmenopausal | 3.24 |
| OCSTAMP | Osteoporosis, Postmenopausal | 3.24 |
| OTP | Osteoporosis, Postmenopausal | 3.24 |
| POU5F2 | Osteoporosis, Postmenopausal | 3.24 |
| PPP1R27 | Osteoporosis, Postmenopausal | 3.24 |
| PTCD3 | Osteoporosis, Postmenopausal | 3.24 |
| QPCTL | Osteoporosis, Postmenopausal | 3.24 |
| RAB28 | Osteoporosis, Postmenopausal | 3.24 |
| SCARNA17 | Osteoporosis, Postmenopausal | 3.24 |
| SMN2 | Osteoporosis, Postmenopausal | 3.24 |
| SMUG1 | Osteoporosis, Postmenopausal | 3.24 |
| SNAPC3 | Osteoporosis, Postmenopausal | 3.24 |
| SNORD22 | Osteoporosis, Postmenopausal | 3.24 |
| SPMIP6 | Osteoporosis, Postmenopausal | 3.24 |
| SRGAP2 | Osteoporosis, Postmenopausal | 3.24 |
| TACR3 | Osteoporosis, Postmenopausal | 3.24 |
| TANC1 | Osteoporosis, Postmenopausal | 3.24 |
| TM4SF18 | Osteoporosis, Postmenopausal | 3.24 |
| TPSB2 | Osteoporosis, Postmenopausal | 3.24 |
| TULP3 | Osteoporosis, Postmenopausal | 3.24 |
| UTF1 | Osteoporosis, Postmenopausal | 3.24 |
| WNT8B | Osteoporosis, Postmenopausal | 3.24 |
| ZNF124 | Osteoporosis, Postmenopausal | 3.24 |
| ZNF286A | Osteoporosis, Postmenopausal | 3.24 |
| ZPBP2 | Osteoporosis, Postmenopausal | 3.24 |
| ACKR2 | Osteoporosis, Postmenopausal | 3.23 |
| ACTB1 | Osteoporosis, Postmenopausal | 3.23 |
| AP3B2 | Osteoporosis, Postmenopausal | 3.23 |
| ASPRV1 | Osteoporosis, Postmenopausal | 3.23 |
| BFAR | Osteoporosis, Postmenopausal | 3.23 |
| BSND | Osteoporosis, Postmenopausal | 3.23 |
| C2CD4D | Osteoporosis, Postmenopausal | 3.23 |
| CCDC83 | Osteoporosis, Postmenopausal | 3.23 |
| CHST12 | Osteoporosis, Postmenopausal | 3.23 |
| CMIP | Osteoporosis, Postmenopausal | 3.23 |
| COG5 | Osteoporosis, Postmenopausal | 3.23 |
| CTRL | Osteoporosis, Postmenopausal | 3.23 |
| CTSLA | Osteoporosis, Postmenopausal | 3.23 |
| CUEDC2 | Osteoporosis, Postmenopausal | 3.23 |
| DMP1 | Osteoporosis, Postmenopausal | 3.23 |
| ECD | Osteoporosis, Postmenopausal | 3.23 |
| EPM2A | Osteoporosis, Postmenopausal | 3.23 |
| EXD1 | Osteoporosis, Postmenopausal | 3.23 |
| F13A1B | Osteoporosis, Postmenopausal | 3.23 |
| H2-EB1 | Osteoporosis, Postmenopausal | 3.23 |
| H2-Q5 | Osteoporosis, Postmenopausal | 3.23 |
| HOXC11 | Osteoporosis, Postmenopausal | 3.23 |
| IGSF23 | Osteoporosis, Postmenopausal | 3.23 |
| INO80C | Osteoporosis, Postmenopausal | 3.23 |
| KIF17 | Osteoporosis, Postmenopausal | 3.23 |
| KRT72 | Osteoporosis, Postmenopausal | 3.23 |
| LGR6 | Osteoporosis, Postmenopausal | 3.23 |
| LRRC19 | Osteoporosis, Postmenopausal | 3.23 |
| LRRK1 | Osteoporosis, Postmenopausal | 3.23 |
| MFSD8 | Osteoporosis, Postmenopausal | 3.23 |
| MLIP | Osteoporosis, Postmenopausal | 3.23 |
| MS4A8A | Osteoporosis, Postmenopausal | 3.23 |
| NAA80 | Osteoporosis, Postmenopausal | 3.23 |
| NXPE1 | Osteoporosis, Postmenopausal | 3.23 |
| PABPC1B | Osteoporosis, Postmenopausal | 3.23 |
| PCDHGB2 | Osteoporosis, Postmenopausal | 3.23 |
| PHKG1 | Osteoporosis, Postmenopausal | 3.23 |
| PIK3R4 | Osteoporosis, Postmenopausal | 3.23 |
| PNP4B | Osteoporosis, Postmenopausal | 3.23 |
| POGLUT3 | Osteoporosis, Postmenopausal | 3.23 |
| PTPRT | Osteoporosis, Postmenopausal | 3.23 |
| RANBP10 | Osteoporosis, Postmenopausal | 3.23 |
| RING1 | Osteoporosis, Postmenopausal | 3.23 |
| RNF103 | Osteoporosis, Postmenopausal | 3.23 |
| SLC25A25A | Osteoporosis, Postmenopausal | 3.23 |
| SLC35F1 | Osteoporosis, Postmenopausal | 3.23 |
| SLC4A5 | Osteoporosis, Postmenopausal | 3.23 |
| SPATA22 | Osteoporosis, Postmenopausal | 3.23 |
| STIM2 | Osteoporosis, Postmenopausal | 3.23 |
| SULT1A4 | Osteoporosis, Postmenopausal | 3.23 |
| TEGT | Osteoporosis, Postmenopausal | 3.23 |
| TEX36 | Osteoporosis, Postmenopausal | 3.23 |
| TMEM196 | Osteoporosis, Postmenopausal | 3.23 |
| WASHC2A | Osteoporosis, Postmenopausal | 3.23 |
| ZFP131 | Osteoporosis, Postmenopausal | 3.23 |
| ANAPC10 | Osteoporosis, Postmenopausal | 3.22 |
| APH1B | Osteoporosis, Postmenopausal | 3.22 |
| BEST4 | Osteoporosis, Postmenopausal | 3.22 |
| CKMT1A | Osteoporosis, Postmenopausal | 3.22 |
| CNOT10 | Osteoporosis, Postmenopausal | 3.22 |
| DHX40 | Osteoporosis, Postmenopausal | 3.22 |
| FAM120C | Osteoporosis, Postmenopausal | 3.22 |
| FAM200B | Osteoporosis, Postmenopausal | 3.22 |
| FFAR1 | Osteoporosis, Postmenopausal | 3.22 |
| GPR182 | Osteoporosis, Postmenopausal | 3.22 |
| H3C12 | Osteoporosis, Postmenopausal | 3.22 |
| HOXD13 | Osteoporosis, Postmenopausal | 3.22 |
| IL17RC | Osteoporosis, Postmenopausal | 3.22 |
| KCNH4 | Osteoporosis, Postmenopausal | 3.22 |
| KCTD6 | Osteoporosis, Postmenopausal | 3.22 |
| KIAA0232 | Osteoporosis, Postmenopausal | 3.22 |
| KIF19 | Osteoporosis, Postmenopausal | 3.22 |
| LY6K | Osteoporosis, Postmenopausal | 3.22 |
| MIR708 | Osteoporosis, Postmenopausal | 3.22 |
| MRPL39 | Osteoporosis, Postmenopausal | 3.22 |
| NKIRAS1 | Osteoporosis, Postmenopausal | 3.22 |
| NOX3 | Osteoporosis, Postmenopausal | 3.22 |
| NT5C3 | Osteoporosis, Postmenopausal | 3.22 |
| PHRF1 | Osteoporosis, Postmenopausal | 3.22 |
| PPCDC | Osteoporosis, Postmenopausal | 3.22 |
| PSPN | Osteoporosis, Postmenopausal | 3.22 |
| PTPRM | Osteoporosis, Postmenopausal | 3.22 |
| RABL2A | Osteoporosis, Postmenopausal | 3.22 |
| SCRT2 | Osteoporosis, Postmenopausal | 3.22 |
| SLC1A6 | Osteoporosis, Postmenopausal | 3.22 |
| STK16 | Osteoporosis, Postmenopausal | 3.22 |
| TM2D3 | Osteoporosis, Postmenopausal | 3.22 |
| ULK4 | Osteoporosis, Postmenopausal | 3.22 |
| VPS52 | Osteoporosis, Postmenopausal | 3.22 |
| WDR76 | Osteoporosis, Postmenopausal | 3.22 |
| ZFP82 | Osteoporosis, Postmenopausal | 3.22 |
| ZFP9 | Osteoporosis, Postmenopausal | 3.22 |
| ZNF131 | Osteoporosis, Postmenopausal | 3.22 |
| ZNF287 | Osteoporosis, Postmenopausal | 3.22 |
| ZNF671 | Osteoporosis, Postmenopausal | 3.22 |
| AMY2A3 | Osteoporosis, Postmenopausal | 3.21 |
| APOBEC3 | Osteoporosis, Postmenopausal | 3.21 |
| APOL7B | Osteoporosis, Postmenopausal | 3.21 |
| ASB18 | Osteoporosis, Postmenopausal | 3.21 |
| AZU1 | Osteoporosis, Postmenopausal | 3.21 |
| BACC1 | Osteoporosis, Postmenopausal | 3.21 |
| BOLL | Osteoporosis, Postmenopausal | 3.21 |
| BTBD2 | Osteoporosis, Postmenopausal | 3.21 |
| C2ORF15 | Osteoporosis, Postmenopausal | 3.21 |
| CCDC102B | Osteoporosis, Postmenopausal | 3.21 |
| CCDC116 | Osteoporosis, Postmenopausal | 3.21 |
| CCDC74B | Osteoporosis, Postmenopausal | 3.21 |
| CCDC78 | Osteoporosis, Postmenopausal | 3.21 |
| CFHR4 | Osteoporosis, Postmenopausal | 3.21 |
| COX20 | Osteoporosis, Postmenopausal | 3.21 |
| CRISP3 | Osteoporosis, Postmenopausal | 3.21 |
| CTC1 | Osteoporosis, Postmenopausal | 3.21 |
| CYP2U1 | Osteoporosis, Postmenopausal | 3.21 |
| DLGAP1-AS1 | Osteoporosis, Postmenopausal | 3.21 |
| DOLK | Osteoporosis, Postmenopausal | 3.21 |
| EFCAB11 | Osteoporosis, Postmenopausal | 3.21 |
| ERGIC2 | Osteoporosis, Postmenopausal | 3.21 |
| EXOC3L1 | Osteoporosis, Postmenopausal | 3.21 |
| FAM129A | Osteoporosis, Postmenopausal | 3.21 |
| FARP2 | Osteoporosis, Postmenopausal | 3.21 |
| FFAR2 | Osteoporosis, Postmenopausal | 3.21 |
| FGF16 | Osteoporosis, Postmenopausal | 3.21 |
| GLOD5 | Osteoporosis, Postmenopausal | 3.21 |
| GPRIN2 | Osteoporosis, Postmenopausal | 3.21 |
| H13 | Osteoporosis, Postmenopausal | 3.21 |
| INO80 | Osteoporosis, Postmenopausal | 3.21 |
| JUNBB | Osteoporosis, Postmenopausal | 3.21 |
| KCNG4 | Osteoporosis, Postmenopausal | 3.21 |
| KLF17 | Osteoporosis, Postmenopausal | 3.21 |
| KLHL35 | Osteoporosis, Postmenopausal | 3.21 |
| L3MBTL2 | Osteoporosis, Postmenopausal | 3.21 |
| LAIR1 | Osteoporosis, Postmenopausal | 3.21 |
| LAMTOR1 | Osteoporosis, Postmenopausal | 3.21 |
| LRMDA | Osteoporosis, Postmenopausal | 3.21 |
| LRRN4 | Osteoporosis, Postmenopausal | 3.21 |
| MCTS2 | Osteoporosis, Postmenopausal | 3.21 |
| MED30 | Osteoporosis, Postmenopausal | 3.21 |
| MIR197 | Osteoporosis, Postmenopausal | 3.21 |
| MIR331 | Osteoporosis, Postmenopausal | 3.21 |
| MIR380 | Osteoporosis, Postmenopausal | 3.21 |
| MISP3 | Osteoporosis, Postmenopausal | 3.21 |
| MORC4 | Osteoporosis, Postmenopausal | 3.21 |
| MYSM1 | Osteoporosis, Postmenopausal | 3.21 |
| NLRP1A | Osteoporosis, Postmenopausal | 3.21 |
| NME9 | Osteoporosis, Postmenopausal | 3.21 |
| PITHD1 | Osteoporosis, Postmenopausal | 3.21 |
| PRDM12 | Osteoporosis, Postmenopausal | 3.21 |
| RATNP-3B | Osteoporosis, Postmenopausal | 3.21 |
| RELCH | Osteoporosis, Postmenopausal | 3.21 |
| RNASE2A | Osteoporosis, Postmenopausal | 3.21 |
| RT1-CE16 | Osteoporosis, Postmenopausal | 3.21 |
| RTL3 | Osteoporosis, Postmenopausal | 3.21 |
| RTL9 | Osteoporosis, Postmenopausal | 3.21 |
| SCNN1D | Osteoporosis, Postmenopausal | 3.21 |
| SHPK | Osteoporosis, Postmenopausal | 3.21 |
| SLC24A5 | Osteoporosis, Postmenopausal | 3.21 |
| SLC43A3 | Osteoporosis, Postmenopausal | 3.21 |
| SMIM10L2A | Osteoporosis, Postmenopausal | 3.21 |
| SP9 | Osteoporosis, Postmenopausal | 3.21 |
| STOML3 | Osteoporosis, Postmenopausal | 3.21 |
| T | Osteoporosis, Postmenopausal | 3.21 |
| TBC1D10B | Osteoporosis, Postmenopausal | 3.21 |
| TMEM151A | Osteoporosis, Postmenopausal | 3.21 |
| TRBC1 | Osteoporosis, Postmenopausal | 3.21 |
| TTC5 | Osteoporosis, Postmenopausal | 3.21 |
| UGT1A6A | Osteoporosis, Postmenopausal | 3.21 |
| VGLL4 | Osteoporosis, Postmenopausal | 3.21 |
| VWC2 | Osteoporosis, Postmenopausal | 3.21 |
| ZFP688 | Osteoporosis, Postmenopausal | 3.21 |
| ZG16 | Osteoporosis, Postmenopausal | 3.21 |
| ZNF385C | Osteoporosis, Postmenopausal | 3.21 |
| ZNF486 | Osteoporosis, Postmenopausal | 3.21 |
| ZNF574 | Osteoporosis, Postmenopausal | 3.21 |
| ZNF587B | Osteoporosis, Postmenopausal | 3.21 |
| ZNF770 | Osteoporosis, Postmenopausal | 3.21 |
| ZNF780A | Osteoporosis, Postmenopausal | 3.21 |
| ZNF821 | Osteoporosis, Postmenopausal | 3.21 |
| ZPLD1 | Osteoporosis, Postmenopausal | 3.21 |
| CLNS1A | Osteoporosis, Postmenopausal | 3.2 |
| CNOT9 | Osteoporosis, Postmenopausal | 3.2 |
| FAM174A | Osteoporosis, Postmenopausal | 3.2 |
| GHDC | Osteoporosis, Postmenopausal | 3.2 |
| INKA2 | Osteoporosis, Postmenopausal | 3.2 |
| LETM2 | Osteoporosis, Postmenopausal | 3.2 |
| LRRC4C | Osteoporosis, Postmenopausal | 3.2 |
| MIR132 | Osteoporosis, Postmenopausal | 3.2 |
| NIPAL4 | Osteoporosis, Postmenopausal | 3.2 |
| PCDH15 | Osteoporosis, Postmenopausal | 3.2 |
| STYX | Osteoporosis, Postmenopausal | 3.2 |
| SULT2A6 | Osteoporosis, Postmenopausal | 3.2 |
| TIMM8A1 | Osteoporosis, Postmenopausal | 3.2 |
| TRIM23 | Osteoporosis, Postmenopausal | 3.2 |
| ZC3H18 | Osteoporosis, Postmenopausal | 3.2 |
| ACTL6B | Osteoporosis, Postmenopausal | 3.19 |
| ANXA1A | Osteoporosis, Postmenopausal | 3.19 |
| APELA | Osteoporosis, Postmenopausal | 3.19 |
| ARHGAP44 | Osteoporosis, Postmenopausal | 3.19 |
| ASPSCR1 | Osteoporosis, Postmenopausal | 3.19 |
| BTBD9 | Osteoporosis, Postmenopausal | 3.19 |
| C12ORF76 | Osteoporosis, Postmenopausal | 3.19 |
| C1D | Osteoporosis, Postmenopausal | 3.19 |
| CACNG1 | Osteoporosis, Postmenopausal | 3.19 |
| CASTOR3P | Osteoporosis, Postmenopausal | 3.19 |
| CDK11B | Osteoporosis, Postmenopausal | 3.19 |
| CHAMP1 | Osteoporosis, Postmenopausal | 3.19 |
| CNGA3 | Osteoporosis, Postmenopausal | 3.19 |
| DNHD1 | Osteoporosis, Postmenopausal | 3.19 |
| EIF4EBP2 | Osteoporosis, Postmenopausal | 3.19 |
| FAM46A | Osteoporosis, Postmenopausal | 3.19 |
| FAM86C1P | Osteoporosis, Postmenopausal | 3.19 |
| FBXO47 | Osteoporosis, Postmenopausal | 3.19 |
| FTMT | Osteoporosis, Postmenopausal | 3.19 |
| GAREM2 | Osteoporosis, Postmenopausal | 3.19 |
| GASK1A | Osteoporosis, Postmenopausal | 3.19 |
| GET1 | Osteoporosis, Postmenopausal | 3.19 |
| GJB6 | Osteoporosis, Postmenopausal | 3.19 |
| GLULA | Osteoporosis, Postmenopausal | 3.19 |
| H2BC12L | Osteoporosis, Postmenopausal | 3.19 |
| IER3IP1 | Osteoporosis, Postmenopausal | 3.19 |
| IFITM7 | Osteoporosis, Postmenopausal | 3.19 |
| IFT172 | Osteoporosis, Postmenopausal | 3.19 |
| INTU | Osteoporosis, Postmenopausal | 3.19 |
| KAP | Osteoporosis, Postmenopausal | 3.19 |
| KAT8 | Osteoporosis, Postmenopausal | 3.19 |
| KCNK10 | Osteoporosis, Postmenopausal | 3.19 |
| KLHL20 | Osteoporosis, Postmenopausal | 3.19 |
| KLRB1 | Osteoporosis, Postmenopausal | 3.19 |
| KRT76 | Osteoporosis, Postmenopausal | 3.19 |
| KRT83 | Osteoporosis, Postmenopausal | 3.19 |
| LCA5L | Osteoporosis, Postmenopausal | 3.19 |
| LINC01089 | Osteoporosis, Postmenopausal | 3.19 |
| LMTK3 | Osteoporosis, Postmenopausal | 3.19 |
| LRRC3B | Osteoporosis, Postmenopausal | 3.19 |
| LSMEM1 | Osteoporosis, Postmenopausal | 3.19 |
| MIR195 | Osteoporosis, Postmenopausal | 3.19 |
| MIR452 | Osteoporosis, Postmenopausal | 3.19 |
| MT | Osteoporosis, Postmenopausal | 3.19 |
| NBPF3 | Osteoporosis, Postmenopausal | 3.19 |
| NPM2 | Osteoporosis, Postmenopausal | 3.19 |
| NTSR2 | Osteoporosis, Postmenopausal | 3.19 |
| NUDT14 | Osteoporosis, Postmenopausal | 3.19 |
| NUDT9 | Osteoporosis, Postmenopausal | 3.19 |
| OTOP1 | Osteoporosis, Postmenopausal | 3.19 |
| PANK2 | Osteoporosis, Postmenopausal | 3.19 |
| PHACTR4 | Osteoporosis, Postmenopausal | 3.19 |
| PIRA2 | Osteoporosis, Postmenopausal | 3.19 |
| POGLUT2 | Osteoporosis, Postmenopausal | 3.19 |
| PRELP | Osteoporosis, Postmenopausal | 3.19 |
| PSMG2 | Osteoporosis, Postmenopausal | 3.19 |
| RGS6 | Osteoporosis, Postmenopausal | 3.19 |
| RSBN1L | Osteoporosis, Postmenopausal | 3.19 |
| RSC1A1 | Osteoporosis, Postmenopausal | 3.19 |
| RTL8C | Osteoporosis, Postmenopausal | 3.19 |
| RUP2 | Osteoporosis, Postmenopausal | 3.19 |
| SAMD4B | Osteoporosis, Postmenopausal | 3.19 |
| SHISA6 | Osteoporosis, Postmenopausal | 3.19 |
| SLC43A2B | Osteoporosis, Postmenopausal | 3.19 |
| SPIN2B | Osteoporosis, Postmenopausal | 3.19 |
| STRN4 | Osteoporosis, Postmenopausal | 3.19 |
| SYCE2 | Osteoporosis, Postmenopausal | 3.19 |
| SYT16 | Osteoporosis, Postmenopausal | 3.19 |
| TBCCD1 | Osteoporosis, Postmenopausal | 3.19 |
| TCP11L1 | Osteoporosis, Postmenopausal | 3.19 |
| TMEM101 | Osteoporosis, Postmenopausal | 3.19 |
| TMEM221 | Osteoporosis, Postmenopausal | 3.19 |
| TSPYL3 | Osteoporosis, Postmenopausal | 3.19 |
| YJEFN3 | Osteoporosis, Postmenopausal | 3.19 |
| ZFP40 | Osteoporosis, Postmenopausal | 3.19 |
| ZFP93 | Osteoporosis, Postmenopausal | 3.19 |
| ZNF687 | Osteoporosis, Postmenopausal | 3.19 |
| AHSA2 | Osteoporosis, Postmenopausal | 3.18 |
| ANO10 | Osteoporosis, Postmenopausal | 3.18 |
| BCLAF3 | Osteoporosis, Postmenopausal | 3.18 |
| CFAP263 | Osteoporosis, Postmenopausal | 3.18 |
| CFAP410 | Osteoporosis, Postmenopausal | 3.18 |
| CNTN3 | Osteoporosis, Postmenopausal | 3.18 |
| COL23A1 | Osteoporosis, Postmenopausal | 3.18 |
| CRIPT | Osteoporosis, Postmenopausal | 3.18 |
| CYB5RL | Osteoporosis, Postmenopausal | 3.18 |
| DDX59 | Osteoporosis, Postmenopausal | 3.18 |
| DOC2B | Osteoporosis, Postmenopausal | 3.18 |
| DPF2 | Osteoporosis, Postmenopausal | 3.18 |
| DUSP23 | Osteoporosis, Postmenopausal | 3.18 |
| FAM20B | Osteoporosis, Postmenopausal | 3.18 |
| FGF14 | Osteoporosis, Postmenopausal | 3.18 |
| FRAT2 | Osteoporosis, Postmenopausal | 3.18 |
| GAN | Osteoporosis, Postmenopausal | 3.18 |
| GPATCH1 | Osteoporosis, Postmenopausal | 3.18 |
| GRIN3A | Osteoporosis, Postmenopausal | 3.18 |
| KIF16B | Osteoporosis, Postmenopausal | 3.18 |
| LBX2 | Osteoporosis, Postmenopausal | 3.18 |
| LRIT1 | Osteoporosis, Postmenopausal | 3.18 |
| LY6G | Osteoporosis, Postmenopausal | 3.18 |
| LY6H | Osteoporosis, Postmenopausal | 3.18 |
| MED9 | Osteoporosis, Postmenopausal | 3.18 |
| METTL5 | Osteoporosis, Postmenopausal | 3.18 |
| NEPRO | Osteoporosis, Postmenopausal | 3.18 |
| NT5DC1 | Osteoporosis, Postmenopausal | 3.18 |
| POU4F2 | Osteoporosis, Postmenopausal | 3.18 |
| PRKRIP1 | Osteoporosis, Postmenopausal | 3.18 |
| PSMG4 | Osteoporosis, Postmenopausal | 3.18 |
| RAB19 | Osteoporosis, Postmenopausal | 3.18 |
| RAB44 | Osteoporosis, Postmenopausal | 3.18 |
| SAMD1 | Osteoporosis, Postmenopausal | 3.18 |
| SEPTIN8 | Osteoporosis, Postmenopausal | 3.18 |
| SMARCAD1 | Osteoporosis, Postmenopausal | 3.18 |
| SPTLC1 | Osteoporosis, Postmenopausal | 3.18 |
| ST7L | Osteoporosis, Postmenopausal | 3.18 |
| TCEANC | Osteoporosis, Postmenopausal | 3.18 |
| TMBIM4 | Osteoporosis, Postmenopausal | 3.18 |
| TMC6 | Osteoporosis, Postmenopausal | 3.18 |
| TMEM181 | Osteoporosis, Postmenopausal | 3.18 |
| TMEM186 | Osteoporosis, Postmenopausal | 3.18 |
| TNMD | Osteoporosis, Postmenopausal | 3.18 |
| UTS2R | Osteoporosis, Postmenopausal | 3.18 |
| VPS26C | Osteoporosis, Postmenopausal | 3.18 |
| VPS4B | Osteoporosis, Postmenopausal | 3.18 |
| ZNF263 | Osteoporosis, Postmenopausal | 3.18 |
| ZNF562 | Osteoporosis, Postmenopausal | 3.18 |
| ZNF678 | Osteoporosis, Postmenopausal | 3.18 |
| ADGRB1 | Osteoporosis, Postmenopausal | 3.17 |
| ADH8B | Osteoporosis, Postmenopausal | 3.17 |
| ARHGAP12 | Osteoporosis, Postmenopausal | 3.17 |
| ASTL | Osteoporosis, Postmenopausal | 3.17 |
| AZGP1 | Osteoporosis, Postmenopausal | 3.17 |
| B4GALT4 | Osteoporosis, Postmenopausal | 3.17 |
| BHLHE23 | Osteoporosis, Postmenopausal | 3.17 |
| BPIFC | Osteoporosis, Postmenopausal | 3.17 |
| C1ORF198 | Osteoporosis, Postmenopausal | 3.17 |
| C6ORF62 | Osteoporosis, Postmenopausal | 3.17 |
| CC2D1B | Osteoporosis, Postmenopausal | 3.17 |
| CDC16 | Osteoporosis, Postmenopausal | 3.17 |
| CFAP157 | Osteoporosis, Postmenopausal | 3.17 |
| CHRNB3 | Osteoporosis, Postmenopausal | 3.17 |
| CST1 | Osteoporosis, Postmenopausal | 3.17 |
| CYTH3 | Osteoporosis, Postmenopausal | 3.17 |
| DHX29 | Osteoporosis, Postmenopausal | 3.17 |
| DOCK3 | Osteoporosis, Postmenopausal | 3.17 |
| DOK1 | Osteoporosis, Postmenopausal | 3.17 |
| DUSP26 | Osteoporosis, Postmenopausal | 3.17 |
| FAM174C | Osteoporosis, Postmenopausal | 3.17 |
| FAM178B | Osteoporosis, Postmenopausal | 3.17 |
| FAM193A | Osteoporosis, Postmenopausal | 3.17 |
| FBXO48 | Osteoporosis, Postmenopausal | 3.17 |
| FTH1A | Osteoporosis, Postmenopausal | 3.17 |
| GNL1 | Osteoporosis, Postmenopausal | 3.17 |
| GUCY2F | Osteoporosis, Postmenopausal | 3.17 |
| HGFAC | Osteoporosis, Postmenopausal | 3.17 |
| HSP90AA1.1 | Osteoporosis, Postmenopausal | 3.17 |
| IGG-2A | Osteoporosis, Postmenopausal | 3.17 |
| KIAA1755 | Osteoporosis, Postmenopausal | 3.17 |
| LBFABP | Osteoporosis, Postmenopausal | 3.17 |
| LGALS2B | Osteoporosis, Postmenopausal | 3.17 |
| LINS1 | Osteoporosis, Postmenopausal | 3.17 |
| MAP7 | Osteoporosis, Postmenopausal | 3.17 |
| MEGF8 | Osteoporosis, Postmenopausal | 3.17 |
| MIR181D | Osteoporosis, Postmenopausal | 3.17 |
| MNAT1 | Osteoporosis, Postmenopausal | 3.17 |
| MNX1 | Osteoporosis, Postmenopausal | 3.17 |
| MUC6 | Osteoporosis, Postmenopausal | 3.17 |
| NR1D2A | Osteoporosis, Postmenopausal | 3.17 |
| NT5C | Osteoporosis, Postmenopausal | 3.17 |
| NTN5 | Osteoporosis, Postmenopausal | 3.17 |
| NYAP1 | Osteoporosis, Postmenopausal | 3.17 |
| PALS1 | Osteoporosis, Postmenopausal | 3.17 |
| PIK3R5 | Osteoporosis, Postmenopausal | 3.17 |
| PSORS1C2 | Osteoporosis, Postmenopausal | 3.17 |
| RAB9B | Osteoporosis, Postmenopausal | 3.17 |
| RABL2 | Osteoporosis, Postmenopausal | 3.17 |
| RIMBP2 | Osteoporosis, Postmenopausal | 3.17 |
| RPP38 | Osteoporosis, Postmenopausal | 3.17 |
| SCAMP1 | Osteoporosis, Postmenopausal | 3.17 |
| SLC25A40 | Osteoporosis, Postmenopausal | 3.17 |
| SLC43A1 | Osteoporosis, Postmenopausal | 3.17 |
| SRRD | Osteoporosis, Postmenopausal | 3.17 |
| TCEANC2 | Osteoporosis, Postmenopausal | 3.17 |
| TMEM145 | Osteoporosis, Postmenopausal | 3.17 |
| TMEM88B | Osteoporosis, Postmenopausal | 3.17 |
| TRIM71 | Osteoporosis, Postmenopausal | 3.17 |
| TRMT10B | Osteoporosis, Postmenopausal | 3.17 |
| ZFP142 | Osteoporosis, Postmenopausal | 3.17 |
| ZFP189 | Osteoporosis, Postmenopausal | 3.17 |
| ZFP280C | Osteoporosis, Postmenopausal | 3.17 |
| ZNF300 | Osteoporosis, Postmenopausal | 3.17 |
| ZNF345 | Osteoporosis, Postmenopausal | 3.17 |
| ZNF45 | Osteoporosis, Postmenopausal | 3.17 |
| ADGRA1 | Osteoporosis, Postmenopausal | 3.16 |
| ANAPC4 | Osteoporosis, Postmenopausal | 3.16 |
| ARL14EP | Osteoporosis, Postmenopausal | 3.16 |
| C1QL3 | Osteoporosis, Postmenopausal | 3.16 |
| CD2 | Osteoporosis, Postmenopausal | 3.16 |
| CEP20 | Osteoporosis, Postmenopausal | 3.16 |
| CHRM5 | Osteoporosis, Postmenopausal | 3.16 |
| DNPEP | Osteoporosis, Postmenopausal | 3.16 |
| EMC4 | Osteoporosis, Postmenopausal | 3.16 |
| EXOC8 | Osteoporosis, Postmenopausal | 3.16 |
| GALNT6 | Osteoporosis, Postmenopausal | 3.16 |
| KLHL9 | Osteoporosis, Postmenopausal | 3.16 |
| MIPOL1 | Osteoporosis, Postmenopausal | 3.16 |
| MTIF2 | Osteoporosis, Postmenopausal | 3.16 |
| MYPOP | Osteoporosis, Postmenopausal | 3.16 |
| NXPE2 | Osteoporosis, Postmenopausal | 3.16 |
| PHOX2A | Osteoporosis, Postmenopausal | 3.16 |
| RANBP6 | Osteoporosis, Postmenopausal | 3.16 |
| RUNDC1 | Osteoporosis, Postmenopausal | 3.16 |
| SIVA1 | Osteoporosis, Postmenopausal | 3.16 |
| SMIM15 | Osteoporosis, Postmenopausal | 3.16 |
| TCTN3 | Osteoporosis, Postmenopausal | 3.16 |
| TRAPPC10 | Osteoporosis, Postmenopausal | 3.16 |
| TRAPPC5 | Osteoporosis, Postmenopausal | 3.16 |
| TRIM11 | Osteoporosis, Postmenopausal | 3.16 |
| VPS16 | Osteoporosis, Postmenopausal | 3.16 |
| WDR44 | Osteoporosis, Postmenopausal | 3.16 |
| ZKSCAN4 | Osteoporosis, Postmenopausal | 3.16 |
| ZNF326 | Osteoporosis, Postmenopausal | 3.16 |
| ZNF507 | Osteoporosis, Postmenopausal | 3.16 |
| AIRIM | Osteoporosis, Postmenopausal | 3.15 |
| AXUD1 | Osteoporosis, Postmenopausal | 3.15 |
| C5H1ORF210 | Osteoporosis, Postmenopausal | 3.15 |
| CCDC126 | Osteoporosis, Postmenopausal | 3.15 |
| CLEC18A | Osteoporosis, Postmenopausal | 3.15 |
| COPZ1 | Osteoporosis, Postmenopausal | 3.15 |
| DCP1A | Osteoporosis, Postmenopausal | 3.15 |
| DNAH14 | Osteoporosis, Postmenopausal | 3.15 |
| DPH6 | Osteoporosis, Postmenopausal | 3.15 |
| FAT2 | Osteoporosis, Postmenopausal | 3.15 |
| FEM1C | Osteoporosis, Postmenopausal | 3.15 |
| G6PC1A.1 | Osteoporosis, Postmenopausal | 3.15 |
| H4C5 | Osteoporosis, Postmenopausal | 3.15 |
| HBB-B2 | Osteoporosis, Postmenopausal | 3.15 |
| HIST1H2BQ | Osteoporosis, Postmenopausal | 3.15 |
| IL22RA1 | Osteoporosis, Postmenopausal | 3.15 |
| KDM1A | Osteoporosis, Postmenopausal | 3.15 |
| KRT6C | Osteoporosis, Postmenopausal | 3.15 |
| LIN7B | Osteoporosis, Postmenopausal | 3.15 |
| LRCH1 | Osteoporosis, Postmenopausal | 3.15 |
| METTL21A | Osteoporosis, Postmenopausal | 3.15 |
| MOG | Osteoporosis, Postmenopausal | 3.15 |
| MPX | Osteoporosis, Postmenopausal | 3.15 |
| MRI1 | Osteoporosis, Postmenopausal | 3.15 |
| N6AMT1 | Osteoporosis, Postmenopausal | 3.15 |
| NAALADL1 | Osteoporosis, Postmenopausal | 3.15 |
| NAPB | Osteoporosis, Postmenopausal | 3.15 |
| NFKBIAB | Osteoporosis, Postmenopausal | 3.15 |
| P3H4 | Osteoporosis, Postmenopausal | 3.15 |
| PATJ | Osteoporosis, Postmenopausal | 3.15 |
| PCDHA11 | Osteoporosis, Postmenopausal | 3.15 |
| PCDHB11 | Osteoporosis, Postmenopausal | 3.15 |
| PHAX | Osteoporosis, Postmenopausal | 3.15 |
| PILRB2 | Osteoporosis, Postmenopausal | 3.15 |
| POGZ | Osteoporosis, Postmenopausal | 3.15 |
| PSAPL1 | Osteoporosis, Postmenopausal | 3.15 |
| RHBDL2 | Osteoporosis, Postmenopausal | 3.15 |
| RPS6KB2 | Osteoporosis, Postmenopausal | 3.15 |
| RRP7A | Osteoporosis, Postmenopausal | 3.15 |
| RUFY1 | Osteoporosis, Postmenopausal | 3.15 |
| SGSM2 | Osteoporosis, Postmenopausal | 3.15 |
| SLC25A16 | Osteoporosis, Postmenopausal | 3.15 |
| SLC66A1LP | Osteoporosis, Postmenopausal | 3.15 |
| SLFN1 | Osteoporosis, Postmenopausal | 3.15 |
| SPAST | Osteoporosis, Postmenopausal | 3.15 |
| SPINT2 | Osteoporosis, Postmenopausal | 3.15 |
| STRADA | Osteoporosis, Postmenopausal | 3.15 |
| TBC1D9B | Osteoporosis, Postmenopausal | 3.15 |
| TERF2 | Osteoporosis, Postmenopausal | 3.15 |
| TTBK2 | Osteoporosis, Postmenopausal | 3.15 |
| ZFHX4 | Osteoporosis, Postmenopausal | 3.15 |
| ZNF275 | Osteoporosis, Postmenopausal | 3.15 |
| ZNF480 | Osteoporosis, Postmenopausal | 3.15 |
| ABCA4 | Osteoporosis, Postmenopausal | 3.14 |
| ABCG3 | Osteoporosis, Postmenopausal | 3.14 |
| ABRAXAS2 | Osteoporosis, Postmenopausal | 3.14 |
| ANKRD65 | Osteoporosis, Postmenopausal | 3.14 |
| APOEA | Osteoporosis, Postmenopausal | 3.14 |
| BAG1 | Osteoporosis, Postmenopausal | 3.14 |
| BNC1 | Osteoporosis, Postmenopausal | 3.14 |
| BTNL8 | Osteoporosis, Postmenopausal | 3.14 |
| CAR14 | Osteoporosis, Postmenopausal | 3.14 |
| CARMIL1 | Osteoporosis, Postmenopausal | 3.14 |
| CASC15 | Osteoporosis, Postmenopausal | 3.14 |
| CCDC25 | Osteoporosis, Postmenopausal | 3.14 |
| CD209 | Osteoporosis, Postmenopausal | 3.14 |
| CD300LD | Osteoporosis, Postmenopausal | 3.14 |
| CD79A | Osteoporosis, Postmenopausal | 3.14 |
| CDH12 | Osteoporosis, Postmenopausal | 3.14 |
| CDKL4 | Osteoporosis, Postmenopausal | 3.14 |
| CDSN | Osteoporosis, Postmenopausal | 3.14 |
| CFAP184 | Osteoporosis, Postmenopausal | 3.14 |
| CLDN22 | Osteoporosis, Postmenopausal | 3.14 |
| CYP3A | Osteoporosis, Postmenopausal | 3.14 |
| DCLK3 | Osteoporosis, Postmenopausal | 3.14 |
| DICER1-AS1 | Osteoporosis, Postmenopausal | 3.14 |
| DPEP3 | Osteoporosis, Postmenopausal | 3.14 |
| EFCAB12 | Osteoporosis, Postmenopausal | 3.14 |
| EPD | Osteoporosis, Postmenopausal | 3.14 |
| FAM222B | Osteoporosis, Postmenopausal | 3.14 |
| FGFBP3 | Osteoporosis, Postmenopausal | 3.14 |
| FMN2 | Osteoporosis, Postmenopausal | 3.14 |
| GNRH3 | Osteoporosis, Postmenopausal | 3.14 |
| GPX6 | Osteoporosis, Postmenopausal | 3.14 |
| GSG1 | Osteoporosis, Postmenopausal | 3.14 |
| H1-4 | Osteoporosis, Postmenopausal | 3.14 |
| HSPA7 | Osteoporosis, Postmenopausal | 3.14 |
| KCTD14 | Osteoporosis, Postmenopausal | 3.14 |
| LPAR2 | Osteoporosis, Postmenopausal | 3.14 |
| LYRM4 | Osteoporosis, Postmenopausal | 3.14 |
| MCF2L2 | Osteoporosis, Postmenopausal | 3.14 |
| METTL26 | Osteoporosis, Postmenopausal | 3.14 |
| MFSD2 | Osteoporosis, Postmenopausal | 3.14 |
| MIRLET7G | Osteoporosis, Postmenopausal | 3.14 |
| NCAPH2 | Osteoporosis, Postmenopausal | 3.14 |
| NRDE2 | Osteoporosis, Postmenopausal | 3.14 |
| PABIR2 | Osteoporosis, Postmenopausal | 3.14 |
| PGK2 | Osteoporosis, Postmenopausal | 3.14 |
| PIWIL1 | Osteoporosis, Postmenopausal | 3.14 |
| PLCXD1 | Osteoporosis, Postmenopausal | 3.14 |
| PPIC | Osteoporosis, Postmenopausal | 3.14 |
| PRDM14 | Osteoporosis, Postmenopausal | 3.14 |
| PRR11 | Osteoporosis, Postmenopausal | 3.14 |
| PSG4 | Osteoporosis, Postmenopausal | 3.14 |
| RAD9B | Osteoporosis, Postmenopausal | 3.14 |
| SERPINB1B | Osteoporosis, Postmenopausal | 3.14 |
| SERPINB9B | Osteoporosis, Postmenopausal | 3.14 |
| SHISA9 | Osteoporosis, Postmenopausal | 3.14 |
| SLC10A6 | Osteoporosis, Postmenopausal | 3.14 |
| SNX1 | Osteoporosis, Postmenopausal | 3.14 |
| STX10 | Osteoporosis, Postmenopausal | 3.14 |
| STX6 | Osteoporosis, Postmenopausal | 3.14 |
| SYT4 | Osteoporosis, Postmenopausal | 3.14 |
| TASOR | Osteoporosis, Postmenopausal | 3.14 |
| TSGA10 | Osteoporosis, Postmenopausal | 3.14 |
| TUBGCP4 | Osteoporosis, Postmenopausal | 3.14 |
| VIRMA | Osteoporosis, Postmenopausal | 3.14 |
| ZDHHC19 | Osteoporosis, Postmenopausal | 3.14 |
| ZFP612 | Osteoporosis, Postmenopausal | 3.14 |
| A3GALT2 | Osteoporosis, Postmenopausal | 3.13 |
| ACTR1A | Osteoporosis, Postmenopausal | 3.13 |
| ADAT3 | Osteoporosis, Postmenopausal | 3.13 |
| ANO7 | Osteoporosis, Postmenopausal | 3.13 |
| ATP6AP1 | Osteoporosis, Postmenopausal | 3.13 |
| BEND5 | Osteoporosis, Postmenopausal | 3.13 |
| CCDC85C | Osteoporosis, Postmenopausal | 3.13 |
| CD300LG | Osteoporosis, Postmenopausal | 3.13 |
| CPB1 | Osteoporosis, Postmenopausal | 3.13 |
| DDX19A | Osteoporosis, Postmenopausal | 3.13 |
| DENND10 | Osteoporosis, Postmenopausal | 3.13 |
| EFCC1 | Osteoporosis, Postmenopausal | 3.13 |
| EIF2B2 | Osteoporosis, Postmenopausal | 3.13 |
| EN1 | Osteoporosis, Postmenopausal | 3.13 |
| EXOSC1 | Osteoporosis, Postmenopausal | 3.13 |
| GARIN1B | Osteoporosis, Postmenopausal | 3.13 |
| GEMIN7 | Osteoporosis, Postmenopausal | 3.13 |
| GPN3 | Osteoporosis, Postmenopausal | 3.13 |
| HOXA2 | Osteoporosis, Postmenopausal | 3.13 |
| INTS4 | Osteoporosis, Postmenopausal | 3.13 |
| ISOC2 | Osteoporosis, Postmenopausal | 3.13 |
| KCNK4 | Osteoporosis, Postmenopausal | 3.13 |
| LRFN5 | Osteoporosis, Postmenopausal | 3.13 |
| METAP1D | Osteoporosis, Postmenopausal | 3.13 |
| MIR497 | Osteoporosis, Postmenopausal | 3.13 |
| MNT | Osteoporosis, Postmenopausal | 3.13 |
| MTRF1L | Osteoporosis, Postmenopausal | 3.13 |
| NAPA | Osteoporosis, Postmenopausal | 3.13 |
| NDUFAF7 | Osteoporosis, Postmenopausal | 3.13 |
| OTOG | Osteoporosis, Postmenopausal | 3.13 |
| PKN1 | Osteoporosis, Postmenopausal | 3.13 |
| POLR3D | Osteoporosis, Postmenopausal | 3.13 |
| PRSS36 | Osteoporosis, Postmenopausal | 3.13 |
| RBM4B | Osteoporosis, Postmenopausal | 3.13 |
| RFNG | Osteoporosis, Postmenopausal | 3.13 |
| RPS19BP1 | Osteoporosis, Postmenopausal | 3.13 |
| RRP36 | Osteoporosis, Postmenopausal | 3.13 |
| RSPRY1 | Osteoporosis, Postmenopausal | 3.13 |
| RTRAF | Osteoporosis, Postmenopausal | 3.13 |
| SEBOX | Osteoporosis, Postmenopausal | 3.13 |
| SIRT7 | Osteoporosis, Postmenopausal | 3.13 |
| SLC24A1 | Osteoporosis, Postmenopausal | 3.13 |
| SLC5A10 | Osteoporosis, Postmenopausal | 3.13 |
| SOX14 | Osteoporosis, Postmenopausal | 3.13 |
| TBCK | Osteoporosis, Postmenopausal | 3.13 |
| TMPRSS9 | Osteoporosis, Postmenopausal | 3.13 |
| TTC14 | Osteoporosis, Postmenopausal | 3.13 |
| TTLL3 | Osteoporosis, Postmenopausal | 3.13 |
| ZAN | Osteoporosis, Postmenopausal | 3.13 |
| ZBTB25 | Osteoporosis, Postmenopausal | 3.13 |
| AKAP14 | Osteoporosis, Postmenopausal | 3.12 |
| AMY2A | Osteoporosis, Postmenopausal | 3.12 |
| ANXA8L1 | Osteoporosis, Postmenopausal | 3.12 |
| AP4S1 | Osteoporosis, Postmenopausal | 3.12 |
| APOEB | Osteoporosis, Postmenopausal | 3.12 |
| ATF6B | Osteoporosis, Postmenopausal | 3.12 |
| ATXN7L3 | Osteoporosis, Postmenopausal | 3.12 |
| B3GNT6 | Osteoporosis, Postmenopausal | 3.12 |
| C11ORF24 | Osteoporosis, Postmenopausal | 3.12 |
| CBR4 | Osteoporosis, Postmenopausal | 3.12 |
| CCDC110 | Osteoporosis, Postmenopausal | 3.12 |
| CEND1 | Osteoporosis, Postmenopausal | 3.12 |
| CEP170B | Osteoporosis, Postmenopausal | 3.12 |
| CKMT1B | Osteoporosis, Postmenopausal | 3.12 |
| CLCNKA | Osteoporosis, Postmenopausal | 3.12 |
| CLN5 | Osteoporosis, Postmenopausal | 3.12 |
| DGLUCY | Osteoporosis, Postmenopausal | 3.12 |
| EAR2 | Osteoporosis, Postmenopausal | 3.12 |
| FAM241A | Osteoporosis, Postmenopausal | 3.12 |
| FAM78B | Osteoporosis, Postmenopausal | 3.12 |
| FBH1 | Osteoporosis, Postmenopausal | 3.12 |
| FBXW17 | Osteoporosis, Postmenopausal | 3.12 |
| FIGNL2 | Osteoporosis, Postmenopausal | 3.12 |
| GALNT11 | Osteoporosis, Postmenopausal | 3.12 |
| GBF1 | Osteoporosis, Postmenopausal | 3.12 |
| GLT8D1 | Osteoporosis, Postmenopausal | 3.12 |
| GPATCH2L | Osteoporosis, Postmenopausal | 3.12 |
| HHIPL1 | Osteoporosis, Postmenopausal | 3.12 |
| IFNE | Osteoporosis, Postmenopausal | 3.12 |
| KIAA1328 | Osteoporosis, Postmenopausal | 3.12 |
| KLHL8 | Osteoporosis, Postmenopausal | 3.12 |
| KRTAP2-3 | Osteoporosis, Postmenopausal | 3.12 |
| LDB2 | Osteoporosis, Postmenopausal | 3.12 |
| LDHC | Osteoporosis, Postmenopausal | 3.12 |
| LRRC41 | Osteoporosis, Postmenopausal | 3.12 |
| LRRC55 | Osteoporosis, Postmenopausal | 3.12 |
| MAGI3 | Osteoporosis, Postmenopausal | 3.12 |
| MEIG1 | Osteoporosis, Postmenopausal | 3.12 |
| MFSD4B | Osteoporosis, Postmenopausal | 3.12 |
| MTCL3 | Osteoporosis, Postmenopausal | 3.12 |
| MTCP1 | Osteoporosis, Postmenopausal | 3.12 |
| MTFR1L | Osteoporosis, Postmenopausal | 3.12 |
| MUC13 | Osteoporosis, Postmenopausal | 3.12 |
| NAIP2 | Osteoporosis, Postmenopausal | 3.12 |
| NKX3-2 | Osteoporosis, Postmenopausal | 3.12 |
| OSTF1 | Osteoporosis, Postmenopausal | 3.12 |
| OXLD1 | Osteoporosis, Postmenopausal | 3.12 |
| PEX14 | Osteoporosis, Postmenopausal | 3.12 |
| PHF21B | Osteoporosis, Postmenopausal | 3.12 |
| PPIL4 | Osteoporosis, Postmenopausal | 3.12 |
| RDH12 | Osteoporosis, Postmenopausal | 3.12 |
| REP15 | Osteoporosis, Postmenopausal | 3.12 |
| RER1 | Osteoporosis, Postmenopausal | 3.12 |
| RFT1 | Osteoporosis, Postmenopausal | 3.12 |
| SCN7A | Osteoporosis, Postmenopausal | 3.12 |
| SEMG1 | Osteoporosis, Postmenopausal | 3.12 |
| SLCO1D1 | Osteoporosis, Postmenopausal | 3.12 |
| SOX15 | Osteoporosis, Postmenopausal | 3.12 |
| SPATA18 | Osteoporosis, Postmenopausal | 3.12 |
| SPTBN2 | Osteoporosis, Postmenopausal | 3.12 |
| SVOP | Osteoporosis, Postmenopausal | 3.12 |
| TMEM165 | Osteoporosis, Postmenopausal | 3.12 |
| TMEM65 | Osteoporosis, Postmenopausal | 3.12 |
| TNFRSF6B | Osteoporosis, Postmenopausal | 3.12 |
| TOMM70 | Osteoporosis, Postmenopausal | 3.12 |
| TSX | Osteoporosis, Postmenopausal | 3.12 |
| UBE2A | Osteoporosis, Postmenopausal | 3.12 |
| UBE2M | Osteoporosis, Postmenopausal | 3.12 |
| UBE2Q2 | Osteoporosis, Postmenopausal | 3.12 |
| USP11 | Osteoporosis, Postmenopausal | 3.12 |
| WDR7 | Osteoporosis, Postmenopausal | 3.12 |
| ZMAT5 | Osteoporosis, Postmenopausal | 3.12 |
| ZNF443 | Osteoporosis, Postmenopausal | 3.12 |
| ZNF688 | Osteoporosis, Postmenopausal | 3.12 |
| ZNF70 | Osteoporosis, Postmenopausal | 3.12 |
| ZNF784 | Osteoporosis, Postmenopausal | 3.12 |
| ZW10 | Osteoporosis, Postmenopausal | 3.12 |
| ABCA7 | Osteoporosis, Postmenopausal | 3.11 |
| AKAP7 | Osteoporosis, Postmenopausal | 3.11 |
| ATPAF2 | Osteoporosis, Postmenopausal | 3.11 |
| CACNB1 | Osteoporosis, Postmenopausal | 3.11 |
| CCAR2 | Osteoporosis, Postmenopausal | 3.11 |
| CMA1 | Osteoporosis, Postmenopausal | 3.11 |
| CSGALNACT2 | Osteoporosis, Postmenopausal | 3.11 |
| CUTC | Osteoporosis, Postmenopausal | 3.11 |
| CYREN | Osteoporosis, Postmenopausal | 3.11 |
| FAM76A | Osteoporosis, Postmenopausal | 3.11 |
| GRK1 | Osteoporosis, Postmenopausal | 3.11 |
| H2AC20 | Osteoporosis, Postmenopausal | 3.11 |
| KLHL17 | Osteoporosis, Postmenopausal | 3.11 |
| LIG3 | Osteoporosis, Postmenopausal | 3.11 |
| MAGED1 | Osteoporosis, Postmenopausal | 3.11 |
| MAMSTR | Osteoporosis, Postmenopausal | 3.11 |
| MED10 | Osteoporosis, Postmenopausal | 3.11 |
| MIR186 | Osteoporosis, Postmenopausal | 3.11 |
| MROH7 | Osteoporosis, Postmenopausal | 3.11 |
| MTNR1B | Osteoporosis, Postmenopausal | 3.11 |
| NT5M | Osteoporosis, Postmenopausal | 3.11 |
| OSBPL10 | Osteoporosis, Postmenopausal | 3.11 |
| OSBPL1A | Osteoporosis, Postmenopausal | 3.11 |
| PDAP1 | Osteoporosis, Postmenopausal | 3.11 |
| PLEKHG4 | Osteoporosis, Postmenopausal | 3.11 |
| PLEKHM1 | Osteoporosis, Postmenopausal | 3.11 |
| POU3F3 | Osteoporosis, Postmenopausal | 3.11 |
| RETREG2 | Osteoporosis, Postmenopausal | 3.11 |
| SENP1 | Osteoporosis, Postmenopausal | 3.11 |
| SLC7A3 | Osteoporosis, Postmenopausal | 3.11 |
| SP8 | Osteoporosis, Postmenopausal | 3.11 |
| TENT2 | Osteoporosis, Postmenopausal | 3.11 |
| TIGD5 | Osteoporosis, Postmenopausal | 3.11 |
| TMEM204 | Osteoporosis, Postmenopausal | 3.11 |
| TMEM74B | Osteoporosis, Postmenopausal | 3.11 |
| TRAPPC13 | Osteoporosis, Postmenopausal | 3.11 |
| ZFP148 | Osteoporosis, Postmenopausal | 3.11 |
| ACSBG1 | Osteoporosis, Postmenopausal | 3.1 |
| ADCK3 | Osteoporosis, Postmenopausal | 3.1 |
| AMELX | Osteoporosis, Postmenopausal | 3.1 |
| ANG2 | Osteoporosis, Postmenopausal | 3.1 |
| ANKK1 | Osteoporosis, Postmenopausal | 3.1 |
| ARHGAP21 | Osteoporosis, Postmenopausal | 3.1 |
| ARHGAP36 | Osteoporosis, Postmenopausal | 3.1 |
| ARMC5 | Osteoporosis, Postmenopausal | 3.1 |
| ATL1 | Osteoporosis, Postmenopausal | 3.1 |
| BCL2L14 | Osteoporosis, Postmenopausal | 3.1 |
| CFAP61 | Osteoporosis, Postmenopausal | 3.1 |
| CLEC2G | Osteoporosis, Postmenopausal | 3.1 |
| COL28A1 | Osteoporosis, Postmenopausal | 3.1 |
| CRNKL1 | Osteoporosis, Postmenopausal | 3.1 |
| CYP4F6 | Osteoporosis, Postmenopausal | 3.1 |
| DDX60L | Osteoporosis, Postmenopausal | 3.1 |
| DENND5B | Osteoporosis, Postmenopausal | 3.1 |
| DIS3L | Osteoporosis, Postmenopausal | 3.1 |
| DPH3 | Osteoporosis, Postmenopausal | 3.1 |
| EFCAB10 | Osteoporosis, Postmenopausal | 3.1 |
| EFCAB3 | Osteoporosis, Postmenopausal | 3.1 |
| EMC2 | Osteoporosis, Postmenopausal | 3.1 |
| ETV7 | Osteoporosis, Postmenopausal | 3.1 |
| FCRL5 | Osteoporosis, Postmenopausal | 3.1 |
| GTF3C3 | Osteoporosis, Postmenopausal | 3.1 |
| H2-AA | Osteoporosis, Postmenopausal | 3.1 |
| H3C13 | Osteoporosis, Postmenopausal | 3.1 |
| HLA-DMA | Osteoporosis, Postmenopausal | 3.1 |
| HOXB1 | Osteoporosis, Postmenopausal | 3.1 |
| INTS5 | Osteoporosis, Postmenopausal | 3.1 |
| KIF2A | Osteoporosis, Postmenopausal | 3.1 |
| LMBRD1 | Osteoporosis, Postmenopausal | 3.1 |
| MIR152 | Osteoporosis, Postmenopausal | 3.1 |
| MIR23B | Osteoporosis, Postmenopausal | 3.1 |
| MIR31 | Osteoporosis, Postmenopausal | 3.1 |
| MMP27 | Osteoporosis, Postmenopausal | 3.1 |
| NCKAP5L | Osteoporosis, Postmenopausal | 3.1 |
| NELFCD | Osteoporosis, Postmenopausal | 3.1 |
| NLK | Osteoporosis, Postmenopausal | 3.1 |
| NPY4R | Osteoporosis, Postmenopausal | 3.1 |
| NRSN2 | Osteoporosis, Postmenopausal | 3.1 |
| NUP214 | Osteoporosis, Postmenopausal | 3.1 |
| OMA1 | Osteoporosis, Postmenopausal | 3.1 |
| PCBD2 | Osteoporosis, Postmenopausal | 3.1 |
| PEX5L | Osteoporosis, Postmenopausal | 3.1 |
| PROKR2 | Osteoporosis, Postmenopausal | 3.1 |
| PRR36 | Osteoporosis, Postmenopausal | 3.1 |
| PRSS53 | Osteoporosis, Postmenopausal | 3.1 |
| PTCD2 | Osteoporosis, Postmenopausal | 3.1 |
| PTPN3 | Osteoporosis, Postmenopausal | 3.1 |
| RBM4 | Osteoporosis, Postmenopausal | 3.1 |
| SEPTIN1 | Osteoporosis, Postmenopausal | 3.1 |
| SKIC8 | Osteoporosis, Postmenopausal | 3.1 |
| SLC16A6B | Osteoporosis, Postmenopausal | 3.1 |
| SLC9A8 | Osteoporosis, Postmenopausal | 3.1 |
| SPO11 | Osteoporosis, Postmenopausal | 3.1 |
| STK31 | Osteoporosis, Postmenopausal | 3.1 |
| SYNDIG1L | Osteoporosis, Postmenopausal | 3.1 |
| TACO1 | Osteoporosis, Postmenopausal | 3.1 |
| TADA2B | Osteoporosis, Postmenopausal | 3.1 |
| TAS1R2 | Osteoporosis, Postmenopausal | 3.1 |
| TDRD3 | Osteoporosis, Postmenopausal | 3.1 |
| TRAPPC14 | Osteoporosis, Postmenopausal | 3.1 |
| TRIM17 | Osteoporosis, Postmenopausal | 3.1 |
| UBAP2 | Osteoporosis, Postmenopausal | 3.1 |
| URI1 | Osteoporosis, Postmenopausal | 3.1 |
| VEGFAA | Osteoporosis, Postmenopausal | 3.1 |
| XKR9 | Osteoporosis, Postmenopausal | 3.1 |
| ZBED5 | Osteoporosis, Postmenopausal | 3.1 |
| ZBTB37 | Osteoporosis, Postmenopausal | 3.1 |
| ZFP318 | Osteoporosis, Postmenopausal | 3.1 |
| ZKSCAN8 | Osteoporosis, Postmenopausal | 3.1 |
| ZNF202 | Osteoporosis, Postmenopausal | 3.1 |
| ZNF460 | Osteoporosis, Postmenopausal | 3.1 |
| ZNF567 | Osteoporosis, Postmenopausal | 3.1 |
| ZNF597 | Osteoporosis, Postmenopausal | 3.1 |
| ZSWIM1 | Osteoporosis, Postmenopausal | 3.1 |
| ACOT3 | Osteoporosis, Postmenopausal | 3.09 |
| ANKRD23 | Osteoporosis, Postmenopausal | 3.09 |
| ARHGAP40 | Osteoporosis, Postmenopausal | 3.09 |
| BATF | Osteoporosis, Postmenopausal | 3.09 |
| CAPN13 | Osteoporosis, Postmenopausal | 3.09 |
| CBLN3 | Osteoporosis, Postmenopausal | 3.09 |
| CCDC153 | Osteoporosis, Postmenopausal | 3.09 |
| CDH10 | Osteoporosis, Postmenopausal | 3.09 |
| CDK16 | Osteoporosis, Postmenopausal | 3.09 |
| CEP70 | Osteoporosis, Postmenopausal | 3.09 |
| CFAP58 | Osteoporosis, Postmenopausal | 3.09 |
| CLEC9A | Osteoporosis, Postmenopausal | 3.09 |
| CLPTM1L | Osteoporosis, Postmenopausal | 3.09 |
| COPS4 | Osteoporosis, Postmenopausal | 3.09 |
| CRHB | Osteoporosis, Postmenopausal | 3.09 |
| CTLA2B | Osteoporosis, Postmenopausal | 3.09 |
| CYP4F39 | Osteoporosis, Postmenopausal | 3.09 |
| DNMT3L | Osteoporosis, Postmenopausal | 3.09 |
| DRAP1 | Osteoporosis, Postmenopausal | 3.09 |
| FAM151B | Osteoporosis, Postmenopausal | 3.09 |
| FNDC11 | Osteoporosis, Postmenopausal | 3.09 |
| GATD3A | Osteoporosis, Postmenopausal | 3.09 |
| GBGT1 | Osteoporosis, Postmenopausal | 3.09 |
| GLB1L3 | Osteoporosis, Postmenopausal | 3.09 |
| GOLGA3 | Osteoporosis, Postmenopausal | 3.09 |
| GPR22 | Osteoporosis, Postmenopausal | 3.09 |
| H4C9 | Osteoporosis, Postmenopausal | 3.09 |
| HNRNPA0 | Osteoporosis, Postmenopausal | 3.09 |
| HSC70 | Osteoporosis, Postmenopausal | 3.09 |
| IFT70B | Osteoporosis, Postmenopausal | 3.09 |
| IKBKE | Osteoporosis, Postmenopausal | 3.09 |
| LCTL | Osteoporosis, Postmenopausal | 3.09 |
| MAP3K15 | Osteoporosis, Postmenopausal | 3.09 |
| MT1HL1 | Osteoporosis, Postmenopausal | 3.09 |
| NOX5 | Osteoporosis, Postmenopausal | 3.09 |
| PI4KA | Osteoporosis, Postmenopausal | 3.09 |
| PTPRQ | Osteoporosis, Postmenopausal | 3.09 |
| RIMOC1 | Osteoporosis, Postmenopausal | 3.09 |
| RNF157 | Osteoporosis, Postmenopausal | 3.09 |
| RSPH14 | Osteoporosis, Postmenopausal | 3.09 |
| SCGB2A2 | Osteoporosis, Postmenopausal | 3.09 |
| SHANK1 | Osteoporosis, Postmenopausal | 3.09 |
| SLC35E2B | Osteoporosis, Postmenopausal | 3.09 |
| SLURP1 | Osteoporosis, Postmenopausal | 3.09 |
| SNCB | Osteoporosis, Postmenopausal | 3.09 |
| TPST2 | Osteoporosis, Postmenopausal | 3.09 |
| TRPM3 | Osteoporosis, Postmenopausal | 3.09 |
| XRRA1 | Osteoporosis, Postmenopausal | 3.09 |
| ZNF428 | Osteoporosis, Postmenopausal | 3.09 |
| AKR1C19 | Osteoporosis, Postmenopausal | 3.08 |
| ARL15 | Osteoporosis, Postmenopausal | 3.08 |
| ASB3 | Osteoporosis, Postmenopausal | 3.08 |
| ATG14 | Osteoporosis, Postmenopausal | 3.08 |
| ATG16L2 | Osteoporosis, Postmenopausal | 3.08 |
| ATN1 | Osteoporosis, Postmenopausal | 3.08 |
| BMAL2 | Osteoporosis, Postmenopausal | 3.08 |
| C11ORF96 | Osteoporosis, Postmenopausal | 3.08 |
| CAPSL | Osteoporosis, Postmenopausal | 3.08 |
| CHIL3 | Osteoporosis, Postmenopausal | 3.08 |
| CNKSR2 | Osteoporosis, Postmenopausal | 3.08 |
| COA6 | Osteoporosis, Postmenopausal | 3.08 |
| COMMD7 | Osteoporosis, Postmenopausal | 3.08 |
| DCST1 | Osteoporosis, Postmenopausal | 3.08 |
| DENR | Osteoporosis, Postmenopausal | 3.08 |
| E4F1 | Osteoporosis, Postmenopausal | 3.08 |
| FAM53B | Osteoporosis, Postmenopausal | 3.08 |
| GALNT17 | Osteoporosis, Postmenopausal | 3.08 |
| GATD3 | Osteoporosis, Postmenopausal | 3.08 |
| GFRA4 | Osteoporosis, Postmenopausal | 3.08 |
| INSM2 | Osteoporosis, Postmenopausal | 3.08 |
| LRRC61 | Osteoporosis, Postmenopausal | 3.08 |
| LUZP1 | Osteoporosis, Postmenopausal | 3.08 |
| LYPLAL1 | Osteoporosis, Postmenopausal | 3.08 |
| MEPCE | Osteoporosis, Postmenopausal | 3.08 |
| MIR203 | Osteoporosis, Postmenopausal | 3.08 |
| MOCS2 | Osteoporosis, Postmenopausal | 3.08 |
| NKX2-2 | Osteoporosis, Postmenopausal | 3.08 |
| OLFM3 | Osteoporosis, Postmenopausal | 3.08 |
| PABPC1L | Osteoporosis, Postmenopausal | 3.08 |
| PCGF5 | Osteoporosis, Postmenopausal | 3.08 |
| PHF10 | Osteoporosis, Postmenopausal | 3.08 |
| PRDM13 | Osteoporosis, Postmenopausal | 3.08 |
| RBFOX2 | Osteoporosis, Postmenopausal | 3.08 |
| SAP30BP | Osteoporosis, Postmenopausal | 3.08 |
| SCHIP1 | Osteoporosis, Postmenopausal | 3.08 |
| SCN3A | Osteoporosis, Postmenopausal | 3.08 |
| SHARPIN | Osteoporosis, Postmenopausal | 3.08 |
| SIK2 | Osteoporosis, Postmenopausal | 3.08 |
| SPIN1 | Osteoporosis, Postmenopausal | 3.08 |
| STX1B | Osteoporosis, Postmenopausal | 3.08 |
| SYT2 | Osteoporosis, Postmenopausal | 3.08 |
| TAF11 | Osteoporosis, Postmenopausal | 3.08 |
| TAF7 | Osteoporosis, Postmenopausal | 3.08 |
| TBC1D12 | Osteoporosis, Postmenopausal | 3.08 |
| TRIAP1 | Osteoporosis, Postmenopausal | 3.08 |
| UBXN2B | Osteoporosis, Postmenopausal | 3.08 |
| WDR55 | Osteoporosis, Postmenopausal | 3.08 |
| ZBTB1 | Osteoporosis, Postmenopausal | 3.08 |
| ZNF410 | Osteoporosis, Postmenopausal | 3.08 |
| AARSD1 | Osteoporosis, Postmenopausal | 3.07 |
| ABCA1A | Osteoporosis, Postmenopausal | 3.07 |
| ADGRG5 | Osteoporosis, Postmenopausal | 3.07 |
| AKAP17B | Osteoporosis, Postmenopausal | 3.07 |
| ATP2A2B | Osteoporosis, Postmenopausal | 3.07 |
| ATP6V1G2 | Osteoporosis, Postmenopausal | 3.07 |
| BEST2 | Osteoporosis, Postmenopausal | 3.07 |
| BIN3 | Osteoporosis, Postmenopausal | 3.07 |
| C6ORF132 | Osteoporosis, Postmenopausal | 3.07 |
| CACTIN | Osteoporosis, Postmenopausal | 3.07 |
| CASP5 | Osteoporosis, Postmenopausal | 3.07 |
| CD101 | Osteoporosis, Postmenopausal | 3.07 |
| CFAP20DC | Osteoporosis, Postmenopausal | 3.07 |
| CLCA4 | Osteoporosis, Postmenopausal | 3.07 |
| CPLANE2 | Osteoporosis, Postmenopausal | 3.07 |
| CPNE2 | Osteoporosis, Postmenopausal | 3.07 |
| CTGF | Osteoporosis, Postmenopausal | 3.07 |
| CYP2F1 | Osteoporosis, Postmenopausal | 3.07 |
| DENND1A | Osteoporosis, Postmenopausal | 3.07 |
| DHX32 | Osteoporosis, Postmenopausal | 3.07 |
| ELA3L | Osteoporosis, Postmenopausal | 3.07 |
| EXD2 | Osteoporosis, Postmenopausal | 3.07 |
| FAM13C | Osteoporosis, Postmenopausal | 3.07 |
| FAM25A | Osteoporosis, Postmenopausal | 3.07 |
| FERRY3 | Osteoporosis, Postmenopausal | 3.07 |
| GJC3 | Osteoporosis, Postmenopausal | 3.07 |
| GSDMC | Osteoporosis, Postmenopausal | 3.07 |
| H2BC13 | Osteoporosis, Postmenopausal | 3.07 |
| H2-Q1 | Osteoporosis, Postmenopausal | 3.07 |
| IQCN | Osteoporosis, Postmenopausal | 3.07 |
| ITGA7 | Osteoporosis, Postmenopausal | 3.07 |
| MCM9 | Osteoporosis, Postmenopausal | 3.07 |
| MED28 | Osteoporosis, Postmenopausal | 3.07 |
| METTL25 | Osteoporosis, Postmenopausal | 3.07 |
| MIR126 | Osteoporosis, Postmenopausal | 3.07 |
| MLLT1 | Osteoporosis, Postmenopausal | 3.07 |
| NHLRC2 | Osteoporosis, Postmenopausal | 3.07 |
| NPAS4A | Osteoporosis, Postmenopausal | 3.07 |
| ODAD1 | Osteoporosis, Postmenopausal | 3.07 |
| OFD1 | Osteoporosis, Postmenopausal | 3.07 |
| PCDHB4 | Osteoporosis, Postmenopausal | 3.07 |
| PDE8B | Osteoporosis, Postmenopausal | 3.07 |
| PRPF40B | Osteoporosis, Postmenopausal | 3.07 |
| PTPN5 | Osteoporosis, Postmenopausal | 3.07 |
| RASL2-9 | Osteoporosis, Postmenopausal | 3.07 |
| RPAP2 | Osteoporosis, Postmenopausal | 3.07 |
| RPP30 | Osteoporosis, Postmenopausal | 3.07 |
| SCIMP | Osteoporosis, Postmenopausal | 3.07 |
| SLCO1C1 | Osteoporosis, Postmenopausal | 3.07 |
| SPATA6L | Osteoporosis, Postmenopausal | 3.07 |
| STRIP1 | Osteoporosis, Postmenopausal | 3.07 |
| STYXL1 | Osteoporosis, Postmenopausal | 3.07 |
| SYNPR | Osteoporosis, Postmenopausal | 3.07 |
| TLR10 | Osteoporosis, Postmenopausal | 3.07 |
| TMEM92 | Osteoporosis, Postmenopausal | 3.07 |
| TREM3 | Osteoporosis, Postmenopausal | 3.07 |
| TSR2 | Osteoporosis, Postmenopausal | 3.07 |
| TSTD2 | Osteoporosis, Postmenopausal | 3.07 |
| VPS37C | Osteoporosis, Postmenopausal | 3.07 |
| ZC3H12B | Osteoporosis, Postmenopausal | 3.07 |
| ZFP207 | Osteoporosis, Postmenopausal | 3.07 |
| ZFP37 | Osteoporosis, Postmenopausal | 3.07 |
| ZFP609 | Osteoporosis, Postmenopausal | 3.07 |
| ZNF142 | Osteoporosis, Postmenopausal | 3.07 |
| ZNF143 | Osteoporosis, Postmenopausal | 3.07 |
| ZNF485 | Osteoporosis, Postmenopausal | 3.07 |
| ZNF569 | Osteoporosis, Postmenopausal | 3.07 |
| ZSWIM7 | Osteoporosis, Postmenopausal | 3.07 |
| ALKBH3 | Osteoporosis, Postmenopausal | 3.06 |
| AMPH | Osteoporosis, Postmenopausal | 3.06 |
| ARHGAP17 | Osteoporosis, Postmenopausal | 3.06 |
| ASPDH | Osteoporosis, Postmenopausal | 3.06 |
| BBS9 | Osteoporosis, Postmenopausal | 3.06 |
| CA11 | Osteoporosis, Postmenopausal | 3.06 |
| CCDC162 | Osteoporosis, Postmenopausal | 3.06 |
| CLASP1 | Osteoporosis, Postmenopausal | 3.06 |
| CLP1 | Osteoporosis, Postmenopausal | 3.06 |
| CPA1 | Osteoporosis, Postmenopausal | 3.06 |
| CRYBA1 | Osteoporosis, Postmenopausal | 3.06 |
| CUL2 | Osteoporosis, Postmenopausal | 3.06 |
| CUL4B | Osteoporosis, Postmenopausal | 3.06 |
| CYP2D3 | Osteoporosis, Postmenopausal | 3.06 |
| DCAF12L1 | Osteoporosis, Postmenopausal | 3.06 |
| DDX49 | Osteoporosis, Postmenopausal | 3.06 |
| DMPK | Osteoporosis, Postmenopausal | 3.06 |
| DNAH1 | Osteoporosis, Postmenopausal | 3.06 |
| EXD3 | Osteoporosis, Postmenopausal | 3.06 |
| EXOC2 | Osteoporosis, Postmenopausal | 3.06 |
| FAM20A | Osteoporosis, Postmenopausal | 3.06 |
| FBXL18 | Osteoporosis, Postmenopausal | 3.06 |
| FBXW2 | Osteoporosis, Postmenopausal | 3.06 |
| FNTA | Osteoporosis, Postmenopausal | 3.06 |
| GABRD | Osteoporosis, Postmenopausal | 3.06 |
| GFM1 | Osteoporosis, Postmenopausal | 3.06 |
| GPR12 | Osteoporosis, Postmenopausal | 3.06 |
| H2-T23 | Osteoporosis, Postmenopausal | 3.06 |
| HIST1H4B | Osteoporosis, Postmenopausal | 3.06 |
| HLA-DQB1 | Osteoporosis, Postmenopausal | 3.06 |
| IFI27L2 | Osteoporosis, Postmenopausal | 3.06 |
| IGLL1 | Osteoporosis, Postmenopausal | 3.06 |
| IPO5 | Osteoporosis, Postmenopausal | 3.06 |
| ISOC2B | Osteoporosis, Postmenopausal | 3.06 |
| JSRP1 | Osteoporosis, Postmenopausal | 3.06 |
| KEG1 | Osteoporosis, Postmenopausal | 3.06 |
| L1TD1 | Osteoporosis, Postmenopausal | 3.06 |
| MAN2C1 | Osteoporosis, Postmenopausal | 3.06 |
| METTL23 | Osteoporosis, Postmenopausal | 3.06 |
| MINDY2 | Osteoporosis, Postmenopausal | 3.06 |
| MIR449A | Osteoporosis, Postmenopausal | 3.06 |
| MTMR11 | Osteoporosis, Postmenopausal | 3.06 |
| MUP4 | Osteoporosis, Postmenopausal | 3.06 |
| NIPA2 | Osteoporosis, Postmenopausal | 3.06 |
| NMBR | Osteoporosis, Postmenopausal | 3.06 |
| NUP88 | Osteoporosis, Postmenopausal | 3.06 |
| PCDHB5 | Osteoporosis, Postmenopausal | 3.06 |
| PEX6 | Osteoporosis, Postmenopausal | 3.06 |
| POLDIP3 | Osteoporosis, Postmenopausal | 3.06 |
| PSG1 | Osteoporosis, Postmenopausal | 3.06 |
| R3HCC1L | Osteoporosis, Postmenopausal | 3.06 |
| RARS2 | Osteoporosis, Postmenopausal | 3.06 |
| REM1 | Osteoporosis, Postmenopausal | 3.06 |
| RTN4IP1 | Osteoporosis, Postmenopausal | 3.06 |
| SBDS | Osteoporosis, Postmenopausal | 3.06 |
| SERPINB4 | Osteoporosis, Postmenopausal | 3.06 |
| SNRNP48 | Osteoporosis, Postmenopausal | 3.06 |
| SNX15 | Osteoporosis, Postmenopausal | 3.06 |
| TAF9B | Osteoporosis, Postmenopausal | 3.06 |
| TDRD5 | Osteoporosis, Postmenopausal | 3.06 |
| TEX12 | Osteoporosis, Postmenopausal | 3.06 |
| TMCC2 | Osteoporosis, Postmenopausal | 3.06 |
| TMEM216 | Osteoporosis, Postmenopausal | 3.06 |
| TTC17 | Osteoporosis, Postmenopausal | 3.06 |
| USP43 | Osteoporosis, Postmenopausal | 3.06 |
| VWA2 | Osteoporosis, Postmenopausal | 3.06 |
| ZNF394 | Osteoporosis, Postmenopausal | 3.06 |
| ZNF622 | Osteoporosis, Postmenopausal | 3.06 |
| ZNF800 | Osteoporosis, Postmenopausal | 3.06 |
| ZSCAN16 | Osteoporosis, Postmenopausal | 3.06 |
| ZSWIM8 | Osteoporosis, Postmenopausal | 3.06 |
| BMP10 | Osteoporosis, Postmenopausal | 3.05 |
| BUD13 | Osteoporosis, Postmenopausal | 3.05 |
| CAMK1 | Osteoporosis, Postmenopausal | 3.05 |
| CEP295 | Osteoporosis, Postmenopausal | 3.05 |
| CNTNAP1 | Osteoporosis, Postmenopausal | 3.05 |
| CRX | Osteoporosis, Postmenopausal | 3.05 |
| DMAC1 | Osteoporosis, Postmenopausal | 3.05 |
| EDA2R | Osteoporosis, Postmenopausal | 3.05 |
| EXOC3L2 | Osteoporosis, Postmenopausal | 3.05 |
| FAM3A | Osteoporosis, Postmenopausal | 3.05 |
| FRA10AC1 | Osteoporosis, Postmenopausal | 3.05 |
| FYB2 | Osteoporosis, Postmenopausal | 3.05 |
| GPSM3 | Osteoporosis, Postmenopausal | 3.05 |
| GSTD1 | Osteoporosis, Postmenopausal | 3.05 |
| HELZ | Osteoporosis, Postmenopausal | 3.05 |
| INTS14 | Osteoporosis, Postmenopausal | 3.05 |
| KCNJ9 | Osteoporosis, Postmenopausal | 3.05 |
| KLHDC10 | Osteoporosis, Postmenopausal | 3.05 |
| LZIC | Osteoporosis, Postmenopausal | 3.05 |
| MAD2L1BP | Osteoporosis, Postmenopausal | 3.05 |
| METTL6 | Osteoporosis, Postmenopausal | 3.05 |
| MIP | Osteoporosis, Postmenopausal | 3.05 |
| MIR30D | Osteoporosis, Postmenopausal | 3.05 |
| ND3 | Osteoporosis, Postmenopausal | 3.05 |
| NKX6-1 | Osteoporosis, Postmenopausal | 3.05 |
| NPB | Osteoporosis, Postmenopausal | 3.05 |
| OSGIN2 | Osteoporosis, Postmenopausal | 3.05 |
| PCLO | Osteoporosis, Postmenopausal | 3.05 |
| PIANP | Osteoporosis, Postmenopausal | 3.05 |
| POGK | Osteoporosis, Postmenopausal | 3.05 |
| PPIG | Osteoporosis, Postmenopausal | 3.05 |
| RGS7BP | Osteoporosis, Postmenopausal | 3.05 |
| RIBC2 | Osteoporosis, Postmenopausal | 3.05 |
| RLF | Osteoporosis, Postmenopausal | 3.05 |
| RPP14 | Osteoporosis, Postmenopausal | 3.05 |
| RUSC1 | Osteoporosis, Postmenopausal | 3.05 |
| SNHG1 | Osteoporosis, Postmenopausal | 3.05 |
| SUPT6H | Osteoporosis, Postmenopausal | 3.05 |
| TANGO6 | Osteoporosis, Postmenopausal | 3.05 |
| TMEM39A | Osteoporosis, Postmenopausal | 3.05 |
| TNFAIP8L2 | Osteoporosis, Postmenopausal | 3.05 |
| UBE4A | Osteoporosis, Postmenopausal | 3.05 |
| UCHL5 | Osteoporosis, Postmenopausal | 3.05 |
| ZC3H4 | Osteoporosis, Postmenopausal | 3.05 |
| ZNF655 | Osteoporosis, Postmenopausal | 3.05 |
| ADAL | Osteoporosis, Postmenopausal | 3.04 |
| AIRE | Osteoporosis, Postmenopausal | 3.04 |
| AKNAD1 | Osteoporosis, Postmenopausal | 3.04 |
| ALKBH1 | Osteoporosis, Postmenopausal | 3.04 |
| APOL2 | Osteoporosis, Postmenopausal | 3.04 |
| ARF3 | Osteoporosis, Postmenopausal | 3.04 |
| BPIFA1 | Osteoporosis, Postmenopausal | 3.04 |
| BRD9 | Osteoporosis, Postmenopausal | 3.04 |
| C2ORF68 | Osteoporosis, Postmenopausal | 3.04 |
| CCDC137 | Osteoporosis, Postmenopausal | 3.04 |
| CCDC158 | Osteoporosis, Postmenopausal | 3.04 |
| CD79B | Osteoporosis, Postmenopausal | 3.04 |
| CDHR4 | Osteoporosis, Postmenopausal | 3.04 |
| CRISP1 | Osteoporosis, Postmenopausal | 3.04 |
| CYP27C1 | Osteoporosis, Postmenopausal | 3.04 |
| DCAF6 | Osteoporosis, Postmenopausal | 3.04 |
| DCTN3 | Osteoporosis, Postmenopausal | 3.04 |
| DGKG | Osteoporosis, Postmenopausal | 3.04 |
| DISC1 | Osteoporosis, Postmenopausal | 3.04 |
| FBXO24 | Osteoporosis, Postmenopausal | 3.04 |
| FSBP | Osteoporosis, Postmenopausal | 3.04 |
| FZD3 | Osteoporosis, Postmenopausal | 3.04 |
| GLYATL2 | Osteoporosis, Postmenopausal | 3.04 |
| HERC2 | Osteoporosis, Postmenopausal | 3.04 |
| HOOK2 | Osteoporosis, Postmenopausal | 3.04 |
| INSRR | Osteoporosis, Postmenopausal | 3.04 |
| JPH2 | Osteoporosis, Postmenopausal | 3.04 |
| KCNB2 | Osteoporosis, Postmenopausal | 3.04 |
| KDELR2 | Osteoporosis, Postmenopausal | 3.04 |
| KRT222 | Osteoporosis, Postmenopausal | 3.04 |
| LAX1 | Osteoporosis, Postmenopausal | 3.04 |
| LTB4R2 | Osteoporosis, Postmenopausal | 3.04 |
| MADCAM1 | Osteoporosis, Postmenopausal | 3.04 |
| MAPRE3 | Osteoporosis, Postmenopausal | 3.04 |
| MDGA2 | Osteoporosis, Postmenopausal | 3.04 |
| MEAF6 | Osteoporosis, Postmenopausal | 3.04 |
| MED29 | Osteoporosis, Postmenopausal | 3.04 |
| MIR30E | Osteoporosis, Postmenopausal | 3.04 |
| MOV10L1 | Osteoporosis, Postmenopausal | 3.04 |
| MTFR1 | Osteoporosis, Postmenopausal | 3.04 |
| MYH15 | Osteoporosis, Postmenopausal | 3.04 |
| NADK | Osteoporosis, Postmenopausal | 3.04 |
| NAXD | Osteoporosis, Postmenopausal | 3.04 |
| NR2C1 | Osteoporosis, Postmenopausal | 3.04 |
| NR2E3 | Osteoporosis, Postmenopausal | 3.04 |
| PGAP4 | Osteoporosis, Postmenopausal | 3.04 |
| POLR1D | Osteoporosis, Postmenopausal | 3.04 |
| PPM1N | Osteoporosis, Postmenopausal | 3.04 |
| PRICKLE3 | Osteoporosis, Postmenopausal | 3.04 |
| PXDC1 | Osteoporosis, Postmenopausal | 3.04 |
| RAB33B | Osteoporosis, Postmenopausal | 3.04 |
| RHBDD2 | Osteoporosis, Postmenopausal | 3.04 |
| SNHG17 | Osteoporosis, Postmenopausal | 3.04 |
| SSTR4 | Osteoporosis, Postmenopausal | 3.04 |
| TBX4 | Osteoporosis, Postmenopausal | 3.04 |
| TLR13 | Osteoporosis, Postmenopausal | 3.04 |
| TMEM167A | Osteoporosis, Postmenopausal | 3.04 |
| TMEM19 | Osteoporosis, Postmenopausal | 3.04 |
| TMEM238 | Osteoporosis, Postmenopausal | 3.04 |
| TPD52 | Osteoporosis, Postmenopausal | 3.04 |
| TPRG1 | Osteoporosis, Postmenopausal | 3.04 |
| TSNAXIP1 | Osteoporosis, Postmenopausal | 3.04 |
| USE1 | Osteoporosis, Postmenopausal | 3.04 |
| XAB2 | Osteoporosis, Postmenopausal | 3.04 |
| ZC3H14 | Osteoporosis, Postmenopausal | 3.04 |
| ZFP385A | Osteoporosis, Postmenopausal | 3.04 |
| ZNF226 | Osteoporosis, Postmenopausal | 3.04 |
| ZUP1 | Osteoporosis, Postmenopausal | 3.04 |
| ACOT6 | Osteoporosis, Postmenopausal | 3.03 |
| ADGRE2 | Osteoporosis, Postmenopausal | 3.03 |
| AKR1CL | Osteoporosis, Postmenopausal | 3.03 |
| AP1B1 | Osteoporosis, Postmenopausal | 3.03 |
| ATG9B | Osteoporosis, Postmenopausal | 3.03 |
| ATP13A5 | Osteoporosis, Postmenopausal | 3.03 |
| B3GALNT2 | Osteoporosis, Postmenopausal | 3.03 |
| BFSP2 | Osteoporosis, Postmenopausal | 3.03 |
| BRD10 | Osteoporosis, Postmenopausal | 3.03 |
| BTBD19 | Osteoporosis, Postmenopausal | 3.03 |
| CCDC12 | Osteoporosis, Postmenopausal | 3.03 |
| CDH26 | Osteoporosis, Postmenopausal | 3.03 |
| CEP162 | Osteoporosis, Postmenopausal | 3.03 |
| CFAP210 | Osteoporosis, Postmenopausal | 3.03 |
| CFAP36 | Osteoporosis, Postmenopausal | 3.03 |
| CFAP91 | Osteoporosis, Postmenopausal | 3.03 |
| CHRDL2 | Osteoporosis, Postmenopausal | 3.03 |
| COG2 | Osteoporosis, Postmenopausal | 3.03 |
| CPEB1 | Osteoporosis, Postmenopausal | 3.03 |
| CWF19L1 | Osteoporosis, Postmenopausal | 3.03 |
| CYP4F15 | Osteoporosis, Postmenopausal | 3.03 |
| DNAJC1 | Osteoporosis, Postmenopausal | 3.03 |
| DUSP15 | Osteoporosis, Postmenopausal | 3.03 |
| DYRK4 | Osteoporosis, Postmenopausal | 3.03 |
| ELAC1 | Osteoporosis, Postmenopausal | 3.03 |
| FAM162B | Osteoporosis, Postmenopausal | 3.03 |
| FANCM | Osteoporosis, Postmenopausal | 3.03 |
| FEZF2 | Osteoporosis, Postmenopausal | 3.03 |
| GDF1 | Osteoporosis, Postmenopausal | 3.03 |
| GPR75 | Osteoporosis, Postmenopausal | 3.03 |
| GTF2H5 | Osteoporosis, Postmenopausal | 3.03 |
| H1-0 | Osteoporosis, Postmenopausal | 3.03 |
| H2-K1 | Osteoporosis, Postmenopausal | 3.03 |
| H3-3A | Osteoporosis, Postmenopausal | 3.03 |
| HAGHL | Osteoporosis, Postmenopausal | 3.03 |
| HBD | Osteoporosis, Postmenopausal | 3.03 |
| HGS | Osteoporosis, Postmenopausal | 3.03 |
| IGDCC4 | Osteoporosis, Postmenopausal | 3.03 |
| INPP5E | Osteoporosis, Postmenopausal | 3.03 |
| KCP | Osteoporosis, Postmenopausal | 3.03 |
| LRRC31 | Osteoporosis, Postmenopausal | 3.03 |
| MAN1A2 | Osteoporosis, Postmenopausal | 3.03 |
| MEA1 | Osteoporosis, Postmenopausal | 3.03 |
| MIR15A | Osteoporosis, Postmenopausal | 3.03 |
| MIR204 | Osteoporosis, Postmenopausal | 3.03 |
| MIR330 | Osteoporosis, Postmenopausal | 3.03 |
| MIR375 | Osteoporosis, Postmenopausal | 3.03 |
| MOGS | Osteoporosis, Postmenopausal | 3.03 |
| MRPL36 | Osteoporosis, Postmenopausal | 3.03 |
| MRPL44 | Osteoporosis, Postmenopausal | 3.03 |
| MRPL50 | Osteoporosis, Postmenopausal | 3.03 |
| MRPS23 | Osteoporosis, Postmenopausal | 3.03 |
| MRPS9 | Osteoporosis, Postmenopausal | 3.03 |
| NMB | Osteoporosis, Postmenopausal | 3.03 |
| NUDT13 | Osteoporosis, Postmenopausal | 3.03 |
| PCP2 | Osteoporosis, Postmenopausal | 3.03 |
| PIN1 | Osteoporosis, Postmenopausal | 3.03 |
| PNKP | Osteoporosis, Postmenopausal | 3.03 |
| PNMA8B | Osteoporosis, Postmenopausal | 3.03 |
| PPP1R11 | Osteoporosis, Postmenopausal | 3.03 |
| PRPF40A | Osteoporosis, Postmenopausal | 3.03 |
| PRRG2 | Osteoporosis, Postmenopausal | 3.03 |
| RALBP1 | Osteoporosis, Postmenopausal | 3.03 |
| REC8 | Osteoporosis, Postmenopausal | 3.03 |
| RFX6 | Osteoporosis, Postmenopausal | 3.03 |
| RHCG | Osteoporosis, Postmenopausal | 3.03 |
| RPUSD1 | Osteoporosis, Postmenopausal | 3.03 |
| SAP25 | Osteoporosis, Postmenopausal | 3.03 |
| SCGB1D2 | Osteoporosis, Postmenopausal | 3.03 |
| SH2D1A | Osteoporosis, Postmenopausal | 3.03 |
| SPEF1 | Osteoporosis, Postmenopausal | 3.03 |
| TCERG1L | Osteoporosis, Postmenopausal | 3.03 |
| THTPA | Osteoporosis, Postmenopausal | 3.03 |
| TMED9 | Osteoporosis, Postmenopausal | 3.03 |
| TMUB2 | Osteoporosis, Postmenopausal | 3.03 |
| TRPM1 | Osteoporosis, Postmenopausal | 3.03 |
| TTYH3 | Osteoporosis, Postmenopausal | 3.03 |
| UBASH3A | Osteoporosis, Postmenopausal | 3.03 |
| VRK3 | Osteoporosis, Postmenopausal | 3.03 |
| WDR73 | Osteoporosis, Postmenopausal | 3.03 |
| YIF1B | Osteoporosis, Postmenopausal | 3.03 |
| ZFP346 | Osteoporosis, Postmenopausal | 3.03 |
| ZFP655 | Osteoporosis, Postmenopausal | 3.03 |
| ZFTA | Osteoporosis, Postmenopausal | 3.03 |
| ZNF451 | Osteoporosis, Postmenopausal | 3.03 |
| ZNF672 | Osteoporosis, Postmenopausal | 3.03 |
| ADGRL3 | Osteoporosis, Postmenopausal | 3.02 |
| ATCAY | Osteoporosis, Postmenopausal | 3.02 |
| BANP | Osteoporosis, Postmenopausal | 3.02 |
| BRF1 | Osteoporosis, Postmenopausal | 3.02 |
| CDK20 | Osteoporosis, Postmenopausal | 3.02 |
| EFNA3 | Osteoporosis, Postmenopausal | 3.02 |
| FAM149B1 | Osteoporosis, Postmenopausal | 3.02 |
| FBRS | Osteoporosis, Postmenopausal | 3.02 |
| IL17F | Osteoporosis, Postmenopausal | 3.02 |
| ILKAP | Osteoporosis, Postmenopausal | 3.02 |
| INIP | Osteoporosis, Postmenopausal | 3.02 |
| JKAMP | Osteoporosis, Postmenopausal | 3.02 |
| KCNAB1 | Osteoporosis, Postmenopausal | 3.02 |
| KIF3A | Osteoporosis, Postmenopausal | 3.02 |
| MED8 | Osteoporosis, Postmenopausal | 3.02 |
| MICA | Osteoporosis, Postmenopausal | 3.02 |
| MIR183 | Osteoporosis, Postmenopausal | 3.02 |
| MIR494 | Osteoporosis, Postmenopausal | 3.02 |
| MOB1A | Osteoporosis, Postmenopausal | 3.02 |
| MRPS15 | Osteoporosis, Postmenopausal | 3.02 |
| MXRA7 | Osteoporosis, Postmenopausal | 3.02 |
| NRN1 | Osteoporosis, Postmenopausal | 3.02 |
| OBI1 | Osteoporosis, Postmenopausal | 3.02 |
| PLXNB1 | Osteoporosis, Postmenopausal | 3.02 |
| POLR2K | Osteoporosis, Postmenopausal | 3.02 |
| PREPL | Osteoporosis, Postmenopausal | 3.02 |
| PTPRD | Osteoporosis, Postmenopausal | 3.02 |
| RNF20 | Osteoporosis, Postmenopausal | 3.02 |
| SIX1 | Osteoporosis, Postmenopausal | 3.02 |
| SLC10A4 | Osteoporosis, Postmenopausal | 3.02 |
| SLC24A4 | Osteoporosis, Postmenopausal | 3.02 |
| SPG7 | Osteoporosis, Postmenopausal | 3.02 |
| TAF2 | Osteoporosis, Postmenopausal | 3.02 |
| TFAP2E | Osteoporosis, Postmenopausal | 3.02 |
| TOPORS | Osteoporosis, Postmenopausal | 3.02 |
| TRIT1 | Osteoporosis, Postmenopausal | 3.02 |
| UGT2A3 | Osteoporosis, Postmenopausal | 3.02 |
| USH2A | Osteoporosis, Postmenopausal | 3.02 |
| VWA5B2 | Osteoporosis, Postmenopausal | 3.02 |
| ZNF296 | Osteoporosis, Postmenopausal | 3.02 |
| ACD | Osteoporosis, Postmenopausal | 3.01 |
| ANGEL1 | Osteoporosis, Postmenopausal | 3.01 |
| ANKRD35 | Osteoporosis, Postmenopausal | 3.01 |
| ARHGDIG | Osteoporosis, Postmenopausal | 3.01 |
| AVL9 | Osteoporosis, Postmenopausal | 3.01 |
| CACNG7 | Osteoporosis, Postmenopausal | 3.01 |
| CARMIL2 | Osteoporosis, Postmenopausal | 3.01 |
| CATSPERG | Osteoporosis, Postmenopausal | 3.01 |
| CCDC142 | Osteoporosis, Postmenopausal | 3.01 |
| CCDC170 | Osteoporosis, Postmenopausal | 3.01 |
| CDX1 | Osteoporosis, Postmenopausal | 3.01 |
| CEP120 | Osteoporosis, Postmenopausal | 3.01 |
| COLEC11 | Osteoporosis, Postmenopausal | 3.01 |
| CSNK1G2 | Osteoporosis, Postmenopausal | 3.01 |
| CST8 | Osteoporosis, Postmenopausal | 3.01 |
| CWF19L2 | Osteoporosis, Postmenopausal | 3.01 |
| DHX35 | Osteoporosis, Postmenopausal | 3.01 |
| DHX57 | Osteoporosis, Postmenopausal | 3.01 |
| DLX5 | Osteoporosis, Postmenopausal | 3.01 |
| FAM32A | Osteoporosis, Postmenopausal | 3.01 |
| FBLL1 | Osteoporosis, Postmenopausal | 3.01 |
| FSTL4 | Osteoporosis, Postmenopausal | 3.01 |
| GLIS1 | Osteoporosis, Postmenopausal | 3.01 |
| GPR137 | Osteoporosis, Postmenopausal | 3.01 |
| GTF2F1 | Osteoporosis, Postmenopausal | 3.01 |
| HARS2 | Osteoporosis, Postmenopausal | 3.01 |
| HCFC2 | Osteoporosis, Postmenopausal | 3.01 |
| HOTAIR | Osteoporosis, Postmenopausal | 3.01 |
| IL16 | Osteoporosis, Postmenopausal | 3.01 |
| IL19 | Osteoporosis, Postmenopausal | 3.01 |
| KCNK7 | Osteoporosis, Postmenopausal | 3.01 |
| KCNN1 | Osteoporosis, Postmenopausal | 3.01 |
| LLPH | Osteoporosis, Postmenopausal | 3.01 |
| MIB2 | Osteoporosis, Postmenopausal | 3.01 |
| MIR146B | Osteoporosis, Postmenopausal | 3.01 |
| NAP1L1 | Osteoporosis, Postmenopausal | 3.01 |
| PKNOX1 | Osteoporosis, Postmenopausal | 3.01 |
| POU3F2 | Osteoporosis, Postmenopausal | 3.01 |
| PTBP2 | Osteoporosis, Postmenopausal | 3.01 |
| PTH2R | Osteoporosis, Postmenopausal | 3.01 |
| RAB6B | Osteoporosis, Postmenopausal | 3.01 |
| RBM12B | Osteoporosis, Postmenopausal | 3.01 |
| RGS9BP | Osteoporosis, Postmenopausal | 3.01 |
| RIC8B | Osteoporosis, Postmenopausal | 3.01 |
| RPS23 | Osteoporosis, Postmenopausal | 3.01 |
| RRAGB | Osteoporosis, Postmenopausal | 3.01 |
| RSPH3 | Osteoporosis, Postmenopausal | 3.01 |
| RSPH4A | Osteoporosis, Postmenopausal | 3.01 |
| SDF2 | Osteoporosis, Postmenopausal | 3.01 |
| SEPHS1 | Osteoporosis, Postmenopausal | 3.01 |
| SERP1 | Osteoporosis, Postmenopausal | 3.01 |
| SHLD2 | Osteoporosis, Postmenopausal | 3.01 |
| SLC2A8 | Osteoporosis, Postmenopausal | 3.01 |
| SPRYD4 | Osteoporosis, Postmenopausal | 3.01 |
| TIMM22 | Osteoporosis, Postmenopausal | 3.01 |
| TMEM241 | Osteoporosis, Postmenopausal | 3.01 |
| TTC39B | Osteoporosis, Postmenopausal | 3.01 |
| TXNDC12 | Osteoporosis, Postmenopausal | 3.01 |
| UMODL1 | Osteoporosis, Postmenopausal | 3.01 |
| UNC119B | Osteoporosis, Postmenopausal | 3.01 |
| VWA7 | Osteoporosis, Postmenopausal | 3.01 |
| YKT6 | Osteoporosis, Postmenopausal | 3.01 |
| ZDHHC16 | Osteoporosis, Postmenopausal | 3.01 |
| ZFP462 | Osteoporosis, Postmenopausal | 3.01 |
| ZNF354A | Osteoporosis, Postmenopausal | 3.01 |
| ZNF638 | Osteoporosis, Postmenopausal | 3.01 |
| ZSCAN12 | Osteoporosis, Postmenopausal | 3.01 |
| AGK | Osteoporosis, Postmenopausal | 3 |
| ARMH1 | Osteoporosis, Postmenopausal | 3 |
| ARRDC1 | Osteoporosis, Postmenopausal | 3 |
| CCDC159 | Osteoporosis, Postmenopausal | 3 |
| CDC37 | Osteoporosis, Postmenopausal | 3 |
| CEACAM3 | Osteoporosis, Postmenopausal | 3 |
| CEP43 | Osteoporosis, Postmenopausal | 3 |
| CYP2A3 | Osteoporosis, Postmenopausal | 3 |
| CYP2C38 | Osteoporosis, Postmenopausal | 3 |
| CYP2D9 | Osteoporosis, Postmenopausal | 3 |
| DCTN5 | Osteoporosis, Postmenopausal | 3 |
| DNAAF5 | Osteoporosis, Postmenopausal | 3 |
| DYNLRB1 | Osteoporosis, Postmenopausal | 3 |
| EPGN | Osteoporosis, Postmenopausal | 3 |
| ESAM | Osteoporosis, Postmenopausal | 3 |
| EXOG | Osteoporosis, Postmenopausal | 3 |
| EXOSC10 | Osteoporosis, Postmenopausal | 3 |
| FAM217B | Osteoporosis, Postmenopausal | 3 |
| FANCF | Osteoporosis, Postmenopausal | 3 |
| FOXJ2 | Osteoporosis, Postmenopausal | 3 |
| FOXN1 | Osteoporosis, Postmenopausal | 3 |
| GFM2 | Osteoporosis, Postmenopausal | 3 |
| GTF2IRD2 | Osteoporosis, Postmenopausal | 3 |
| HOXD9 | Osteoporosis, Postmenopausal | 3 |
| INSM1 | Osteoporosis, Postmenopausal | 3 |
| IRGM1 | Osteoporosis, Postmenopausal | 3 |
| JMJD7 | Osteoporosis, Postmenopausal | 3 |
| KPNA5 | Osteoporosis, Postmenopausal | 3 |
| KRI1 | Osteoporosis, Postmenopausal | 3 |
| LMF2 | Osteoporosis, Postmenopausal | 3 |
| LSM8 | Osteoporosis, Postmenopausal | 3 |
| MAMDC4 | Osteoporosis, Postmenopausal | 3 |
| MAST1 | Osteoporosis, Postmenopausal | 3 |
| MED17 | Osteoporosis, Postmenopausal | 3 |
| MEGF11 | Osteoporosis, Postmenopausal | 3 |
| MIR130A | Osteoporosis, Postmenopausal | 3 |
| MIR23A | Osteoporosis, Postmenopausal | 3 |
| MIR320A | Osteoporosis, Postmenopausal | 3 |
| MIR335 | Osteoporosis, Postmenopausal | 3 |
| MIR96 | Osteoporosis, Postmenopausal | 3 |
| MORF4L1 | Osteoporosis, Postmenopausal | 3 |
| MRPS25 | Osteoporosis, Postmenopausal | 3 |
| MTX3 | Osteoporosis, Postmenopausal | 3 |
| MVB12A | Osteoporosis, Postmenopausal | 3 |
| NAA30 | Osteoporosis, Postmenopausal | 3 |
| NAA40 | Osteoporosis, Postmenopausal | 3 |
| NARS2 | Osteoporosis, Postmenopausal | 3 |
| OCRL | Osteoporosis, Postmenopausal | 3 |
| OTOGL | Osteoporosis, Postmenopausal | 3 |
| PALM3 | Osteoporosis, Postmenopausal | 3 |
| PDCL | Osteoporosis, Postmenopausal | 3 |
| PDIA2 | Osteoporosis, Postmenopausal | 3 |
| PLSCR3 | Osteoporosis, Postmenopausal | 3 |
| PNMA8A | Osteoporosis, Postmenopausal | 3 |
| POLR2E | Osteoporosis, Postmenopausal | 3 |
| PSMG3 | Osteoporosis, Postmenopausal | 3 |
| PSTK | Osteoporosis, Postmenopausal | 3 |
| RALGAPB | Osteoporosis, Postmenopausal | 3 |
| RBCK1 | Osteoporosis, Postmenopausal | 3 |
| RHBDL3 | Osteoporosis, Postmenopausal | 3 |
| RNF168 | Osteoporosis, Postmenopausal | 3 |
| RNF24 | Osteoporosis, Postmenopausal | 3 |
| RSKR | Osteoporosis, Postmenopausal | 3 |
| SCO1 | Osteoporosis, Postmenopausal | 3 |
| SELENOS | Osteoporosis, Postmenopausal | 3 |
| SKIC3 | Osteoporosis, Postmenopausal | 3 |
| SLC16A8 | Osteoporosis, Postmenopausal | 3 |
| SLC35B3 | Osteoporosis, Postmenopausal | 3 |
| SNAPC5 | Osteoporosis, Postmenopausal | 3 |
| SNX8 | Osteoporosis, Postmenopausal | 3 |
| SPAG8 | Osteoporosis, Postmenopausal | 3 |
| SPRY2 | Osteoporosis, Postmenopausal | 3 |
| STING1 | Osteoporosis, Postmenopausal | 3 |
| TESC | Osteoporosis, Postmenopausal | 3 |
| TMEM11 | Osteoporosis, Postmenopausal | 3 |
| TMEM258 | Osteoporosis, Postmenopausal | 3 |
| TNP2 | Osteoporosis, Postmenopausal | 3 |
| TPRG1L | Osteoporosis, Postmenopausal | 3 |
| TRIM68 | Osteoporosis, Postmenopausal | 3 |
| UBXN10 | Osteoporosis, Postmenopausal | 3 |
| USP21 | Osteoporosis, Postmenopausal | 3 |
| VANGL1 | Osteoporosis, Postmenopausal | 3 |
| VEZT | Osteoporosis, Postmenopausal | 3 |
| VPS11 | Osteoporosis, Postmenopausal | 3 |
| WDR37 | Osteoporosis, Postmenopausal | 3 |
| ZFP292 | Osteoporosis, Postmenopausal | 3 |
| ZNF224 | Osteoporosis, Postmenopausal | 3 |
| AK8 | Osteoporosis, Postmenopausal | 2.99 |
| ALG9 | Osteoporosis, Postmenopausal | 2.99 |
| ATP6V0B | Osteoporosis, Postmenopausal | 2.99 |
| BBLN | Osteoporosis, Postmenopausal | 2.99 |
| CCDC134 | Osteoporosis, Postmenopausal | 2.99 |
| CD302 | Osteoporosis, Postmenopausal | 2.99 |
| CFAP77 | Osteoporosis, Postmenopausal | 2.99 |
| CFAP96 | Osteoporosis, Postmenopausal | 2.99 |
| COL11A2 | Osteoporosis, Postmenopausal | 2.99 |
| CPA5 | Osteoporosis, Postmenopausal | 2.99 |
| DCAF10 | Osteoporosis, Postmenopausal | 2.99 |
| DMRTA1 | Osteoporosis, Postmenopausal | 2.99 |
| DNAJC4 | Osteoporosis, Postmenopausal | 2.99 |
| EIF4E2 | Osteoporosis, Postmenopausal | 2.99 |
| ERICH3 | Osteoporosis, Postmenopausal | 2.99 |
| EXOC4 | Osteoporosis, Postmenopausal | 2.99 |
| EYA3 | Osteoporosis, Postmenopausal | 2.99 |
| FAM47E | Osteoporosis, Postmenopausal | 2.99 |
| FBXL19 | Osteoporosis, Postmenopausal | 2.99 |
| FNDC10 | Osteoporosis, Postmenopausal | 2.99 |
| FOXN4 | Osteoporosis, Postmenopausal | 2.99 |
| FSCN2 | Osteoporosis, Postmenopausal | 2.99 |
| GNAL | Osteoporosis, Postmenopausal | 2.99 |
| H1F5 | Osteoporosis, Postmenopausal | 2.99 |
| H2AC1 | Osteoporosis, Postmenopausal | 2.99 |
| H2BC6 | Osteoporosis, Postmenopausal | 2.99 |
| HAPLN4 | Osteoporosis, Postmenopausal | 2.99 |
| HCAR1 | Osteoporosis, Postmenopausal | 2.99 |
| HMX2 | Osteoporosis, Postmenopausal | 2.99 |
| KRT81 | Osteoporosis, Postmenopausal | 2.99 |
| LRRC39 | Osteoporosis, Postmenopausal | 2.99 |
| MAGOHB | Osteoporosis, Postmenopausal | 2.99 |
| MIR193B | Osteoporosis, Postmenopausal | 2.99 |
| MORN5 | Osteoporosis, Postmenopausal | 2.99 |
| MTX1 | Osteoporosis, Postmenopausal | 2.99 |
| MUTYH | Osteoporosis, Postmenopausal | 2.99 |
| NABP2 | Osteoporosis, Postmenopausal | 2.99 |
| NONO | Osteoporosis, Postmenopausal | 2.99 |
| NPVF | Osteoporosis, Postmenopausal | 2.99 |
| OLIG3 | Osteoporosis, Postmenopausal | 2.99 |
| OSBPL8 | Osteoporosis, Postmenopausal | 2.99 |
| PAF1 | Osteoporosis, Postmenopausal | 2.99 |
| PGA5 | Osteoporosis, Postmenopausal | 2.99 |
| PHETA2 | Osteoporosis, Postmenopausal | 2.99 |
| PHF20 | Osteoporosis, Postmenopausal | 2.99 |
| PHF7 | Osteoporosis, Postmenopausal | 2.99 |
| PITPNM1 | Osteoporosis, Postmenopausal | 2.99 |
| PPP1R42 | Osteoporosis, Postmenopausal | 2.99 |
| RD3 | Osteoporosis, Postmenopausal | 2.99 |
| RRP8 | Osteoporosis, Postmenopausal | 2.99 |
| RT1-BA | Osteoporosis, Postmenopausal | 2.99 |
| SLC26A10 | Osteoporosis, Postmenopausal | 2.99 |
| SLC9A4 | Osteoporosis, Postmenopausal | 2.99 |
| SMIM8 | Osteoporosis, Postmenopausal | 2.99 |
| SPATS2 | Osteoporosis, Postmenopausal | 2.99 |
| SRP54 | Osteoporosis, Postmenopausal | 2.99 |
| STX19 | Osteoporosis, Postmenopausal | 2.99 |
| STX4 | Osteoporosis, Postmenopausal | 2.99 |
| SYN3 | Osteoporosis, Postmenopausal | 2.99 |
| TACR2 | Osteoporosis, Postmenopausal | 2.99 |
| TAS1R3 | Osteoporosis, Postmenopausal | 2.99 |
| TIFAB | Osteoporosis, Postmenopausal | 2.99 |
| TLR12 | Osteoporosis, Postmenopausal | 2.99 |
| TPGS1 | Osteoporosis, Postmenopausal | 2.99 |
| TRIML2 | Osteoporosis, Postmenopausal | 2.99 |
| TTC19 | Osteoporosis, Postmenopausal | 2.99 |
| TYRO3 | Osteoporosis, Postmenopausal | 2.99 |
| UROS | Osteoporosis, Postmenopausal | 2.99 |
| WDR59 | Osteoporosis, Postmenopausal | 2.99 |
| ZFP52 | Osteoporosis, Postmenopausal | 2.99 |
| ZNF280C | Osteoporosis, Postmenopausal | 2.99 |
| ABCA8A | Osteoporosis, Postmenopausal | 2.98 |
| CCDC120 | Osteoporosis, Postmenopausal | 2.98 |
| CDC40 | Osteoporosis, Postmenopausal | 2.98 |
| CELF1 | Osteoporosis, Postmenopausal | 2.98 |
| CLK2 | Osteoporosis, Postmenopausal | 2.98 |
| CPSF1 | Osteoporosis, Postmenopausal | 2.98 |
| ENTREP3 | Osteoporosis, Postmenopausal | 2.98 |
| FOXRED1 | Osteoporosis, Postmenopausal | 2.98 |
| GLCCI1 | Osteoporosis, Postmenopausal | 2.98 |
| GTF2H1 | Osteoporosis, Postmenopausal | 2.98 |
| HACE1 | Osteoporosis, Postmenopausal | 2.98 |
| IL27 | Osteoporosis, Postmenopausal | 2.98 |
| LMAN2 | Osteoporosis, Postmenopausal | 2.98 |
| LY6G6C | Osteoporosis, Postmenopausal | 2.98 |
| MRPL57 | Osteoporosis, Postmenopausal | 2.98 |
| MRPS11 | Osteoporosis, Postmenopausal | 2.98 |
| NAGLU | Osteoporosis, Postmenopausal | 2.98 |
| OSGEP | Osteoporosis, Postmenopausal | 2.98 |
| PIGF | Osteoporosis, Postmenopausal | 2.98 |
| PIGM | Osteoporosis, Postmenopausal | 2.98 |
| PLEKHA8 | Osteoporosis, Postmenopausal | 2.98 |
| PQBP1 | Osteoporosis, Postmenopausal | 2.98 |
| PTPN14 | Osteoporosis, Postmenopausal | 2.98 |
| SF3B4 | Osteoporosis, Postmenopausal | 2.98 |
| SMU1 | Osteoporosis, Postmenopausal | 2.98 |
| TBCA | Osteoporosis, Postmenopausal | 2.98 |
| TCAP | Osteoporosis, Postmenopausal | 2.98 |
| TRAPPC6B | Osteoporosis, Postmenopausal | 2.98 |
| TRIM31 | Osteoporosis, Postmenopausal | 2.98 |
| TRMT12 | Osteoporosis, Postmenopausal | 2.98 |
| UGT2B5 | Osteoporosis, Postmenopausal | 2.98 |
| AGPAT9 | Osteoporosis, Postmenopausal | 2.97 |
| ANKRD46 | Osteoporosis, Postmenopausal | 2.97 |
| APOC4 | Osteoporosis, Postmenopausal | 2.97 |
| AURKC | Osteoporosis, Postmenopausal | 2.97 |
| BNIPL | Osteoporosis, Postmenopausal | 2.97 |
| CCDC127 | Osteoporosis, Postmenopausal | 2.97 |
| CD1D | Osteoporosis, Postmenopausal | 2.97 |
| CD8B1 | Osteoporosis, Postmenopausal | 2.97 |
| CFAP300 | Osteoporosis, Postmenopausal | 2.97 |
| COA4 | Osteoporosis, Postmenopausal | 2.97 |
| COG7 | Osteoporosis, Postmenopausal | 2.97 |
| CPTP | Osteoporosis, Postmenopausal | 2.97 |
| CRACR2B | Osteoporosis, Postmenopausal | 2.97 |
| DCTN6 | Osteoporosis, Postmenopausal | 2.97 |
| DENND6A | Osteoporosis, Postmenopausal | 2.97 |
| DHX16 | Osteoporosis, Postmenopausal | 2.97 |
| DNAJC18 | Osteoporosis, Postmenopausal | 2.97 |
| DPCD | Osteoporosis, Postmenopausal | 2.97 |
| DZIP1 | Osteoporosis, Postmenopausal | 2.97 |
| ELP5 | Osteoporosis, Postmenopausal | 2.97 |
| FAM110D | Osteoporosis, Postmenopausal | 2.97 |
| FAM151A | Osteoporosis, Postmenopausal | 2.97 |
| FAM227B | Osteoporosis, Postmenopausal | 2.97 |
| FAM89B | Osteoporosis, Postmenopausal | 2.97 |
| FCRLB | Osteoporosis, Postmenopausal | 2.97 |
| FN3KRP | Osteoporosis, Postmenopausal | 2.97 |
| GCSAM | Osteoporosis, Postmenopausal | 2.97 |
| GIMAP1 | Osteoporosis, Postmenopausal | 2.97 |
| GPRASP3 | Osteoporosis, Postmenopausal | 2.97 |
| GRM7 | Osteoporosis, Postmenopausal | 2.97 |
| H3C3 | Osteoporosis, Postmenopausal | 2.97 |
| HCCS | Osteoporosis, Postmenopausal | 2.97 |
| HDGFL2 | Osteoporosis, Postmenopausal | 2.97 |
| IMMT | Osteoporosis, Postmenopausal | 2.97 |
| IPPK | Osteoporosis, Postmenopausal | 2.97 |
| IQSEC3 | Osteoporosis, Postmenopausal | 2.97 |
| ITPKA | Osteoporosis, Postmenopausal | 2.97 |
| KIAA0319 | Osteoporosis, Postmenopausal | 2.97 |
| KICS2 | Osteoporosis, Postmenopausal | 2.97 |
| LY6C1 | Osteoporosis, Postmenopausal | 2.97 |
| MIEN1 | Osteoporosis, Postmenopausal | 2.97 |
| MIGA2 | Osteoporosis, Postmenopausal | 2.97 |
| MIR135B | Osteoporosis, Postmenopausal | 2.97 |
| MIR99AHG | Osteoporosis, Postmenopausal | 2.97 |
| NDUFAF3 | Osteoporosis, Postmenopausal | 2.97 |
| NEK11 | Osteoporosis, Postmenopausal | 2.97 |
| NODAL | Osteoporosis, Postmenopausal | 2.97 |
| NTMT1 | Osteoporosis, Postmenopausal | 2.97 |
| PARN | Osteoporosis, Postmenopausal | 2.97 |
| PCID2 | Osteoporosis, Postmenopausal | 2.97 |
| PEDS1 | Osteoporosis, Postmenopausal | 2.97 |
| PRPSAP2 | Osteoporosis, Postmenopausal | 2.97 |
| PRR32 | Osteoporosis, Postmenopausal | 2.97 |
| RBPJL | Osteoporosis, Postmenopausal | 2.97 |
| RIBC1 | Osteoporosis, Postmenopausal | 2.97 |
| RNASEK | Osteoporosis, Postmenopausal | 2.97 |
| RTN1 | Osteoporosis, Postmenopausal | 2.97 |
| SDHC | Osteoporosis, Postmenopausal | 2.97 |
| SEC23B | Osteoporosis, Postmenopausal | 2.97 |
| SERPINA12 | Osteoporosis, Postmenopausal | 2.97 |
| SMIM11 | Osteoporosis, Postmenopausal | 2.97 |
| SMIM7 | Osteoporosis, Postmenopausal | 2.97 |
| SRRM4 | Osteoporosis, Postmenopausal | 2.97 |
| ST8SIA5 | Osteoporosis, Postmenopausal | 2.97 |
| SYT10 | Osteoporosis, Postmenopausal | 2.97 |
| TMED8 | Osteoporosis, Postmenopausal | 2.97 |
| TMEM229A | Osteoporosis, Postmenopausal | 2.97 |
| VAMP3 | Osteoporosis, Postmenopausal | 2.97 |
| WASHC4 | Osteoporosis, Postmenopausal | 2.97 |
| WDPCP | Osteoporosis, Postmenopausal | 2.97 |
| YIPF4 | Osteoporosis, Postmenopausal | 2.97 |
| ZBTB9 | Osteoporosis, Postmenopausal | 2.97 |
| ZFTRAF1 | Osteoporosis, Postmenopausal | 2.97 |
| ZKSCAN3 | Osteoporosis, Postmenopausal | 2.97 |
| ZNF365 | Osteoporosis, Postmenopausal | 2.97 |
| ZNF627 | Osteoporosis, Postmenopausal | 2.97 |
| ACKR1 | Osteoporosis, Postmenopausal | 2.96 |
| AFG2B | Osteoporosis, Postmenopausal | 2.96 |
| AGGF1 | Osteoporosis, Postmenopausal | 2.96 |
| AKAP5 | Osteoporosis, Postmenopausal | 2.96 |
| AKR1C12 | Osteoporosis, Postmenopausal | 2.96 |
| ALK | Osteoporosis, Postmenopausal | 2.96 |
| AMER2 | Osteoporosis, Postmenopausal | 2.96 |
| ANKRD13C | Osteoporosis, Postmenopausal | 2.96 |
| APON | Osteoporosis, Postmenopausal | 2.96 |
| ARL2 | Osteoporosis, Postmenopausal | 2.96 |
| ARMC1 | Osteoporosis, Postmenopausal | 2.96 |
| BABAM1 | Osteoporosis, Postmenopausal | 2.96 |
| BPTF | Osteoporosis, Postmenopausal | 2.96 |
| C4ORF19 | Osteoporosis, Postmenopausal | 2.96 |
| CCDC103 | Osteoporosis, Postmenopausal | 2.96 |
| CDK9 | Osteoporosis, Postmenopausal | 2.96 |
| CHP2 | Osteoporosis, Postmenopausal | 2.96 |
| CISD2 | Osteoporosis, Postmenopausal | 2.96 |
| CNTNAP3 | Osteoporosis, Postmenopausal | 2.96 |
| COMMD1 | Osteoporosis, Postmenopausal | 2.96 |
| CSN3 | Osteoporosis, Postmenopausal | 2.96 |
| DNAAF4 | Osteoporosis, Postmenopausal | 2.96 |
| DNAJC8 | Osteoporosis, Postmenopausal | 2.96 |
| FBXW7 | Osteoporosis, Postmenopausal | 2.96 |
| FUS | Osteoporosis, Postmenopausal | 2.96 |
| GDI2 | Osteoporosis, Postmenopausal | 2.96 |
| H1F0 | Osteoporosis, Postmenopausal | 2.96 |
| IGHA | Osteoporosis, Postmenopausal | 2.96 |
| IL9 | Osteoporosis, Postmenopausal | 2.96 |
| IPP | Osteoporosis, Postmenopausal | 2.96 |
| KBTBD2 | Osteoporosis, Postmenopausal | 2.96 |
| KLHDC3 | Osteoporosis, Postmenopausal | 2.96 |
| LAMTOR5 | Osteoporosis, Postmenopausal | 2.96 |
| LMNTD2 | Osteoporosis, Postmenopausal | 2.96 |
| MAP6D1 | Osteoporosis, Postmenopausal | 2.96 |
| MAST3 | Osteoporosis, Postmenopausal | 2.96 |
| MCMDC2 | Osteoporosis, Postmenopausal | 2.96 |
| MIDN | Osteoporosis, Postmenopausal | 2.96 |
| MIR423 | Osteoporosis, Postmenopausal | 2.96 |
| NEUROD2 | Osteoporosis, Postmenopausal | 2.96 |
| NLRC3 | Osteoporosis, Postmenopausal | 2.96 |
| NPY5R | Osteoporosis, Postmenopausal | 2.96 |
| PHACTR2 | Osteoporosis, Postmenopausal | 2.96 |
| POLR2D | Osteoporosis, Postmenopausal | 2.96 |
| PRDM9 | Osteoporosis, Postmenopausal | 2.96 |
| RAB11FIP4 | Osteoporosis, Postmenopausal | 2.96 |
| RAB3GAP1 | Osteoporosis, Postmenopausal | 2.96 |
| RDH14 | Osteoporosis, Postmenopausal | 2.96 |
| RPAIN | Osteoporosis, Postmenopausal | 2.96 |
| RPS18 | Osteoporosis, Postmenopausal | 2.96 |
| S100A5 | Osteoporosis, Postmenopausal | 2.96 |
| SCAI | Osteoporosis, Postmenopausal | 2.96 |
| SCN2B | Osteoporosis, Postmenopausal | 2.96 |
| SIAH1A | Osteoporosis, Postmenopausal | 2.96 |
| SLC34A1 | Osteoporosis, Postmenopausal | 2.96 |
| SLF2 | Osteoporosis, Postmenopausal | 2.96 |
| SPG11 | Osteoporosis, Postmenopausal | 2.96 |
| SPRR2D | Osteoporosis, Postmenopausal | 2.96 |
| SPTBN5 | Osteoporosis, Postmenopausal | 2.96 |
| TBC1D23 | Osteoporosis, Postmenopausal | 2.96 |
| TGOLN2 | Osteoporosis, Postmenopausal | 2.96 |
| THG1L | Osteoporosis, Postmenopausal | 2.96 |
| TRAPPC1 | Osteoporosis, Postmenopausal | 2.96 |
| TRPM5 | Osteoporosis, Postmenopausal | 2.96 |
| TTC7B | Osteoporosis, Postmenopausal | 2.96 |
| UBALD1 | Osteoporosis, Postmenopausal | 2.96 |
| UFD1 | Osteoporosis, Postmenopausal | 2.96 |
| VPS25 | Osteoporosis, Postmenopausal | 2.96 |
| VTI1B | Osteoporosis, Postmenopausal | 2.96 |
| ABHD14A | Osteoporosis, Postmenopausal | 2.95 |
| ABHD17A | Osteoporosis, Postmenopausal | 2.95 |
| ACTR10 | Osteoporosis, Postmenopausal | 2.95 |
| AFG1L | Osteoporosis, Postmenopausal | 2.95 |
| AHSA2P | Osteoporosis, Postmenopausal | 2.95 |
| AKR1C13 | Osteoporosis, Postmenopausal | 2.95 |
| AMY1A | Osteoporosis, Postmenopausal | 2.95 |
| ARHGAP45 | Osteoporosis, Postmenopausal | 2.95 |
| ARMC3 | Osteoporosis, Postmenopausal | 2.95 |
| ASB14 | Osteoporosis, Postmenopausal | 2.95 |
| ATP1B4 | Osteoporosis, Postmenopausal | 2.95 |
| C12ORF57 | Osteoporosis, Postmenopausal | 2.95 |
| C1QTNF12 | Osteoporosis, Postmenopausal | 2.95 |
| CCDC13 | Osteoporosis, Postmenopausal | 2.95 |
| CCDC74A | Osteoporosis, Postmenopausal | 2.95 |
| CEP15 | Osteoporosis, Postmenopausal | 2.95 |
| CERS2 | Osteoporosis, Postmenopausal | 2.95 |
| CLCC1 | Osteoporosis, Postmenopausal | 2.95 |
| DDA1 | Osteoporosis, Postmenopausal | 2.95 |
| EDEM2 | Osteoporosis, Postmenopausal | 2.95 |
| ELAPOR2 | Osteoporosis, Postmenopausal | 2.95 |
| GGA2 | Osteoporosis, Postmenopausal | 2.95 |
| GLRA3 | Osteoporosis, Postmenopausal | 2.95 |
| GLYATL1 | Osteoporosis, Postmenopausal | 2.95 |
| GSDMA | Osteoporosis, Postmenopausal | 2.95 |
| GTF3A | Osteoporosis, Postmenopausal | 2.95 |
| H3C6 | Osteoporosis, Postmenopausal | 2.95 |
| HSPB11 | Osteoporosis, Postmenopausal | 2.95 |
| INTS2 | Osteoporosis, Postmenopausal | 2.95 |
| KRT79 | Osteoporosis, Postmenopausal | 2.95 |
| LIN54 | Osteoporosis, Postmenopausal | 2.95 |
| LRFN4 | Osteoporosis, Postmenopausal | 2.95 |
| MIR143 | Osteoporosis, Postmenopausal | 2.95 |
| MPLKIP | Osteoporosis, Postmenopausal | 2.95 |
| MRPL32 | Osteoporosis, Postmenopausal | 2.95 |
| MRPL34 | Osteoporosis, Postmenopausal | 2.95 |
| MTO1 | Osteoporosis, Postmenopausal | 2.95 |
| MYCBPAP | Osteoporosis, Postmenopausal | 2.95 |
| NDUFAF8 | Osteoporosis, Postmenopausal | 2.95 |
| NOL11 | Osteoporosis, Postmenopausal | 2.95 |
| NPM3 | Osteoporosis, Postmenopausal | 2.95 |
| POLR2G | Osteoporosis, Postmenopausal | 2.95 |
| RFC1 | Osteoporosis, Postmenopausal | 2.95 |
| RNF32 | Osteoporosis, Postmenopausal | 2.95 |
| RTL6 | Osteoporosis, Postmenopausal | 2.95 |
| RXFP1 | Osteoporosis, Postmenopausal | 2.95 |
| SLC25A18 | Osteoporosis, Postmenopausal | 2.95 |
| SLC5A12 | Osteoporosis, Postmenopausal | 2.95 |
| SLFN4 | Osteoporosis, Postmenopausal | 2.95 |
| SNTG1 | Osteoporosis, Postmenopausal | 2.95 |
| SRMS | Osteoporosis, Postmenopausal | 2.95 |
| STK19 | Osteoporosis, Postmenopausal | 2.95 |
| STX17 | Osteoporosis, Postmenopausal | 2.95 |
| SYT5 | Osteoporosis, Postmenopausal | 2.95 |
| TEKT2 | Osteoporosis, Postmenopausal | 2.95 |
| TMEM178B | Osteoporosis, Postmenopausal | 2.95 |
| TNFRSF13C | Osteoporosis, Postmenopausal | 2.95 |
| TPGS2 | Osteoporosis, Postmenopausal | 2.95 |
| TTLL4 | Osteoporosis, Postmenopausal | 2.95 |
| WDR31 | Osteoporosis, Postmenopausal | 2.95 |
| ZDHHC1 | Osteoporosis, Postmenopausal | 2.95 |
| ZFP62 | Osteoporosis, Postmenopausal | 2.95 |
| ZNF239 | Osteoporosis, Postmenopausal | 2.95 |
| AGPAT1 | Osteoporosis, Postmenopausal | 2.94 |
| AMMECR1L | Osteoporosis, Postmenopausal | 2.94 |
| APEH | Osteoporosis, Postmenopausal | 2.94 |
| ASB4 | Osteoporosis, Postmenopausal | 2.94 |
| B4GALNT3 | Osteoporosis, Postmenopausal | 2.94 |
| B4GALNT4 | Osteoporosis, Postmenopausal | 2.94 |
| B4GALT7 | Osteoporosis, Postmenopausal | 2.94 |
| BRSK1 | Osteoporosis, Postmenopausal | 2.94 |
| C6ORF141 | Osteoporosis, Postmenopausal | 2.94 |
| CABCOCO1 | Osteoporosis, Postmenopausal | 2.94 |
| CDC123 | Osteoporosis, Postmenopausal | 2.94 |
| CFAP97 | Osteoporosis, Postmenopausal | 2.94 |
| CHD3 | Osteoporosis, Postmenopausal | 2.94 |
| DCUN1D3 | Osteoporosis, Postmenopausal | 2.94 |
| DDN | Osteoporosis, Postmenopausal | 2.94 |
| DKKL1 | Osteoporosis, Postmenopausal | 2.94 |
| DNAL4 | Osteoporosis, Postmenopausal | 2.94 |
| DRAXIN | Osteoporosis, Postmenopausal | 2.94 |
| EFHB | Osteoporosis, Postmenopausal | 2.94 |
| ERCC6L2 | Osteoporosis, Postmenopausal | 2.94 |
| FAM91A1 | Osteoporosis, Postmenopausal | 2.94 |
| FLYWCH1 | Osteoporosis, Postmenopausal | 2.94 |
| FSD1 | Osteoporosis, Postmenopausal | 2.94 |
| GCC1 | Osteoporosis, Postmenopausal | 2.94 |
| GMIP | Osteoporosis, Postmenopausal | 2.94 |
| H1F10 | Osteoporosis, Postmenopausal | 2.94 |
| HUS1 | Osteoporosis, Postmenopausal | 2.94 |
| HYDIN | Osteoporosis, Postmenopausal | 2.94 |
| IFT52 | Osteoporosis, Postmenopausal | 2.94 |
| IKZF4 | Osteoporosis, Postmenopausal | 2.94 |
| IL11RA | Osteoporosis, Postmenopausal | 2.94 |
| IL31RA | Osteoporosis, Postmenopausal | 2.94 |
| KCTD15 | Osteoporosis, Postmenopausal | 2.94 |
| KRT9 | Osteoporosis, Postmenopausal | 2.94 |
| MAP7D1 | Osteoporosis, Postmenopausal | 2.94 |
| MARCHF5 | Osteoporosis, Postmenopausal | 2.94 |
| MARK4 | Osteoporosis, Postmenopausal | 2.94 |
| MILR1 | Osteoporosis, Postmenopausal | 2.94 |
| MRPL22 | Osteoporosis, Postmenopausal | 2.94 |
| MYL4 | Osteoporosis, Postmenopausal | 2.94 |
| NACC2 | Osteoporosis, Postmenopausal | 2.94 |
| NFE2L2A | Osteoporosis, Postmenopausal | 2.94 |
| NGP | Osteoporosis, Postmenopausal | 2.94 |
| NGRN | Osteoporosis, Postmenopausal | 2.94 |
| ODAD3 | Osteoporosis, Postmenopausal | 2.94 |
| PIKFYVE | Osteoporosis, Postmenopausal | 2.94 |
| PIN4 | Osteoporosis, Postmenopausal | 2.94 |
| POMGNT2 | Osteoporosis, Postmenopausal | 2.94 |
| PPIL6 | Osteoporosis, Postmenopausal | 2.94 |
| PRAME | Osteoporosis, Postmenopausal | 2.94 |
| PROK1 | Osteoporosis, Postmenopausal | 2.94 |
| PRPF18 | Osteoporosis, Postmenopausal | 2.94 |
| PRPF4B | Osteoporosis, Postmenopausal | 2.94 |
| PRRT3 | Osteoporosis, Postmenopausal | 2.94 |
| RAB2B | Osteoporosis, Postmenopausal | 2.94 |
| RAD51B | Osteoporosis, Postmenopausal | 2.94 |
| RASGRP4 | Osteoporosis, Postmenopausal | 2.94 |
| RBBP5 | Osteoporosis, Postmenopausal | 2.94 |
| RERE | Osteoporosis, Postmenopausal | 2.94 |
| RHD | Osteoporosis, Postmenopausal | 2.94 |
| RPS6KL1 | Osteoporosis, Postmenopausal | 2.94 |
| SEC11A | Osteoporosis, Postmenopausal | 2.94 |
| SIKE1 | Osteoporosis, Postmenopausal | 2.94 |
| SIMC1 | Osteoporosis, Postmenopausal | 2.94 |
| SMARCAL1 | Osteoporosis, Postmenopausal | 2.94 |
| SPNS3 | Osteoporosis, Postmenopausal | 2.94 |
| SPOP | Osteoporosis, Postmenopausal | 2.94 |
| STAG2 | Osteoporosis, Postmenopausal | 2.94 |
| SUB1 | Osteoporosis, Postmenopausal | 2.94 |
| SVBP | Osteoporosis, Postmenopausal | 2.94 |
| SYT14 | Osteoporosis, Postmenopausal | 2.94 |
| THOC3 | Osteoporosis, Postmenopausal | 2.94 |
| TMEM70 | Osteoporosis, Postmenopausal | 2.94 |
| TMEM9 | Osteoporosis, Postmenopausal | 2.94 |
| TRAF3IP1 | Osteoporosis, Postmenopausal | 2.94 |
| TRIM9 | Osteoporosis, Postmenopausal | 2.94 |
| UGT1AB | Osteoporosis, Postmenopausal | 2.94 |
| XKR6 | Osteoporosis, Postmenopausal | 2.94 |
| YBEY | Osteoporosis, Postmenopausal | 2.94 |
| ZDHHC11 | Osteoporosis, Postmenopausal | 2.94 |
| ZFP422 | Osteoporosis, Postmenopausal | 2.94 |
| ZNF750 | Osteoporosis, Postmenopausal | 2.94 |
| ZSCAN20 | Osteoporosis, Postmenopausal | 2.94 |
| AJAP1 | Osteoporosis, Postmenopausal | 2.93 |
| CCDC28B | Osteoporosis, Postmenopausal | 2.93 |
| CLIP3 | Osteoporosis, Postmenopausal | 2.93 |
| CNTLN | Osteoporosis, Postmenopausal | 2.93 |
| CTBP2 | Osteoporosis, Postmenopausal | 2.93 |
| DCC | Osteoporosis, Postmenopausal | 2.93 |
| DIRAS2 | Osteoporosis, Postmenopausal | 2.93 |
| ETV6 | Osteoporosis, Postmenopausal | 2.93 |
| GNG7 | Osteoporosis, Postmenopausal | 2.93 |
| ITFG2 | Osteoporosis, Postmenopausal | 2.93 |
| LDHD | Osteoporosis, Postmenopausal | 2.93 |
| MAPKAP1 | Osteoporosis, Postmenopausal | 2.93 |
| MFSD1 | Osteoporosis, Postmenopausal | 2.93 |
| OSTM1 | Osteoporosis, Postmenopausal | 2.93 |
| PAGR1 | Osteoporosis, Postmenopausal | 2.93 |
| PPP2R1B | Osteoporosis, Postmenopausal | 2.93 |
| RAB24 | Osteoporosis, Postmenopausal | 2.93 |
| RAD1 | Osteoporosis, Postmenopausal | 2.93 |
| SCN2A | Osteoporosis, Postmenopausal | 2.93 |
| STARD3NL | Osteoporosis, Postmenopausal | 2.93 |
| TOLLIP | Osteoporosis, Postmenopausal | 2.93 |
| UBE2V1 | Osteoporosis, Postmenopausal | 2.93 |
| UCHL3 | Osteoporosis, Postmenopausal | 2.93 |
| WBP11 | Osteoporosis, Postmenopausal | 2.93 |
| WBP4 | Osteoporosis, Postmenopausal | 2.93 |
| WDR19 | Osteoporosis, Postmenopausal | 2.93 |
| ADD1 | Osteoporosis, Postmenopausal | 2.92 |
| AJUBA | Osteoporosis, Postmenopausal | 2.92 |
| ATAD1 | Osteoporosis, Postmenopausal | 2.92 |
| ATP6V1F | Osteoporosis, Postmenopausal | 2.92 |
| BORCS8 | Osteoporosis, Postmenopausal | 2.92 |
| C1QTNF5 | Osteoporosis, Postmenopausal | 2.92 |
| C8B | Osteoporosis, Postmenopausal | 2.92 |
| CCDC186 | Osteoporosis, Postmenopausal | 2.92 |
| CCDC61 | Osteoporosis, Postmenopausal | 2.92 |
| CCDC90B | Osteoporosis, Postmenopausal | 2.92 |
| CGGBP1 | Osteoporosis, Postmenopausal | 2.92 |
| COLEC10 | Osteoporosis, Postmenopausal | 2.92 |
| CUL5 | Osteoporosis, Postmenopausal | 2.92 |
| DCP2 | Osteoporosis, Postmenopausal | 2.92 |
| DRC1 | Osteoporosis, Postmenopausal | 2.92 |
| EAPP | Osteoporosis, Postmenopausal | 2.92 |
| EMC8 | Osteoporosis, Postmenopausal | 2.92 |
| EPB41 | Osteoporosis, Postmenopausal | 2.92 |
| FABP12 | Osteoporosis, Postmenopausal | 2.92 |
| FGF3 | Osteoporosis, Postmenopausal | 2.92 |
| FOXO6 | Osteoporosis, Postmenopausal | 2.92 |
| FOXRED2 | Osteoporosis, Postmenopausal | 2.92 |
| GDI1 | Osteoporosis, Postmenopausal | 2.92 |
| GPR88 | Osteoporosis, Postmenopausal | 2.92 |
| GRAMD2A | Osteoporosis, Postmenopausal | 2.92 |
| ICMT | Osteoporosis, Postmenopausal | 2.92 |
| ITLN1 | Osteoporosis, Postmenopausal | 2.92 |
| JMJD4 | Osteoporosis, Postmenopausal | 2.92 |
| KCNV1 | Osteoporosis, Postmenopausal | 2.92 |
| KIAA1549 | Osteoporosis, Postmenopausal | 2.92 |
| KLHL11 | Osteoporosis, Postmenopausal | 2.92 |
| LMBRD2 | Osteoporosis, Postmenopausal | 2.92 |
| LRRC73 | Osteoporosis, Postmenopausal | 2.92 |
| MAGI2 | Osteoporosis, Postmenopausal | 2.92 |
| MIR196B | Osteoporosis, Postmenopausal | 2.92 |
| MIR26B | Osteoporosis, Postmenopausal | 2.92 |
| MPHOSPH10 | Osteoporosis, Postmenopausal | 2.92 |
| NFU1 | Osteoporosis, Postmenopausal | 2.92 |
| OAS1B | Osteoporosis, Postmenopausal | 2.92 |
| PCDHB14 | Osteoporosis, Postmenopausal | 2.92 |
| PCSK1N | Osteoporosis, Postmenopausal | 2.92 |
| PGAP3 | Osteoporosis, Postmenopausal | 2.92 |
| PLA2G4B | Osteoporosis, Postmenopausal | 2.92 |
| PM20D1 | Osteoporosis, Postmenopausal | 2.92 |
| PSKH1 | Osteoporosis, Postmenopausal | 2.92 |
| PWWP2A | Osteoporosis, Postmenopausal | 2.92 |
| QTRT1 | Osteoporosis, Postmenopausal | 2.92 |
| RABGAP1 | Osteoporosis, Postmenopausal | 2.92 |
| RABGGTB | Osteoporosis, Postmenopausal | 2.92 |
| RAVER1 | Osteoporosis, Postmenopausal | 2.92 |
| RHOX5 | Osteoporosis, Postmenopausal | 2.92 |
| RUNDC3A | Osteoporosis, Postmenopausal | 2.92 |
| SCAF1 | Osteoporosis, Postmenopausal | 2.92 |
| SELENON | Osteoporosis, Postmenopausal | 2.92 |
| SLC25A48 | Osteoporosis, Postmenopausal | 2.92 |
| SPRN | Osteoporosis, Postmenopausal | 2.92 |
| SSBP1 | Osteoporosis, Postmenopausal | 2.92 |
| SUMF2 | Osteoporosis, Postmenopausal | 2.92 |
| SYT6 | Osteoporosis, Postmenopausal | 2.92 |
| TBC1D10A | Osteoporosis, Postmenopausal | 2.92 |
| THAP11 | Osteoporosis, Postmenopausal | 2.92 |
| THUMPD3 | Osteoporosis, Postmenopausal | 2.92 |
| TNIP3 | Osteoporosis, Postmenopausal | 2.92 |
| TRAPPC3 | Osteoporosis, Postmenopausal | 2.92 |
| TTC32 | Osteoporosis, Postmenopausal | 2.92 |
| UBTD2 | Osteoporosis, Postmenopausal | 2.92 |
| UGT3A1 | Osteoporosis, Postmenopausal | 2.92 |
| USP48 | Osteoporosis, Postmenopausal | 2.92 |
| UTP11 | Osteoporosis, Postmenopausal | 2.92 |
| VPS9D1 | Osteoporosis, Postmenopausal | 2.92 |
| VSX2 | Osteoporosis, Postmenopausal | 2.92 |
| WNT16 | Osteoporosis, Postmenopausal | 2.92 |
| ZDHHC18 | Osteoporosis, Postmenopausal | 2.92 |
| ZFP395 | Osteoporosis, Postmenopausal | 2.92 |
| ZNF521 | Osteoporosis, Postmenopausal | 2.92 |
| ABHD17C | Osteoporosis, Postmenopausal | 2.91 |
| ACAA1 | Osteoporosis, Postmenopausal | 2.91 |
| ACOT5 | Osteoporosis, Postmenopausal | 2.91 |
| ACP6 | Osteoporosis, Postmenopausal | 2.91 |
| ANAPC15 | Osteoporosis, Postmenopausal | 2.91 |
| ATG4D | Osteoporosis, Postmenopausal | 2.91 |
| BICDL2 | Osteoporosis, Postmenopausal | 2.91 |
| BRSK2 | Osteoporosis, Postmenopausal | 2.91 |
| CEP95 | Osteoporosis, Postmenopausal | 2.91 |
| CH25H | Osteoporosis, Postmenopausal | 2.91 |
| CMC1 | Osteoporosis, Postmenopausal | 2.91 |
| CPAMD8 | Osteoporosis, Postmenopausal | 2.91 |
| CSPG5 | Osteoporosis, Postmenopausal | 2.91 |
| CSTF1 | Osteoporosis, Postmenopausal | 2.91 |
| DAPK3 | Osteoporosis, Postmenopausal | 2.91 |
| DNAAF10 | Osteoporosis, Postmenopausal | 2.91 |
| DNAAF11 | Osteoporosis, Postmenopausal | 2.91 |
| DPP8 | Osteoporosis, Postmenopausal | 2.91 |
| EVC2 | Osteoporosis, Postmenopausal | 2.91 |
| FADS6 | Osteoporosis, Postmenopausal | 2.91 |
| FAM133B | Osteoporosis, Postmenopausal | 2.91 |
| GALNT3 | Osteoporosis, Postmenopausal | 2.91 |
| GBA3 | Osteoporosis, Postmenopausal | 2.91 |
| GNRH2 | Osteoporosis, Postmenopausal | 2.91 |
| H2-Q10 | Osteoporosis, Postmenopausal | 2.91 |
| JAKMIP2 | Osteoporosis, Postmenopausal | 2.91 |
| KLHL31 | Osteoporosis, Postmenopausal | 2.91 |
| KNG2 | Osteoporosis, Postmenopausal | 2.91 |
| KRBA1 | Osteoporosis, Postmenopausal | 2.91 |
| LIN28A | Osteoporosis, Postmenopausal | 2.91 |
| LYPD6 | Osteoporosis, Postmenopausal | 2.91 |
| MIR429 | Osteoporosis, Postmenopausal | 2.91 |
| MRPL21 | Osteoporosis, Postmenopausal | 2.91 |
| MRPS16 | Osteoporosis, Postmenopausal | 2.91 |
| MSH4 | Osteoporosis, Postmenopausal | 2.91 |
| MTNR1A | Osteoporosis, Postmenopausal | 2.91 |
| MTPAP | Osteoporosis, Postmenopausal | 2.91 |
| NLGN2 | Osteoporosis, Postmenopausal | 2.91 |
| OPA3 | Osteoporosis, Postmenopausal | 2.91 |
| PLET1 | Osteoporosis, Postmenopausal | 2.91 |
| PPP2R5C | Osteoporosis, Postmenopausal | 2.91 |
| PSMD11 | Osteoporosis, Postmenopausal | 2.91 |
| PTTG1IP | Osteoporosis, Postmenopausal | 2.91 |
| RASL10A | Osteoporosis, Postmenopausal | 2.91 |
| RSBN1 | Osteoporosis, Postmenopausal | 2.91 |
| SARNP | Osteoporosis, Postmenopausal | 2.91 |
| SIT1 | Osteoporosis, Postmenopausal | 2.91 |
| SLC17A4 | Osteoporosis, Postmenopausal | 2.91 |
| SLC27A5 | Osteoporosis, Postmenopausal | 2.91 |
| SLC28A1 | Osteoporosis, Postmenopausal | 2.91 |
| SLC5A2 | Osteoporosis, Postmenopausal | 2.91 |
| SMNDC1 | Osteoporosis, Postmenopausal | 2.91 |
| SPRED3 | Osteoporosis, Postmenopausal | 2.91 |
| TARS3 | Osteoporosis, Postmenopausal | 2.91 |
| TLE5 | Osteoporosis, Postmenopausal | 2.91 |
| TRIM33 | Osteoporosis, Postmenopausal | 2.91 |
| WRAP73 | Osteoporosis, Postmenopausal | 2.91 |
| YIF1A | Osteoporosis, Postmenopausal | 2.91 |
| ZFP385B | Osteoporosis, Postmenopausal | 2.91 |
| ZNF281 | Osteoporosis, Postmenopausal | 2.91 |
| ZNF430 | Osteoporosis, Postmenopausal | 2.91 |
| ZP2 | Osteoporosis, Postmenopausal | 2.91 |
| ADAM1A | Osteoporosis, Postmenopausal | 2.9 |
| ADAMTS19 | Osteoporosis, Postmenopausal | 2.9 |
| ANGPTL6 | Osteoporosis, Postmenopausal | 2.9 |
| BIN2 | Osteoporosis, Postmenopausal | 2.9 |
| BLTP3B | Osteoporosis, Postmenopausal | 2.9 |
| CCDC117 | Osteoporosis, Postmenopausal | 2.9 |
| CDC20B | Osteoporosis, Postmenopausal | 2.9 |
| CERKL | Osteoporosis, Postmenopausal | 2.9 |
| CHRNA6 | Osteoporosis, Postmenopausal | 2.9 |
| CLEC12A | Osteoporosis, Postmenopausal | 2.9 |
| CLEC4F | Osteoporosis, Postmenopausal | 2.9 |
| CLXN | Osteoporosis, Postmenopausal | 2.9 |
| CYB5D1 | Osteoporosis, Postmenopausal | 2.9 |
| CYP2B3 | Osteoporosis, Postmenopausal | 2.9 |
| DGKQ | Osteoporosis, Postmenopausal | 2.9 |
| DOC2A | Osteoporosis, Postmenopausal | 2.9 |
| EED | Osteoporosis, Postmenopausal | 2.9 |
| ENKD1 | Osteoporosis, Postmenopausal | 2.9 |
| FAM118B | Osteoporosis, Postmenopausal | 2.9 |
| FEM1A | Osteoporosis, Postmenopausal | 2.9 |
| GALNTL6 | Osteoporosis, Postmenopausal | 2.9 |
| GATA5 | Osteoporosis, Postmenopausal | 2.9 |
| H2AC15 | Osteoporosis, Postmenopausal | 2.9 |
| HCRTR2 | Osteoporosis, Postmenopausal | 2.9 |
| HELQ | Osteoporosis, Postmenopausal | 2.9 |
| HIKESHI | Osteoporosis, Postmenopausal | 2.9 |
| HPS3 | Osteoporosis, Postmenopausal | 2.9 |
| HSBP1 | Osteoporosis, Postmenopausal | 2.9 |
| IGLON5 | Osteoporosis, Postmenopausal | 2.9 |
| IL18BP | Osteoporosis, Postmenopausal | 2.9 |
| IRS3 | Osteoporosis, Postmenopausal | 2.9 |
| LEO1 | Osteoporosis, Postmenopausal | 2.9 |
| LIN7C | Osteoporosis, Postmenopausal | 2.9 |
| MCEE | Osteoporosis, Postmenopausal | 2.9 |
| MIR19A | Osteoporosis, Postmenopausal | 2.9 |
| MON1A | Osteoporosis, Postmenopausal | 2.9 |
| MRPL15 | Osteoporosis, Postmenopausal | 2.9 |
| NCBP2 | Osteoporosis, Postmenopausal | 2.9 |
| NCR3LG1 | Osteoporosis, Postmenopausal | 2.9 |
| NFYC | Osteoporosis, Postmenopausal | 2.9 |
| NUDT16L1 | Osteoporosis, Postmenopausal | 2.9 |
| OGDHL | Osteoporosis, Postmenopausal | 2.9 |
| PBSN | Osteoporosis, Postmenopausal | 2.9 |
| PDE6A | Osteoporosis, Postmenopausal | 2.9 |
| PFDN4 | Osteoporosis, Postmenopausal | 2.9 |
| PIP4P2 | Osteoporosis, Postmenopausal | 2.9 |
| PPP2R5D | Osteoporosis, Postmenopausal | 2.9 |
| RBM28 | Osteoporosis, Postmenopausal | 2.9 |
| RMDN1 | Osteoporosis, Postmenopausal | 2.9 |
| RPS6KC1 | Osteoporosis, Postmenopausal | 2.9 |
| RSL1D1 | Osteoporosis, Postmenopausal | 2.9 |
| SACM1L | Osteoporosis, Postmenopausal | 2.9 |
| SEM1 | Osteoporosis, Postmenopausal | 2.9 |
| SENP2 | Osteoporosis, Postmenopausal | 2.9 |
| SH3BP5L | Osteoporosis, Postmenopausal | 2.9 |
| SLC35E3 | Osteoporosis, Postmenopausal | 2.9 |
| SLC36A2 | Osteoporosis, Postmenopausal | 2.9 |
| SNHG15 | Osteoporosis, Postmenopausal | 2.9 |
| SPACA6 | Osteoporosis, Postmenopausal | 2.9 |
| STK33 | Osteoporosis, Postmenopausal | 2.9 |
| SULT4A1 | Osteoporosis, Postmenopausal | 2.9 |
| TEKT1 | Osteoporosis, Postmenopausal | 2.9 |
| TMEM109 | Osteoporosis, Postmenopausal | 2.9 |
| TMEM150A | Osteoporosis, Postmenopausal | 2.9 |
| TMEM178A | Osteoporosis, Postmenopausal | 2.9 |
| TMEM184B | Osteoporosis, Postmenopausal | 2.9 |
| TOMM7 | Osteoporosis, Postmenopausal | 2.9 |
| TTC21A | Osteoporosis, Postmenopausal | 2.9 |
| TTLL11 | Osteoporosis, Postmenopausal | 2.9 |
| TUT4 | Osteoporosis, Postmenopausal | 2.9 |
| UBLCP1 | Osteoporosis, Postmenopausal | 2.9 |
| UBXN4 | Osteoporosis, Postmenopausal | 2.9 |
| ULK3 | Osteoporosis, Postmenopausal | 2.9 |
| USF2 | Osteoporosis, Postmenopausal | 2.9 |
| USP39 | Osteoporosis, Postmenopausal | 2.9 |
| UTS2 | Osteoporosis, Postmenopausal | 2.9 |
| VPS33B | Osteoporosis, Postmenopausal | 2.9 |
| WWP2 | Osteoporosis, Postmenopausal | 2.9 |
| YAF2 | Osteoporosis, Postmenopausal | 2.9 |
| ZC3H12D | Osteoporosis, Postmenopausal | 2.9 |
| ZCCHC18 | Osteoporosis, Postmenopausal | 2.9 |
| ZNF292 | Osteoporosis, Postmenopausal | 2.9 |
| ZNF598 | Osteoporosis, Postmenopausal | 2.9 |
| AFG3L2 | Osteoporosis, Postmenopausal | 2.89 |
| APTX | Osteoporosis, Postmenopausal | 2.89 |
| ARHGEF38 | Osteoporosis, Postmenopausal | 2.89 |
| BOD1L1 | Osteoporosis, Postmenopausal | 2.89 |
| CA5B | Osteoporosis, Postmenopausal | 2.89 |
| CABLES2 | Osteoporosis, Postmenopausal | 2.89 |
| CAR4 | Osteoporosis, Postmenopausal | 2.89 |
| CCDC115 | Osteoporosis, Postmenopausal | 2.89 |
| CDADC1 | Osteoporosis, Postmenopausal | 2.89 |
| CDC26 | Osteoporosis, Postmenopausal | 2.89 |
| CEP290 | Osteoporosis, Postmenopausal | 2.89 |
| CFHR1 | Osteoporosis, Postmenopausal | 2.89 |
| CLDN23 | Osteoporosis, Postmenopausal | 2.89 |
| CLHC1 | Osteoporosis, Postmenopausal | 2.89 |
| CMYA5 | Osteoporosis, Postmenopausal | 2.89 |
| COCH | Osteoporosis, Postmenopausal | 2.89 |
| COG4 | Osteoporosis, Postmenopausal | 2.89 |
| CPNE5 | Osteoporosis, Postmenopausal | 2.89 |
| DENND2C | Osteoporosis, Postmenopausal | 2.89 |
| DHX34 | Osteoporosis, Postmenopausal | 2.89 |
| DIRAS3 | Osteoporosis, Postmenopausal | 2.89 |
| EMC1 | Osteoporosis, Postmenopausal | 2.89 |
| ERMAP | Osteoporosis, Postmenopausal | 2.89 |
| FAAP100 | Osteoporosis, Postmenopausal | 2.89 |
| FBXO43 | Osteoporosis, Postmenopausal | 2.89 |
| FOXN2 | Osteoporosis, Postmenopausal | 2.89 |
| GAB3 | Osteoporosis, Postmenopausal | 2.89 |
| GP2 | Osteoporosis, Postmenopausal | 2.89 |
| GPBP1L1 | Osteoporosis, Postmenopausal | 2.89 |
| GPN2 | Osteoporosis, Postmenopausal | 2.89 |
| HARBI1 | Osteoporosis, Postmenopausal | 2.89 |
| HCP5 | Osteoporosis, Postmenopausal | 2.89 |
| HESX1 | Osteoporosis, Postmenopausal | 2.89 |
| IFTAP | Osteoporosis, Postmenopausal | 2.89 |
| ITSN1 | Osteoporosis, Postmenopausal | 2.89 |
| KCND1 | Osteoporosis, Postmenopausal | 2.89 |
| KIF27 | Osteoporosis, Postmenopausal | 2.89 |
| LONP2 | Osteoporosis, Postmenopausal | 2.89 |
| LRRC4B | Osteoporosis, Postmenopausal | 2.89 |
| LRRC75A | Osteoporosis, Postmenopausal | 2.89 |
| MARVELD3 | Osteoporosis, Postmenopausal | 2.89 |
| MFSD5 | Osteoporosis, Postmenopausal | 2.89 |
| MIR30A | Osteoporosis, Postmenopausal | 2.89 |
| MIS12 | Osteoporosis, Postmenopausal | 2.89 |
| MKKS | Osteoporosis, Postmenopausal | 2.89 |
| MPZL1 | Osteoporosis, Postmenopausal | 2.89 |
| MRPL24 | Osteoporosis, Postmenopausal | 2.89 |
| MRPL46 | Osteoporosis, Postmenopausal | 2.89 |
| NATD1 | Osteoporosis, Postmenopausal | 2.89 |
| NOP10 | Osteoporosis, Postmenopausal | 2.89 |
| NSF | Osteoporosis, Postmenopausal | 2.89 |
| OLFM4 | Osteoporosis, Postmenopausal | 2.89 |
| OLR59 | Osteoporosis, Postmenopausal | 2.89 |
| P2RY13 | Osteoporosis, Postmenopausal | 2.89 |
| PARD6G | Osteoporosis, Postmenopausal | 2.89 |
| PDXDC1 | Osteoporosis, Postmenopausal | 2.89 |
| PSD2 | Osteoporosis, Postmenopausal | 2.89 |
| PTPN2 | Osteoporosis, Postmenopausal | 2.89 |
| RAB5B | Osteoporosis, Postmenopausal | 2.89 |
| RHPN1 | Osteoporosis, Postmenopausal | 2.89 |
| SCAMP2 | Osteoporosis, Postmenopausal | 2.89 |
| SIM1 | Osteoporosis, Postmenopausal | 2.89 |
| SLC15A4 | Osteoporosis, Postmenopausal | 2.89 |
| SLF1 | Osteoporosis, Postmenopausal | 2.89 |
| SOWAHB | Osteoporosis, Postmenopausal | 2.89 |
| SPHKAP | Osteoporosis, Postmenopausal | 2.89 |
| STAG3 | Osteoporosis, Postmenopausal | 2.89 |
| SYNDIG1 | Osteoporosis, Postmenopausal | 2.89 |
| TAF1C | Osteoporosis, Postmenopausal | 2.89 |
| TBC1D19 | Osteoporosis, Postmenopausal | 2.89 |
| TBC1D2B | Osteoporosis, Postmenopausal | 2.89 |
| TMEM17 | Osteoporosis, Postmenopausal | 2.89 |
| TMEM237 | Osteoporosis, Postmenopausal | 2.89 |
| TMEM63C | Osteoporosis, Postmenopausal | 2.89 |
| TRIM55 | Osteoporosis, Postmenopausal | 2.89 |
| UNC93A | Osteoporosis, Postmenopausal | 2.89 |
| UNK | Osteoporosis, Postmenopausal | 2.89 |
| VPS33A | Osteoporosis, Postmenopausal | 2.89 |
| WHRN | Osteoporosis, Postmenopausal | 2.89 |
| XKRX | Osteoporosis, Postmenopausal | 2.89 |
| ZC2HC1A | Osteoporosis, Postmenopausal | 2.89 |
| AHDC1 | Osteoporosis, Postmenopausal | 2.88 |
| ALG1 | Osteoporosis, Postmenopausal | 2.88 |
| AMHR2 | Osteoporosis, Postmenopausal | 2.88 |
| AP2A2 | Osteoporosis, Postmenopausal | 2.88 |
| APC2 | Osteoporosis, Postmenopausal | 2.88 |
| ARX | Osteoporosis, Postmenopausal | 2.88 |
| ASB1 | Osteoporosis, Postmenopausal | 2.88 |
| BOLA2 | Osteoporosis, Postmenopausal | 2.88 |
| CDH16 | Osteoporosis, Postmenopausal | 2.88 |
| CHCHD7 | Osteoporosis, Postmenopausal | 2.88 |
| CHSY3 | Osteoporosis, Postmenopausal | 2.88 |
| CIR1 | Osteoporosis, Postmenopausal | 2.88 |
| COPS3 | Osteoporosis, Postmenopausal | 2.88 |
| CPT1C | Osteoporosis, Postmenopausal | 2.88 |
| CRTC2 | Osteoporosis, Postmenopausal | 2.88 |
| CYP2C22 | Osteoporosis, Postmenopausal | 2.88 |
| CYP2J4 | Osteoporosis, Postmenopausal | 2.88 |
| CYTOR | Osteoporosis, Postmenopausal | 2.88 |
| DAW1 | Osteoporosis, Postmenopausal | 2.88 |
| DNASE2 | Osteoporosis, Postmenopausal | 2.88 |
| DPM1 | Osteoporosis, Postmenopausal | 2.88 |
| DTWD2 | Osteoporosis, Postmenopausal | 2.88 |
| ELP2 | Osteoporosis, Postmenopausal | 2.88 |
| FBXO22 | Osteoporosis, Postmenopausal | 2.88 |
| FES | Osteoporosis, Postmenopausal | 2.88 |
| FUZ | Osteoporosis, Postmenopausal | 2.88 |
| FXYD4 | Osteoporosis, Postmenopausal | 2.88 |
| GBP7 | Osteoporosis, Postmenopausal | 2.88 |
| GGA1 | Osteoporosis, Postmenopausal | 2.88 |
| GTF2A1 | Osteoporosis, Postmenopausal | 2.88 |
| GZMK | Osteoporosis, Postmenopausal | 2.88 |
| HCRTR1 | Osteoporosis, Postmenopausal | 2.88 |
| IK | Osteoporosis, Postmenopausal | 2.88 |
| ILDR2 | Osteoporosis, Postmenopausal | 2.88 |
| ISCA2 | Osteoporosis, Postmenopausal | 2.88 |
| KCTD10 | Osteoporosis, Postmenopausal | 2.88 |
| LHFPL6 | Osteoporosis, Postmenopausal | 2.88 |
| LPAR5 | Osteoporosis, Postmenopausal | 2.88 |
| LPCAT4 | Osteoporosis, Postmenopausal | 2.88 |
| LRFN3 | Osteoporosis, Postmenopausal | 2.88 |
| LRRC47 | Osteoporosis, Postmenopausal | 2.88 |
| MCHR1 | Osteoporosis, Postmenopausal | 2.88 |
| MDH1B | Osteoporosis, Postmenopausal | 2.88 |
| MGAT5B | Osteoporosis, Postmenopausal | 2.88 |
| MRPS14 | Osteoporosis, Postmenopausal | 2.88 |
| NAF1 | Osteoporosis, Postmenopausal | 2.88 |
| NALF2 | Osteoporosis, Postmenopausal | 2.88 |
| NELFB | Osteoporosis, Postmenopausal | 2.88 |
| NTF4 | Osteoporosis, Postmenopausal | 2.88 |
| NUDT16 | Osteoporosis, Postmenopausal | 2.88 |
| PDCD1LG2 | Osteoporosis, Postmenopausal | 2.88 |
| PHF5A | Osteoporosis, Postmenopausal | 2.88 |
| PIGV | Osteoporosis, Postmenopausal | 2.88 |
| POLR3A | Osteoporosis, Postmenopausal | 2.88 |
| POP5 | Osteoporosis, Postmenopausal | 2.88 |
| PPP4R3B | Osteoporosis, Postmenopausal | 2.88 |
| PWWP3B | Osteoporosis, Postmenopausal | 2.88 |
| RASA2 | Osteoporosis, Postmenopausal | 2.88 |
| RCHY1 | Osteoporosis, Postmenopausal | 2.88 |
| REPS1 | Osteoporosis, Postmenopausal | 2.88 |
| RPS6KA6 | Osteoporosis, Postmenopausal | 2.88 |
| RUBCN | Osteoporosis, Postmenopausal | 2.88 |
| SERP2 | Osteoporosis, Postmenopausal | 2.88 |
| SIX2 | Osteoporosis, Postmenopausal | 2.88 |
| SLC28A3 | Osteoporosis, Postmenopausal | 2.88 |
| SLFN13 | Osteoporosis, Postmenopausal | 2.88 |
| SMIM5 | Osteoporosis, Postmenopausal | 2.88 |
| SRP19 | Osteoporosis, Postmenopausal | 2.88 |
| ST18 | Osteoporosis, Postmenopausal | 2.88 |
| TET1 | Osteoporosis, Postmenopausal | 2.88 |
| TLCD2 | Osteoporosis, Postmenopausal | 2.88 |
| TMEM184A | Osteoporosis, Postmenopausal | 2.88 |
| TOR1B | Osteoporosis, Postmenopausal | 2.88 |
| USP14 | Osteoporosis, Postmenopausal | 2.88 |
| UTP23 | Osteoporosis, Postmenopausal | 2.88 |
| VPS50 | Osteoporosis, Postmenopausal | 2.88 |
| ADGRE4 | Osteoporosis, Postmenopausal | 2.87 |
| ANKS1A | Osteoporosis, Postmenopausal | 2.87 |
| AQR | Osteoporosis, Postmenopausal | 2.87 |
| ARF5 | Osteoporosis, Postmenopausal | 2.87 |
| ARRDC2 | Osteoporosis, Postmenopausal | 2.87 |
| ART5 | Osteoporosis, Postmenopausal | 2.87 |
| ASB5 | Osteoporosis, Postmenopausal | 2.87 |
| CEP112 | Osteoporosis, Postmenopausal | 2.87 |
| CFAP53 | Osteoporosis, Postmenopausal | 2.87 |
| CHFR | Osteoporosis, Postmenopausal | 2.87 |
| CYP2C13 | Osteoporosis, Postmenopausal | 2.87 |
| DMTF1 | Osteoporosis, Postmenopausal | 2.87 |
| DNASE1L3 | Osteoporosis, Postmenopausal | 2.87 |
| EID1 | Osteoporosis, Postmenopausal | 2.87 |
| EIF2B5 | Osteoporosis, Postmenopausal | 2.87 |
| FAM181B | Osteoporosis, Postmenopausal | 2.87 |
| FCER2 | Osteoporosis, Postmenopausal | 2.87 |
| GANC | Osteoporosis, Postmenopausal | 2.87 |
| GCNT4 | Osteoporosis, Postmenopausal | 2.87 |
| GFOD2 | Osteoporosis, Postmenopausal | 2.87 |
| GTF2E1 | Osteoporosis, Postmenopausal | 2.87 |
| H3C8 | Osteoporosis, Postmenopausal | 2.87 |
| HINT2 | Osteoporosis, Postmenopausal | 2.87 |
| IFT56 | Osteoporosis, Postmenopausal | 2.87 |
| IFT57 | Osteoporosis, Postmenopausal | 2.87 |
| IL17D | Osteoporosis, Postmenopausal | 2.87 |
| INTS1 | Osteoporosis, Postmenopausal | 2.87 |
| JTB | Osteoporosis, Postmenopausal | 2.87 |
| MC2R | Osteoporosis, Postmenopausal | 2.87 |
| MORC3 | Osteoporosis, Postmenopausal | 2.87 |
| MRPL3 | Osteoporosis, Postmenopausal | 2.87 |
| MSANTD4 | Osteoporosis, Postmenopausal | 2.87 |
| NBEAL1 | Osteoporosis, Postmenopausal | 2.87 |
| NDUFS6 | Osteoporosis, Postmenopausal | 2.87 |
| NELFA | Osteoporosis, Postmenopausal | 2.87 |
| ODAD2 | Osteoporosis, Postmenopausal | 2.87 |
| ORMDL3 | Osteoporosis, Postmenopausal | 2.87 |
| OVGP1 | Osteoporosis, Postmenopausal | 2.87 |
| PECR | Osteoporosis, Postmenopausal | 2.87 |
| PGGT1B | Osteoporosis, Postmenopausal | 2.87 |
| POF1B | Osteoporosis, Postmenopausal | 2.87 |
| PRMT5 | Osteoporosis, Postmenopausal | 2.87 |
| PTK6 | Osteoporosis, Postmenopausal | 2.87 |
| RAB7A | Osteoporosis, Postmenopausal | 2.87 |
| RAP2A | Osteoporosis, Postmenopausal | 2.87 |
| RBBP9 | Osteoporosis, Postmenopausal | 2.87 |
| RNF122 | Osteoporosis, Postmenopausal | 2.87 |
| RPL7L1 | Osteoporosis, Postmenopausal | 2.87 |
| RT1-EC2 | Osteoporosis, Postmenopausal | 2.87 |
| RTCB | Osteoporosis, Postmenopausal | 2.87 |
| SLC35C1 | Osteoporosis, Postmenopausal | 2.87 |
| SNHG6 | Osteoporosis, Postmenopausal | 2.87 |
| SYPL2 | Osteoporosis, Postmenopausal | 2.87 |
| TLCD5 | Osteoporosis, Postmenopausal | 2.87 |
| TMEM203 | Osteoporosis, Postmenopausal | 2.87 |
| TMEM62 | Osteoporosis, Postmenopausal | 2.87 |
| TSPAN32 | Osteoporosis, Postmenopausal | 2.87 |
| TTLL6 | Osteoporosis, Postmenopausal | 2.87 |
| USP16 | Osteoporosis, Postmenopausal | 2.87 |
| USP38 | Osteoporosis, Postmenopausal | 2.87 |
| WASHC2C | Osteoporosis, Postmenopausal | 2.87 |
| WFIKKN1 | Osteoporosis, Postmenopausal | 2.87 |
| YTHDF3 | Osteoporosis, Postmenopausal | 2.87 |
| ZBTB42 | Osteoporosis, Postmenopausal | 2.87 |
| ZBTB45 | Osteoporosis, Postmenopausal | 2.87 |
| ZFP14 | Osteoporosis, Postmenopausal | 2.87 |
| ZFP57 | Osteoporosis, Postmenopausal | 2.87 |
| ZNF267 | Osteoporosis, Postmenopausal | 2.87 |
| ATG2B | Osteoporosis, Postmenopausal | 2.86 |
| ATP2C2 | Osteoporosis, Postmenopausal | 2.86 |
| CACFD1 | Osteoporosis, Postmenopausal | 2.86 |
| CAMKV | Osteoporosis, Postmenopausal | 2.86 |
| CASS4 | Osteoporosis, Postmenopausal | 2.86 |
| CFAP52 | Osteoporosis, Postmenopausal | 2.86 |
| CLEC4A1 | Osteoporosis, Postmenopausal | 2.86 |
| CLUAP1 | Osteoporosis, Postmenopausal | 2.86 |
| COMMD6 | Osteoporosis, Postmenopausal | 2.86 |
| CRTC3 | Osteoporosis, Postmenopausal | 2.86 |
| CST7 | Osteoporosis, Postmenopausal | 2.86 |
| CX3CR1 | Osteoporosis, Postmenopausal | 2.86 |
| DIP2B | Osteoporosis, Postmenopausal | 2.86 |
| FAM83A | Osteoporosis, Postmenopausal | 2.86 |
| FER | Osteoporosis, Postmenopausal | 2.86 |
| GALK2 | Osteoporosis, Postmenopausal | 2.86 |
| GLB1L2 | Osteoporosis, Postmenopausal | 2.86 |
| GTPBP6 | Osteoporosis, Postmenopausal | 2.86 |
| IFITM6 | Osteoporosis, Postmenopausal | 2.86 |
| INSL6 | Osteoporosis, Postmenopausal | 2.86 |
| KANSL1L | Osteoporosis, Postmenopausal | 2.86 |
| KCNJ4 | Osteoporosis, Postmenopausal | 2.86 |
| KIF12 | Osteoporosis, Postmenopausal | 2.86 |
| KMT5C | Osteoporosis, Postmenopausal | 2.86 |
| LRRC27 | Osteoporosis, Postmenopausal | 2.86 |
| LRRC56 | Osteoporosis, Postmenopausal | 2.86 |
| MBTPS2 | Osteoporosis, Postmenopausal | 2.86 |
| MIR210 | Osteoporosis, Postmenopausal | 2.86 |
| MOB2 | Osteoporosis, Postmenopausal | 2.86 |
| MRPS7 | Osteoporosis, Postmenopausal | 2.86 |
| MXI1 | Osteoporosis, Postmenopausal | 2.86 |
| NFAM1 | Osteoporosis, Postmenopausal | 2.86 |
| NIPSNAP3B | Osteoporosis, Postmenopausal | 2.86 |
| NKX2-1 | Osteoporosis, Postmenopausal | 2.86 |
| NUBP2 | Osteoporosis, Postmenopausal | 2.86 |
| PDE6D | Osteoporosis, Postmenopausal | 2.86 |
| POLB | Osteoporosis, Postmenopausal | 2.86 |
| PRDM4 | Osteoporosis, Postmenopausal | 2.86 |
| PRR14L | Osteoporosis, Postmenopausal | 2.86 |
| PYGO1 | Osteoporosis, Postmenopausal | 2.86 |
| RAB21 | Osteoporosis, Postmenopausal | 2.86 |
| RINL | Osteoporosis, Postmenopausal | 2.86 |
| SAMD12 | Osteoporosis, Postmenopausal | 2.86 |
| SLC36A4 | Osteoporosis, Postmenopausal | 2.86 |
| SOX1 | Osteoporosis, Postmenopausal | 2.86 |
| SPINK4 | Osteoporosis, Postmenopausal | 2.86 |
| SUGP2 | Osteoporosis, Postmenopausal | 2.86 |
| SWI5 | Osteoporosis, Postmenopausal | 2.86 |
| TAF1 | Osteoporosis, Postmenopausal | 2.86 |
| TAF6L | Osteoporosis, Postmenopausal | 2.86 |
| TLE6 | Osteoporosis, Postmenopausal | 2.86 |
| TMX2 | Osteoporosis, Postmenopausal | 2.86 |
| TRO | Osteoporosis, Postmenopausal | 2.86 |
| U2AF2 | Osteoporosis, Postmenopausal | 2.86 |
| VAMP7 | Osteoporosis, Postmenopausal | 2.86 |
| VTI1A | Osteoporosis, Postmenopausal | 2.86 |
| WBP1 | Osteoporosis, Postmenopausal | 2.86 |
| WDR11 | Osteoporosis, Postmenopausal | 2.86 |
| WDR13 | Osteoporosis, Postmenopausal | 2.86 |
| WDR89 | Osteoporosis, Postmenopausal | 2.86 |
| WSCD1 | Osteoporosis, Postmenopausal | 2.86 |
| ZNF593 | Osteoporosis, Postmenopausal | 2.86 |
| AATF | Osteoporosis, Postmenopausal | 2.85 |
| ABCC12 | Osteoporosis, Postmenopausal | 2.85 |
| ABHD15 | Osteoporosis, Postmenopausal | 2.85 |
| ACAA1A | Osteoporosis, Postmenopausal | 2.85 |
| ADAMTS8 | Osteoporosis, Postmenopausal | 2.85 |
| AFTPH | Osteoporosis, Postmenopausal | 2.85 |
| APBB1 | Osteoporosis, Postmenopausal | 2.85 |
| ARFGAP2 | Osteoporosis, Postmenopausal | 2.85 |
| ARL11 | Osteoporosis, Postmenopausal | 2.85 |
| ASXL3 | Osteoporosis, Postmenopausal | 2.85 |
| C1QTNF7 | Osteoporosis, Postmenopausal | 2.85 |
| CCM2L | Osteoporosis, Postmenopausal | 2.85 |
| CDC42EP5 | Osteoporosis, Postmenopausal | 2.85 |
| CHCHD2 | Osteoporosis, Postmenopausal | 2.85 |
| CIBAR1 | Osteoporosis, Postmenopausal | 2.85 |
| CLEC4A | Osteoporosis, Postmenopausal | 2.85 |
| CYB5D2 | Osteoporosis, Postmenopausal | 2.85 |
| DCTN1 | Osteoporosis, Postmenopausal | 2.85 |
| DENND4B | Osteoporosis, Postmenopausal | 2.85 |
| DNAI3 | Osteoporosis, Postmenopausal | 2.85 |
| DXO | Osteoporosis, Postmenopausal | 2.85 |
| EMD | Osteoporosis, Postmenopausal | 2.85 |
| ESCO1 | Osteoporosis, Postmenopausal | 2.85 |
| FHIT | Osteoporosis, Postmenopausal | 2.85 |
| FIBP | Osteoporosis, Postmenopausal | 2.85 |
| FRMD8 | Osteoporosis, Postmenopausal | 2.85 |
| GXYLT1 | Osteoporosis, Postmenopausal | 2.85 |
| HBS1L | Osteoporosis, Postmenopausal | 2.85 |
| HTR1D | Osteoporosis, Postmenopausal | 2.85 |
| HYPK | Osteoporosis, Postmenopausal | 2.85 |
| IGKC | Osteoporosis, Postmenopausal | 2.85 |
| IKZF5 | Osteoporosis, Postmenopausal | 2.85 |
| IWS1 | Osteoporosis, Postmenopausal | 2.85 |
| KIRREL1 | Osteoporosis, Postmenopausal | 2.85 |
| KLHL15 | Osteoporosis, Postmenopausal | 2.85 |
| LMO1 | Osteoporosis, Postmenopausal | 2.85 |
| LNPEP | Osteoporosis, Postmenopausal | 2.85 |
| MPP3 | Osteoporosis, Postmenopausal | 2.85 |
| MRPS5 | Osteoporosis, Postmenopausal | 2.85 |
| NAPG | Osteoporosis, Postmenopausal | 2.85 |
| OASL1 | Osteoporosis, Postmenopausal | 2.85 |
| OCA2 | Osteoporosis, Postmenopausal | 2.85 |
| OTOF | Osteoporosis, Postmenopausal | 2.85 |
| PAK4 | Osteoporosis, Postmenopausal | 2.85 |
| PAXBP1 | Osteoporosis, Postmenopausal | 2.85 |
| PCGF3 | Osteoporosis, Postmenopausal | 2.85 |
| PERM1 | Osteoporosis, Postmenopausal | 2.85 |
| PGLYRP2 | Osteoporosis, Postmenopausal | 2.85 |
| PTDSS1 | Osteoporosis, Postmenopausal | 2.85 |
| RAD52 | Osteoporosis, Postmenopausal | 2.85 |
| RESP18 | Osteoporosis, Postmenopausal | 2.85 |
| RIOK2 | Osteoporosis, Postmenopausal | 2.85 |
| RNF6 | Osteoporosis, Postmenopausal | 2.85 |
| RT1-DB1 | Osteoporosis, Postmenopausal | 2.85 |
| SCO2 | Osteoporosis, Postmenopausal | 2.85 |
| SHF | Osteoporosis, Postmenopausal | 2.85 |
| SIL1 | Osteoporosis, Postmenopausal | 2.85 |
| SLC14A2 | Osteoporosis, Postmenopausal | 2.85 |
| SLC23A3 | Osteoporosis, Postmenopausal | 2.85 |
| SNX11 | Osteoporosis, Postmenopausal | 2.85 |
| SPCS1 | Osteoporosis, Postmenopausal | 2.85 |
| STOX1 | Osteoporosis, Postmenopausal | 2.85 |
| TBX1 | Osteoporosis, Postmenopausal | 2.85 |
| TIMM23 | Osteoporosis, Postmenopausal | 2.85 |
| TMED4 | Osteoporosis, Postmenopausal | 2.85 |
| TMEM129 | Osteoporosis, Postmenopausal | 2.85 |
| TMEM144 | Osteoporosis, Postmenopausal | 2.85 |
| TMEM59L | Osteoporosis, Postmenopausal | 2.85 |
| TRIM3 | Osteoporosis, Postmenopausal | 2.85 |
| TUT7 | Osteoporosis, Postmenopausal | 2.85 |
| UAP1L1 | Osteoporosis, Postmenopausal | 2.85 |
| UGGT2 | Osteoporosis, Postmenopausal | 2.85 |
| VMA21 | Osteoporosis, Postmenopausal | 2.85 |
| ZFC3H1 | Osteoporosis, Postmenopausal | 2.85 |
| ZRANB3 | Osteoporosis, Postmenopausal | 2.85 |
| AAMP | Osteoporosis, Postmenopausal | 2.84 |
| AMZ1 | Osteoporosis, Postmenopausal | 2.84 |
| BLCAP | Osteoporosis, Postmenopausal | 2.84 |
| CALY | Osteoporosis, Postmenopausal | 2.84 |
| CCDC146 | Osteoporosis, Postmenopausal | 2.84 |
| CD177 | Osteoporosis, Postmenopausal | 2.84 |
| CDH23 | Osteoporosis, Postmenopausal | 2.84 |
| CHST10 | Osteoporosis, Postmenopausal | 2.84 |
| CORO1B | Osteoporosis, Postmenopausal | 2.84 |
| CPSF4 | Osteoporosis, Postmenopausal | 2.84 |
| CYP4V2 | Osteoporosis, Postmenopausal | 2.84 |
| DAAM2 | Osteoporosis, Postmenopausal | 2.84 |
| DDX51 | Osteoporosis, Postmenopausal | 2.84 |
| DIMT1 | Osteoporosis, Postmenopausal | 2.84 |
| EPHA10 | Osteoporosis, Postmenopausal | 2.84 |
| FAM210A | Osteoporosis, Postmenopausal | 2.84 |
| FBXO16 | Osteoporosis, Postmenopausal | 2.84 |
| GAS8 | Osteoporosis, Postmenopausal | 2.84 |
| GATB | Osteoporosis, Postmenopausal | 2.84 |
| HYKK | Osteoporosis, Postmenopausal | 2.84 |
| IDNK | Osteoporosis, Postmenopausal | 2.84 |
| IFT122 | Osteoporosis, Postmenopausal | 2.84 |
| IQSEC2 | Osteoporosis, Postmenopausal | 2.84 |
| KIF6 | Osteoporosis, Postmenopausal | 2.84 |
| LRFN1 | Osteoporosis, Postmenopausal | 2.84 |
| MEIOB | Osteoporosis, Postmenopausal | 2.84 |
| MICU3 | Osteoporosis, Postmenopausal | 2.84 |
| MIR144 | Osteoporosis, Postmenopausal | 2.84 |
| MOAP1 | Osteoporosis, Postmenopausal | 2.84 |
| MRPS28 | Osteoporosis, Postmenopausal | 2.84 |
| MYOT | Osteoporosis, Postmenopausal | 2.84 |
| NPHP3 | Osteoporosis, Postmenopausal | 2.84 |
| PARD6A | Osteoporosis, Postmenopausal | 2.84 |
| PGAP6 | Osteoporosis, Postmenopausal | 2.84 |
| PIGS | Osteoporosis, Postmenopausal | 2.84 |
| POLL | Osteoporosis, Postmenopausal | 2.84 |
| POP7 | Osteoporosis, Postmenopausal | 2.84 |
| PRND | Osteoporosis, Postmenopausal | 2.84 |
| RAD51D | Osteoporosis, Postmenopausal | 2.84 |
| RHBDL1 | Osteoporosis, Postmenopausal | 2.84 |
| RNF186 | Osteoporosis, Postmenopausal | 2.84 |
| RPL15 | Osteoporosis, Postmenopausal | 2.84 |
| RPP21 | Osteoporosis, Postmenopausal | 2.84 |
| SCD5 | Osteoporosis, Postmenopausal | 2.84 |
| SGIP1 | Osteoporosis, Postmenopausal | 2.84 |
| SGSM3 | Osteoporosis, Postmenopausal | 2.84 |
| SH3BP1 | Osteoporosis, Postmenopausal | 2.84 |
| SNX4 | Osteoporosis, Postmenopausal | 2.84 |
| SPEF2 | Osteoporosis, Postmenopausal | 2.84 |
| STT3B | Osteoporosis, Postmenopausal | 2.84 |
| TAF10 | Osteoporosis, Postmenopausal | 2.84 |
| TIMM44 | Osteoporosis, Postmenopausal | 2.84 |
| TMEM104 | Osteoporosis, Postmenopausal | 2.84 |
| TOMM6 | Osteoporosis, Postmenopausal | 2.84 |
| TTC8 | Osteoporosis, Postmenopausal | 2.84 |
| UBE2D4 | Osteoporosis, Postmenopausal | 2.84 |
| VNN3 | Osteoporosis, Postmenopausal | 2.84 |
| ABHD1 | Osteoporosis, Postmenopausal | 2.83 |
| ADPRS | Osteoporosis, Postmenopausal | 2.83 |
| AIM2 | Osteoporosis, Postmenopausal | 2.83 |
| ANKRD13B | Osteoporosis, Postmenopausal | 2.83 |
| BAG6 | Osteoporosis, Postmenopausal | 2.83 |
| BCCIP | Osteoporosis, Postmenopausal | 2.83 |
| BRCC3 | Osteoporosis, Postmenopausal | 2.83 |
| C15ORF39 | Osteoporosis, Postmenopausal | 2.83 |
| CAPZA2 | Osteoporosis, Postmenopausal | 2.83 |
| CCDC167 | Osteoporosis, Postmenopausal | 2.83 |
| CCDC65 | Osteoporosis, Postmenopausal | 2.83 |
| CCL26 | Osteoporosis, Postmenopausal | 2.83 |
| CDC5L | Osteoporosis, Postmenopausal | 2.83 |
| CELF5 | Osteoporosis, Postmenopausal | 2.83 |
| CHCHD4 | Osteoporosis, Postmenopausal | 2.83 |
| CHIT1 | Osteoporosis, Postmenopausal | 2.83 |
| CHP1 | Osteoporosis, Postmenopausal | 2.83 |
| CLTA | Osteoporosis, Postmenopausal | 2.83 |
| CNIH1 | Osteoporosis, Postmenopausal | 2.83 |
| CNNM1 | Osteoporosis, Postmenopausal | 2.83 |
| CNPY4 | Osteoporosis, Postmenopausal | 2.83 |
| CYFIP1 | Osteoporosis, Postmenopausal | 2.83 |
| CYP4V3 | Osteoporosis, Postmenopausal | 2.83 |
| CYS1 | Osteoporosis, Postmenopausal | 2.83 |
| CYTH2 | Osteoporosis, Postmenopausal | 2.83 |
| DHX36 | Osteoporosis, Postmenopausal | 2.83 |
| DNAJB12 | Osteoporosis, Postmenopausal | 2.83 |
| DNALI1 | Osteoporosis, Postmenopausal | 2.83 |
| DYNC2H1 | Osteoporosis, Postmenopausal | 2.83 |
| DYNC2LI1 | Osteoporosis, Postmenopausal | 2.83 |
| EPC1 | Osteoporosis, Postmenopausal | 2.83 |
| EXT2 | Osteoporosis, Postmenopausal | 2.83 |
| FAM8A1 | Osteoporosis, Postmenopausal | 2.83 |
| FOXF1 | Osteoporosis, Postmenopausal | 2.83 |
| GIT2 | Osteoporosis, Postmenopausal | 2.83 |
| GPR162 | Osteoporosis, Postmenopausal | 2.83 |
| GPS2 | Osteoporosis, Postmenopausal | 2.83 |
| GRAP | Osteoporosis, Postmenopausal | 2.83 |
| GUCY1B2 | Osteoporosis, Postmenopausal | 2.83 |
| H3F3B | Osteoporosis, Postmenopausal | 2.83 |
| HOXB4 | Osteoporosis, Postmenopausal | 2.83 |
| HOXC10 | Osteoporosis, Postmenopausal | 2.83 |
| HOXD10 | Osteoporosis, Postmenopausal | 2.83 |
| INPP4A | Osteoporosis, Postmenopausal | 2.83 |
| KDM4C | Osteoporosis, Postmenopausal | 2.83 |
| KLHDC9 | Osteoporosis, Postmenopausal | 2.83 |
| LCA5 | Osteoporosis, Postmenopausal | 2.83 |
| LSG1 | Osteoporosis, Postmenopausal | 2.83 |
| MATN1 | Osteoporosis, Postmenopausal | 2.83 |
| MIR221 | Osteoporosis, Postmenopausal | 2.83 |
| MMP23 | Osteoporosis, Postmenopausal | 2.83 |
| MSTO1 | Osteoporosis, Postmenopausal | 2.83 |
| NCOA5 | Osteoporosis, Postmenopausal | 2.83 |
| NDUFA6 | Osteoporosis, Postmenopausal | 2.83 |
| NEURL1 | Osteoporosis, Postmenopausal | 2.83 |
| NIPAL3 | Osteoporosis, Postmenopausal | 2.83 |
| NKX2-5 | Osteoporosis, Postmenopausal | 2.83 |
| OVOL2 | Osteoporosis, Postmenopausal | 2.83 |
| P2RX2 | Osteoporosis, Postmenopausal | 2.83 |
| P2RX6 | Osteoporosis, Postmenopausal | 2.83 |
| PACRG | Osteoporosis, Postmenopausal | 2.83 |
| PARP10 | Osteoporosis, Postmenopausal | 2.83 |
| PARP11 | Osteoporosis, Postmenopausal | 2.83 |
| PATL1 | Osteoporosis, Postmenopausal | 2.83 |
| PHF6 | Osteoporosis, Postmenopausal | 2.83 |
| PLEKHN1 | Osteoporosis, Postmenopausal | 2.83 |
| PLIN1 | Osteoporosis, Postmenopausal | 2.83 |
| POMGNT1 | Osteoporosis, Postmenopausal | 2.83 |
| PPAP2A | Osteoporosis, Postmenopausal | 2.83 |
| PPP4R2 | Osteoporosis, Postmenopausal | 2.83 |
| RPL39L | Osteoporosis, Postmenopausal | 2.83 |
| RWDD3 | Osteoporosis, Postmenopausal | 2.83 |
| SBSN | Osteoporosis, Postmenopausal | 2.83 |
| SCCPDH | Osteoporosis, Postmenopausal | 2.83 |
| SIGLEC5 | Osteoporosis, Postmenopausal | 2.83 |
| SLC30A7 | Osteoporosis, Postmenopausal | 2.83 |
| SPINK5 | Osteoporosis, Postmenopausal | 2.83 |
| SSH3 | Osteoporosis, Postmenopausal | 2.83 |
| SYCP2 | Osteoporosis, Postmenopausal | 2.83 |
| SYNGAP1 | Osteoporosis, Postmenopausal | 2.83 |
| TGFBRAP1 | Osteoporosis, Postmenopausal | 2.83 |
| TMEM183A | Osteoporosis, Postmenopausal | 2.83 |
| TMEM185B | Osteoporosis, Postmenopausal | 2.83 |
| TMEM231 | Osteoporosis, Postmenopausal | 2.83 |
| TSPAN11 | Osteoporosis, Postmenopausal | 2.83 |
| VDAC3 | Osteoporosis, Postmenopausal | 2.83 |
| ZNF219 | Osteoporosis, Postmenopausal | 2.83 |
| ZSWIM4 | Osteoporosis, Postmenopausal | 2.83 |
| ADO | Osteoporosis, Postmenopausal | 2.82 |
| AKAP8 | Osteoporosis, Postmenopausal | 2.82 |
| ANAPC7 | Osteoporosis, Postmenopausal | 2.82 |
| ANKIB1 | Osteoporosis, Postmenopausal | 2.82 |
| AP3M1 | Osteoporosis, Postmenopausal | 2.82 |
| ARL10 | Osteoporosis, Postmenopausal | 2.82 |
| ARPIN | Osteoporosis, Postmenopausal | 2.82 |
| ATP4A | Osteoporosis, Postmenopausal | 2.82 |
| CA8 | Osteoporosis, Postmenopausal | 2.82 |
| CERCAM | Osteoporosis, Postmenopausal | 2.82 |
| CERT1 | Osteoporosis, Postmenopausal | 2.82 |
| CFAP43 | Osteoporosis, Postmenopausal | 2.82 |
| CYP4A8 | Osteoporosis, Postmenopausal | 2.82 |
| DIPK1A | Osteoporosis, Postmenopausal | 2.82 |
| DIRAS1 | Osteoporosis, Postmenopausal | 2.82 |
| DIS3 | Osteoporosis, Postmenopausal | 2.82 |
| DNAJC7 | Osteoporosis, Postmenopausal | 2.82 |
| G6PC3 | Osteoporosis, Postmenopausal | 2.82 |
| GRTP1 | Osteoporosis, Postmenopausal | 2.82 |
| IFT27 | Osteoporosis, Postmenopausal | 2.82 |
| INPP5K | Osteoporosis, Postmenopausal | 2.82 |
| JPH3 | Osteoporosis, Postmenopausal | 2.82 |
| KCNQ3 | Osteoporosis, Postmenopausal | 2.82 |
| KCTD11 | Osteoporosis, Postmenopausal | 2.82 |
| KCTD5 | Osteoporosis, Postmenopausal | 2.82 |
| KDM3A | Osteoporosis, Postmenopausal | 2.82 |
| LRRC8E | Osteoporosis, Postmenopausal | 2.82 |
| MAP9 | Osteoporosis, Postmenopausal | 2.82 |
| MBPA | Osteoporosis, Postmenopausal | 2.82 |
| MED18 | Osteoporosis, Postmenopausal | 2.82 |
| MIDEAS | Osteoporosis, Postmenopausal | 2.82 |
| MIEF1 | Osteoporosis, Postmenopausal | 2.82 |
| MPV17L2 | Osteoporosis, Postmenopausal | 2.82 |
| MRPL58 | Osteoporosis, Postmenopausal | 2.82 |
| NDUFS2 | Osteoporosis, Postmenopausal | 2.82 |
| NR6A1 | Osteoporosis, Postmenopausal | 2.82 |
| PIH1D1 | Osteoporosis, Postmenopausal | 2.82 |
| PLEKHH3 | Osteoporosis, Postmenopausal | 2.82 |
| POLR2M | Osteoporosis, Postmenopausal | 2.82 |
| PRKAG1 | Osteoporosis, Postmenopausal | 2.82 |
| PRPSAP1 | Osteoporosis, Postmenopausal | 2.82 |
| QRSL1 | Osteoporosis, Postmenopausal | 2.82 |
| RAB8A | Osteoporosis, Postmenopausal | 2.82 |
| RANBP2 | Osteoporosis, Postmenopausal | 2.82 |
| RGL3 | Osteoporosis, Postmenopausal | 2.82 |
| RHOT2 | Osteoporosis, Postmenopausal | 2.82 |
| RO60 | Osteoporosis, Postmenopausal | 2.82 |
| RPE65 | Osteoporosis, Postmenopausal | 2.82 |
| SEPTIN2 | Osteoporosis, Postmenopausal | 2.82 |
| SLC25A51 | Osteoporosis, Postmenopausal | 2.82 |
| SLC45A1 | Osteoporosis, Postmenopausal | 2.82 |
| SLITRK4 | Osteoporosis, Postmenopausal | 2.82 |
| SNRPD2 | Osteoporosis, Postmenopausal | 2.82 |
| SNX5 | Osteoporosis, Postmenopausal | 2.82 |
| SRP68 | Osteoporosis, Postmenopausal | 2.82 |
| SUCO | Osteoporosis, Postmenopausal | 2.82 |
| SUPT16H | Osteoporosis, Postmenopausal | 2.82 |
| TAF12 | Osteoporosis, Postmenopausal | 2.82 |
| TIMMDC1 | Osteoporosis, Postmenopausal | 2.82 |
| TM4SF20 | Osteoporosis, Postmenopausal | 2.82 |
| TMEM259 | Osteoporosis, Postmenopausal | 2.82 |
| UBA1 | Osteoporosis, Postmenopausal | 2.82 |
| UNC80 | Osteoporosis, Postmenopausal | 2.82 |
| ZNF532 | Osteoporosis, Postmenopausal | 2.82 |
| ADAM11 | Osteoporosis, Postmenopausal | 2.81 |
| AK1 | Osteoporosis, Postmenopausal | 2.81 |
| AMPD1 | Osteoporosis, Postmenopausal | 2.81 |
| ANKLE2 | Osteoporosis, Postmenopausal | 2.81 |
| ASAP3 | Osteoporosis, Postmenopausal | 2.81 |
| C1QTNF4 | Osteoporosis, Postmenopausal | 2.81 |
| CAPS2 | Osteoporosis, Postmenopausal | 2.81 |
| CDH18 | Osteoporosis, Postmenopausal | 2.81 |
| CES2C | Osteoporosis, Postmenopausal | 2.81 |
| CMAH | Osteoporosis, Postmenopausal | 2.81 |
| COLGALT1 | Osteoporosis, Postmenopausal | 2.81 |
| CTIF | Osteoporosis, Postmenopausal | 2.81 |
| CYP2C18 | Osteoporosis, Postmenopausal | 2.81 |
| DDX23 | Osteoporosis, Postmenopausal | 2.81 |
| DMAP1 | Osteoporosis, Postmenopausal | 2.81 |
| ETAA1 | Osteoporosis, Postmenopausal | 2.81 |
| FAR1 | Osteoporosis, Postmenopausal | 2.81 |
| FBLN7 | Osteoporosis, Postmenopausal | 2.81 |
| FBXO15 | Osteoporosis, Postmenopausal | 2.81 |
| FBXO36 | Osteoporosis, Postmenopausal | 2.81 |
| FMNL3 | Osteoporosis, Postmenopausal | 2.81 |
| FOXD3 | Osteoporosis, Postmenopausal | 2.81 |
| FRRS1 | Osteoporosis, Postmenopausal | 2.81 |
| GLE1 | Osteoporosis, Postmenopausal | 2.81 |
| H1F4 | Osteoporosis, Postmenopausal | 2.81 |
| HLA-DPB1 | Osteoporosis, Postmenopausal | 2.81 |
| HTR6 | Osteoporosis, Postmenopausal | 2.81 |
| IQCE | Osteoporosis, Postmenopausal | 2.81 |
| KCMF1 | Osteoporosis, Postmenopausal | 2.81 |
| MFSD12 | Osteoporosis, Postmenopausal | 2.81 |
| MORF4L2 | Osteoporosis, Postmenopausal | 2.81 |
| MPL | Osteoporosis, Postmenopausal | 2.81 |
| MRAP | Osteoporosis, Postmenopausal | 2.81 |
| NAA25 | Osteoporosis, Postmenopausal | 2.81 |
| NAE1 | Osteoporosis, Postmenopausal | 2.81 |
| NAP1L3 | Osteoporosis, Postmenopausal | 2.81 |
| NCBP1 | Osteoporosis, Postmenopausal | 2.81 |
| NCDN | Osteoporosis, Postmenopausal | 2.81 |
| NHSL2 | Osteoporosis, Postmenopausal | 2.81 |
| NUDT21 | Osteoporosis, Postmenopausal | 2.81 |
| PCED1B | Osteoporosis, Postmenopausal | 2.81 |
| PCSK2 | Osteoporosis, Postmenopausal | 2.81 |
| PDE8A | Osteoporosis, Postmenopausal | 2.81 |
| PLAGL1 | Osteoporosis, Postmenopausal | 2.81 |
| PLD6 | Osteoporosis, Postmenopausal | 2.81 |
| PLXNA1 | Osteoporosis, Postmenopausal | 2.81 |
| PMPCA | Osteoporosis, Postmenopausal | 2.81 |
| PNMA2 | Osteoporosis, Postmenopausal | 2.81 |
| PREB | Osteoporosis, Postmenopausal | 2.81 |
| PROZ | Osteoporosis, Postmenopausal | 2.81 |
| PUS7L | Osteoporosis, Postmenopausal | 2.81 |
| PYROXD1 | Osteoporosis, Postmenopausal | 2.81 |
| RAB5IF | Osteoporosis, Postmenopausal | 2.81 |
| RANGRF | Osteoporosis, Postmenopausal | 2.81 |
| RASGEF1A | Osteoporosis, Postmenopausal | 2.81 |
| RGS18 | Osteoporosis, Postmenopausal | 2.81 |
| SCRN3 | Osteoporosis, Postmenopausal | 2.81 |
| SDHD | Osteoporosis, Postmenopausal | 2.81 |
| SHISAL1 | Osteoporosis, Postmenopausal | 2.81 |
| SMARCA2 | Osteoporosis, Postmenopausal | 2.81 |
| SOX13 | Osteoporosis, Postmenopausal | 2.81 |
| SP6 | Osteoporosis, Postmenopausal | 2.81 |
| SPATA20 | Osteoporosis, Postmenopausal | 2.81 |
| SRA1 | Osteoporosis, Postmenopausal | 2.81 |
| STX7 | Osteoporosis, Postmenopausal | 2.81 |
| TEDC1 | Osteoporosis, Postmenopausal | 2.81 |
| TMCO4 | Osteoporosis, Postmenopausal | 2.81 |
| TMCO6 | Osteoporosis, Postmenopausal | 2.81 |
| TMEM132C | Osteoporosis, Postmenopausal | 2.81 |
| TREH | Osteoporosis, Postmenopausal | 2.81 |
| TSPOAP1 | Osteoporosis, Postmenopausal | 2.81 |
| UGGT1 | Osteoporosis, Postmenopausal | 2.81 |
| UGT2B37 | Osteoporosis, Postmenopausal | 2.81 |
| VPS13C | Osteoporosis, Postmenopausal | 2.81 |
| YBX2 | Osteoporosis, Postmenopausal | 2.81 |
| ZBTB48 | Osteoporosis, Postmenopausal | 2.81 |
| ZBTB8OS | Osteoporosis, Postmenopausal | 2.81 |
| ZFP503 | Osteoporosis, Postmenopausal | 2.81 |
| ZNHIT3 | Osteoporosis, Postmenopausal | 2.81 |
| ACTR3B | Osteoporosis, Postmenopausal | 2.8 |
| ACVR1B | Osteoporosis, Postmenopausal | 2.8 |
| ADH1A | Osteoporosis, Postmenopausal | 2.8 |
| AMER1 | Osteoporosis, Postmenopausal | 2.8 |
| ANKRD27 | Osteoporosis, Postmenopausal | 2.8 |
| APOBR | Osteoporosis, Postmenopausal | 2.8 |
| ARIH2 | Osteoporosis, Postmenopausal | 2.8 |
| ATP5MK | Osteoporosis, Postmenopausal | 2.8 |
| BTN3A2 | Osteoporosis, Postmenopausal | 2.8 |
| CCDC112 | Osteoporosis, Postmenopausal | 2.8 |
| CCDC124 | Osteoporosis, Postmenopausal | 2.8 |
| CFAP45 | Osteoporosis, Postmenopausal | 2.8 |
| CHMP1B | Osteoporosis, Postmenopausal | 2.8 |
| CHRNB1 | Osteoporosis, Postmenopausal | 2.8 |
| CLASRP | Osteoporosis, Postmenopausal | 2.8 |
| CLEC4A3 | Osteoporosis, Postmenopausal | 2.8 |
| COL4A4 | Osteoporosis, Postmenopausal | 2.8 |
| CRB3 | Osteoporosis, Postmenopausal | 2.8 |
| CTCF | Osteoporosis, Postmenopausal | 2.8 |
| CYP21A1 | Osteoporosis, Postmenopausal | 2.8 |
| CYTL1 | Osteoporosis, Postmenopausal | 2.8 |
| DHDH | Osteoporosis, Postmenopausal | 2.8 |
| DMRT1 | Osteoporosis, Postmenopausal | 2.8 |
| DNAAF1 | Osteoporosis, Postmenopausal | 2.8 |
| DNAJC11 | Osteoporosis, Postmenopausal | 2.8 |
| DRG1 | Osteoporosis, Postmenopausal | 2.8 |
| EDA | Osteoporosis, Postmenopausal | 2.8 |
| EMC6 | Osteoporosis, Postmenopausal | 2.8 |
| EML5 | Osteoporosis, Postmenopausal | 2.8 |
| EXOC3L4 | Osteoporosis, Postmenopausal | 2.8 |
| FBXL17 | Osteoporosis, Postmenopausal | 2.8 |
| FCF1 | Osteoporosis, Postmenopausal | 2.8 |
| FCGR1 | Osteoporosis, Postmenopausal | 2.8 |
| FHIP1A | Osteoporosis, Postmenopausal | 2.8 |
| FOXG1 | Osteoporosis, Postmenopausal | 2.8 |
| GLIS2 | Osteoporosis, Postmenopausal | 2.8 |
| GOLPH3L | Osteoporosis, Postmenopausal | 2.8 |
| GPR84 | Osteoporosis, Postmenopausal | 2.8 |
| GTF2H2 | Osteoporosis, Postmenopausal | 2.8 |
| GTPBP3 | Osteoporosis, Postmenopausal | 2.8 |
| HBA2 | Osteoporosis, Postmenopausal | 2.8 |
| HBE1 | Osteoporosis, Postmenopausal | 2.8 |
| IL11RA1 | Osteoporosis, Postmenopausal | 2.8 |
| IL7 | Osteoporosis, Postmenopausal | 2.8 |
| KCNE4 | Osteoporosis, Postmenopausal | 2.8 |
| KCNH3 | Osteoporosis, Postmenopausal | 2.8 |
| KCNQ4 | Osteoporosis, Postmenopausal | 2.8 |
| KIF9 | Osteoporosis, Postmenopausal | 2.8 |
| KLHL22 | Osteoporosis, Postmenopausal | 2.8 |
| KLHL28 | Osteoporosis, Postmenopausal | 2.8 |
| KLHL3 | Osteoporosis, Postmenopausal | 2.8 |
| LGI1 | Osteoporosis, Postmenopausal | 2.8 |
| LHX9 | Osteoporosis, Postmenopausal | 2.8 |
| LTK | Osteoporosis, Postmenopausal | 2.8 |
| MARCHF8 | Osteoporosis, Postmenopausal | 2.8 |
| MEIS3 | Osteoporosis, Postmenopausal | 2.8 |
| MGRN1 | Osteoporosis, Postmenopausal | 2.8 |
| MOBP | Osteoporosis, Postmenopausal | 2.8 |
| MRS2 | Osteoporosis, Postmenopausal | 2.8 |
| MRTFA | Osteoporosis, Postmenopausal | 2.8 |
| MSH3 | Osteoporosis, Postmenopausal | 2.8 |
| MTCL2 | Osteoporosis, Postmenopausal | 2.8 |
| MYH8 | Osteoporosis, Postmenopausal | 2.8 |
| NEURL2 | Osteoporosis, Postmenopausal | 2.8 |
| NICN1 | Osteoporosis, Postmenopausal | 2.8 |
| ODF2L | Osteoporosis, Postmenopausal | 2.8 |
| PCDHGC3 | Osteoporosis, Postmenopausal | 2.8 |
| PCGF2 | Osteoporosis, Postmenopausal | 2.8 |
| PDCD2L | Osteoporosis, Postmenopausal | 2.8 |
| PEAK1 | Osteoporosis, Postmenopausal | 2.8 |
| PHLPP2 | Osteoporosis, Postmenopausal | 2.8 |
| PISD | Osteoporosis, Postmenopausal | 2.8 |
| PKHD1L1 | Osteoporosis, Postmenopausal | 2.8 |
| PLEKHM3 | Osteoporosis, Postmenopausal | 2.8 |
| PLPPR4 | Osteoporosis, Postmenopausal | 2.8 |
| PMEL | Osteoporosis, Postmenopausal | 2.8 |
| RASSF9 | Osteoporosis, Postmenopausal | 2.8 |
| RBM41 | Osteoporosis, Postmenopausal | 2.8 |
| RETREG3 | Osteoporosis, Postmenopausal | 2.8 |
| RFX5 | Osteoporosis, Postmenopausal | 2.8 |
| RHOD | Osteoporosis, Postmenopausal | 2.8 |
| RIMS4 | Osteoporosis, Postmenopausal | 2.8 |
| RNF10 | Osteoporosis, Postmenopausal | 2.8 |
| RNF180 | Osteoporosis, Postmenopausal | 2.8 |
| RNF181 | Osteoporosis, Postmenopausal | 2.8 |
| RPL36AL | Osteoporosis, Postmenopausal | 2.8 |
| SART3 | Osteoporosis, Postmenopausal | 2.8 |
| SCAP | Osteoporosis, Postmenopausal | 2.8 |
| SELENOH | Osteoporosis, Postmenopausal | 2.8 |
| SENP6 | Osteoporosis, Postmenopausal | 2.8 |
| SGSH | Osteoporosis, Postmenopausal | 2.8 |
| SH3BP4 | Osteoporosis, Postmenopausal | 2.8 |
| SIX3 | Osteoporosis, Postmenopausal | 2.8 |
| SLC38A1 | Osteoporosis, Postmenopausal | 2.8 |
| SLC9A5 | Osteoporosis, Postmenopausal | 2.8 |
| SLC9A9 | Osteoporosis, Postmenopausal | 2.8 |
| SNRNP200 | Osteoporosis, Postmenopausal | 2.8 |
| SNRNP35 | Osteoporosis, Postmenopausal | 2.8 |
| SPDYA | Osteoporosis, Postmenopausal | 2.8 |
| SRPK3 | Osteoporosis, Postmenopausal | 2.8 |
| STAP1 | Osteoporosis, Postmenopausal | 2.8 |
| STARD7 | Osteoporosis, Postmenopausal | 2.8 |
| SUSD6 | Osteoporosis, Postmenopausal | 2.8 |
| SYDE2 | Osteoporosis, Postmenopausal | 2.8 |
| TERF1 | Osteoporosis, Postmenopausal | 2.8 |
| TESK2 | Osteoporosis, Postmenopausal | 2.8 |
| TM6SF1 | Osteoporosis, Postmenopausal | 2.8 |
| TPP1 | Osteoporosis, Postmenopausal | 2.8 |
| TRMT13 | Osteoporosis, Postmenopausal | 2.8 |
| TSEN54 | Osteoporosis, Postmenopausal | 2.8 |
| UBXN6 | Osteoporosis, Postmenopausal | 2.8 |
| UGT2B10 | Osteoporosis, Postmenopausal | 2.8 |
| UTP25 | Osteoporosis, Postmenopausal | 2.8 |
| VPS26A | Osteoporosis, Postmenopausal | 2.8 |
| WDR91 | Osteoporosis, Postmenopausal | 2.8 |
| ZC3HAV1L | Osteoporosis, Postmenopausal | 2.8 |
| ZDHHC9 | Osteoporosis, Postmenopausal | 2.8 |
| AGPS | Osteoporosis, Postmenopausal | 2.79 |
| AP2A1 | Osteoporosis, Postmenopausal | 2.79 |
| ARHGEF17 | Osteoporosis, Postmenopausal | 2.79 |
| ARL8B | Osteoporosis, Postmenopausal | 2.79 |
| ARPP21 | Osteoporosis, Postmenopausal | 2.79 |
| ASPHD2 | Osteoporosis, Postmenopausal | 2.79 |
| ATP6V1H | Osteoporosis, Postmenopausal | 2.79 |
| BLOC1S1 | Osteoporosis, Postmenopausal | 2.79 |
| BRPF1 | Osteoporosis, Postmenopausal | 2.79 |
| BUD31 | Osteoporosis, Postmenopausal | 2.79 |
| CCL27 | Osteoporosis, Postmenopausal | 2.79 |
| CHPF2 | Osteoporosis, Postmenopausal | 2.79 |
| CLDN9 | Osteoporosis, Postmenopausal | 2.79 |
| CMTM4 | Osteoporosis, Postmenopausal | 2.79 |
| COA5 | Osteoporosis, Postmenopausal | 2.79 |
| COX15 | Osteoporosis, Postmenopausal | 2.79 |
| CPPED1 | Osteoporosis, Postmenopausal | 2.79 |
| CREBZF | Osteoporosis, Postmenopausal | 2.79 |
| DDX31 | Osteoporosis, Postmenopausal | 2.79 |
| DDX47 | Osteoporosis, Postmenopausal | 2.79 |
| DPEP2 | Osteoporosis, Postmenopausal | 2.79 |
| EEF2KMT | Osteoporosis, Postmenopausal | 2.79 |
| EIF1AD | Osteoporosis, Postmenopausal | 2.79 |
| EIF1B | Osteoporosis, Postmenopausal | 2.79 |
| ELMOD3 | Osteoporosis, Postmenopausal | 2.79 |
| FAM124A | Osteoporosis, Postmenopausal | 2.79 |
| GABPB1 | Osteoporosis, Postmenopausal | 2.79 |
| GBA1 | Osteoporosis, Postmenopausal | 2.79 |
| GIGYF1 | Osteoporosis, Postmenopausal | 2.79 |
| GOT2 | Osteoporosis, Postmenopausal | 2.79 |
| HNRNPH2 | Osteoporosis, Postmenopausal | 2.79 |
| INPP5J | Osteoporosis, Postmenopausal | 2.79 |
| IP6K3 | Osteoporosis, Postmenopausal | 2.79 |
| KLC1 | Osteoporosis, Postmenopausal | 2.79 |
| KLHL6 | Osteoporosis, Postmenopausal | 2.79 |
| LLGL1 | Osteoporosis, Postmenopausal | 2.79 |
| LSM6 | Osteoporosis, Postmenopausal | 2.79 |
| LYL1 | Osteoporosis, Postmenopausal | 2.79 |
| MAT2B | Osteoporosis, Postmenopausal | 2.79 |
| MRPL17 | Osteoporosis, Postmenopausal | 2.79 |
| MRPL55 | Osteoporosis, Postmenopausal | 2.79 |
| MRPS21 | Osteoporosis, Postmenopausal | 2.79 |
| MSTN | Osteoporosis, Postmenopausal | 2.79 |
| MT1L | Osteoporosis, Postmenopausal | 2.79 |
| MTPN | Osteoporosis, Postmenopausal | 2.79 |
| MYL6B | Osteoporosis, Postmenopausal | 2.79 |
| MYO7B | Osteoporosis, Postmenopausal | 2.79 |
| MYOZ2 | Osteoporosis, Postmenopausal | 2.79 |
| N4BP1 | Osteoporosis, Postmenopausal | 2.79 |
| NAGK | Osteoporosis, Postmenopausal | 2.79 |
| NEU3 | Osteoporosis, Postmenopausal | 2.79 |
| NUP43 | Osteoporosis, Postmenopausal | 2.79 |
| PCBP4 | Osteoporosis, Postmenopausal | 2.79 |
| PLAAT1 | Osteoporosis, Postmenopausal | 2.79 |
| PRRC2A | Osteoporosis, Postmenopausal | 2.79 |
| RABEP2 | Osteoporosis, Postmenopausal | 2.79 |
| RBM7 | Osteoporosis, Postmenopausal | 2.79 |
| RPL29 | Osteoporosis, Postmenopausal | 2.79 |
| SCAMP5 | Osteoporosis, Postmenopausal | 2.79 |
| SEPTIN3 | Osteoporosis, Postmenopausal | 2.79 |
| SERPINB6B | Osteoporosis, Postmenopausal | 2.79 |
| SLC17A8 | Osteoporosis, Postmenopausal | 2.79 |
| SLC25A11 | Osteoporosis, Postmenopausal | 2.79 |
| SLC9B2 | Osteoporosis, Postmenopausal | 2.79 |
| SMCO4 | Osteoporosis, Postmenopausal | 2.79 |
| SNRPE | Osteoporosis, Postmenopausal | 2.79 |
| SPAG6 | Osteoporosis, Postmenopausal | 2.79 |
| ST8SIA3 | Osteoporosis, Postmenopausal | 2.79 |
| SURF1 | Osteoporosis, Postmenopausal | 2.79 |
| TARBP2 | Osteoporosis, Postmenopausal | 2.79 |
| TCAIM | Osteoporosis, Postmenopausal | 2.79 |
| TEX264 | Osteoporosis, Postmenopausal | 2.79 |
| TLK2 | Osteoporosis, Postmenopausal | 2.79 |
| TLNRD1 | Osteoporosis, Postmenopausal | 2.79 |
| TMEM254 | Osteoporosis, Postmenopausal | 2.79 |
| TMEM69 | Osteoporosis, Postmenopausal | 2.79 |
| TRABD2B | Osteoporosis, Postmenopausal | 2.79 |
| TRUB1 | Osteoporosis, Postmenopausal | 2.79 |
| TXLNB | Osteoporosis, Postmenopausal | 2.79 |
| USP24 | Osteoporosis, Postmenopausal | 2.79 |
| VAPA | Osteoporosis, Postmenopausal | 2.79 |
| VDAC2 | Osteoporosis, Postmenopausal | 2.79 |
| VPS13B | Osteoporosis, Postmenopausal | 2.79 |
| VPS29 | Osteoporosis, Postmenopausal | 2.79 |
| WDR35 | Osteoporosis, Postmenopausal | 2.79 |
| ZBTB20 | Osteoporosis, Postmenopausal | 2.79 |
| ZNHIT2 | Osteoporosis, Postmenopausal | 2.79 |
| ZNHIT6 | Osteoporosis, Postmenopausal | 2.79 |
| ABHD17B | Osteoporosis, Postmenopausal | 2.78 |
| ADCY10 | Osteoporosis, Postmenopausal | 2.78 |
| ADGRA2 | Osteoporosis, Postmenopausal | 2.78 |
| ANKRD24 | Osteoporosis, Postmenopausal | 2.78 |
| AP3B1 | Osteoporosis, Postmenopausal | 2.78 |
| ARL6IP4 | Osteoporosis, Postmenopausal | 2.78 |
| BTBD7 | Osteoporosis, Postmenopausal | 2.78 |
| CADM4 | Osteoporosis, Postmenopausal | 2.78 |
| CDC42BPG | Osteoporosis, Postmenopausal | 2.78 |
| CHERP | Osteoporosis, Postmenopausal | 2.78 |
| CHIC1 | Osteoporosis, Postmenopausal | 2.78 |
| COL24A1 | Osteoporosis, Postmenopausal | 2.78 |
| CUL1 | Osteoporosis, Postmenopausal | 2.78 |
| DOK2 | Osteoporosis, Postmenopausal | 2.78 |
| EVA1C | Osteoporosis, Postmenopausal | 2.78 |
| FGD2 | Osteoporosis, Postmenopausal | 2.78 |
| FOSAB | Osteoporosis, Postmenopausal | 2.78 |
| GNS | Osteoporosis, Postmenopausal | 2.78 |
| GOLT1B | Osteoporosis, Postmenopausal | 2.78 |
| GPATCH8 | Osteoporosis, Postmenopausal | 2.78 |
| HBB-B1 | Osteoporosis, Postmenopausal | 2.78 |
| HTATSF1 | Osteoporosis, Postmenopausal | 2.78 |
| IFT140 | Osteoporosis, Postmenopausal | 2.78 |
| IQCG | Osteoporosis, Postmenopausal | 2.78 |
| IRGM2 | Osteoporosis, Postmenopausal | 2.78 |
| KNDC1 | Osteoporosis, Postmenopausal | 2.78 |
| LPAR4 | Osteoporosis, Postmenopausal | 2.78 |
| MBTPS1 | Osteoporosis, Postmenopausal | 2.78 |
| MINDY3 | Osteoporosis, Postmenopausal | 2.78 |
| MMP25 | Osteoporosis, Postmenopausal | 2.78 |
| NALCN | Osteoporosis, Postmenopausal | 2.78 |
| PMF1 | Osteoporosis, Postmenopausal | 2.78 |
| POPDC1 | Osteoporosis, Postmenopausal | 2.78 |
| PRAM1 | Osteoporosis, Postmenopausal | 2.78 |
| PRR13 | Osteoporosis, Postmenopausal | 2.78 |
| RARS1 | Osteoporosis, Postmenopausal | 2.78 |
| RFXANK | Osteoporosis, Postmenopausal | 2.78 |
| RGL2 | Osteoporosis, Postmenopausal | 2.78 |
| RNF126 | Osteoporosis, Postmenopausal | 2.78 |
| RPL13 | Osteoporosis, Postmenopausal | 2.78 |
| RPP25L | Osteoporosis, Postmenopausal | 2.78 |
| SERGEF | Osteoporosis, Postmenopausal | 2.78 |
| SHISA3 | Osteoporosis, Postmenopausal | 2.78 |
| SIM2 | Osteoporosis, Postmenopausal | 2.78 |
| SIRT4 | Osteoporosis, Postmenopausal | 2.78 |
| SLC17A1 | Osteoporosis, Postmenopausal | 2.78 |
| SNRPC | Osteoporosis, Postmenopausal | 2.78 |
| SPEG | Osteoporosis, Postmenopausal | 2.78 |
| STRADB | Osteoporosis, Postmenopausal | 2.78 |
| SYCP1 | Osteoporosis, Postmenopausal | 2.78 |
| TAAR1 | Osteoporosis, Postmenopausal | 2.78 |
| TFEC | Osteoporosis, Postmenopausal | 2.78 |
| TGDS | Osteoporosis, Postmenopausal | 2.78 |
| TM4SF19 | Osteoporosis, Postmenopausal | 2.78 |
| TM9SF3 | Osteoporosis, Postmenopausal | 2.78 |
| TMEM134 | Osteoporosis, Postmenopausal | 2.78 |
| TMEM161A | Osteoporosis, Postmenopausal | 2.78 |
| TTC38 | Osteoporosis, Postmenopausal | 2.78 |
| UBE2F | Osteoporosis, Postmenopausal | 2.78 |
| UPK1B | Osteoporosis, Postmenopausal | 2.78 |
| ZBTB46 | Osteoporosis, Postmenopausal | 2.78 |
| ZDHHC5 | Osteoporosis, Postmenopausal | 2.78 |
| ZMYND10 | Osteoporosis, Postmenopausal | 2.78 |
| ACTR2 | Osteoporosis, Postmenopausal | 2.77 |
| ADAMTS10 | Osteoporosis, Postmenopausal | 2.77 |
| ADPRHL1 | Osteoporosis, Postmenopausal | 2.77 |
| ALKBH8 | Osteoporosis, Postmenopausal | 2.77 |
| ARHGAP6 | Osteoporosis, Postmenopausal | 2.77 |
| ATP13A4 | Osteoporosis, Postmenopausal | 2.77 |
| ATP8 | Osteoporosis, Postmenopausal | 2.77 |
| BRD3 | Osteoporosis, Postmenopausal | 2.77 |
| CCDC71L | Osteoporosis, Postmenopausal | 2.77 |
| CCL25 | Osteoporosis, Postmenopausal | 2.77 |
| CIMAP1B | Osteoporosis, Postmenopausal | 2.77 |
| CKAP5 | Osteoporosis, Postmenopausal | 2.77 |
| CNRIP1 | Osteoporosis, Postmenopausal | 2.77 |
| COX8A | Osteoporosis, Postmenopausal | 2.77 |
| CPSF6 | Osteoporosis, Postmenopausal | 2.77 |
| CWC27 | Osteoporosis, Postmenopausal | 2.77 |
| CYP3A18 | Osteoporosis, Postmenopausal | 2.77 |
| DHX15 | Osteoporosis, Postmenopausal | 2.77 |
| DNAH12 | Osteoporosis, Postmenopausal | 2.77 |
| EIF1AX | Osteoporosis, Postmenopausal | 2.77 |
| ELL3 | Osteoporosis, Postmenopausal | 2.77 |
| EMID1 | Osteoporosis, Postmenopausal | 2.77 |
| EPN2 | Osteoporosis, Postmenopausal | 2.77 |
| FAM169A | Osteoporosis, Postmenopausal | 2.77 |
| FZD10 | Osteoporosis, Postmenopausal | 2.77 |
| GIMAP6 | Osteoporosis, Postmenopausal | 2.77 |
| GPR4 | Osteoporosis, Postmenopausal | 2.77 |
| IFNGR1 | Osteoporosis, Postmenopausal | 2.77 |
| MAP3K7CL | Osteoporosis, Postmenopausal | 2.77 |
| MBD5 | Osteoporosis, Postmenopausal | 2.77 |
| MMRN2 | Osteoporosis, Postmenopausal | 2.77 |
| MRPL30 | Osteoporosis, Postmenopausal | 2.77 |
| MT1B | Osteoporosis, Postmenopausal | 2.77 |
| MYH4 | Osteoporosis, Postmenopausal | 2.77 |
| NAGPA | Osteoporosis, Postmenopausal | 2.77 |
| NKRF | Osteoporosis, Postmenopausal | 2.77 |
| NMNAT3 | Osteoporosis, Postmenopausal | 2.77 |
| NPEPPS | Osteoporosis, Postmenopausal | 2.77 |
| NUDT2 | Osteoporosis, Postmenopausal | 2.77 |
| PAIP2 | Osteoporosis, Postmenopausal | 2.77 |
| PANX1 | Osteoporosis, Postmenopausal | 2.77 |
| PAXIP1 | Osteoporosis, Postmenopausal | 2.77 |
| PDZD8 | Osteoporosis, Postmenopausal | 2.77 |
| PFDN1 | Osteoporosis, Postmenopausal | 2.77 |
| PIGC | Osteoporosis, Postmenopausal | 2.77 |
| PMPCB | Osteoporosis, Postmenopausal | 2.77 |
| PTGR3 | Osteoporosis, Postmenopausal | 2.77 |
| RHOJ | Osteoporosis, Postmenopausal | 2.77 |
| RPH3AL | Osteoporosis, Postmenopausal | 2.77 |
| RT1-DA | Osteoporosis, Postmenopausal | 2.77 |
| SERPINB7 | Osteoporosis, Postmenopausal | 2.77 |
| SF3A1 | Osteoporosis, Postmenopausal | 2.77 |
| SLC5A11 | Osteoporosis, Postmenopausal | 2.77 |
| SLC6A8 | Osteoporosis, Postmenopausal | 2.77 |
| SLX4 | Osteoporosis, Postmenopausal | 2.77 |
| SMYD1 | Osteoporosis, Postmenopausal | 2.77 |
| SNRPN | Osteoporosis, Postmenopausal | 2.77 |
| SNX29 | Osteoporosis, Postmenopausal | 2.77 |
| SPHK2 | Osteoporosis, Postmenopausal | 2.77 |
| STAU1 | Osteoporosis, Postmenopausal | 2.77 |
| SYNE2 | Osteoporosis, Postmenopausal | 2.77 |
| TAPBPL | Osteoporosis, Postmenopausal | 2.77 |
| TDP1 | Osteoporosis, Postmenopausal | 2.77 |
| TMEM160 | Osteoporosis, Postmenopausal | 2.77 |
| TMEM25 | Osteoporosis, Postmenopausal | 2.77 |
| TOR1A | Osteoporosis, Postmenopausal | 2.77 |
| TRIM69 | Osteoporosis, Postmenopausal | 2.77 |
| TXLNG | Osteoporosis, Postmenopausal | 2.77 |
| UBL5 | Osteoporosis, Postmenopausal | 2.77 |
| UBL7 | Osteoporosis, Postmenopausal | 2.77 |
| UQCR11 | Osteoporosis, Postmenopausal | 2.77 |
| YEATS2 | Osteoporosis, Postmenopausal | 2.77 |
| ZHX1 | Osteoporosis, Postmenopausal | 2.77 |
| ZNRD2 | Osteoporosis, Postmenopausal | 2.77 |
| ZZEF1 | Osteoporosis, Postmenopausal | 2.77 |
| ACOD1 | Osteoporosis, Postmenopausal | 2.76 |
| ADGRF1 | Osteoporosis, Postmenopausal | 2.76 |
| AIP | Osteoporosis, Postmenopausal | 2.76 |
| AKIP1 | Osteoporosis, Postmenopausal | 2.76 |
| ALKBH7 | Osteoporosis, Postmenopausal | 2.76 |
| ANAPC11 | Osteoporosis, Postmenopausal | 2.76 |
| ANKRD6 | Osteoporosis, Postmenopausal | 2.76 |
| AP2B1 | Osteoporosis, Postmenopausal | 2.76 |
| APLF | Osteoporosis, Postmenopausal | 2.76 |
| ARFIP2 | Osteoporosis, Postmenopausal | 2.76 |
| ATP8B4 | Osteoporosis, Postmenopausal | 2.76 |
| AUP1 | Osteoporosis, Postmenopausal | 2.76 |
| BCL9L | Osteoporosis, Postmenopausal | 2.76 |
| CCL1 | Osteoporosis, Postmenopausal | 2.76 |
| CDK12 | Osteoporosis, Postmenopausal | 2.76 |
| CFAP20 | Osteoporosis, Postmenopausal | 2.76 |
| CHCHD3 | Osteoporosis, Postmenopausal | 2.76 |
| CHMP2A | Osteoporosis, Postmenopausal | 2.76 |
| COX10 | Osteoporosis, Postmenopausal | 2.76 |
| CYP11B1 | Osteoporosis, Postmenopausal | 2.76 |
| CYP21A2 | Osteoporosis, Postmenopausal | 2.76 |
| DCBLD1 | Osteoporosis, Postmenopausal | 2.76 |
| DNAH5 | Osteoporosis, Postmenopausal | 2.76 |
| DRAM2 | Osteoporosis, Postmenopausal | 2.76 |
| EFTUD2 | Osteoporosis, Postmenopausal | 2.76 |
| EIF2B4 | Osteoporosis, Postmenopausal | 2.76 |
| ELOC | Osteoporosis, Postmenopausal | 2.76 |
| FBXO9 | Osteoporosis, Postmenopausal | 2.76 |
| FTSJ1 | Osteoporosis, Postmenopausal | 2.76 |
| GRIK1 | Osteoporosis, Postmenopausal | 2.76 |
| GRIN3B | Osteoporosis, Postmenopausal | 2.76 |
| GRM4 | Osteoporosis, Postmenopausal | 2.76 |
| HIC2 | Osteoporosis, Postmenopausal | 2.76 |
| HLA-G | Osteoporosis, Postmenopausal | 2.76 |
| HNF1B | Osteoporosis, Postmenopausal | 2.76 |
| INTS6 | Osteoporosis, Postmenopausal | 2.76 |
| KIDINS220 | Osteoporosis, Postmenopausal | 2.76 |
| KLRD1 | Osteoporosis, Postmenopausal | 2.76 |
| MIER3 | Osteoporosis, Postmenopausal | 2.76 |
| MIR15B | Osteoporosis, Postmenopausal | 2.76 |
| NDUFA7 | Osteoporosis, Postmenopausal | 2.76 |
| OTX2 | Osteoporosis, Postmenopausal | 2.76 |
| POLK | Osteoporosis, Postmenopausal | 2.76 |
| POMT1 | Osteoporosis, Postmenopausal | 2.76 |
| PWWP2B | Osteoporosis, Postmenopausal | 2.76 |
| RNH1 | Osteoporosis, Postmenopausal | 2.76 |
| SAR1A | Osteoporosis, Postmenopausal | 2.76 |
| SCAF4 | Osteoporosis, Postmenopausal | 2.76 |
| SCRN1 | Osteoporosis, Postmenopausal | 2.76 |
| SERPINB1A | Osteoporosis, Postmenopausal | 2.76 |
| SGSM1 | Osteoporosis, Postmenopausal | 2.76 |
| SLC2A13 | Osteoporosis, Postmenopausal | 2.76 |
| SLC35E4 | Osteoporosis, Postmenopausal | 2.76 |
| SLC9A3 | Osteoporosis, Postmenopausal | 2.76 |
| SRCAP | Osteoporosis, Postmenopausal | 2.76 |
| SSRP1 | Osteoporosis, Postmenopausal | 2.76 |
| ST3GAL3 | Osteoporosis, Postmenopausal | 2.76 |
| TCTN1 | Osteoporosis, Postmenopausal | 2.76 |
| TMOD4 | Osteoporosis, Postmenopausal | 2.76 |
| TPCN2 | Osteoporosis, Postmenopausal | 2.76 |
| TRMT1L | Osteoporosis, Postmenopausal | 2.76 |
| TTC12 | Osteoporosis, Postmenopausal | 2.76 |
| TXNRD2 | Osteoporosis, Postmenopausal | 2.76 |
| UGT2B35 | Osteoporosis, Postmenopausal | 2.76 |
| VARS2 | Osteoporosis, Postmenopausal | 2.76 |
| ZMYND15 | Osteoporosis, Postmenopausal | 2.76 |
| ABI3 | Osteoporosis, Postmenopausal | 2.75 |
| ANKZF1 | Osteoporosis, Postmenopausal | 2.75 |
| AQP8 | Osteoporosis, Postmenopausal | 2.75 |
| ARMCX1 | Osteoporosis, Postmenopausal | 2.75 |
| ASMTL | Osteoporosis, Postmenopausal | 2.75 |
| AZIN2 | Osteoporosis, Postmenopausal | 2.75 |
| BCORL1 | Osteoporosis, Postmenopausal | 2.75 |
| BEGAIN | Osteoporosis, Postmenopausal | 2.75 |
| CBLN1 | Osteoporosis, Postmenopausal | 2.75 |
| CCDC28A | Osteoporosis, Postmenopausal | 2.75 |
| CCDC82 | Osteoporosis, Postmenopausal | 2.75 |
| CD1D1 | Osteoporosis, Postmenopausal | 2.75 |
| CD248 | Osteoporosis, Postmenopausal | 2.75 |
| CDYL2 | Osteoporosis, Postmenopausal | 2.75 |
| CECR2 | Osteoporosis, Postmenopausal | 2.75 |
| CEP131 | Osteoporosis, Postmenopausal | 2.75 |
| CHRNA9 | Osteoporosis, Postmenopausal | 2.75 |
| CSRNP3 | Osteoporosis, Postmenopausal | 2.75 |
| CYB5R1 | Osteoporosis, Postmenopausal | 2.75 |
| DERL2 | Osteoporosis, Postmenopausal | 2.75 |
| EIF1A | Osteoporosis, Postmenopausal | 2.75 |
| EPOP | Osteoporosis, Postmenopausal | 2.75 |
| EXOSC5 | Osteoporosis, Postmenopausal | 2.75 |
| FBXO6 | Osteoporosis, Postmenopausal | 2.75 |
| FBXW5 | Osteoporosis, Postmenopausal | 2.75 |
| FCGR1A | Osteoporosis, Postmenopausal | 2.75 |
| FCRL2 | Osteoporosis, Postmenopausal | 2.75 |
| FGD1 | Osteoporosis, Postmenopausal | 2.75 |
| GLYR1 | Osteoporosis, Postmenopausal | 2.75 |
| HAPLN3 | Osteoporosis, Postmenopausal | 2.75 |
| HIRA | Osteoporosis, Postmenopausal | 2.75 |
| HOXB7 | Osteoporosis, Postmenopausal | 2.75 |
| IFI204 | Osteoporosis, Postmenopausal | 2.75 |
| IFT81 | Osteoporosis, Postmenopausal | 2.75 |
| IMP4 | Osteoporosis, Postmenopausal | 2.75 |
| KCTD3 | Osteoporosis, Postmenopausal | 2.75 |
| KPNA6 | Osteoporosis, Postmenopausal | 2.75 |
| LARS1 | Osteoporosis, Postmenopausal | 2.75 |
| LONRF2 | Osteoporosis, Postmenopausal | 2.75 |
| LRIF1 | Osteoporosis, Postmenopausal | 2.75 |
| MACC1 | Osteoporosis, Postmenopausal | 2.75 |
| MAD2L2 | Osteoporosis, Postmenopausal | 2.75 |
| MIR150 | Osteoporosis, Postmenopausal | 2.75 |
| MIR99A | Osteoporosis, Postmenopausal | 2.75 |
| MPPE1 | Osteoporosis, Postmenopausal | 2.75 |
| MPV17 | Osteoporosis, Postmenopausal | 2.75 |
| MRPL19 | Osteoporosis, Postmenopausal | 2.75 |
| MRPL52 | Osteoporosis, Postmenopausal | 2.75 |
| MTCH1 | Osteoporosis, Postmenopausal | 2.75 |
| MYPN | Osteoporosis, Postmenopausal | 2.75 |
| NANP | Osteoporosis, Postmenopausal | 2.75 |
| NDUFS8 | Osteoporosis, Postmenopausal | 2.75 |
| NECAP1 | Osteoporosis, Postmenopausal | 2.75 |
| NOXA1 | Osteoporosis, Postmenopausal | 2.75 |
| NRG4 | Osteoporosis, Postmenopausal | 2.75 |
| NSG2 | Osteoporosis, Postmenopausal | 2.75 |
| NSUN5 | Osteoporosis, Postmenopausal | 2.75 |
| NUFIP1 | Osteoporosis, Postmenopausal | 2.75 |
| ODAD4 | Osteoporosis, Postmenopausal | 2.75 |
| ODF2 | Osteoporosis, Postmenopausal | 2.75 |
| ORAI2 | Osteoporosis, Postmenopausal | 2.75 |
| P3H1 | Osteoporosis, Postmenopausal | 2.75 |
| PAOX | Osteoporosis, Postmenopausal | 2.75 |
| PCSK4 | Osteoporosis, Postmenopausal | 2.75 |
| PGGHG | Osteoporosis, Postmenopausal | 2.75 |
| PHF12 | Osteoporosis, Postmenopausal | 2.75 |
| PI4K2B | Osteoporosis, Postmenopausal | 2.75 |
| PITPNM2 | Osteoporosis, Postmenopausal | 2.75 |
| POLE4 | Osteoporosis, Postmenopausal | 2.75 |
| PPP1CC | Osteoporosis, Postmenopausal | 2.75 |
| PPP4C | Osteoporosis, Postmenopausal | 2.75 |
| PRMT8 | Osteoporosis, Postmenopausal | 2.75 |
| PTGES2 | Osteoporosis, Postmenopausal | 2.75 |
| RAD17 | Osteoporosis, Postmenopausal | 2.75 |
| RASL12 | Osteoporosis, Postmenopausal | 2.75 |
| RFLNA | Osteoporosis, Postmenopausal | 2.75 |
| RGS8 | Osteoporosis, Postmenopausal | 2.75 |
| RILP | Osteoporosis, Postmenopausal | 2.75 |
| RIOK1 | Osteoporosis, Postmenopausal | 2.75 |
| RNMT | Osteoporosis, Postmenopausal | 2.75 |
| SEC14L4 | Osteoporosis, Postmenopausal | 2.75 |
| SLAIN1 | Osteoporosis, Postmenopausal | 2.75 |
| SLC35F6 | Osteoporosis, Postmenopausal | 2.75 |
| SMYD4 | Osteoporosis, Postmenopausal | 2.75 |
| SNX13 | Osteoporosis, Postmenopausal | 2.75 |
| SPATA17 | Osteoporosis, Postmenopausal | 2.75 |
| STK32C | Osteoporosis, Postmenopausal | 2.75 |
| STK35 | Osteoporosis, Postmenopausal | 2.75 |
| SUPT5H | Osteoporosis, Postmenopausal | 2.75 |
| TBL2 | Osteoporosis, Postmenopausal | 2.75 |
| TDG | Osteoporosis, Postmenopausal | 2.75 |
| TMEM256 | Osteoporosis, Postmenopausal | 2.75 |
| TNIP2 | Osteoporosis, Postmenopausal | 2.75 |
| TNXB | Osteoporosis, Postmenopausal | 2.75 |
| USP31 | Osteoporosis, Postmenopausal | 2.75 |
| WDR90 | Osteoporosis, Postmenopausal | 2.75 |
| WNT1 | Osteoporosis, Postmenopausal | 2.75 |
| WWC3 | Osteoporosis, Postmenopausal | 2.75 |
| ZBTB7C | Osteoporosis, Postmenopausal | 2.75 |
| ZRSR2 | Osteoporosis, Postmenopausal | 2.75 |
| ATP8B3 | Osteoporosis, Postmenopausal | 2.74 |
| BAHCC1 | Osteoporosis, Postmenopausal | 2.74 |
| CCNJ | Osteoporosis, Postmenopausal | 2.74 |
| CDHR1 | Osteoporosis, Postmenopausal | 2.74 |
| CEP41 | Osteoporosis, Postmenopausal | 2.74 |
| CHD5 | Osteoporosis, Postmenopausal | 2.74 |
| CLEC14A | Osteoporosis, Postmenopausal | 2.74 |
| CTTNBP2NL | Osteoporosis, Postmenopausal | 2.74 |
| CUL4A | Osteoporosis, Postmenopausal | 2.74 |
| DYRK1A | Osteoporosis, Postmenopausal | 2.74 |
| EGFL7 | Osteoporosis, Postmenopausal | 2.74 |
| ELFN1 | Osteoporosis, Postmenopausal | 2.74 |
| GCN1 | Osteoporosis, Postmenopausal | 2.74 |
| GPC2 | Osteoporosis, Postmenopausal | 2.74 |
| GPR157 | Osteoporosis, Postmenopausal | 2.74 |
| GRAP2 | Osteoporosis, Postmenopausal | 2.74 |
| GSTM4 | Osteoporosis, Postmenopausal | 2.74 |
| GTF3C4 | Osteoporosis, Postmenopausal | 2.74 |
| HAS3 | Osteoporosis, Postmenopausal | 2.74 |
| HLA-DPA1 | Osteoporosis, Postmenopausal | 2.74 |
| KCNA5 | Osteoporosis, Postmenopausal | 2.74 |
| LETMD1 | Osteoporosis, Postmenopausal | 2.74 |
| LOXL3 | Osteoporosis, Postmenopausal | 2.74 |
| LZTS3 | Osteoporosis, Postmenopausal | 2.74 |
| MC4R | Osteoporosis, Postmenopausal | 2.74 |
| MIF4GD | Osteoporosis, Postmenopausal | 2.74 |
| MTSS2 | Osteoporosis, Postmenopausal | 2.74 |
| NDC1 | Osteoporosis, Postmenopausal | 2.74 |
| NDUFS5 | Osteoporosis, Postmenopausal | 2.74 |
| PAKAP | Osteoporosis, Postmenopausal | 2.74 |
| PAX7 | Osteoporosis, Postmenopausal | 2.74 |
| PKIG | Osteoporosis, Postmenopausal | 2.74 |
| PLA2G3 | Osteoporosis, Postmenopausal | 2.74 |
| PLEKHA4 | Osteoporosis, Postmenopausal | 2.74 |
| PLSCR2 | Osteoporosis, Postmenopausal | 2.74 |
| PPP4R1 | Osteoporosis, Postmenopausal | 2.74 |
| RAP1B | Osteoporosis, Postmenopausal | 2.74 |
| RC3H1 | Osteoporosis, Postmenopausal | 2.74 |
| RGS14 | Osteoporosis, Postmenopausal | 2.74 |
| RHBDD1 | Osteoporosis, Postmenopausal | 2.74 |
| RIMKLB | Osteoporosis, Postmenopausal | 2.74 |
| RNF114 | Osteoporosis, Postmenopausal | 2.74 |
| SHTN1 | Osteoporosis, Postmenopausal | 2.74 |
| SINHCAF | Osteoporosis, Postmenopausal | 2.74 |
| SLC27A3 | Osteoporosis, Postmenopausal | 2.74 |
| SLC4A8 | Osteoporosis, Postmenopausal | 2.74 |
| SLC50A1 | Osteoporosis, Postmenopausal | 2.74 |
| SMARCE1 | Osteoporosis, Postmenopausal | 2.74 |
| SRFBP1 | Osteoporosis, Postmenopausal | 2.74 |
| SYPL1 | Osteoporosis, Postmenopausal | 2.74 |
| TMEM80 | Osteoporosis, Postmenopausal | 2.74 |
| TOX2 | Osteoporosis, Postmenopausal | 2.74 |
| USP45 | Osteoporosis, Postmenopausal | 2.74 |
| WDR74 | Osteoporosis, Postmenopausal | 2.74 |
| ACAP1 | Osteoporosis, Postmenopausal | 2.73 |
| ADAMTSL5 | Osteoporosis, Postmenopausal | 2.73 |
| AMN | Osteoporosis, Postmenopausal | 2.73 |
| ANKRD29 | Osteoporosis, Postmenopausal | 2.73 |
| ATP5ME | Osteoporosis, Postmenopausal | 2.73 |
| ATXN7 | Osteoporosis, Postmenopausal | 2.73 |
| CALHM2 | Osteoporosis, Postmenopausal | 2.73 |
| CANT1 | Osteoporosis, Postmenopausal | 2.73 |
| CDK2AP1 | Osteoporosis, Postmenopausal | 2.73 |
| COL4A6 | Osteoporosis, Postmenopausal | 2.73 |
| COL9A1 | Osteoporosis, Postmenopausal | 2.73 |
| DMC1 | Osteoporosis, Postmenopausal | 2.73 |
| DNAH11 | Osteoporosis, Postmenopausal | 2.73 |
| EEF1E1 | Osteoporosis, Postmenopausal | 2.73 |
| EFNA2 | Osteoporosis, Postmenopausal | 2.73 |
| FBXO33 | Osteoporosis, Postmenopausal | 2.73 |
| GHITM | Osteoporosis, Postmenopausal | 2.73 |
| GNB3 | Osteoporosis, Postmenopausal | 2.73 |
| GOSR1 | Osteoporosis, Postmenopausal | 2.73 |
| GPR132 | Osteoporosis, Postmenopausal | 2.73 |
| HES5 | Osteoporosis, Postmenopausal | 2.73 |
| ING4 | Osteoporosis, Postmenopausal | 2.73 |
| IRX2 | Osteoporosis, Postmenopausal | 2.73 |
| JAGN1 | Osteoporosis, Postmenopausal | 2.73 |
| KARS1 | Osteoporosis, Postmenopausal | 2.73 |
| LATS1 | Osteoporosis, Postmenopausal | 2.73 |
| LCMT1 | Osteoporosis, Postmenopausal | 2.73 |
| LIN37 | Osteoporosis, Postmenopausal | 2.73 |
| LRRC32 | Osteoporosis, Postmenopausal | 2.73 |
| MAP1S | Osteoporosis, Postmenopausal | 2.73 |
| MLYCD | Osteoporosis, Postmenopausal | 2.73 |
| NCLN | Osteoporosis, Postmenopausal | 2.73 |
| NOC4L | Osteoporosis, Postmenopausal | 2.73 |
| NOMO1 | Osteoporosis, Postmenopausal | 2.73 |
| NXT1 | Osteoporosis, Postmenopausal | 2.73 |
| PHKA1 | Osteoporosis, Postmenopausal | 2.73 |
| POLD3 | Osteoporosis, Postmenopausal | 2.73 |
| PPP6R1 | Osteoporosis, Postmenopausal | 2.73 |
| PRX | Osteoporosis, Postmenopausal | 2.73 |
| RAD54L2 | Osteoporosis, Postmenopausal | 2.73 |
| RFX3 | Osteoporosis, Postmenopausal | 2.73 |
| RNF115 | Osteoporosis, Postmenopausal | 2.73 |
| RTP3 | Osteoporosis, Postmenopausal | 2.73 |
| SAR1B | Osteoporosis, Postmenopausal | 2.73 |
| SEC63 | Osteoporosis, Postmenopausal | 2.73 |
| SEPHS2 | Osteoporosis, Postmenopausal | 2.73 |
| SLC12A7 | Osteoporosis, Postmenopausal | 2.73 |
| SLC39A7 | Osteoporosis, Postmenopausal | 2.73 |
| SMARCC2 | Osteoporosis, Postmenopausal | 2.73 |
| SMCHD1 | Osteoporosis, Postmenopausal | 2.73 |
| SPIB | Osteoporosis, Postmenopausal | 2.73 |
| SRL | Osteoporosis, Postmenopausal | 2.73 |
| SUZ12 | Osteoporosis, Postmenopausal | 2.73 |
| TAX1BP1 | Osteoporosis, Postmenopausal | 2.73 |
| TDRP | Osteoporosis, Postmenopausal | 2.73 |
| TMEM38A | Osteoporosis, Postmenopausal | 2.73 |
| UBALD2 | Osteoporosis, Postmenopausal | 2.73 |
| VILL | Osteoporosis, Postmenopausal | 2.73 |
| WDTC1 | Osteoporosis, Postmenopausal | 2.73 |
| ZCCHC14 | Osteoporosis, Postmenopausal | 2.73 |
| ZXDC | Osteoporosis, Postmenopausal | 2.73 |
| ACP1 | Osteoporosis, Postmenopausal | 2.72 |
| ACRBP | Osteoporosis, Postmenopausal | 2.72 |
| ATP12A | Osteoporosis, Postmenopausal | 2.72 |
| BEND6 | Osteoporosis, Postmenopausal | 2.72 |
| CCSER2 | Osteoporosis, Postmenopausal | 2.72 |
| CYP4F3 | Osteoporosis, Postmenopausal | 2.72 |
| CYRIB | Osteoporosis, Postmenopausal | 2.72 |
| DDO | Osteoporosis, Postmenopausal | 2.72 |
| DPH2 | Osteoporosis, Postmenopausal | 2.72 |
| ETFRF1 | Osteoporosis, Postmenopausal | 2.72 |
| FAM184A | Osteoporosis, Postmenopausal | 2.72 |
| HSPB2 | Osteoporosis, Postmenopausal | 2.72 |
| IGSF9B | Osteoporosis, Postmenopausal | 2.72 |
| MAF1 | Osteoporosis, Postmenopausal | 2.72 |
| MED12L | Osteoporosis, Postmenopausal | 2.72 |
| MEGF9 | Osteoporosis, Postmenopausal | 2.72 |
| MGAT3 | Osteoporosis, Postmenopausal | 2.72 |
| MKRN1 | Osteoporosis, Postmenopausal | 2.72 |
| MPG | Osteoporosis, Postmenopausal | 2.72 |
| MRPL37 | Osteoporosis, Postmenopausal | 2.72 |
| MT4 | Osteoporosis, Postmenopausal | 2.72 |
| NCAM2 | Osteoporosis, Postmenopausal | 2.72 |
| NIPSNAP1 | Osteoporosis, Postmenopausal | 2.72 |
| PPP1R3G | Osteoporosis, Postmenopausal | 2.72 |
| PREX2 | Osteoporosis, Postmenopausal | 2.72 |
| RAB11FIP5 | Osteoporosis, Postmenopausal | 2.72 |
| RELT | Osteoporosis, Postmenopausal | 2.72 |
| RT1-S3 | Osteoporosis, Postmenopausal | 2.72 |
| RXYLT1 | Osteoporosis, Postmenopausal | 2.72 |
| SCAND1 | Osteoporosis, Postmenopausal | 2.72 |
| SCUBE1 | Osteoporosis, Postmenopausal | 2.72 |
| SIN3B | Osteoporosis, Postmenopausal | 2.72 |
| SORBS3 | Osteoporosis, Postmenopausal | 2.72 |
| SUCNR1 | Osteoporosis, Postmenopausal | 2.72 |
| TBKBP1 | Osteoporosis, Postmenopausal | 2.72 |
| TMEM116 | Osteoporosis, Postmenopausal | 2.72 |
| TRAF3IP3 | Osteoporosis, Postmenopausal | 2.72 |
| TRIM54 | Osteoporosis, Postmenopausal | 2.72 |
| TRIQK | Osteoporosis, Postmenopausal | 2.72 |
| TSHZ3 | Osteoporosis, Postmenopausal | 2.72 |
| TXN1 | Osteoporosis, Postmenopausal | 2.72 |
| VAT1L | Osteoporosis, Postmenopausal | 2.72 |
| VPS18 | Osteoporosis, Postmenopausal | 2.72 |
| WASF2 | Osteoporosis, Postmenopausal | 2.72 |
| XIST | Osteoporosis, Postmenopausal | 2.72 |
| ABHD10 | Osteoporosis, Postmenopausal | 2.71 |
| ACBD3 | Osteoporosis, Postmenopausal | 2.71 |
| AFM | Osteoporosis, Postmenopausal | 2.71 |
| ARMCX2 | Osteoporosis, Postmenopausal | 2.71 |
| ATP11B | Osteoporosis, Postmenopausal | 2.71 |
| BSDC1 | Osteoporosis, Postmenopausal | 2.71 |
| C1QTNF2 | Osteoporosis, Postmenopausal | 2.71 |
| CCDC3 | Osteoporosis, Postmenopausal | 2.71 |
| CCL28 | Osteoporosis, Postmenopausal | 2.71 |
| CCNDBP1 | Osteoporosis, Postmenopausal | 2.71 |
| CEBPE | Osteoporosis, Postmenopausal | 2.71 |
| CETP | Osteoporosis, Postmenopausal | 2.71 |
| CHMP4B | Osteoporosis, Postmenopausal | 2.71 |
| CHST8 | Osteoporosis, Postmenopausal | 2.71 |
| CRELD1 | Osteoporosis, Postmenopausal | 2.71 |
| DAZAP1 | Osteoporosis, Postmenopausal | 2.71 |
| DCAF13 | Osteoporosis, Postmenopausal | 2.71 |
| DENND2A | Osteoporosis, Postmenopausal | 2.71 |
| DTX3 | Osteoporosis, Postmenopausal | 2.71 |
| DYRK3 | Osteoporosis, Postmenopausal | 2.71 |
| ECE2 | Osteoporosis, Postmenopausal | 2.71 |
| EPG5 | Osteoporosis, Postmenopausal | 2.71 |
| EPX | Osteoporosis, Postmenopausal | 2.71 |
| FAXDC2 | Osteoporosis, Postmenopausal | 2.71 |
| FREM2 | Osteoporosis, Postmenopausal | 2.71 |
| GNPDA1 | Osteoporosis, Postmenopausal | 2.71 |
| GOSR2 | Osteoporosis, Postmenopausal | 2.71 |
| GPC5 | Osteoporosis, Postmenopausal | 2.71 |
| GTF2H3 | Osteoporosis, Postmenopausal | 2.71 |
| H2BC9 | Osteoporosis, Postmenopausal | 2.71 |
| HDDC3 | Osteoporosis, Postmenopausal | 2.71 |
| HYAL1 | Osteoporosis, Postmenopausal | 2.71 |
| IAH1 | Osteoporosis, Postmenopausal | 2.71 |
| ICOSLG | Osteoporosis, Postmenopausal | 2.71 |
| IFNAR2 | Osteoporosis, Postmenopausal | 2.71 |
| IFT20 | Osteoporosis, Postmenopausal | 2.71 |
| INHBC | Osteoporosis, Postmenopausal | 2.71 |
| INPP5F | Osteoporosis, Postmenopausal | 2.71 |
| KANK3 | Osteoporosis, Postmenopausal | 2.71 |
| KCNG3 | Osteoporosis, Postmenopausal | 2.71 |
| KCNJ13 | Osteoporosis, Postmenopausal | 2.71 |
| KCNK6 | Osteoporosis, Postmenopausal | 2.71 |
| KLHL23 | Osteoporosis, Postmenopausal | 2.71 |
| KMT2B | Osteoporosis, Postmenopausal | 2.71 |
| KMT5A | Osteoporosis, Postmenopausal | 2.71 |
| LMBR1 | Osteoporosis, Postmenopausal | 2.71 |
| MAB21L3 | Osteoporosis, Postmenopausal | 2.71 |
| MAGEE1 | Osteoporosis, Postmenopausal | 2.71 |
| MATN4 | Osteoporosis, Postmenopausal | 2.71 |
| MCPH1 | Osteoporosis, Postmenopausal | 2.71 |
| MINDY1 | Osteoporosis, Postmenopausal | 2.71 |
| MYLK3 | Osteoporosis, Postmenopausal | 2.71 |
| MYT1 | Osteoporosis, Postmenopausal | 2.71 |
| NIPAL1 | Osteoporosis, Postmenopausal | 2.71 |
| NLRC4 | Osteoporosis, Postmenopausal | 2.71 |
| NUDCD1 | Osteoporosis, Postmenopausal | 2.71 |
| NUP205 | Osteoporosis, Postmenopausal | 2.71 |
| OAZ1 | Osteoporosis, Postmenopausal | 2.71 |
| OPN3 | Osteoporosis, Postmenopausal | 2.71 |
| PDPR | Osteoporosis, Postmenopausal | 2.71 |
| PHF21A | Osteoporosis, Postmenopausal | 2.71 |
| PKD1L2 | Osteoporosis, Postmenopausal | 2.71 |
| PMS1 | Osteoporosis, Postmenopausal | 2.71 |
| PPM1F | Osteoporosis, Postmenopausal | 2.71 |
| PRSS2 | Osteoporosis, Postmenopausal | 2.71 |
| PTCH2 | Osteoporosis, Postmenopausal | 2.71 |
| PYCR3 | Osteoporosis, Postmenopausal | 2.71 |
| RABGGTA | Osteoporosis, Postmenopausal | 2.71 |
| RALGPS1 | Osteoporosis, Postmenopausal | 2.71 |
| RNF38 | Osteoporosis, Postmenopausal | 2.71 |
| ROGDI | Osteoporosis, Postmenopausal | 2.71 |
| RORB | Osteoporosis, Postmenopausal | 2.71 |
| SAMD11 | Osteoporosis, Postmenopausal | 2.71 |
| SCUBE3 | Osteoporosis, Postmenopausal | 2.71 |
| SDF4 | Osteoporosis, Postmenopausal | 2.71 |
| SDK2 | Osteoporosis, Postmenopausal | 2.71 |
| SEMA6B | Osteoporosis, Postmenopausal | 2.71 |
| SERPINA3M | Osteoporosis, Postmenopausal | 2.71 |
| SH2D2A | Osteoporosis, Postmenopausal | 2.71 |
| SHQ1 | Osteoporosis, Postmenopausal | 2.71 |
| SIGIRR | Osteoporosis, Postmenopausal | 2.71 |
| SPRYD7 | Osteoporosis, Postmenopausal | 2.71 |
| STAM | Osteoporosis, Postmenopausal | 2.71 |
| SYNGR2 | Osteoporosis, Postmenopausal | 2.71 |
| TAF15 | Osteoporosis, Postmenopausal | 2.71 |
| TEX10 | Osteoporosis, Postmenopausal | 2.71 |
| THEM4 | Osteoporosis, Postmenopausal | 2.71 |
| TM2D1 | Osteoporosis, Postmenopausal | 2.71 |
| TRHDE | Osteoporosis, Postmenopausal | 2.71 |
| TSPAN9 | Osteoporosis, Postmenopausal | 2.71 |
| TTC23 | Osteoporosis, Postmenopausal | 2.71 |
| ZBTB5 | Osteoporosis, Postmenopausal | 2.71 |
| ZFAND5 | Osteoporosis, Postmenopausal | 2.71 |
| ABCG4 | Osteoporosis, Postmenopausal | 2.7 |
| ADAM23 | Osteoporosis, Postmenopausal | 2.7 |
| ADGRL4 | Osteoporosis, Postmenopausal | 2.7 |
| ANKRD2 | Osteoporosis, Postmenopausal | 2.7 |
| APOL9A | Osteoporosis, Postmenopausal | 2.7 |
| ARHGEF40 | Osteoporosis, Postmenopausal | 2.7 |
| ASCL2 | Osteoporosis, Postmenopausal | 2.7 |
| B3GALT5 | Osteoporosis, Postmenopausal | 2.7 |
| C1RL | Osteoporosis, Postmenopausal | 2.7 |
| COPG2 | Osteoporosis, Postmenopausal | 2.7 |
| COQ10A | Osteoporosis, Postmenopausal | 2.7 |
| CPLX2 | Osteoporosis, Postmenopausal | 2.7 |
| CPNE1 | Osteoporosis, Postmenopausal | 2.7 |
| DACH1 | Osteoporosis, Postmenopausal | 2.7 |
| DCX | Osteoporosis, Postmenopausal | 2.7 |
| DDX46 | Osteoporosis, Postmenopausal | 2.7 |
| DNAJA2 | Osteoporosis, Postmenopausal | 2.7 |
| DYRK1B | Osteoporosis, Postmenopausal | 2.7 |
| DZIP3 | Osteoporosis, Postmenopausal | 2.7 |
| EFS | Osteoporosis, Postmenopausal | 2.7 |
| FXN | Osteoporosis, Postmenopausal | 2.7 |
| GCC2 | Osteoporosis, Postmenopausal | 2.7 |
| GOLGA4 | Osteoporosis, Postmenopausal | 2.7 |
| GSTM7 | Osteoporosis, Postmenopausal | 2.7 |
| GTF3C1 | Osteoporosis, Postmenopausal | 2.7 |
| H2AC25 | Osteoporosis, Postmenopausal | 2.7 |
| HACD3 | Osteoporosis, Postmenopausal | 2.7 |
| HEXA | Osteoporosis, Postmenopausal | 2.7 |
| HNRNPR | Osteoporosis, Postmenopausal | 2.7 |
| KDM5B | Osteoporosis, Postmenopausal | 2.7 |
| LHX8 | Osteoporosis, Postmenopausal | 2.7 |
| LRRC1 | Osteoporosis, Postmenopausal | 2.7 |
| MACROD2 | Osteoporosis, Postmenopausal | 2.7 |
| MBOAT2 | Osteoporosis, Postmenopausal | 2.7 |
| MYOM3 | Osteoporosis, Postmenopausal | 2.7 |
| NDUFA11 | Osteoporosis, Postmenopausal | 2.7 |
| NDUFB11 | Osteoporosis, Postmenopausal | 2.7 |
| NUB1 | Osteoporosis, Postmenopausal | 2.7 |
| NUP98 | Osteoporosis, Postmenopausal | 2.7 |
| PARP4 | Osteoporosis, Postmenopausal | 2.7 |
| PI4K2A | Osteoporosis, Postmenopausal | 2.7 |
| PLA2R1 | Osteoporosis, Postmenopausal | 2.7 |
| PLCL1 | Osteoporosis, Postmenopausal | 2.7 |
| RBM5 | Osteoporosis, Postmenopausal | 2.7 |
| SAA4 | Osteoporosis, Postmenopausal | 2.7 |
| SLC25A42 | Osteoporosis, Postmenopausal | 2.7 |
| SMAD9 | Osteoporosis, Postmenopausal | 2.7 |
| SNAP23 | Osteoporosis, Postmenopausal | 2.7 |
| SRR | Osteoporosis, Postmenopausal | 2.7 |
| SSB | Osteoporosis, Postmenopausal | 2.7 |
| TBCEL | Osteoporosis, Postmenopausal | 2.7 |
| TM9SF2 | Osteoporosis, Postmenopausal | 2.7 |
| UBE2B | Osteoporosis, Postmenopausal | 2.7 |
| VANGL2 | Osteoporosis, Postmenopausal | 2.7 |
| VIPR2 | Osteoporosis, Postmenopausal | 2.7 |
| VOPP1 | Osteoporosis, Postmenopausal | 2.7 |
| ADAP1 | Osteoporosis, Postmenopausal | 2.69 |
| ADH1C | Osteoporosis, Postmenopausal | 2.69 |
| ALDH16A1 | Osteoporosis, Postmenopausal | 2.69 |
| ANGPT4 | Osteoporosis, Postmenopausal | 2.69 |
| ANKRD17 | Osteoporosis, Postmenopausal | 2.69 |
| ARSB | Osteoporosis, Postmenopausal | 2.69 |
| BCKDHB | Osteoporosis, Postmenopausal | 2.69 |
| BLVRB | Osteoporosis, Postmenopausal | 2.69 |
| CAPN8 | Osteoporosis, Postmenopausal | 2.69 |
| CCDC85B | Osteoporosis, Postmenopausal | 2.69 |
| CCR7 | Osteoporosis, Postmenopausal | 2.69 |
| CD5 | Osteoporosis, Postmenopausal | 2.69 |
| CD6 | Osteoporosis, Postmenopausal | 2.69 |
| CGRRF1 | Osteoporosis, Postmenopausal | 2.69 |
| CLCA2 | Osteoporosis, Postmenopausal | 2.69 |
| CNMD | Osteoporosis, Postmenopausal | 2.69 |
| DDI2 | Osteoporosis, Postmenopausal | 2.69 |
| DOP1B | Osteoporosis, Postmenopausal | 2.69 |
| EIF3G | Osteoporosis, Postmenopausal | 2.69 |
| ENTPD4 | Osteoporosis, Postmenopausal | 2.69 |
| FAM78A | Osteoporosis, Postmenopausal | 2.69 |
| FOXK2 | Osteoporosis, Postmenopausal | 2.69 |
| FSD1L | Osteoporosis, Postmenopausal | 2.69 |
| GGTA1 | Osteoporosis, Postmenopausal | 2.69 |
| GUK1 | Osteoporosis, Postmenopausal | 2.69 |
| H4C16 | Osteoporosis, Postmenopausal | 2.69 |
| IFFO2 | Osteoporosis, Postmenopausal | 2.69 |
| IFT74 | Osteoporosis, Postmenopausal | 2.69 |
| KANSL2 | Osteoporosis, Postmenopausal | 2.69 |
| KIZ | Osteoporosis, Postmenopausal | 2.69 |
| KLHDC8B | Osteoporosis, Postmenopausal | 2.69 |
| KLRG1 | Osteoporosis, Postmenopausal | 2.69 |
| LDB1 | Osteoporosis, Postmenopausal | 2.69 |
| LECT2 | Osteoporosis, Postmenopausal | 2.69 |
| LHX2 | Osteoporosis, Postmenopausal | 2.69 |
| LTV1 | Osteoporosis, Postmenopausal | 2.69 |
| MTCH2 | Osteoporosis, Postmenopausal | 2.69 |
| MYO1F | Osteoporosis, Postmenopausal | 2.69 |
| MYRF | Osteoporosis, Postmenopausal | 2.69 |
| NDUFA5 | Osteoporosis, Postmenopausal | 2.69 |
| NOSTRIN | Osteoporosis, Postmenopausal | 2.69 |
| NUP93 | Osteoporosis, Postmenopausal | 2.69 |
| OVOL1 | Osteoporosis, Postmenopausal | 2.69 |
| P4HTM | Osteoporosis, Postmenopausal | 2.69 |
| POLR3H | Osteoporosis, Postmenopausal | 2.69 |
| POPDC3 | Osteoporosis, Postmenopausal | 2.69 |
| PPDPF | Osteoporosis, Postmenopausal | 2.69 |
| PPFIA1 | Osteoporosis, Postmenopausal | 2.69 |
| PPM1A | Osteoporosis, Postmenopausal | 2.69 |
| PRPF19 | Osteoporosis, Postmenopausal | 2.69 |
| PRR15L | Osteoporosis, Postmenopausal | 2.69 |
| PVT1 | Osteoporosis, Postmenopausal | 2.69 |
| RAB1A | Osteoporosis, Postmenopausal | 2.69 |
| RDH10 | Osteoporosis, Postmenopausal | 2.69 |
| RDH11 | Osteoporosis, Postmenopausal | 2.69 |
| REG3G | Osteoporosis, Postmenopausal | 2.69 |
| RHOV | Osteoporosis, Postmenopausal | 2.69 |
| RIOX2 | Osteoporosis, Postmenopausal | 2.69 |
| RSPH1 | Osteoporosis, Postmenopausal | 2.69 |
| RSPH9 | Osteoporosis, Postmenopausal | 2.69 |
| SEPTIN5 | Osteoporosis, Postmenopausal | 2.69 |
| SLC25A23 | Osteoporosis, Postmenopausal | 2.69 |
| SLC35D2 | Osteoporosis, Postmenopausal | 2.69 |
| SMN1 | Osteoporosis, Postmenopausal | 2.69 |
| SSU72 | Osteoporosis, Postmenopausal | 2.69 |
| SURF6 | Osteoporosis, Postmenopausal | 2.69 |
| TBC1D30 | Osteoporosis, Postmenopausal | 2.69 |
| TCF3 | Osteoporosis, Postmenopausal | 2.69 |
| TMEM119 | Osteoporosis, Postmenopausal | 2.69 |
| TMEM150C | Osteoporosis, Postmenopausal | 2.69 |
| TMEM42 | Osteoporosis, Postmenopausal | 2.69 |
| TOM1L2 | Osteoporosis, Postmenopausal | 2.69 |
| TRIP4 | Osteoporosis, Postmenopausal | 2.69 |
| UBE2H | Osteoporosis, Postmenopausal | 2.69 |
| USP12 | Osteoporosis, Postmenopausal | 2.69 |
| USP49 | Osteoporosis, Postmenopausal | 2.69 |
| UTP14A | Osteoporosis, Postmenopausal | 2.69 |
| WASF1 | Osteoporosis, Postmenopausal | 2.69 |
| XYLB | Osteoporosis, Postmenopausal | 2.69 |
| ZMIZ2 | Osteoporosis, Postmenopausal | 2.69 |
| AFAP1L1 | Osteoporosis, Postmenopausal | 2.68 |
| ALDH7A1 | Osteoporosis, Postmenopausal | 2.68 |
| ALOXE3 | Osteoporosis, Postmenopausal | 2.68 |
| BCL2L10 | Osteoporosis, Postmenopausal | 2.68 |
| BLK | Osteoporosis, Postmenopausal | 2.68 |
| BOLA1 | Osteoporosis, Postmenopausal | 2.68 |
| CTLA2A | Osteoporosis, Postmenopausal | 2.68 |
| EEA1 | Osteoporosis, Postmenopausal | 2.68 |
| GAPDHS | Osteoporosis, Postmenopausal | 2.68 |
| GARNL3 | Osteoporosis, Postmenopausal | 2.68 |
| GFER | Osteoporosis, Postmenopausal | 2.68 |
| LRRC8B | Osteoporosis, Postmenopausal | 2.68 |
| M6PR | Osteoporosis, Postmenopausal | 2.68 |
| MTA3 | Osteoporosis, Postmenopausal | 2.68 |
| MTRR | Osteoporosis, Postmenopausal | 2.68 |
| NCKAP1L | Osteoporosis, Postmenopausal | 2.68 |
| NDUFV2 | Osteoporosis, Postmenopausal | 2.68 |
| OPLAH | Osteoporosis, Postmenopausal | 2.68 |
| PES1 | Osteoporosis, Postmenopausal | 2.68 |
| PHC1 | Osteoporosis, Postmenopausal | 2.68 |
| PKP1 | Osteoporosis, Postmenopausal | 2.68 |
| PORCN | Osteoporosis, Postmenopausal | 2.68 |
| PPOX | Osteoporosis, Postmenopausal | 2.68 |
| PRPF38B | Osteoporosis, Postmenopausal | 2.68 |
| PRRC1 | Osteoporosis, Postmenopausal | 2.68 |
| PSMD7 | Osteoporosis, Postmenopausal | 2.68 |
| RABL6 | Osteoporosis, Postmenopausal | 2.68 |
| RMND5A | Osteoporosis, Postmenopausal | 2.68 |
| SEC24A | Osteoporosis, Postmenopausal | 2.68 |
| SH3TC2 | Osteoporosis, Postmenopausal | 2.68 |
| SIK3 | Osteoporosis, Postmenopausal | 2.68 |
| SLC17A3 | Osteoporosis, Postmenopausal | 2.68 |
| SYVN1 | Osteoporosis, Postmenopausal | 2.68 |
| TMEM209 | Osteoporosis, Postmenopausal | 2.68 |
| VEZF1 | Osteoporosis, Postmenopausal | 2.68 |
| ZBTB40 | Osteoporosis, Postmenopausal | 2.68 |
| ZBTB7B | Osteoporosis, Postmenopausal | 2.68 |
| ACTN3 | Osteoporosis, Postmenopausal | 2.67 |
| ADAMTS6 | Osteoporosis, Postmenopausal | 2.67 |
| AGO1 | Osteoporosis, Postmenopausal | 2.67 |
| AIFM3 | Osteoporosis, Postmenopausal | 2.67 |
| ANXA11 | Osteoporosis, Postmenopausal | 2.67 |
| ASAH2 | Osteoporosis, Postmenopausal | 2.67 |
| CACHD1 | Osteoporosis, Postmenopausal | 2.67 |
| CAMSAP1 | Osteoporosis, Postmenopausal | 2.67 |
| CBLC | Osteoporosis, Postmenopausal | 2.67 |
| CES1F | Osteoporosis, Postmenopausal | 2.67 |
| CHID1 | Osteoporosis, Postmenopausal | 2.67 |
| CLEC4D | Osteoporosis, Postmenopausal | 2.67 |
| CMTM8 | Osteoporosis, Postmenopausal | 2.67 |
| CNOT1 | Osteoporosis, Postmenopausal | 2.67 |
| CRYBG2 | Osteoporosis, Postmenopausal | 2.67 |
| CYTH4 | Osteoporosis, Postmenopausal | 2.67 |
| DAPP1 | Osteoporosis, Postmenopausal | 2.67 |
| DARS2 | Osteoporosis, Postmenopausal | 2.67 |
| DDX24 | Osteoporosis, Postmenopausal | 2.67 |
| DLG3 | Osteoporosis, Postmenopausal | 2.67 |
| DLST | Osteoporosis, Postmenopausal | 2.67 |
| EBPL | Osteoporosis, Postmenopausal | 2.67 |
| EFR3B | Osteoporosis, Postmenopausal | 2.67 |
| ESRP2 | Osteoporosis, Postmenopausal | 2.67 |
| FAU | Osteoporosis, Postmenopausal | 2.67 |
| FKBP9 | Osteoporosis, Postmenopausal | 2.67 |
| FZR1 | Osteoporosis, Postmenopausal | 2.67 |
| GFI1 | Osteoporosis, Postmenopausal | 2.67 |
| GKAP1 | Osteoporosis, Postmenopausal | 2.67 |
| GMFB | Osteoporosis, Postmenopausal | 2.67 |
| GPX1A | Osteoporosis, Postmenopausal | 2.67 |
| H2BC4 | Osteoporosis, Postmenopausal | 2.67 |
| HK3 | Osteoporosis, Postmenopausal | 2.67 |
| HLA-C | Osteoporosis, Postmenopausal | 2.67 |
| ICAM5 | Osteoporosis, Postmenopausal | 2.67 |
| IFI27L2B | Osteoporosis, Postmenopausal | 2.67 |
| IL15RA | Osteoporosis, Postmenopausal | 2.67 |
| ISCU | Osteoporosis, Postmenopausal | 2.67 |
| LARGE1 | Osteoporosis, Postmenopausal | 2.67 |
| LARP4 | Osteoporosis, Postmenopausal | 2.67 |
| LDLRAD3 | Osteoporosis, Postmenopausal | 2.67 |
| LRP3 | Osteoporosis, Postmenopausal | 2.67 |
| MAFG | Osteoporosis, Postmenopausal | 2.67 |
| MAML2 | Osteoporosis, Postmenopausal | 2.67 |
| MDGA1 | Osteoporosis, Postmenopausal | 2.67 |
| MPV17L | Osteoporosis, Postmenopausal | 2.67 |
| NAPRT | Osteoporosis, Postmenopausal | 2.67 |
| NAPSA | Osteoporosis, Postmenopausal | 2.67 |
| PBLD | Osteoporosis, Postmenopausal | 2.67 |
| PFDN2 | Osteoporosis, Postmenopausal | 2.67 |
| PHLPP1 | Osteoporosis, Postmenopausal | 2.67 |
| PLAGL2 | Osteoporosis, Postmenopausal | 2.67 |
| PLCB2 | Osteoporosis, Postmenopausal | 2.67 |
| PPFIA3 | Osteoporosis, Postmenopausal | 2.67 |
| PPP1R9B | Osteoporosis, Postmenopausal | 2.67 |
| PRPF39 | Osteoporosis, Postmenopausal | 2.67 |
| PRPS2 | Osteoporosis, Postmenopausal | 2.67 |
| PTS | Osteoporosis, Postmenopausal | 2.67 |
| QSER1 | Osteoporosis, Postmenopausal | 2.67 |
| RAB13 | Osteoporosis, Postmenopausal | 2.67 |
| RAB34 | Osteoporosis, Postmenopausal | 2.67 |
| RAB3C | Osteoporosis, Postmenopausal | 2.67 |
| RASSF8 | Osteoporosis, Postmenopausal | 2.67 |
| RCSD1 | Osteoporosis, Postmenopausal | 2.67 |
| RUSC2 | Osteoporosis, Postmenopausal | 2.67 |
| SDSL | Osteoporosis, Postmenopausal | 2.67 |
| SH2D3C | Osteoporosis, Postmenopausal | 2.67 |
| SRSF10 | Osteoporosis, Postmenopausal | 2.67 |
| STRIP2 | Osteoporosis, Postmenopausal | 2.67 |
| TEAD2 | Osteoporosis, Postmenopausal | 2.67 |
| TMEM154 | Osteoporosis, Postmenopausal | 2.67 |
| TMEM44 | Osteoporosis, Postmenopausal | 2.67 |
| TMEM88 | Osteoporosis, Postmenopausal | 2.67 |
| TNFRSF4 | Osteoporosis, Postmenopausal | 2.67 |
| UBE2V2 | Osteoporosis, Postmenopausal | 2.67 |
| UBR3 | Osteoporosis, Postmenopausal | 2.67 |
| UGT2A1 | Osteoporosis, Postmenopausal | 2.67 |
| USH1C | Osteoporosis, Postmenopausal | 2.67 |
| VASH1 | Osteoporosis, Postmenopausal | 2.67 |
| VWA8 | Osteoporosis, Postmenopausal | 2.67 |
| XPR1 | Osteoporosis, Postmenopausal | 2.67 |
| ACOT12 | Osteoporosis, Postmenopausal | 2.66 |
| AMOT | Osteoporosis, Postmenopausal | 2.66 |
| ARHGAP31 | Osteoporosis, Postmenopausal | 2.66 |
| ARHGEF9 | Osteoporosis, Postmenopausal | 2.66 |
| ASGR2 | Osteoporosis, Postmenopausal | 2.66 |
| ASIC2 | Osteoporosis, Postmenopausal | 2.66 |
| B9D1 | Osteoporosis, Postmenopausal | 2.66 |
| CD2BP2 | Osteoporosis, Postmenopausal | 2.66 |
| CNTF | Osteoporosis, Postmenopausal | 2.66 |
| CORIN | Osteoporosis, Postmenopausal | 2.66 |
| DAGLA | Osteoporosis, Postmenopausal | 2.66 |
| DBT | Osteoporosis, Postmenopausal | 2.66 |
| DCAF8 | Osteoporosis, Postmenopausal | 2.66 |
| DOK3 | Osteoporosis, Postmenopausal | 2.66 |
| EPHA5 | Osteoporosis, Postmenopausal | 2.66 |
| ERCC4 | Osteoporosis, Postmenopausal | 2.66 |
| FAM167A | Osteoporosis, Postmenopausal | 2.66 |
| FBXO21 | Osteoporosis, Postmenopausal | 2.66 |
| GPR37 | Osteoporosis, Postmenopausal | 2.66 |
| GPR68 | Osteoporosis, Postmenopausal | 2.66 |
| GPRIN3 | Osteoporosis, Postmenopausal | 2.66 |
| HYAL2 | Osteoporosis, Postmenopausal | 2.66 |
| IRGM | Osteoporosis, Postmenopausal | 2.66 |
| ISLR | Osteoporosis, Postmenopausal | 2.66 |
| ITM2C | Osteoporosis, Postmenopausal | 2.66 |
| KCNQ5 | Osteoporosis, Postmenopausal | 2.66 |
| KIRREL3 | Osteoporosis, Postmenopausal | 2.66 |
| KRTCAP2 | Osteoporosis, Postmenopausal | 2.66 |
| LAPTM4B | Osteoporosis, Postmenopausal | 2.66 |
| MEGF10 | Osteoporosis, Postmenopausal | 2.66 |
| MYCBP | Osteoporosis, Postmenopausal | 2.66 |
| NENF | Osteoporosis, Postmenopausal | 2.66 |
| NPHP1 | Osteoporosis, Postmenopausal | 2.66 |
| OSBPL5 | Osteoporosis, Postmenopausal | 2.66 |
| PALD1 | Osteoporosis, Postmenopausal | 2.66 |
| PCX | Osteoporosis, Postmenopausal | 2.66 |
| PHLDB3 | Osteoporosis, Postmenopausal | 2.66 |
| PHOSPHO1 | Osteoporosis, Postmenopausal | 2.66 |
| POLR1A | Osteoporosis, Postmenopausal | 2.66 |
| RIOK3 | Osteoporosis, Postmenopausal | 2.66 |
| RSU1 | Osteoporosis, Postmenopausal | 2.66 |
| SLC30A2 | Osteoporosis, Postmenopausal | 2.66 |
| SLCO1B1 | Osteoporosis, Postmenopausal | 2.66 |
| SUSD4 | Osteoporosis, Postmenopausal | 2.66 |
| TAPBP | Osteoporosis, Postmenopausal | 2.66 |
| TEX2 | Osteoporosis, Postmenopausal | 2.66 |
| TIE1 | Osteoporosis, Postmenopausal | 2.66 |
| TMEM14C | Osteoporosis, Postmenopausal | 2.66 |
| TNFAIP1 | Osteoporosis, Postmenopausal | 2.66 |
| TPH2 | Osteoporosis, Postmenopausal | 2.66 |
| UNC45B | Osteoporosis, Postmenopausal | 2.66 |
| VASH2 | Osteoporosis, Postmenopausal | 2.66 |
| ZFR | Osteoporosis, Postmenopausal | 2.66 |
| ADAMTSL2 | Osteoporosis, Postmenopausal | 2.65 |
| AGAP2 | Osteoporosis, Postmenopausal | 2.65 |
| AGXT2 | Osteoporosis, Postmenopausal | 2.65 |
| APOBEC1 | Osteoporosis, Postmenopausal | 2.65 |
| ARCN1 | Osteoporosis, Postmenopausal | 2.65 |
| ARHGAP28 | Osteoporosis, Postmenopausal | 2.65 |
| ARHGAP35 | Osteoporosis, Postmenopausal | 2.65 |
| ARHGEF10 | Osteoporosis, Postmenopausal | 2.65 |
| BCO2 | Osteoporosis, Postmenopausal | 2.65 |
| BET1L | Osteoporosis, Postmenopausal | 2.65 |
| CD84 | Osteoporosis, Postmenopausal | 2.65 |
| CENPT | Osteoporosis, Postmenopausal | 2.65 |
| CIB2 | Osteoporosis, Postmenopausal | 2.65 |
| CLINT1 | Osteoporosis, Postmenopausal | 2.65 |
| DBH | Osteoporosis, Postmenopausal | 2.65 |
| DDX1 | Osteoporosis, Postmenopausal | 2.65 |
| DGKI | Osteoporosis, Postmenopausal | 2.65 |
| DHX37 | Osteoporosis, Postmenopausal | 2.65 |
| DUSP3 | Osteoporosis, Postmenopausal | 2.65 |
| ENPP5 | Osteoporosis, Postmenopausal | 2.65 |
| FAHD1 | Osteoporosis, Postmenopausal | 2.65 |
| FHL3 | Osteoporosis, Postmenopausal | 2.65 |
| FNTB | Osteoporosis, Postmenopausal | 2.65 |
| GRAMD1C | Osteoporosis, Postmenopausal | 2.65 |
| GRM2 | Osteoporosis, Postmenopausal | 2.65 |
| HECA | Osteoporosis, Postmenopausal | 2.65 |
| HLX | Osteoporosis, Postmenopausal | 2.65 |
| HNRNPM | Osteoporosis, Postmenopausal | 2.65 |
| HOMER3 | Osteoporosis, Postmenopausal | 2.65 |
| HOXB3 | Osteoporosis, Postmenopausal | 2.65 |
| IGSF10 | Osteoporosis, Postmenopausal | 2.65 |
| IMMP2L | Osteoporosis, Postmenopausal | 2.65 |
| ITGAE | Osteoporosis, Postmenopausal | 2.65 |
| LANCL1 | Osteoporosis, Postmenopausal | 2.65 |
| LETM1 | Osteoporosis, Postmenopausal | 2.65 |
| LRP11 | Osteoporosis, Postmenopausal | 2.65 |
| LRP12 | Osteoporosis, Postmenopausal | 2.65 |
| MARF1 | Osteoporosis, Postmenopausal | 2.65 |
| METRN | Osteoporosis, Postmenopausal | 2.65 |
| MIR222 | Osteoporosis, Postmenopausal | 2.65 |
| MRPL38 | Osteoporosis, Postmenopausal | 2.65 |
| NDUFA8 | Osteoporosis, Postmenopausal | 2.65 |
| NECAB1 | Osteoporosis, Postmenopausal | 2.65 |
| NSD1 | Osteoporosis, Postmenopausal | 2.65 |
| NSL1 | Osteoporosis, Postmenopausal | 2.65 |
| NXPE3 | Osteoporosis, Postmenopausal | 2.65 |
| OASL2 | Osteoporosis, Postmenopausal | 2.65 |
| OS9 | Osteoporosis, Postmenopausal | 2.65 |
| PAQR4 | Osteoporosis, Postmenopausal | 2.65 |
| PARD3B | Osteoporosis, Postmenopausal | 2.65 |
| PLEKHH2 | Osteoporosis, Postmenopausal | 2.65 |
| PPM1B | Osteoporosis, Postmenopausal | 2.65 |
| PRTN3 | Osteoporosis, Postmenopausal | 2.65 |
| PSMA7 | Osteoporosis, Postmenopausal | 2.65 |
| RAB3A | Osteoporosis, Postmenopausal | 2.65 |
| RBFOX1 | Osteoporosis, Postmenopausal | 2.65 |
| RBPMS2 | Osteoporosis, Postmenopausal | 2.65 |
| RPL5 | Osteoporosis, Postmenopausal | 2.65 |
| SCG3 | Osteoporosis, Postmenopausal | 2.65 |
| SEMA4F | Osteoporosis, Postmenopausal | 2.65 |
| SLAMF7 | Osteoporosis, Postmenopausal | 2.65 |
| SLC29A4 | Osteoporosis, Postmenopausal | 2.65 |
| SPIRE1 | Osteoporosis, Postmenopausal | 2.65 |
| STK3 | Osteoporosis, Postmenopausal | 2.65 |
| STOX2 | Osteoporosis, Postmenopausal | 2.65 |
| TAF13 | Osteoporosis, Postmenopausal | 2.65 |
| THBS3 | Osteoporosis, Postmenopausal | 2.65 |
| TIRAP | Osteoporosis, Postmenopausal | 2.65 |
| TMEM43 | Osteoporosis, Postmenopausal | 2.65 |
| TSEN2 | Osteoporosis, Postmenopausal | 2.65 |
| UQCC2 | Osteoporosis, Postmenopausal | 2.65 |
| UTP4 | Osteoporosis, Postmenopausal | 2.65 |
| VKORC1L1 | Osteoporosis, Postmenopausal | 2.65 |
| VSIR | Osteoporosis, Postmenopausal | 2.65 |
| ACAD9 | Osteoporosis, Postmenopausal | 2.64 |
| AP2S1 | Osteoporosis, Postmenopausal | 2.64 |
| BCL7A | Osteoporosis, Postmenopausal | 2.64 |
| CD19 | Osteoporosis, Postmenopausal | 2.64 |
| CDK19 | Osteoporosis, Postmenopausal | 2.64 |
| CIB1 | Osteoporosis, Postmenopausal | 2.64 |
| CLTC | Osteoporosis, Postmenopausal | 2.64 |
| CTDSP2 | Osteoporosis, Postmenopausal | 2.64 |
| DEXI | Osteoporosis, Postmenopausal | 2.64 |
| DIAPH1 | Osteoporosis, Postmenopausal | 2.64 |
| DIP2C | Osteoporosis, Postmenopausal | 2.64 |
| DNAJC10 | Osteoporosis, Postmenopausal | 2.64 |
| EIF3D | Osteoporosis, Postmenopausal | 2.64 |
| EXPH5 | Osteoporosis, Postmenopausal | 2.64 |
| G6PDX | Osteoporosis, Postmenopausal | 2.64 |
| GLTP | Osteoporosis, Postmenopausal | 2.64 |
| HDHD3 | Osteoporosis, Postmenopausal | 2.64 |
| HEYL | Osteoporosis, Postmenopausal | 2.64 |
| HPDL | Osteoporosis, Postmenopausal | 2.64 |
| ITGA9 | Osteoporosis, Postmenopausal | 2.64 |
| LAMTOR2 | Osteoporosis, Postmenopausal | 2.64 |
| METTL1 | Osteoporosis, Postmenopausal | 2.64 |
| MRPL18 | Osteoporosis, Postmenopausal | 2.64 |
| PIP5K1A | Osteoporosis, Postmenopausal | 2.64 |
| PTPN7 | Osteoporosis, Postmenopausal | 2.64 |
| SPOCK2 | Osteoporosis, Postmenopausal | 2.64 |
| SYMPK | Osteoporosis, Postmenopausal | 2.64 |
| U2AF1 | Osteoporosis, Postmenopausal | 2.64 |
| UNC5C | Osteoporosis, Postmenopausal | 2.64 |
| VAMP2 | Osteoporosis, Postmenopausal | 2.64 |
| VPS37A | Osteoporosis, Postmenopausal | 2.64 |
| ZDHHC14 | Osteoporosis, Postmenopausal | 2.64 |
| ZHX3 | Osteoporosis, Postmenopausal | 2.64 |
| ABCA8 | Osteoporosis, Postmenopausal | 2.63 |
| ACTR3 | Osteoporosis, Postmenopausal | 2.63 |
| ADGRF5 | Osteoporosis, Postmenopausal | 2.63 |
| ANKRD9 | Osteoporosis, Postmenopausal | 2.63 |
| APBB3 | Osteoporosis, Postmenopausal | 2.63 |
| ATP6V1D | Osteoporosis, Postmenopausal | 2.63 |
| C4A | Osteoporosis, Postmenopausal | 2.63 |
| CDC42SE1 | Osteoporosis, Postmenopausal | 2.63 |
| CELF4 | Osteoporosis, Postmenopausal | 2.63 |
| CEPT1 | Osteoporosis, Postmenopausal | 2.63 |
| CLN6 | Osteoporosis, Postmenopausal | 2.63 |
| COX8B | Osteoporosis, Postmenopausal | 2.63 |
| DCHS1 | Osteoporosis, Postmenopausal | 2.63 |
| DENND5A | Osteoporosis, Postmenopausal | 2.63 |
| DMAC2L | Osteoporosis, Postmenopausal | 2.63 |
| DMTN | Osteoporosis, Postmenopausal | 2.63 |
| ELMOD1 | Osteoporosis, Postmenopausal | 2.63 |
| ERG | Osteoporosis, Postmenopausal | 2.63 |
| ESYT2 | Osteoporosis, Postmenopausal | 2.63 |
| FAT3 | Osteoporosis, Postmenopausal | 2.63 |
| FBXL4 | Osteoporosis, Postmenopausal | 2.63 |
| FHOD1 | Osteoporosis, Postmenopausal | 2.63 |
| FYCO1 | Osteoporosis, Postmenopausal | 2.63 |
| GBP6 | Osteoporosis, Postmenopausal | 2.63 |
| GLIS3 | Osteoporosis, Postmenopausal | 2.63 |
| GOLGA2 | Osteoporosis, Postmenopausal | 2.63 |
| HAND2 | Osteoporosis, Postmenopausal | 2.63 |
| HIPK3 | Osteoporosis, Postmenopausal | 2.63 |
| HOGA1 | Osteoporosis, Postmenopausal | 2.63 |
| HPCAL4 | Osteoporosis, Postmenopausal | 2.63 |
| IGSF11 | Osteoporosis, Postmenopausal | 2.63 |
| IL3 | Osteoporosis, Postmenopausal | 2.63 |
| IREB2 | Osteoporosis, Postmenopausal | 2.63 |
| ISCA1 | Osteoporosis, Postmenopausal | 2.63 |
| JPT2 | Osteoporosis, Postmenopausal | 2.63 |
| KCNG1 | Osteoporosis, Postmenopausal | 2.63 |
| KHDRBS3 | Osteoporosis, Postmenopausal | 2.63 |
| LGALS4 | Osteoporosis, Postmenopausal | 2.63 |
| MARK3 | Osteoporosis, Postmenopausal | 2.63 |
| MIA3 | Osteoporosis, Postmenopausal | 2.63 |
| MLXIP | Osteoporosis, Postmenopausal | 2.63 |
| MPP2 | Osteoporosis, Postmenopausal | 2.63 |
| MUC4 | Osteoporosis, Postmenopausal | 2.63 |
| NFKBID | Osteoporosis, Postmenopausal | 2.63 |
| NLGN3 | Osteoporosis, Postmenopausal | 2.63 |
| NLRX1 | Osteoporosis, Postmenopausal | 2.63 |
| OSTC | Osteoporosis, Postmenopausal | 2.63 |
| P3H2 | Osteoporosis, Postmenopausal | 2.63 |
| PDCD6 | Osteoporosis, Postmenopausal | 2.63 |
| PHC3 | Osteoporosis, Postmenopausal | 2.63 |
| PRDM2 | Osteoporosis, Postmenopausal | 2.63 |
| PSMB7 | Osteoporosis, Postmenopausal | 2.63 |
| PTPN22 | Osteoporosis, Postmenopausal | 2.63 |
| RDH16 | Osteoporosis, Postmenopausal | 2.63 |
| RDH5 | Osteoporosis, Postmenopausal | 2.63 |
| RETNLA | Osteoporosis, Postmenopausal | 2.63 |
| RNF152 | Osteoporosis, Postmenopausal | 2.63 |
| RNPS1 | Osteoporosis, Postmenopausal | 2.63 |
| SBNO2 | Osteoporosis, Postmenopausal | 2.63 |
| SCLY | Osteoporosis, Postmenopausal | 2.63 |
| SERTAD4 | Osteoporosis, Postmenopausal | 2.63 |
| SH3GL2 | Osteoporosis, Postmenopausal | 2.63 |
| SIPA1L3 | Osteoporosis, Postmenopausal | 2.63 |
| SKAP1 | Osteoporosis, Postmenopausal | 2.63 |
| SLC29A2 | Osteoporosis, Postmenopausal | 2.63 |
| SLC35B1 | Osteoporosis, Postmenopausal | 2.63 |
| SSR2 | Osteoporosis, Postmenopausal | 2.63 |
| STMN3 | Osteoporosis, Postmenopausal | 2.63 |
| SYCP3 | Osteoporosis, Postmenopausal | 2.63 |
| THSD7A | Osteoporosis, Postmenopausal | 2.63 |
| TMEM229B | Osteoporosis, Postmenopausal | 2.63 |
| TRIM35 | Osteoporosis, Postmenopausal | 2.63 |
| TSPAN2 | Osteoporosis, Postmenopausal | 2.63 |
| TULP4 | Osteoporosis, Postmenopausal | 2.63 |
| UCHL1 | Osteoporosis, Postmenopausal | 2.63 |
| USP3 | Osteoporosis, Postmenopausal | 2.63 |
| WASF3 | Osteoporosis, Postmenopausal | 2.63 |
| WWP1 | Osteoporosis, Postmenopausal | 2.63 |
| XK | Osteoporosis, Postmenopausal | 2.63 |
| ABHD12 | Osteoporosis, Postmenopausal | 2.62 |
| ABLIM2 | Osteoporosis, Postmenopausal | 2.62 |
| ACVR1C | Osteoporosis, Postmenopausal | 2.62 |
| ACYP2 | Osteoporosis, Postmenopausal | 2.62 |
| ADAMTS15 | Osteoporosis, Postmenopausal | 2.62 |
| AGPAT5 | Osteoporosis, Postmenopausal | 2.62 |
| BMS1 | Osteoporosis, Postmenopausal | 2.62 |
| BTC | Osteoporosis, Postmenopausal | 2.62 |
| CALCOCO1 | Osteoporosis, Postmenopausal | 2.62 |
| COL5A3 | Osteoporosis, Postmenopausal | 2.62 |
| CTSF | Osteoporosis, Postmenopausal | 2.62 |
| DCAF11 | Osteoporosis, Postmenopausal | 2.62 |
| DNAJA3 | Osteoporosis, Postmenopausal | 2.62 |
| DNAJC6 | Osteoporosis, Postmenopausal | 2.62 |
| EGFL6 | Osteoporosis, Postmenopausal | 2.62 |
| EPDR1 | Osteoporosis, Postmenopausal | 2.62 |
| FMN1 | Osteoporosis, Postmenopausal | 2.62 |
| FYB1 | Osteoporosis, Postmenopausal | 2.62 |
| GEMIN5 | Osteoporosis, Postmenopausal | 2.62 |
| KMT2E | Osteoporosis, Postmenopausal | 2.62 |
| LAMC3 | Osteoporosis, Postmenopausal | 2.62 |
| LONP1 | Osteoporosis, Postmenopausal | 2.62 |
| LYNX1 | Osteoporosis, Postmenopausal | 2.62 |
| MARK1 | Osteoporosis, Postmenopausal | 2.62 |
| MCOLN3 | Osteoporosis, Postmenopausal | 2.62 |
| MFAP3L | Osteoporosis, Postmenopausal | 2.62 |
| MGARP | Osteoporosis, Postmenopausal | 2.62 |
| MGAT4B | Osteoporosis, Postmenopausal | 2.62 |
| MOB3B | Osteoporosis, Postmenopausal | 2.62 |
| MSRB2 | Osteoporosis, Postmenopausal | 2.62 |
| MVB12B | Osteoporosis, Postmenopausal | 2.62 |
| NCS1 | Osteoporosis, Postmenopausal | 2.62 |
| NEURL1B | Osteoporosis, Postmenopausal | 2.62 |
| PADI4 | Osteoporosis, Postmenopausal | 2.62 |
| PCNX1 | Osteoporosis, Postmenopausal | 2.62 |
| PDLIM4 | Osteoporosis, Postmenopausal | 2.62 |
| PIK3C2G | Osteoporosis, Postmenopausal | 2.62 |
| PTPN18 | Osteoporosis, Postmenopausal | 2.62 |
| RCOR2 | Osteoporosis, Postmenopausal | 2.62 |
| RTKN | Osteoporosis, Postmenopausal | 2.62 |
| SELENOI | Osteoporosis, Postmenopausal | 2.62 |
| SEPTIN6 | Osteoporosis, Postmenopausal | 2.62 |
| SETX | Osteoporosis, Postmenopausal | 2.62 |
| SOWAHC | Osteoporosis, Postmenopausal | 2.62 |
| SP110 | Osteoporosis, Postmenopausal | 2.62 |
| STAC3 | Osteoporosis, Postmenopausal | 2.62 |
| SUMF1 | Osteoporosis, Postmenopausal | 2.62 |
| SYNRG | Osteoporosis, Postmenopausal | 2.62 |
| TBRG4 | Osteoporosis, Postmenopausal | 2.62 |
| TMEM98 | Osteoporosis, Postmenopausal | 2.62 |
| TOMM20 | Osteoporosis, Postmenopausal | 2.62 |
| VKORC1 | Osteoporosis, Postmenopausal | 2.62 |
| WDR46 | Osteoporosis, Postmenopausal | 2.62 |
| WNT3 | Osteoporosis, Postmenopausal | 2.62 |
| XPNPEP1 | Osteoporosis, Postmenopausal | 2.62 |
| ZBTB4 | Osteoporosis, Postmenopausal | 2.62 |
| AFAP1L2 | Osteoporosis, Postmenopausal | 2.61 |
| ANKRD33B | Osteoporosis, Postmenopausal | 2.61 |
| CAMKK1 | Osteoporosis, Postmenopausal | 2.61 |
| CCNL2 | Osteoporosis, Postmenopausal | 2.61 |
| CETN2 | Osteoporosis, Postmenopausal | 2.61 |
| CNKSR3 | Osteoporosis, Postmenopausal | 2.61 |
| CNNM2 | Osteoporosis, Postmenopausal | 2.61 |
| DENND4A | Osteoporosis, Postmenopausal | 2.61 |
| EPB41L4A | Osteoporosis, Postmenopausal | 2.61 |
| FAM89A | Osteoporosis, Postmenopausal | 2.61 |
| HARS1 | Osteoporosis, Postmenopausal | 2.61 |
| MPI | Osteoporosis, Postmenopausal | 2.61 |
| MSRB1 | Osteoporosis, Postmenopausal | 2.61 |
| NHSL1 | Osteoporosis, Postmenopausal | 2.61 |
| NME3 | Osteoporosis, Postmenopausal | 2.61 |
| PDRG1 | Osteoporosis, Postmenopausal | 2.61 |
| PRDX3 | Osteoporosis, Postmenopausal | 2.61 |
| PSMD1 | Osteoporosis, Postmenopausal | 2.61 |
| RBM39 | Osteoporosis, Postmenopausal | 2.61 |
| REXO2 | Osteoporosis, Postmenopausal | 2.61 |
| SECISBP2L | Osteoporosis, Postmenopausal | 2.61 |
| SLC16A9 | Osteoporosis, Postmenopausal | 2.61 |
| SMYD2 | Osteoporosis, Postmenopausal | 2.61 |
| SPART | Osteoporosis, Postmenopausal | 2.61 |
| SPR | Osteoporosis, Postmenopausal | 2.61 |
| TCEAL9 | Osteoporosis, Postmenopausal | 2.61 |
| TEAD1 | Osteoporosis, Postmenopausal | 2.61 |
| ADAP2 | Osteoporosis, Postmenopausal | 2.6 |
| ADCYAP1R1 | Osteoporosis, Postmenopausal | 2.6 |
| ANXA10 | Osteoporosis, Postmenopausal | 2.6 |
| ANXA13 | Osteoporosis, Postmenopausal | 2.6 |
| ARAP3 | Osteoporosis, Postmenopausal | 2.6 |
| ARID3A | Osteoporosis, Postmenopausal | 2.6 |
| ATG10 | Osteoporosis, Postmenopausal | 2.6 |
| C2CD2L | Osteoporosis, Postmenopausal | 2.6 |
| CACNB4 | Osteoporosis, Postmenopausal | 2.6 |
| CCBE1 | Osteoporosis, Postmenopausal | 2.6 |
| CCNI | Osteoporosis, Postmenopausal | 2.6 |
| CCRL2 | Osteoporosis, Postmenopausal | 2.6 |
| CDK18 | Osteoporosis, Postmenopausal | 2.6 |
| CHST2 | Osteoporosis, Postmenopausal | 2.6 |
| CORO7 | Osteoporosis, Postmenopausal | 2.6 |
| DNAJC15 | Osteoporosis, Postmenopausal | 2.6 |
| DTX3L | Osteoporosis, Postmenopausal | 2.6 |
| DYNLL2 | Osteoporosis, Postmenopausal | 2.6 |
| EPHB3 | Osteoporosis, Postmenopausal | 2.6 |
| G3BP2 | Osteoporosis, Postmenopausal | 2.6 |
| GALNT12 | Osteoporosis, Postmenopausal | 2.6 |
| GRIA4 | Osteoporosis, Postmenopausal | 2.6 |
| GRWD1 | Osteoporosis, Postmenopausal | 2.6 |
| GUCY2C | Osteoporosis, Postmenopausal | 2.6 |
| HABP2 | Osteoporosis, Postmenopausal | 2.6 |
| HUNK | Osteoporosis, Postmenopausal | 2.6 |
| IKZF2 | Osteoporosis, Postmenopausal | 2.6 |
| IVD | Osteoporosis, Postmenopausal | 2.6 |
| NCK2 | Osteoporosis, Postmenopausal | 2.6 |
| NDUFS7 | Osteoporosis, Postmenopausal | 2.6 |
| NEURL3 | Osteoporosis, Postmenopausal | 2.6 |
| NPHP4 | Osteoporosis, Postmenopausal | 2.6 |
| OSBP2 | Osteoporosis, Postmenopausal | 2.6 |
| PLCL2 | Osteoporosis, Postmenopausal | 2.6 |
| PLN | Osteoporosis, Postmenopausal | 2.6 |
| PPM1G | Osteoporosis, Postmenopausal | 2.6 |
| PSMC2 | Osteoporosis, Postmenopausal | 2.6 |
| RENBP | Osteoporosis, Postmenopausal | 2.6 |
| RPL35A | Osteoporosis, Postmenopausal | 2.6 |
| SARAF | Osteoporosis, Postmenopausal | 2.6 |
| SERBP1 | Osteoporosis, Postmenopausal | 2.6 |
| SLC30A3 | Osteoporosis, Postmenopausal | 2.6 |
| STARD5 | Osteoporosis, Postmenopausal | 2.6 |
| SUN2 | Osteoporosis, Postmenopausal | 2.6 |
| TAF4B | Osteoporosis, Postmenopausal | 2.6 |
| TENM4 | Osteoporosis, Postmenopausal | 2.6 |
| TRAK1 | Osteoporosis, Postmenopausal | 2.6 |
| UBFD1 | Osteoporosis, Postmenopausal | 2.6 |
| UVRAG | Osteoporosis, Postmenopausal | 2.6 |
| ABCF1 | Osteoporosis, Postmenopausal | 2.59 |
| ACVR2B | Osteoporosis, Postmenopausal | 2.59 |
| ANO6 | Osteoporosis, Postmenopausal | 2.59 |
| APMAP | Osteoporosis, Postmenopausal | 2.59 |
| ARHGEF4 | Osteoporosis, Postmenopausal | 2.59 |
| ARID4B | Osteoporosis, Postmenopausal | 2.59 |
| ATRN | Osteoporosis, Postmenopausal | 2.59 |
| BICC1 | Osteoporosis, Postmenopausal | 2.59 |
| CCL17 | Osteoporosis, Postmenopausal | 2.59 |
| CENPV | Osteoporosis, Postmenopausal | 2.59 |
| CLOCK | Osteoporosis, Postmenopausal | 2.59 |
| CMKLR1 | Osteoporosis, Postmenopausal | 2.59 |
| CRMP1 | Osteoporosis, Postmenopausal | 2.59 |
| CTBP1 | Osteoporosis, Postmenopausal | 2.59 |
| CTNNAL1 | Osteoporosis, Postmenopausal | 2.59 |
| CYP2F2 | Osteoporosis, Postmenopausal | 2.59 |
| DENND2B | Osteoporosis, Postmenopausal | 2.59 |
| DHTKD1 | Osteoporosis, Postmenopausal | 2.59 |
| DNAJC5 | Osteoporosis, Postmenopausal | 2.59 |
| EFHC1 | Osteoporosis, Postmenopausal | 2.59 |
| ELMO1 | Osteoporosis, Postmenopausal | 2.59 |
| FA2H | Osteoporosis, Postmenopausal | 2.59 |
| FGFRL1 | Osteoporosis, Postmenopausal | 2.59 |
| H2BC12 | Osteoporosis, Postmenopausal | 2.59 |
| HAP1 | Osteoporosis, Postmenopausal | 2.59 |
| IDH3G | Osteoporosis, Postmenopausal | 2.59 |
| IL17RA | Osteoporosis, Postmenopausal | 2.59 |
| IL17RD | Osteoporosis, Postmenopausal | 2.59 |
| KAT6A | Osteoporosis, Postmenopausal | 2.59 |
| LARP7 | Osteoporosis, Postmenopausal | 2.59 |
| MAPK8IP3 | Osteoporosis, Postmenopausal | 2.59 |
| NAT10 | Osteoporosis, Postmenopausal | 2.59 |
| NDUFAF4 | Osteoporosis, Postmenopausal | 2.59 |
| NIBAN2 | Osteoporosis, Postmenopausal | 2.59 |
| NPTX2 | Osteoporosis, Postmenopausal | 2.59 |
| NTM | Osteoporosis, Postmenopausal | 2.59 |
| NXN | Osteoporosis, Postmenopausal | 2.59 |
| OCIAD2 | Osteoporosis, Postmenopausal | 2.59 |
| PCBD1 | Osteoporosis, Postmenopausal | 2.59 |
| PCMTD1 | Osteoporosis, Postmenopausal | 2.59 |
| PILRA | Osteoporosis, Postmenopausal | 2.59 |
| PLA2G5 | Osteoporosis, Postmenopausal | 2.59 |
| PURA | Osteoporosis, Postmenopausal | 2.59 |
| RMDN2 | Osteoporosis, Postmenopausal | 2.59 |
| RREB1 | Osteoporosis, Postmenopausal | 2.59 |
| SEC16A | Osteoporosis, Postmenopausal | 2.59 |
| SLC25A15 | Osteoporosis, Postmenopausal | 2.59 |
| SLC48A1 | Osteoporosis, Postmenopausal | 2.59 |
| SPCS3 | Osteoporosis, Postmenopausal | 2.59 |
| SSR4 | Osteoporosis, Postmenopausal | 2.59 |
| TOX | Osteoporosis, Postmenopausal | 2.59 |
| UBE2S | Osteoporosis, Postmenopausal | 2.59 |
| ULK2 | Osteoporosis, Postmenopausal | 2.59 |
| UNKL | Osteoporosis, Postmenopausal | 2.59 |
| WSB2 | Osteoporosis, Postmenopausal | 2.59 |
| YPEL1 | Osteoporosis, Postmenopausal | 2.59 |
| ZBP1 | Osteoporosis, Postmenopausal | 2.59 |
| CD164 | Osteoporosis, Postmenopausal | 2.58 |
| CISD1 | Osteoporosis, Postmenopausal | 2.58 |
| CLIC1 | Osteoporosis, Postmenopausal | 2.58 |
| CRKL | Osteoporosis, Postmenopausal | 2.58 |
| DDR2 | Osteoporosis, Postmenopausal | 2.58 |
| DIXDC1 | Osteoporosis, Postmenopausal | 2.58 |
| DYNC1I1 | Osteoporosis, Postmenopausal | 2.58 |
| EIF4E3 | Osteoporosis, Postmenopausal | 2.58 |
| EML1 | Osteoporosis, Postmenopausal | 2.58 |
| FGGY | Osteoporosis, Postmenopausal | 2.58 |
| GATAD2A | Osteoporosis, Postmenopausal | 2.58 |
| GNRHR | Osteoporosis, Postmenopausal | 2.58 |
| HACD4 | Osteoporosis, Postmenopausal | 2.58 |
| HAO1 | Osteoporosis, Postmenopausal | 2.58 |
| HNRNPA3 | Osteoporosis, Postmenopausal | 2.58 |
| IL6RA | Osteoporosis, Postmenopausal | 2.58 |
| LSM2 | Osteoporosis, Postmenopausal | 2.58 |
| NDST1 | Osteoporosis, Postmenopausal | 2.58 |
| PIEZO1 | Osteoporosis, Postmenopausal | 2.58 |
| PMM1 | Osteoporosis, Postmenopausal | 2.58 |
| PRKRA | Osteoporosis, Postmenopausal | 2.58 |
| RHEB | Osteoporosis, Postmenopausal | 2.58 |
| SFMBT2 | Osteoporosis, Postmenopausal | 2.58 |
| STON2 | Osteoporosis, Postmenopausal | 2.58 |
| TBC1D16 | Osteoporosis, Postmenopausal | 2.58 |
| THNSL2 | Osteoporosis, Postmenopausal | 2.58 |
| VPS37B | Osteoporosis, Postmenopausal | 2.58 |
| ARF6 | Osteoporosis, Postmenopausal | 2.57 |
| ATG7 | Osteoporosis, Postmenopausal | 2.57 |
| BIRC6 | Osteoporosis, Postmenopausal | 2.57 |
| BST1 | Osteoporosis, Postmenopausal | 2.57 |
| BTD | Osteoporosis, Postmenopausal | 2.57 |
| CD99L2 | Osteoporosis, Postmenopausal | 2.57 |
| CHCHD6 | Osteoporosis, Postmenopausal | 2.57 |
| CLIC5 | Osteoporosis, Postmenopausal | 2.57 |
| CLSTN2 | Osteoporosis, Postmenopausal | 2.57 |
| CSF2RA | Osteoporosis, Postmenopausal | 2.57 |
| DOCK7 | Osteoporosis, Postmenopausal | 2.57 |
| DPP3 | Osteoporosis, Postmenopausal | 2.57 |
| EHMT1 | Osteoporosis, Postmenopausal | 2.57 |
| ETFA | Osteoporosis, Postmenopausal | 2.57 |
| FN3K | Osteoporosis, Postmenopausal | 2.57 |
| GNG12 | Osteoporosis, Postmenopausal | 2.57 |
| GRAMD1B | Osteoporosis, Postmenopausal | 2.57 |
| HACD1 | Osteoporosis, Postmenopausal | 2.57 |
| IDE | Osteoporosis, Postmenopausal | 2.57 |
| IFI16 | Osteoporosis, Postmenopausal | 2.57 |
| ING3 | Osteoporosis, Postmenopausal | 2.57 |
| ITM2A | Osteoporosis, Postmenopausal | 2.57 |
| KIF21B | Osteoporosis, Postmenopausal | 2.57 |
| LRRK2 | Osteoporosis, Postmenopausal | 2.57 |
| ND5 | Osteoporosis, Postmenopausal | 2.57 |
| PMM2 | Osteoporosis, Postmenopausal | 2.57 |
| PRCP | Osteoporosis, Postmenopausal | 2.57 |
| PUS7 | Osteoporosis, Postmenopausal | 2.57 |
| RAB27A | Osteoporosis, Postmenopausal | 2.57 |
| RPA1 | Osteoporosis, Postmenopausal | 2.57 |
| RPL17 | Osteoporosis, Postmenopausal | 2.57 |
| RPL36A | Osteoporosis, Postmenopausal | 2.57 |
| SIDT2 | Osteoporosis, Postmenopausal | 2.57 |
| SLC11A1 | Osteoporosis, Postmenopausal | 2.57 |
| SLC24A3 | Osteoporosis, Postmenopausal | 2.57 |
| SLC4A1 | Osteoporosis, Postmenopausal | 2.57 |
| SMPX | Osteoporosis, Postmenopausal | 2.57 |
| SPINT1 | Osteoporosis, Postmenopausal | 2.57 |
| SYNPO2 | Osteoporosis, Postmenopausal | 2.57 |
| SYT11 | Osteoporosis, Postmenopausal | 2.57 |
| TBC1D15 | Osteoporosis, Postmenopausal | 2.57 |
| TMEFF2 | Osteoporosis, Postmenopausal | 2.57 |
| TMEM171 | Osteoporosis, Postmenopausal | 2.57 |
| YWHAH | Osteoporosis, Postmenopausal | 2.57 |
| AAAS | Osteoporosis, Postmenopausal | 2.56 |
| AADAC | Osteoporosis, Postmenopausal | 2.56 |
| ATP6V0C | Osteoporosis, Postmenopausal | 2.56 |
| CARTPT | Osteoporosis, Postmenopausal | 2.56 |
| CCL22 | Osteoporosis, Postmenopausal | 2.56 |
| CHST1 | Osteoporosis, Postmenopausal | 2.56 |
| CLIC3 | Osteoporosis, Postmenopausal | 2.56 |
| COL4A3 | Osteoporosis, Postmenopausal | 2.56 |
| DDB1 | Osteoporosis, Postmenopausal | 2.56 |
| DOT1L | Osteoporosis, Postmenopausal | 2.56 |
| EIF3I | Osteoporosis, Postmenopausal | 2.56 |
| EMILIN1 | Osteoporosis, Postmenopausal | 2.56 |
| EPRS1 | Osteoporosis, Postmenopausal | 2.56 |
| FCGRT | Osteoporosis, Postmenopausal | 2.56 |
| FGF19 | Osteoporosis, Postmenopausal | 2.56 |
| FKTN | Osteoporosis, Postmenopausal | 2.56 |
| FNDC1 | Osteoporosis, Postmenopausal | 2.56 |
| FNIP1 | Osteoporosis, Postmenopausal | 2.56 |
| FUBP1 | Osteoporosis, Postmenopausal | 2.56 |
| GAR1 | Osteoporosis, Postmenopausal | 2.56 |
| GCDH | Osteoporosis, Postmenopausal | 2.56 |
| GDPD1 | Osteoporosis, Postmenopausal | 2.56 |
| H2AZ2 | Osteoporosis, Postmenopausal | 2.56 |
| HIC1 | Osteoporosis, Postmenopausal | 2.56 |
| HIP1R | Osteoporosis, Postmenopausal | 2.56 |
| IGF2BP1 | Osteoporosis, Postmenopausal | 2.56 |
| ITGB7 | Osteoporosis, Postmenopausal | 2.56 |
| LLGL2 | Osteoporosis, Postmenopausal | 2.56 |
| LRRC8C | Osteoporosis, Postmenopausal | 2.56 |
| LRRN3 | Osteoporosis, Postmenopausal | 2.56 |
| NAA38 | Osteoporosis, Postmenopausal | 2.56 |
| NAGA | Osteoporosis, Postmenopausal | 2.56 |
| NDUFB2 | Osteoporosis, Postmenopausal | 2.56 |
| NPTN | Osteoporosis, Postmenopausal | 2.56 |
| PCDH18 | Osteoporosis, Postmenopausal | 2.56 |
| PDGFD | Osteoporosis, Postmenopausal | 2.56 |
| PGLS | Osteoporosis, Postmenopausal | 2.56 |
| POP1 | Osteoporosis, Postmenopausal | 2.56 |
| PPP1R15B | Osteoporosis, Postmenopausal | 2.56 |
| PRKD2 | Osteoporosis, Postmenopausal | 2.56 |
| PRPH | Osteoporosis, Postmenopausal | 2.56 |
| RPS4X | Osteoporosis, Postmenopausal | 2.56 |
| SERTAD2 | Osteoporosis, Postmenopausal | 2.56 |
| SLC13A4 | Osteoporosis, Postmenopausal | 2.56 |
| SLC35F2 | Osteoporosis, Postmenopausal | 2.56 |
| SMARCD2 | Osteoporosis, Postmenopausal | 2.56 |
| TANC2 | Osteoporosis, Postmenopausal | 2.56 |
| TCERG1 | Osteoporosis, Postmenopausal | 2.56 |
| TLCD1 | Osteoporosis, Postmenopausal | 2.56 |
| TNFSF12 | Osteoporosis, Postmenopausal | 2.56 |
| UQCRC1 | Osteoporosis, Postmenopausal | 2.56 |
| WDR6 | Osteoporosis, Postmenopausal | 2.56 |
| ATP11C | Osteoporosis, Postmenopausal | 2.55 |
| BCO1 | Osteoporosis, Postmenopausal | 2.55 |
| CA1 | Osteoporosis, Postmenopausal | 2.55 |
| CCR3 | Osteoporosis, Postmenopausal | 2.55 |
| CDC42EP2 | Osteoporosis, Postmenopausal | 2.55 |
| CDC42EP4 | Osteoporosis, Postmenopausal | 2.55 |
| CYP2A5 | Osteoporosis, Postmenopausal | 2.55 |
| DPYS | Osteoporosis, Postmenopausal | 2.55 |
| ECHDC2 | Osteoporosis, Postmenopausal | 2.55 |
| ELK3 | Osteoporosis, Postmenopausal | 2.55 |
| GJC1 | Osteoporosis, Postmenopausal | 2.55 |
| HCFC1 | Osteoporosis, Postmenopausal | 2.55 |
| HERC5 | Osteoporosis, Postmenopausal | 2.55 |
| HIVEP3 | Osteoporosis, Postmenopausal | 2.55 |
| HUWE1 | Osteoporosis, Postmenopausal | 2.55 |
| IRF2 | Osteoporosis, Postmenopausal | 2.55 |
| JARID2 | Osteoporosis, Postmenopausal | 2.55 |
| KMT2C | Osteoporosis, Postmenopausal | 2.55 |
| LIMS2 | Osteoporosis, Postmenopausal | 2.55 |
| LPAR3 | Osteoporosis, Postmenopausal | 2.55 |
| LY86 | Osteoporosis, Postmenopausal | 2.55 |
| MEOX2 | Osteoporosis, Postmenopausal | 2.55 |
| MIPEP | Osteoporosis, Postmenopausal | 2.55 |
| NRBP2 | Osteoporosis, Postmenopausal | 2.55 |
| NSG1 | Osteoporosis, Postmenopausal | 2.55 |
| OMD | Osteoporosis, Postmenopausal | 2.55 |
| P2RY6 | Osteoporosis, Postmenopausal | 2.55 |
| PABPN1 | Osteoporosis, Postmenopausal | 2.55 |
| PBXIP1 | Osteoporosis, Postmenopausal | 2.55 |
| PKIA | Osteoporosis, Postmenopausal | 2.55 |
| POLR1B | Osteoporosis, Postmenopausal | 2.55 |
| POU2F2 | Osteoporosis, Postmenopausal | 2.55 |
| PPIL1 | Osteoporosis, Postmenopausal | 2.55 |
| PSMA5 | Osteoporosis, Postmenopausal | 2.55 |
| PTPRJ | Osteoporosis, Postmenopausal | 2.55 |
| RCC1 | Osteoporosis, Postmenopausal | 2.55 |
| REEP1 | Osteoporosis, Postmenopausal | 2.55 |
| RPL37A | Osteoporosis, Postmenopausal | 2.55 |
| RPS17 | Osteoporosis, Postmenopausal | 2.55 |
| SLC12A3 | Osteoporosis, Postmenopausal | 2.55 |
| SLC12A8 | Osteoporosis, Postmenopausal | 2.55 |
| SLC14A1 | Osteoporosis, Postmenopausal | 2.55 |
| SLC25A36 | Osteoporosis, Postmenopausal | 2.55 |
| SLC44A2 | Osteoporosis, Postmenopausal | 2.55 |
| TSPAN7 | Osteoporosis, Postmenopausal | 2.55 |
| TTLL7 | Osteoporosis, Postmenopausal | 2.55 |
| UPP2 | Osteoporosis, Postmenopausal | 2.55 |
| UQCRFS1 | Osteoporosis, Postmenopausal | 2.55 |
| UQCRQ | Osteoporosis, Postmenopausal | 2.55 |
| ABCA2 | Osteoporosis, Postmenopausal | 2.54 |
| ANP32B | Osteoporosis, Postmenopausal | 2.54 |
| BACH2 | Osteoporosis, Postmenopausal | 2.54 |
| CCT2 | Osteoporosis, Postmenopausal | 2.54 |
| EHD3 | Osteoporosis, Postmenopausal | 2.54 |
| ETF1 | Osteoporosis, Postmenopausal | 2.54 |
| FARSA | Osteoporosis, Postmenopausal | 2.54 |
| FAT4 | Osteoporosis, Postmenopausal | 2.54 |
| FCGR3 | Osteoporosis, Postmenopausal | 2.54 |
| FMOD | Osteoporosis, Postmenopausal | 2.54 |
| HEBP1 | Osteoporosis, Postmenopausal | 2.54 |
| HERC6 | Osteoporosis, Postmenopausal | 2.54 |
| ICA1 | Osteoporosis, Postmenopausal | 2.54 |
| LRPAP1 | Osteoporosis, Postmenopausal | 2.54 |
| NECTIN3 | Osteoporosis, Postmenopausal | 2.54 |
| PSME2 | Osteoporosis, Postmenopausal | 2.54 |
| RGMA | Osteoporosis, Postmenopausal | 2.54 |
| SAMD9L | Osteoporosis, Postmenopausal | 2.54 |
| SERINC3 | Osteoporosis, Postmenopausal | 2.54 |
| SKI | Osteoporosis, Postmenopausal | 2.54 |
| SLC43A2 | Osteoporosis, Postmenopausal | 2.54 |
| TNRC6A | Osteoporosis, Postmenopausal | 2.54 |
| TPPP3 | Osteoporosis, Postmenopausal | 2.54 |
| TRAFD1 | Osteoporosis, Postmenopausal | 2.54 |
| TXNDC5 | Osteoporosis, Postmenopausal | 2.54 |
| ADCY3 | Osteoporosis, Postmenopausal | 2.53 |
| ATP11A | Osteoporosis, Postmenopausal | 2.53 |
| ATP5PF | Osteoporosis, Postmenopausal | 2.53 |
| BZW2 | Osteoporosis, Postmenopausal | 2.53 |
| CD70 | Osteoporosis, Postmenopausal | 2.53 |
| CLMP | Osteoporosis, Postmenopausal | 2.53 |
| EBI3 | Osteoporosis, Postmenopausal | 2.53 |
| GRAMD2B | Osteoporosis, Postmenopausal | 2.53 |
| GRIK2 | Osteoporosis, Postmenopausal | 2.53 |
| IGTP | Osteoporosis, Postmenopausal | 2.53 |
| KAT6B | Osteoporosis, Postmenopausal | 2.53 |
| MCC | Osteoporosis, Postmenopausal | 2.53 |
| MED13L | Osteoporosis, Postmenopausal | 2.53 |
| MPP1 | Osteoporosis, Postmenopausal | 2.53 |
| NDUFB6 | Osteoporosis, Postmenopausal | 2.53 |
| PNPLA7 | Osteoporosis, Postmenopausal | 2.53 |
| PPP1R14A | Osteoporosis, Postmenopausal | 2.53 |
| PPP5C | Osteoporosis, Postmenopausal | 2.53 |
| PTBP3 | Osteoporosis, Postmenopausal | 2.53 |
| RBX1 | Osteoporosis, Postmenopausal | 2.53 |
| RPL14 | Osteoporosis, Postmenopausal | 2.53 |
| RPL9 | Osteoporosis, Postmenopausal | 2.53 |
| RTN4RL1 | Osteoporosis, Postmenopausal | 2.53 |
| SEC23A | Osteoporosis, Postmenopausal | 2.53 |
| SLC2A6 | Osteoporosis, Postmenopausal | 2.53 |
| TLE4 | Osteoporosis, Postmenopausal | 2.53 |
| UBE4B | Osteoporosis, Postmenopausal | 2.53 |
| VARS1 | Osteoporosis, Postmenopausal | 2.53 |
| ACP2 | Osteoporosis, Postmenopausal | 2.52 |
| ADAR | Osteoporosis, Postmenopausal | 2.52 |
| AVPI1 | Osteoporosis, Postmenopausal | 2.52 |
| BAIAP2L1 | Osteoporosis, Postmenopausal | 2.52 |
| CSNK1E | Osteoporosis, Postmenopausal | 2.52 |
| DDX6 | Osteoporosis, Postmenopausal | 2.52 |
| FLI1 | Osteoporosis, Postmenopausal | 2.52 |
| GGCT | Osteoporosis, Postmenopausal | 2.52 |
| GLI3 | Osteoporosis, Postmenopausal | 2.52 |
| GNG2 | Osteoporosis, Postmenopausal | 2.52 |
| GPC4 | Osteoporosis, Postmenopausal | 2.52 |
| HELZ2 | Osteoporosis, Postmenopausal | 2.52 |
| HIP1 | Osteoporosis, Postmenopausal | 2.52 |
| HTRA3 | Osteoporosis, Postmenopausal | 2.52 |
| IFI47 | Osteoporosis, Postmenopausal | 2.52 |
| IRF5 | Osteoporosis, Postmenopausal | 2.52 |
| KIF1A | Osteoporosis, Postmenopausal | 2.52 |
| MOXD1 | Osteoporosis, Postmenopausal | 2.52 |
| NIT1 | Osteoporosis, Postmenopausal | 2.52 |
| PGRMC2 | Osteoporosis, Postmenopausal | 2.52 |
| PIP4K2A | Osteoporosis, Postmenopausal | 2.52 |
| PLA2G4C | Osteoporosis, Postmenopausal | 2.52 |
| PPP2R2A | Osteoporosis, Postmenopausal | 2.52 |
| PRUNE2 | Osteoporosis, Postmenopausal | 2.52 |
| RCN3 | Osteoporosis, Postmenopausal | 2.52 |
| RPL36 | Osteoporosis, Postmenopausal | 2.52 |
| SEMA4D | Osteoporosis, Postmenopausal | 2.52 |
| SLC25A3 | Osteoporosis, Postmenopausal | 2.52 |
| SLC25A33 | Osteoporosis, Postmenopausal | 2.52 |
| SLC26A4 | Osteoporosis, Postmenopausal | 2.52 |
| SLC6A15 | Osteoporosis, Postmenopausal | 2.52 |
| THOP1 | Osteoporosis, Postmenopausal | 2.52 |
| WIF1 | Osteoporosis, Postmenopausal | 2.52 |
| ADCY1 | Osteoporosis, Postmenopausal | 2.51 |
| AKAP8L | Osteoporosis, Postmenopausal | 2.51 |
| AKR1C4 | Osteoporosis, Postmenopausal | 2.51 |
| ANGPTL3 | Osteoporosis, Postmenopausal | 2.51 |
| C7 | Osteoporosis, Postmenopausal | 2.51 |
| CCL19 | Osteoporosis, Postmenopausal | 2.51 |
| CCR1 | Osteoporosis, Postmenopausal | 2.51 |
| CD22 | Osteoporosis, Postmenopausal | 2.51 |
| CD47 | Osteoporosis, Postmenopausal | 2.51 |
| DAAM1 | Osteoporosis, Postmenopausal | 2.51 |
| DCTPP1 | Osteoporosis, Postmenopausal | 2.51 |
| DDT | Osteoporosis, Postmenopausal | 2.51 |
| EVL | Osteoporosis, Postmenopausal | 2.51 |
| KTN1 | Osteoporosis, Postmenopausal | 2.51 |
| MTDH | Osteoporosis, Postmenopausal | 2.51 |
| PCM1 | Osteoporosis, Postmenopausal | 2.51 |
| PDE9A | Osteoporosis, Postmenopausal | 2.51 |
| PLEK2 | Osteoporosis, Postmenopausal | 2.51 |
| POLG | Osteoporosis, Postmenopausal | 2.51 |
| PTPRS | Osteoporosis, Postmenopausal | 2.51 |
| RBMS3 | Osteoporosis, Postmenopausal | 2.51 |
| RPL27 | Osteoporosis, Postmenopausal | 2.51 |
| SEMA6A | Osteoporosis, Postmenopausal | 2.51 |
| SPRED1 | Osteoporosis, Postmenopausal | 2.51 |
| TANK | Osteoporosis, Postmenopausal | 2.51 |
| UBQLN1 | Osteoporosis, Postmenopausal | 2.51 |
| CALML4 | Osteoporosis, Postmenopausal | 2.5 |
| DDX39B | Osteoporosis, Postmenopausal | 2.5 |
| EIF5B | Osteoporosis, Postmenopausal | 2.5 |
| FLT3 | Osteoporosis, Postmenopausal | 2.5 |
| FZD8 | Osteoporosis, Postmenopausal | 2.5 |
| GNAT1 | Osteoporosis, Postmenopausal | 2.5 |
| KHK | Osteoporosis, Postmenopausal | 2.5 |
| KLF7 | Osteoporosis, Postmenopausal | 2.5 |
| LRBA | Osteoporosis, Postmenopausal | 2.5 |
| LUC7L3 | Osteoporosis, Postmenopausal | 2.5 |
| LYZ2 | Osteoporosis, Postmenopausal | 2.5 |
| MAGED2 | Osteoporosis, Postmenopausal | 2.5 |
| MRAS | Osteoporosis, Postmenopausal | 2.5 |
| PDE7A | Osteoporosis, Postmenopausal | 2.5 |
| PEMT | Osteoporosis, Postmenopausal | 2.5 |
| PSME3 | Osteoporosis, Postmenopausal | 2.5 |
| RPL11 | Osteoporosis, Postmenopausal | 2.5 |
| SEPTIN9 | Osteoporosis, Postmenopausal | 2.5 |
| TLR9 | Osteoporosis, Postmenopausal | 2.5 |
| TOR3A | Osteoporosis, Postmenopausal | 2.5 |
| UBA52 | Osteoporosis, Postmenopausal | 2.5 |
| ZC3H12A | Osteoporosis, Postmenopausal | 2.5 |
| AHNAK2 | Osteoporosis, Postmenopausal | 2.49 |
| AKR1B7 | Osteoporosis, Postmenopausal | 2.49 |
| ARHGAP24 | Osteoporosis, Postmenopausal | 2.49 |
| ARL6IP1 | Osteoporosis, Postmenopausal | 2.49 |
| ASPA | Osteoporosis, Postmenopausal | 2.49 |
| BBX | Osteoporosis, Postmenopausal | 2.49 |
| CGREF1 | Osteoporosis, Postmenopausal | 2.49 |
| CHRDL1 | Osteoporosis, Postmenopausal | 2.49 |
| CIITA | Osteoporosis, Postmenopausal | 2.49 |
| EML4 | Osteoporosis, Postmenopausal | 2.49 |
| ERAP1 | Osteoporosis, Postmenopausal | 2.49 |
| FLOT2 | Osteoporosis, Postmenopausal | 2.49 |
| GABRB2 | Osteoporosis, Postmenopausal | 2.49 |
| GBP4 | Osteoporosis, Postmenopausal | 2.49 |
| KRT4 | Osteoporosis, Postmenopausal | 2.49 |
| LGALSL | Osteoporosis, Postmenopausal | 2.49 |
| LRP6 | Osteoporosis, Postmenopausal | 2.49 |
| LURAP1L | Osteoporosis, Postmenopausal | 2.49 |
| MDM4 | Osteoporosis, Postmenopausal | 2.49 |
| MOV10 | Osteoporosis, Postmenopausal | 2.49 |
| MRC2 | Osteoporosis, Postmenopausal | 2.49 |
| NPL | Osteoporosis, Postmenopausal | 2.49 |
| PCYT2 | Osteoporosis, Postmenopausal | 2.49 |
| PREP | Osteoporosis, Postmenopausal | 2.49 |
| PRRX1 | Osteoporosis, Postmenopausal | 2.49 |
| RPL12 | Osteoporosis, Postmenopausal | 2.49 |
| RPS27A | Osteoporosis, Postmenopausal | 2.49 |
| SEPTIN4 | Osteoporosis, Postmenopausal | 2.49 |
| SNRPD1 | Osteoporosis, Postmenopausal | 2.49 |
| SPRED2 | Osteoporosis, Postmenopausal | 2.49 |
| THBS4 | Osteoporosis, Postmenopausal | 2.49 |
| TTPA | Osteoporosis, Postmenopausal | 2.49 |
| VAMP8 | Osteoporosis, Postmenopausal | 2.49 |
| VEPH1 | Osteoporosis, Postmenopausal | 2.49 |
| ADCY6 | Osteoporosis, Postmenopausal | 2.48 |
| CPOX | Osteoporosis, Postmenopausal | 2.48 |
| CXXC5 | Osteoporosis, Postmenopausal | 2.48 |
| EIF3C | Osteoporosis, Postmenopausal | 2.48 |
| EXT1 | Osteoporosis, Postmenopausal | 2.48 |
| FBP2 | Osteoporosis, Postmenopausal | 2.48 |
| FRMD4A | Osteoporosis, Postmenopausal | 2.48 |
| GALNT2 | Osteoporosis, Postmenopausal | 2.48 |
| GM2A | Osteoporosis, Postmenopausal | 2.48 |
| GNA14 | Osteoporosis, Postmenopausal | 2.48 |
| GNE | Osteoporosis, Postmenopausal | 2.48 |
| JAM2 | Osteoporosis, Postmenopausal | 2.48 |
| MYO1E | Osteoporosis, Postmenopausal | 2.48 |
| OGN | Osteoporosis, Postmenopausal | 2.48 |
| PPA1 | Osteoporosis, Postmenopausal | 2.48 |
| RPL24 | Osteoporosis, Postmenopausal | 2.48 |
| RPS13 | Osteoporosis, Postmenopausal | 2.48 |
| RPS14 | Osteoporosis, Postmenopausal | 2.48 |
| SEMA7A | Osteoporosis, Postmenopausal | 2.48 |
| SLCO3A1 | Osteoporosis, Postmenopausal | 2.48 |
| SMOC2 | Osteoporosis, Postmenopausal | 2.48 |
| STK10 | Osteoporosis, Postmenopausal | 2.48 |
| UPB1 | Osteoporosis, Postmenopausal | 2.48 |
| ABL2 | Osteoporosis, Postmenopausal | 2.47 |
| ATP5F1D | Osteoporosis, Postmenopausal | 2.47 |
| CSF2RB | Osteoporosis, Postmenopausal | 2.47 |
| EFNB1 | Osteoporosis, Postmenopausal | 2.47 |
| FARSB | Osteoporosis, Postmenopausal | 2.47 |
| HMGB3 | Osteoporosis, Postmenopausal | 2.47 |
| IFI44L | Osteoporosis, Postmenopausal | 2.47 |
| KLF12 | Osteoporosis, Postmenopausal | 2.47 |
| MAN2A1 | Osteoporosis, Postmenopausal | 2.47 |
| MATN2 | Osteoporosis, Postmenopausal | 2.47 |
| NR0B1 | Osteoporosis, Postmenopausal | 2.47 |
| OSM | Osteoporosis, Postmenopausal | 2.47 |
| PCP4L1 | Osteoporosis, Postmenopausal | 2.47 |
| PDE1A | Osteoporosis, Postmenopausal | 2.47 |
| PIGA | Osteoporosis, Postmenopausal | 2.47 |
| PTPRO | Osteoporosis, Postmenopausal | 2.47 |
| RBM14 | Osteoporosis, Postmenopausal | 2.47 |
| RPL18 | Osteoporosis, Postmenopausal | 2.47 |
| RTP4 | Osteoporosis, Postmenopausal | 2.47 |
| SCP2 | Osteoporosis, Postmenopausal | 2.47 |
| SEMA5A | Osteoporosis, Postmenopausal | 2.47 |
| STMN2 | Osteoporosis, Postmenopausal | 2.47 |
| TLN2 | Osteoporosis, Postmenopausal | 2.47 |
| ATP1A3 | Osteoporosis, Postmenopausal | 2.46 |
| BMPER | Osteoporosis, Postmenopausal | 2.46 |
| CHPT1 | Osteoporosis, Postmenopausal | 2.46 |
| CRY2 | Osteoporosis, Postmenopausal | 2.46 |
| ETV1 | Osteoporosis, Postmenopausal | 2.46 |
| FEZ1 | Osteoporosis, Postmenopausal | 2.46 |
| ITIH4 | Osteoporosis, Postmenopausal | 2.46 |
| NTN1 | Osteoporosis, Postmenopausal | 2.46 |
| PCYOX1 | Osteoporosis, Postmenopausal | 2.46 |
| PICALM | Osteoporosis, Postmenopausal | 2.46 |
| PON3 | Osteoporosis, Postmenopausal | 2.46 |
| RPS12 | Osteoporosis, Postmenopausal | 2.46 |
| SATB1 | Osteoporosis, Postmenopausal | 2.46 |
| SGK2 | Osteoporosis, Postmenopausal | 2.46 |
| STRA6 | Osteoporosis, Postmenopausal | 2.46 |
| ARHGAP26 | Osteoporosis, Postmenopausal | 2.45 |
| CALM3 | Osteoporosis, Postmenopausal | 2.45 |
| CCT7 | Osteoporosis, Postmenopausal | 2.45 |
| CDC14B | Osteoporosis, Postmenopausal | 2.45 |
| CHRNB4 | Osteoporosis, Postmenopausal | 2.45 |
| DHX58 | Osteoporosis, Postmenopausal | 2.45 |
| EPHB2 | Osteoporosis, Postmenopausal | 2.45 |
| GRM5 | Osteoporosis, Postmenopausal | 2.45 |
| KCNJ2 | Osteoporosis, Postmenopausal | 2.45 |
| MBL2 | Osteoporosis, Postmenopausal | 2.45 |
| MEF2D | Osteoporosis, Postmenopausal | 2.45 |
| MEST | Osteoporosis, Postmenopausal | 2.45 |
| MYADM | Osteoporosis, Postmenopausal | 2.45 |
| NEK6 | Osteoporosis, Postmenopausal | 2.45 |
| NOG | Osteoporosis, Postmenopausal | 2.45 |
| RBM47 | Osteoporosis, Postmenopausal | 2.45 |
| RPL22 | Osteoporosis, Postmenopausal | 2.45 |
| TCEA3 | Osteoporosis, Postmenopausal | 2.45 |
| TCN2 | Osteoporosis, Postmenopausal | 2.45 |
| TFEB | Osteoporosis, Postmenopausal | 2.45 |
| TLN1 | Osteoporosis, Postmenopausal | 2.45 |
| TLR6 | Osteoporosis, Postmenopausal | 2.45 |
| ABCE1 | Osteoporosis, Postmenopausal | 2.44 |
| ALDH3B1 | Osteoporosis, Postmenopausal | 2.44 |
| COX17 | Osteoporosis, Postmenopausal | 2.44 |
| GNL3 | Osteoporosis, Postmenopausal | 2.44 |
| GTF2I | Osteoporosis, Postmenopausal | 2.44 |
| H6PD | Osteoporosis, Postmenopausal | 2.44 |
| ITGA8 | Osteoporosis, Postmenopausal | 2.44 |
| KCNJ8 | Osteoporosis, Postmenopausal | 2.44 |
| PDE4A | Osteoporosis, Postmenopausal | 2.44 |
| PLCE1 | Osteoporosis, Postmenopausal | 2.44 |
| RPS15 | Osteoporosis, Postmenopausal | 2.44 |
| SERPINA3N | Osteoporosis, Postmenopausal | 2.44 |
| WWOX | Osteoporosis, Postmenopausal | 2.44 |
| BCL11A | Osteoporosis, Postmenopausal | 2.43 |
| COX4I1 | Osteoporosis, Postmenopausal | 2.43 |
| EBF1 | Osteoporosis, Postmenopausal | 2.43 |
| EDEM1 | Osteoporosis, Postmenopausal | 2.43 |
| LAMA4 | Osteoporosis, Postmenopausal | 2.43 |
| LRRC59 | Osteoporosis, Postmenopausal | 2.43 |
| MCF2L | Osteoporosis, Postmenopausal | 2.43 |
| NPTX1 | Osteoporosis, Postmenopausal | 2.43 |
| PDE10A | Osteoporosis, Postmenopausal | 2.43 |
| PIK3C2A | Osteoporosis, Postmenopausal | 2.43 |
| PLAC8 | Osteoporosis, Postmenopausal | 2.43 |
| PRICKLE1 | Osteoporosis, Postmenopausal | 2.43 |
| PSMB2 | Osteoporosis, Postmenopausal | 2.43 |
| RPL13A | Osteoporosis, Postmenopausal | 2.43 |
| RPL7 | Osteoporosis, Postmenopausal | 2.43 |
| RUNX3 | Osteoporosis, Postmenopausal | 2.43 |
| SFXN1 | Osteoporosis, Postmenopausal | 2.43 |
| SGMS1 | Osteoporosis, Postmenopausal | 2.43 |
| ACOT2 | Osteoporosis, Postmenopausal | 2.42 |
| ADAMTS5 | Osteoporosis, Postmenopausal | 2.42 |
| AIFM2 | Osteoporosis, Postmenopausal | 2.42 |
| EEF1D | Osteoporosis, Postmenopausal | 2.42 |
| EEF1G | Osteoporosis, Postmenopausal | 2.42 |
| EHMT2 | Osteoporosis, Postmenopausal | 2.42 |
| FBL | Osteoporosis, Postmenopausal | 2.42 |
| IFITM2 | Osteoporosis, Postmenopausal | 2.42 |
| RPS15A | Osteoporosis, Postmenopausal | 2.42 |
| RPS27 | Osteoporosis, Postmenopausal | 2.42 |
| RPS7 | Osteoporosis, Postmenopausal | 2.42 |
| SLCO1B2 | Osteoporosis, Postmenopausal | 2.42 |
| TEF | Osteoporosis, Postmenopausal | 2.42 |
| AKAP13 | Osteoporosis, Postmenopausal | 2.41 |
| AS3MT | Osteoporosis, Postmenopausal | 2.41 |
| C1R | Osteoporosis, Postmenopausal | 2.41 |
| FABP2 | Osteoporosis, Postmenopausal | 2.41 |
| GNAQ | Osteoporosis, Postmenopausal | 2.41 |
| MAP1LC3A | Osteoporosis, Postmenopausal | 2.41 |
| RARG | Osteoporosis, Postmenopausal | 2.41 |
| RPLP0 | Osteoporosis, Postmenopausal | 2.41 |
| CCT6A | Osteoporosis, Postmenopausal | 2.4 |
| CYFIP2 | Osteoporosis, Postmenopausal | 2.4 |
| GUCY1B1 | Osteoporosis, Postmenopausal | 2.4 |
| KCNQ1 | Osteoporosis, Postmenopausal | 2.4 |
| NR1H2 | Osteoporosis, Postmenopausal | 2.4 |
| PDIA6 | Osteoporosis, Postmenopausal | 2.4 |
| PLA2G12A | Osteoporosis, Postmenopausal | 2.4 |
| PPRC1 | Osteoporosis, Postmenopausal | 2.4 |
| PSMB3 | Osteoporosis, Postmenopausal | 2.4 |
| RAPGEF4 | Osteoporosis, Postmenopausal | 2.4 |
| RPL31 | Osteoporosis, Postmenopausal | 2.4 |
| SLC37A4 | Osteoporosis, Postmenopausal | 2.4 |
| AMBP | Osteoporosis, Postmenopausal | 2.39 |
| CREG1 | Osteoporosis, Postmenopausal | 2.39 |
| DHRS9 | Osteoporosis, Postmenopausal | 2.39 |
| GABARAPL1 | Osteoporosis, Postmenopausal | 2.39 |
| GAD2 | Osteoporosis, Postmenopausal | 2.39 |
| GALE | Osteoporosis, Postmenopausal | 2.39 |
| JMJD6 | Osteoporosis, Postmenopausal | 2.39 |
| MT1F | Osteoporosis, Postmenopausal | 2.39 |
| NEU1 | Osteoporosis, Postmenopausal | 2.39 |
| NID1 | Osteoporosis, Postmenopausal | 2.39 |
| PFKL | Osteoporosis, Postmenopausal | 2.39 |
| CCT3 | Osteoporosis, Postmenopausal | 2.38 |
| OPA1 | Osteoporosis, Postmenopausal | 2.38 |
| PVR | Osteoporosis, Postmenopausal | 2.38 |
| RBPMS | Osteoporosis, Postmenopausal | 2.38 |
| RPS3 | Osteoporosis, Postmenopausal | 2.38 |
| SLC19A2 | Osteoporosis, Postmenopausal | 2.38 |
| SPON1 | Osteoporosis, Postmenopausal | 2.38 |
| ABCB6 | Osteoporosis, Postmenopausal | 2.37 |
| ADK | Osteoporosis, Postmenopausal | 2.37 |
| ALDH1L1 | Osteoporosis, Postmenopausal | 2.37 |
| ATXN1 | Osteoporosis, Postmenopausal | 2.37 |
| INPP5D | Osteoporosis, Postmenopausal | 2.37 |
| SLC25A25 | Osteoporosis, Postmenopausal | 2.37 |
| ZFP36L2 | Osteoporosis, Postmenopausal | 2.37 |
| CAST | Osteoporosis, Postmenopausal | 2.36 |
| CRAT | Osteoporosis, Postmenopausal | 2.36 |
| FSCN1 | Osteoporosis, Postmenopausal | 2.36 |
| LOXL2 | Osteoporosis, Postmenopausal | 2.36 |
| MLXIPL | Osteoporosis, Postmenopausal | 2.36 |
| NNT | Osteoporosis, Postmenopausal | 2.36 |
| NTF3 | Osteoporosis, Postmenopausal | 2.36 |
| PLS3 | Osteoporosis, Postmenopausal | 2.36 |
| PRDM1 | Osteoporosis, Postmenopausal | 2.36 |
| RRAS2 | Osteoporosis, Postmenopausal | 2.36 |
| UNC5B | Osteoporosis, Postmenopausal | 2.36 |
| LASP1 | Osteoporosis, Postmenopausal | 2.35 |
| MT1E | Osteoporosis, Postmenopausal | 2.35 |
| NOL3 | Osteoporosis, Postmenopausal | 2.35 |
| LGMN | Osteoporosis, Postmenopausal | 2.34 |
| PDLIM5 | Osteoporosis, Postmenopausal | 2.34 |
| SLIT2 | Osteoporosis, Postmenopausal | 2.34 |
| SPRY4 | Osteoporosis, Postmenopausal | 2.34 |
| NRCAM | Osteoporosis, Postmenopausal | 2.33 |
| PRKN | Osteoporosis, Postmenopausal | 2.33 |
| SORT1 | Osteoporosis, Postmenopausal | 2.33 |
| GAMT | Osteoporosis, Postmenopausal | 2.32 |
| OPRK1 | Osteoporosis, Postmenopausal | 2.32 |
| LCP1 | Osteoporosis, Postmenopausal | 2.31 |
| MSN | Osteoporosis, Postmenopausal | 2.31 |
| SMPD3 | Osteoporosis, Postmenopausal | 2.31 |
| SYNCRIP | Osteoporosis, Postmenopausal | 2.31 |
| DDAH1 | Osteoporosis, Postmenopausal | 2.3 |
| GRIA3 | Osteoporosis, Postmenopausal | 2.3 |
| ITGB5 | Osteoporosis, Postmenopausal | 2.3 |
| NOCT | Osteoporosis, Postmenopausal | 2.3 |
| SERPING1 | Osteoporosis, Postmenopausal | 2.3 |
| FAAH | Osteoporosis, Postmenopausal | 2.29 |
| FECH | Osteoporosis, Postmenopausal | 2.29 |
| GABRG2 | Osteoporosis, Postmenopausal | 2.29 |
| MVK | Osteoporosis, Postmenopausal | 2.29 |
| NFIA | Osteoporosis, Postmenopausal | 2.29 |
| RPS27L | Osteoporosis, Postmenopausal | 2.29 |
| DBI | Osteoporosis, Postmenopausal | 2.28 |
| NSDHL | Osteoporosis, Postmenopausal | 2.28 |
| HAVCR1 | Osteoporosis, Postmenopausal | 2.26 |
| SORL1 | Osteoporosis, Postmenopausal | 2.26 |
| ACSS2 | Osteoporosis, Postmenopausal | 2.25 |
| CAMK2B | Osteoporosis, Postmenopausal | 2.25 |
| PER3 | Osteoporosis, Postmenopausal | 2.24 |
| S1PR1 | Osteoporosis, Postmenopausal | 2.24 |
| SLC6A9 | Osteoporosis, Postmenopausal | 2.24 |
| HADHA | Osteoporosis, Postmenopausal | 2.22 |
| MARCKS | Osteoporosis, Postmenopausal | 2.21 |
| PER1 | Osteoporosis, Postmenopausal | 2.21 |
| TUBB2A | Osteoporosis, Postmenopausal | 2.2 |
| GABRA1 | Osteoporosis, Postmenopausal | 2.19 |
| FTL | Osteoporosis, Postmenopausal | 2.17 |
| NR3C2 | Osteoporosis, Postmenopausal | 2.17 |
| TUBB3 | Osteoporosis, Postmenopausal | 2.16 |
| ADAMTS1 | Osteoporosis, Postmenopausal | 2.15 |
| RHOB | Osteoporosis, Postmenopausal | 2.13 |
| CES1 | Osteoporosis, Postmenopausal | 2.08 |
